# Supplementary material for: Docking 14 Million Virtual Isoquinuclidines against the μ and κ Opioid Receptors Reveals Dual Antagonists–Inverse Agonists with Reduced Withdrawal Effects
Source: ACS Cent Sci. 2025 Apr 29;11(5):770–90. doi: 10.1021/acscentsci.5c00052 (PMC12123460; doi:10.1021/acscentsci.5c00052)
Supplement: Supplementary file 1 [file oc5c00052_si_001.pdf]

Supporting Information

Docking 14 million virtual isoquinuclidines against the mu and kappa opioid receptors reveals dual antagonists-inverse agonists with reduced withdrawal effects

Seth F. Vigneron†[1], Shohei Ohno†[2], Joao Braz†[3], Joseph Kim†[1], Oh Sang Kweon[2], Chase Webb[1], Christian B. Billesbølle[1], Karthik Srinivasan[1], Karnika Bhardwaj[3], John J. Irwin[1]*, Aashish Manglik[1]*, Allan I. Basbaum[3]*, Jonathan A. Ellman[2]*, Brian K. Shoichet[1]*

† Authors contributed equally to this work

1. Department of Pharmaceutical Chemistry, University of California, San Francisco.
2. Department of Chemistry, Yale University
3. Department of Anatomy, University of California, San Francisco

* Corresponding authors:
jir322@gmail.com, Aashish.Manglik@ucsf.edu, Allan.Basbaum@ucsf.edu, bshoichet@gmail.com, jonathan.ellman@yale.edu

Table of Contents

1. **Supplementary Tables and Figures……………………………………………………..…S2**
2. **General Synthesis Sequence……………………………………………………………..…S6**
3. **General Procedures………………………………………………………………………S6**
4. **Initial Virtual Screening Hits (#03-#64): Synthesis Procedures and Analytical Data.…….S9**
5. **Analogs Based upon Screening Hits (#001-#036): Synthesis Procedures and Analytical Data…………………………………………………………………………………………S35**
6. **X-ray crystallography of Compound #020_E2……………………………………………..S84**
7. **Pharmacokinetic Report of Compound (±)-#020…………………………………………..S92**
8. **CryoEM Validation Statistics……………………………………………………………..S93**
9. **References…………………………………………………………………………………..S95**
10. **NMR spectra of all synthesized compounds……………………………………………….S97**

1. Supplementary Tables and Figures

Reaction Class	Reaction Codes	Reaction Class	Reaction Codes
Amide Coupling	11, 22, 58, 527, 1606, 1626, 2740, 188690, 240690, 269946, 270006, 270062, 270112, 271144, 272270, 272430, 272610, 273012, 273290, 273450, 273652, 273692, 274079, 274614, 274860, 276010, 276750, 280130, 280190	Acrylamide Furnishing	273270
Multi-Amide Coupling	274552, 275592	Purine Formation	275054
Urea Coupling	68, 487, 512, 2430, 2554, 2708, 272164, 273390, 273392, 273452, 273454, 273458, 273464, 273574, 274080	Pyrrole Formation	274092
Reductive Amination	207, 269862, 270004, 270302, 271302, 271304, 272690, 273456, 274370, 275532, 275550, 279530	Imine Formation	4
Esterification	1458, 276436, 279392	Condensation Product	6, 28, 46
Carbamate Coupling	274081, 282070	Oxazolidinone Formation	10
Amine Alkylation	27, 38, 44, 61, 88, 2230, 269956, 269982, 270034, 270122, 270166, 275550, 280170, 280178, 280530, 281630, 282490	Biginelli Product	12, 276390, 276950
Multi-Amine Alkylation	282030	Enamine Formation	17, 570
Sulfonamide Coupling	20, 40, 1606, 196680, 232682, 270084, 270188, 271082, 273578, 274078, 278578, 280530, 282270	Mannich Product	26
Multi-Sulfonamid Coupling	271242	Thiodiazine Formation	41
Sulfonic Esterification	87	Quinoline Formation	42
Oxamide Coupling	2718, 271948, 271949, 273460, 273462, 273492, 273494, 273496, 273498	Cyclic Hydrazine Formation	49, 52, 279312
Suzuki Coupling	271570, 273584, 273712, 275652	Benzofurane Formation	55
Thiazole Formation	1, 2, 3, 24, 43, 50, 272150, 274972, 276096	Benzoimidazole Formation	60
Oxadiazole Formation	265764, 270036, 270196, 270690, 271722, 271362, 274072	Sulfide Product	62
Oxadiazoline Formation	274990	Epoxide Opening or Michael Adduct	51, 63, 273610, 273790, 277110
1,2,4-Triazole Formation	270942, 274052, 276090, 276630, 276670, 276910, 279370	Tetrahydroquinazolone Formation	66
Click 1,2,3-Triazole Formation	273910, 274090, 274860, 274952, 274972, 275090, 275530, 275534, 275536, 276010, 280190, 282512	Thiourea Formation	8
Nuc-Halide SN2	7, 34, 270344, 272692, 272710, 273580, 273654, 280172	Cyclic Thiourea Formation	248
Piperazindione Formation	276530	Pyrimidine-4-one	708
Tetrazole Formation	275630, 277030, 280130, 280172, 280178, 281630, 282490, 280170	Cyclic Imide Formation	1070
Pyrrolidinone Formation	279390	Sulfoxide Product	1498, 265282
Dihydrouracyl Formation	276550, 277130	Sulfone Product	1500, 2714
Aryldiazepinone Formation	276770	Aminoquinazoline Formation	1982
Diazepindione_Formation	279770	Guanidine Product	58668, 264724, 264822, 275310, 276492
Bargellini Reaction	276110, 277030	Activated Methylene Product	194680
Hydrotoin Formation	272104, 272126, 272212	Uracyl Formation	270192
Hydantoinic Acid Formation	272230, 272310, 272330	Thiazolidinone-thione Formation	571

Table S1. Full list of reaction codes used to synthesize the Enamine REAL make-on-demand library categorized into their respective reaction classes.

Synthon Class	Inclusion SMARTS	Exclusion SMARTS
**Applied to all Synthons**	n/a	[SH1] [CX4]S S~S S[F,Cl,Br,I] C[Br,I] [CX4]Cl N[F,Cl,Br,I] [B] [*]=C=[*] N=[CX3,cX3] [Si] N#[CX1] [N,n]~[O,o,s] [H]C([H])=C([H])[CX4,c] [H]C([H])=C([CX4,c,Cl])[CX4,c] [Cl]C([H])=C([H])[CX4,c,Cl] [CX4]([O,N,S,n,+n])[O,N,n,S] [OH,NH2]cc[OH,NH2] [OH,NH2]caac[OH,NH2] NC#N [C]OS(=O)=O [N,n][N,n][C,c]=O [O,N]C=C P[H] [P+] [C-] [c-] N1=NC1 O1CC1 [C,c](=S)[N,n] [F,Cl,Br,I]C=O C=CC=C C(=O)C(=O) C(=O)CC(=O) C(=O)C=CC(=O) C(=O)([CH])C=CC(=O) [NX3,OX2]CCC=O [NX3,OX2]CC=CC=O [H]C([CX4,c,O,N,n,S,s])=O [CX4,c]C(=O)[CX4,c] N=[+N]=[-N] [+n] C(=O)C(F)(F)[H] [CX4,c][NH1][CX4,c]
**Amines**	[C][N]([H])[H] [C]N([H])C(OC(C)(C)C)=O	[c,O,N,n,S][N]([H])[H] [CH0][N]([H])[H] C=CC[N]([H])[H] [CX4]N([H])[CX4] [H]N([H])CC(=O)[NX3&H1] [nX2,o,s][c,n]C[N]([H])[H] c(=O)[c,n]C[N]([H])[H] c(O)[c,n]C[N]([H])[H] O=CN([H])[H] [C,c](=O)[C,c]=[C,c] C#C
**Anilines**	[c][N]([H])[H] [c]N([H])C(OC(C)(C)C)=O	[C,O,N,n][N]([H])[H] [CX4]N([H])[CX4] [H]N([H])cc(=O) O=CN([H])[H] [H]N([H])c([n])[n,cH0&R1] [H]N([H])c([cH0,NH0])[cH0,NH0] [C,c](=O)[C,c]=[C,c] C#C [H]N(c1ncncn1)[H] [H]N(c1nnncc1)[H] [H]N(c1cnnnc1)[H] [H]N(c1nnccn1)[H] [H]N(c1nncnc1)[H] [H]N(c1cnncn1)[H] [nX3]aac(=[S,O,N]) [nX3]c(=[S,O,N])cN [nX3]c(=[S,O,N])cN [H]N([H])ca[nX3]c(=[S,O,N]) [CX4][NH1]c
**Enone**	[C]C(=O)C([C,a])=C([H])[H] [H]C(=O)C([C,a])=C([H])[H] [C]C(=O)/C=C([H])/[C,a,F] [H]C(=O)/C=C([H])/[C,a,F]	C(=O)\C=C([H])/[C,a] [C,c,O,N,n][N]([H])[H] [C;R](=O)/[C;R]=[C;R]([H])/[C,a] C=CC(C=C)=O C(=O)CC=C C#C [C]C(=O)/C([H])=C([H])/[C] [a]C(=O)C=C [CX4&H0]C(=O)C=C C(=O)C([CX4&H0,Cl,F,CX2])=C C(=O)C=C([CX4&H0,Cl,CX2]) C(=O)C=Cnn [C;r4](=O)C=C
**Internal Alkyne**	C#C	[C,c,O,N,n][N]([H])[H] [C,c](=O)[C,c]=[C,c] C#C[H] C#C[CX4&H0,O,N,S,F,Cl] C#CC(=O)[C,c] C#CC(=O)[H] C#C[CX4][OH,NX4&H0] C#C[CH2]OC=O C#CC=C C(=O)C#CC(=O) C#CC#N [NX3]CC#N C(=O)C#N C(=O)CC#N

Table S2. Inclusion and Exclusion SMARTS patterns used for each synthon type for reaction compatibility filtering of building blocks. All Inclusion and Exclusion SMARTS used to generate the isoquinuclidine library can be found at the Chemistry Commons: https://commons.docking.org/reaction/CC-201

Alkyne Pattern	Reaction SMARTS Used	Reaction ID
[*:5]C#C[*:6]	[*:1][N]([H])[H].[*:2]C(=O)C([*:3])=C([H])[*:4].[*:5]C#C[*:6].[H]C([H])=C([H])[*:7]>>[*:1]N1[C@H]([*:6])[C@]2([*:5])C([*:4])=C([*:3])[C@]1([C@@H]([*:7])C2)[*:2]'	CC-201
	'[*:1][N]([H])[H].[*:2]C(=O)C([*:3])=C([H])[*:4].[*:5]C#C[*:6].[H]C([H])=C([H])[*:7]>>[*:1]N1[C@@]([*:2])([C@H]([*:7])C2)C([*:3])=C([*:4])[C@]2([*:5])[C@@H]1[*:6]'	CC-202
[a:5]C#C[A:6]	[*:1][N]([H])[H].[*:2]C(=O)C([*:3])=C([H])[*:4].[*:5]C#C[*:6].[H]C([H])=C([H])[*:7]>>[*:1]N1[C@H]([*:5])[C@]2([*:6])C([*:4])=C([*:3])[C@]1([C@@H]([*:7])C2)[*:2]'	CC-203
	[*:1][N]([H])[H].[*:2]C(=O)C([*:3])=C([H])[*:4].[*:5]C#C[*:6].[H]C([H])=C([H])[*:7]>>[*:1]N1[C@@]([*:2])([C@H]([*:7])C2)C([*:3])=C([*:4])[C@]2([*:6])[C@@H]1[*:5]'	CC-204
[*:5]C(C#C[A:6])=O	[*:1][N]([H])[H].[*:2]C(=O)C([*:3])=C([H])[*:4].[a:5]C#C[A:6].[H]C([H])=C([H])[*:7]>>[*:1]N1[C@H]([A:6])[C@]2([a:5])C([*:4])=C([*:3])[C@]1([C@@H]([*:7])C2)[*:2]'	CC-205
	'[*:1][N]([H])[H].[*:2]C(=O)C([*:3])=C([H])[*:4].[a:5]C#C[A:6].[H]C([H])=C([H])[*:7]>>[*:1]N1[C@@]([*:2])([C@H]([*:7])C2)C([*:3])=C([*:4])[C@]2([a:5])[C@@H]1[A:6]'	CC-206
[CX3:6]=CC#C[CX4:5]	[*:1][N]([H])[H].[*:2]C(=O)C([*:3])=C([H])[*:4].[*:5]C(C#C[A:6])=O.[H]C([H])=C([H])[*:7]>>[*:1]N1[C@H]([*:6])[C@]2(C([*:5])=O)C([*:4])=C([*:3])[C@]1([C@@H]([*:7])C2)[*:2]'	CC-207
	'[*:1][N]([H])[H].[*:2]C(=O)C([*:3])=C([H])[*:4].[*:5]C(C#C[A:6])=O.[H]C([H])=C([H])[*:7]>>[*:1]N1[C@@]([*:2])([C@H]([*:7])C2)C([*:3])=C([*:4])[C@]2(C([*:5])=O)[C@@H]1[A:6]'	CC-208
[CX3:6]=C([H])C#C[CX4:5]	[*:1][N]([H])[H].[*:2]C(=O)C([*:3])=C([H])[*:4].[CX3:6]=C([H])C#C[CX4:5].[H]C([H])=C([H])[*:7]>>[*:1]N1[C@H](C=[CX3:6])[C@]2([CX4:5])C([*:4])=C([*:3])[C@]1([C@@H]([*:7])C2)[*:2]'	CC-209
	'[*:1][N]([H])[H].[*:2]C(=O)C([*:3])=C([H])[*:4].[CX3:6]=C([H])C#C[CX4:5].[H]C([H])=C([H])[*:7]>>[*:1]N1[C@@]([*:2])([C@H]([*:7])C2)C([*:3])=C([*:4])[C@]2([CX4:5])[C@@H]1C=[CX3:6]'	CC-210

Table S3. Reaction SMARTS used to combine building blocks into final isoquinuclidines. The substituents on the internal alkyne building block dictate it's regiochemistry in the final product. For every combination of amine, enone, alkyne and alkene, both the plus and minus enantiomers were generated with the regiochemistry dictated by the alkyne. All reaction used SMARTS, along with their associated inclusion and exclusion SMARTS patterns, are available at the Chemistry Commons under reaction IDs CC-201 – CC-210 (https://commons.docking.org/reaction/CC-201)

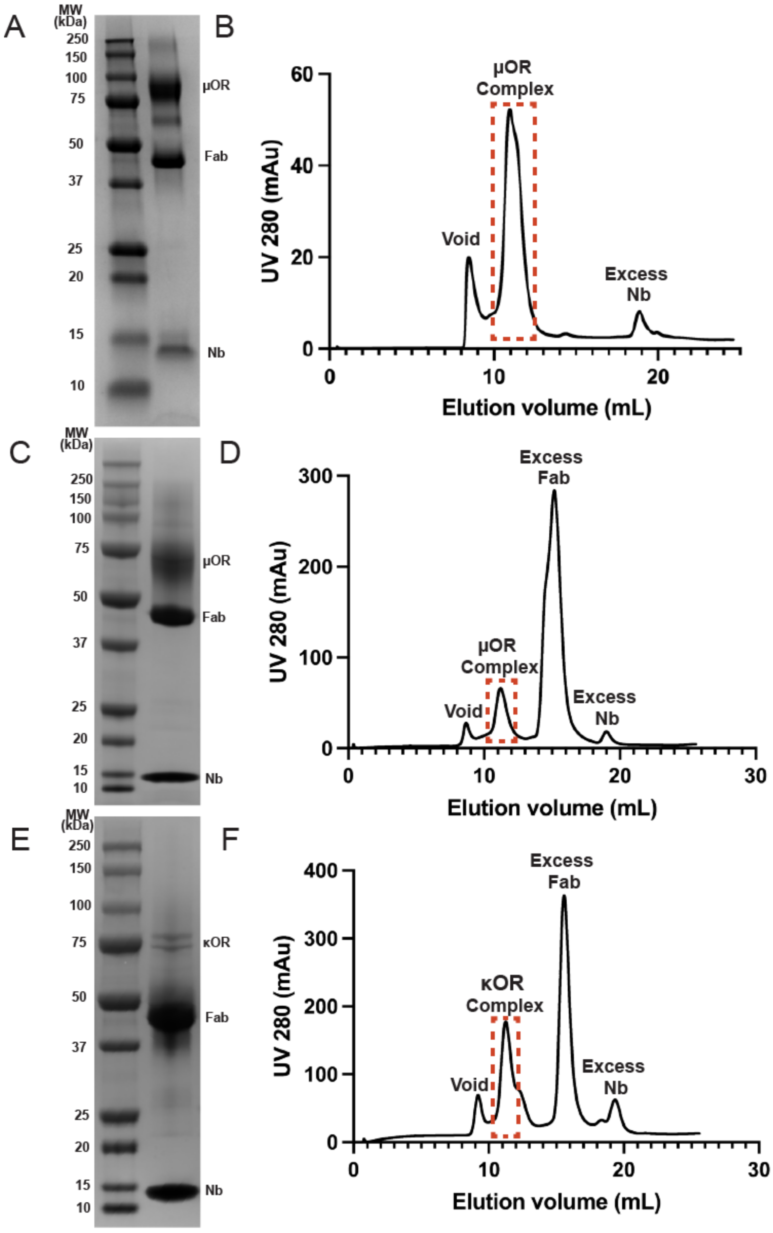

Figure S1: Biochemistry of purified MOR and KOR used for cryoEM. A. Cropped SDS-PAGE of MOR complexed with compound **#33**, Nb6M, NabFab, and Anti-Fab Nanobody. The MOR was mutated at the ICL3 to enable binding to Nb6M. **B.** Final size-exclusion chromatogram (SEC) profile of the MOR complex. The fractionated peaks that corresponds to the MOR complex (dotted red box) were immediately collected and concentrated prior to cryoEM sample preparation. **C.** Cropped SDS-PAGE of MOR complexed with compound **#020_E1**, Nb6M, NabFab, and Anti-Fab Nanobody. **D.** Final size-exclusion chromatogram (SEC) profile of the MOR complex. The fractionated peaks that corresponds to the MOR complex (dotted red box) were immediately collected and concentrated prior to cryoEM sample preparation. **E.** Cropped SDS-PAGE of KOR

complexed with compound **#020_E1**, Nb6M, NabFab, and Anti-Fab Nanobody. **F.** Final SEC profile of the KOR complex. The fractionated peaks that corresponds to the KOR complex (dotted red box) were immediately collected and concentrated prior to cryoEM sample preparation.

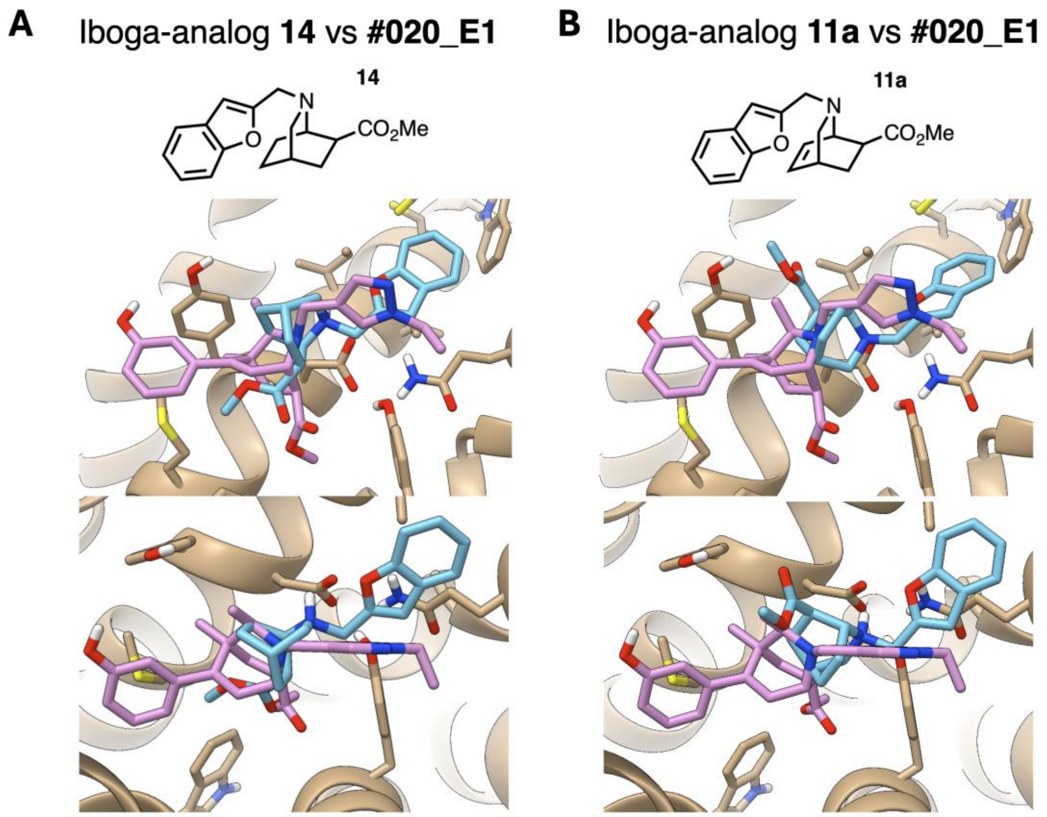

Figure S2: Overlay of the MOR docked pose of previously reported iboga-analogs **14** (**A**, blue) and **11a** (**B**, blue) to the cryoEM pose of **#020_E1** (pink, PDB: 9MQJ) from a side angle (top) and from above (bottom). In both cases, the binding pose of the iboga-analogs form similar poses to that of **#020_E1**, with minor differences in translation of the isoquinuclidine core. For **B**, iboga-analog **11a** also exhibits a rotation of the core, placing the methyl ester group point up towards the solvent exposed portion of the binding site, while maintaining a similar placement of the amine substituent.

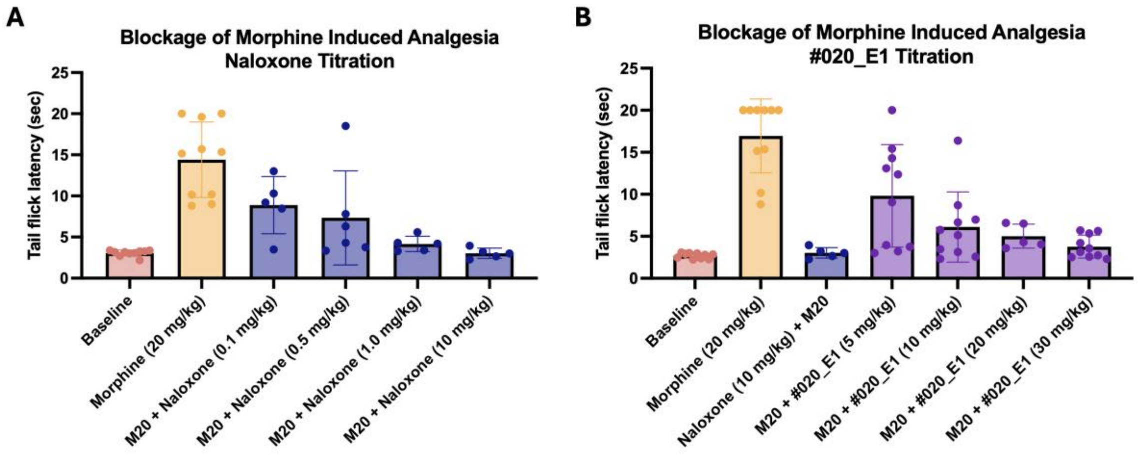

Figure S3: Dose response of increasing levels of naloxone (**A**) or **#020_E1** (**B**) in the blocking of 20mg/kg morphine induced analgesia. An i.p. dose of 30mg/kg **#020_E1** is required to return tail flick latency to baseline levels, comparable to that of a 10mg/kg dose of naloxone. Data presented is mean ± SD from n=5-10 mice.

2. SCHEME S1. General Synthesis Sequence

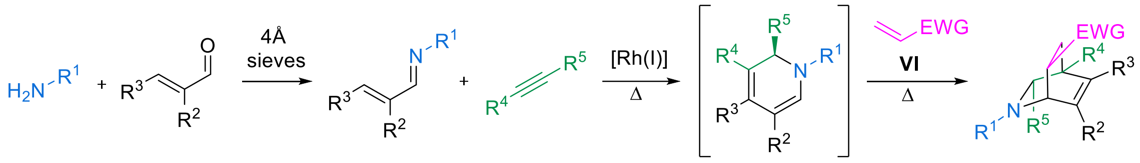

3. GENERAL PROCEDURES

Preparation of Rhodium Catalyst Stock Solution

$[RhCl(coe)_2]_2$ was purchased from Strem and stored inside a N_2-filled inert atmosphere glovebox at -25 ºC. Stock solutions of the rhodium catalyst were prepared in the glovebox. For the preparation of a 100 mM solution in THF, a 5 mL glass vial was charged with $[RhCl(coe)_2]_2$ (100 mg, 0.139 mmol) and p-Me_2N-C_6H_4-PEt_2 (58 mg, 0.278 mmol) in anhydrous THF (1.4 mL). Stock solutions, when stored in a –25 ºC freezer inside a N_2-filled glovebox, could be used for several weeks without loss of catalytic activity.

General Procedure A for the synthesis of imines III

To a flame-dried round-bottom flask was added a magnetic stir bar, amine **I** (1 equiv), enal **II** (1-5 equiv), dry THF (0.5 M), and 20 vol% of molecular sieves 4 Å. The reaction solution was stirred under N_2 at rt for 12 h. Upon reaction completion, the reaction

mixture was filtered through a pad of celite. The filtrate was concentrated in vacuo to provide the desired imine **III**, which was stored at –20 °C in a nitrogen filled glovebox and taken on to the next step without further purification.

General Procedure B for the synthesis of DHPs V

Inside a N_2-filled inert atmosphere glovebox, imine **III** (1.0 equiv) was dissolved in anhydrous THF (0.5 M total concentration), and the solution was transferred to a flame-dried sealable glass vessel equipped with a magnetic stir bar. A stock solution of Rh catalyst (see General Information) at the catalyst loading indicated was added to the tube, followed by alkyne **IV** (2-5 equiv). A small portion (~0.4 mL) of this reaction mixture was transferred to an oven-dried J. Young NMR tube that was equipped with a flame-sealed melting point capillary containing benzene-d_6 for reaction monitoring. The J. Young NMR tube and glass vessel were then sealed, removed from the glovebox, and heated at the indicated temperature. The reaction progress was monitored by ^{1}H NMR spectroscopy based on the disappearance of the imine **III** signals and the increasing resonances of the dihydropyridine **V**. Upon reaction completion, this crude DHP solution was used for the synthesis of isoquinuclidines **VII** (General Procedure C).

General Procedure C for the synthesis of isoquinuclidines VII from DHPs V via the Diels–Alder reaction

To the reaction vessel including the crude DHP **V** solution was added the desired alkene **VI** (10 equiv). The reaction vessel was then sealed, and the mixture was stirred at the indicated temperature. After reaction completion, the reaction vessel was opened, and the solvent was removed in vacuo. The resulting residue was purified by column chromatography on silica gel or preparative thin-layer chromatography to afford the desired isoquinuclidine **VII**.

General Procedure D for the cleavage of acid-labile protecting groups from isoquinuclidines VII

To an oven-dried round-bottom flask equipped with a magnetic stir bar was added the isoquinuclidine **VII** (1.0 equiv) in CH_2Cl_2 (0.1 M). The reaction mixture was cooled to 0 °C, and trifluoroacetic acid (10.0 equiv) was added. The ice bath was removed and the reaction mixture was stirred at room temperature until the reaction was complete as monitored by thin-layer chromatography. The reaction mixture was then diluted with CH_2Cl_2 and saturated $NaHCO_3$ (aq) and extracted three times with CH_2Cl_2. The combined organic layers were washed with brine, dried with Na_2SO_4, filtered and concentrated in

vacuo. The crude material was purified by the indicated chromatographic method to afford the desired product.

General Procedure E for the cleavage of *p*-methoxybenzyl group on nitrogen

To a flame-dried round-bottom flask equipped with a magnetic stir bar was added isoquinuclidine **VII** (1.0 equiv) and dichloroethane (0.2 M). The reaction mixture was cooled to 0 °C, and 1-chloroethyl chloroformate (5 equiv) was added. The mixture warmed to room temperature and was stirred at rt until complete as monitored by thin-layer chromatography. The reaction mixture was then concentrated in vacuo, and the crude material was purified by silica gel chromatography with only pure fractions isolated to afford the carbamate intermediate. To a flame-dried round-bottom flask was added the purified intermediate followed by excess MeOH, and the reaction solution was stirred at 40 °C for 12 h. Upon reaction completion, the crude material was purified by the indicated chromatographic method to afford the desired product.

4. Initial Virtual Screening Hits (#03-#64): Synthesis Procedures and Analytical Data

Synthesis of #03

・**Synthesis of Aldehyde**

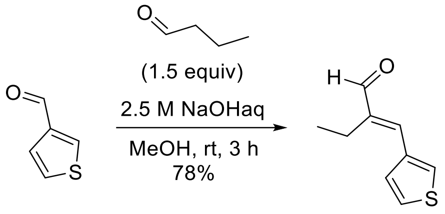

To a round-bottom flask was added a magnetic stir bar, thiophene-3-carbaldehyde (1 g, 8.92 mmol, 1 equiv) and butanal (1.21 mL, 13.4 mmol, 1.5 equiv) dissolved in MeOH (9 mL). To this solution was then added sodium hydroxide (2.5 M aq., 3.56 mL, 1.0 equiv) dropwise at 0 °C. The reaction mixture was stirred at room temperature for 3 h. After the reaction was complete as monitored by thin-layer chromatography, the mixture was then diluted with water, extracted with Et_2O, washed with brine, dried with Na_2SO_4 and filtered. The organic layer was concentrated and the crude residue was purified via silica gel chromatography (gradient of 0-100% EtOAc in hexane) to give 1.15 g of desired aldehyde in 78% yield. ^{1}H NMR (400 MHz, $CDCl_3$) δ 9.48 (s, 1H), 7.60 (dd, *J* = 3.0, 1.3 Hz, 1H), 7.40 (dd, *J* = 5.1, 2.9 Hz, 1H), 7.32 (dd, *J* = 5.1, 1.3 Hz, 1H), 7.16 (s, 1H), 2.57 (q, *J* = 7.6 Hz, 2H), 1.11 (t, *J* = 7.5 Hz, 3H). ^{13}C NMR (101 MHz, $CDCl_3$) δ 195.2, 142.7, 142.6, 136.7, 128.8, 128.3, 126.6, 18.1, 12.5. HRMS (ESI+, m/z) calcd for $C_9H_{11}OS$ $(M+H)^+$: 167.0525, found: 167.0529.

・**Synthesis of Imine**

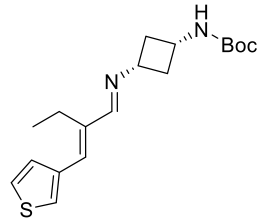

General procedure A was followed using above obtained aldehyde (200 mg, 1.20 mmol, 1.0 equiv), *cis*-*tert*-butyl *N*-(3-aminocyclobutyl)carbamate (246 mg, 1.32 mmol, 1.1 equiv), molecular sieves 4 Å, and THF (2.4 mL). Imine (370 mg, 99% yield) was obtained as a white solid. ^{1}H NMR (400 MHz, CD_3OD) δ 7.82 (d, *J* = 1.3 Hz, 1H), 7.52 (d, *J* = 2.4 Hz, 1H), 7.44 (dd, *J* = 5.1, 2.9 Hz, 1H), 7.26 (dd, *J* = 5.0, 1.3 Hz, 1H), 6.81 (s, 1H), 3.86 (p, *J* = 8.4 Hz, 1H), 3.74 (p, *J* = 7.9 Hz, 1H), 2.75 – 2.52 (m, 4H), 1.99 (qd, *J* = 9.0, 2.8 Hz, 2H), 1.42 (s, 9H), 1.11 (t, *J* = 7.5 Hz, 3H).

・**Key Reaction**

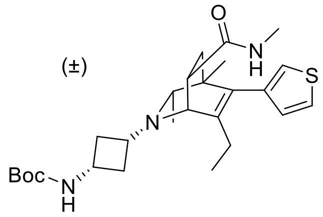

General procedure B was followed using above obtained imine (80 mg, 0.240 mmol, 1.0 equiv) and 2-butyne (94 μL, 1.20 mmol, 5 equiv) in THF (0.24 mL). Rh catalyst (10 mol %, 0.24 mL, 0.024 mmol, 100 mM in THF) was added, and the reaction was carried out at 65 °C for 12 h to produce the DHP intermediate. Following general procedure C, *N*-methyl acryamide (204 mg, 2.40 mmol, 10 equiv) was added to the solution of DHP intermediate, and the reaction was carried out at 90 °C for 16 h. The resulting crude material was purified by column chromatography (5% MeOH/CH_2Cl_2 + 1% NH_4OH) to afford the desired product (26 mg, 23% yield from imine) as a white solid. ^{1}H NMR (400 MHz, CD_3OD) δ 7.34 (dd, *J* = 4.9, 2.9 Hz, 1H), 7.00 (dd, *J* = 2.9, 1.2 Hz, 1H), 6.87 (dd, *J* = 4.9, 1.2 Hz, 1H), 3.74 (p, *J* = 8.5 Hz, 1H), 3.45 (d, *J* = 3.0 Hz, 1H), 3.02 (dt, *J* = 8.9, 4.1 Hz, 1H), 2.94 (p, *J* = 8.5, 7.9 Hz, 1H), 2.66 (s, 3H), 2.65 – 2.57 (m, 1H), 2.53 – 2.41 (m, 1H), 2.19 (q, *J* = 6.3 Hz, 1H), 1.96 – 1.85 (m, 3H), 1.74 (q, *J* = 9.7 Hz, 1H), 1.49-1.34 (m, 11H), 0.97 (d, *J* = 6.2 Hz, 3H), 0.84 (t, *J* = 7.6 Hz, 3H), 0.82 (s, 3H). HRMS (ESI+, m/z) calcd for $C_{26}H_{40}N_3O_3S$ $(M+H)^+$: 474.2785, found: 474.2791.

・**Deprotection**

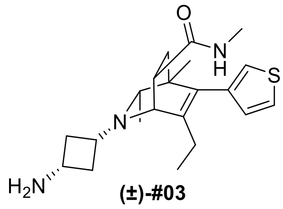

(±)-#03

Final Compound #03 General Procedure D was followed using protected isoquinuclidine (26 mg, 0.055 mmol, 1.00 equiv) in CH_2Cl_2 (1.0 mL) and was reacted with trifluoroacetic acid (41 μL, 0.55 mmol, 10.0 equiv) for 12 h. The crude product was purified by preparative thin-layer chromatography (10% MeOH/CH_2Cl_2 + 1% NH_4OH) to yield the racemic isoquinuclidine (16 mg, 78% yield) as a pale-yellow oil. ^{1}H NMR (400 MHz, CD_3OD) δ 7.35 (dd, *J* = 4.9, 3.0 Hz, 1H), 7.00 (dd, *J* = 3.0, 1.2 Hz, 1H), 6.87 (dd, *J* = 4.9, 1.2 Hz, 1H), 3.47 (d, *J* = 3.0 Hz, 1H), 3.12 (p, *J* = 8.3 Hz, 1H), 3.01 (dt, *J* = 8.7, 4.0 Hz, 1H), 2.91 (p, *J* = 7.9 Hz, 1H), 2.68 – 2.59 (m, 1H), 2.66 (s, 3H), 2.55 – 2.33 (m, 1H), 2.18 (q, *J* = 6.3 Hz, 1H), 2.09 – 1.76 (m, 4H), 1.66 (q, *J* = 9.6 Hz, 1H), 1.39 (dd, *J* = 12.8, 9.4 Hz, 1H), 0.98 (d, *J* = 6.2 Hz, 3H), 0.84 (t, *J* = 7.8 Hz, 3H), 0.82 (s, 3H). ^{13}C NMR (101 MHz, CD_3OD) δ 174.8, 141.2, 138.5, 134.8, 128.5, 124.1, 121.8, 62.9, 56.7, 50.2, 41.5, 40.4, 39.3, 38.7, 38.3, 35.3, 25.3, 24.5, 20.6, 19.9, 11.4. HRMS (ESI+, m/z) calcd for $C_{21}H_{31}N_3OS$ $(M+H)^+$: 374.2261, found: 374.2248.

Synthesis of #05

・**Synthesis of Imine**

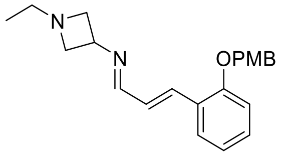

General procedure A was followed using (*E*)-3-(2-((4 methoxybenzyl)oxy)phenyl)acrylaldehyde[1] (121 mg, 0.449 mmol, 0.9 equiv), 1-ethylazetidin-3-amine (50 mg, 0.50 mmol, 1.0 equiv), molecular sieves 4 Å, and THF (1.0 mL). Imine (175 mg, 99% yield) was obtained as pale-yellow oil. ^{1}H NMR (400 MHz, $CDCl_3$) δ 7.96 (d, J = 8.9 Hz, 1H), 7.55 (dd, J = 8.1, 1.7 Hz, 1H), 7.37 – 7.33 (m, 2H), 7.28 – 7.24 (m, 2H), 7.19 – 7.15 (m, 1H), 6.97 – 6.91 (m, 4H), 5.05 (s, 2H), 4.23 (p, J = 6.7 Hz, 1H), 3.83 (m, 5H), 3.17 (t, J = 7.3 Hz, 2H), 2.62 (q, J = 7.2 Hz, 2H), 1.04 (t, J = 7.2 Hz, 3H).

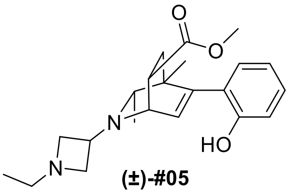

(±)-#05

Final Compound #05 Following General Procedure B for the formation of 1,2-dihydropyridines, the reaction was set up with above obtained imine (200 mg, 0.571 mmol, 1.00 equiv), Rh stock solution (857 µL, 85.7 µmol, 15 mol %), 2-butyne (447 µL, 5.71 mmol, 10.0 equiv), and a reaction time of 20 h at 90 ºC. Isoquinuclidine formation was conducted according to General Procedure C with methyl acrylate (517 µL, 5.71 mmol, 10.0 equiv) for 16 h at 90 ºC. After reaction completion, the solvent was removed in vacuo and the crude isoquinuclidine product was carried forward to deprotection without purification. Following General Procedure D, CH_2Cl_2 (4.76 mL) was added to the crude protected isoquinuclidine and was reacted with trifluoroacetic acid (437 µL, 5.71 mmol, 10.0 equiv) for 10 h. The crude product was purified by silica gel chromatography (5% MeOH/CH_2Cl_2 + 1% NH_4OH) to yield two isomers of the final product. Isomer 1 (50 mg, 24% yield over three steps) and Isomer 2 (12 mg, 5.7% yield over three steps) were isolated as pale-yellow oil.

Isomer 1

^{1}H NMR (500 MHz, $CDCl_3$) δ 7.16 (td, *J* = 7.7, 1.7 Hz, 1H), 7.00 (dd, *J* = 7.6, 1.7 Hz, 1H), 6.89 – 6.84 (m, 2H), 6.55 (d, *J* = 6.4 Hz, 1H), 3.90 (dd, *J* = 6.4, 1.8 Hz, 1H), 3.76 (s, 3H), 3.52 – 3.47 (m, 3H), 2.87 – 2.84 (m, 1H), 2.73 (m, 1H), 2.64 – 2.57 (m, 2H), 2.48 (m, 2H), 2.18 (dd, *J* = 13.3, 5.7 Hz,

1H), 1.78 (dd, *J* = 13.3, 11.3 Hz, 1H), 0.99 – 0.95 (m, 6H), 0.91 (d, *J* = 6.4 Hz, 3H). ^{13}C NMR (126 MHz, $CDCl_3$) δ 174.5, 152.9, 143.8, 133.8, 129.5, 128.6, 126.1, 119.8, 115.3, 63.8, 61.7, 59.8, 53.6, 52.0, 51.9, 51.8, 45.3, 41.8, 34.8, 20.6, 18.5, 12.4. HRMS (ESI+, m/z) $[M+H]^+$ calcd: 371.2329, found: 371.2323

Isomer 2

^{1}H NMR (500 MHz, $CDCl_3$) δ 7.17 (td, *J* = 7.7, 1.7 Hz, 1H), 6.98 (dd, *J* = 7.6, 1.7 Hz, 1H), 6.93 (d, *J* = 8.1 Hz, 1H), 6.84 (d, *J* = 7.3 Hz, 1H), 6.25 (d, *J* = 6.4 Hz, 1H), 3.74 – 3.69 (m, 5H), 3.61 (dd, *J* = 6.4, 3.3 Hz, 1H), 3.54 – 3.49 (m, 1H), 3.24 (m, 1H), 2.99 (t, *J* = 7.0 Hz, 1H), 2.92 (t, *J* = 6.1, 4.9 Hz, 1H), 2.58 (m, 2H), 2.20 (q, *J* = 6.4 Hz, 1H), 1.88 (dd, *J* = 13.6, 3.6 Hz, 1H), 1.58 (dd, *J* = 13.5, 9.2 Hz, 1H), 1.04 – 1.00 (m, 6H), 0.90 (s, 3H). ^{13}C NMR (126 MHz, $CDCl_3$) δ 177.3, 154.0, 144.4, 130.5, 128.8, 128.8, 119.3, 116.3, 113.8, 77.3, 77.0, 76.8, 63.1, 60.3, 59.0, 53.0, 52.7, 52.1, 39.3, 36.0, 20.9, 20.2, 12.0. HRMS (ESI+, m/z) $[M+H]^+$ calcd: 371.2329, found: 371.2324

Synthesis of #06

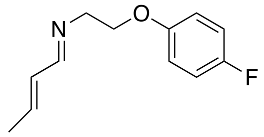

General Procedure A was followed using 2-(4-fluorophenoxy)ethan-1-amine (100 mg, 0.644 mmol, 1.00 equiv), *trans*-crotonaldehyde (0.26 mL, 3.22 mmol, 5.00 equiv), K_2CO_3 (31.2 mg, 0.225 mmol, 0.35 equiv), and THF (1.29 mL) for 4 h at rt. The product imine was obtained as pale-yellow oil (133 mg, 100%). ^{1}H NMR (400 MHz, $CDCl_3$) δ 7.92 (m, 1H), 6.96 – 6.92 (m, 2H), 6.86 – 6.82 (m, 2H), 6.25 (m, 2H), 4.15 (t, *J* = 5.6 Hz, 2H), 3.79 (t, *J* = 5.6 Hz, 2H), 1.89 (d, *J* = 5.1 Hz, 3H).

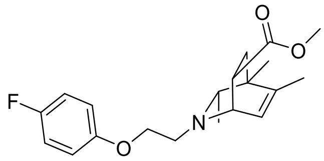

(±)-#06

Final Compound #06 Following General Procedure B for the formation of 1,2-dihydropyridines, the reaction was set up with above obtained imine (50.0 mg, 0.241 mmol, 1.00 equiv), Rh stock solution (241 µL, 24.1 µmol, 10 mol %), 2-butyne (189 µL, 2.41 mmol, 10.0 equiv), and a reaction time of 20 h at 70 ºC. Isoquinuclidine formation was conducted according to the General Procedure C with methyl acrylate (218 µL, 2.41 mmol, 10.0 equiv) for 16 h at 90 ºC. The product after the Diels-Alder reaction was purified by silica gel chromatography (50% EtOAc/hexanes + 1% Et_3N) to afford the isoquinuclidine product (20 mg, 24% yield over two steps) as pale-yellow oil. ^{1}H NMR (400 MHz, $CDCl_3$) δ 6.94 (t, *J* = 8.7 Hz, 2H), 6.82 (m, 2H), 6.01 (d, *J* = 6.0 Hz, 1H), 4.06 (t, *J* = 6.3 Hz, 2H), 3.76

(m, 1H), 3.62 (s, 3H), 3.18 (m, 1H), 2.99 (m, 2H), 2.10 (m, 1H), 1.74 (m, 4H), 1.45 (dd, *J* = 13.0, 9.5 Hz, 1H), 1.08 (s, 3H), 0.85 (d, *J* = 6.3 Hz, 3H). ^{13}C NMR (126 MHz, $CDCl_3$) δ 161.7, 158.2(d, *J* = 5.1 Hz), 156.3, 154.8-154.6 (m), 124.9, 115.9, 115.7, 115.4, 115.4, 68.2, 65.0, 54.9, 53.2, 51.8, 40.3, 37.5, 35.4, 19.8, 19.7, 18.8. ^{19}F NMR (376 MHz, $CDCl_3$) δ -124.08. HRMS (ESI+, m/z) $[M+H]^+$ calcd: 348.1969, found: 348.1966

#09 (mixture of diastereomers)

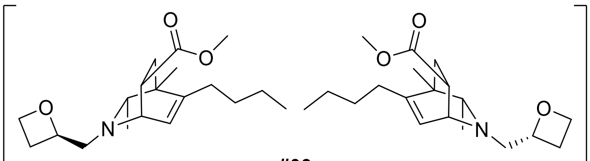

#09
mixture of diastereomers

Final Compound #09 Following General Procedure B for the formation of 1,2-dihydropyridines, the reaction was set up with (1*E*,2*E*)-*N*-(((*R*)-oxetan-2-yl)methyl)hept-2-en-1-imine (175 mg, 0.965 mmol, 1.00 equiv), Rh stock solution (965 µL, 96.5 µmol, 10 mol %), 2-butyne (755 µL, 9.65 mmol, 10.0 equiv), and a reaction time of 14 h at 70 ºC. Isoquinuclidine formation was conducted according to the General Procedure C with methyl acrylate (874 µL, 9.65 mmol, 10.0 equiv) for 16 h at 90 ºC. The product after the Diels-Alder reaction was purified by silica gel chromatography (8% MeOH/CH_2Cl_2 + 1% NH_4OH) to afford the isoquinuclidine product (86 mg, 28% yield over two steps) as a mixture of diastereomers.

^{1}H NMR (500 MHz, $CDCl_3$) δ 5.93 (d, *J* = 6.4 Hz, 1H), 4.99 – 4.90 (m, 1H), 4.65 (m, 1H), 4.51 (m, 1H), 3.68 (dd, *J* = 6.6, 3.2 Hz, 1H), 3.61 (s, 3H), 3.15 (m, 1H), 2.99 (dd, *J* = 13.0, 7.1 Hz, 1H), 2.94 – 2.76 (m, 1H), 2.74 – 2.63 (m, 2H), 2.44 – 2.29 (m, 1H), 2.08 – 1.91 (m, 3H), 1.72 (dd, *J* = 12.9, 4.7 Hz, 1H), 1.40 (m, 3H), 1.31 (m, 3H), 1.07 (s, 3H), 0.88 (t, *J* = 7.1 Hz, 3H), 0.77 (d, *J* = 6.0 Hz, 3H). ^{13}C NMR (126 MHz, $CDCl_3$) δ 175.1, 174.9, 147.5, 146.9, 123.7, 123.1, 82.1, 81.4, 77.3, 77.0, 76.8, 68.3, 68.3, 64.8, 64.3, 61.9, 61.0, 56.0, 54.6, 51.7, 40.5, 40.3, 37.8, 37.5, 35.9, 35.4, 31.3, 29.3, 29.3, 26.5, 26.3, 22.5, 22.5, 19.9, 19.7, 19.5, 14.0. HRMS (ESI+, m/z) $[M+H]^+$ calcd: 322.2377, found: 322.2368

Synthesis of #11

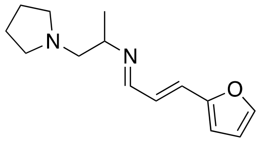

General Procedure A was followed using 1-(pyrrolidin-1-yl)propan-2-amine (200 mg, 1.56 mmol, 1.00 equiv), (*E*)-3-(furan-2-yl)acrylaldehyde (171 mg, 1.40 mmol, 0.9 equiv), 3 Å MS (780 mg), and THF

(3.12 mL) for 4 h at 80 ºC. The product imine was obtained as pale-yellow oil (362 mg, 100%). ^{1}H NMR (400 MHz, $CDCl_3$) δ 7.98 (d, *J* = 8.8 Hz, 1H), 7.43 (s, 1H), 6.84 – 6.67 (m, 2H), 6.48 – 6.40 (m, 2H), 3.40 (m, 1H), 2.64 – 2.58 (m, 2H), 2.51 (m, 4H), 1.73 (m, 4H), 1.20 (d, *J* = 6.4 Hz, 3H).

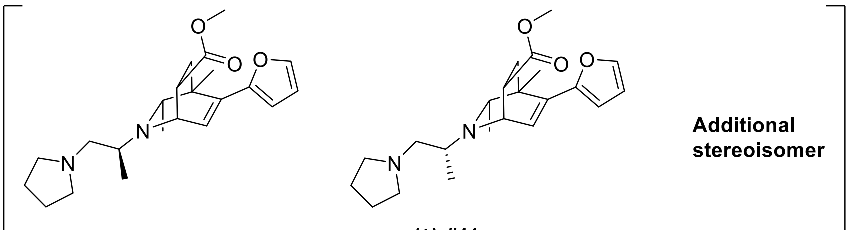

(±)-#11
3 isomers not assigned to specific structures

Final Compound #11 Following General Procedure B for the formation of 1,2-dihydropyridines, the reaction was set up with above obtained imine (300 mg, 1.29 mmol, 1.00 equiv), Rh stock solution (1.94 mL, 0.194 mmol, 15 mol %), 2-butyne (1.01 mL, 12.9 mmol, 10.0 equiv), and a reaction time of 20 h at 90 ºC. Isoquinuclidine formation was conducted according to the General Procedure C with methyl acrylate (1.17 mL, 12.9 mmol, 10.0 equiv) for 16 h at 90 ºC. The product after the Diels-Alder reaction was purified by silica gel chromatography (5% MeOH/CH_2Cl_2 + 1% NH_4OH) to afford three isomers of the isoquinuclidine product. Isomer 1 (135 mg, 28% yield over two steps), Isomer 2 (135 mg, 28% yield over two steps), and Isomer 3 (17 mg, 3.5% yield over two steps) were isolated as pale-yellow oil.

Isomer 1:
^{1}H NMR (500 MHz, $CDCl_3$) δ 7.32 (d, *J* = 1.7 Hz, 1H), 6.59 (d, *J* = 6.6 Hz, 1H), 6.31 (m, 2H), 4.17 (dd, *J* = 6.7, 3.1 Hz, 1H), 3.62 (m, 4H), 3.23 (dt, *J* = 9.9, 3.8 Hz, 1H), 2.80 (m, 1H), 2.65 (dd, *J* = 12.1, 5.6 Hz, 1H), 2.54 – 2.46 (m, 4H), 2.35 (m, 1H), 1.95 (dd, *J* = 13.1, 4.3 Hz, 1H), 1.74 (m, 4H), 1.55 (dd, *J* = 13.2, 9.8 Hz, 1H), 1.35 (s, 3H), 1.19 (d, *J* = 6.4 Hz, 3H), 0.89 (d, *J* = 6.0 Hz, 3H). ^{13}C NMR (126 MHz, $CDCl_3$) δ 174.7, 152.8, 141.4, 137.1, 127.8, 110.7, 107.1, 62.8, 62.0, 55.3, 54.7, 51.7, 50.9, 40.7, 39.5, 35.9, 23.5, 22.2, 20.9, 20.1. HRMS (ESI+, m/z) $[M+H]^+$ calcd: 373.2486, found: 373.2477

Isomer 2:
^{1}H NMR (500 MHz, $CDCl_3$) δ 7.32 (m, 1H), 6.56 (d, *J* = 6.7 Hz, 1H), 6.31 (m, 2H), 4.13 (dd, *J* = 7.0, 3.3 Hz, 1H), 3.62 (m, 4H), 3.15 (dt, *J* = 9.7, 3.7 Hz, 1H), 2.60 – 2.45 (m, 6H), 2.27 (m, 1H), 1.98 (dd, *J* = 13.1, 4.1 Hz, 1H), 1.76 (m, 4H), 1.52 (dd, *J* = 13.2, 9.6 Hz, 1H), 1.36 (s, 3H), 1.26 (d, *J* = 6.2 Hz, 3H), 0.90 (d, *J* = 6.0 Hz, 3H). ^{13}C NMR (126 MHz, $CDCl_3$) δ 174.5, 152.6, 141.5, 137.6, 127.0, 110.7, 107.3, 63.7, 61.9, 54.9, 54.7, 51.8, 50.7, 40.7, 35.9, 35.3, 23.5, 22.2, 21.3, 17.5. HRMS (ESI+, m/z) $[M+H]^+$ calcd: 373.2486, found: 373.2491

Isomer 3:

^{1}H NMR (500 MHz, $CDCl_3$) δ 7.34 (d, J = 1.8 Hz, 1H), 6.76 (d, J = 6.2 Hz, 1H), 6.35 (dd, J = 3.3, 1.8 Hz, 1H), 6.30 (d, J = 3.4 Hz, 1H), 3.87 (dd, J = 6.3, 2.0 Hz, 1H), 3.70 (s, 3H), 3.12 (q, J = 6.4 Hz, 1H), 2.97 (m, 1H), 2.59 – 2.41 (m, 7H), 2.13 (dd, J = 13.1, 5.4 Hz, 1H), 1.80 – 1.72 (m, 4H), 1.55 (dd, J = 13.1, 10.9 Hz, 1H), 1.37 (s, 3H), 1.04 (d, J = 6.6 Hz, 3H), 0.86 (d, J = 6.3 Hz, 3H). ^{13}C NMR (126 MHz, $CDCl_3$) δ 174.6, 153.0, 141.3, 135.6, 130.2, 110.9, 106.8, 63.3, 54.4, 51.6, 51.2, 50.8, 46.5, 41.6, 34.5, 29.7, 23.4, 22.1, 19.0, 15.7. HRMS (ESI+, m/z) $[M+H]^+$ calcd: 373.2486, found: 373.2491

Synthesis of #12

・**Synthesis of Amine**

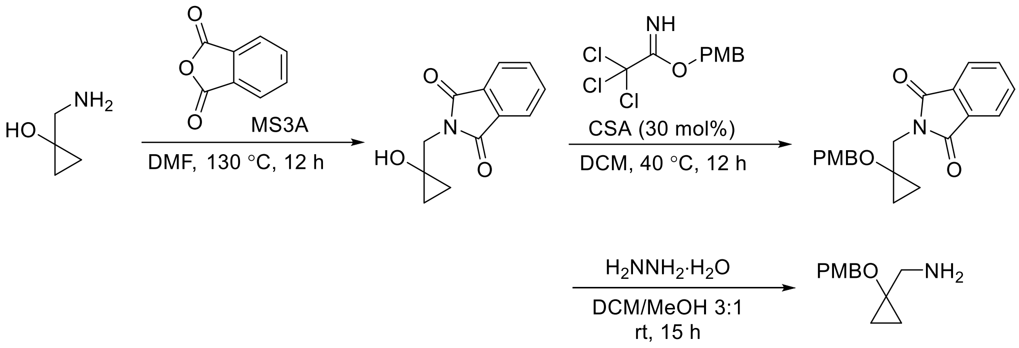

To a flame-dried round-bottom flask was added a magnetic stir bar, 1-(aminomethyl)cyclopropanol (1.0 g, 11.5 mmol, 1.0 equiv) and phthalic anhydride (1.7 g, 11.5 mmol, 1.0 equiv) dissolved in DMF (23 mL). To this solution was then added molecular sieves 4 Å. The reaction mixture was stirred at 130 °C for 12 h. After the reaction was complete as monitored by thin-layer chromatography, the mixture was concentrated and the crude residue was purified via silica gel chromatography (gradient of 0-100% EtOAc in hexane +1% Et_3N) to give 2.2 g of the amine protected compound in 88% yield. To a stirred solution of the above obtained compound (1.0 g, 4.6 mmol, 1.0 equiv) in DCM (23 mL) was added 4-methoxybenzyl-2,2,2-trichloroacetimidate (1431 mg, 5.06 mmol, 1.1 equiv) and 10-camphorsulfonic acid (321 mg, 1.38 mmol, 0.3 equiv). The reaction mixture was stirred at 40 °C for 12 h. After the reaction was complete as monitored by thin-layer chromatography, the mixture was then diluted with saturated $NaHCO_3$ (aq), extracted with DCM, washed with brine, dried with Na_2SO_4 and filtered. The organic layer was concentrated and the crude residue was purified via silica gel chromatography (gradient of 0-100% EtOAc in hexane) to give 1.3 g of alcohol protected compound in 84% yield. To a stirred solution of the above obtained compound (1.3 g, 3.85 mmol, 1.0 equiv) in MeOH/DCM 1:3 (39 mL) was added hydrazine monohydrate (2.9 g, 57.8 mmol, 15 equiv) and the reaction mixture was stirred at room temperature for 15 h. After the reaction was complete as monitored by thin-layer chromatography, the mixture was concentrated and the crude residue was

purified via silica gel chromatography (10% MeOH/CH_2Cl_2 + 1% NH_4OH) to give 320 mg of desired amine in 40% yield. ^{1}H NMR (500 MHz, $CDCl_3$) δ 7.23 (d, J = 8.2 Hz, 2H), 6.86 (d, J = 8.2 Hz, 2H), 4.45 (s, 2H), 3.79 (s, 3H), 2.80 (br, 2H), 0.89 (t, J = 6.0 Hz, 2H), 0.54 (t, J = 6.0 Hz, 2H). HRMS (ESI+, m/z) calcd for $C_{12}H_{18}NO_2$ $(M+H)^+$: 208.1332, found: 208.1338.

・**Synthesis of Imine**

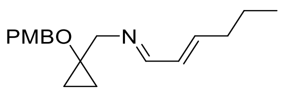

General procedure A was followed using above obtained amine (150 mg, 0.724 mmol, 1.0 equiv), *trans*-2-hexenal (71 mg, 0.724 mmol, 1.0 equiv), molecular sieves 4 Å, and DCM (1.5 mL). Imine (180 mg, 95% yield) was obtained as a pale-yellow oil. ^{1}H NMR (400 MHz, $CDCl_3$) δ 7.82 (d, J = 8.5 Hz, 1H), 7.18 (d, J = 8.5 Hz, 2H), 6.81 (d, J = 8.6 Hz, 2H), 6.30 – 6.09 (m, 2H), 4.50 (s, 2H), 3.77 (s, 3H), 3.67 (s, 2H), 2.17 (q, J = 7.3 Hz, 2H), 1.47 (h, J = 7.3 Hz, 2H), 0.96 – 0.88 (m, 5H), 0.68 – 0.63 (dd, J = 7.2, 5.2 Hz, 2H).

・**Key Reaction**

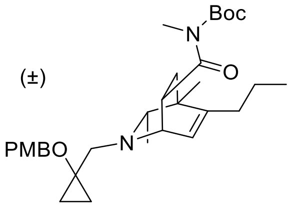

General procedure B was followed using above obtained imine (120 mg, 0.418 mmol, 1.0 equiv) and 2-butyne (164 µL, 2.09 mmol, 5 equiv) in THF (0.42 mL). Rh catalyst (10 mol %, 0.42 mL, 0.042 mmol, 100 mM in THF) was added, and the reaction was carried out at 65 °C for 12 h to produce the DHP intermediate. Following general procedure C, Boc-protected *N*-methyl acrylamide (773 mg, 4.18 mmol, 10 equiv) was added to the solution of DHP intermediate, and the reaction was carried out at 50 °C for 12 h. The resulting crude material was purified by column chromatography (30% *t*BuOMe/Pentane+1% Et_3N) to afford the desired product (80 mg, 36% yield from imine) as a pale-yellow oil. ^{1}H NMR (400 MHz, CD_3OD) δ 7.18 (d, J = 8.6 Hz, 2H), 6.81 (d, J = 8.6 Hz, 2H), 5.92 (dt, J = 6.5, 1.8 Hz, 1H), 4.55 (d, J = 10.6 Hz, 1H), 4.48 (d, J = 10.6 Hz, 1H), 4.28 – 4.21 (m, 1H), 3.79 – 3.71 (m, 4H), 3.08 (d, J = 14.2 Hz, 1H), 3.00 (s, 3H), 2.71 (d, J = 14.2 Hz, 1H), 2.17 – 1.90 (m, 3H), 1.59 – 1.40 (m, 13H), 1.06 (s, 3H), 0.93 (t, J = 7.3 Hz, 3H), 0.87 (d, J = 6.2 Hz, 3H), 0.85 – 0.81 (m, 2H), 0.63 – 0.60 (m, 2H).

・**Deprotection**

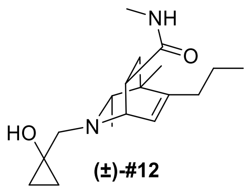

(±)-#12

Final Compound #12 General Procedure D was followed using protected isoquinuclidine (80 mg, 0.152 mmol, 1.00 equiv) in CH_2Cl_2 (1.5 mL) and was reacted with trifluoroacetic acid (113 µL, 1.52 mmol, 10.0 equiv) for 12 h. The crude product was purified by preparative thin-layer chromatography (10% MeOH/CH_2Cl_2 + 1% NH_4OH) to yield the racemic isoquinuclidine (28 mg, 60% yield) as a pale-yellow oil. ^{1}H NMR (400 MHz, CD_3OD) δ 5.95 (dt, *J* = 6.5, 1.9 Hz, 1H), 3.70 (dd, *J* = 6.5, 2.7 Hz, 1H), 3.00 (ddd, *J* = 9.5, 5.2, 2.7 Hz, 1H), 2.88 (d, *J* = 13.4 Hz, 1H), 2.65 (s, 3H), 2.58 (d, *J* = 13.4 Hz, 1H), 2.16 – 1.92 (m, 3H), 1.59 (dd, *J* = 12.7, 5.2 Hz, 1H), 1.55 – 1.38 (m, 3H), 1.09 (s, 3H), 0.95 (t, *J* = 7.3 Hz, 3H), 0.87 (d, *J* = 6.2 Hz, 3H), 0.71 – 0.69 (m, 2H), 0.60 – 0.51 (m, 2H). ^{13}C NMR (101 MHz, CD_3OD) δ 175.7, 146.5, 123.4, 64.9, 60.9, 55.6, 52.9, 40.3, 37.9, 36.0, 33.4, 25.1, 20.1, 18.6, 18.2, 12.9, 11.5, 11.3. HRMS (ESI+, m/z) calcd for $C_{18}H_{31}N_2O_2$ $(M+H)^+$: 307.2380, found: 307.2375.

Synthesis of #14

・**Synthesis of Imine**

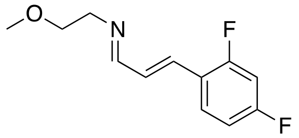

General procedure A was followed using 2-methoxyethan-1-amine (100 mg, 1.33 mmol, 1.00 equiv), (*E*)-3-(2,4-difluorophenyl)acrylaldehyde (224 mg, 1.33 mmol, 1.00 equiv), K_2CO_3 (64 mg, 0.466 mmol, 0.35 equiv), and THF (2.66 mL) for 4 h at rt. The product imine was obtained as pale-yellow oil (198 mg, 66%). ^{1}H NMR (400 MHz, $CDCl_3$) δ 8.06 (m, 1H), 7.51 (m, 1H), 7.06 (m, 1H), 6.96 – 6.79 (m, 3H), 3.71 (m, 2H), 3.69 – 3.64 (m, 2H), 3.38 (s, 3H).

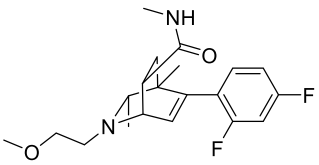

(±)-#14

Final Compound #14 Following General Procedure B for the formation of 1,2-dihydropyridines, the reaction was set up with above obtained imine (40.0 mg, 0.178 mmol, 1.00 equiv), Rh stock solution (178 µL, 17.8 µmol, 10 mol %), 2-butyne (139 µL, 1.78 mmol, 10.0 equiv), and a reaction time of 20 h at 70 ºC. Isoquinuclidine formation was conducted according to the General Procedure C with N-methylacrylamide (151 mg, 1.78 mmol, 10.0 equiv) for 16 h at 90 ºC. The product after the Diels-

Alder reaction was purified by silica gel chromatography (50% EtOAc/hexanes + 1% Et_3N) to afford the isoquinuclidine product as pale-yellow oil (13.3 mg, 21% yield over two steps). ^{1}H NMR (400 MHz, $CDCl_3$) δ 7.16 (td, J = 8.4, 6.5 Hz, 1H), 6.79 (m, 2H), 6.26 (d, J = 6.4 Hz, 1H), 5.67 (s, 1H), 3.80 (dd, J = 6.6, 2.7 Hz, 1H), 3.58 (m, 2H), 3.36 (s, 3H), 3.16 (m, 1H), 2.86 (m, 2H), 2.75 (d, J = 4.8 Hz, 3H), 2.08 (q, J = 6.4 Hz, 1H), 1.88 (dd, J = 13.1, 5.2 Hz, 1H), 1.52 (dd, J = 13.0, 9.5 Hz, 1H), 0.95 (d, J = 6.2 Hz, 3H), 0.92 (s, 3H). ^{13}C NMR (126 MHz, $CDCl_3$) δ 174.3, 163.2 (d, J = 11.8 Hz), 161.3 (d, J = 11.9 Hz), 160.7 (d, J = 12.4 Hz), 142.5, 132.1(dd, J = 9.3, 5.6 Hz), 131.6, 111.1 (dd, J = 20.9, 3.4 Hz), 103.79 – 103.28 (m), 72.5, 65.1, 58.9, 55.4, 53.1, 41.2, 38.7, 36.8, 26.5, 20.2 (d, J = 2.6 Hz), 19.3. ^{19}F NMR (376 MHz, $CDCl_3$) δ -107.9 (m), -111.6 (m). HRMS (ESI+, m/z) $[M+H]^+$ calcd: 365.2035, found: 365.2033

Synthesis of #17

・**Synthesis of Amine**

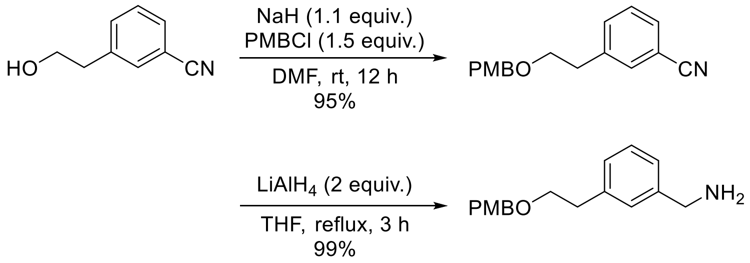

To a flame-dried round-bottom flask was added a magnetic stir bar, 3-(2-hydroxyethyl)benzonitrile (580 mg, 3.94 mmol, 1 equiv) and 4-methoxybenzyl chloride (926 mg, 5.91 mmol, 1.5 equiv) dissolved in DMF (8 mL). To this solution was then added sodium hydride (109 mg, 4.73 mmol, 1.2 equiv). The reaction mixture was stirred at room temperature for 12 h. After the reaction was complete as monitored by thin-layer chromatography, the mixture was then diluted with water, extracted with ethyl acetate, washed with brine, dried with Na_2SO_4 and filtered. The organic layer was concentrated and the crude residue was purified via silica gel chromatography (gradient of 0-100% EtOAc in hexane +1% Et_3N) to give 1001 mg of the protected alcohol in 95% yield. To a stirred solution of the above obtained compound in THF (37 mL) was added $LiAlH_4$ (284 mg, 7.48 mmol, 2 equiv), and the reaction mixture was heated under reflux for 12 h. After the reaction was complete as monitored by thin-layer chromatography, the mixture was then quenched by careful addition of 1N NaOH and then H_2O, extracted with ethyl acetate, washed with brine, dried with Na_2SO_4 and filtered. The organic layer was concentrated and the crude residue was purified via silica gel chromatography (5% MeOH/CH_2Cl_2 + 1% NH_4OH) to give 1005 mg of the amine in 99% yield. ^{1}H NMR (400 MHz, $CDCl_3$) δ 7.30 – 7.18 (m, 3H), 7.14 (d, J = 7.8 Hz, 2H), 7.09 (d, J = 7.1 Hz, 1H), 6.85 (d, J = 8.6 Hz, 2H), 4.44 (s, 2H), 3.82 (s, 2H), 3.79 (s, 3H), 3.65 (t, J = 7.2 Hz, 2H), 2.90 (t, J = 7.2 Hz, 2H). ^{13}C NMR (101 MHz, $CDCl_3$)

δ 159.1, 143.2, 139.3, 130.5, 129.2, 128.5, 127.7, 127.4, 124.9, 113.8, 72.6, 70.9, 55.3, 46.4, 36.3. HRMS (ESI+, m/z) calcd for $C_{17}H_{22}NO_2$ $(M+H)^+$: 272.1645, found: 272.1653.

・**Synthesis of Imine**

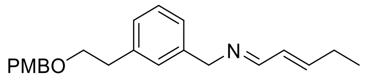

General procedure A was followed using above obtained amine (200 mg, 0.737 mmol, 1.0 equiv), *trans*-2-pentenal (0.36 mL, 3.69 mmol, 5.0 equiv), molecular sieves 4 Å, and THF (1.5 mL). Imine (225 mg, 91% yield) was obtained as a pale-yellow oil. ^{1}H NMR (400 MHz, $CDCl_3$) δ 8.02 – 7.89 (m, 1H), 7.23 – 7.18 (m, 3H), 7.16 – 7.02 (m, 3H), 6.85 (d, *J* = 8.6 Hz, 2H), 6.32 – 6.21 (m, 2H), 4.57 (s, 2H), 4.43 (s, 2H), 3.78 (s, 3H), 3.64 (t, *J* = 7.3 Hz, 2H), 2.89 (t, *J* = 7.3 Hz, 2H), 2.22 (dq, *J* = 11.4, 7.3 Hz, 2H), 1.05 (t, *J* = 7.4 Hz, 3H).

・**Key Reaction**

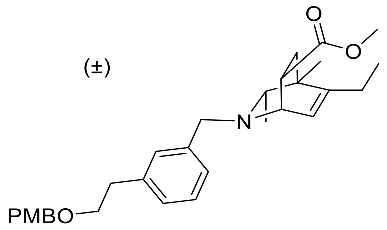

General procedure B was followed using above obtained imine (220 mg, 0.652 mmol, 1.0 equiv) and 2-butyne (256 μL, 3.26 mmol, 5 equiv) in THF (0.65 mL). Rh catalyst (10 mol %, 0.65 mL, 0.065 mmol, 100 mM in THF) was added, and the reaction was carried out at 65 °C for 12 h to produce the DHP intermediate. Following general procedure C, methyl acrylate (0.59 mL, 6.52 mmol, 10 equiv) was added to the solution of DHP intermediate, and the reaction was carried out at 50 °C for 12 h. The resulting crude material was purified by column chromatography (30% *t*BuOMe/Pentane+1% Et_3N) to afford the desired product (65 mg, 21% yield from imine) as a pale-yellow oil. ^{1}H NMR (400 MHz, CD_3OD) δ 7.29 – 7.06 (m, 6H), 6.83 (d, *J* = 8.7 Hz, 2H), 5.81 (dt, *J* = 6.5, 2.0 Hz, 1H), 4.41 (s, 2H), 3.75 (s, 3H), 3.74 – 3.56 (m, 3H), 3.56 (s, 3H), 3.36 – 3.29 (m, 1H), 2.86 (t, *J* = 6.9 Hz, 2H), 2.22 – 1.94 (m, 3H), 1.70 (dd, *J* = 12.9, 4.7 Hz, 1H), 1.52 (dd, *J* = 12.9, 9.4 Hz, 1H), 1.08 (s, 3H), 0.99 (t, *J* = 7.3 Hz, 3H), 0.66 (d, *J* = 6.2 Hz, 3H). HRMS (ESI+, m/z) calcd for $C_{30}H_{40}NO_4$ $(M+H)^+$: 478.2952, found: 478.2959.

・**Deprotection**

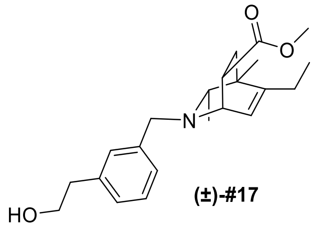

(±)-#17

Final Compound #17 General Procedure D was followed using protected isoquinuclidine (32 mg, 0.067 mmol, 1.00 equiv) in CH_2Cl_2 (1.0 mL) and was reacted with trifluoroacetic acid (50 µL, 0.67 mmol, 10.0 equiv) for 12 h. The crude product was purified by preparative thin-layer chromatography (10% MeOH/CH_2Cl_2 + 1% NH_4OH) to yield the racemic isoquinuclidine (22 mg, 92% yield) as a pale-yellow oil. ^{1}H NMR (400 MHz, CD_3OD) δ 7.30 – 7.17 (m, 3H), 7.12 (dt, J = 6.6, 2.1 Hz, 1H), 5.82 (dt, J = 6.5, 2.0 Hz, 1H), 3.80 – 3.68 (m, 3H), 3.63 – 3.51 (m, 2H), 3.57 (s, 3H), 3.35 (ddd, J = 9.1, 4.5, 2.9 Hz, 1H), 2.80 (t, J = 7.2 Hz, 2H), 2.23 – 1.93 (m, 3H), 1.71 (dd, J = 12.9, 4.7 Hz, 1H), 1.52 (dd, J = 12.9, 9.4 Hz, 1H), 1.09 (s, 3H), 0.99 (t, J = 7.3 Hz, 3H), 0.68 (d, J = 6.2 Hz, 3H). ^{13}C NMR (101 MHz, CD_3OD) δ 175.3, 149.0, 138.8, 138.5, 130.2, 127.9, 127.6, 127.3, 122.3, 64.1, 62.9, 57.3, 52.8, 50.8, 40.3, 38.8, 36.6, 35.4, 23.9, 18.4, 17.9, 10.4. HRMS (ESI+, m/z) calcd for $C_{22}H_{31}NO_3$ $(M+H)^+$: 358.2377, found: 358.2365.

Synthesis of #19

・Synthesis of Imine

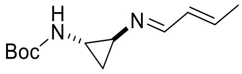

General procedure A was followed using *tert*-butyl *N*-[(1*S*)-2-aminocyclopropyl]carbamate (200 mg, 1.16 mmol, 1.0 equiv), *trans*-crotonaldehyde (0.47 mL, 5.8 mmol, 5.0 equiv), molecular sieves 4 Å, and THF (2 mL). Imine (260 mg, 99% yield) was obtained as a pale-yellow oil. ^{1}H NMR (400 MHz, $CDCl_3$) δ 8.00 – 7.93 (dd, J = 5.2, 2.9 Hz, 1H), 6.16 – 6.12 (m, 2H), 2.87 – 2.79 (m, 2H), 1.85 – 1.82 (m, 3H), 1.41 (s, 9H), 1.30 – 1.17 (m, 1H), 1.07 – 1.02 (m, 1H).

・Key reaction and Deprotection

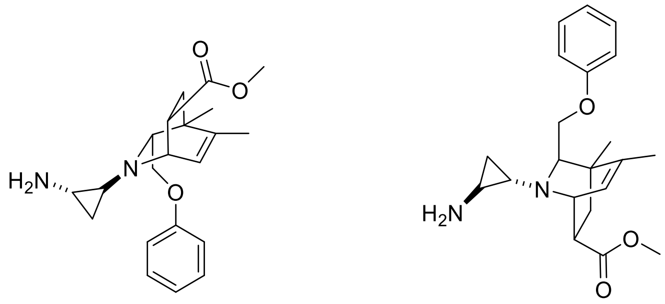

#19
Major and Minor diastereomers not assigned to specific structures

Final Compound #19 General procedure B was followed using above obtained imine (200 mg, 0.892 mmol, 1.0 equiv) and 2-butyne (349 µL, 4.46 mmol, 5 equiv) in THF (0.89 mL). Rh catalyst (10 mol %, 0.89 mL, 0.089 mmol, 100 mM in THF) was added, and the reaction was carried out at 75 °C for 12 h to produce the DHP intermediate. Following general procedure C, methyl acrylate (0.8 mL, 8.92 mmol, 10 equiv) was added to the solution of DHP intermediate, and the reaction was carried out at 50 °C for 12 h. The resulting crude material was purified by preparative thin-layer chromatography (30% *t*BuOMe/Pentane+1% Et_3N) to afford the mixture of diastereomers of protected product (53 mg, 13% yield from imine) as a pale-yellow oil. And General Procedure D was followed using protected isoquinuclidine (53 mg, 0.116 mmol, 1.00 equiv) in CH_2Cl_2 (1.2 mL) and was reacted with trifluoroacetic acid (86 µL, 1.16 mmol, 10.0 equiv) for 12 h. The crude product was purified by preparative thin-layer chromatography (10% MeOH/CH_2Cl_2 + 1% NH_4OH) to yield the diastereomers of isoquinuclidines (23 mg, 56% yield, 2:1 ratio) as a pale-yellow oil.

Major Diastereomer

^{1}H NMR (400 MHz, CD_3OD) δ 7.38 – 7.12 (m, 2H), 7.00 – 6.76 (m, 3H), 5.91 – 5.89 (m, 1H), 3.80 (dd, *J* = 10.0, 5.3 Hz, 1H), 3.67 (dd, *J* = 6.4, 3.2 Hz, 1H), 3.64 – 3.57 (m, 4H), 3.40 – 3.30 (m, 1H), 2.73 (t, *J* = 5.1 Hz, 1H), 2.50 – 2.41 (m, 1H), 1.95 – 1.86 (m, 1H), 1.80 – 1.71 (m, 4H), 1.65 (dd, *J* = 13.1, 9.4 Hz, 1H), 1.23 (s, 3H), 0.66 (ddd, *J* = 8.0, 5.6, 4.3 Hz, 1H), 0.58 (ddd, *J* = 7.7, 5.6, 4.3 Hz, 1H). ^{13}C NMR (101 MHz, CD_3OD) δ 175.0, 158.7, 144.3, 129.1, 124.8, 120.3, 113.9, 69.8, 68.5, 54.2, 50.9, 44.3, 39.3, 38.1, 34.9, 32.9, 18.9, 17.1, 12.9. HRMS (ESI+, m/z) calcd for $C_{21}H_{28}N_2O_3$ $(M+H)^+$: 357.2173, found: 357.2164.

Minor Diastereomer

^{1}H NMR (400 MHz, CD_3OD) δ 7.22 (dd, *J* = 8.7, 7.3 Hz, 2H), 6.89 – 6.84 (m, 1H), 6.82 (dd, *J* = 8.9, 1.1 Hz, 2H), 5.96– 5.94 (m, 1H), 3.82 (dd, *J* = 6.3, 3.2 Hz, 1H), 3.66 (dd, *J* = 10.1, 6.0 Hz, 1H), 3.63 (s, 3H), 3.55 (dd, *J* = 10.2, 4.2 Hz, 1H), 3.41 (dt, *J* = 9.2, 4.0 Hz ,1H), 2.63 (dd, *J* = 6.0, 4.2 Hz, 1H), 2.33 – 2.24 (m, 1H), 2.05 – 1.96 (m, 1H), 1.78 – 1.70 (m, 4H), 1.65 (dd, *J* = 13.1, 9.4 Hz, 1H), 1.21 (s, 3H), 0.94 (ddd, *J* = 8.0, 5.2, 4.1 Hz, 1H), 0.67 (dt, *J* = 7.4, 4.8 Hz, 1H). ^{13}C NMR (101 MHz,

CD_3OD) δ 175.1, 158.7, 144.2, 129.0, 125.0, 120.2, 113.9, 69.8, 67.9, 55.0, 50.9, 44.4, 39.4, 38.3, 34.9, 29.9, 18.9, 17.2, 16.9. HRMS (ESI+, m/z) calcd for $C_{21}H_{28}N_2O_3$ $(M+H)^+$: 357.2173, found: 357.2184.

Synthesis of #21

・**Synthesis of Imine**

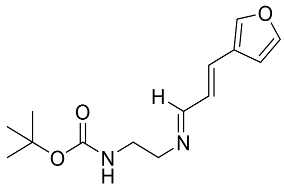

General procedure A was followed using *tert*-butyl *N*-(2-aminoethyl)carbamate (361 mg, 2.25 mmol, 1.0 equiv), (*E*)-3-(furan-3-yl)acrylaldehyde (275 mg, 2.25 mmol, 1.0 equiv), molecular sieves 4 Å, and THF (5 mL). Imine (590 mg, 99% yield) was obtained as a pale-yellow oil. ^1H NMR (400 MHz, $CDCl_3$) δ 7.96 (d, J = 9.0 Hz, 1H), 7.57 (s, 1H), 7.41 (t, J = 1.9 Hz, 1H), 6.87 (d, J = 15.9 Hz, 1H), 6.63 (dd, J = 15.9, 9.0 Hz, 1H), 6.59 (d, J = 1.9 Hz, 1H), 4.84 (br, 1H), 3.59 (t, J = 5.8 Hz, 2H), 3.41 (q, J = 5.8 Hz, 2H), 1.42 (s, 9H).

・**Key Reaction**

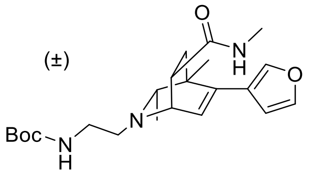

General procedure B was followed using above obtained imine (200 mg, 0.757 mmol, 1.0 equiv) and 2-butyne (296 μL, 3.78 mmol, 5 equiv) in THF (0.76 mL). Rh catalyst (10 mol %, 0.76 mL, 0.076 mmol, 100 mM in THF) was added, and the reaction was carried out at 65 °C for 12 h to produce the DHP intermediate. Following general procedure C, *N*-methyl acrylamide (644 mg, 7.57 mmol, 10 equiv) was added to the solution of DHP intermediate, and the reaction was carried out at 90 °C for 16 h. The resulting crude material was purified by column chromatography (5% MeOH/CH_2Cl_2 + 1% NH_4OH) to afford the desired product (27 mg, 9% yield from imine) as a pale-yellow oil. ^1H NMR (400 MHz, CD_3OD) δ 7.46 (s, 1H), 7.41 (t, J = 1.8 Hz, 1H), 6.43 (d, J = 1.8, Hz, 1H), 6.26 (d, J = 6.5 Hz, 1H), 3.62 (dd, J = 6.5, 2.8 Hz, 1H), 3.23 – 3.07 (m, 1H), 2.81 – 2.68 (m, 4H), 2.67 (s, 3H), 2.23 – 2.08 (m, 1H), 1.81 (dd, J = 12.8, 4.8 Hz, 1H), 1.56 – 1.48 (m, 1H), 1.42 (s, 9H), 1.17 (s, 3H), 0.87 (d, J = 6.2 Hz, 3H).

・**Deprotection**

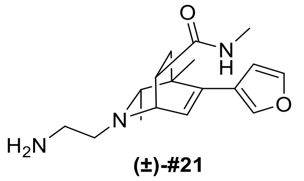

(±)-#21

Final Compound #21 General Procedure D was followed using protected isoquinuclidine (27 mg, 0.067 mmol, 1.00 equiv) in CH_2Cl_2 (1.0 mL) and was reacted with trifluoroacetic acid (50 µL, 0.67 mmol, 10.0 equiv) for 12 h. The crude product was purified by preparative thin-layer chromatography (10% MeOH/CH_2Cl_2 + 1% NH_4OH) to yield the racemic isoquinuclidine (17.5 mg, 86% yield) as a pale-yellow oil. ^{1}H NMR (500 MHz, CD_3OD) δ 7.47 (s, 1H), 7.42 (t, *J* = 1.8 Hz, 1H), 6.45 (d, *J* = 1.8 Hz, 1H), 6.28 (d, *J* = 6.5 Hz, 1H), 3.58 (dd, *J* = 6.5, 2.8 Hz, 1H), 3.14 (ddd, *J* = 9.5, 5.0, 2.8 Hz, 1H), 2.80 – 2.68 (m, 4H), 2.68 (s, 3H), 2.12 (q, *J* = 6.2 Hz, 1H), 1.81 (dd, *J* = 12.8, 5.0 Hz, 1H), 1.53 (dd, *J* = 12.8, 9.5 Hz, 1H), 1.19 (s, 3H), 0.88 (d, *J* = 6.2 Hz, 3H). ^{13}C NMR (126 MHz, CD_3OD) δ 175.4, 142.3, 139.1, 138.4, 127.4, 124.1, 110.1, 64.6, 56.0, 55.1, 40.7, 39.7, 38.0, 35.9, 25.2, 20.9, 18.9. HRMS (ESI+, m/z) calcd for $C_{17}H_{26}N_3O_2$ $(M+H)^+$: 304.2020, found: 304.2011.

Synthesis of #33

・Synthesis of imine

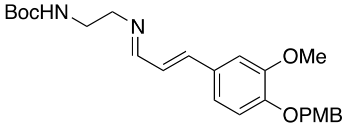

General Procedure A.1 was followed using *tert*-butyl (2-aminoethyl)carbamate (100 mg, 0.624 mmol 1.00 equiv), (*E*)-3-(3-methoxy-4-((4-methoxybenzyl)oxy)phenyl)acrylaldehyde (186 mg, 0.624 mmol, 1.00 equiv), K_2CO_3 (30.2 mg, 0.218 mmol, 0.35 equiv), and THF (1.25 mL) for 4 h at rt. The product imine was obtained as pale-yellow oil (245 mg, 89%). ^{1}H NMR (500 MHz, $CDCl_3$) δ 8.01 (d, *J* = 8.9 Hz, 1H), 7.36 (d, *J* = 8.6 Hz, 2H), 7.05 (s, 1H), 6.96 (d, *J* = 8.3 Hz, 1H), 6.92 – 6.88 (m, 4H), 6.79 (d, *J* = 8.7 Hz, 1H), 5.10 (s, 2H), 3.89 (s, 3H), 3.80 (s, 3H), 3.61 (t, *J* = 5.7 Hz, 2H), 3.42 (m, 2H), 1.44 (s, 9H).

・Key Reaction

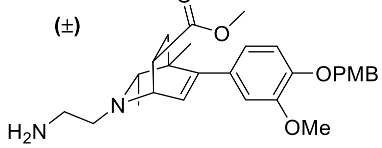

Following General Procedure B for the formation of 1,2-dihydropyridines, the reaction was set up with above obtained imine (350 mg, 0.794 mmol, 1.00 equiv), Rh stock solution (794 µL, 79.4 µmol,

10 mol %), 2-butyne (621 μL, 7.94 mmol, 10.0 equiv), and a reaction time of 16 h at 70 ºC. Isoquinuclidine formation was conducted according to the General Procedure C with methyl acrylate (720 μL, 7.94 mmol, 10.0 equiv) for 16 h at 90 ºC. The product after the Diels-Alder reaction was purified by silica gel chromatography (50% EtOAc/hexanes+1% Et_3N) to afford the protected isoquinuclidine as pale-yellow oil (254 mg, 55% yield over two steps). ^{1}H NMR (400 MHz, $CDCl_3$) δ 7.33 (d, *J* = 8.4 Hz, 2H), 6.86 (d, *J* = 8.4 Hz, 2H), 6.80 (d, *J* = 8.1 Hz, 1H), 6.67 (d, *J* = 1.9 Hz, 1H), 6.62 (dd, *J* = 8.2, 2.0 Hz, 1H), 6.10 (d, *J* = 6.3 Hz, 1H), 5.03 (s, 2H), 3.84 (s, 3H), 3.77 (s, 3H), 3.74 (m, 1H), 3.62 (s, 3H), 3.33 – 3.24 (m, 1H), 3.22 – 3.15 (m, 2H), 2.70 (m, 5.9 Hz, 2H), 2.13 (s, 3H), 2.08 (q, *J* = 6.5 Hz, 1H), 1.95 (dd, *J* = 13.1, 4.4 Hz, 1H), 1.55 – 1.49 (m, 1H), 1.43 (s, 9H), 1.00 (s, 3H), 0.91 (d, *J* = 6.2 Hz, 3H).

・**Deprotection**

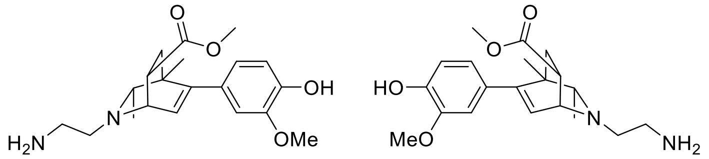

#33
enantiomers separated by chiral HPLC

Final Compound #33 General Procedure D was followed using protected isoquinuclidine (254 mg, 0.437 mmol, 1.00 equiv) in CH_2Cl_2 (3.64 mL) and was reacted with trifluoroacetic acid (335 μL, 4.37 mmol, 10.0 equiv) for 10 h. The crude product was purified by silica gel chromatography (10% MeOH/CH_2Cl_2 + 1% NH_4OH) to yield the racemic isoquinuclidine (48.0 mg, 31% yield) as pale-yellow oil. ^{1}H NMR (500 MHz, $CDCl_3$) δ 6.83 (d, *J* = 8.6 Hz, 1H), 6.65 (m, 2H), 6.12 (d, *J* = 6.3 Hz, 1H), 3.87 (s, 3H), 3.76 (dd, *J* = 6.4, 3.1 Hz, 1H), 3.64 (s, 3H), 3.25 – 3.19 (m, 1H), 3.02 (bs, 2H), 2.84 (m, 2H), 2.74 (dt, *J* = 12.2, 6.1 Hz, 1H), 2.65 (dt, *J* = 12.4, 6.3 Hz, 1H), 2.08 (q, *J* = 6.2 Hz, 1H), 1.97 (dd, *J* = 12.8, 4.2 Hz, 1H), 1.54 (dd, *J* = 13.0, 9.4 Hz, 1H), 1.02 (s, 3H), 0.94 (d, *J* = 6.2 Hz, 3H). ^{13}C NMR (126 MHz, $CDCl_3$) δ 174.8, 148.3, 146.1, 144.9, 132.2, 127.7, 121.3, 114.0, 111.2, 65.1, 57.3, 55.9, 54.5, 51.8, 41.2, 40.6, 38.1, 36.0, 21.7, 20.5. HRMS (ESI+, m/z) $[M+H]^+$ calcd: 361.2122, found: 361.2111

HPLC Traces

A portion of this material was separated utilizing semi-preparative chiral HPLC (Chiralpak AD-H column, 250 x 10 mm, 10% EtOH/Hexanes + 0.1% DEA, 2.5 mL/min) to provide pure enantiomers with t = 20 min and 25 min.

Chiralpak AD-H, analytical column (250 x 4.6 mm), 10% EtOH/Hexanes+0.1% DEA, 1 mL/min, 254 nm

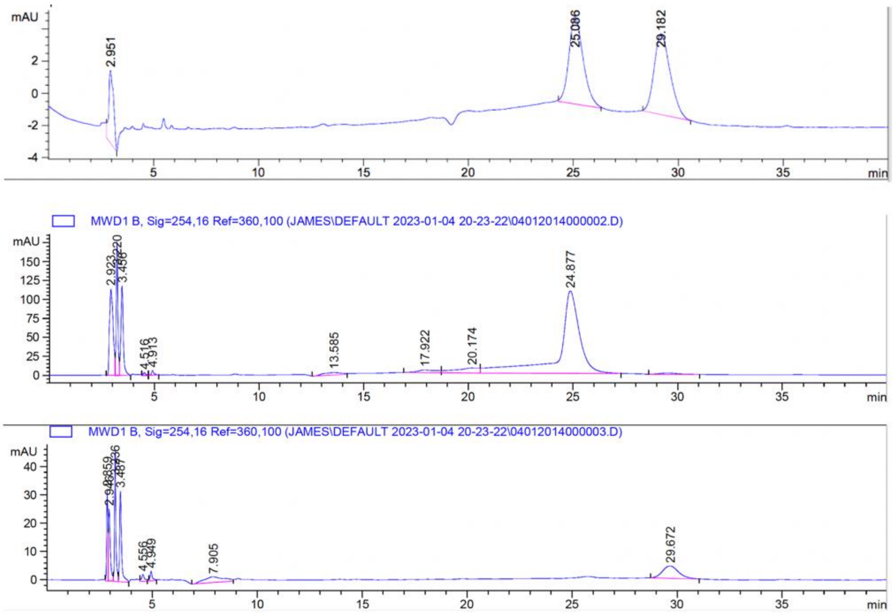

Synthesis of #39

・**Synthesis of Imine**

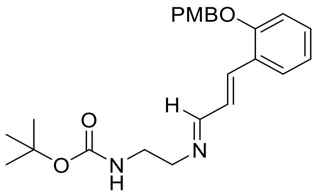

General procedure A was followed using *tert*-butyl *N*-(2-aminoethyl)carbamate (300 mg, 1.86 mmol, 1.0 equiv), (*E*)-3-[2-(4-Methoxy-benzyloxy)-phenyl]-propenal (500 mg, 1.86 mmol, 1.0 equiv), molecular sieves 4 Å, and THF (4 mL). Imine (760 mg, 99% yield) was obtained as a pale-yellow oil. ^{1}H NMR (400 MHz, $CDCl_3$) δ 8.01 (d, J = 9.0 Hz, 1H), 7.56 (d, J = 8.0 Hz, 1H), 7.42 (d, J = 16.2 Hz, 1H), 7.34 (d, J = 8.6 Hz, 2H), 7.27 (ddd, J = 8.6, 7.6, 1.6 Hz, 1H), 6.98 – 6.90 (m, 6H), 5.05 (s, 2H), 3.81 (s, 3H), 3.59 (t, J = 5.8 Hz, 2H), 3.41 (q, J = 5.8 Hz, 2H), 1.42 (s, 9H).

・**Key Reaction**

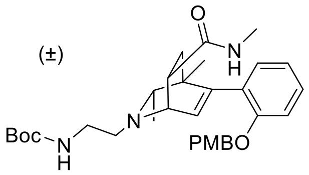

General procedure B was followed using above obtained imine (100 mg, 0.244 mmol, 1.0 equiv) and 2-butyne (95 µL, 1.22 mmol, 5 equiv) in THF (0.25 mL). Rh catalyst (10 mol %, 0.25 mL, 0.025 mmol, 100 mM in THF) was added, and the reaction was carried out at 65 °C for 12 h to produce the DHP

intermediate. Following general procedure C, *N*-methyl acrylamide (207 mg, 2.44 mmol, 10 equiv) was added to the solution of DHP intermediate, and the reaction was carried out at 90 °C for 16 h. The resulting crude material was purified by column chromatography (5% MeOH/CH_2Cl_2 + 1% NH_4OH) to afford the desired product (29 mg, 22% yield from imine) as a pale-yellow oil. ^{1}H NMR (400 MHz, CD_3OD) δ 7.37 (d, *J* = 8.6 Hz, 2H), 7.24 (ddd, *J* = 7.9, 7.3, 1.8 Hz, 1H), 7.08 (dd, *J* = 7.4, 1.8 Hz, 1H), 7.05 (d, *J* = 8.3 Hz, 1H), 6.98 – 6.84 (m, 3H), 6.10 (d, *J* = 6.4 Hz, 1H), 5.04 (d, *J* = 10.8 Hz, 1H), 4.98 (d, *J* = 10.8 Hz, 1H), 3.77 (s, 3H), 3.64 – 3.54 (m, 1H), 3.21 – 3.04 (m, 4H), 2.74 – 2.65 (m, 2H), 2.50 (s, 3H), 1.69 (dd, *J* = 13.0, 4.8 Hz, 1H), 1.49 (dd, *J* = 13.0, 9.8 Hz, 1H), 1.42 (s, 9H), 0.93 (s, 3H), 0.86 (d, *J* = 6.3 Hz, 3H).

・**Deprotection**

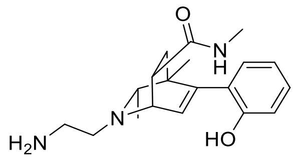

(±)-#39

Final Compound #39 General Procedure D was followed using protected isoquinuclidine (29 mg, 0.053 mmol, 1.00 equiv) in CH_2Cl_2 (1.0 mL) and was reacted with trifluoroacetic acid (39 µL, 0.53 mmol, 10.0 equiv) for 12 h. The crude product was purified by preparative thin-layer chromatography (10% MeOH/CH_2Cl_2 + 1% NH_4OH) to yield the racemic isoquinuclidine (12 mg, 69% yield) as a pale-yellow oil. ^{1}H NMR (400 MHz, CD_3OD) δ 7.10 (ddd, *J* = 8.5, 7.5, 1.7 Hz, 1H), 6.97 (dd, *J* = 7.5, 1.7 Hz, 1H), 6.87 – 6.73 (m, 2H), 6.14 (d, *J* = 6.4 Hz, 1H), 3.52 (dd, *J* = 6.4, 3.1 Hz, 1H), 3.26 (dt, *J* = 9.3, 3.1 Hz, 1H), 2.86 – 2.70 (m, 4H), 2.68 (s, 3H), 2.16 (q, *J* = 6.5 Hz, 1H), 1.82 (dd, *J* = 13.2, 4.0 Hz, 1H), 1.59 (dd, *J* = 13.2, 9.3 Hz, 1H), 1.00 (d, *J* = 6.2 Hz, 3H), 0.86 (s, 3H). ^{13}C NMR (101 MHz, CD_3OD) δ 176.5, 154.5, 143.7, 130.2, 128.9, 128.1, 126.1, 118.7, 115.3, 64.9, 56.3, 55.1, 48.4, 41.1, 39.6, 36.3, 25.2, 19.6, 19.5. HRMS (ESI+, m/z) calcd for $C_{19}H_{28}N_3O_2$ $(M+H)^+$: 330.2176, found: 330.2163.

Synthesis of #53

・**Synthesis of Amine**

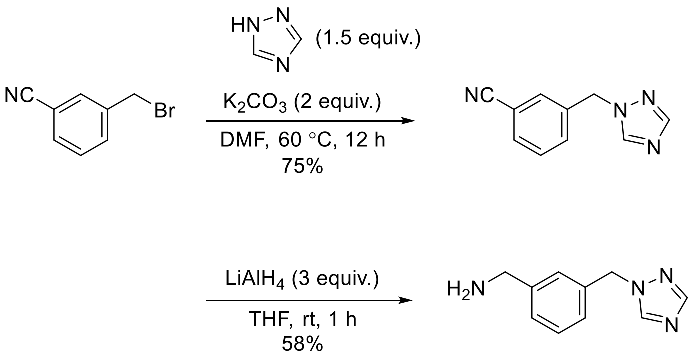

To a flame-dried round-bottom flask was added a magnetic stir bar, 3-(bromomethyl)benzonitrile (1.5 g, 7.65 mmol, 1 equiv) and 1H-1,2,4-triazole (793 mg, 11.5 mmol, 1.5 equiv) dissolved in DMF (15 mL). To this solution was then added potassium carbonate (2.1 g, 15.3 mmol, 2 equiv). The reaction mixture was stirred at 60 °C for 12 h. After the reaction was complete as monitored by thin-layer chromatography, the mixture was then diluted with water, extracted with ethyl acetate, washed with brine, dried with Na_2SO_4 and filtered. The organic layer was concentrated and the crude residue was purified via silica gel chromatography (gradient of 0-100% EtOAc in hexane +1% Et_3N) to give 1000 mg of the 3-((1H-1,2,4-triazol-1-yl)methyl)benzonitrile in 75% yield. To a stirred solution of the above obtained compound (500 mg, 2.71 mmol, 1 equiv) in THF (27 mL) was added $LiAlH_4$ (309 mg, 8.14 mmol, 3 equiv), and the reaction mixture was stirred at room temperature for 1 h. After the reaction was complete as monitored by thin-layer chromatography, the mixture was then quenched by careful addition of 1N NaOH and then H_2O, extracted with ethyl acetate, washed with brine, dried with Na_2SO_4 and filtered. The organic layer was concentrated and the crude residue was purified via silica gel chromatography (5% $MeOH/CH_2Cl_2$ + 1% NH_4OH) to give 295 mg of the amine in 58% yield. ^1H NMR (400 MHz, $_3$) δ 8.04 (s, 1H), 7.95 (s, 1H), 7.36 – 7.26 (m, 2H), 7.22 (s, 1H), 7.13 (d, J = 7.2 Hz, 1H), 5.32 (s, 2H), 3.86 (s, 2H).

・**Synthesis of Imine**

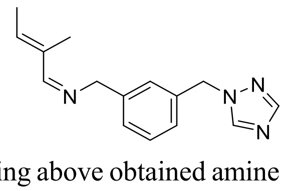

General procedure A was followed using above obtained amine (295 mg, 1.57 mmol, 1.0 equiv), *trans*-2-methyl-2-butenal (264 mg, 3.13 mmol, 2.0 equiv), molecular sieves 4 Å, and THF (3 mL). Imine (350 mg, 88% yield) was obtained as a pale-yellow oil. ^1H NMR (400 MHz, $CDCl_3$) δ 8.02 (s, 1H), 7.94 (s, 1H), 7.89 (s, 1H), 7.31 (dd, J = 8.0, 7.6 Hz, 1H), 7.25 (d, 8.0 Hz, 1H), 7.17 (s, 1H), 7.11 (d, J = 7.6 Hz, 1H), 5.99 (q, J = 6.3 Hz, 1H), 5.31 (s, 2H), 4.63 (s, 2H), 1.85 (s, 3H), 1.84 (d, J = 6.3 Hz, 3 H).

・**Key Reaction**

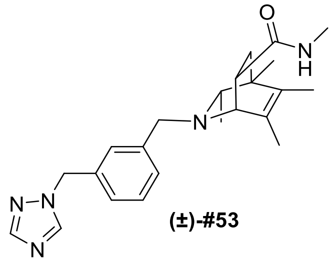

(±)-#53

Final Compound #53 General procedure B was followed using above obtained imine (170 mg, 0.668 mmol, 1.0 equiv) and 2-butyne (262 µL, 3.34 mmol, 5 equiv) in THF (0.67 mL). Rh catalyst (10 mol %, 0.67 mL, 0.067 mmol, 100 mM in THF) was added, and the reaction was carried out at 65 °C for 12 h to produce the DHP intermediate. Following general procedure C, *N*-methyl acrylamide (569 mg, 6.68 mmol, 10 equiv) was added to the solution of DHP intermediate, and the reaction was carried out at 90 °C for 16 h. The resulting crude material was purified by column chromatography (10% MeOH/CH_2Cl_2 + 1% NH_4OH) to afford the desired product (24 mg, 9% yield from imine) as a pale-yellow oil. ^{1}H NMR (400 MHz, CD_3OD) δ 8.52 (s, 1H), 7.97 (s, 1H), 7.37 – 7.34 (m, 2H), 7.30 (dd, *J* = 8.0, 7.7 Hz, 1H), 7.20 (d, *J* = 7.7 Hz, 1H), 5.40 (s, 2H), 3.70 (s, 2H), 3.15 – 3.06 (m, 2H), 2.63 (s, 3H), 2.13 (q, *J* = 6.2 Hz, 1H), 1.65 (s, 3H), 1.64 – 1.56 (m, 4H), 1.43 (dd, *J* = 12.7, 9.4 Hz, 1H), 1.04 (s, 3H), 0.56 (d, *J* = 6.2 Hz, 3H). ^{13}C NMR (101 MHz, CD_3OD) δ 175.5, 150.9, 143.6, 140.7, 135.3, 132.2, 132.1, 129.0, 128.6, 128.4, 126.5, 64.2, 60.5, 56.9, 52.8, 40.1, 37.8, 36.2, 25.1, 19.4, 18.1, 15.7, 12.4. HRMS (ESI+, m/z) calcd for $C_{23}H_{31}N_5O$ $(M+H)^+$: 394.2601, found: 394.2582.

Synthesis of #56

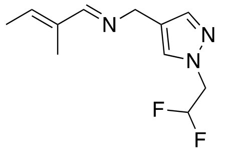

A modified version of General Procedure A was followed using the HCl salt of (1-(2,2-difluoroethyl)-1*H*-pyrazol-4-yl)methanamine (100 mg, 0.506 mmol, 1.00 equiv), (*E*)-2-methylbut-2-enal (244 µL, 2.53 mmol, 5.00 equiv), 3 Å MS (253 mg), and EtOH (1.01 mL) for 7 h at rt. The product imine was obtained as pale-yellow oil (115 mg, 100%). ^{1}H NMR (500 MHz, $CDCl_3$) δ 7.87 (s, 1H), 7.45 (s, 1H), 7.34 (s, 1H), 6.17 – 5.92 (m, 2H), 4.53 (s, 2H), 4.40 (td, *J* = 13.6, 4.3 Hz, 2H), 1.84 (m, 6H).

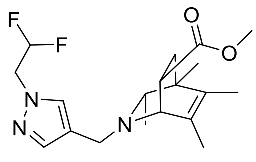

(±)-#56

Final Compound #56 Following General Procedure B for the formation of 1,2-dihydropyridines, the reaction was set up with above obtained imine (120 mg, 0.528 mmol, 1.0 equiv), Rh stock solution (528 µL, 52.8 µmol, 10 mol%), 2-butyne (413 µL, 5.28 mmol, 10.0 equiv), and a reaction time of 20 h at 70 ºC. Isoquinuclidine formation was conducted according to the General Procedure C with methyl acrylate (478 µL, 5.28 mmol, 10.0 equiv) for 16 h at 70 ºC. The product after the Diels-Alder reaction was purified by silica gel chromatography (10% MeOH/CH_2Cl_2+1% NH_4OH) to afford the isoquinuclidine product as pale-yellow oil (15.5 mg, 8% yield over two steps). ^{1}H NMR (500 MHz, $CDCl_3$) δ 7.54 (s, 1H), 7.44 (s, 1H), 6.06 (tt, *J* = 55.5, 4.4 Hz, 1H), 4.43 (td, *J* = 13.6, 4.4 Hz, 2H), 3.61 (m, 4H), 3.47 (d, *J* = 13.4 Hz, 1H), 3.39 (s, 1H), 3.13 (m, 1H), 2.02 (m, 1H), 1.77 – 1.73 (m, 1H), 1.64 (m, 6H), 1.44 – 1.36 (m, 1H), 1.06 (s, 3H), 0.76 (d, *J* = 6.2 Hz, 3H). ^{13}C NMR (126 MHz, $CDCl_3$) δ 159.7, 148.0, 141.3, 130.4, 130.4, 113.4 (t, *J* = 243.6 Hz), 64.8, 58.2, 54.0 (t, *J* = 28.1 Hz), 51.6, 46.7, 40.3, 36.3, 20.4, 19.0, 16.6, 13.7. ^{19}F NMR (471 MHz, $CDCl_3$) δ -122.28 (m), -122.40 (m). HRMS (ESI+, m/z) $[M+H]^+$ calcd: 368.2144, found: 368.2135

Synthesis of #57

・**Synthesis of Amine**

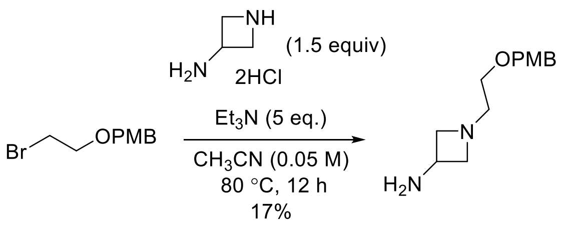

To a flame-dried round-bottom flask was added a magnetic stir bar, 1-[(2-bromoethoxy)methyl]-4-methoxy-benzene[2] (750 mg, 3.06 mmol, 1 equiv) and azetidin-3-amine dihydrochloride (666 mg, 4.59 mmol, 1.5 equiv) dissolved in CH_3CN (60 mL). To this solution was then added triethylamine (2.13 mL, 15.3 mmol, 5 equiv). The reaction mixture was stirred at 80 °C for 12 h. After the reaction was complete as monitored by thin-layer chromatography, the mixture was concentrated and the crude residue was purified via silica gel chromatography (5% MeOH/CH_2Cl_2 + 1% NH_4OH) to give 120 mg of the desired amine in 17% yield. ^{1}H NMR (400 MHz, CD_3OD) δ 7.23 (d, *J* = 8.5 Hz, 2H), 6.86 (d, *J* = 8.5 Hz, 2H), 4.39 (s, 2H), 3.76 (s, 3H), 3.62 (t, *J* = 6.8 Hz, 2H), 3.51 (p, *J* = 6.9 Hz, 1H), 3.44 (t, *J* = 5.5 Hz, 2H), 2.82 (t, *J* = 6.8 Hz, 2H), 2.65 (t, *J* = 5.5 Hz, 2H).

・**Synthesis of Imine**

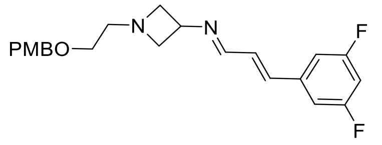

General procedure A was followed using above obtained amine (80 mg, 0.339 mmol, 1.0 equiv), (*E*)-3-(3,5-difluorophenyl)acrylaldehyde (56.9 mg, 0.339 mmol, 1.0 equiv), molecular sieves 4 Å, and THF (1 mL). Imine (130 mg, 99% yield) was obtained as a pale-yellow oil. ^{1}H NMR (400 MHz, CD_3OD) δ 8.01 (dd, *J* = 8.9, 1.0 Hz, 1H), 7.24 (d, *J* = 8.6 Hz, 2H), 7.21 – 7.15 (m, 2H), 7.11 (d, *J* = 16.0 Hz, 1H), 6.97 – 6.88 (m, 2H), 6.86 (d, *J* = 8.7 Hz, 2H), 4.41 (s, 2H), 4.23 (p, *J* = 6.9 Hz, 1H), 3.76 (s, 3H), 3.73 (td, *J* = 6.8, 1.9 Hz, 2H), 3.49 (t, *J* = 5.4 Hz, 2H), 3.22 (td, *J* = 6.9, 1.9 Hz, 2H), 2.73 (t, *J* = 5.4 Hz, 2H).

・**Key Reaction**

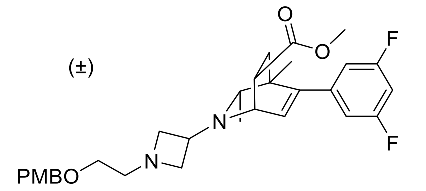

General procedure B was followed using above obtained imine (130 mg, 0.336 mmol, 1.0 equiv) and 2-butyne (132 µL, 1.68 mmol, 5 equiv) in THF (0.34 mL). Rh catalyst (10 mol %, 0.34 mL, 0.034 mmol, 100 mM in THF) was added, and the reaction was carried out at 65 °C for 12 h to produce the DHP intermediate. Following general procedure C, methyl acrylate (0.3 mL, 3.36 mmol, 10 equiv) was added to the solution of DHP intermediate, and the reaction was carried out at 50 °C for 12 h. The resulting crude material was purified by column chromatography (30% *t*BuOMe/Pentane+1% Et_3N) to afford the desired product (140 mg, 79% yield from imine) as a pale-yellow oil. ^{1}H NMR (400 MHz, CD_3OD) δ 7.25 (d, *J* = 8.6 Hz, 2H), 6.90 – 6.81 (m, 3H), 6.72 – 6.69 (m, 2H), 6.17 (d, *J* = 6.4 Hz, 1H), 4.42 (s, 2H), 3.76 (s, 3H), 3.67 (dd, *J* = 6.4, 3.2 Hz, 1H), 3.63 (s, 3H), 3.58 (t, *J* = 6.9 Hz, 2H), 3.53 – 3.43 (m, 3H), 3.16 (dt, *J* = 9.3, 3.8 Hz, 1H), 3.07 (t, *J* = 7.3 Hz, 1H), 3.00 (t, *J* = 7.7 Hz, 1H), 2.71 (t, *J* = 5.4 Hz, 2H), 2.23 (q, *J* = 6.3 Hz, 1H), 1.91 (dd, *J* = 13.2, 4.1 Hz, 1H), 1.60 (dd, *J* = 13.2, 9.4 Hz, 1H), 1.00 (s, 3H), 0.91 (d, *J* = 6.3 Hz, 3H). HRMS (ESI+, m/z) calcd for $C_{30}H_{37}F_2N_2O_4$ $(M+H)^+$: 527.2716, found: 527.2725.

・**Deprotection**

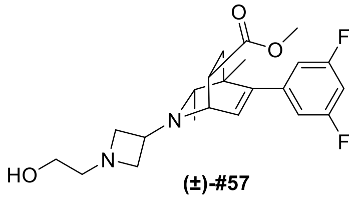

(±)-#57

Final Compound #57 General Procedure D was followed using protected isoquinuclidine (90 mg, 0.171 mmol, 1.00 equiv) in CH_2Cl_2 (1.0 mL) and was reacted with trifluoroacetic acid (127 µL, 1.71

mmol, 10.0 equiv) for 12 h. The crude product was purified by preparative thin-layer chromatography (10% MeOH/CH_2Cl_2 + 1% NH_4OH) to yield the racemic isoquinuclidine (69 mg, 99% yield) as a pale-yellow oil. ^{1}H NMR (400 MHz, cd$_3$od) δ 6.85 (tt, *J* = 9.3, 2.4 Hz, 1H), 6.71 (dd, *J* = 8.3, 2.1 Hz, 2H), 6.18 (d, *J* = 6.4 Hz, 1H), 3.69 (dd, *J* = 6.4, 3.2 Hz, 1H), 3.63 (s, 3H), 3.63 – 3.59 (m, 2H), 3.56 (t, *J* = 5.8 Hz, 2H), 3.49 (p, *J* = 7.1 Hz, 1H), 3.18 (dt, *J* = 9.3, 3.8 Hz, 1H), 3.07 (t, *J* = 7.3 Hz, 1H), 3.00 (t, *J* = 7.7 Hz, 1H), 2.64 (t, *J* = 5.8 Hz, 2H), 2.24 (q, *J* = 6.3 Hz, 1H), 1.91 (dd, *J* = 13.2, 4.1 Hz, 1H), 1.61 (dd, *J* = 13.2, 9.4 Hz, 1H), 1.01 (s, 3H), 0.92 (d, *J* = 6.3 Hz, 3H). ^{13}C NMR (101 MHz, CD_3OD) δ 174.4, 162.6 (dd, *J* = 248.5, 13.1 Hz), 146.9, 143.3 (t, *J* = 9.1 Hz), 129.2, 110.8 (dd, *J* = 19.2, 7.1 Hz), 101.9 (t, *J* = 26.3 Hz), 62.8, 60.9, 60.7, 59.7, 59.5, 53.1, 51.8, 51.1, 40.8, 38.3, 35.1, 20.0, 19.5. ^{19}F NMR (376 MHz, CD_3OD) δ -112.1. HRMS (ESI+, m/z) calcd for $C_{22}H_{28}F_2N_2O_3$ $(M+H)^+$: 407.2141, found: 407.2124.

Synthesis of #58

・Synthesis of Imine

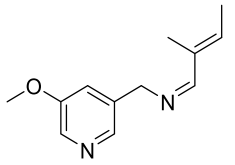

General procedure A was followed using (5-methoxy-3-pyridyl)methanamine (300 mg, 2.17 mmol, 1.0 equiv), *trans*-2-methyl-2-butenal (365 mg, 4.34 mmol, 2.0 equiv), molecular sieves 4 Å, and THF (5 mL). Imine (380 mg, 86% yield) was obtained as a pale-yellow oil. ^{1}H NMR (400 MHz, $CDCl_3$) δ 8.17 (d, *J* = 2.8 Hz, 1H), 8.13 (s, 1H), 7.92 (s, 1H), 7.13 (s, 1H), 6.01 (q, *J* = 6.6 Hz, 1H), 4.63 (s, 2H), 3.83 (s, 3H), 1.85 (s, 3H), 1.84 (d, *J* = 7.7 Hz, 3H).

・Key Reaction

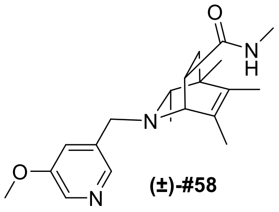

(±)-#58

Final Compound #58 General procedure B was followed using above obtained imine (180 mg, 0.881 mmol, 1.0 equiv) and 2-butyne (345 μL, 4.41 mmol, 5 equiv) in THF (0.88 mL). Rh catalyst (10 mol %, 0.88 mL, 0.088 mmol, 100 mM in THF) was added, and the reaction was carried out at 65 °C for 12 h to produce the DHP intermediate. Following general procedure C, *N*-methyl acrylamide (750 mg, 8.81 mmol, 10 equiv) was added to the solution of DHP intermediate, and the reaction was carried out

at 90 °C for 16 h. The resulting crude material was purified by column chromatography (10% MeOH/CH_2Cl_2 + 1% NH_4OH) to afford the desired product (70 mg, 23% yield from imine) as a white solid. ^{1}H NMR (500 MHz, CD_3OD) δ 8.14 (s, 1H), 8.11 (s, 1H), 7.47 (s, 1H), 3.87 (s, 3H), 3.79 (d, *J* = 14.0 Hz, 1H), 3.75 (d, J = 14.0 Hz 1H), 3.18 – 3.07 (m, 2H), 2.65 (s, 3H), 2.18 (q, *J* = 6.2 Hz, 1H), 1.68 (s, 3H), 1.68 – 1.62 (m, 4H), 1.48 (dd, *J* = 12.7, 9.2 Hz, 1H), 1.08 (s, 3H), 0.66 (d, *J* = 6.2 Hz, 3H). ^{13}C NMR (126 MHz, CD_3OD) δ 175.4, 156.3, 141.3, 137.4, 135.3, 132.3, 131.9, 121.6, 64.3, 60.5, 54.8, 53.9, 40.2, 37.9, 36.3, 25.1, 19.4, 18.2, 15.7, 12.4. HRMS (ESI+, m/z) calcd for $C_{20}H_{29}N_3O_2$ $(M+H)^+$: 344.2333, found: 344.2316.

Synthesis of #61

・**Synthesis of Imine**

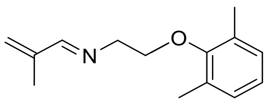

General procedure A was followed using [2-(2,6-dimethylphenoxy)ethyl]amine (250 mg, 1.39 mmol, 1.0 equiv), 2-methylprop-2-enal (486 mg, 6.93 mmol, 5.0 equiv), molecular sieves 4 Å, and THF (3 mL). Imine (310 mg, 96% yield) was obtained as a pale-yellow oil. ^{1}H NMR (400 MHz, $cdcl_3$) δ 8.03 (s, 1H), 6.97 (d, *J* = 7.4 Hz, 2H), 6.89 (dd, *J* = 8.3, 6.6 Hz, 1H), 5.61 (s, 1H), 5.40 (s, 1H), 4.00 (t, *J* = 5.4 Hz, 2H), 3.87 (t, *J* = 5.4 Hz, 2H), 2.24 (s, 6H), 1.95 (s, 3H).

・**Key Reaction**

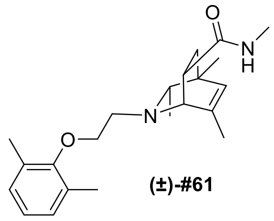

(±)-#61

Final Compound #61 General procedure B was followed using above obtained imine (200 mg, 0.920 mmol, 1.0 equiv) and 2-butyne (361 µL, 4.60 mmol, 5 equiv) in THF (0.92 mL). Rh catalyst (10 mol %, 0.92 mL, 0.092 mmol, 100 mM in THF) was added, and the reaction was carried out at 65 °C for 12 h to produce the DHP intermediate. Following general procedure C, *N*-methyl acrylamide (783 mg, 9.20 mmol, 10 equiv) was added to the solution of DHP intermediate, and the reaction was carried out at 90 °C for 16 h. The resulting crude material was purified by column chromatography (10% MeOH/CH_2Cl_2 + 1% NH_4OH) to afford the desired product (25 mg, 8% yield from imine) as a pale-yellow oil. ^{1}H NMR (400 MHz, CD_3OD) δ 6.97 (d, *J* = 7.4 Hz, 2H), 6.87 (dd, *J* = 8.2, 6.8 Hz, 1H), 5.51 (s, 1H), 3.93 (dt, *J* = 9.6, 6.3 Hz, 1H), 3.85 (dt, *J* = 9.6, 6.2 Hz, 1H), 3.44 (dd, *J* = 2.9, 1.4 Hz,

1H), 3.16 (ddd, *J* = 9.3, 4.9, 2.9 Hz, 1H), 3.09 (dt, *J* = 12.8, 6.3 Hz, 1H), 2.98 (dt, *J* = 12.9, 6.2 Hz, 1H), 2.65 (s, 3H), 2.27 (s, 6H), 2.24 (d, *J* = 2.7 Hz, 1H), 2.09 (q, *J* = 6.4 Hz, 1H), 1.76 (d, *J* = 1.7 Hz, 3H), 1.61 (dd, *J* = 12.6, 4.9 Hz, 1H), 1.44 (dd, *J* = 12.6, 9.4 Hz, 1H), 1.07 (s, 3H), 0.84 (d, *J* = 6.2 Hz, 3H). ^{13}C NMR (101 MHz, CD_3OD) δ 175.2, 155.9, 140.4, 130.4, 128.9, 128.5, 123.6, 71.2, 64.8, 60.6, 53.9, 38.2, 37.7, 35.9, 25.1, 21.1, 19.1, 18.7, 15.3. HRMS (ESI+, m/z) calcd for $C_{22}H_{33}N_2O_2$ $(M+H)^+$: 357.2537, found: 357.2541.

Synthesis of #64

・**Synthesis of Imine**

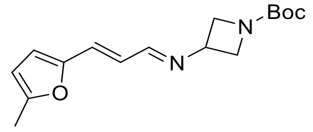

General procedure A was followed using *tert*-butyl-3-aminoazetidine-1-carboxylate (190 mg, 1.10 mmol, 1.0 equiv), (*E*)-3-(5-methyl-2-furyl)-2-propenal (150 mg, 1.10 mmol, 1.0 equiv), molecular sieves 4 Å, and THF (2 mL). Imine (320 mg, 99% yield) was obtained as a pale-yellow oil. ^{1}H NMR (500 MHz, $CDCl_3$) δ 7.86 (d, *J* = 7.7 Hz, 1H), 6.80 – 6.57 (m, 2H), 6.40 (d, *J* = 3.2 Hz, 1H), 6.04 (d, *J* = 3.2 Hz, 1H), 4.28 – 4.10 (m, 3H), 3.98 (d, *J* = 4.8 Hz, 2H), 2.33 (s, 3H), 1.44 (s, 9H).

・**Key Reaction**

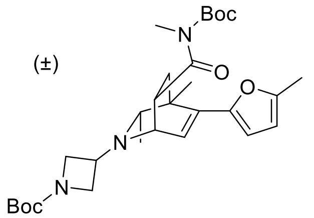

General procedure B was followed using above obtained imine (300 mg, 1.03 mmol, 1.0 equiv) and 2-butyne (405 µL, 5.17 mmol, 5 equiv) in THF (1.0 mL). Rh catalyst (20 mol %, 2.06 mL, 0.0206 mmol, 100 mM in THF) was added, and the reaction was carried out at 75 °C for 12 h to produce the DHP intermediate. Following general procedure C, Boc-protected *N*-methyl acrylamide (956 mg, 5.17 mmol, 5 equiv) was added to the solution of DHP intermediate, and the reaction was carried out at 50 °C for 12 h. The resulting crude material was purified by column chromatography (30% *t*BuOMe/Pentane+1% Et_3N) to afford the desired product (125 mg, 23% yield from imine) as a pale-yellow oil. ^{1}H NMR (400 MHz, CD_3OD) δ 6.34 (d, *J* = 6.6 Hz, 1H), 6.26 (d, *J* = 3.2 Hz, 1H), 5.95 (dd, *J* = 3.2, 1.3 Hz, 1H), 4.33 (dt, *J* = 9.3, 4.3 Hz, 1H), 4.16 (t, *J* = 7.2 Hz, 1H), 4.01 (t, *J* = 7.6 Hz, 1H), 3.93 (t, *J* = 5.6 Hz, 1H), 3.82 – 3.75 (m, 2H), 3.71 (dd, *J* = 6.8, 2.8 Hz, 1H), 3.04 (s, 3H), 2.27 – 2.22 (m, 4H), 2.01 (dd, *J* = 16.6, 4.3 Hz, 1H), 1.55 (s, 9H), 1.52 – 1.47 (m, 1H), 1.42 (s, 9H), 1.35 (s, 3H),

0.92 (d, J = 6.2 Hz, 3H).

・**Deprotection**

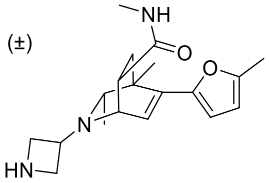

General Procedure D was followed using protected isoquinuclidine (80 mg, 0.151 mmol, 1.00 equiv) in CH_2Cl_2 (1.5 mL) and was reacted with trifluoroacetic acid (224 µL, 3.02 mmol, 20.0 equiv) for 12 h. The crude product was purified by preparative thin-layer chromatography (10% MeOH/CH_2Cl_2 + 1% NH_4OH) to yield the deprotected isoquinuclidine (33 mg, 51% yield) as a pale-yellow oil. ^1H NMR (400 MHz, CD_3OD) δ 6.47 (d, J = 6.6 Hz, 1H), 6.27 (d, J = 3.3 Hz, 1H), 5.95 (dd, J = 3.3, 1.1 Hz, 1H), 3.90 – 3.48 (m, 6H), 2.93 (dt, J = 9.4, 4.4 Hz, 1H), 2.64 (s, 3H), 2.29 – 2.17 (m, 4H), 1.80 (dd, J = 13.1, 4.4 Hz, 1H), 1.52 (dd, J = 13.1, 9.4 Hz, 1H), 1.34 (s, 3H), 0.85 (d, J = 6.2 Hz, 3H).

・**Addition to 3-chloro pyridazine**

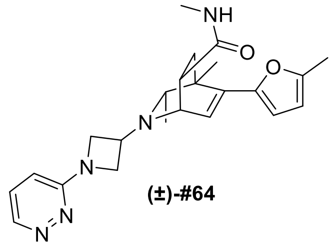

(±)-#64

Final Compound #64 To a flame-dried round-bottom flask was added a magnetic stir bar, above obtained isoquinuclidine (33 mg, 0.1 mmol, 1.0 equiv) and 3-chloropyridazine (57 mg, 0.5 mmol, 5.0 equiv) dissolved in EtOH (1 mL). To this solution was then added *N*,*N*-diisopropylethylamine (174 µL, 1.0 mmol, 10 equiv). The reaction mixture was stirred at 90 °C for 12 h. After the reaction was complete as monitored by thin-layer chromatography, the mixture was concentrated and the crude residue was purified by preparative thin-layer chromatography (10% MeOH/CH_2Cl_2 + 1% NH_4OH) to give the racemic isoquinuclidine (21 mg, 51% yield) as a pale-yellow oil. ^1H NMR (400 MHz, CD_3OD) δ 8.46 (dd, J = 4.5, 1.3 Hz, 1H), 7.39 (dd, J = 9.1, 4.5 Hz, 1H), 6.87 (dd, J = 9.1, 1.4 Hz, 1H), 6.49 (d, J = 6.6 Hz, 1H), 6.28 (d, J = 3.3 Hz, 1H), 5.96 (dd, J = 3.3, 1.1 Hz, 1H), 4.33 – 4.20 (m, 2H), 4.10 – 3.94 (m, 3H), 3.73 (dd, J = 6.6, 3.0 Hz, 1H), 3.03 (dt, J = 9.6, 4.0 Hz, 1H), 2.67 (s, 3H), 2.36 (q, J = 6.3 Hz, 1H), 2.24 (s, 3H), 1.87 (dd, J = 13.2, 4.4 Hz, 1H), 1.57 (dd, J = 13.1, 9.6 Hz, 1H), 1.37 (s, 3H), 0.94 (d, J = 6.3 Hz, 3H). ^{13}C NMR (101 MHz, CD_3OD) δ 174.7, 160.9, 151.3, 150.5, 142.6, 136.7, 128.1, 124.6, 113.0, 108.5, 106.7, 62.3, 55.6, 55.5, 52.6, 52.4, 40.4, 39.1, 35.3, 25.2, 20.9, 19.3, 11.9. HRMS (ESI+, m/z) calcd for $C_{23}H_{29}N_5O_2$ $(M+H)^+$: 408.2394, found: 408.2375.

5. Analogs Based upon Screening Hits (#001-#036): Synthesis Procedures and Analytical Data

Synthesis of #001

・**Synthesis of 0.8 M imine solution**

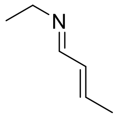

To a flame-dried vial was added a magnetic stir bar, *trans*-crotonaldehyde (0.6 mL, 7.3 mmol, 1.0 equiv) in dry THF (1.2 mL), and molecular sieves 4 Å. To this solution was then slowly added 2.0 M ethylamine in THF (7.3 mL, 14.6 mmol, 2.0 equiv) at 0 °C and the reaction solution was stirred at 0 °C for 2 h. Upon reaction completion, the 0.8 M imine solution was stored at –20 °C in a nitrogen filled glovebox and taken on to the next step without further purification.

・**Key Reaction**

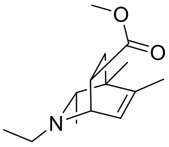

(±)-#001

Final Compound #001 General procedure B was followed using above obtained 0.8 M solution of imine (2 mL, 1.6 mmol, 1.0 equiv) and 2-butyne (625 µL, 8.0 mmol, 5 equiv) in THF (1.6 mL). Rh catalyst (10 mol %, 1.6 mL, 0.16 mmol, 100 mM in THF) was added, and the reaction was carried out at 65 °C for 12 h to produce the DHP intermediate. Following general procedure C, methyl acrylate (1.44 mL, 16.0 mmol, 10 equiv) was added to the solution of DHP intermediate, and the reaction was carried out at 50 °C for 12 h. The resulting crude material was purified by column chromatography (30% *t*BuOMe/Pentane+1% Et_3N) to afford the desired product (50 mg, 13% yield from imine) as a pale-yellow oil. ^{1}H NMR (400 MHz, CD_3OD) δ 5.95 (dd, *J* = 6.3, 1.8 Hz, 1H), 3.67 (dd, *J* = 6.3, 3.1 Hz, 1H), 3.60 (s, 3H), 3.15 (ddd, *J* = 9.5, 4.5, 3.1 Hz, 1H), 2.69 (dq, *J* = 12.2, 7.3 Hz, 1H), 2.54 (dq, *J* = 12.2, 7.3 Hz, 1H), 2.03 (q, *J* = 6.4 Hz, 1H), 1.74 (d, *J* = 1.7 Hz, 3H), 1.69 (dd, *J* = 13.0, 4.5 Hz, 1H), 1.44 (dd, *J* = 13.0, 9.5 Hz, 1H), 1.12 (t, *J* = 7.2 Hz, 3H), 1.09 (s, 3H), 0.84 (d, *J* = 6.4 Hz, 3H). ^{13}C NMR (101 MHz, CD_3OD) δ 174.9, 144.4, 124.3, 64.3, 53.4, 50.9, 48.2, 39.9, 36.6, 34.5, 18.7, 18.5, 17.5, 12.7. HRMS (ESI+, m/z) calcd for $C_{14}H_{24}NO_2$ $(M+H)^+$: 238.1802, found: 238.1798.

Synthesis of #002

・**Synthesis of Imine**

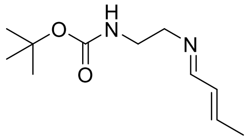

General procedure A was followed using *tert*-butyl *N*-(2-aminoethyl)carbamate (500 mg, 3.12 mmol, 1.0 equiv), *trans*-crotonaldehyde (1.29 mL, 15.6 mmol, 5.0 equiv), molecular sieves 4 Å, and THF (6 mL). Imine (662 mg, 99% yield) was obtained as a pale-yellow oil. ^{1}H NMR (400 MHz, $CDCl_3$) δ 7.82 (d, *J* = 6.9 Hz, 1H), 6.28 – 6.12 (m, 2H), 4.77 (br, 1H), 3.50 (t, *J* = 5.8 Hz, 2H), 3.35 (q, *J* = 5.9 Hz, 2H), 1.87 (d, *J* = 5.1 Hz, 3H), 1.42 (s, 9H).

・**Key Reaction**

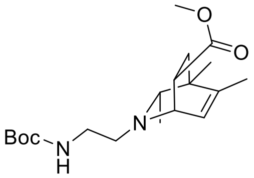

General procedure B was followed using above obtained imine (50 mg, 0.236 mmol, 1.0 equiv) and 2-butyne (93 μL, 1.18 mmol, 5 equiv) in THF (0.24 mL). Rh catalyst (10 mol %, 0.24 mL, 0.024 mmol, 100 mM in THF) was added, and the reaction was carried out at 65 °C for 12 h to produce the DHP intermediate. Following general procedure C, methyl acrylate (212 μL, 2.36 mmol, 10 equiv) was added to the solution of DHP intermediate, and the reaction was carried out at 50 °C for 16 h. The resulting crude material was purified by column chromatography (30% *t*BuOMe/Pentane+1% Et_3N) to afford the desired product (45 mg, 54% yield from imine) as a pale-yellow oil. ^{1}H NMR (400 MHz, $CDCl_3$) δ 5.94 (dd, *J* = 6.4, 1.9 Hz, 1H), 4.97 (s, 1H), 3.58 (s, 3H), 3.54 (dd, *J* = 6.4, 3.1 Hz, 1H), 3.27 – 3.07 (m, 2H), 3.02 (ddd, *J* = 9.5, 4.6, 3.1 Hz, 1H), 2.70 – 2.51 (m, 2H), 1.95 (q, *J* = 6.2 Hz, 1H), 1.76 – 1.64 (m, 4H), 1.46 – 1.31 (m, 10H), 1.03 (s, 3H), 0.76 (d, *J* = 6.2 Hz, 3H).

・**Deprotection**

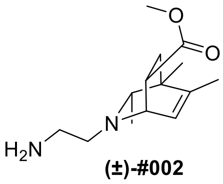

(±)-#002

Final Compound #002 General Procedure D was followed using protected isoquinuclidine (45 mg, 0.128 mmol, 1.0 equiv) in CH_2Cl_2 (1.3 mL) and was reacted with trifluoroacetic acid (95 μL, 1.28 mmol, 10.0 equiv) for 12 h. The crude product was purified by preparative thin-layer chromatography (10% MeOH/CH_2Cl_2 + 1% NH_4OH) to yield the racemic isoquinuclidine (25 mg, 78% yield) as a pale-yellow oil. ^{1}H NMR (400 MHz, CD_3OD) δ 5.95 (dd, *J* = 6.3, 1.8 Hz, 1H), 3.66 – 3.52 (m, 4H),

3.16 (ddd, *J* = 9.5, 4.6, 3.0 Hz, 1H), 2.83 – 2.56 (m, 4H), 2.05 (q, *J* = 6.3 Hz, 1H), 1.73 (d, *J* = 1.8 Hz, 3H), 1.70 (dd, *J* = 13.0, 4.6 Hz, 1H), 1.45 (dd, *J* = 13.0, 9.5 Hz, 1H), 1.09 (s, 3H), 0.81 (d, *J* = 6.3 Hz, 3H). ^{13}C NMR (101 MHz, CD_3OD) δ 175.0, 143.7, 124.5, 64.5, 55.6, 54.2, 50.8, 40.0, 39.3, 37.4, 34.6, 18.8, 18.7, 17.5. HRMS (ESI+, m/z) calcd for $C_{14}H_{25}N_2O_2$ $(M+H)^+$: 253.1911, found: 253.1913.

Synthesis of #003

・**Key Reaction**

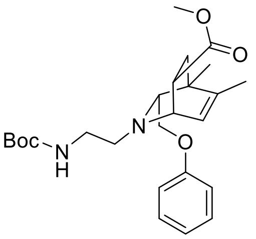

General procedure B was followed using above obtained imine (150 mg, 0.707 mmol, 1.0 equiv) and 2-butyne (277 µL, 3.53 mmol, 5 equiv) in THF (0.71 mL). Rh catalyst (10 mol %, 0.71 mL, 0.071 mmol, 100 mM in THF) was added, and the reaction was carried out at 65 °C for 12 h to produce the DHP intermediate. Following general procedure C, methyl acrylate (636 µL, 7.07 mmol, 10 equiv) was added to the solution of DHP intermediate, and the reaction was carried out at 50 °C for 16 h. The resulting crude material was purified by column chromatography (30% *t*BuOMe/Pentane+1% Et_3N) to afford the desired product (70 mg, 22% yield from imine) as a pale-yellow oil. ^{1}H NMR (400 MHz, CD_3OD) δ 7.28 – 7.15 (m, 2H), 6.94 – 6.78 (m, 3H), 5.97 (dd, *J* = 6.3, 1.8 Hz, 1H), 3.73 (dd, *J* = 9.8, 5.5 Hz, 1H), 3.68 (dd, *J* = 6.4, 3.1 Hz, 1H), 3.61 (s, 3H), 3.57 – 3.49 (m, 1H), 3.25 – 3.13 (m, 3H), 2.81 – 2.70 (m, 2H), 2.39 (t, *J* = 5.3 Hz, 1H), 1.79 – 1.67 (m, 4H), 1.55 (dd, *J* = 13.1, 9.5 Hz, 1H), 1.46 – 1.32 (m, 10H), 1.20 (s, 3H). HRMS (ESI+, m/z) calcd for $C_{25}H_{37}N_2O_5$ $(M+H)^+$: 445.2697, found: 445.2706.

・**Deprotection**

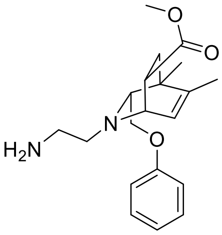

(±)-#003

Final Compound #003 General Procedure D was followed using protected isoquinuclidine (70 mg, 0.157 mmol, 1.0 equiv) in CH_2Cl_2 (1.6 mL) and was reacted with trifluoroacetic acid (120 µL, 1.57 mmol, 10.0 equiv) for 12 h. The crude product was purified by preparative thin-layer chromatography

(10% $MeOH/CH_2Cl_2$ + 1% NH_4OH) to yield the racemic isoquinuclidine (38 mg, 70% yield) as a pale-yellow oil. ^{1}H NMR (500 MHz, CD_3OD) δ 7.31 – 7.17 (m, 2H), 6.96 – 6.81 (m, 3H), 6.00 (dd, *J* = 6.3, 1.9 Hz, 1H), 3.81 (dd, *J* = 9.7, 5.0 Hz, 1H), 3.71 (dd, *J* = 6.4, 3.2 Hz, 1H), 3.63 (s, 3H), 3.60 – 3.52 (m, 1H), 3.24 (dt, *J* = 9.6, 3.9 Hz, 1H), 2.92 – 2.77 (m, 4H), 2.44 (t, *J* = 5.3 Hz, 1H), 1.81 – 1.70 (m, 4H), 1.60 (dd, *J* = 13.1, 9.4 Hz, 1H), 1.24 (s, 3H). ^{13}C NMR (126 MHz, CD_3OD) δ 174.9, 158.7, 144.1, 129.1, 124.9, 120.5, 114.1, 70.6, 68.1, 54.7, 53.8, 50.9, 39.2, 38.9, 37.4, 34.5, 19.0, 17.1. HRMS (ESI+, m/z) calcd for $C_{20}H_{29}N_2O_3$ $(M+H)^+$: 345.2173, found: 345.2166

Synthesis of #004

・**Synthesis of 0.8 M imine solution**

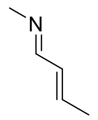

To a flame-dried vial was added a magnetic stir bar, *trans*-crotonaldehyde (0.6 mL, 7.3 mmol, 1.0 equiv) in dry THF (1.2 mL), and molecular sieves 4 Å. To this solution was then slowly added 2.0 M methylamine in THF (7.3 mL, 14.6 mmol, 2.0 equiv) at 0 °C and the reaction solution was stirred at 0 °C for 2 h. Upon reaction completion, the 0.8 M imine solution was stored at –20 °C in a nitrogen filled glovebox and taken on to the next step without further purification.

・**Key Reaction**

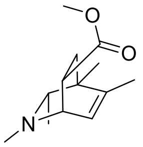

(±)-#004

Final Compound #004 General procedure B was followed using above obtained 0.8 M solution of imine (3 mL, 2.4 mmol, 1.0 equiv) and 2-butyne (941 μL, 8.0 mmol, 5 equiv) in THF (0.6 mL). Rh catalyst (10 mol %, 2.4 mL, 0.24 mmol, 100 mM in THF) was added, and the reaction was carried out at 65 °C for 12 h to produce the DHP intermediate. Following general procedure C, methyl acrylate (2.16 mL, 24.0 mmol, 10 equiv) was added to the solution of DHP intermediate, and the reaction was carried out at 50 °C for 12 h. The resulting crude material was purified by column chromatography (30% *t*BuOMe/Pentane+1% Et_3N) to afford the desired product (42 mg, 8% yield from imine) as a pale-yellow oil. ^{1}H NMR (400 MHz, CD_3OD) δ 5.99 (dd, *J* = 6.2, 1.8 Hz, 1H), 3.61 (s, 3H), 3.46 (dd, *J* = 6.2, 2.9 Hz, 1H), 3.21 (ddd, *J* = 9.5, 5.1, 2.9 Hz, 1H), 2.38 (s, 3H), 1.97 (q, *J* = 6.3 Hz, 1H), 1.74 (d, *J* = 1.8 Hz, 3H), 1.66 (dd, *J* = 12.9, 5.1 Hz, 1H), 1.47 (dd, *J* = 12.9, 9.5 Hz, 1H), 1.08 (s, 3H), 0.82 (d, *J* = 6.3 Hz, 3H). ^{13}C NMR (101 MHz, CD_3OD) δ 174.9, 143.4, 124.9, 64.7, 56.2, 50.9, 39.9, 39.2,

36.3, 35.4, 18.5, 17.4, 16.8. HRMS (ESI+, m/z) calcd for $C_{13}H_{21}NO_2$ $(M+H)^+$: 224.1645, found: 224.1633.

Synthesis of #005

・**Synthesis of Imine**

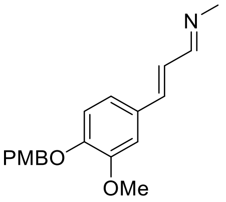

General procedure A was followed using 2.0 M methylamine in THF (0.8 mL, 1.6 mmol, 2.0 equiv), (*E*)-3-(3-methoxy-4-((4-methoxybenzyl)oxy)phenyl)acrylaldehyde (239 mg, 0.80 mmol, 1.0 equiv), molecular sieves 4 Å, and THF (1.6 mL). Imine (245 mg, 98% yield) was obtained as a yellow solid. ^{1}H NMR (400 MHz, $CDCl_3$) δ 7.98 (d, J = 7.4 Hz, 1H), 7.33 (d, J = 8.3 Hz, 2H), 7.04 (s, 1H), 6.95 (d, J = 8.5 Hz, 1H), 6.89 – 6.83 (m, 5H), 5.08 (s, 2H), 3.87 (s, 3H), 3.79 (s, 3H), 3.41 (s, 3H).

・**Key Reaction**

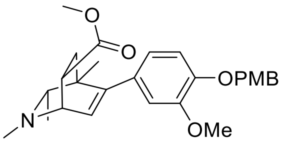

General procedure B was followed using above obtained imine (245 mg, 0.787 mmol, 1.0 equiv) and 2-butyne (308 μL, 3.93 mmol, 5 equiv) in THF (0.79 mL). Rh catalyst (10 mol %, 0.79 mL, 0.079 mmol, 100 mM in THF) was added, and the reaction was carried out at 65 °C for 12 h to produce the DHP intermediate. Following general procedure C, methyl acrylate (0.71 mL, 7.87 mmol, 10 equiv) was added to the solution of DHP intermediate, and the reaction was carried out at 50 °C for 16 h. The resulting crude material was purified by column chromatography (50% *t*BuOMe/Pentane+1% Et_3N) to afford the desired product (150 mg, 42% yield from imine) as a pale-yellow oil. ^{1}H NMR (400 MHz, CD_3OD) δ 7.34 (d, J = 8.7 Hz, 2H), 6.92 (d, J = 8.1 Hz, 1H), 6.89 (d, J = 8.5 Hz, 2H), 6.70 (d, J = 1.9 Hz, 1H), 6.65 (dd, J = 8.2, 1.9 Hz, 1H), 6.12 (d, J = 6.3 Hz, 1H), 4.98 (s, 2H), 3.80 (s, 3H), 3.77 (s, 3H), 3.69 – 3.61 (m, 4H), 3.35 (ddd, J = 9.8, 4.9, 3.1 Hz, 1H), 2.46 (s, 3H), 2.07 (q, J = 6.3 Hz, 1H), 1.90 (dd, J = 13.0, 4.9 Hz, 1H), 1.60 (dd, J = 13.0, 9.4 Hz, 1H), 0.99 (s, 3H), 0.95 (d, J = 6.3 Hz, 3H).

・**Deprotection**

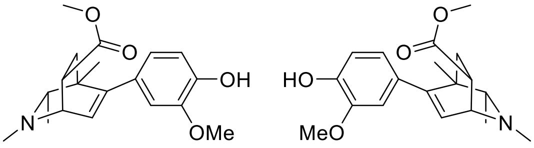

#005
enantiomers separated by chiral HPLC

Final Compound #005 General Procedure D was followed using protected isoquinuclidine (30 mg, 0.066 mmol, 1.0 equiv) in CH_2Cl_2 (1.0 mL) and was reacted with trifluoroacetic acid (49 µL, 0.66 mmol, 10.0 equiv) for 12 h. The crude product was purified by preparative thin-layer chromatography (10% MeOH/CH_2Cl_2 + 1% NH_4OH) to yield the racemic isoquinuclidine (21 mg, 95% yield) as a pale-yellow oil. ^{1}H NMR (400 MHz, CD_3OD) δ 6.73 (d, J = 8.1 Hz, 1H), 6.66 (d, J = 1.9 Hz, 1H), 6.58 (dd, J = 8.1, 2.0 Hz, 1H), 6.11 (d, J = 6.3 Hz, 1H), 3.82 (s, 3H), 3.72 (dd, J = 6.3, 3.0 Hz, 1H), 3.66 (s, 3H), 3.37 (ddd, J = 9.4, 4.9, 3.0 Hz, 1H), 2.53 (s, 3H), 2.18 (q, J = 6.4 Hz, 1H), 1.90 (dd, J = 13.0, 4.9 Hz, 1H), 1.64 (dd, J = 13.0, 9.4 Hz, 1H), 1.01 (s, 3H), 0.99 (d, J = 6.4 Hz, 3H). ^{13}C NMR (101 MHz, CD_3OD) δ 174.5, 148.9, 146.9, 145.7, 131.1, 126.8, 120.8, 114.4, 111.7, 65.4, 56.4, 54.9, 51.1, 40.8, 39.1, 36.6, 36.1, 20.2, 16.9. HRMS (ESI+, m/z) calcd for $C_{19}H_{25}NO_4$ $(M+H)^+$: 332.1856, found: 332.1827.

HPLC Traces of #005

A portion of this material was separated using semi-preparative chiral HPLC (Chiralpak IC column, 250 x 10 mm, 20% *i*PrOH/Hexanes+1% DEA, 2.5 mL/min) to provide the two enantiomers with tr = 15 min and 35 min.

Chiralpak IC, analytical column (250 x 4.6 mm), 30% *i*PrOH/Hexanes+0.1% DEA, 1 mL/min, 254 nm

Racemic

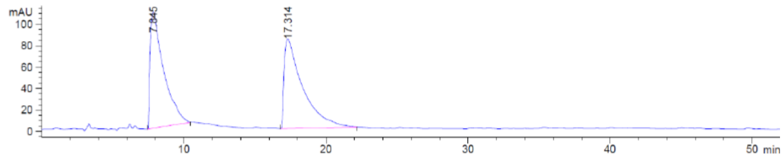

Enantiomer 1

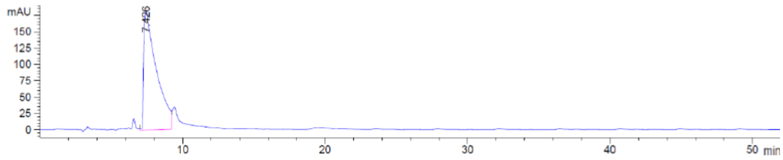

Enantiomer 2

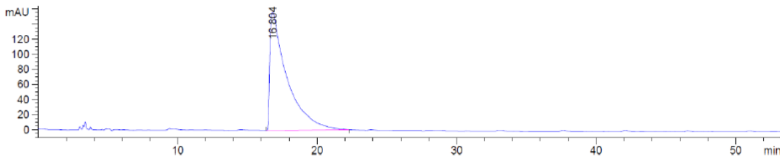

Synthesis of #006

・**Synthesis of Imine**

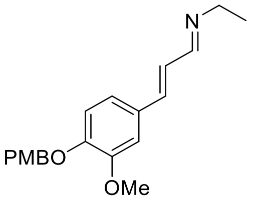

General procedure A was followed using 2.0 M ethylamine in THF (0.8 mL, 1.6 mmol, 2.0 equiv), (*E*)-3-(3-methoxy-4-((4-methoxybenzyl)oxy)phenyl)acrylaldehyde (239 mg, 0.80 mmol, 1.0 equiv), molecular sieves 4 Å, and THF (1.6 mL). Imine (248 mg, 95% yield) was obtained as a yellow solid. ^{1}H NMR (400 MHz, $CDCl_3$) δ 7.99 (d, *J* = 7.4 Hz, 1H), 7.34 (d, *J* = 8.6 Hz, 2H), 7.06 (s, 1H), 6.99 – 6.82 (m, 6H), 5.09 (s, 2H), 3.87 (s, 3H), 3.79 (s, 3H), 3.55 (q, *J* = 8.5 Hz, 2H), 1.32 – 1.21 (m, 3H).

・**Key Reaction**

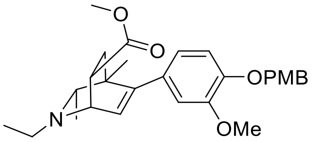

General procedure B was followed using above obtained imine (248 mg, 0.762 mmol, 1.0 equiv) and 2-butyne (299 µL, 3.81 mmol, 5 equiv) in THF (0.76 mL). Rh catalyst (10 mol %, 0.76 mL, 0.076 mmol, 100 mM in THF) was added, and the reaction was carried out at 65 °C for 12 h to produce the DHP intermediate. Following general procedure C, methyl acrylate (0.67 mL, 7.62 mmol, 10 equiv) was added to the solution of DHP intermediate, and the reaction was carried out at 50 °C for 16 h. The resulting crude material was purified by column chromatography (50% *t*BuOMe/Pentane+1% Et_3N) to afford the desired product (180 mg, 51% yield from imine) as a pale-yellow oil. ^{1}H NMR (400 MHz, CD_3OD) δ 7.34 (d, *J* = 8.7 Hz, 2H), 6.92 (d, *J* = 8.3 Hz, 1H), 6.89 (d, *J* = 8.7 Hz, 2H), 6.71 (d, *J* = 2.0 Hz, 1H), 6.66 (dd, *J* = 8.3, 2.0 Hz, 1H), 6.08 (d, *J* = 6.4 Hz, 1H), 4.99 (s, 2H), 3.85 (dd, *J* = 6.4, 3.1 Hz, 1H), 3.80 (s, 3H), 3.77 (s, 3H), 3.65 (s, 3H), 2.76 (dq, *J* = 14.1, 7.2 Hz, 1H), 2.63 (dq, *J* = 14.1, 7.2 Hz, 1H), 2.15 (q, *J* = 6.4 Hz, 1H), 1.93 (dd, *J* = 13.1, 4.3 Hz, 1H), 1.58 (dd, *J* = 13.1, 9.4 Hz, 1H), 1.18 (t, *J* = 7.2 Hz, 3H), 1.01 (s, 3H), 0.98 (d, *J* = 6.4 Hz, 3H). HRMS (ESI+, m/z) calcd for $C_{28}H_{36}NO_5$ $(M+H)^+$: 466.2588, found: 466.2602.

・**Deprotection**

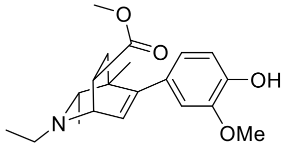
(±)-#006

Final Compound #006 General Procedure D was followed using protected isoquinuclidine (30 mg, 0.064 mmol, 1.0 equiv) in CH_2Cl_2 (1.0 mL) and was reacted with trifluoroacetic acid (48 µL, 0.64 mmol, 10.0 equiv) for 12 h. The crude product was purified by preparative thin-layer chromatography (10% MeOH/CH_2Cl_2 + 1% NH_4OH) to yield the racemic isoquinuclidine (22 mg, 99% yield) as a pale-yellow oil.

^{1}H NMR (400 MHz, CD_3OD) δ 6.72 (d, J = 8.1 Hz, 1H), 6.66 (d, J = 2.0 Hz, 1H), 6.58 (dd, J = 8.1, 2.0 Hz, 1H), 6.07 (d, J = 6.4 Hz, 1H), 3.88 (dd, J = 6.4, 3.1 Hz, 1H), 3.82 (s, 3H), 3.65 (s, 3H), 3.34 – 3.30 (m, 1H), 2.80 (dq, J = 12.2, 7.2 Hz, 1H), 2.66 (dq, J = 12.2, 7.2 Hz, 1H), 2.19 (q, J = 6.4 Hz, 1H), 1.92 (dd, J = 13.1, 4.4 Hz, 1H), 1.59 (dd, J = 13.1, 9.4 Hz, 1H), 1.19 (t, J = 7.2 Hz, 3H), 1.02 (s, 3H), 0.99 (d, J = 6.4 Hz, 3H). ^{13}C NMR (101 MHz, CD_3OD) δ 174.7, 149.6, 146.9, 145.6, 131.4, 126.4, 120.7, 114.3, 111.7, 64.9, 54.9, 53.6, 51.1, 48.3, 40.8, 36.9, 35.2, 20.5, 18.9, 12.6. HRMS (ESI+, m/z) calcd for $C_{20}H_{27}NO_4$ $(M+H)^+$: 346.2013, found: 346.1993.

Synthesis of #007

・Synthesis of Imine

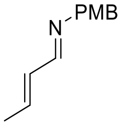

General procedure A was followed using (4-methoxyphenyl)methanamine (230 mg, 1.68 mmol, 1.0 equiv), *trans*-crotonaldehyde (277 µL, 3.35 mmol, 2.0 equiv), molecular sieves 4 Å, and THF (3.4 mL). Imine (300 mg, 95% yield) was obtained as a pale-yellow oil. ^{1}H NMR (400 MHz, $CDCl_3$) δ 7.90 (d, J = 8.1 Hz, 1H), 7.17 (d, J = 8.5 Hz, 2H), 6.85 (d, J = 8.6 Hz, 2H), 6.31 – 6.15 (m, 2H), 4.54 (s, 2H), 3.77 (s, 3H), 1.87 (d, J = 5.7 Hz, 3H).

・Key Reaction

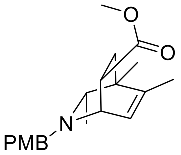

General procedure B was followed using above obtained imine (300 mg, 1.59 mmol, 1.0 equiv) and 2-butyne (621 µL, 7.93 mmol, 5 equiv) in THF (1.6 mL). Rh catalyst (10 mol %, 1.6 mL, 0.16 mmol,

100 mM in THF) was added, and the reaction was carried out at 65 °C for 12 h to produce the DHP intermediate. Following general procedure C, methyl acrylate (1.43 mL, 15.9 mmol, 10 equiv) was added to the solution of DHP intermediate, and the reaction was carried out at 50 °C for 16 h. The resulting crude material was purified by column chromatography (30% *t*BuOMe/Pentane+1% Et_3N) to afford the desired product (155 mg, 30% yield from imine) as a pale-yellow oil. ^{1}H NMR (400 MHz, $CDCl_3$) δ 7.25 (d, *J* = 8.5 Hz, 2H), 6.83 (d, *J* = 8.5 Hz, 2H), 5.92 (dd, *J* = 6.4, 1.8 Hz, 1H), 3.78 (s, 3H), 3.66 (d, *J* = 12.7 Hz, 1H), 3.58 (s, 3H), 3.56 – 3.45 (m, 2H), 3.20 (ddd, *J* = 9.5, 4.9, 3.0 Hz, 1H), 2.07 (q, *J* = 6.2 Hz, 1H), 1.80 – 1.67 (m, 4H), 1.45 (dd, *J* = 12.8, 9.5 Hz, 1H), 1.06 (s, 3H), 0.72 (d, *J* = 6.2 Hz, 3H).

・Deprotection of PMB on nitrogen

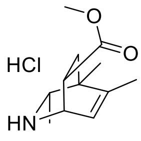

(±)-#007

Final Compound #007 General Procedure E was followed using protected isoquinuclidine (75 mg, 0.228 mmol, 1.0 equiv) in DCE (1.0 mL) and was reacted with 1-chloroethyl chloroformate (123 µL, 1.14 mmol, 5.0 equiv) for 3 h. The crude product was washed with pentane to yield the HCl salt of isoquinuclidine (28 mg, 50% yield) as a white-solid. ^{1}H NMR (400 MHz, CD_3OD) δ 6.01 (dd, *J* = 6.0, 1.8 Hz, 1H), 4.35 (dd, *J* = 5.9, 2.8 Hz, 1H), 3.66 (s, 3H), 3.28 – 3.20 (m, 2H), 1.93 – 1.81 (m, 4H), 1.69 (dd, *J* = 13.4, 5.6 Hz, 1H), 1.24 (s, 3H), 1.09 (d, *J* = 6.8 Hz, 3H). ^{13}C NMR (101 MHz, CD_3OD) δ 171.6, 148.6, 119.2, 55.9, 51.4, 48.2, 39.9, 39.3, 33.8, 17.5, 17.1, 15.2. HRMS (ESI+, m/z) calcd for $C_{12}H_{19}NO_2$ $(M+H)^+$: 210.1489, found: 210.1476.

Synthesis of #008

・Synthesis of Imine

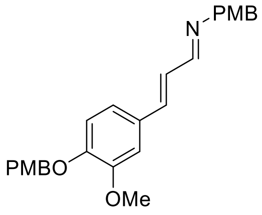

General procedure A was followed using (4-methoxyphenyl)methanamine (104 mg, 0.756 mmol, 1.1 equiv), (*E*)-3-(3-methoxy-4-((4-methoxybenzyl)oxy)phenyl)acrylaldehyde (205 mg, 0.687 mmol, 1.0 equiv), molecular sieves 4 Å, and THF (1.4 mL). Imine (280 mg, 98% yield) was obtained as a yellow solid. ^{1}H NMR (400 MHz, $CDCl_3$) δ 8.04 (d, *J* = 6.9 Hz, 1H), 7.34 (d, *J* = 8.6 Hz, 2H), 7.21 (d, *J* =

8.6 Hz, 2H), 7.03 (d, J = 2.0 Hz, 1H), 6.94 (dd, J = 8.3, 2.0 Hz, 1H), 6.91 – 6.84 (m, 7H), 5.09 (s, 2H), 4.63 (s, 2H), 3.86 (s, 3H), 3.78 (s, 6H).

・**Key Reaction**

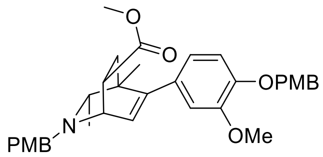

General procedure B was followed using above obtained imine (200 mg, 0.479 mmol, 1.0 equiv) and 2-butyne (188 µL, 2.40 mmol, 5 equiv) in THF (0.48 mL). Rh catalyst (10 mol %, 0.48 mL, 0.048 mmol, 100 mM in THF) was added, and the reaction was carried out at 65 °C for 12 h to produce the DHP intermediate. Following general procedure C, methyl acrylate (0.43 mL, 4.79 mmol, 10 equiv) was added to the solution of DHP intermediate, and the reaction was carried out at 50 °C for 16 h. The resulting crude material was purified by column chromatography (50% *t*BuOMe/Pentane+1% Et_3N) to afford the desired product (115 mg, 43% yield from imine) as a pale-yellow oil. ^{1}H NMR (400 MHz, CD_3OD) δ 7.35 – 7.27 (m, 4H), 6.91 – 6.85 (m, 5H), 6.70 (d, J = 1.9 Hz, 1H), 6.63 (dd, J = 8.2, 1.9 Hz, 1H), 6.00 (d, J = 6.4 Hz, 1H), 4.96 (s, 2H), 3.80 – 3.72 (m, 10H), 3.66 (dd, J = 6.4, 2.9 Hz, 1H), 3.63 – 3.54 (m, 4H), 3.43 (dt, J = 7.8, 3.8 Hz, 1H), 2.23 (q, J = 6.2 Hz, 1H), 1.93 (dd, J = 12.9, 4.5 Hz, 1H), 1.63 (dd, J = 12.9, 9.4 Hz, 1H), 0.99 (s, 3H), 0.81 (d, J = 6.2 Hz, 3H). HRMS (ESI+, m/z) calcd for $C_{34}H_{40}NO_6$ $(M+H)^+$: 558.2850, found: 558.2859.

・**Deprotection of PMB on nitrogen**

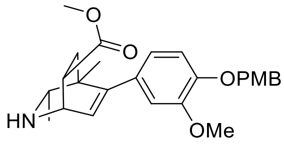

General Procedure E was followed using di-protected isoquinuclidine (25 mg, 0.045 mmol, 1.0 equiv) in DCE (1.0 mL) and was reacted with 1-chloroethyl chloroformate (24 µL, 0.224 mmol, 5.0 equiv) for 12 h. After methanol treatment according to the general procedure, the crude product was purified by preparative thin-layer chromatography (10% MeOH/CH_2Cl_2 + 1% NH_4OH) to yield the mono-protected isoquinuclidine (8.5 mg, 40% yield) as a pale-yellow oil. ^{1}H NMR (400 MHz, $CDCl_3$) δ 7.35 (d, J = 8.7 Hz, 2H), 6.88 (d, J = 8.7 Hz, 2H), 6.81 (d, J = 8.2 Hz, 1H), 6.66 (d, J = 2.0 Hz, 1H), 6.62 (dd, J = 8.2, 2.0 Hz, 1H), 6.15 (d, J = 5.8 Hz, 1H), 5.05 (s, 2H), 3.92 (dd, J = 5.8, 2.9 Hz, 1H), 3.85 (s, 3H), 3.79 (s, 3H), 3.64 (s, 3H), 3.09 (ddd, J = 9.8, 5.2, 2.9 Hz, 1H), 2.82 (q, J = 6.4 Hz, 1H), 1.82 (dd, J = 12.9, 5.2 Hz, 1H), 1.70 (dd, J = 12.9, 9.8 Hz, 1H), 1.06 (s, 3H), 0.88 (d, J = 6.4 Hz, 3H).

・**Deprotection of PMB on oxygen**

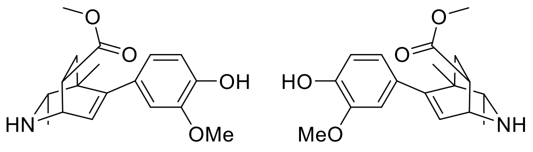

#008
enantiomers separated by chiral HPLC

Final Compound #008 General Procedure D was followed using mono-protected isoquinuclidine (8.5 mg, 0.019 mmol, 1.0 equiv) in CH_2Cl_2 (1.0 mL) and was reacted with trifluoroacetic acid (15 µL, 0.19 mmol, 10.0 equiv) for 12 h. The crude product was purified by preparative thin-layer chromatography (10% MeOH/CH_2Cl_2 + 1% NH_4OH) to yield the racemic isoquinuclidine (6.1 mg, 99% yield) as a dark-brown oil. ^{1}H NMR (400 MHz, CD_3OD) δ 6.72 (d, J = 8.0 Hz, 1H), 6.66 (d, J = 2.0 Hz, 1H), 6.58 (dd, J = 8.0, 2.0 Hz, 1H), 6.06 (d, J = 5.7 Hz, 1H), 3.87 (dd, J = 5.7, 3.0 Hz, 1H), 3.82 (s, 3H), 3.64 (s, 3H), 3.05 (ddd, J = 9.6, 5.0, 3.0 Hz, 1H), 2.75 (q, J = 6.4 Hz, 1H), 1.82 (dd, J = 13.0, 5.0 Hz, 1H), 1.68 (dd, J = 13.0, 9.6 Hz, 1H), 1.05 (s, 3H), 0.91 (d, J = 6.4 Hz, 3H). ^{13}C NMR (126 MHz, CD_3OD) δ 174.2, 147.9, 146.9, 145.5, 131.8, 126.7, 120.9, 114.4, 111.9, 56.3, 55.0, 50.9, 49.0, 44.0, 40.3, 36.4, 19.9, 18.2. HRMS (ESI+, m/z) calcd for $C_{18}H_{23}NO_4$ $(M+H)^+$: 318.1700, found: 318.1674.

HPLC Traces of #008

A portion of this material was separated using semi-preparative chiral HPLC (Chiralpak IC column, 250 x 10 mm, 20% *i*PrOH/Hexanes+1% DEA, 2.5 mL/min) to provide the two enantiomers with tr = 18.5 min and 25 min.

Chiralpak IC, analytical column (250 x 4.6 mm), 20% *i*PrOH/Hexanes+0.1% DEA, 1 mL/min, 254 nm

Racemic

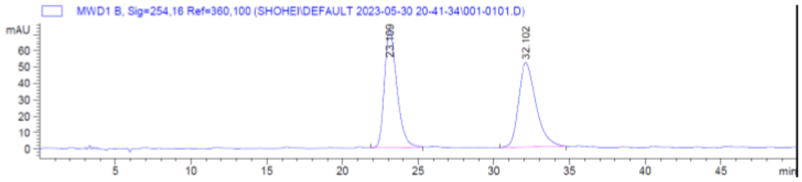

Enantiomer 1

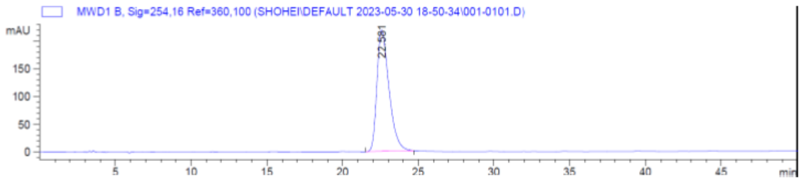

Enantiomer 2

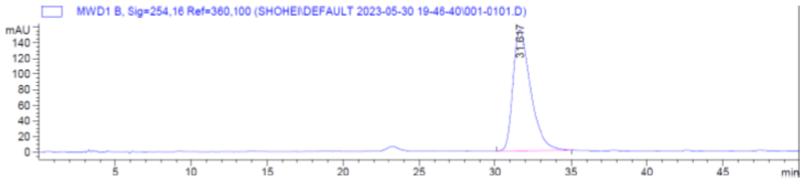

Synthesis of #009

・**Key Reaction**

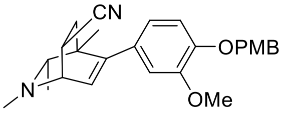

General procedure B was followed using above obtained imine (225 mg, 0.723 mmol, 1.0 equiv) and 2-butyne (283 µL, 3.61 mmol, 5 equiv) in THF (0.72 mL). Rh catalyst (10 mol %, 0.72 mL, 0.072 mmol, 100 mM in THF) was added, and the reaction was carried out at 65 °C for 12 h to produce the DHP intermediate. Following general procedure C, acrylonitrile (383 mg, 7.23 mmol, 10 equiv) was added to the solution of DHP intermediate, and the reaction was carried out at 50 °C for 16 h. The resulting crude material was purified by column chromatography (70% EtOAc/Hexane+1% Et_3N) to afford the desired product (133 mg, 44% yield from imine) as a pale-yellow oil. ^{1}H NMR (400 MHz, CD_3OD) δ 7.34 (d, *J* = 8.6 Hz, 2H), 6.95 (d, *J* = 8.2 Hz, 1H), 6.89 (d, *J* = 8.6 Hz, 2H), 6.76 (d, *J* = 2.0 Hz, 1H), 6.71 (dd, *J* = 8.2, 2.0 Hz, 1H), 6.37 (d, *J* = 6.3 Hz, 1H), 4.99 (s, 2H), 3.81 (s, 3H), 3.76 (s, 3H), 3.63 (dd, *J* = 6.3, 2.8 Hz, 1H), 3.51 (ddd, *J* = 9.7, 4.7, 2.8 Hz, 1H), 2.43 (s, 3H), 2.06 (q, *J* = 6.4 Hz, 1H), 1.94 (dd, *J* = 13.1, 9.7 Hz, 1H), 1.62 (dd, *J* = 13.1, 4.7 Hz, 1H), 1.00 (s, 3H), 0.93 (d, *J* = 6.4 Hz, 3H).

・**Deprotection**

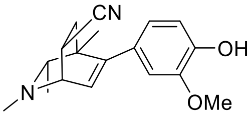

(±)-#009

Final Compound #009 General Procedure D was followed using protected isoquinuclidine (42 mg, 0.787 mmol, 1.0 equiv) in CH_2Cl_2 (1.0 mL) and was reacted with trifluoroacetic acid (75 µL, 1.0 mmol, 10.0 equiv) for 12 h. The crude product was purified by preparative thin-layer chromatography (10% MeOH/CH_2Cl_2 + 1% NH_4OH) to yield the racemic isoquinuclidine (27 mg, 90% yield) as a pale-yellow oil. ^{1}H NMR (400 MHz, CD_3OD) δ 6.76 (d, *J* = 8.1 Hz, 1H), 6.71 (d, *J* = 2.0 Hz, 1H), 6.64 (dd, *J* = 8.1, 2.0 Hz, 1H), 6.35 (d, *J* = 6.4 Hz, 1H), 3.83 (s, 3H), 3.62 (dd, *J* = 6.4, 2.8 Hz, 1H), 3.50 (ddd, *J* = 9.7, 4.8, 2.8 Hz, 1H), 2.42 (s, 3H), 2.06 (q, *J* = 6.3 Hz, 1H), 1.94 (dd, *J* = 13.1, 9.7 Hz, 1H), 1.61 (dd, *J* = 13.1, 4.8 Hz, 1H), 1.01 (s, 3H), 0.93 (d, *J* = 6.3 Hz, 3H). ^{13}C NMR (101 MHz, CD_3OD) δ 149.1, 147.1, 145.9, 130.9, 127.7, 122.2, 120.8, 114.5, 111.7, 64.4, 55.7, 54.9, 40.2, 39.1, 38.6, 22.1, 19.9, 17.3. HRMS (ESI+, m/z) calcd for $C_{18}H_{23}N_2O_2$ $(M+H)^+$: 299.1754, found: 299.1759.

Synthesis of #010

・**Key Reaction**

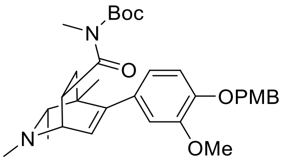

General procedure B was followed using above obtained imine (225 mg, 0.723 mmol, 1.0 equiv) and 2-butyne (283 μL, 3.61 mmol, 5 equiv) in THF (0.72 mL). Rh catalyst (10 mol %, 0.72 mL, 0.072 mmol, 100 mM in THF) was added, and the reaction was carried out at 65 °C for 12 h to produce the DHP intermediate. Following general procedure C, Boc-protected *N*-methyl acrylamide (669 mg, 3.61 mmol, 5 equiv) was added to the solution of DHP intermediate, and the reaction was carried out at 50 °C for 16 h. The resulting crude material was purified by column chromatography (50% EtOAc/Hexane+1% Et_3N) to afford the desired product (125 mg, 31% yield from imine) as a pale-yellow oil. ^{1}H NMR (400 MHz, CD_3OD) δ 7.33 (d, J = 8.6 Hz, 2H), 6.91 (d, J = 8.2 Hz, 1H), 6.89 (d, J = 8.6 Hz, 2H), 6.75 (d, J = 2.0 Hz, 1H), 6.68 (dd, J = 8.2, 2.0 Hz, 1H), 6.07 (d, J = 6.4 Hz, 1H), 4.98 (s, 2H), 4.48 (ddd, J = 9.3, 5.3, 2.5 Hz, 1H), 3.80 (s, 3H), 3.76 (s, 3H), 3.56 (dd, J = 6.4, 2.5 Hz, 1H), 3.08 (s, 3H), 2.49 (s, 3H), 2.07 (q, J = 6.3 Hz, 1H), 1.92 (dd, J = 12.6, 5.3 Hz, 1H), 1.60 (dd, J = 12.6, 9.3 Hz, 1H), 1.54 (s, 9H), 0.98 (s, 3H), 0.96 (d, J = 6.3 Hz, 3H). HRMS (ESI+, m/z) calcd for $C_{32}H_{43}N_2O_6$ $(M+H)^+$: 551.3116, found: 551.3121.

・**Deprotection**

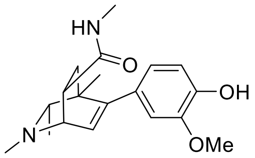

(±)-#010

Final Compound #010 General Procedure D was followed using protected isoquinuclidine (43 mg, 0.078 mmol, 1.0 equiv) in CH_2Cl_2 (1.0 mL) and was reacted with trifluoroacetic acid (58 μL, 0.780 mmol, 10.0 equiv) for 12 h. The crude product was purified by preparative thin-layer chromatography (10% MeOH/CH_2Cl_2 + 1% NH_4OH) to yield the racemic isoquinuclidine (25 mg, 97% yield) as a pale yellow oil. ^{1}H NMR (400 MHz, CD_3OD) δ 6.73 (d, J = 1.9 Hz, 1H), 6.71 (d, J = 8.1 Hz, 1H), 6.62 (dd, J = 8.1, 1.9 Hz, 1H), 6.10 (d, J = 6.3 Hz, 1H), 3.82 (s, 3H), 3.46 (dd, J = 6.3, 2.7 Hz, 1H), 3.18 (ddd, J = 9.4, 5.3, 2.7 Hz, 1H), 2.69 (s, 3H), 2.46 (s, 3H), 2.03 (q, J = 6.3 Hz, 1H), 1.85 (dd, J = 12.6, 5.3 Hz, 1H), 1.52 (dd, J = 12.6, 9.4 Hz, 1H), 0.99 (s, 3H), 0.95 (d, J = 6.3 Hz, 3H). ^{13}C NMR (101 MHz, CD_3OD) δ 175.2, 147.5, 146.9, 145.5, 131.6, 127.6, 120.8, 114.3, 111.9, 64.9, 57.3, 54.9, 40.9, 39.1,

37.2, 36.8, 25.2, 20.4, 17.3. HRMS (ESI+, m/z) calcd for $C_{19}H_{27}N_2O_3$ $(M+H)^+$: 331.2016, found: 331.2023.

Synthesis of #011

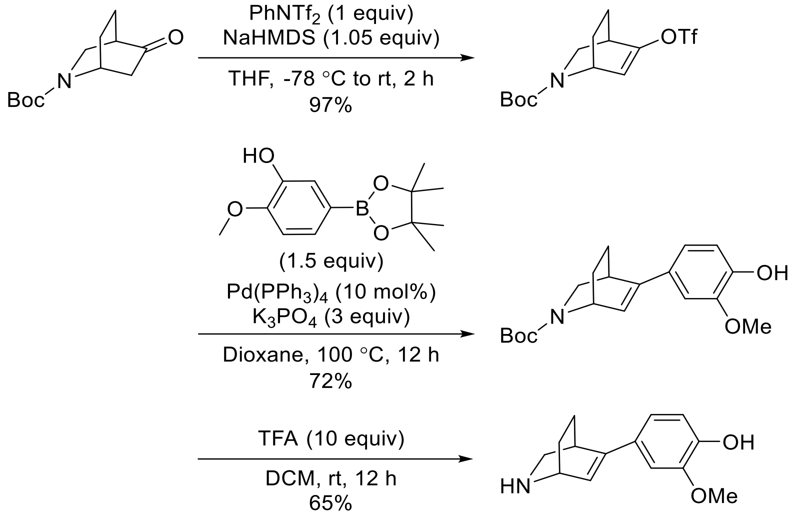

To a flame-dried round-bottom flask was added a magnetic stir bar, 2-azabicyclo[2.2.2]octane-2-carboxylic acid 5-oxo-1,1-dimethylethyl ester[3] (900 mg, 3.99 mmol, 1 equiv) and *N*-phenyl-bis(trifluoromethanesulfonimide) (1570 mg, 4.39 mmol, 1.1 equiv) dissolved in THF (8 mL). This solution was cooled to -78 ° C and then 1.0 M sodium hexamethyldisilazane in THF (4.2 mL, 4.19 mmol, 1.05 equiv) was added dropwise over 5 min. The reaction mixture was stirred at room temperature for 2 h. After the reaction was complete as monitored by thin-layer chromatography, the mixture was then diluted with water, extracted with ethyl acetate, washed with brine, dried with Na_2SO_4 and filtered. The organic layer was concentrated and the crude residue was purified via silica gel chromatography (gradient of 0-50% EtOAc in hexane) to give 1390 mg of the *O*-protected compound in 97% yield. To a stirred solution of the above obtained compound (500 mg, 1.4 mmol, 1 equiv) in dioxane (7 mL) was added K_3PO_4 (891 mg, 4.20 mmol, 3 equiv), 4-hydroxy-3-methoxyphenylboronicacid pinacol ester (525 mg, 2.1 mmol, 1.5 equiv), $Pd(PPh_3)_4$ (162 mg, 0.14 mmol, 10 mol%) and the reaction mixture was heated under reflux for 12 h. After the reaction was complete as monitored by thin-layer chromatography, the mixture was then diluted with water, extracted with ethyl acetate, washed with brine, dried with Na_2SO_4 and filtered. The organic layer was concentrated and the crude residue was purified via silica gel chromatography (gradient of 0-100% EtOAc in hexane) to give 335 mg of the arylated compound in 72% yield. ^1H NMR (400 MHz, CD_3OD) δ 7.00 (s, 1H), 6.90 (d, J = 8.2 Hz, 1H), 6.75 (dd, J = 8.3, 1.1 Hz, 1H), 6.54 (d, J = 7.0 Hz, 1H), 4.75 – 4.64 (m, 1H), 3.84 (s, 3H), 3.40 – 3.21 (m, 2H), 3.04 – 2.89 (m, 1H), 2.07 – 1.91 (m, 1H), 1.82 – 1.68 (m, 1H), 1.49 – 1.39 (m, 11H). HRMS (ESI+, m/z) calcd for $C_{19}H_{26}NO_4$ $(M+H)^+$:

332.1856, found: 332.1851.

Final Compound #011 General Procedure D was followed using above obtained isoquinuclidine (100 mg, 0.302 mmol, 1.0 equiv) in CH_2Cl_2 (3.0 mL) and was reacted with trifluoroacetic acid (224 µL, 3.02 mmol, 10.0 equiv) for 12 h. The crude product was purified by preparative thin-layer chromatography (10% MeOH/CH_2Cl_2 + 1% NH_4OH) to yield the racemic isoquinuclidine (45 mg, 65% yield) as a dark-brown oil. ^{1}H NMR (400 MHz, CD_3OD) δ 7.01 (d, J = 2.0 Hz, 1H), 6.91 (dd, J = 8.2, 2.1 Hz, 1H), 6.74 (d, J = 8.2 Hz, 1H), 6.53 (dd, J = 6.1, 1.9 Hz, 1H), 3.84 (s, 3H), 3.77 (dt, J = 5.9, 2.7 Hz, 1H), 3.21 (q, J = 2.4 Hz, 1H), 3.00 (dd, J = 10.5, 2.0 Hz, 1H), 2.58 (dt, J = 10.5, 2.6 Hz, 1H), 2.10 – 1.91 (m, 1H), 1.89 – 1.68 (m, 1H), 1.52 – 1.36 (m, 2H). ^{13}C NMR (101 MHz, CD_3OD) δ 148.0, 147.2, 145.1, 128.9, 123.0, 117.7, 115.1, 108.1, 54.9, 46.7, 44.7, 31.7, 25.2, 22.6. HRMS (ESI+, m/z) calcd for $C_{14}H_{18}NO_2$ $(M+H)^+$: 232.1332, found: 232.1309.

Synthesis of #012

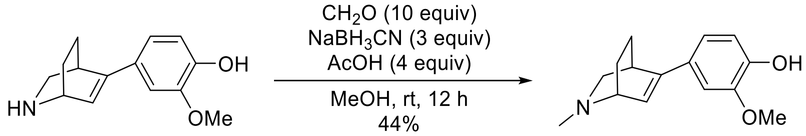

Final Compound #012 The solution of the above obtained compound (45 mg, 0.156 mmol, 1 equiv) in MeOH (3 mL) was added AcOH (36 µL, 0.622 mmol, 4 equiv), formaldehyde (0.14 mL of 37% w/w solution, 4.67 mmol, 10 equiv), and $NaBH_3CN$ (29 mg, 0.467 mmol, 3 equiv) and the reaction mixture was stirred at room temperature for 12 h. After the reaction was complete as monitored by thin-layer chromatography, the mixture was then diluted with water, extracted with CH_2Cl_2, washed with brine, dried with Na_2SO_4 and filtered. The organic layer was concentrated and the crude residue was purified by preparative thin-layer chromatography (5% MeOH/CH_2Cl_2 + 1% NH_4OH) to yield the racemic isoquinuclidine (21 mg, 45% yield) as a white solid. ^{1}H NMR (500 MHz, CD_3OD) δ 7.07 (s, 1H), 6.97 (dd, J = 8.2, 1.9 Hz, 1H), 6.78 (d, J = 8.2 Hz, 1H), 6.50 (dd, J = 5.8, 1.9 Hz, 1H), 3.88 (s, 3H), 3.76 (dt, J = 5.8, 2.9 Hz, 1H), 3.28 – 3.23 (m, 2H), 2.45 (s, 3H), 2.32 (dt, J = 11.1, 2.9 Hz, 1H), 2.17 – 2.09 (m, 1H), 1.80 – 1.68 (m, 1H), 1.55 – 1.45 (m, 1H), 1.45 – 1.34 (m, 1H). ^{13}C NMR (126 MHz, CD_3OD) δ 147.9, 146.9, 146.3, 128.3, 119.7, 118.0, 114.9, 108.3, 55.3, 55.3, 55.0, 42.6, 32.2, 23.9, 20.6. HRMS (ESI+, m/z) calcd for $C_{15}H_{20}NO_2$ $(M+H)^+$: 246.1489, found: 246.1465.

Synthesis of #013

・**Synthesis of Imine**

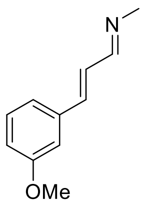

General procedure A was followed using 2.0 M methylamine in THF (1.67 mL, 3.33 mmol, 2.0 equiv), (*E*)-3-methoxycinnamaldehyde (270 mg, 1.66 mmol, 1.0 equiv), molecular sieves 4 Å, and CH_2Cl_2 (1.67 mL). Imine (285 mg, 98% yield) was obtained as a yellow solid. ^{1}H NMR (400 MHz, $CDCl_3$) δ 8.00 (dd, *J* = 8.3, 1.6 Hz, 1H), 7.28 (t, *J* = 7.9 Hz, 1H), 7.08 (d, *J* = 7.8 Hz, 1H), 7.05 – 6.95 (m, 3H), 6.89 (dd, *J* = 8.3, 2.5 Hz, 1H), 3.81 (s, 3H), 3.44 (d, *J* = 1.6 Hz, 3H).

・**Key Reaction**

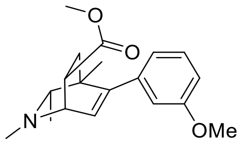

(±)-#013

Final Compound #013 General procedure B was followed using above obtained imine (140 mg, 0.799 mmol, 1.0 equiv) and 2-butyne (313 µL, 3.99 mmol, 5 equiv) in THF (0.8 mL). Rh catalyst (10 mol %, 0.8 mL, 0.08 mmol, 100 mM in THF) was added, and the reaction was carried out at 65 °C for 12 h to produce the DHP intermediate. Following general procedure C, methyl acrylate (0.72 mL, 7.99 mmol, 10 equiv) was added to the solution of DHP intermediate, and the reaction was carried out at 50 °C for 16 h. The resulting crude material was purified by column chromatography (50% EtOAc/Hexane+1% Et_3N) to yield the racemic isoquinuclidine (72 mg, 29% yield) as a pale-yellow oil. ^{1}H NMR (500 MHz, CD_3OD) δ 7.21 (t, *J* = 7.9 Hz, 1H), 6.83 (dd, *J* = 8.3, 2.6 Hz, 1H), 6.71 (d, *J* = 7.7 Hz, 1H), 6.70 – 6.64 (m, 1H), 6.15 (d, *J* = 6.3 Hz, 1H), 3.77 (s, 3H), 3.66 – 3.64 (m, 4H), 3.35 (ddd, *J* = 9.5, 4.9, 2.9 Hz, 1H), 2.47 (s, 3H), 2.07 (q, *J* = 6.3 Hz, 1H), 1.92 (dd, *J* = 12.9, 4.9 Hz, 1H), 1.61 (dd, *J* = 12.9, 9.5 Hz, 1H), 0.98 (s, 3H), 0.97 (d, *J* = 6.3 Hz, 3H). ^{13}C NMR (126 MHz, CD_3OD) δ 174.8, 159.3, 148.3, 141.1, 128.6, 128.1, 120.3, 113.9, 111.9, 65.1, 56.4, 54.2, 51.0, 40.7, 39.4, 36.7, 36.3, 20.3, 17.4. HRMS (ESI+, m/z) calcd for $C_{19}H_{26}NO_3$ $(M+H)^+$: 316.1907, found: 316.1877.

Synthesis of #014

・**Synthesis of Imine**

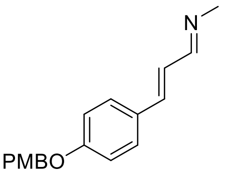

General procedure A was followed using 2.0 M methylamine in THF (0.56 mL, 1.12 mmol, 2.0 equiv), (*E*)-3-(4-(4-methoxybenzyloxy)phenyl)acrylaldehyde[4] (150 mg, 0.559 mmol, 1.0 equiv), molecular sieves 4 Å, and CH_2Cl_2 (0.56 mL). Imine (150 mg, 95% yield) was obtained as a yellow solid. ^1H NMR (400 MHz, $CDCl_3$) δ 7.97 (d, J = 8.5 Hz, 1H), 7.41 (d, J = 8.7 Hz, 2H), 7.34 (d, J = 8.6 Hz, 2H), 7.01 – 6.86 (m, 6H), 5.00 (s, 2H), 3.80 (s, 3H), 3.40 (s, 3H).

・**Key Reaction**

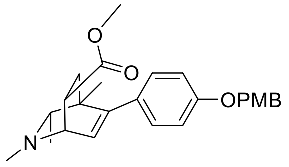

General procedure B was followed using above obtained imine (150 mg, 0.533 mmol, 1.0 equiv) and 2-butyne (209 µL, 2.67 mmol, 5 equiv) in THF (0.53 mL). Rh catalyst (10 mol %, 0.53 mL, 0.053 mmol, 100 mM in THF) was added, and the reaction was carried out at 65 °C for 12 h to produce the DHP intermediate. Following general procedure C, methyl acrylate (0.48 mL, 5.33 mmol, 10 equiv) was added to the solution of DHP intermediate, and the reaction was carried out at 50 °C for 16 h. The resulting crude material was purified by column chromatography (50% EtOAc/Hexane+1% Et_3N) to afford the desired product (110 mg, 49% yield from imine) as a pale-yellow oil. ^1H NMR (400 MHz, CD_3OD) δ 7.33 (d, J = 8.6 Hz, 2H), 7.04 (d, J = 8.6 Hz, 2H), 6.90 (dd, J = 8.7, 2.9 Hz, 4H), 6.10 (d, J = 6.3 Hz, 1H), 4.96 (s, 2H), 3.77 (s, 3H), 3.67 – 3.61 (m, 4H), 3.37 – 3.32 (m, 1H), 2.46 (s, 3H), 2.07 (q, J = 6.3 Hz, 1H), 1.88 (dd, J = 12.9, 4.9 Hz, 1H), 1.60 (dd, J = 12.9, 9.5 Hz, 1H), 0.98 (s, 3H), 0.95 (d, J = 6.3 Hz, 3H). HRMS (ESI+, m/z) calcd for $C_{26}H_{32}NO_4$ $(M+H)^+$: 422.2326, found: 422.2333.

・**Deprotection**

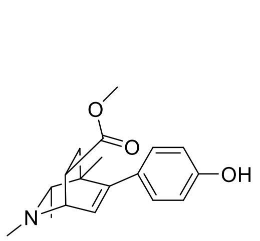

(±)-#014

Final Compound #014 General Procedure D was followed using protected isoquinuclidine (60 mg, 0.142 mmol, 1.0 equiv) in CH_2Cl_2 (1.5 mL) and was reacted with trifluoroacetic acid (106 µL, 1.42 mmol, 10.0 equiv) for 12 h. The crude product was purified by preparative thin-layer chromatography (10% MeOH/CH_2Cl_2 + 1% NH_4OH) to yield the racemic isoquinuclidine (42 mg, 98% yield) as a

pale-yellow oil. ^{1}H NMR (400 MHz, CD_3OD) δ 6.95 (d, *J* = 8.5 Hz, 2H), 6.70 (d, *J* = 8.5 Hz, 2H), 6.07 (d, *J* = 6.3 Hz, 1H), 3.64 (s, 3H), 3.61 (dd, *J* = 6.3, 3.0 Hz, 1H), 3.36 – 3.30 (m, 1H), 2.44 (s, 3H), 2.04 (q, *J* = 6.3 Hz, 1H), 1.87 (dd, *J* = 12.9, 4.9 Hz, 1H), 1.58 (dd, *J* = 12.9, 9.4 Hz, 1H), 0.97 (s, 3H), 0.94 (d, *J* = 6.3 Hz, 3H). ^{13}C NMR (101 MHz, CD_3OD) δ 174.9, 156.4, 148.4, 130.9, 129.0, 127.3, 114.3, 65.1, 56.4, 51.0, 40.8, 39.4, 36.7, 36.2, 20.4, 17.4. HRMS (ESI+, m/z) calcd for $C_{18}H_{24}NO_3$ $(M+H)^+$: 302.1751, found: 302.1721.

Synthesis of #015

・**Key Reaction**

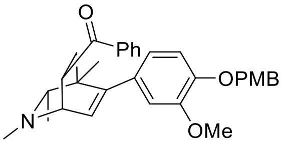

General procedure B was followed using above obtained imine (150 mg, 0.482 mmol, 1.0 equiv) and 2-butyne (189 µL, 2.41 mmol, 5 equiv) in THF (0.48 mL). Rh catalyst (10 mol %, 0.48 mL, 0.048 mmol, 100 mM in THF) was added, and the reaction was carried out at 65 °C for 12 h to produce the DHP intermediate. Following general procedure C, phenylvinyl ketone (318 mg, 2.41 mmol, 5 equiv) was added to the solution of DHP intermediate, and the reaction was carried out at 50 °C for 16 h. The resulting crude material was purified by column chromatography (50% EtOAc/Hexane+1% Et_3N) to afford the desired product (25 mg, 10% yield from imine) as a pale-yellow oil. ^{1}H NMR (400 MHz, CD_3OD) δ 7.96 (d, *J* = 7.1 Hz, 2H), 7.59 (dd, *J* = 8.4, 6.3 Hz, 1H), 7.55 – 7.45 (m, 2H), 7.34 (d, *J* = 8.6 Hz, 2H), 6.92 (d, *J* = 8.4 Hz, 1H), 6.89 (d, *J* = 8.6 Hz, 2H), 6.74 (d, *J* = 2.0 Hz, 1H), 6.67 (dd, *J* = 8.2, 2.0 Hz, 1H), 6.02 (d, *J* = 6.3 Hz, 1H), 4.98 (s, 2H), 4.41 (ddd, *J* = 8.7, 5.4, 2.5 Hz, 1H), 3.81 (s, 3H), 3.77 (s, 3H), 3.61 (dd, *J* = 6.4, 2.5 Hz, 1H), 2.63 (s, 3H), 2.23 (q, *J* = 6.3 Hz, 1H), 1.99 (dd, *J* = 12.6, 5.4 Hz, 1H), 1.71 (dd, *J* = 12.6, 9.2 Hz, 1H), 1.03 (s, 3H), 0.99 (d, *J* = 6.3 Hz, 3H).

・**Deprotection**

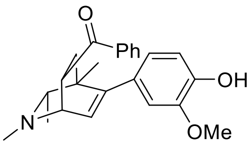

(±)-#015

Final Compound #015 General Procedure D was followed using protected isoquinuclidine (25 mg, 0.05 mmol, 1.0 equiv) in CH_2Cl_2 (1.0 mL) and was reacted with trifluoroacetic acid (37 µL, 0.50 mmol, 10.0 equiv) for 12 h. The crude product was purified by preparative thin-layer chromatography (10% MeOH/CH_2Cl_2 + 1% NH_4OH) to yield the racemic isoquinuclidine (18 mg, 95% yield) as a pale-

yellow oil. ^{1}H NMR (500 MHz, CD_3OD) δ 7.97 (d, *J* = 7.1 Hz, 2H), 7.68 – 7.57 (m, 1H), 7.52 (dd, *J* = 8.4, 7.1 Hz, 2H), 6.74 (d, *J* = 8.1 Hz, 1H), 6.70 (d, *J* = 2.0 Hz, 1H), 6.61 (dd, *J* = 8.1, 2.0 Hz, 1H), 6.00 (d, *J* = 6.4 Hz, 1H), 4.47 – 4.35 (m, 1H), 3.84 (s, 3H), 3.60 (dd, *J* = 6.4, 2.5 Hz, 1H), 2.63 (s, 3H), 2.21 (q, *J* = 6.3 Hz, 1H), 2.01 (dd, *J* = 12.6, 5.3 Hz, 1H), 1.69 (dd, *J* = 12.6, 9.2 Hz, 1H), 1.04 (s, 3H), 1.00 (d, *J* = 6.3 Hz, 3H). ^{13}C NMR (126 MHz, CD_3OD) δ 200.7, 147.6, 146.9, 145.6, 136.5, 132.8, 131.4, 128.5, 127.9, 127.4, 120.8, 114.4, 111.9, 65.2, 57.1, 54.9, 41.2, 40.4, 39.3, 36.4, 20.5, 17.3. HRMS (ESI+, m/z) calcd for $C_{24}H_{28}NO_3$ $(M+H)^+$: 378.2064, found: 378.2026.

Synthesis of #016

・**Synthesis of Imine**

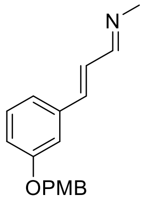

General procedure A was followed using 2.0 M methylamine in THF (0.73 mL, 1.45 mmol, 2.0 equiv), (*E*)-3-[3-(4-methoxy-benzyloxy)-phenyl]-propenal[5] (195 mg, 0.727 mmol, 1.0 equiv), molecular sieves 4 Å, and CH_2Cl_2 (0.73 mL). Imine (200 mg, 98% yield) was obtained as a yellow solid. ^{1}H NMR (400 MHz, $CDCl_3$) δ 8.00 (d, *J* = 7.3 Hz, 1H), 7.34 (d, *J* = 8.3 Hz, 2H), 7.29 – 7.21 (m, 1H), 7.09 – 7.01 (m, 2H), 6.95 – 6.82 (m, 5H), 4.98 (s, 2H), 3.80 (s, 3H), 3.41 (d, *J* = 1.5 Hz, 3H).

・**Key Reaction**

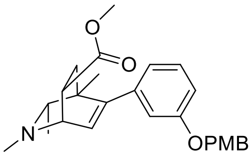

General procedure B was followed using above obtained imine (200 mg, 0.711 mmol, 1.0 equiv) and 2-butyne (279 µL, 3.55 mmol, 5 equiv) in THF (0.71 mL). Rh catalyst (10 mol %, 0.71 mL, 0.071 mmol, 100 mM in THF) was added, and the reaction was carried out at 65 °C for 12 h to produce the DHP intermediate. Following general procedure C, methyl acrylate (0.64 mL, 7.11 mmol, 10 equiv) was added to the solution of DHP intermediate, and the reaction was carried out at 50 °C for 16 h. The resulting crude material was purified by column chromatography (50% EtOAc/Hexane+1% Et_3N) to afford the desired product (155 mg, 52% yield from imine) as a pale-yellow oil. ^{1}H NMR (400 MHz, CD_3OD) δ 7.32 (d, *J* = 8.6 Hz, 2H), 7.18 (t, *J* = 8.1 Hz, 1H), 6.95 – 6.82 (m, 3H), 6.74 – 6.61 (m, 2H), 6.11 (d, *J* = 6.3 Hz, 1H), 4.97 (s, 2H), 3.76 (s, 3H), 3.70 – 3.59 (m, 4H), 3.41 – 3.33 (m, 1H), 2.45 (s,

3H), 2.05 (q, *J* = 6.3 Hz, 1H), 1.88 (dd, *J* = 13.0, 4.9 Hz, 1H), 1.59 (dd, *J* = 13.0, 9.4 Hz, 1H), 0.93 (d, *J* = 6.3 Hz, 3H), 0.91 (s, 3H).

・**Deprotection**

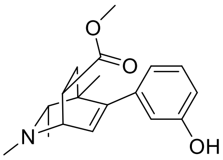

(±)-#016

Final Compound #016 General Procedure D was followed using protected isoquinuclidine (30 mg, 0.071 mmol, 1.0 equiv) in CH_2Cl_2 (1.0 mL) and was reacted with trifluoroacetic acid (53 µL, 0.71 mmol, 10.0 equiv) for 12 h. The crude product was purified by preparative thin-layer chromatography (10% MeOH/CH_2Cl_2 + 1% NH_4OH) to yield the racemic isoquinuclidine (21 mg, 98% yield) as a pale-yellow oil. ^{1}H NMR (400 MHz, CD_3OD) δ 7.09 (t, *J* = 7.9 Hz, 1H), 6.72 – 6.63 (m, 1H), 6.58 (dd, *J* = 7.2, 1.5 Hz, 2H), 6.11 (d, *J* = 6.3 Hz, 1H), 3.69 – 3.56 (m, 4H), 3.37 – 3.30 (m, 1H), 2.45 (s, 3H), 2.05 (q, *J* = 6.4 Hz, 1H), 1.89 (dd, *J* = 12.9, 4.8 Hz, 1H), 1.59 (dd, *J* = 12.9, 9.4 Hz, 1H), 0.97 (s, 3H), 0.95 (d, *J* = 6.4 Hz, 3H). ^{13}C NMR (101 MHz, CD_3OD) δ 174.8, 156.7, 148.5, 141.0, 128.5, 127.7, 119.2, 114.8, 113.7, 65.1, 56.3, 51.0, 40.7, 39.4, 36.7, 36.3, 20.3, 17.4. HRMS (ESI+, m/z) calcd for $C_{18}H_{24}NO_3$ $(M+H)^+$: 302.1751, found: 302.1756.

Synthesis of #017

・**Synthesis of Imine**

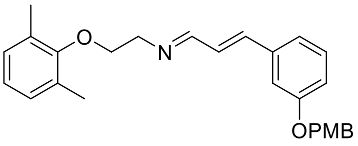

General procedure A was followed using 2-(2,6-dimethylphenoxy)ethanamine (68 mg, 0.410 mmol, 1.1 equiv), (*E*)-3-[3-(4-methoxy-benzyloxy)-phenyl]-propenal (100 mg, 0.373 mmol, 1.0 equiv), molecular sieves 4 Å, and CH_2Cl_2 (0.75 mL). Imine (154 mg, 99% yield) was obtained as a pale-yellow oil. ^{1}H NMR (400 MHz, $CDCl_3$) δ 8.15 (d, *J* = 6.0 Hz, 1H), 7.35 (dd, *J* = 8.7, 2.0 Hz, 2H), 7.30 – 7.25 (m, 1H), 7.14 – 7.04 (m, 2H), 7.04 – 6.81 (m, 8H), 4.99 (s, 2H), 4.02 (t, *J* = 5.2 Hz, 2H), 3.91 (t, *J* = 5.2 Hz, 2H), 3.81 (s, 3H), 2.25 (s, 6H).

・**Key Reaction**

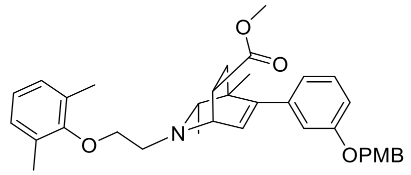

General procedure B was followed using above obtained imine (155 mg, 0.373 mmol, 1.0 equiv) and 2-butyne (146 µL, 1.87 mmol, 5 equiv) in THF (0.37 mL). Rh catalyst (10 mol %, 0.37 mL, 0.037 mmol, 100 mM in THF) was added, and the reaction was carried out at 65 °C for 12 h to produce the DHP intermediate. Following general procedure C, methyl acrylate (0.34 mL, 3.73 mmol, 10 equiv) was added to the solution of DHP intermediate, and the reaction was carried out at 50 °C for 16 h. The resulting crude material was purified by column chromatography (50% EtOAc/Hexane+1% Et_3N) to afford the desired product (90 mg, 43% yield from imine) as a pale-yellow oil. ^{1}H NMR (400 MHz, CD_3OD) δ 7.32 (d, J = 8.6 Hz, 2H), 7.19 (t, J = 8.1 Hz, 1H), 6.98 (d, J = 7.4 Hz, 2H), 6.94 – 6.82 (m, 4H), 6.76 – 6.66 (m, 2H), 6.10 (d, J = 6.3 Hz, 1H), 4.98 (s, 2H), 4.03 – 3.85 (m, 3H), 3.76 (s, 3H), 3.64 (s, 3H), 3.40 (dt, J = 9.5, 4.0 Hz, 1H), 3.17 – 2.99 (m, 2H), 2.28 (s, 6H), 2.24 (q, J = 6.3 Hz, 1H), 1.94 (dd, J = 13.1, 4.4 Hz, 1H), 1.63 (dd, J = 13.1, 9.5 Hz, 1H), 0.97 (d, J = 6.3 Hz, 3H), 0.95 (s, 3H).

・Deprotection

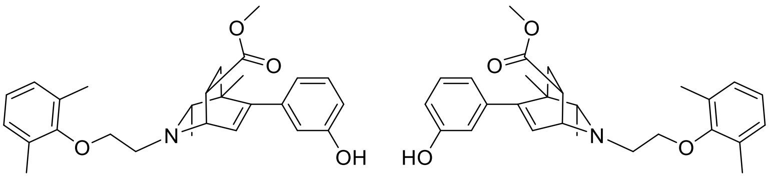

#017
enantiomers separated by chiral HPLC

Final Compound #017 General Procedure D was followed using protected isoquinuclidine (90 mg, 0.162 mmol, 1.0 equiv) in CH_2Cl_2 (1.6 mL) and was reacted with trifluoroacetic acid (120 µL, 1.62 mmol, 10.0 equiv) for 12 h. The crude product was purified by preparative thin-layer chromatography (10% MeOH/CH_2Cl_2 + 1% NH_4OH) to yield the racemic isoquinuclidine (55 mg, 78% yield) as a pale-yellow oil. ^{1}H NMR (400 MHz, CD_3OD) δ 7.09 (t, J = 8.1 Hz, 1H), 6.97 (d, J = 7.4 Hz, 2H), 6.87 (dd, J = 8.1, 6.8 Hz, 1H), 6.71 – 6.64 (m, 1H), 6.59 (dd, J = 6.8, 1.5 Hz, 2H), 6.10 (d, J = 6.3 Hz, 1H), 4.04 – 3.82 (m, 3H), 3.63 (s, 3H), 3.39 (ddd, J = 9.4, 4.4, 3.1 Hz, 1H), 3.15 – 2.99 (m, 2H), 2.28 (s, 6H), 2.23 (q, J = 6.3 Hz, 1H), 1.95 (dd, J = 13.0, 4.4 Hz, 1H), 1.63 (dd, J = 13.0, 9.4 Hz, 1H), 1.00 (s, 3H), 0.99 (d, J = 6.3 Hz, 3H). ^{13}C NMR (126 MHz, CD_3OD) δ 174.9, 156.6, 155.9, 148.8, 141.2, 130.4, 129.4, 128.5, 127.4, 123.6, 119.2, 114.8, 113.6, 70.9, 65.3, 54.8, 54.1, 51.0, 40.8, 37.7, 35.7, 20.6, 19.1, 15.3. HRMS (ESI+, m/z) calcd for $C_{27}H_{34}NO_4$ $(M+H)^+$: 436.2482, found: 436.2495.

HPLC Traces of #017

A portion of this material was separated using semi-preparative chiral HPLC (Chiralpak IC column,

250 x 10 mm, 10% *i*PrOH/Hexanes+1% DEA, 2.5 mL/min) to provide the two enantiomers with tr = 26.5 min and 55.5 min.

Chiralpak IC, analytical column (250 x 4.6 mm), 10% *i*PrOH/Hexanes+0.1% DEA, 1 mL/min, 254 nm

Racemic

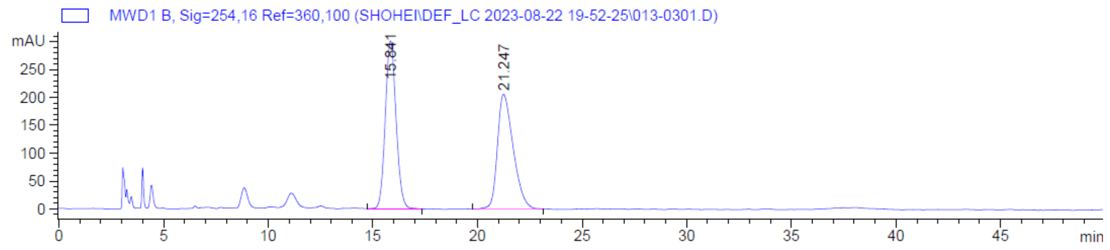

Enantiomer 1

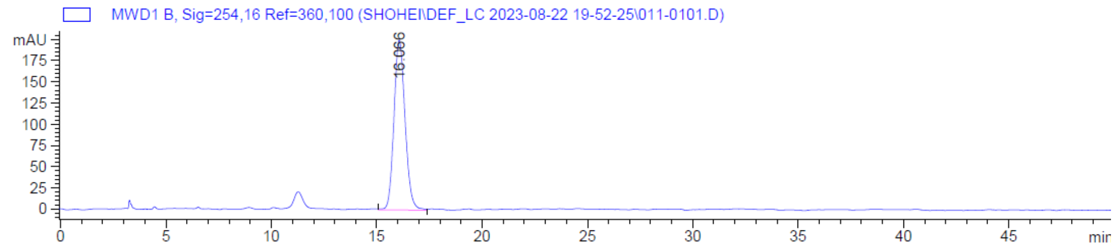

Enantiomer 2

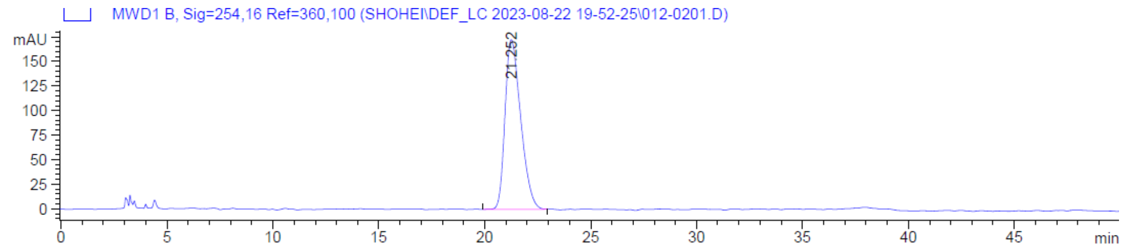

Synthesis of #018

・**Synthesis of Imine**

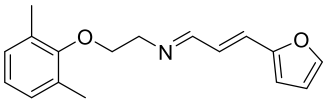

General procedure A was followed using 2-(2,6-dimethylphenoxy)ethanamine (127 mg, 0.766 mmol, 1.1 equiv), (*E*)-3-(2-furyl)propenal (85 mg, 0.696 mmol, 1.0 equiv), molecular sieves 4 Å, and THF (1.4 mL). Imine (187 mg, 99% yield) was obtained as a pale-yellow oil. ^{1}H NMR (400 MHz, $CDCl_3$) δ 8.07 (dd, *J* = 8.1, 1.4 Hz, 1H), 7.44 (d, *J* = 1.8 Hz, 1H), 6.97 (d, *J* = 7.1 Hz, 2H), 6.89 (dd, *J* = 8.2,

6.6 Hz, 1H), 6.85 – 6.69 (m, 2H), 6.48 (d, *J* = 3.4 Hz, 1H), 6.43 (dd, *J* = 3.4, 1.8 Hz, 1H), 4.01 (t, *J* = 5.5 Hz, 2H), 3.88 (t, *J* = 5.5 Hz, 2H), 2.25 (s, 6H).

・**Key Reaction**

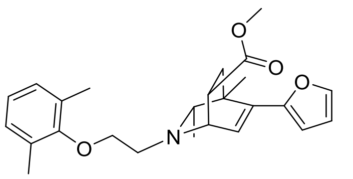

(±)-#018

Final Compound #018 General procedure B was followed using above obtained imine (187 mg, 0.694 mmol, 1.0 equiv) and 2-butyne (272 µL, 3.47 mmol, 5 equiv) in THF (0.7 mL). Rh catalyst (10 mol %, 0.7 mL, 0.07 mmol, 100 mM in THF) was added, and the reaction was carried out at 65 °C for 12 h to produce the DHP intermediate. Following general procedure C, methyl acrylate (0.63 mL, 6.94 mmol, 10 equiv) was added to the solution of DHP intermediate, and the reaction was carried out at 50 °C for 16 h. The resulting crude material was purified by column chromatography (50% EtOAc/Hexane+1% Et_3N) to afford the desired product (101 mg, 36% yield from imine) as a pale-yellow oil. ^{1}H NMR (500 MHz, CD_3OD) δ 7.41 (d, *J* = 1.8 Hz, 1H), 6.98 (d, *J* = 7.5 Hz, 2H), 6.88 (t, *J* = 7.5 Hz, 1H), 6.59 (d, *J* = 6.5 Hz, 1H), 6.43 – 6.31 (m, 2H), 4.02 (dd, *J* = 6.6, 3.0 Hz, 1H), 3.98 – 3.79 (m, 2H), 3.61 (s, 3H), 3.44 – 3.35 (m, 1H), 3.17 – 2.95 (m, 2H), 2.28 (s, 6H), 2.22 (q, *J* = 6.3 Hz, 1H), 1.91 (dd, *J* = 13.1, 4.7 Hz, 1H), 1.62 (dd, *J* = 13.1, 9.7 Hz, 1H), 1.34 (s, 3H), 0.89 (d, *J* = 6.3 Hz, 3H). ^{13}C NMR (126 MHz, CD_3OD) δ 174.7, 155.9, 152.2, 141.6, 136.8, 130.4, 128.5, 127.9, 126.9, 123.6, 119.3, 110.6, 107.4, 71.0, 65.1, 54.3, 53.8, 51.0, 40.3, 37.1, 35.7, 20.9, 18.6, 15.3. HRMS (ESI+, m/z) calcd for $C_{25}H_{32}NO_4$ $(M+H)^+$: 410.2326, found: 410.2337.

Synthesis of #019

・**Synthesis of Imine**

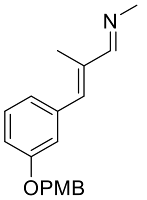

General procedure A was followed using 2.0 M methylamine in THF (3.72 mL, 7.44 mmol, 5.0 equiv), (*E*)-3-[3-[(4-methoxyphenyl)methoxy]phenyl]-2-methyl-prop-2-enal[6] (420 mg, 1.49 mmol, 1.0 equiv), molecular sieves 4 Å, and CH_2Cl_2 (3.0 mL) at 50 °C. Imine (435 mg, 99% yield) was obtained as a yellow solid. ^{1}H NMR (400 MHz, $CDCl_3$) δ 7.96 (d, *J* = 1.7 Hz, 1H), 7.35 (d, *J* = 8.5 Hz, 2H), 7.28 (t,

J = 8.1 Hz, 1H), 7.03 – 6.93 (m, 2H), 6.93 – 6.86 (m, 3H), 6.73 (s, 1H), 4.99 (s, 2H), 3.80 (s, 3H), 3.43 (s, 3H), 2.08 (s, 3H).

・**Key Reaction**

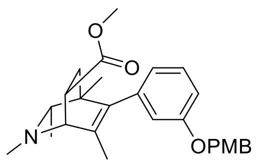

General procedure B was followed using above obtained imine (155 mg, 0.525 mmol, 1.0 equiv) and 2-butyne (206 μL, 2.62 mmol, 5 equiv) in THF (0.53 mL). Rh catalyst (10 mol %, 0.53 mL, 0.053 mmol, 100 mM in THF) was added, and the reaction was carried out at 65 °C for 12 h to produce the DHP intermediate. Following general procedure C, methyl acrylate (0.47 mL, 5.25 mmol, 10 equiv) was added to the solution of DHP intermediate, and the reaction was carried out at 50 °C for 16 h. The resulting crude material was purified by column chromatography (50% EtOAc/Hexane+1% Et_3N) to afford the desired product (65 mg, 28% yield from imine) as a pale-yellow oil. ^{1}H NMR (400 MHz, CD_3OD) δ 7.31 (d, J = 8.1 Hz, 2H), 7.20 (t, J = 7.9 Hz, 1H), 6.96 – 6.78 (m, 3H), 6.69 – 6.52 (m, 2H), 4.97 (s, 2H), 3.75 (s, 3H), 3.66 (s, 3H), 3.45 (d, J = 3.0 Hz, 1H), 3.36 – 3.30 (m, 1H), 2.46 (s, 3H), 2.03 (q, J = 6.3 Hz, 1H), 1.90 (dd, J = 12.9, 4.7 Hz, 1H), 1.61 – 1.45 (m, 4H), 0.94 (d, J = 6.3 Hz, 3H), 0.72 (s, 3H). LRMS (ESI+, m/z) calcd for $C_{27}H_{34}NO_4$ $(M+H)^+$: 436.2, found: 437.0.

・**Deprotection**

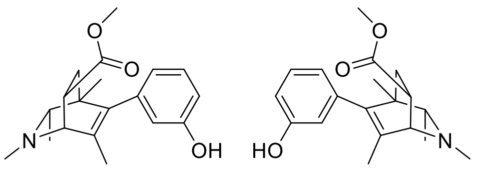

#019
enantiomers separated by chiral HPLC

Final Compound #019 General Procedure D was followed using protected isoquinuclidine (65 mg, 0.149 mmol, 1.0 equiv) in CH_2Cl_2 (1.5 mL) and was reacted with trifluoroacetic acid (111 μL, 1.49 mmol, 10.0 equiv) for 12 h. The crude product was purified by preparative thin-layer chromatography (10% MeOH/CH_2Cl_2 + 1% NH_4OH) to yield the racemic isoquinuclidine (33 mg, 70% yield) as a pale-yellow oil. ^{1}H NMR (500 MHz, CD_3OD) δ 7.13 (t, J = 7.8 Hz, 1H), 6.67 (d, J = 8.1 Hz, 1H), 6.59 – 6.42 (m, 2H), 3.68 (s, 3H), 3.48 (d, J = 2.4 Hz, 1H), 3.40 – 3.34 (m, 1H), 2.49 (s, 3H), 2.07 (q, J = 6.3 Hz, 1H), 1.94 (dd, J = 12.9, 4.7 Hz, 1H), 1.64 – 1.53 (m, 4H), 1.00 (d, J = 6.3 Hz, 3H), 0.82 (s, 3H). ^{13}C NMR (126 MHz, CD_3OD, 50 °C) δ 174.7, 156.7, 139.9, 139.6, 134.6, 128.5, 120.0, 115.6, 113.3, 65.8, 61.9, 50.8, 40.6, 39.6, 36.9, 36.6, 20.3, 17.4, 16.5. HRMS (ESI+, m/z) calcd for $C_{19}H_{26}NO_3$ $(M+H)^+$: 316.1907, found: 316.1915.

HPLC Traces of #019

A portion of this material was separated using semi-preparative chiral HPLC (Chiralpak IC column, 250 x 10 mm, 5% *i*PrOH/Hexanes+1% DEA, 2.5 mL/min) to provide the two enantiomers with tr = 15 min and 25.5 min.

Chiralpak IC, analytical column (250 x 4.6 mm), 5% *i*PrOH/Hexanes+0.1% DEA, 1 mL/min, 210 nm

Racemic

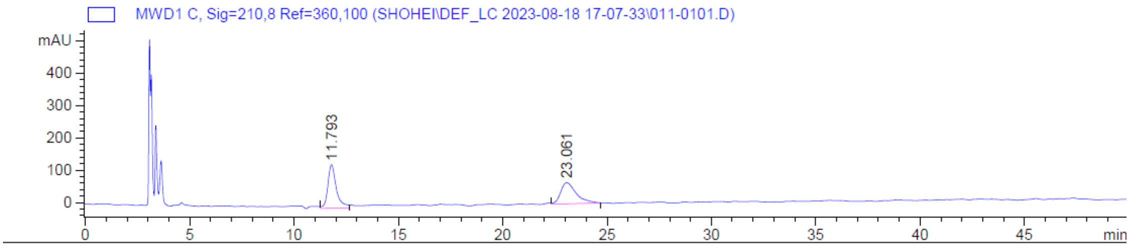

Enantiomer 1

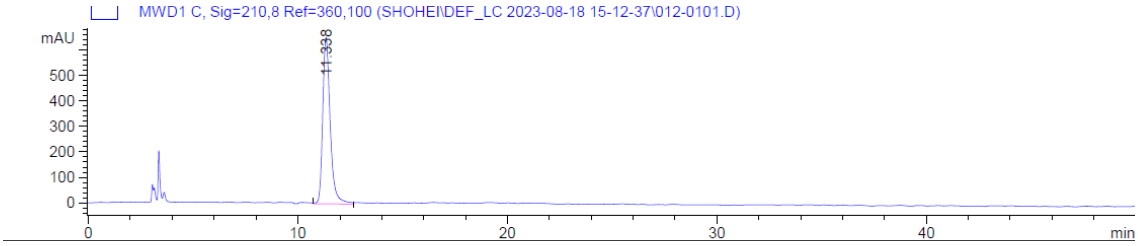

Enantiomer 2

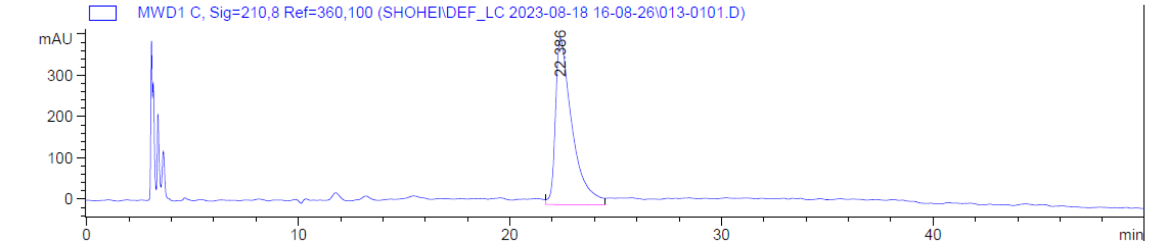

Synthesis of #020

・**Synthesis of Imine**

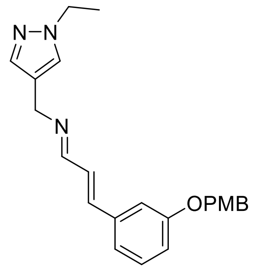

General procedure A was followed using (1-ethylpyrazol-4-yl)methanamine (52 mg, 0.418 mmol, 1.1 equiv), (*E*)-3-[3-(4-Methoxy-benzyloxy)-phenyl]-propenal[5] (102 mg, 0.380 mmol, 1.0 equiv), molecular sieves 4 Å, and CH_2Cl_2 (0.76 mL). Imine (137 mg, 97% yield) was obtained as a pale-yellow oil. ^{1}H NMR (400 MHz, $CDCl_3$) δ 8.07 (dd, *J* = 6.4, 1.8 Hz, 1H), 7.41 (s, 1H), 7.35 (s, 1H), 7.33 (d, *J* = 1.9 Hz, 2H), 7.27 – 7.23 (m, 1H), 7.08 – 7.02 (m, 2H), 6.95 – 6.87 (m, 5H), 4.98 (s, 2H), 4.57 (s, 2H), 4.13 (q, *J* = 7.3 Hz, 2H), 3.80 (s, 3H), 1.46 (t, *J* = 7.3 Hz, 3H).

・**Key Reaction**

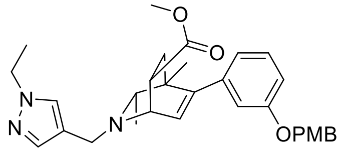

General procedure B was followed using above obtained imine (143 mg, 0.381 mmol, 1.0 equiv) and 2-butyne (149 µL, 1.90 mmol, 5 equiv) in THF (0.38 mL). Rh catalyst (10 mol %, 0.38 mL, 0.038 mmol, 100 mM in THF) was added, and the reaction was carried out at 65 °C for 12 h to produce the DHP intermediate. Following general procedure C, methyl acrylate (0.34 mL, 3.81 mmol, 10 equiv) was added to the solution of DHP intermediate, and the reaction was carried out at 50 °C for 16 h. The resulting crude material was purified by column chromatography (70% EtOAc/Hexane+1% Et_3N) to afford the desired product (75 mg, 38% yield from imine) as a pale-yellow oil. ^{1}H NMR (400 MHz, CD_3OD) δ 7.62 (s, 1H), 7.50 (s, 1H), 7.30 (d, *J* = 8.6 Hz, 2H), 7.17 (t, *J* = 8.1 Hz, 1H), 6.93 – 6.81 (m, 3H), 6.76 – 6.62 (m, 2H), 6.02 (d, *J* = 6.4 Hz, 1H), 4.95 (s, 2H), 4.13 (q, *J* = 7.3 Hz, 2H), 3.78 – 3.72 (m, 5H), 3.62 (s, 3H), 3.56 (d, *J* = 13.3 Hz, 1H), 3.43 – 3.36 (m, 1H), 2.20 (q, *J* = 6.3 Hz, 1H), 1.92 (dd, *J* = 13.0, 4.5 Hz, 1H), 1.61 (dd, *J* = 13.0, 9.3 Hz, 1H), 1.41 (t, *J* = 7.3 Hz, 3H), 0.92 (s, 3H), 0.84 (d, *J* = 6.3 Hz, 3H). ^{13}C NMR (101 MHz, CD_3OD) δ 174.9, 159.5, 158.3, 149.2, 141.1, 139.5, 129.6, 129.2, 128.8, 128.5, 127.6, 120.5, 117.5, 114.7, 113.5, 113.4, 69.4, 64.4, 54.3, 52.8, 51.1, 46.7, 46.4, 40.9, 36.9, 35.8, 20.3, 18.5, 14.7. HRMS (ESI+, m/z) calcd for $C_{31}H_{38}N_3O_4$ $(M+H)^+$: 516.2857, found: 516.2837.

・**Deprotection**

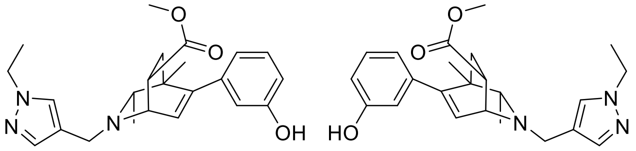

#020
enantiomers separated by chiral HPLC

Final Compound #020 General Procedure D was followed using protected isoquinuclidine (75 mg, 0.145 mmol, 1.0 equiv) in CH_2Cl_2 (1.5 mL) and was reacted with trifluoroacetic acid (53 µL, 1.45 mmol, 10.0 equiv) for 12 h. The crude product was purified by preparative thin-layer chromatography (10% MeOH/CH_2Cl_2 + 1% NH_4OH) to yield the racemic isoquinuclidine (47 mg, 82% yield) as a white solid. ^{1}H NMR (500 MHz, CD_3OD) δ 7.66 (s, 1H), 7.53 (s, 1H), 7.10 (t, J = 7.5 Hz, 1H), 6.68 (dd, J = 8.7, 1.7 Hz, 1H), 6.65 – 6.54 (m, 2H), 6.03 (dd, J = 6.4, 1.3 Hz, 1H), 4.17 (q, J = 7.3 Hz, 2H), 3.82 – 3.76 (m, 2H), 3.65 (s, 3H), 3.60 (d, J = 13.2 Hz, 1H), 3.46 – 3.40 (m, 1H), 2.26 (q, J = 6.2 Hz, 1H), 1.96 (dd, J = 13.0, 4.5 Hz, 1H), 1.66 (dd, J = 13.0, 9.4 Hz, 1H), 1.44 (t, J = 7.3Hz, 3H), 1.01 (s, 3H), 0.90 (d, J = 6.2 Hz, 3H). ^{13}C NMR (126 MHz, CD_3OD) δ 174.9, 156.6, 149.4, 141.1, 139.5, 129.7, 128.5, 127.3, 119.2, 117.5, 114.8, 113.6, 64.4, 52.8, 51.0, 46.8, 46.4, 40.9, 36.9, 35.8, 20.4, 18.5, 14.7. HRMS (ESI+, m/z) calcd for $C_{23}H_{30}N_3O_3$ $(M+H)^+$: 396.2282, found: 396.2291.

HPLC Traces of #020

A portion of this material was separated using semi-preparative chiral HPLC (Chiralpak AD-H column, 250 x 10 mm, 12% *i*PrOH/Hexanes+1% DEA, 2.5 mL/min) to provide the two enantiomers with tr = 35 min and 42 min.

Chiralpak AD-H, analytical column (250 x 4.6 mm), 12% *i*PrOH/Hexanes+0.1% DEA, 1 mL/min, 280 nm

Racemic

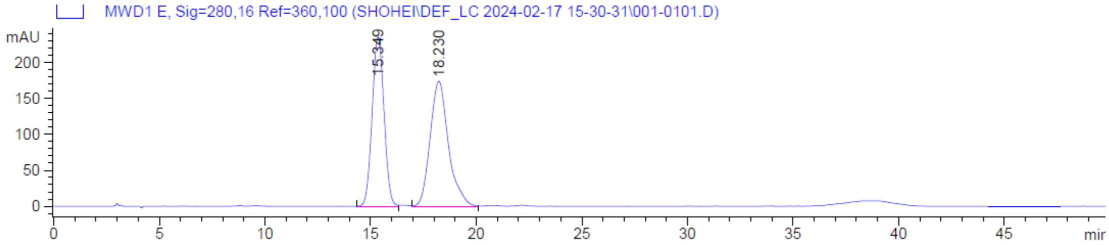

Enantiomer 1

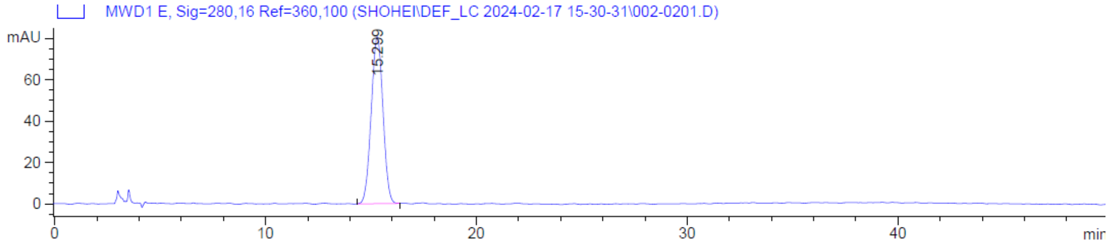

Enantiomer 2

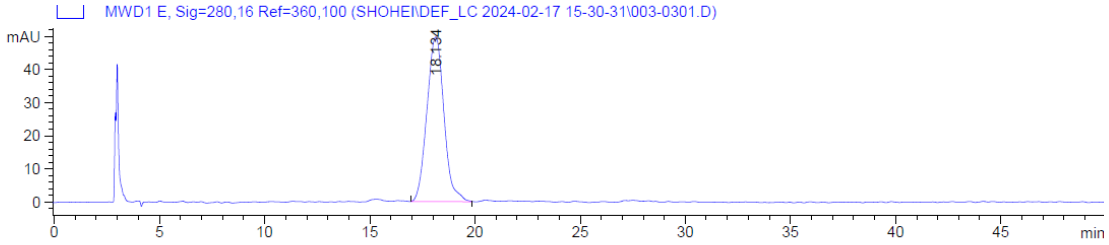

Synthesis of #021

・**Synthesis of Imine**

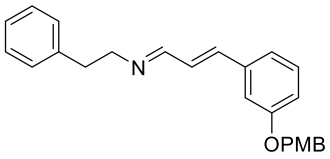

General procedure A was followed using 2-phenylethanamine (50 mg, 0.410 mmol, 1.1 equiv), (*E*)-3-[3-(4-Methoxy-benzyloxy)-phenyl]-propenal (100 mg, 0.373 mmol, 1.0 equiv), molecular sieves 4 Å, and CH_2Cl_2 (0.75 mL). Imine (138 mg, 99% yield) was obtained as a pale-yellow oil. ^{1}H NMR (400 MHz, $CDCl_3$) δ 7.88 (dd, *J* = 6.7, 1.5 Hz, 1H), 7.34 (d, *J* = 8.7 Hz, 2H), 7.31 – 7.25 (m, 3H), 7.22 – 7.16 (m, 3H), 7.07 – 7.02 (m, 2H), 6.95 – 6.88 (m, 3H), 6.88 – 6.83 (m, 2H), 4.98 (s, 2H), 3.80 (s, 3H), 3.75 (t, *J* = 7.5 Hz, 2H), 2.96 (t, *J* = 7.5 Hz, 2H).

・**Key Reaction**

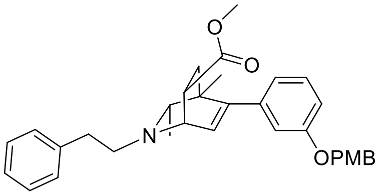

General procedure B was followed using above obtained imine (138 mg, 0.371 mmol, 1.0 equiv) and 2-butyne (146 µL, 1.86 mmol, 5 equiv) in THF (0.37 mL). Rh catalyst (10 mol %, 0.37 mL, 0.037 mmol, 100 mM in THF) was added, and the reaction was carried out at 65 °C for 12 h to produce the DHP intermediate. Following general procedure C, methyl acrylate (0.34 mL, 3.71 mmol, 10 equiv) was added to the solution of DHP intermediate, and the reaction was carried out at 50 °C for 16 h. The resulting crude material was purified by column chromatography (50% EtOAc/Hexane+1% Et_3N) to afford the desired product (70 mg, 37% yield from imine) as a pale-yellow oil. ^{1}H NMR (400 MHz, CD_3OD) δ 7.31 (d, *J* = 8.6 Hz, 2H), 7.29 – 7.12 (m, 6H), 6.98 – 6.80 (m, 3H), 6.79 – 6.62 (m, 2H), 6.09 (d, *J* = 6.3 Hz, 1H), 4.97 (s, 2H), 3.88 (dd, *J* = 6.4, 3.1 Hz, 1H), 3.75 (s, 3H), 3.62 (s, 3H), 3.31 –

3.25 (m, 1H), 2.94 – 2.72 (m, 4H), 2.19 (q, J = 6.3 Hz, 1H), 1.91 (dd, J = 13.1, 4.3 Hz, 1H), 1.56 (dd, J = 13.1, 9.4 Hz, 1H), 0.97 (d, J = 6.3 Hz, 3H), 0.93 (s, 3H).

・**Deprotection**

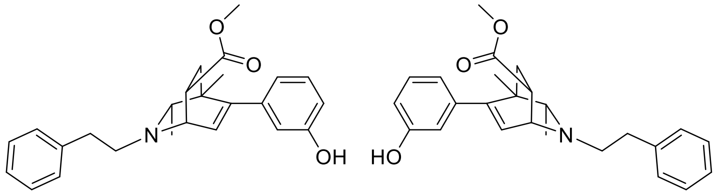

#021
enantiomers separated by chiral HPLC

Final Compound #021 General Procedure D was followed using protected isoquinuclidine (70 mg, 0.137 mmol, 1.0 equiv) in CH_2Cl_2 (1.4 mL) and was reacted with trifluoroacetic acid (102 µL, 1.37 mmol, 10.0 equiv) for 12 h. The crude product was purified by preparative thin-layer chromatography (10% MeOH/CH_2Cl_2 + 1% NH_4OH) to yield the racemic isoquinuclidine (50 mg, 93% yield) as a pale-yellow oil. ^1H NMR (400 MHz, CD_3OD) δ 7.31 – 7.19 (m, 4H), 7.21 – 7.13 (m, 1H), 7.10 (t, J = 8.2 Hz, 1H), 6.68 (ddd, J = 8.2, 2.4, 1.1 Hz, 1H), 6.64 – 6.56 (m, 2H), 6.09 (d, J = 6.4 Hz, 1H), 3.92 (dd, J = 6.4, 3.1 Hz, 1H), 3.63 (s, 3H), 3.33 – 3.27 (m, 1H), 2.94 – 2.73 (m, 4H), 2.25 (q, J = 6.3 Hz, 1H), 1.93 (dd, J = 13.1, 4.3 Hz, 1H), 1.60 (dd, J = 13.1, 9.4 Hz, 1H), 1.015 (d, J = 6.3 Hz, 3H), 1.01 (s 3H). ^{13}C NMR (101 MHz, CD_3OD) δ 174.7, 156.7, 149.3, 141.0, 139.7, 128.5, 128.4, 128.1, 126.9, 125.9, 119.1, 114.8, 113.7, 64.9, 56.8, 54.3, 51.0, 40.8, 37.4, 35.3, 35.2, 20.5, 19.1. HRMS (ESI+, m/z) calcd for $C_{25}H_{30}NO_3$ $(M+H)^+$: 392.2220, found: 392.2230.

HPLC Traces of #021

A portion of this material was separated using semi-preparative chiral HPLC (Chiralpak IC column, 250 x 10 mm, 15% *i*PrOH/Hexanes+1% DEA, 2.5 mL/min) to provide the two enantiomers with tr = 24 min and 47 min.

Chiralpak IC, analytical column (250 x 4.6 mm), 15% *i*PrOH/Hexanes+0.1% DEA, 1 mL/min, 210 nm

Racemic

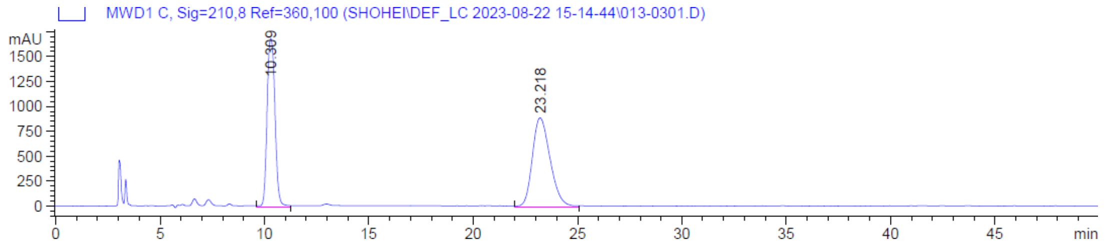

Enantiomer 1

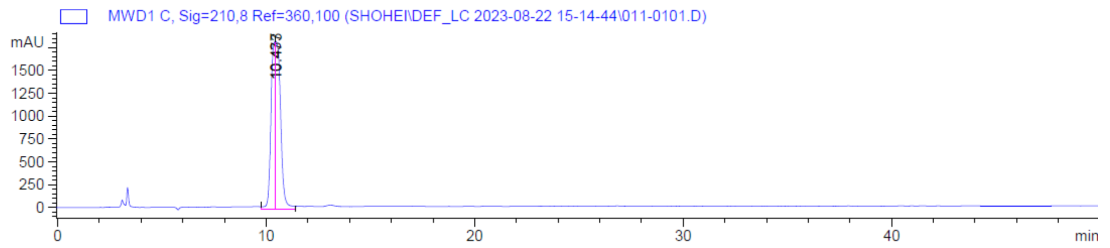

Enantiomer 2

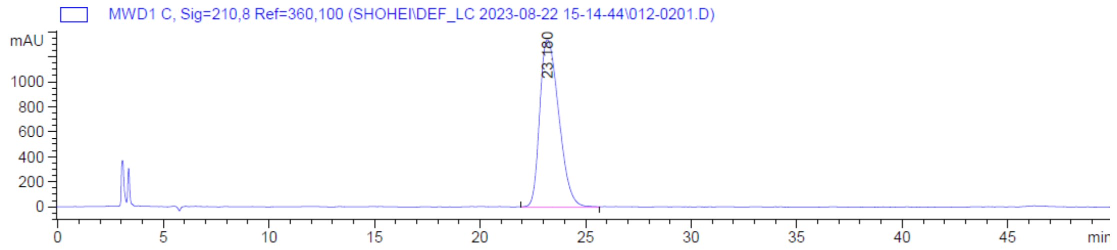

Synthesis of #022

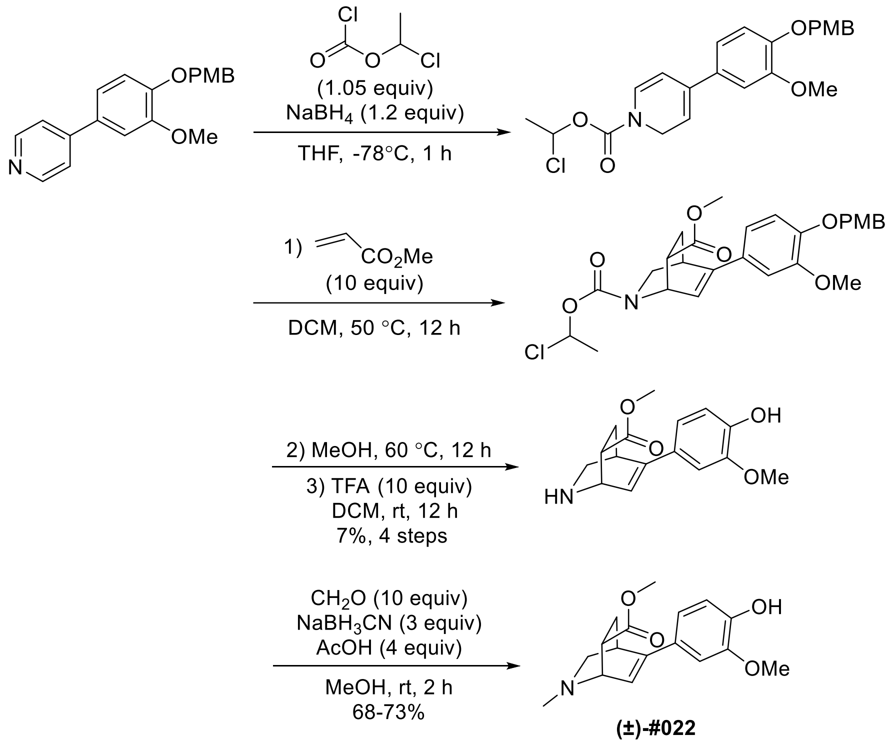

To a flame-dried round-bottom flask was added a magnetic stir bar, 4-[3-methoxy-4-[(4-methoxyphenyl)methoxy]phenyl]pyridine[7] (1620 mg, 5.04 mmol, 1 equiv) and $NaBH_4$ (248 mg, 6.355 mmol, 1.3 equiv) dissolved in dry THF (10 mL). This solution was cooled to -78 °C and then 1-chloroethyl chloroformate (793 mg, 5.55 mmol, 1.1 equiv) was added dropwise over 5 min. The reaction mixture was stirred at -78 ° C for 1 h. The mixture was then diluted with water, extracted with Et_2O, washed with brine, dried with Na_2SO_4 and filtered. The organic layer was concentrated and the crude residue was purified via silica gel chromatography (gradient of 0-50% EtOAc in hexane) to give

477 mg of the *N*-protected dihydropyridine in 22% yield. To a stirred solution of the above obtained compound (477 mg, 1.11 mmol, 1 equiv) in CH_2Cl_2 (0.1 mL) was added methyl acrylate (1.0 mL, 11.1 mmol, 10 equiv) and the reaction mixture was heated at 50 ° C for 12 h. The mixture was concentrated and the crude residue was purified via silica gel chromatography (gradient of 0-50% EtOAc in hexane) to give 170 mg of the *N*-protected isoquinuclidine in 31% yield. The solution of the above obtained compound (170 mg, 0.34 mmol, 1 equiv) in MeOH (34 mL) was heated at 60 °C for 12 h. The mixture was concentrated and diluted with CH_2Cl_2 (3.4 mL). Trifluoroacetic acid (253 µL, 3.40 mmol, 10.0 equiv) was added to this solution and stirred at room temperature for 12h. The mixture was concentrated and the crude residue was purified by preparative thin-layer chromatography (10% MeOH/CH_2Cl_2 + 1% NH_4OH) to yield the separate diastereomers of isoquinuclidines (93 mg, 95% combined yield, 2:1 ratio) as a dark-brown oil.

・**Major diastereomer**

^{1}H NMR (400 MHz, CD_3OD) δ 7.03 (d, *J* = 2.0 Hz, 1H), 6.92 (dd, *J* = 8.2, 2.0 Hz, 1H), 6.75 (d, *J* = 8.2 Hz, 1H), 6.59 (dd, *J* = 6.3, 2.0 Hz, 1H), 3.96 (dd, *J* = 6.3, 2.5 Hz, 1H), 3.85 (s, 3H), 3.73 (s, 3H), 3.25 – 3.17 (m, 1H), 3.01 (dd, *J* = 10.2, 2.0 Hz, 1H), 2.62 – 2.48 (m, 2H), 2.14 (ddd, *J* = 13.0, 4.6, 2.5 Hz, 1H), 1.73 – 1.60 (m, 1H).

・**Minor diastereomer**

^{1}H NMR (400 MHz, CD_3OD) δ 6.99 (d, *J* = 2.1 Hz, 1H), 6.91 (dd, *J* = 8.2, 2.1 Hz, 1H), 6.74 (d, *J* = 8.2 Hz, 1H), 6.42 (dd, *J* = 6.0, 2.0 Hz, 1H), 4.05 (dd, *J* = 6.0, 2.9 Hz, 1H), 3.84 (s, 3H), 3.62 (s, 3H), 3.28 – 3.24 (m, 1H), 3.09 (ddd, *J* = 9.8, 5.2, 2.9 Hz, 1H), 2.93 (dd, *J* = 10.4, 2.0 Hz, 1H), 2.51 (dt, *J* = 10.4, 2.8 Hz, 1H), 1.99 (ddd, *J* = 12.7, 9.8, 2.8 Hz, 1H), 1.87 – 1.75 (m, 1H).

Final Compound #022

・**Major diastereomer**

To a stirred solution of the above obtained major diastereomer (60 mg, 0.207 mmol, 1 equiv) in MeOH (2 mL) was added AcOH (48 µL, 0.830 mmol, 4 equiv), formaldehyde (0.19 mL of 37% w/w solution, 6.22 mmol, 10 equiv), $NaBH_3CN$ (39 mg, 0.622 mmol, 3 equiv) and the reaction mixture was stirred at room temperature for 12 h. After the reaction was complete as monitored by thin-layer chromatography, the mixture was then diluted with water, extracted with CH_2Cl_2, washed with brine, dried with Na_2SO_4 and filtered. The organic layer was concentrated and the crude residue was purified by preparative thin-layer chromatography (5% MeOH/CH_2Cl_2 + 1% NH_4OH) to yield the racemic isoquinuclidine (43 mg, 68% yield) as a pale-yellow oil. ^{1}H NMR (500 MHz, CD_3OD) δ 7.04 (d, *J* = 2.1 Hz, 1H), 6.93 (dd, *J* = 8.3, 2.1 Hz, 1H), 6.77 (d, *J* = 8.3 Hz, 1H), 6.50 (dd, *J* = 5.8, 1.9 Hz, 1H), 3.87 (s, 3H), 3.84 (dd, *J* = 5.8, 2.6 Hz, 1H), 3.73 (s, 3H), 3.18 (dd, *J* = 9.9, 2.3 Hz, 1H), 3.15 – 3.10

(m, 1H), 2.54 (ddd, *J* = 11.4, 4.6, 2.6 Hz, 1H), 2.25 – 2.13 (m, 4H), 1.92 (dt, *J* = 9.9, 2.6 Hz, 1H), 1.57 – 1.47 (m, 1H). ^{13}C NMR (126 MHz, CD_3OD) δ 174.7, 147.8, 146.4, 145.8, 128.9, 121.2, 117.8, 114.8, 108.2, 57.4, 55.8, 55.0, 51.1, 44.9, 44.2, 33.3, 23.7. HRMS (ESI+, m/z) calcd for $C_{17}H_{22}NO_4$ $(M+H)^+$: 304.1543, found: 304.1529.

・**Minor diastereomer**

To a stirred solution of the above obtained minor diastereomer (33 mg, 0.114 mmol, 1 equiv) in MeOH (1.2 mL) was added AcOH (26 µL, 0.456 mmol, 4 equiv), formaldehyde (0.1 mL of 37% w/w solution, 3.42 mmol, 10 equiv), $NaBH_3CN$ (22 mg, 0.342 mmol, 3 equiv) and the reaction mixture was stirred at room temperature for 12 h. After the reaction was complete as monitored by thin-layer chromatography, the mixture was then diluted with water, extracted with CH_2Cl_2, washed with brine, dried with Na_2SO_4 and filtered. The organic layer was concentrated and the crude residue was purified by preparative thin-layer chromatography (5% MeOH/CH_2Cl_2 + 1% NH_4OH) to yield the racemic isoquinuclidine (25 mg, 72% yield) as a pale-yellow oil. ^{1}H NMR (500 MHz, CD_3OD) δ 7.01 (d, *J* = 2.1 Hz, 1H), 6.93 (dd, *J* = 8.2, 2.1 Hz, 1H), 6.77 (d, *J* = 8.2 Hz, 1H), 6.37 (dd, *J* = 5.7, 1.9 Hz, 1H), 3.92 – 3.83 (m, 4H), 3.64 (s, 3H), 3.24 – 3.15 (m, 2H), 3.04 (dd, *J* = 10.4, 2.2 Hz, 1H), 2.33 (s, 3H), 2.10 (dt, *J* = 10.4, 2.7 Hz, 1H), 1.90 (ddd, *J* = 12.6, 9.8, 2.7 Hz, 1H), 1.86 – 1.75 (m, 1H). ^{13}C NMR (126 MHz, CD_3OD) δ 173.9, 147.8, 146.6, 146.2, 128.7, 119.6, 117.9, 114.8, 108.3, 56.3, 55.0, 54.6, 51.0, 42.9, 42.6, 33.1, 25.4. HRMS (ESI+, m/z) calcd for $C_{17}H_{22}NO_4$ $(M+H)^+$: 304.1543, found: 304.1529.

Synthesis of #023

・**Synthesis of Imine**

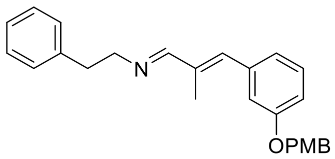

General procedure A was followed using 2-phenylethanamine (71 mg, 0.584 mmol, 1.1 equiv), above obtained aldehyde (150 mg, 0.531 mmol, 1.0 equiv), molecular sieves 4 Å, and CH_2Cl_2 (1.0 mL) at 50 °C. Imine (200 mg, 98% yield) was obtained as a pale-yellow oil. ^{1}H NMR (400 MHz, $CDCl_3$) δ 7.84 (s, 1H), 7.35 (d, *J* = 8.6 Hz, 2H), 7.28 (m, 3H), 7.23 – 7.14 (m, 3H), 7.01 – 6.94 (m, 2H), 6.94 – 6.87 (m, 3H), 6.67 (s, 1H), 4.99 (s, 2H), 3.80 (s, 3H), 3.80 – 3.74 (m, 2H), 2.95 (t, *J* = 7.6 Hz, 2H), 2.11 (s, 3H).

・**Key Reaction**

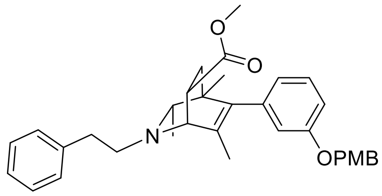

General procedure B was followed using above obtained imine (200 mg, 0.519 mmol, 1.0 equiv) and 2-butyne (203 µL, 2.59 mmol, 5 equiv) in THF (0.52 mL). Rh catalyst (10 mol %, 0.52 mL, 0.052 mmol, 100 mM in THF) was added, and the reaction was carried out at 65 °C for 12 h to produce the DHP intermediate. Following general procedure C, methyl acrylate (0.23 mL, 2.59 mmol, 5 equiv) was added to the solution of DHP intermediate, and the reaction was carried out at 50 °C for 16 h. The resulting crude material was purified by column chromatography (50% EtOAc/Hexane+1% Et_3N) to afford the desired product (185 mg, 68% yield from imine) as a pale-yellow oil. ^{1}H NMR (400 MHz, CD_3OD) δ 7.35 – 7.16 (m, 8H), 6.91 – 6.84 (m, 3H), 6.60 (m, 2H), 4.99 (s, 2H), 3.80 – 3.74 (m, 4H), 3.69 – 3.61 (m, 4H), 2.93 – 2.71 (m, 4H), 2.18 (q, *J* = 6.3 Hz, 1H), 1.94 (dd, *J* = 13.0, 4.2 Hz, 1H), 1.58 – 1.46 (m, 4H), 0.98 (d, *J* = 6.2 Hz, 3H), 0.76 (s, 3H).

・**Deprotection**

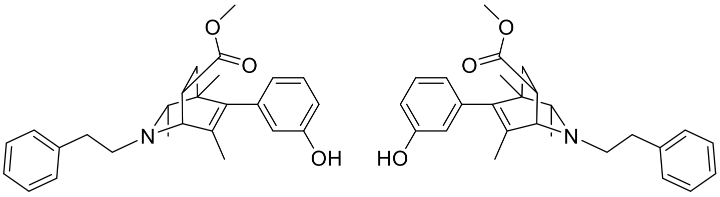

#023
enantiomers separated by chiral HPLC

Final Compound #023 General Procedure D was followed using protected isoquinuclidine (100 mg, 0.190 mmol, 1.0 equiv) in CH_2Cl_2 (1.9 mL) and was reacted with trifluoroacetic acid (141 µL, 1.90 mmol, 10.0 equiv) for 12 h. The crude product was purified by preparative thin-layer chromatography (10% MeOH/CH_2Cl_2 + 1% NH_4OH) to yield the racemic isoquinuclidine (73 mg, 95% yield) as a white solid. ^{1}H NMR (500 MHz, CD_3OD, 50 °C) δ 7.27 (d, *J* = 7.8 Hz, 1H), 7.26 – 7.22 (m, 3H), 7.20 – 7.14 (m, 1H), 7.12 (t, *J* = 7.8 Hz, 1H), 6.68 (dd, *J* = 7.7, 1.9 Hz, 1H), 6.52 (d, *J* = 8.2 Hz, 2H), 3.69 – 3.63 (m, 4H), 3.30 – 3.26 (m, 1H), 2.93 – 2.76 (m, 4H), 2.17 (q, *J* = 6.3 Hz, 1H), 1.96 (dd, *J* = 13.0, 4.3 Hz, 1H), 1.58 (s, 3H), 1.53 (dd, *J* = 13.0, 9.4 Hz, 1H), 1.00 (d, *J* = 6.3 Hz, 3H), 0.83 (s, 3H). ^{13}C NMR (126 MHz, CD_3OD, 50 °C) δ 174.8, 156.7, 140.3, 140.1, 139.9, 134.0, 128.5, 128.4, 128.0, 125.7, 120.1, 115.7, 113.2, 65.5, 59.8, 56.6, 50.8, 40.8, 37.4, 36.1, 35.5, 20.6, 19.3, 16.5. HRMS (ESI+, m/z) calcd for $C_{26}H_{32}NO_3$ $(M+H)^+$: 406.2377, found: 406.2390.

HPLC Traces of #023

A portion of this material was separated using semi-preparative chiral HPLC (Chiralpak IC column, 250 x 10 mm, 4% *i*PrOH/Hexanes+1% DEA, 2.5 mL/min) to provide the two enantiomers with tr = 15 min and 25 min.

Chiralpak IC, analytical column (250 x 4.6 mm), 4% *i*PrOH/Hexanes+0.1% DEA, 1 mL/min, 210 nm

Racemic

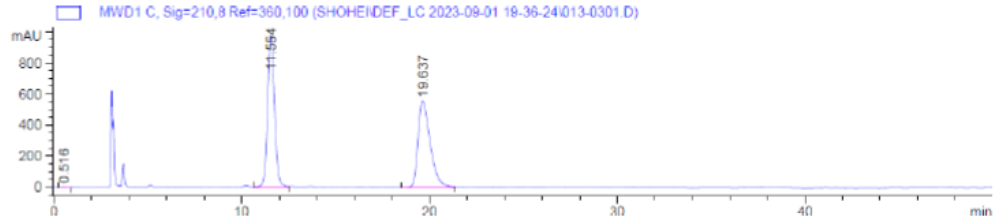

Enantiomer 1

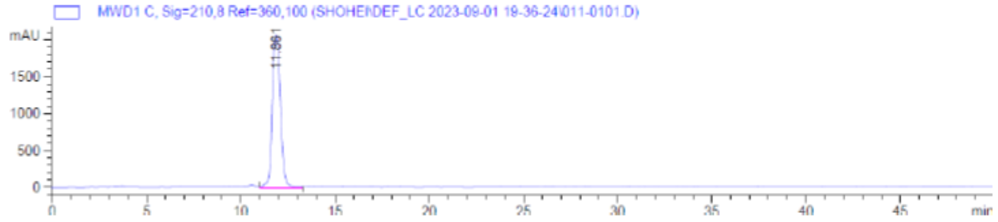

Enantiomer 2

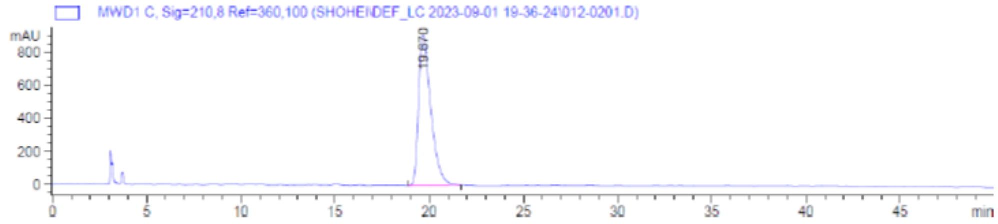

Synthesis of #024

・**Key Reaction**

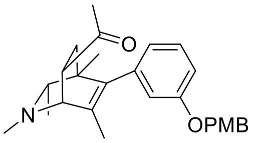

General procedure B was followed using above obtained imine (250 mg, 0.846 mmol, 1.0 equiv) and 2-butyne (332 µL, 4.23 mmol, 5 equiv) in THF (0.85 mL). Rh catalyst (10 mol %, 0.85 mL, 0.085 mmol, 100 mM in THF) was added, and the reaction was carried out at 65 °C for 12 h to produce the DHP intermediate. Following general procedure C, methyl vinyl ketone (106 µL, 1.27 mmol, 1.5 equiv) was added to the solution of DHP intermediate, and the reaction was carried out at room

temperature for 12 h. The resulting crude material was purified by column chromatography (50% EtOAc/Hexane+1% Et_3N) to afford the desired product (101 mg, 28% yield from imine) as a pale-yellow oil. ^1H NMR (400 MHz, CD_3OD) δ 7.32 (d, *J* = 7.9 Hz, 2H), 7.19 (t, *J* = 8.1 Hz, 1H), 6.95 – 6.80 (m, 3H), 6.59 (d, *J* = 7.4 Hz, 2H), 4.97 (s, 2H), 3.75 (s, 3H), 3.57 – 3.42 (m, 2H), 2.49 (s, 3H), 2.18 (s, 3H), 2.00 (q, *J* = 6.2 Hz, 1H), 1.90 (dd, *J* = 12.6, 5.1 Hz, 1H), 1.51 (s, 3H), 1.38 (dd, *J* = 12.6, 9.1 Hz, 1H), 0.94 (d, *J* = 6.3 Hz, 3H), 0.72 (s, 3H).

・Deprotection

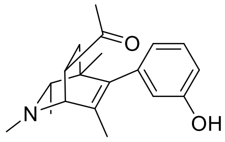

(±)-#024

Final Compound #024 General Procedure D was followed using protected isoquinuclidine (101 mg, 0.241 mmol, 1.0 equiv) in CH_2Cl_2 (2.4 mL) and was reacted with trifluoroacetic acid (179 µL, 2.41 mmol, 10.0 equiv) for 12 h. The crude product was purified by preparative thin-layer chromatography (10% MeOH/CH_2Cl_2 + 1% NH_4OH) to yield the racemic isoquinuclidine (57 mg, 79% yield) as a white solid. ^1H NMR (400 MHz, CD_3OD) δ 7.10 (t, *J* = 7.9 Hz, 1H), 6.65 (dd, *J* = 8.2, 1.2 Hz, 1H), 6.49 (m, 2H), 3.57 – 3.41 (m, 2H), 2.50 (s, 3H), 2.18 (s, 3H), 2.03 (q, *J* = 6.3 Hz, 1H), 1.91 (dd, *J* = 12.6, 5.1 Hz, 1H), 1.55 (s, 3H), 1.41 (dd, *J* = 12.6, 8.9 Hz, 1H), 0.98 (d, *J* = 6.3 Hz, 3H), 0.79 (s, 3H). ^{13}C NMR (126 MHz, CD_3OD, 50 °C) δ 209.2, 156.7, 139.7, 139.7, 134.4, 128.5, 120.1, 115.7, 113.2, 65.7, 61.8, 45.4, 40.8, 39.6, 35.9, 27.3, 20.4, 17.5, 16.7. HRMS (ESI+, m/z) calcd for $C_{19}H_{26}NO_2$ $(M+H)^+$: 300.1958, found: 316.1968.

Synthesis of #025

・Key Reaction

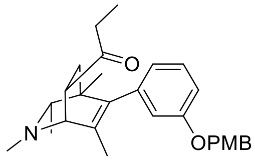

General procedure B was followed using above obtained imine (250 mg, 0.846 mmol, 1.0 equiv) and 2-butyne (332 µL, 4.23 mmol, 5 equiv) in THF (0.85 mL). Rh catalyst (10 mol %, 0.85 mL, 0.085 mmol, 100 mM in THF) was added, and the reaction was carried out at 65 °C for 12 h to produce the DHP intermediate. Following general procedure C, ethyl vinyl ketone (126 µL, 1.27 mmol, 1.5 equiv) was added to the solution of DHP intermediate, and the reaction was carried out at room temperature for 12 h. The resulting crude material was purified by column chromatography (50%

EtOAc/Hexane+1% Et_3N) to afford the desired product (78 mg, 21% yield from imine) as a pale-yellow oil. ^1H NMR (400 MHz, CD_3OD) δ 7.33 (d, J = 6.8 Hz, 2H), 7.20 (t, J = 7.9 Hz, 1H), 6.99 – 6.78 (m, 3H), 6.61 (d, J = 7.5 Hz, 2H), 4.99 (s, 2H), 3.76 (s, 3H), 3.57 – 3.44 (m, 1H), 3.41 (d, J = 2.6 Hz, 1H), 2.70 – 2.41 (m, 5H), 2.01 (q, J = 6.3 Hz, 1H), 1.91 (dd, J = 12.5, 5.2 Hz, 1H), 1.48 (s, 3H), 1.38 (dd, J = 12.5, 9.1 Hz, 1H), 1.02 (t, J = 7.3 Hz, 3H), 0.94 (d, J = 6.3 Hz, 3H), 0.72 (s, 3H).

・Deprotection

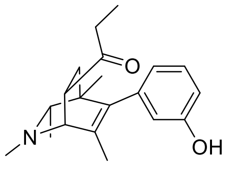

(±)-#025

Final Compound #025 General Procedure D was followed using protected isoquinuclidine (78 mg, 0.180 mmol, 1.0 equiv) in CH_2Cl_2 (2.4 mL) and was reacted with trifluoroacetic acid (134 µL, 1.80 mmol, 10.0 equiv) for 12 h. The crude product was purified by preparative thin-layer chromatography (10% MeOH/CH_2Cl_2 + 1% NH_4OH) to yield the racemic isoquinuclidine (47 mg, 83% yield) as a white solid. ^1H NMR (400 MHz, CD_3OD) δ 7.10 (t, J = 8.0 Hz, 1H), 6.65 (ddd, J = 8.1, 2.4, 1.1 Hz, 1H), 6.58 – 6.44 (m, 2H), 3.50 (ddd, J = 8.9, 5.3, 2.7 Hz, 1H), 3.41 (d, J = 2.7 Hz, 1H), 2.67 – 2.41 (m, 5H), 2.02 (q, J = 6.3 Hz, 1H), 1.92 (dd, J = 12.5, 5.3 Hz, 1H), 1.52 (s, 3H), 1.40 (dd, J = 12.5, 8.9 Hz, 1H), 1.02 (t, J = 7.3 Hz, 3H), 0.97 (d, J = 6.3 Hz, 3H), 0.79 (s, 3H). ^{13}C NMR (126 MHz, CD_3OD, 50 ℃) δ 211.8, 156.7, 139.7, 139.5, 134.4, 128.5, 120.1, 115.7, 113.2, 65.6, 62.1, 44.3, 40.8, 39.5, 36.1, 33.9, 20.4, 17.5, 16.6, 6.8. HRMS (ESI+, m/z) calcd for $C_{20}H_{28}NO_2$ $(M+H)^+$: 314.2115, found: 314.2124.

Synthesis of #026

・Hydrogenation

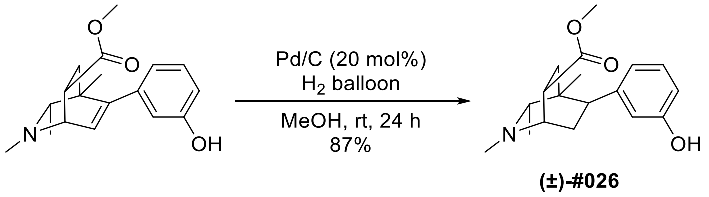

(±)-#026

Final Compound #026 To a flame-dried round-bottom flask was added a magnetic stir bar, above obtained isoquinuclidine (35 mg, 0.083 mmol, 1 equiv) and 10 wt. % Pd/C (17.6 mg, 0.016 mmol, 20 mol%) dissolved in MeOH (5 mL). The headspace was purged with H_2 balloon and the reaction mixture was stirred at room temperature for 24 h. The reaction mixture was filtered through a pad of

celite and washed with MeOH. The filtrate was concentrated and the crude residue was purified by preparative thin-layer chromatography (5% MeOH/CH_2Cl_2 + 1% NH_4OH) to yield the desired isoquinuclidine (22 mg, 87% yield) as a white solid. ^{1}H NMR (500 MHz, $CDCl_3$) δ 7.15 (t, *J* = 7.8 Hz, 1H), 6.94 (s, 1H), 6.88 (d, *J* = 7.6 Hz, 1H), 6.73 (dd, *J* = 8.2, 2.4 Hz, 1H), 3.78 (s, 3H), 3.15 – 3.05 (m, 1H), 3.02 – 2.89 (m, 2H), 2.52 – 2.38 (m, 4H), 2.21 – 2.08 (m, 2H), 1.78 (dd, *J* = 13.9, 8.7 Hz, 1H), 1.20 – 1.09 (m, 4H), 0.39 (s, 3H). ^{13}C NMR (126 MHz, $CDCl_3$) δ 175.9, 155.9, 143.9, 129.1, 121.8, 116.5, 113.6, 66.1, 56.7, 52.1, 40.7, 39.4, 36.3, 35.2, 31.4, 30.8, 22.3, 13.5. HRMS (ESI+, m/z) calcd for $C_{18}H_{26}NO_3$ $(M+H)^+$: 304.1907, found: 304.1917.

Synthesis of #027

・Synthesis of Aldehyde[8]

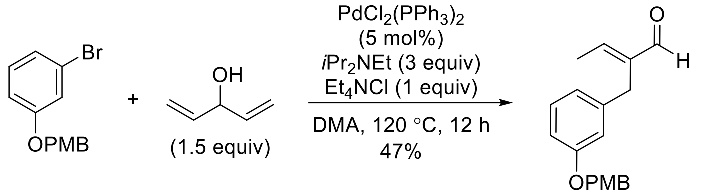

To a round-bottom flask was added a magnetic stir bar, 1-bromo-3-(4-methoxyphenyl)methoxy benzene (1.0 g, 3.41 mmol, 1 equiv) and 1,4-pentadien-3-ol (430 mg, 5.12 mmol, 1.5 equiv) dissolved in DMA (17 mL). To this solution was then added *N*,*N*-diisopropylethylamine (1.78 mL, 10.2 mmol, 3 equiv), dichlorobis(triphenylphosphine)palladium (120 mg, 0.171 mmol, 5 mol%) and tetraethylammonium chloride (565 mg, 3.41 mmol, 1 equiv). The reaction mixture was stirred at 120 °C for 12 h. After the reaction was complete, the mixture was then diluted with water, extracted with CH_2Cl_2, washed with brine, dried with Na_2SO_4 and filtered. The organic layer was concentrated and the crude residue was purified via silica gel chromatography (gradient of 0-100% EtOAc in hexane) to give 0.48 g of desired aldehyde in 47% yield. ^{1}H NMR (400 MHz, $CDCl_3$) δ 9.43 (s, 1H), 7.35 – 7.30 (m, 2H), 7.17 – 7.12 (m, 1H), 6.91 – 6.87 (m, 2H), 6.79 – 6.74 (m, 3H), 6.70 (q, *J* = 7.2 Hz, 1H), 4.92 (s, 2H), 3.80 (s, 3H), 3.59 (s, 2H), 2.00 (d, *J* = 7.2 Hz, 3H).

・Synthesis of Imine

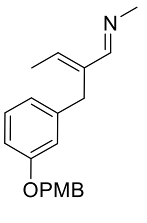

General procedure A was followed using 2.0 M methylamine in THF (1.27 mL, 2.53 mmol, 5.0 equiv),

above obtained aldehyde (150 mg, 0.506 mmol, 1.0 equiv), molecular sieves 4 Å, and CH_2Cl_2 (1.0 mL) at 50 °C. Imine (150 mg, 96% yield) was obtained as a yellow solid. ^{1}H NMR (400 MHz, $CDCl_3$) δ 7.82 (s, 1H), 7.35 – 7.30 (m, 2H), 7.13 (t, *J* = 7.9 Hz, 1H), 6.91 – 6.86 (m, 2H), 6.83 – 6.71 (m, 3H), 6.06 (q, *J* = 7.0 Hz, 1H), 4.92 (s, 2H), 3.80 (s, 3H), 3.72 (s, 2H), 3.33 (s, 2H), 1.82 (d, *J* = 7.0 Hz, 3H).

・**Key Reaction**

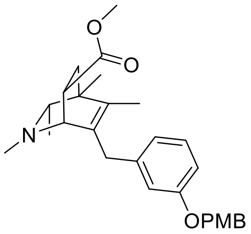

General procedure B was followed using above obtained imine (150 mg, 0.485 mmol, 1.0 equiv) and 2-butyne (190 µL, 2.42 mmol, 5 equiv) in THF (0.49 mL). Rh catalyst (10 mol %, 0.49 mL, 0.049 mmol, 100 mM in THF) was added, and the reaction was carried out at 65 °C for 12 h to produce the DHP intermediate. Following general procedure C, methyl acrylate (0.22 mL, 2.42 mmol, 5 equiv) was added to the solution of DHP intermediate, and the reaction was carried out at 50 °C for 16 h. The resulting crude material was purified by column chromatography (50% EtOAc/Hexane+1% Et_3N) to afford the desired product (61 mg, 28% yield from imine) as a pale-yellow oil. ^{1}H NMR (400 MHz, $CDCl_3$) δ 7.35 (d, *J* = 8.5 Hz, 2H), 7.15 (t, *J* = 7.9 Hz, 1H), 6.99 – 6.83 (m, 3H), 6.83 – 6.70 (m, 2H), 4.97 (d, *J* = 11.1 Hz, 1H), 4.93 (d, *J* = 11.1 Hz, 1H), 3.80 (s, 3H), 3.56 (d, *J* = 15.9 Hz, 1H), 3.40 (d, *J* = 15.9 Hz, 1H), 3.31 (s, 3H), 3.07 (ddd, *J* = 9.4, 5.5, 2.8 Hz, 1H), 2.36 (s, 3H), 1.88 (q, *J* = 6.2 Hz, 1H), 1.80 (dd, *J* = 12.8, 5.5 Hz, 1H), 1.39 (dd, *J* = 12.8, 9.4 Hz, 1H), 1.09 (s, 3H), 0.84 (d, *J* = 6.2 Hz, 3H). HRMS (ESI+, m/z) calcd for $C_{28}H_{36}NO_4$ $(M+H)^+$: 450.2639, found: 450.2642.

・**Deprotection**

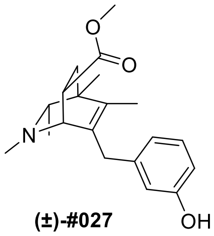

(±)-#027

Final Compound #027 General Procedure D was followed using protected isoquinuclidine (45 mg, 0.100 mmol, 1.0 equiv) in CH_2Cl_2 (1.0 mL) and was reacted with trifluoroacetic acid (74 µL, 1.00 mmol, 10.0 equiv) for 12 h. The crude product was purified by preparative thin-layer chromatography (10% MeOH/CH_2Cl_2 + 1% NH_4OH) to yield the racemic isoquinuclidine (14 mg, 40% yield) as a pale-yellow oil. ^{1}H NMR (400 MHz, CD_3OD) δ 7.01 (t, *J* = 7.8 Hz, 1H), 6.66 – 6.42 (m, 3H), 3.54 (d, *J* = 15.3 Hz, 1H), 3.36 – 3.32 (m, 1H), 3.26 (d, *J* = 15.3 Hz, 1H), 3.13 (s, 3H), 3.06 (ddd, *J* = 9.3, 5.8,

2.7 Hz, 1H), 2.35 (s, 3H), 1.92 (q, *J* = 6.3 Hz, 1H), 1.80 (s, 3H), 1.77 (dd, *J* = 12.9, 5.8 Hz, 1H), 1.38 (dd, *J* = 12.8, 9.3 Hz, 1H), 1.11 (s, 3H), 0.83 (d, *J* = 6.3 Hz, 3H). ^{13}C NMR (101 MHz, CD_3OD) δ 174.3, 157.3, 140.8, 135.4, 135.0, 128.8, 119.7, 115.7, 112.6, 65.0, 61.1, 50.6, 40.1, 39.2, 36.3, 35.9, 35.9, 19.3, 16.8, 12.9. HRMS (ESI+, m/z) calcd for $C_{20}H_{27}NO_3$ $(M+H)^+$: 330.2064, found: 330.2057.

Synthesis of #028

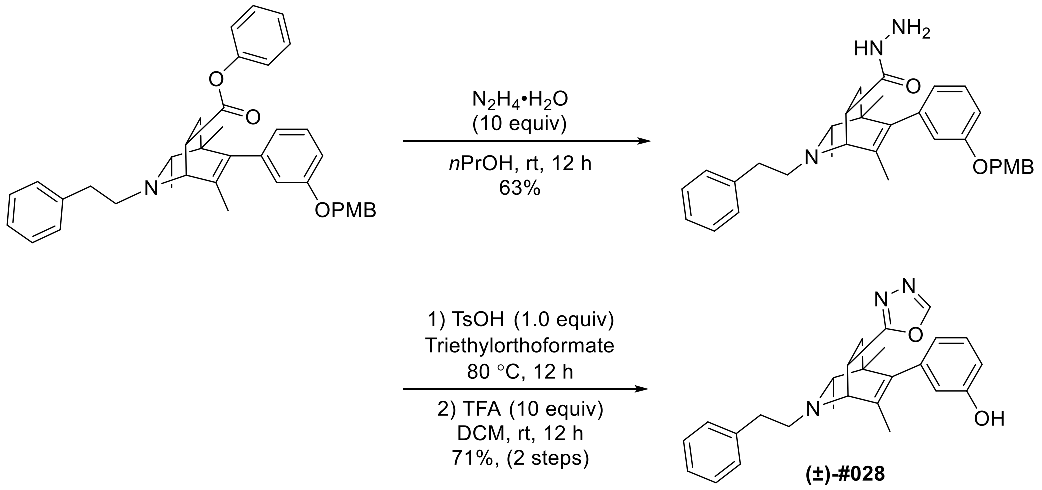

To a round-bottom flask was added a magnetic stir bar, above obtained isoquinuclidine (210 mg, 0.357 mmol, 1 equiv) dissolved in *n*PrOH (1.8 mL). To this solution was then added 65% hydrazine monohydrate (275 mg, 3.57 mmol, 10 equiv) at 0 °C. The reaction mixture was stirred at room temperature for 12 h. After the reaction was complete, the mixture was then diluted with water, extracted with CH_2Cl_2, dried with Na_2SO_4 and filtered. The organic layer was concentrated and the crude residue was purified by preparative thin-layer chromatography (10% $MeOH/CH_2Cl_2$ + 1% NH_4OH) to give 118 mg of amide compound in 63% yield. ^{1}H NMR (400 MHz, CD_3OD) δ 7.34 (d, *J* = 8.2 Hz, 2H), 7.30 – 7.24 (m, 4H), 7.24 – 7.14 (m, 2H), 6.89 (d, *J* = 8.5 Hz, 2H), 6.85 (dd, *J* = 8.3, 2.4 Hz, 1H), 6.80 – 6.57 (m, 2H), 4.99 (s, 2H), 3.76 (s, 3H), 3.45 (d, *J* = 2.9 Hz, 1H), 3.16 – 3.12 (m, 1H), 2.98 – 2.72 (m, 4H), 2.20 (q, *J* = 6.3 Hz, 1H), 1.93 (dd, *J* = 12.7, 4.3 Hz, 1H), 1.57 (s, 3H), 1.47 (dd, *J* = 12.7, 9.3 Hz, 1H), 1.01 (d, *J* = 6.3 Hz, 3H), 0.77 (s, 3H). HRMS (ESI+, m/z) calcd for $C_{33}H_{40}N_3O_3$ $(M+H)^+$: 526.3064, found: 526.3074.

Final Compound #028 To a stirred solution of the above obtained compound (60 mg, 0.114 mmol, 1 equiv) in triethylorthoformate (4.6 mL) was added *p*-toluenesulfonic acid monohydrate (21.7 mg, 0.114 mmol, 1 equiv). The reaction mixture was heated at 80 °C for 12 h. After the reaction was complete, the mixture was concentrated and dissolved in CH_2Cl_2 (1.2 mL). This solution was added trifluoroacetic acid (85 μL, 1.14 mmol, 10.0 equiv) and stirred at room temperature for 12 h. The crude product was purified by preparative thin-layer chromatography (10% $MeOH/CH_2Cl_2$ + 1% NH_4OH)

to yield the racemic isoquinuclidine (34 mg, 71% yield) as a pale yellow oil. ^{1}H NMR (400 MHz, CD_3OD) δ 8.83 (s, 1H), 7.27 (d, *J* = 4.4 Hz, 4H), 7.22 – 7.06 (m, 2H), 6.66 (dd, *J* = 8.0, 2.5 Hz, 1H), 6.53 (d, *J* = 5.3 Hz, 2H), 4.03 – 3.90 (m, 1H), 3.68 (d, *J* = 3.0 Hz, 1H), 3.04 – 2.76 (m, 4H), 2.34 (q, *J* = 6.3 Hz, 1H), 2.12 (dd, *J* = 13.1, 4.5 Hz, 1H), 1.93 (dd, *J* = 13.1, 9.5 Hz, 1H), 1.37 (s, 3H), 1.04 (d, *J* = 6.2 Hz, 3H), 0.88 (s, 3H). ^{13}C NMR (126 MHz, CD_3OD, 50 °C) δ 168.9, 156.8, 153.9, 140.6, 139.9, 139.6, 134.0, 128.6, 128.4, 128.0, 125.8, 115.7, 113.3, 65.2, 60.4, 56.2, 40.8, 37.5, 35.6, 29.7, 20.5, 19.3, 16.3. HRMS (ESI+, m/z) calcd for $C_{26}H_{29}N_3O_2$ $(M+H)^+$: 416.2333, found: 416.2335.

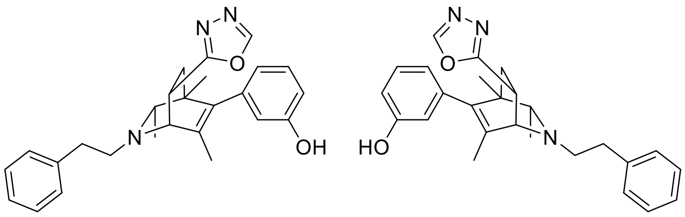

#028
enantiomers separated by chiral HPLC

HPLC Traces of #028

A portion of this material was separated using semi-preparative chiral HPLC (Chiralpak IC column, 250 x 10 mm, 10% *i*PrOH/Hexanes+1% DEA, 2.5 mL/min) to provide the two enantiomers with tr = 26 min and 39 min.

Chiralpak IC, analytical column (250 x 4.6 mm), 10% *i*PrOH/Hexanes+0.1% DEA, 1 mL/min, 210 nm

Racemic

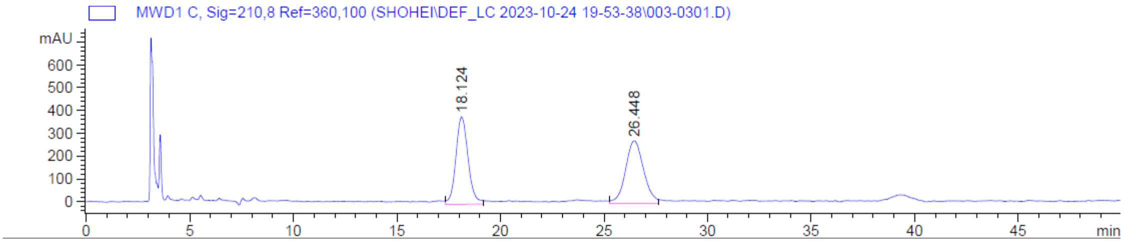

Enantiomer 1

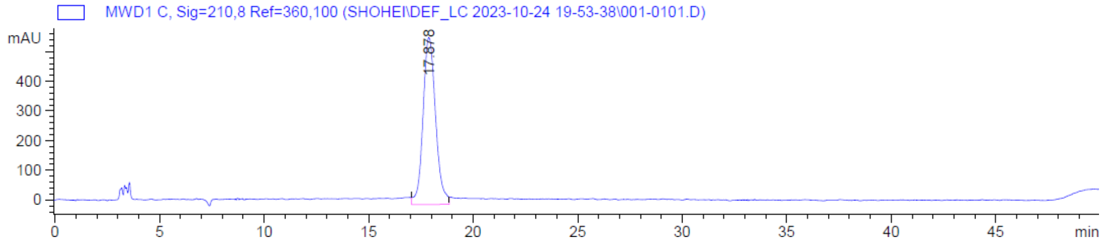

Enantiomer 2

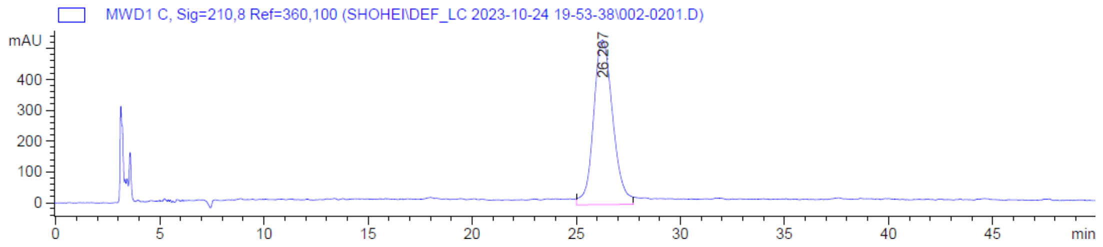

Synthesis of #029

・**Key Reaction**

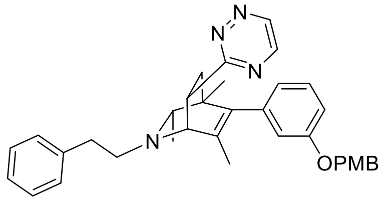

General procedure B was followed using above obtained imine (200 mg, 0.519 mmol, 1.0 equiv) and 2-butyne (203 µL, 2.59 mmol, 5 equiv) in THF (0.52 mL). Rh catalyst (10 mol %, 0.52 mL, 0.052 mmol, 100 mM in THF) was added, and the reaction was carried out at 65 °C for 12 h to produce the DHP intermediate. Following general procedure C, 3-ethenyl-1,2,4-triazine (278 mg, 2.59 mmol, 5 equiv) was added to the solution of DHP intermediate, and the reaction was carried out at 50 °C for 16 h. The resulting crude material was purified by column chromatography (50% EtOAc/Hexane+1% Et_3N) to afford the desired product (95 mg, 34% yield from imine) as a pale-yellow oil. ^1H NMR (400 MHz, CD_3OD) δ 9.11 (d, J = 2.4 Hz, 1H), 8.69 (d, J = 2.4 Hz, 1H), 7.36 – 7.15 (m, 8H), 6.91 – 6.82 (m, 3H), 6.81 – 6.58 (m, 2H), 5.00 (s, 2H), 4.15 (dt, J = 9.3, 3.8 Hz, 1H), 3.81 – 3.71 (m, 4H), 3.07 – 2.79 (m, 4H), 2.57 (dd, J = 12.9, 4.4 Hz, 1H), 2.35 (q, J = 6.3 Hz, 1H), 1.76 (dd, J = 12.9, 9.3 Hz, 1H), 1.09 (s, 3H), 1.04 (d, J = 6.3 Hz, 3H), 0.84 (s, 3H). LRMS (ESI+, m/z) calcd for $C_{35}H_{39}N_4O_2$ $(M+H)^+$: 547.3, found: 547.2.

・**Deprotection**

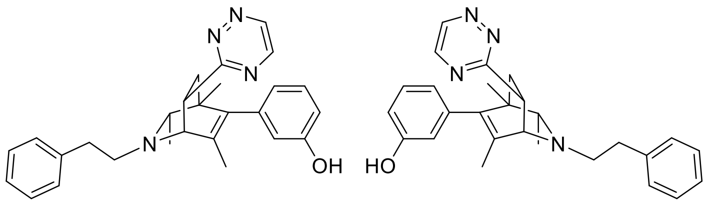

#029
enantiomers separated by chiral HPLC

Final Compound #029 General Procedure D was followed using protected isoquinuclidine (95 mg, 0.174 mmol, 1.0 equiv) in CH_2Cl_2 (1.7 mL) and was reacted with trifluoroacetic acid (129 µL, 1.74

mmol, 10.0 equiv) for 12 h. The crude product was purified by preparative thin-layer chromatography (10% MeOH/CH_2Cl_2 + 1% NH_4OH) to yield the racemic isoquinuclidine (35 mg, 47% yield) as a pale-yellow oil. ^1H NMR (400 MHz, CD_3OD) δ 9.11 (d, J = 2.5 Hz, 1H), 8.72 (d, J = 2.5 Hz, 1H), 7.34 – 7.23 (m, 4H), 7.21 – 7.14 (m, 1H), 7.11 (t, J = 7.7 Hz, 1H), 6.65 (dd, J = 8.1, 2.4 Hz, 1H), 6.62 – 6.51 (m, 2H), 4.16 (dt, J = 8.3, 3.8 Hz, 1H), 3.80 (d, J = 3.1 Hz, 1H), 3.08 – 2.80 (m, 4H), 2.57 (dd, J = 13.0, 4.4 Hz, 1H), 2.39 (q, J = 6.2 Hz, 1H), 1.78 (dd, J = 13.0, 9.5 Hz, 1H), 1.13 (s, 3H), 1.08 (d, J = 6.2 Hz, 3H), 0.91 (s, 3H). ^{13}C NMR (126 MHz, CD_3OD, 50 °C) δ 170.8, 156.6, 149.3, 147.2, 140.8, 140.0, 139.9, 133.2, 128.5, 128.4, 128.0, 125.8, 120.1, 115.8, 113.1, 65.8, 62.6, 56.6, 41.2, 39.4, 36.3, 35.4, 20.7, 19.1, 16.6. HRMS (ESI+, m/z) calcd for $C_{27}H_{31}N_4O$ $(M+H)^+$: 427.2492, found: 427.2522.

HPLC Traces of #029

A portion of this material was separated using semi-preparative chiral HPLC (Chiralpak AD-H column, 250 x 10 mm, 7% *i*PrOH/Hexanes+1% DEA, 2.5 mL/min) to provide the two enantiomers with tr = 44 min and 58 min.

Chiralpak AD-H, analytical column (250 x 4.6 mm), 7% *i*PrOH/Hexanes+0.1% DEA, 1 mL/min, 210 nm

Racemic

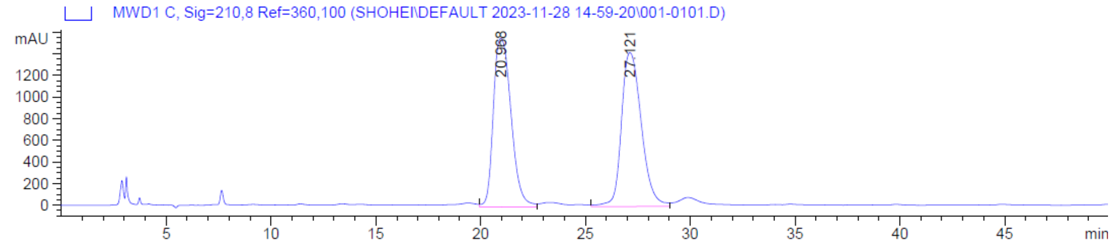

Enantiomer 1

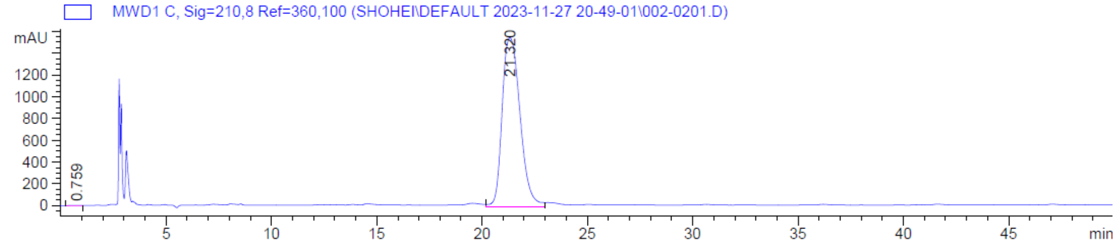

Enantiomer 2

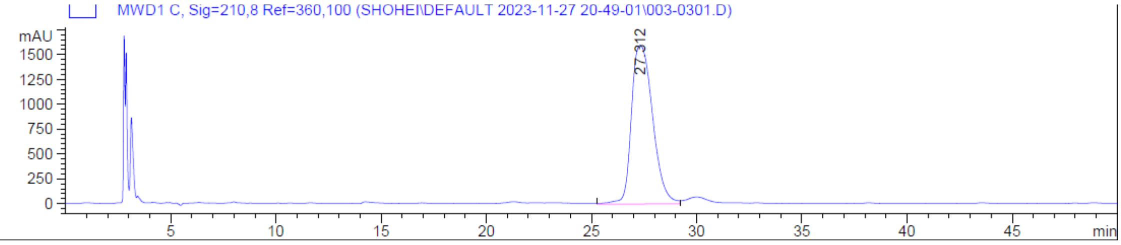

Synthesis of #030

・**Key Reaction**

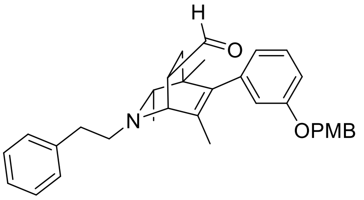

General procedure B was followed using above obtained imine (250 mg, 0.649 mmol, 1.0 equiv) and 2-butyne (254 µL, 3.24 mmol, 5 equiv) in THF (0.65 mL). Rh catalyst (10 mol %, 0.65 mL, 0.065 mmol, 100 mM in THF) was added, and the reaction was carried out at 65 °C for 12 h to produce the DHP intermediate. Following general procedure C, acrolein (217 µL, 3.24 mmol, 5 equiv) was added to the solution of DHP intermediate, and the reaction was carried out at room temperature for 12 h. The resulting crude material was purified by column chromatography (40% EtOAc/Hexane+1% Et_3N) to afford the desired product (90 mg, 28% yield from imine) as a pale-yellow oil. ^1H NMR (400 MHz, $CDCl_3$) δ 9.53 (s, 1H), 7.43 – 7.17 (m, 9H), 6.90 (d, J = 8.6 Hz, 2H), 6.86 (dd, J = 8.2, 2.6 Hz, 1H), 6.75 – 6.51 (m, 2H), 4.97 (s, 2H), 3.80 (s, 3H), 3.69 (d, J = 3.1 Hz, 1H), 3.22 (dt, J = 8.6, 3.8 Hz, 1H), 2.98 – 2.77 (m, 4H), 2.15 (q, J = 6.3 Hz, 1H), 1.97 (dd, J = 13.0, 4.3 Hz, 1H), 1.61 (s, 3H), 1.49 (dd, J = 13.0, 9.2 Hz, 1H), 0.99 (d, J = 6.3 Hz, 3H), 0.84 (s, 3H). LRMS (ESI+, m/z) calcd for $C_{33}H_{38}NO_3$ $(M+H)^+$: 496.3, found: 496.6.

・**Decarbonylation and deprotection**

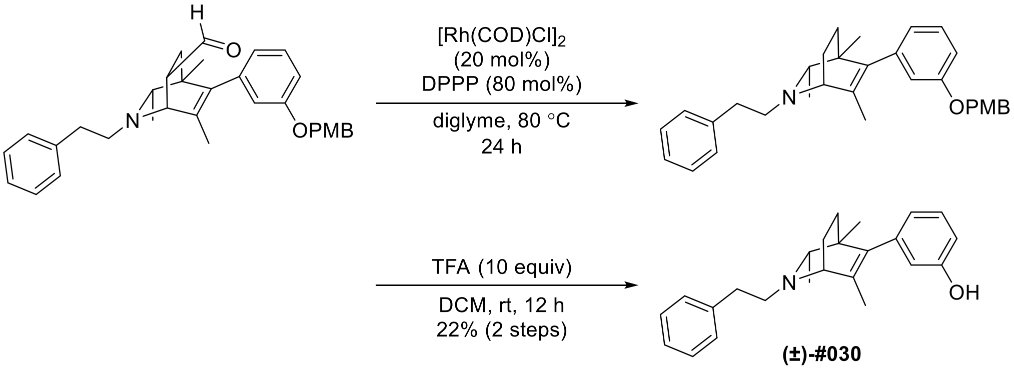

Final Compound #030 To a sealable glass vessel was added a magnetic stir bar, above obtained isoquinuclidine (150 mg, 0.303 mmol, 1 equiv) dissolved in diglyme (10 mL). To this solution was then added chloro(1,5-cyclooctadiene)rhodium(I) dimer (20 mol%, 30 mg, 0.061 mmol) and 1,3-bis(diphenylphosphino) propane (80 mol%, 100 mg, 0.244 mmol) at room temperature. The reaction mixture was stirred at 80 °C for 24 h. After the reaction was complete, the mixture was then diluted

with water, extracted with CH_2Cl_2, dried with Na_2SO_4 and filtered. The organic layer was concentrated and the crude residue was purified by preparative thin-layer chromatography (35% EtOAc/Hexane+1% Et_3N) to give 45 mg of compound in 32% yield. The above obtained compound was dissolved in CH_2Cl_2 (1.0 mL). To this solution was added trifluoroacetic acid (65 μL, 0.877 mmol, 10.0 equiv) and the resulting solution was stirred at room temperature for 12 h. The crude product was purified by preparative thin-layer chromatography (10% MeOH/CH_2Cl_2 + 1% NH_4OH) to yield the racemic isoquinuclidine (21 mg, 69% yield) as a pale-yellow oil. ^{1}H NMR (500 MHz, CD_3OD) δ 7.31 – 7.22 (m, 4H), 7.19 (t, J = 7.1 Hz, 1H), 7.14 (t, J = 7.9 Hz, 1H), 6.68 (dd, J = 8.1, 2.4 Hz, 1H), 6.63 – 6.31 (broad s, 2H), 3.42 – 3.36 (m, 1H), 2.99 – 2.70 (m, 4H), 2.36 (q, J = 6.3 Hz, 1H), 2.29 – 2.18 (m, 1H), 1.73 (s, 3H), 1.54 – 1.29 (m, 3H), 1.08 (d, J = 6.3 Hz, 3H), 0.83 (s, 3H). ^{13}C NMR (126 MHz, CD_3OD) δ 156.8, 139.9, 139.8, 139.6, 136.2, 128.6, 128.3, 128.1, 125.9, 120.0, 115.6, 113.1, 66.8, 57.8, 57.2, 40.5, 34.8, 33.7, 20.9, 19.21, 19.17, 15.6. HRMS (ESI+, m/z) calcd for $C_{24}H_{30}NO$ $(M+H)^+$: 348.2322, found: 348.2313.

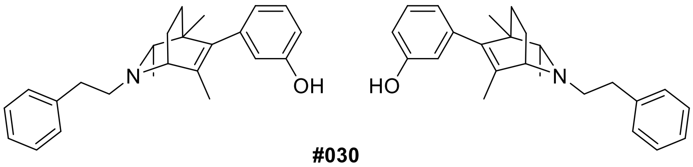

#030
enantiomers separated by chiral HPLC

HPLC Traces of #030

A portion of this material was separated using semi-preparative chiral HPLC (Chiralpak AD-H column, 250 x 10 mm, 4% *i*PrOH/Hexanes+1% DEA, 2.5 mL/min) to provide the two enantiomers with tr = 26 min and 32 min.

Chiralpak AD-H, analytical column (250 x 4.6 mm), 4% *i*PrOH/Hexanes+0.1% DEA, 1 mL/min, 280 nm

Racemic

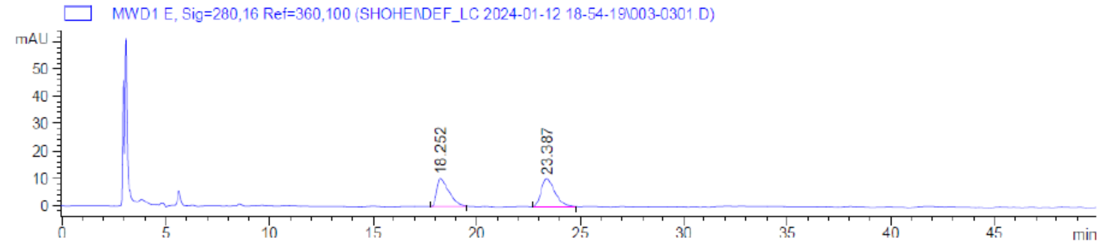

Enantiomer 1

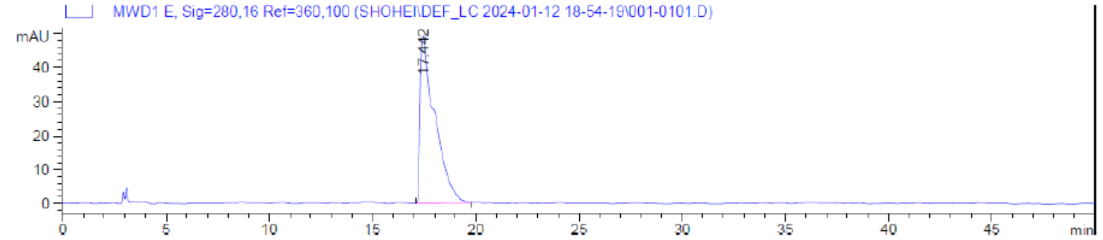

Enantiomer 2

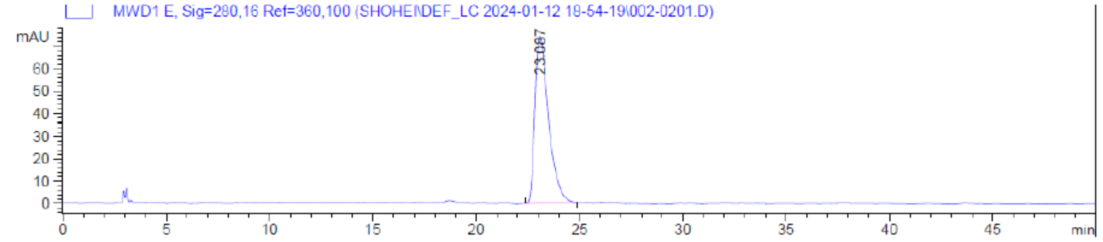

Synthesis of #031

・Synthesis of Imine

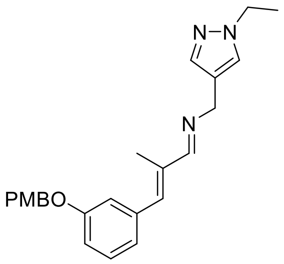

General procedure A was followed using (1-ethyl-1H-pyrazol-4-yl)methanamine (122 mg, 0.974 mmol, 1.1 equiv), above obtained aldehyde (250 mg, 0.885 mmol, 1.0 equiv), molecular sieves 4 Å, and CH_2Cl_2 (1.9 mL) at 50 °C. Imine (340 mg, 99% yield) was obtained as a pale-yellow oil. ^{1}H NMR (400 MHz, $CDCl_3$) δ 8.01 (s, 1H), 7.46 – 7.22 (m, 6H), 7.01 – 6.75 (m, 5H), 4.99 (d, *J* = 2.5 Hz, 2H), 4.62 (s, 2H), 4.13 (q, *J* = 7.5 Hz, 2H), 3.80 (s, 3H), 2.13 (s, 3H), 1.46 (t, *J* = 7.5 Hz, 3H).

・Key Reaction

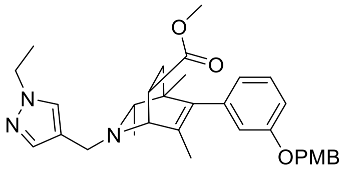

General procedure B was followed using above obtained imine (340 mg, 0.873 mmol, 1.0 equiv) and 2-butyne (342 μL, 4.36 mmol, 5 equiv) in THF (0.87 mL). Rh catalyst (10 mol %, 0.87 mL, 0.087 mmol, 100 mM in THF) was added, and the reaction was carried out at 65 °C for 12 h to produce the DHP intermediate. Following general procedure C, methyl acrylate (0.39 mL, 4.36 mmol, 5 equiv) was added to the solution of DHP intermediate, and the reaction was carried out at 50 °C for 16 h. The resulting crude material was purified by column chromatography (50% EtOAc/Hexane+1% Et_3N) to afford the desired product (280 mg, 61% yield from imine) as a pale-yellow oil. ^{1}H NMR (400 MHz, CD_3OD) δ 7.63 (d, *J* = 2.5 Hz, 1H), 7.51 (d, *J* = 2.5 Hz, 1H), 7.31 (d, *J* = 8.1 Hz, 2H), 7.19 (td, *J* = 8.1, 2.4 Hz, 1H), 6.92 – 6.74 (m, 3H), 6.69 – 6.50 (m, 2H), 4.97 (s, 2H), 4.15 (qd, *J* = 7.3, 2.4 Hz, 2H), 3.80 – 3.66 (m, 4H), 3.64 (s, 3H), 3.60 – 3.49 (m, 2H), 3.42 – 3.35 (m, 1H), 2.18 (q, *J* = 3.9 Hz, 1H), 1.95 (dd, *J* = 12.8, 4.4 Hz, 1H), 1.55 (dd, *J* = 12.8, 9.2 Hz, 1H), 1.49 – 1.34 (m, 6H), 0.84 (d, *J* = 3.9 Hz, 3H), 0.74 (s, 3H). LRMS (ESI+, m/z) calcd for $C_{33}H_{40}N_3O_6$ $(M+H)^+$: 574.3, found: 574.9.

・**Deprotection**

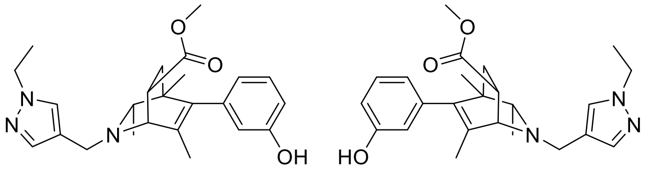

#031
enantiomers separated by chiral HPLC

Final Compound #031 General Procedure D was followed using protected isoquinuclidine (150 mg, 0.283 mmol, 1.0 equiv) in CH_2Cl_2 (2.8 mL) and was reacted with trifluoroacetic acid (210 μL, 2.83 mmol, 10.0 equiv) for 12 h. The crude product was purified by preparative thin-layer chromatography (10% MeOH/CH_2Cl_2 + 1% NH_4OH) to yield the racemic isoquinuclidine (70 mg, 60% yield) as a white solid. ^{1}H NMR (500 MHz, CD_3OD) δ 7.62 (s, 1H), 7.51 (s, 1H), 7.11 (t, *J* = 7.9 Hz, 1H), 6.67 (dd, *J* = 8.2, 3.1 Hz, 1H), 6.58 – 6.46 (m, 2H), 4.16 (q, *J* = 7.4 Hz, 2H), 3.76 (d, *J* = 13.4 Hz, 1H), 3.65 (s, 3H), 3.60 (d, *J* = 13.4 Hz, 1H), 3.58 (d, *J* = 3.0 Hz, 1H), 3.42 – 3.36 (m, 1H), 2.22 (q, *J* = 6.2 Hz, 1H), 1.97 (dd, *J* = 12.9, 4.6 Hz, 1H), 1.58 (dd, *J* = 12.9, 9.4 Hz, 1H), 1.50 (s, 3H), 1.44 (t, *J* = 7.4 Hz, 3H), 0.90 (d, *J* = 6.2 Hz, 3H), 0.83 (s, 3H). ^{13}C NMR (126 MHz, CD_3OD) δ 174.8, 156.7, 140.5, 139.8, 139.4, 134.2, 129.3, 128.5, 120.0, 118.2, 115.6, 113.2, 64.8, 58.6, 50.8, 46.4, 40.8, 36.9, 36.4, 20.5, 18.7, 16.4, 14.5. HRMS (ESI+, m/z) calcd for $C_{24}H_{32}N_3O_3$ $(M+H)^+$: 410.2438, found: 410.2421.

HPLC Traces of #031

A portion of this material was separated using semi-preparative chiral HPLC (Chiralpak IC column, 250 x 10 mm, 12% *i*PrOH/Hexanes+1% DEA, 2.5 mL/min) to provide the two enantiomers with tr = 36 min and 46 min.

Chiralpak IC, analytical column (250 x 4.6 mm), 12% *i*PrOH/Hexanes+0.1% DEA, 1 mL/min, 280 nm

Racemic

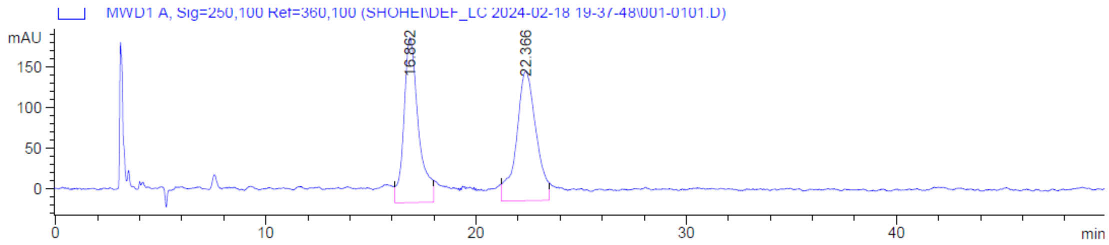

Enantiomer 1

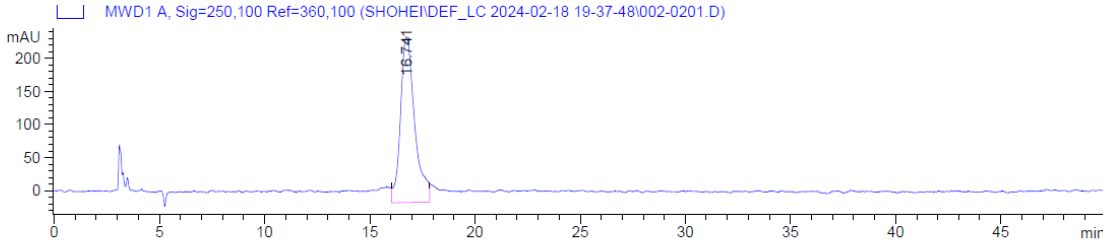

Enantiomer 2

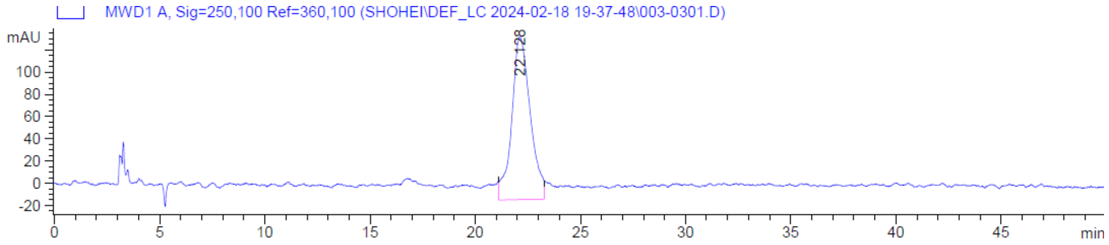

Synthesis of #036

・**Key Reaction**

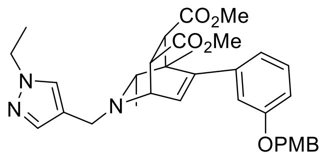

General procedure B was followed using above obtained imine (290 mg, 0.772 mmol, 1.0 equiv) and 2-butyne (303 µL, 3.86 mmol, 5 equiv) in THF (0.77 mL). Rh catalyst (10 mol %, 0.77 mL, 0.077 mmol, 100 mM in THF) was added, and the reaction was carried out at 65 °C for 12 h to produce the DHP intermediate. Following general procedure C, 1,4-dimethyl (2Z)-but-2-enedioate (167 mg, 1.16 mmol, 1.5 equiv) was added to the solution of DHP intermediate, and the reaction was carried out at 50 °C for 16 h. The resulting crude material was purified by column chromatography (70% EtOAc/Hexane+1% Et_3N) to afford the desired product (187 mg, 42% yield from imine) as a pale-yellow oil. ^{1}H NMR (400 MHz, $CDCl_3$) δ 7.52 (s, 1H), 7.46 – 7.37 (m, 1H), 7.33 (d, *J* = 8.6 Hz, 2H), 7.19 (t, *J* = 8.0 Hz, 1H), 6.91 – 6.84 (m, 3H), 6.76 (d, *J* = 8.1 Hz, 2H), 6.15 (d, *J* = 6.5 Hz, 1H), 4.95 (s, 2H), 4.13 (q, *J* = 7.4 Hz, 2H), 3.96 – 3.83 (m, 3H), 3.79 (s, 3H), 3.74 – 3.69 (m, 4H), 3.64 (s, 3H), 3.08 (d, *J* = 5.9 Hz, 1H), 2.82 (s, 1H), 1.46 (t, *J* = 7.3 Hz, 3H), 0.97 (s, 3H), 0.83 (d, *J* = 6.2 Hz, 3H). LRMS (ESI+, m/z) calcd for $C_{32}H_{40}N_3O_4$ $(M+H)^+$: 530.3, found: 530.3.

・**Deprotection**

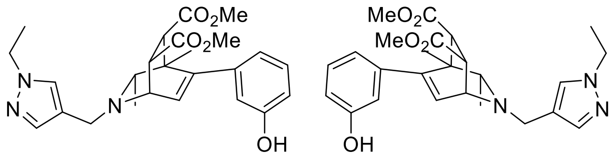

#036
enantiomers separated by chiral HPLC

Final Compound #036 General Procedure D was followed using protected isoquinuclidine (187 mg, 0.326 mmol, 1.0 equiv) in CH_2Cl_2 (3.3 mL) and was reacted with trifluoroacetic acid (242 µL, 3.26 mmol, 10.0 equiv) for 12 h. The crude product was purified by preparative thin-layer chromatography (10% MeOH/CH_2Cl_2 + 1% NH_4OH) to yield the racemic isoquinuclidine (73 mg, 50% yield) as a white solid. ^{1}H NMR (400 MHz, CD_3OD) δ 7.65 (s, 1H), 7.52 (s, 1H), 7.12 (t, *J* = 8.0 Hz, 1H), 6.75 – 6.64 (m, 1H), 6.64 – 6.53 (m, 2H), 6.11 (d, *J* = 6.4 Hz, 1H), 4.16 (q, *J* = 7.3 Hz, 2H), 3.98 (d, *J* = 13.3 Hz, 1H), 3.91 – 3.80 (m, 2H), 3.77 (dd, *J* = 5.9, 2.6 Hz, 1H), 3.73 (s, 3H), 3.66 (s, 3H), 3.05 (d, *J* = 5.9 Hz, 1H), 2.89 (q, *J* = 6.3 Hz, 1H), 1.43 (t, *J* = 7.3 Hz, 3H), 0.99 (s, 3H), 0.82 (d, *J* = 6.3 Hz, 3H). ^{13}C NMR (101 MHz, CD_3OD) δ 173.7, 173.4, 156.8, 148.9, 140.3, 139.6, 129.7, 128.7, 119.2, 117.6, 114.9, 113.9, 56.6, 52.5, 51.4, 50.9, 50.7, 46.4, 44.4, 43.2, 41.1, 18.8, 18.4, 14.7.
HRMS (ESI+, m/z) calcd for $C_{25}H_{31}N_3O_5$ $(M+H)^+$: 454.2336, found: 454.2315.

HPLC Traces of #036

A portion of this material was separated using semi-preparative chiral HPLC (Chiralpak IC column, 250 x 10 mm, 20% *i*PrOH/Hexanes+1% DEA, 2.5 mL/min) to provide the two enantiomers with tr = 16 min and 32 min.

Chiralpak IC, analytical column (250 x 4.6 mm), 20% *i*PrOH/Hexanes+0.1% DEA, 1 mL/min, 254 nm

Racemic

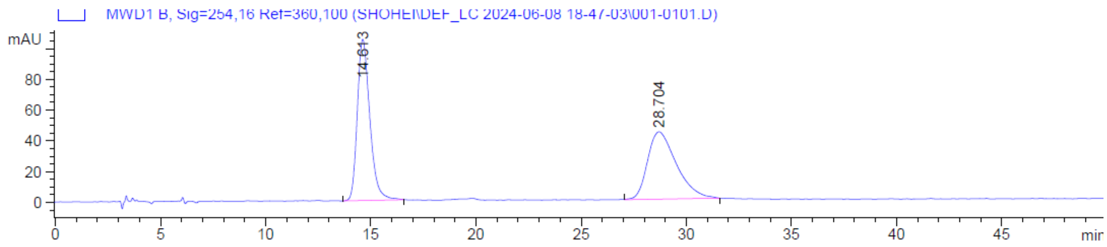

Enantiomer 1

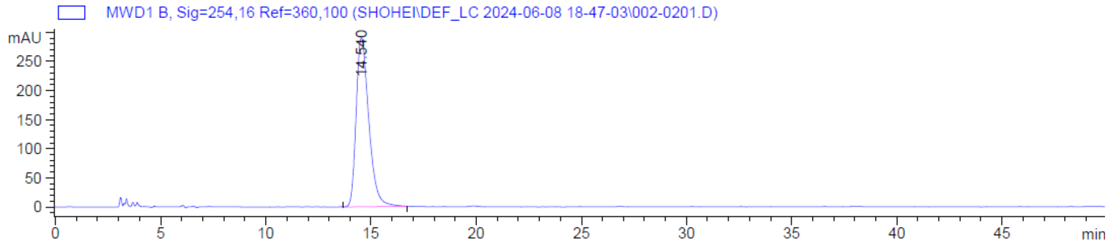

Enantiomer 2

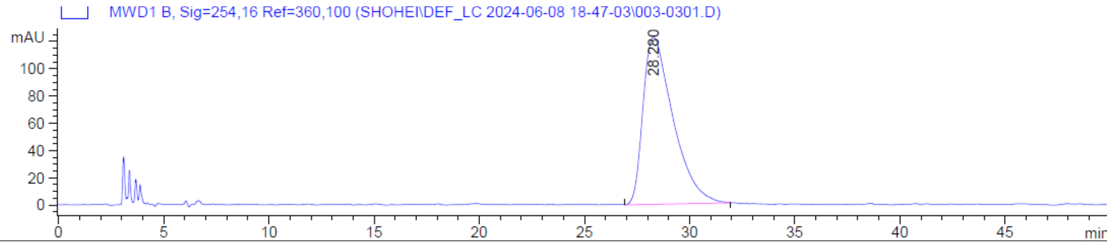

6. X-ray crystallography of Compound #020_E2

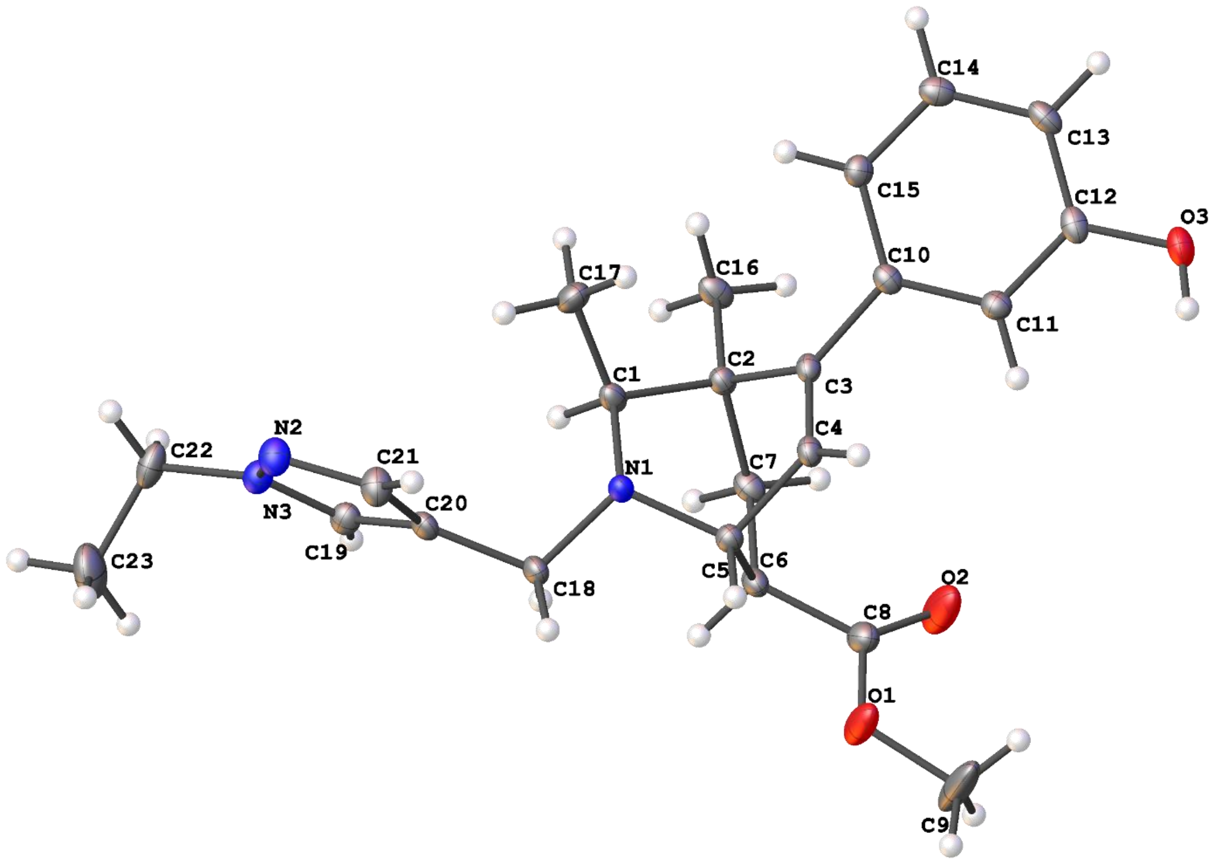

Figure S4. The complete numbering scheme of 007c-24016 with 50% thermal ellipsoid probability levels. The hydrogen atoms are shown as circles for clarity.

Table S4 Crystal data and structure refinement for 007c-24016.

Identification code	007c-24016
Empirical formula	$C_{29}H_{43}N_3O_3$
Formula weight	395.49
Temperature/K	99.96(18)
Crystal system	monoclinic
Space group	$P2_1$
a/Å	9.9513(3)
b/Å	10.1199(3)
c/Å	11.5512(3)
α/°	90
β/°	102.275(3)

γ/°	90
Volume/Å^3	1136.68(6)
Z	2
ρ_{calc}g/cm^3	1.156
μ/mm^{-1}	0.077
F(000)	424.0
Crystal size/mm^3	0.2 × 0.05 × 0.02
Radiation	Mo Kα (λ = 0.71073)
2Θ range for data collection/°	5.81 to 56.56
Index ranges	-13 ≤ h ≤ 13, -13 ≤ k ≤ 13, -15 ≤ l ≤ 15
Reflections collected	38297
Independent reflections	5639 [R_{int} = 0.0452, R_{sigma} = 0.0252]
Data/restraints/parameters	5639/1/267
Goodness-of-fit on F^2	1.034
Final R indexes [I>=2σ (I)]	R_1 = 0.0372, wR_2 = 0.0940
Final R indexes [all data]	R_1 = 0.0391, wR_2 = 0.0950
Largest diff. peak/hole / e Å^{-3}	0.27/-0.19
Flack parameter	0.0(3)

Table S5 Fractional Atomic Coordinates (×10^4) and Equivalent Isotropic Displacement Parameters (Å^2×10^3) for 007c-24016. U_{eq} is defined as 1/3 of the trace of the orthogonalised U_{IJ} tensor.

Atom	*x*	*y*	*z*	U(eq)
O1	7281(2)	1958.1(18)	4284.3(16)	38.5(5)
O2	7780(3)	1501(2)	2540.0(18)	47.5(6)
O3	12784.4(15)	3358.0(16)	1321.6(14)	22.7(3)
N1	6286.0(16)	5875.0(16)	3063.9(14)	13.5(3)
N2	3548.6(18)	9244.1(18)	3480.9(16)	20.3(4)
N3	2558.1(17)	8558.5(17)	2727.4(15)	17.4(3)
C1	6043.5(19)	6123(2)	1754.6(16)	15.0(4)

Table S5 Fractional Atomic Coordinates (×10[4]) and Equivalent Isotropic Displacement Parameters (Å^2×10^3) for 007c-24016. U_{eq} is defined as 1/3 of the trace of the orthogonalised U_{IJ} tensor.

Atom	*x*	*y*	*z*	U(eq)
C2	6799.6(18)	5054.1(19)	1161.9(16)	14.1(3)
C3	8314.8(18)	5042.6(18)	1817.4(16)	13.3(3)
C4	8454.4(18)	4844.6(18)	2985.5(17)	14.6(4)
C5	7130.2(19)	4665.8(19)	3402.8(17)	14.1(3)
C6	6397.3(19)	3427.7(19)	2746.1(17)	15.5(4)
C7	6197.7(19)	3691(2)	1411.9(17)	16.7(4)
C8	7224(2)	2191(2)	3139.1(19)	21.2(4)
C9	8005(5)	773(3)	4755(3)	61.9(11)
C10	9517.2(18)	5136(2)	1243.3(16)	14.2(3)
C11	10577.7(19)	4203.8(19)	1553.6(17)	15.0(3)
C12	11722.8(19)	4236(2)	1030.7(17)	16.3(4)
C13	11804.3(19)	5180(2)	167.7(17)	17.7(4)
C14	10770.1(19)	6126(2)	-120.0(17)	17.3(4)
C15	9638.8(19)	6118.1(19)	417.9(17)	15.6(4)
C16	6511.5(19)	5231(2)	-185.6(17)	18.4(4)
C17	6480(2)	7532(2)	1536.9(19)	21.7(4)
C18	4995.5(19)	5830.4(19)	3513.9(17)	15.7(4)
C19	2916(2)	7286(2)	2629.0(17)	16.8(4)
C20	4206.4(19)	7109(2)	3333.1(17)	15.6(4)
C21	4549(2)	8351(2)	3842.7(19)	21.4(4)
C22	1221(2)	9162(2)	2246.0(19)	23.4(4)
C23	186(3)	8854(3)	3003(3)	39.1(6)

Table S6 Anisotropic Displacement Parameters (Å^2×10^3) for 007c-24016. The Anisotropic displacement factor exponent takes the form: $-2\pi^2[h^2a^{*2}U_{11}+2hka^*b^*U_{12}+\ldots]$.

Atom	U_{11}	U_{22}	U_{33}	U_{23}	U_{13}	U_{12}
O1	63.0(12)	29.9(9)	29.5(9)	11.6(7)	25.0(8)	28.5(9)

Table S6 Anisotropic Displacement Parameters (Å^2×10^3) for 007c-24016. The Anisotropic displacement factor exponent takes the form: -2π^2[h^{2}a*^{2}U$_{11}$+2hka*b*U$_{12}$+…].

Atom	U_{11}	U_{22}	U_{33}	U_{23}	U_{13}	U_{12}
O2	79.3(15)	36.3(11)	35.1(10)	10.2(8)	30.5(10)	34.6(10)
O3	16.3(6)	25.0(8)	28.0(8)	-1.3(6)	7.3(6)	5.8(6)
N1	13.9(7)	14.8(8)	12.4(7)	1.4(6)	4.4(5)	2.8(6)
N2	19.1(8)	18.2(8)	23.0(9)	-3.6(7)	3.0(6)	0.9(7)
N3	18.4(8)	16.6(8)	16.6(8)	-0.6(6)	2.8(6)	2.5(6)
C1	12.2(8)	20.6(9)	12.2(8)	1.7(7)	2.8(6)	2.6(7)
C2	12.8(8)	16.5(9)	13.1(8)	0.6(7)	3.2(6)	1.1(7)
C3	10.7(7)	11.7(8)	17.6(8)	-0.5(7)	3.2(6)	0.3(6)
C4	11.0(8)	14.6(9)	18.3(9)	-0.7(7)	3.5(6)	1.1(6)
C5	14.2(8)	14.7(8)	13.5(8)	0.3(6)	3.6(6)	1.2(7)
C6	14.3(8)	14.5(9)	19.5(9)	-0.8(7)	7.5(7)	0.4(7)
C7	14.7(8)	18.4(9)	17.6(9)	-3.4(7)	5.1(7)	-2.6(7)
C8	25.2(10)	17.0(10)	24.2(10)	1.8(8)	11.3(8)	2.6(8)
C9	110(3)	46.0(18)	41.0(15)	27.0(14)	41.6(18)	55(2)
C10	12.5(8)	16.7(9)	13.5(8)	-2.4(7)	3.0(6)	-1.4(7)
C11	15.1(8)	14.8(8)	15.4(8)	-0.9(7)	3.6(6)	-1.7(7)
C12	11.9(8)	17.2(9)	19.4(9)	-6.9(7)	2.2(7)	-0.8(7)
C13	14.3(8)	23.6(10)	16.8(9)	-5.2(8)	6.7(7)	-3.7(7)
C14	17.3(8)	19.8(9)	14.6(8)	-0.6(7)	2.7(7)	-4.9(7)
C15	13.9(8)	16.4(9)	15.7(8)	-0.6(7)	1.1(6)	0.6(7)
C16	14.8(8)	27.8(10)	12.3(8)	-1.1(7)	2.6(7)	-1.7(8)
C17	27.5(10)	17.5(10)	21.1(10)	6.1(7)	7.7(8)	5.1(8)
C18	15.6(8)	15.6(9)	18.0(8)	0.1(7)	8.4(7)	2.3(7)
C19	19.6(9)	17.5(10)	13.5(8)	-1.3(7)	4.1(7)	2.3(7)
C20	15.7(8)	18.2(9)	14.8(8)	-0.3(7)	7.7(7)	2.1(7)
C21	18.4(9)	20.4(10)	24.9(10)	-3.0(8)	3.3(8)	1.7(8)
C22	23.9(10)	19.7(10)	22.8(10)	-1.9(8)	-3.7(8)	9.2(8)
C23	22.2(11)	49.3(17)	44.8(15)	-1.4(12)	5.1(10)	13.4(11)

Table S7 Bond Lengths for 007c-24016.

Atom	Atom	Length/Å	Atom	Atom	Length/Å
O1	C8	1.333(3)	C3	C4	1.342(3)
O1	C9	1.444(3)	C3	C10	1.489(2)
O2	C8	1.198(3)	C4	C5	1.507(2)
O3	C12	1.367(2)	C5	C6	1.563(3)
N1	C1	1.501(2)	C6	C7	1.535(3)
N1	C5	1.489(2)	C6	C8	1.514(3)
N1	C18	1.485(2)	C10	C11	1.404(3)
N2	N3	1.359(2)	C10	C15	1.400(3)
N2	C21	1.343(3)	C11	C12	1.399(3)
N3	C19	1.347(3)	C12	C13	1.395(3)
N3	C22	1.462(2)	C13	C14	1.393(3)
C1	C2	1.557(3)	C14	C15	1.397(3)
C1	C17	1.526(3)	C18	C20	1.505(3)
C2	C3	1.537(2)	C19	C20	1.378(3)
C2	C7	1.555(3)	C20	C21	1.399(3)
C2	C16	1.532(3)	C22	C23	1.518(4)

Table S8 Bond Angles for 007c-24016.

Atom	Atom	Atom	Angle/°	Atom	Atom	Atom	Angle/°
C8	O1	C9	115.66(19)	C7	C6	C5	107.19(15)
C5	N1	C1	111.30(14)	C8	C6	C5	110.46(16)
C18	N1	C1	113.08(14)	C8	C6	C7	113.01(16)
C18	N1	C5	111.38(14)	C6	C7	C2	111.59(15)
C21	N2	N3	104.14(17)	O1	C8	C6	110.45(17)
N2	N3	C22	120.54(17)	O2	C8	O1	122.9(2)
C19	N3	N2	111.91(16)	O2	C8	C6	126.6(2)
C19	N3	C22	127.05(18)	C11	C10	C3	118.36(17)
N1	C1	C2	109.72(14)	C15	C10	C3	122.84(17)
N1	C1	C17	109.38(16)	C15	C10	C11	118.79(17)
C17	C1	C2	113.30(16)	C12	C11	C10	120.73(18)

Table S8 Bond Angles for 007c-24016.

Atom	Atom	Atom	Angle/°	Atom	Atom	Atom	Angle/°
C3	C2	C1	107.69(14)	O3	C12	C11	122.44(18)
C3	C2	C7	106.15(15)	O3	C12	C13	117.49(17)
C7	C2	C1	107.06(14)	C13	C12	C11	120.07(17)
C16	C2	C1	111.22(16)	C14	C13	C12	119.24(17)
C16	C2	C3	116.97(15)	C13	C14	C15	120.98(18)
C16	C2	C7	107.21(15)	C14	C15	C10	120.08(17)
C4	C3	C2	112.19(16)	N1	C18	C20	112.94(15)
C4	C3	C10	122.42(16)	N3	C19	C20	107.74(18)
C10	C3	C2	125.25(16)	C19	C20	C18	125.94(18)
C3	C4	C5	115.40(16)	C19	C20	C21	104.15(17)
N1	C5	C4	107.46(15)	C21	C20	C18	129.87(18)
N1	C5	C6	110.63(14)	N2	C21	C20	112.05(18)
C4	C5	C6	106.89(15)	N3	C22	C23	111.69(19)

Table S9 Torsion Angles for 007c-24016.

A	B	C	D	Angle/°	A	B	C	D	Angle/°
O3	C12	C13	C14	-177.46(17)	C5	C6	C7	C2	1.07(19)
N1	C1	C2	C3	-53.70(19)	C5	C6	C8	O1	64.4(2)
N1	C1	C2	C7	60.09(18)	C5	C6	C8	O2	-114.7(3)
N1	C1	C2	C16	176.92(15)	C7	C2	C3	C4	-58.7(2)
N1	C5	C6	C7	59.31(18)	C7	C2	C3	C10	117.01(19)
N1	C5	C6	C8	-177.17(15)	C7	C6	C8	O1	-175.52(18)
N1	C18	C20	C19	116.0(2)	C7	C6	C8	O2	5.4(3)
N1	C18	C20	C21	-66.6(3)	C8	C6	C7	C2	-120.87(17)
N2	N3	C19	C20	-0.7(2)	C9	O1	C8	O2	-3.0(4)
N2	N3	C22	C23	-92.6(2)	C9	O1	C8	C6	177.9(3)
N3	N2	C21	C20	-0.2(2)	C10	C3	C4	C5	-175.27(17)
N3	C19	C20	C18	178.50(17)	C10	C11	C12	O3	179.05(18)
N3	C19	C20	C21	0.6(2)	C10	C11	C12	C13	-1.7(3)
C1	N1	C5	C4	56.77(19)	C11	C10	C15	C14	2.8(3)

Table S9 Torsion Angles for 007c-24016.

A	B	C	D	Angle/°	A	B	C	D	Angle/°
C1	N1	C5	C6	-59.58(18)	C11	C12	C13	C14	3.3(3)
C1	N1	C18	C20	-61.5(2)	C12	C13	C14	C15	-1.8(3)
C1	C2	C3	C4	55.7(2)	C13	C14	C15	C10	-1.2(3)
C1	C2	C3	C10	-128.60(18)	C15	C10	C11	C12	-1.3(3)
C1	C2	C7	C6	-59.05(18)	C16	C2	C3	C4	-178.20(18)
C2	C3	C4	C5	0.5(2)	C16	C2	C3	C10	-2.5(3)
C2	C3	C10	C11	-130.02(19)	C16	C2	C7	C6	-178.49(15)
C2	C3	C10	C15	50.5(3)	C17	C1	C2	C3	68.86(19)
C3	C2	C7	C6	55.78(19)	C17	C1	C2	C7	-177.35(15)
C3	C4	C5	N1	-58.7(2)	C17	C1	C2	C16	-60.5(2)
C3	C4	C5	C6	60.1(2)	C18	N1	C1	C2	-128.17(16)
C3	C10	C11	C12	179.22(17)	C18	N1	C1	C17	106.97(18)
C3	C10	C15	C14	-177.80(18)	C18	N1	C5	C4	-176.04(15)
C4	C3	C10	C11	45.2(3)	C18	N1	C5	C6	67.61(18)
C4	C3	C10	C15	-134.2(2)	C18	C20	C21	N2	-178.07(18)
C4	C5	C6	C7	-57.39(18)	C19	N3	C22	C23	78.7(3)
C4	C5	C6	C8	66.13(19)	C19	C20	C21	N2	-0.2(2)
C5	N1	C1	C2	-1.9(2)	C21	N2	N3	C19	0.5(2)
C5	N1	C1	C17	-126.77(16)	C21	N2	N3	C22	173.05(19)
C5	N1	C18	C20	172.25(15)	C22	N3	C19	C20	-172.63(19)

Table S10 Hydrogen Atom Coordinates (Å×10^4) and Isotropic Displacement Parameters (Å^2×10^3) for 007c-24016.

Atom	*x*	*y*	*z*	U(eq)
H3	12773	3014	1981	34
H1	5035	6041	1418	18
H4	9328	4819	3513	18
H5	7319	4532	4280	17
H6	5473	3334	2947	19
H7A	5204	3668	1044	20

Table S10 Hydrogen Atom Coordinates (Å×10[4]) and Isotropic Displacement Parameters (Å[2]×10[3]) for 007c-24016.

Atom	*x*	*y*	*z*	U(eq)
H7B	6656	2985	1046	20
H9A	7529	-1	4353	93
H9B	8030	713	5606	93
H9C	8946	803	4626	93
H11	10517	3544	2125	18
H13	12557	5177	-219	21
H14	10835	6785	-690	21
H15	8951	6780	223	19
H16A	6861	6092	-376	28
H16B	5518	5187	-505	28
H16C	6972	4527	-537	28
H17A	5969	8157	1928	32
H17B	6282	7709	683	32
H17C	7468	7634	1859	32
H18A	5223	5620	4370	19
H18B	4404	5112	3104	19
H19	2376	6629	2158	20
H21	5389	8541	4381	26
H22A	868	8830	1432	28
H22B	1333	10131	2202	28
H23A	-685	9301	2673	59
H23B	541	9165	3814	59
H23C	32	7898	3010	59

Table S11 Solvent masks information for 007c-24016.

Number	X	Y	Z	Volume	Electron count	Content
1	0.183	0.232	0.436	65.1	20.4	1 C6H14
2	-0.183	0.732	0.564	65.1	20.4	1 C6H14

7. Pharmacokinetic Report of Compound (±)-#020

Sample	Administration	Dose, mg/kg	Pharmacokinetic Parameters					
			Tmax, min	Cmax, ng/ml	$AUC_{0\to t\ min}$ (AUClast) ng*min/ml	$AUC_{0\to\infty}$ (AUCINF_obs) ng*min/ml	$T_{1/2}$ (HL_Lambda_z), min	K_{el} (Lambda_z), min^{-1}
Plasma	IP	10	5.00	1410	39800	40000	15.7	0.0443
Brain			15.0	174	5880	8600	31.5	0.022
SCF			5.00	48.6	1460	2340	34.8	0.0199

Table S12. Selected pharmacokinetic parameters for compound (±)-#020 in male CD-1 mice. The pharmacokinetic parameters for compound **(±)-#020** in the blood plasma, brain, and cerebrospinal fluid (CSF) following intraperitoneal (IP) injection using DMSO–Kolliphor EL–Water (1%:10%:89, v/v/v) as the formulation vehicle. 10min prior to the study, 50µL of DMSO was added to 10mg of dry **(±)-#020**, followed by 0.5mL of Kolliphor EL, 4.3ml of water, and 150µL of 20mM NaOH to a final pH of 6.97. Levels of **(±)-#020** in each sample were quantified by LC-MS/MS over time after a single 10mg/kg dose (5mL/kg).

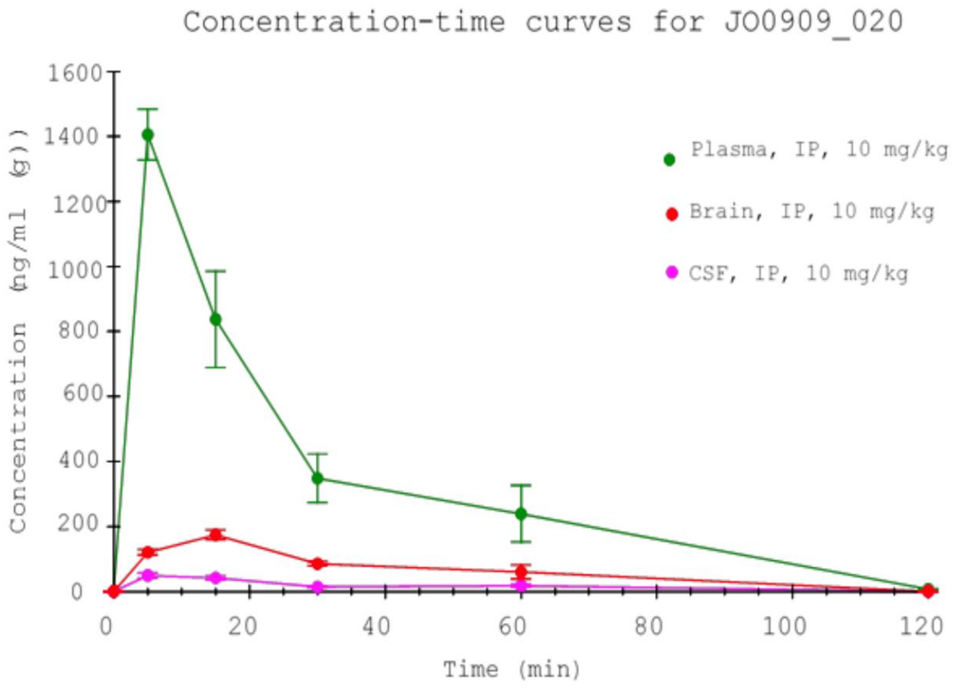

Figure S5. Concentration-time curves for compound (±)-#020 (JO0909_020) in

male CD-1 mice following IP administration. Summarization of the results from of the pharmacokinetic study across multiple timepoints after a single dose.

8. CryoEM Validation Statistics

	**MOR with #33**	**MOR-Nb6M-NabFab-AntiNb Fab complex with #020_E1**	**MOR with #020_E1**	**KOR-Nb6M-NabFab-AntiNb Fab complex with #020_E1**	**KOR with #020_E1**
**Data Collection**					
Microscope	UCSF EM Core, Titan Krios G3i	UCSF EM Core, Titan Krios G3i		UCSF EM Core, Talos Arctica	
Detector	K3	K3		K3	
Voltage (kV)	300	300		200	
Magnification	105,000x	105,000x		45,000x	
Defocus range (µm)	0.8 to 2.1	0.8 to 2.1		0.8 to 2.1	
Pixel size (Å)	0.835	0.8189		0.865	
Total exposure (e^-/Å^2)	45.8	47.7		64.1	
Frame exposure (e^-/Å^2/frame)	0.57	0.596		0.855	
# Frames/Image	80	80		75	
# Images	7.766	9,877		4,967	
# Initial Particles	1,923,561	5,949,666		2,056,565	
# Final Particles	40,722	259,816		98,518	
Symmetry imposed	C1	C1		C1	
Map sharpening *B* factor (Å^2)	-132	-140	-127	-114	-100
Map resolution (Å)	3.90	3.30	3.23	3.18	2.96

FSC threshold	0.143	0.143	0.143	0.143	0.143
**Refinement and validation**					
Initial model used (PDB)	7UL4	7UL4, 7UL3, 7PIJ	7UL4	AF-P41145-F1(Dimer), 7UL3, 7PIJ	AF-P41145-F1 (Monomer)
Model resolution (Å)	4.1	3.8	3.7	3.3	3.1
FSC threshold	0.5	0.5	0.5	0.5	0.5
Model composition					
Chains	1	5	2	7	2
Non-hydrogen atoms	2074	5654	2134	6918	2303
Protein residues	281	785	286	896	285
Water	0	0	0	0	0
Ligands	0	1	1	2	1
B factors ($Å^2$)					
Protein (min/max/mean)	34.66/112.17/70.96	3.99/148.44/65.32	13.63/128.54/64.06	14.76/211.73/92.60	25.49/132.65/55.20
Ligand (min/max/mean)	N/A	44.92/77.28/56.34	20.00/20.00/20.00	31.41/63.88/47.46	40.41/80.23/58.60
R.m.s. deviations					
Bond lengths (Å)	0.003	0.002	0.007	0.004	0.003
Bond angles (°)	0.588	0.572	1.081	0.671	0.512
**Validation**					
MolProbity score	1.97	1.70	1.51	1.87	1.67
Clash score	4.68	7.56	7.48	7.27	5.56
EMRinger score	1.35	1.30	2.26	2.11	2.45

Poor rotamers (%)	4.90	2.38	0.97	2.14	1.95
Ramachandran plot					
Favored (%)	96.75	97.99	97.52	96.64	97.15
Allowed (%)	3.25	2.01	2.48	3.36	2.49
Disallowed (%)	0	0	0	0	0.36

9. References

1) Huang, L. J.; Wang, J. P.; Lai, Y. C.; Kuo, S. C. Synthesis of (2*E*)-3-{2-[(Substituted Benzyl)Oxy]Phenyl}acrylaldehydes as Novel Anti-Inflammatory Agents. *Bioorganic Med. Chem. Lett.* **2006**, *16* (10), 2742–2747. https://doi.org/10.1016/j.bmcl.2006.02.005.

2) Bew, S. P.; Hiatt-Gipson, G. D.; Mills, G. P.; Reeves, C. E. Efficient Syntheses of Climate Relevant Isoprene Nitrates and (1R,5S)-(-)-Myrtenol Nitrate. *Beilstein J. Org. Chem.* **2016**, *12*, 1081–1095. https://doi.org/10.3762/bjoc.12.103.

3) Gong, L.; Hogg, J. H.; Collier, J.; Wilhelm, R. S.; Soderberg, C. Design and Synthesis of Novel CCR3 Antagonists. *Bioorganic Med. Chem. Lett.* **2003**, *13* (20), 3597–3600. https://doi.org/10.1016/ S0960-894X(03)00748-0.

4) Yue, C.; Yuan, Z.; Xu, G.; Guan, X. N.; Wei, B.; Yao, H.; Yang, C. G.; Zhang, T. Structure-Guided Design, Synthesis, and Antivirulence Assessment of Covalent Staphylococcus Aureus Sortase A Inhibitors. *J. Med. Chem.* **2024**, *67* (2), 1127–1146. https://doi.org/10.1021/acs.jmedchem.3c01615.

5) Jeong, H. W.; Kim, M. R.; Son, K. H.; Young Han, M.; Ha, J. H.; Garnier, M.; Meijer, L.; Kwon, B. M. Cinnamaldehydes Inhibit Cyclin Dependent Kinase 4/Cyclin D1. *Bioorganic Med. Chem. Lett.* **2000**, *10* (16), 1819–1822. https://doi.org/10.1016/S0960-894X(00)00357-7.

6) Shi, S. L.; Wong, Z. L.; Buchwald, S. L. Copper-Catalysed Enantioselective Stereodivergent Synthesis of Amino Alcohols. *Nature* **2016**, *532* (7599), 353–356. https://doi.org/10.1038/nature17191.

7) Baxendale, I. R.; Griffiths-Jones, C. M.; Ley, S. V.; Tranmer, G. K. Microwave-Assisted Suzuki Coupling Reactions with an Encapsulated Palladium Catalyst for Batch and Continuous-Flow Trans- formations. *Chem. - A Eur. J.* **2006**, *12* (16), 4407–4416. https://doi.org/10.1002/ chem.200501400.

8) Ndungu, J. M.; Larson, K. K.; Sarpong, R. Development of an Anomalous Heck Reaction: Skeletal Rearrangement of Divinyl and Enyne Carbinols. *Org. Lett.* **2005**, *7* (26), 5845–5848. https://doi.org/10.1021/ol052382p.

10. NMR spectra of all synthesized compounds

Proton (#03)

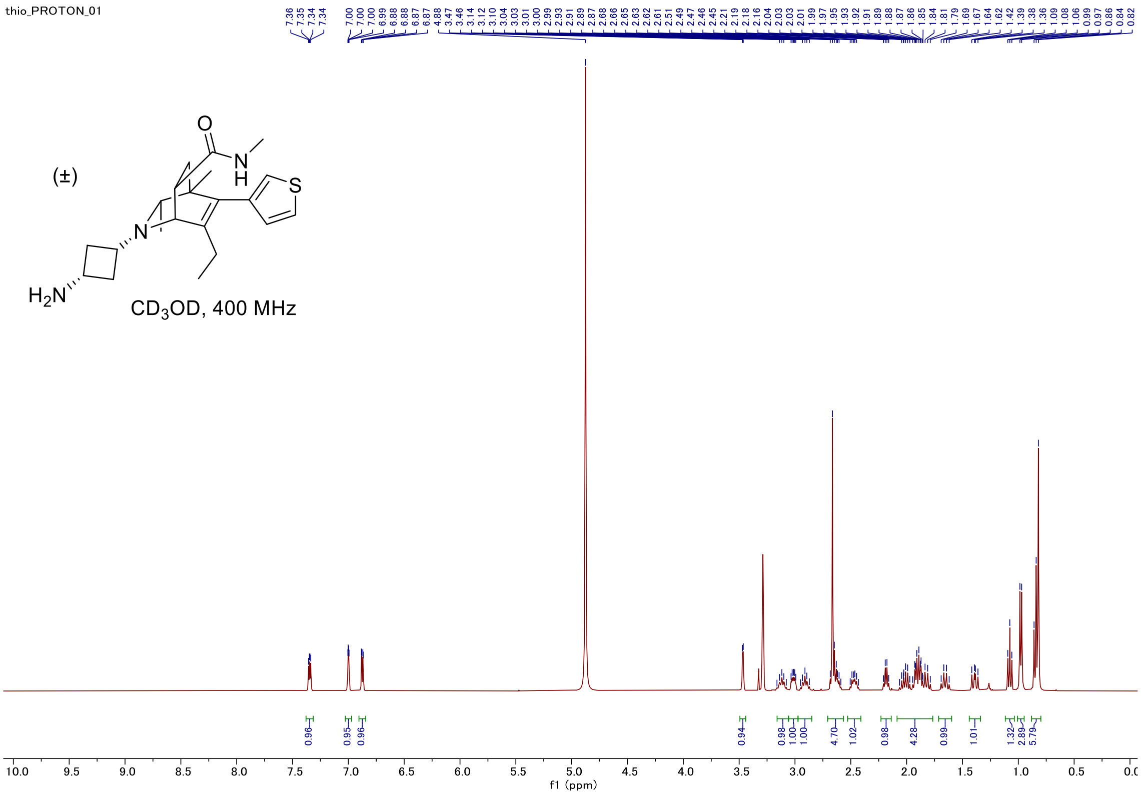

Carbon

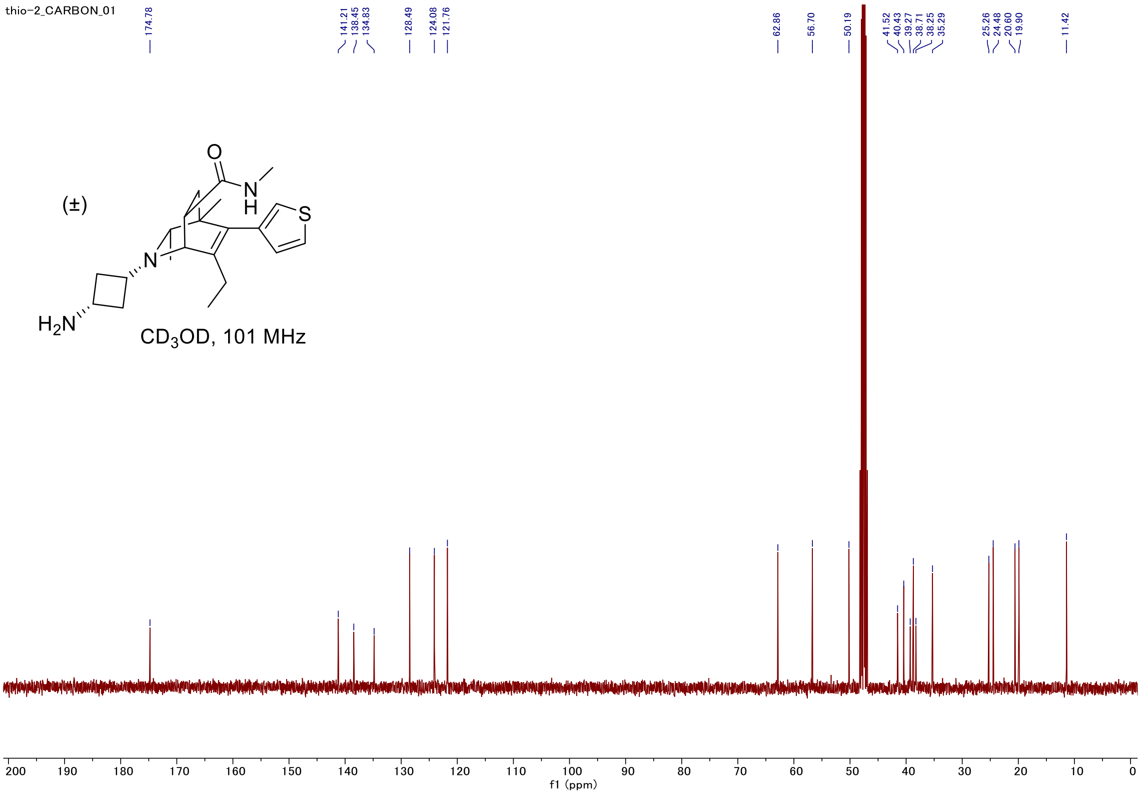

C-H COSY

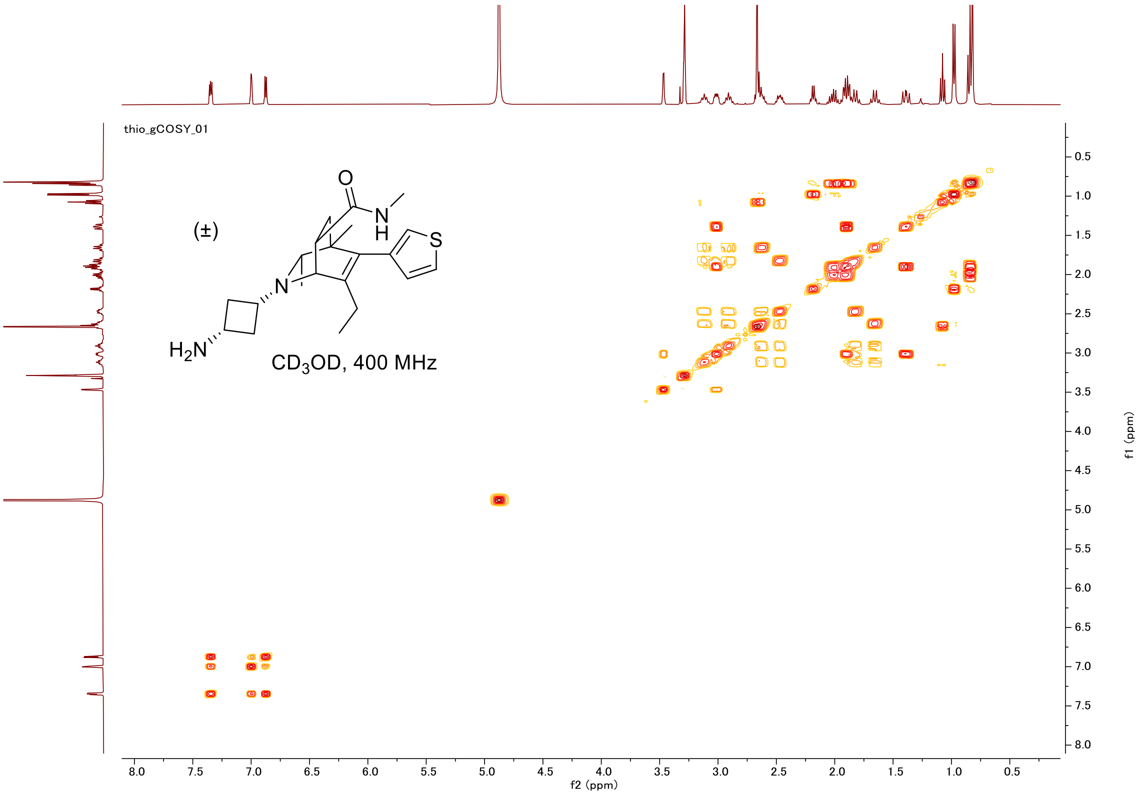
thio_gCOSY_01
(±)
O
N
H
S
N
H2N
CD3OD, 400 MHz
f2 (ppm)
f1 (ppm)

HSQC

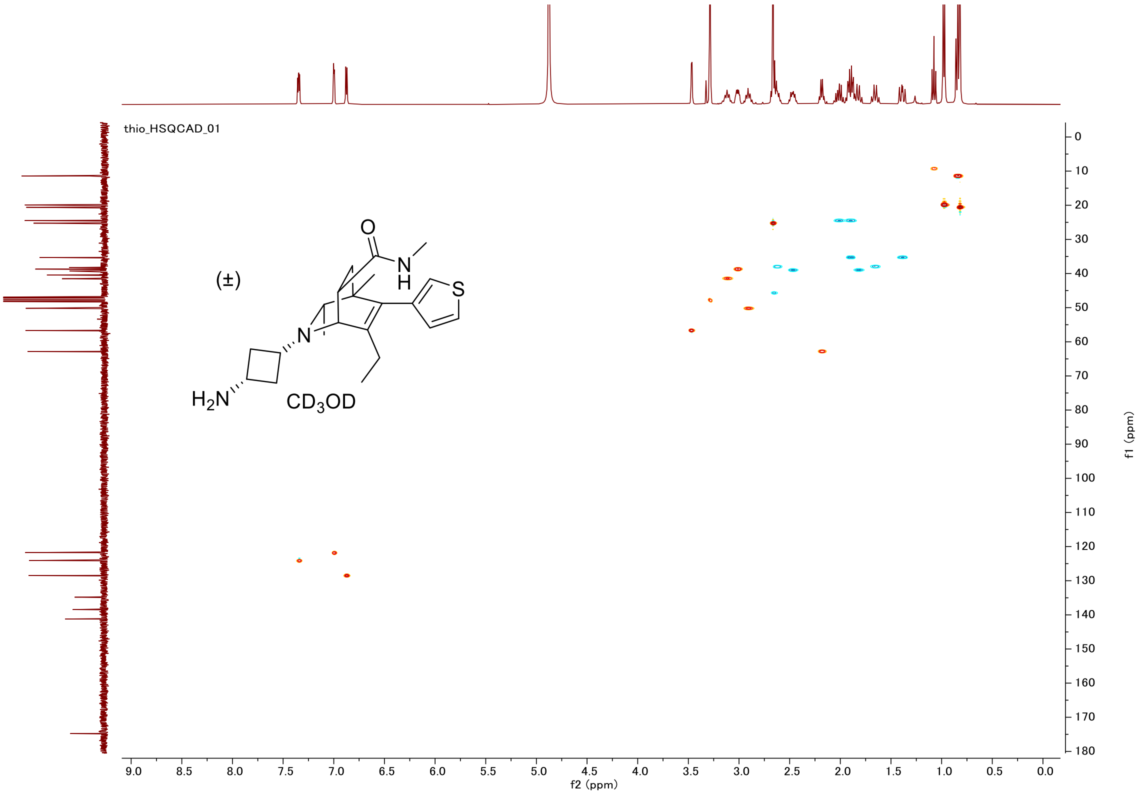
thio_HSQCAD_01
(±)
O
N
H
S
N
H2N
CD3OD
f2 (ppm)
f1 (ppm)

Proton (#05 isomer 1)

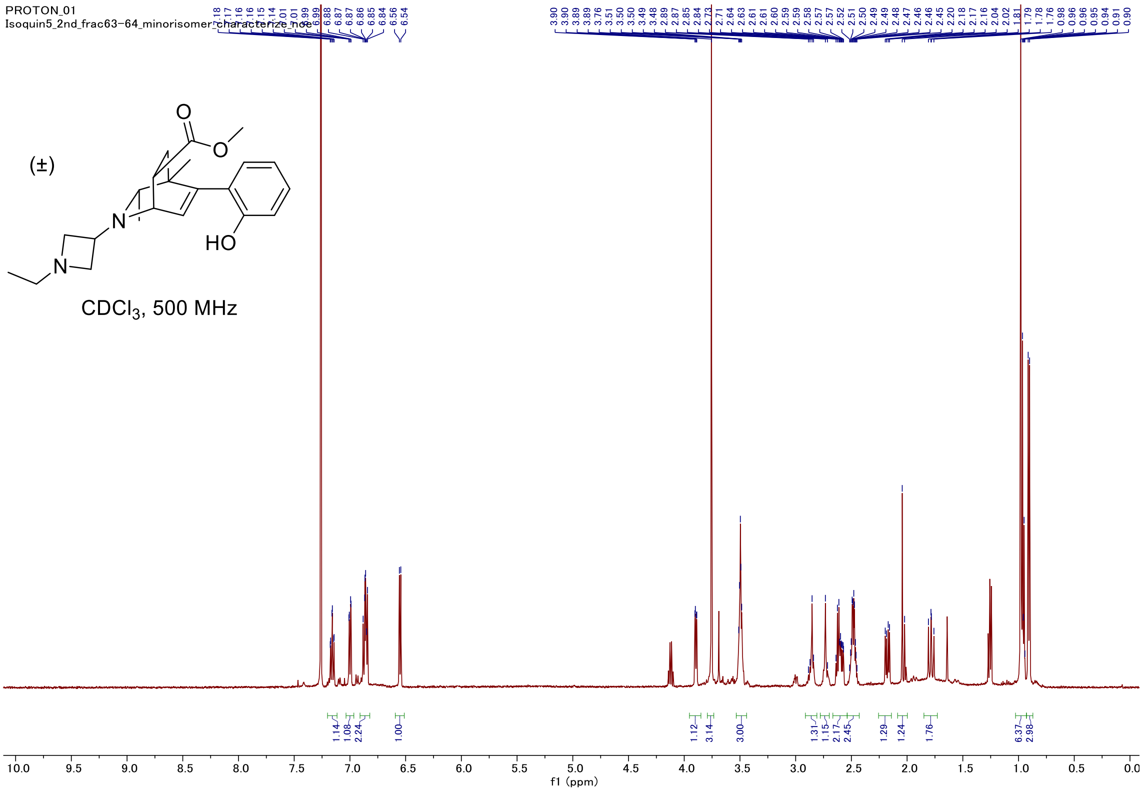

Carbon

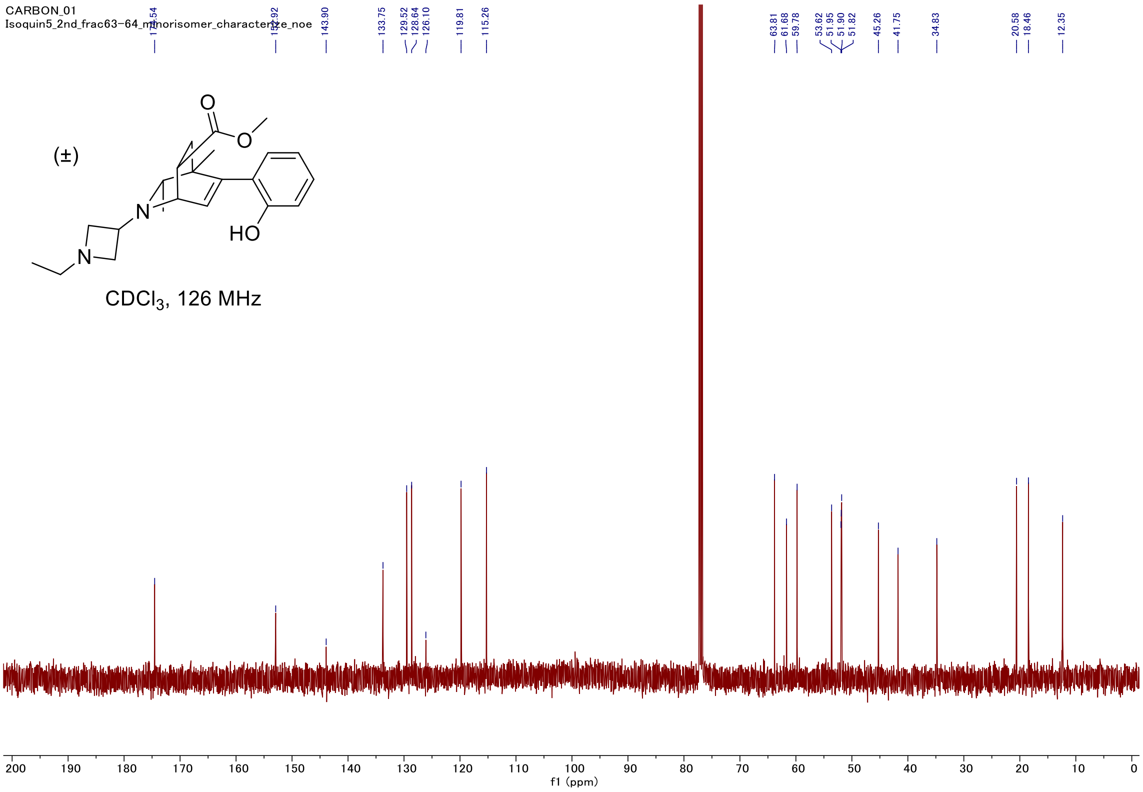

C-H COSY

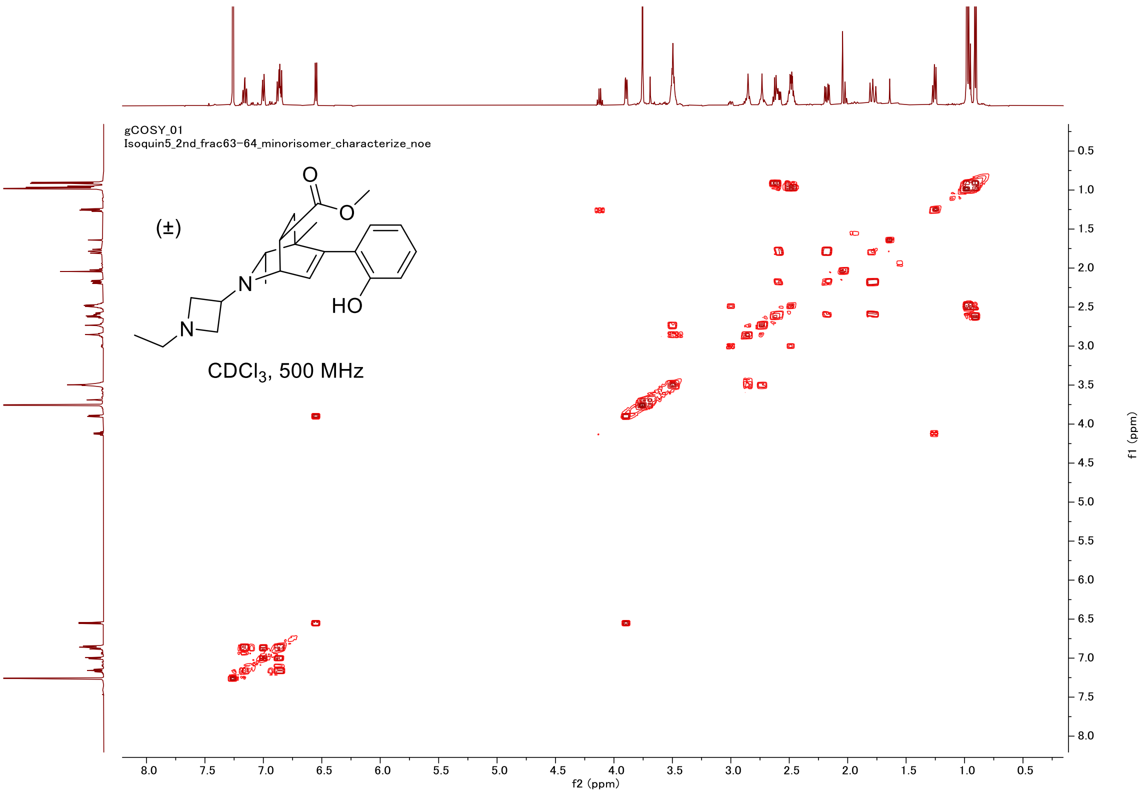
gCOSY_01
Isoquin5_2nd_frac63-64_minorisomer_characterize_noe
(±)
O
O
N
N
HO
CDCl3, 500 MHz
f2 (ppm)
f1 (ppm)

HSQC

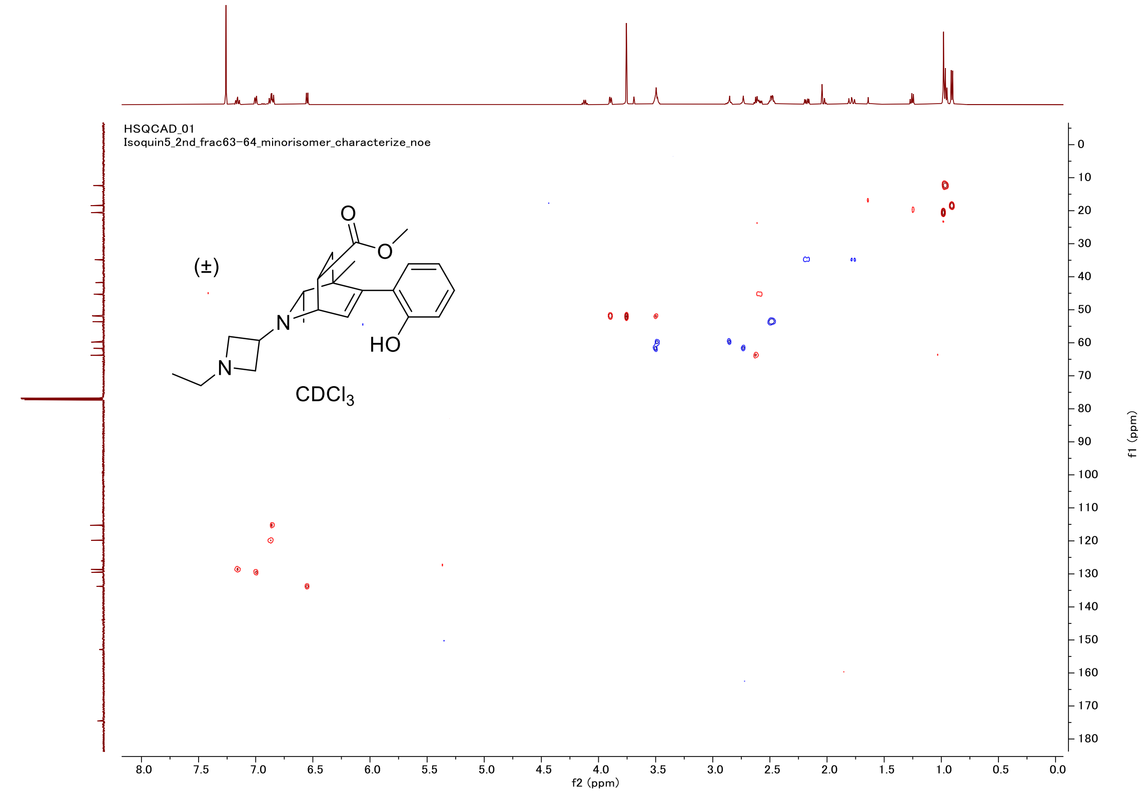
HSQCAD_01
Isoquin5_2nd_frac63-64_minorisomer_characterize_noe
(±)
O
O
N
N
HO
CDCl3
f2 (ppm)
f1 (ppm)

Proton (#05 isomer 2)

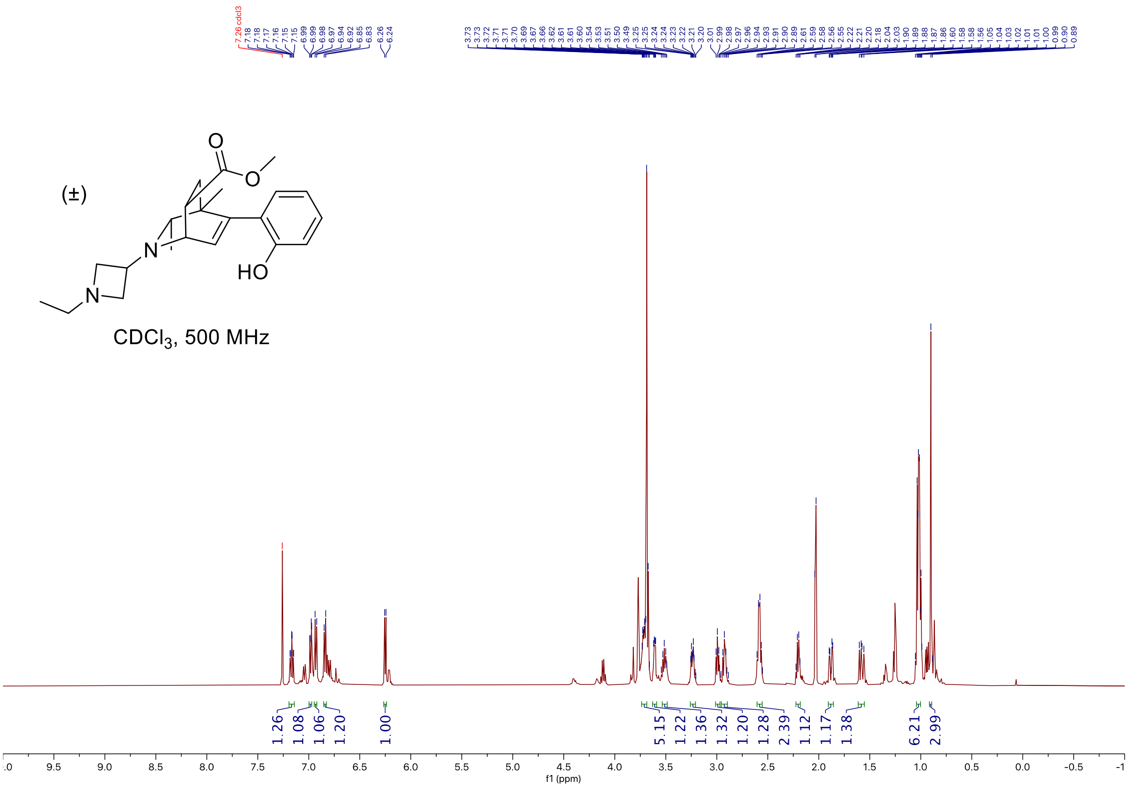

Carbon

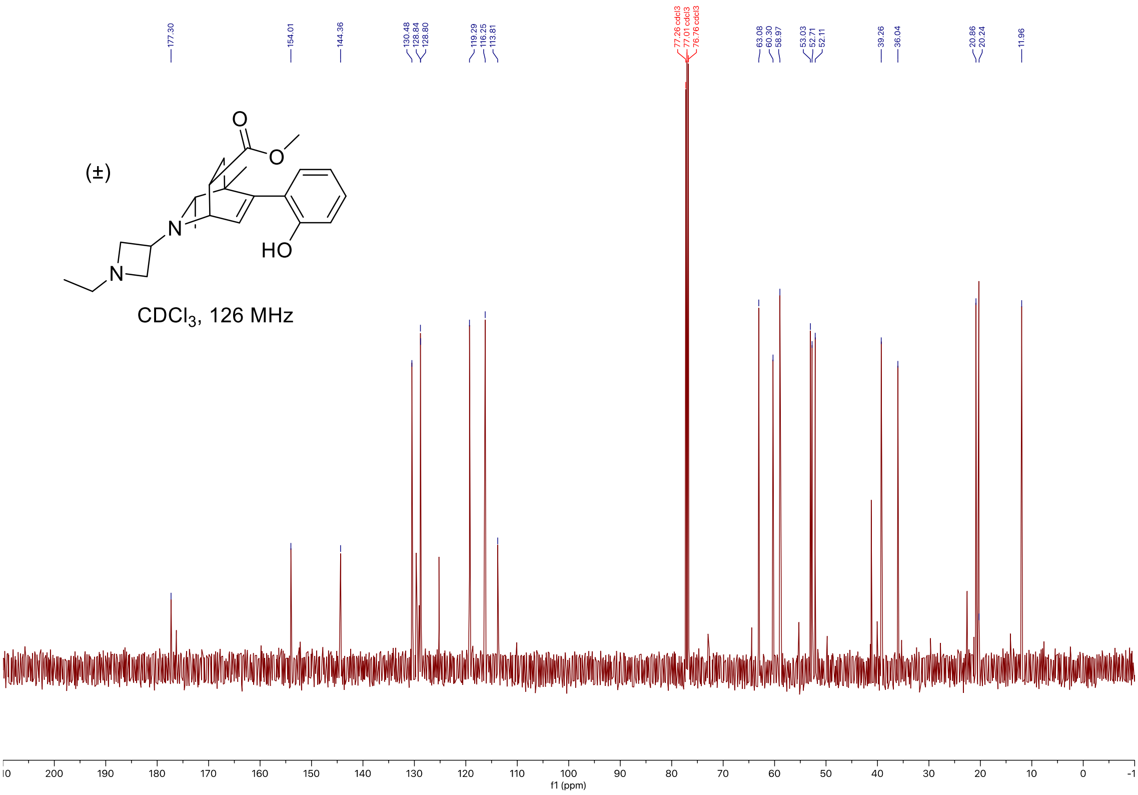

C-H COSY

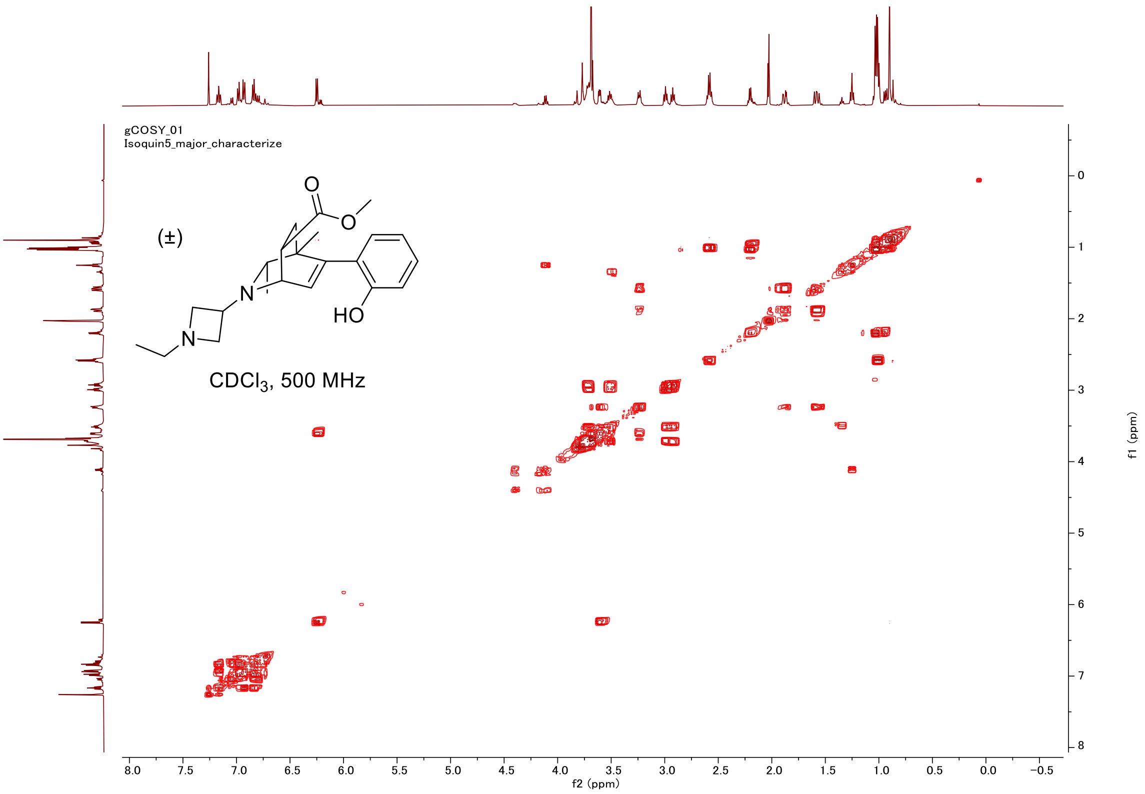
gCOSY_01
Isoquin5_major_characterize
(±)
O
O
N
N
HO
CDCl3, 500 MHz
f2 (ppm)
f1 (ppm)

HSQC

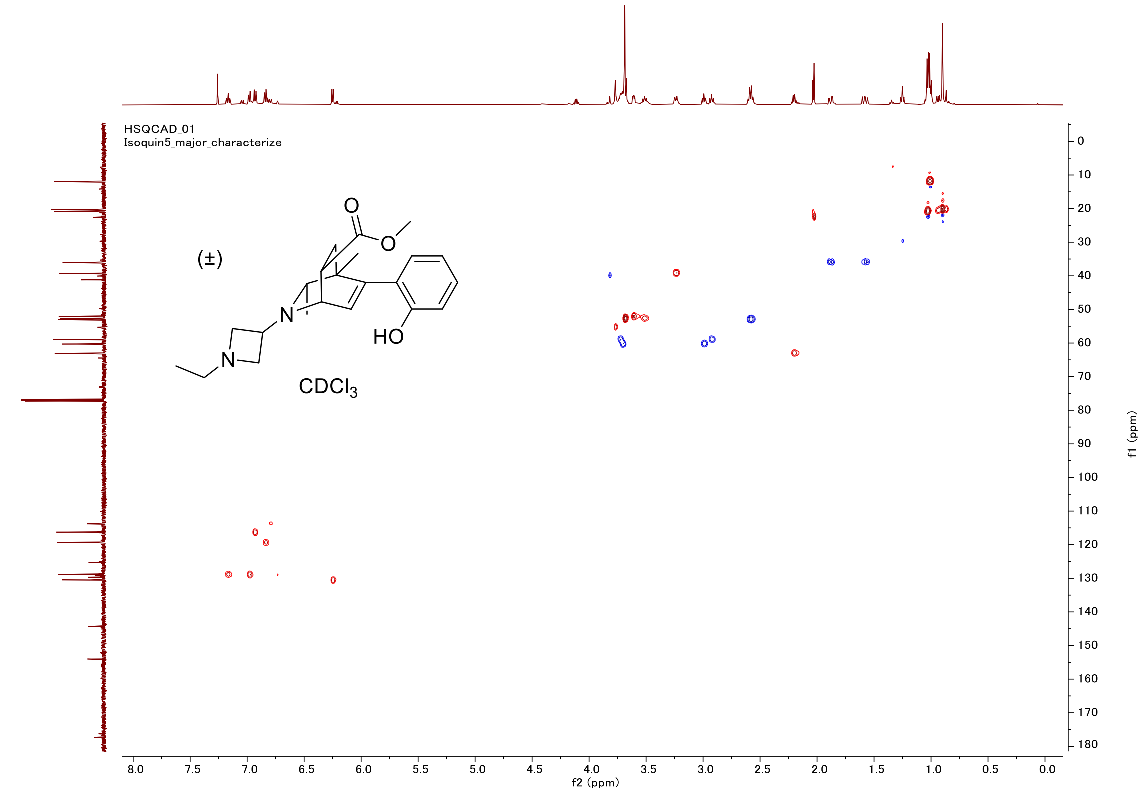
HSQCAD_01
Isoquin5_major_characterize
(±)
O
O
N
N
HO
CDCl3
f2 (ppm)
f1 (ppm)

Proton (#06)

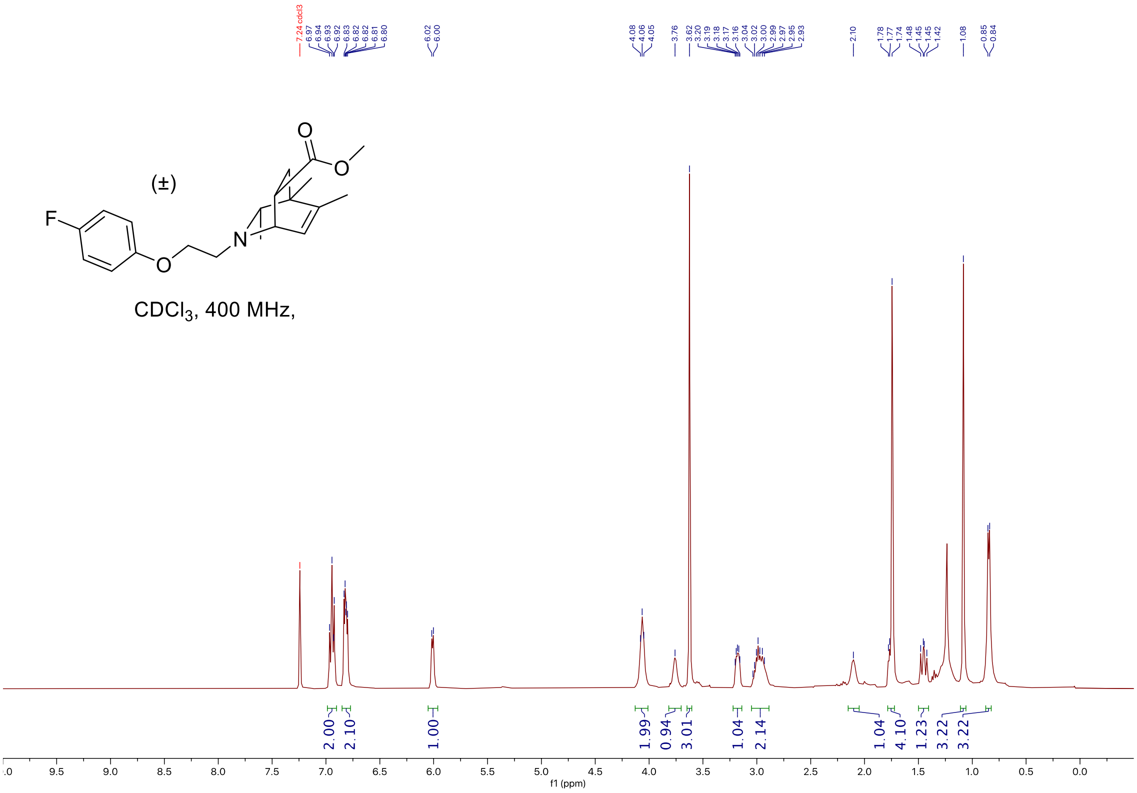

Carbon

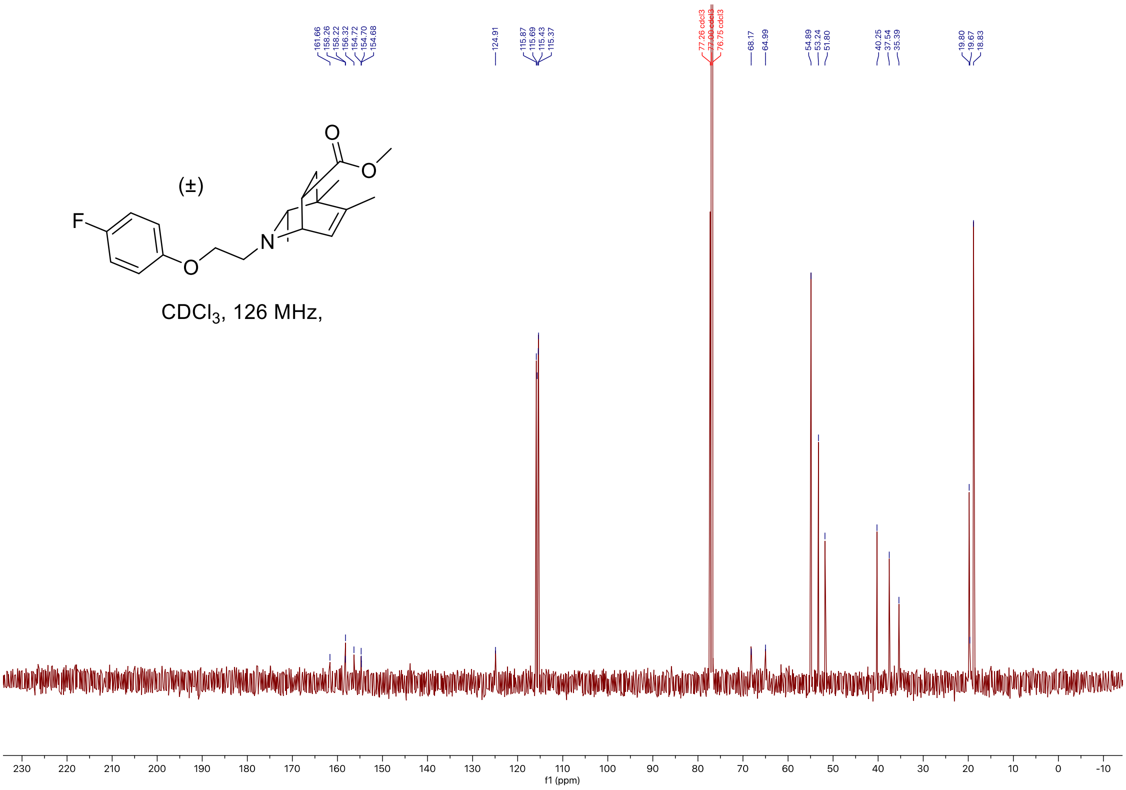

Fluorine

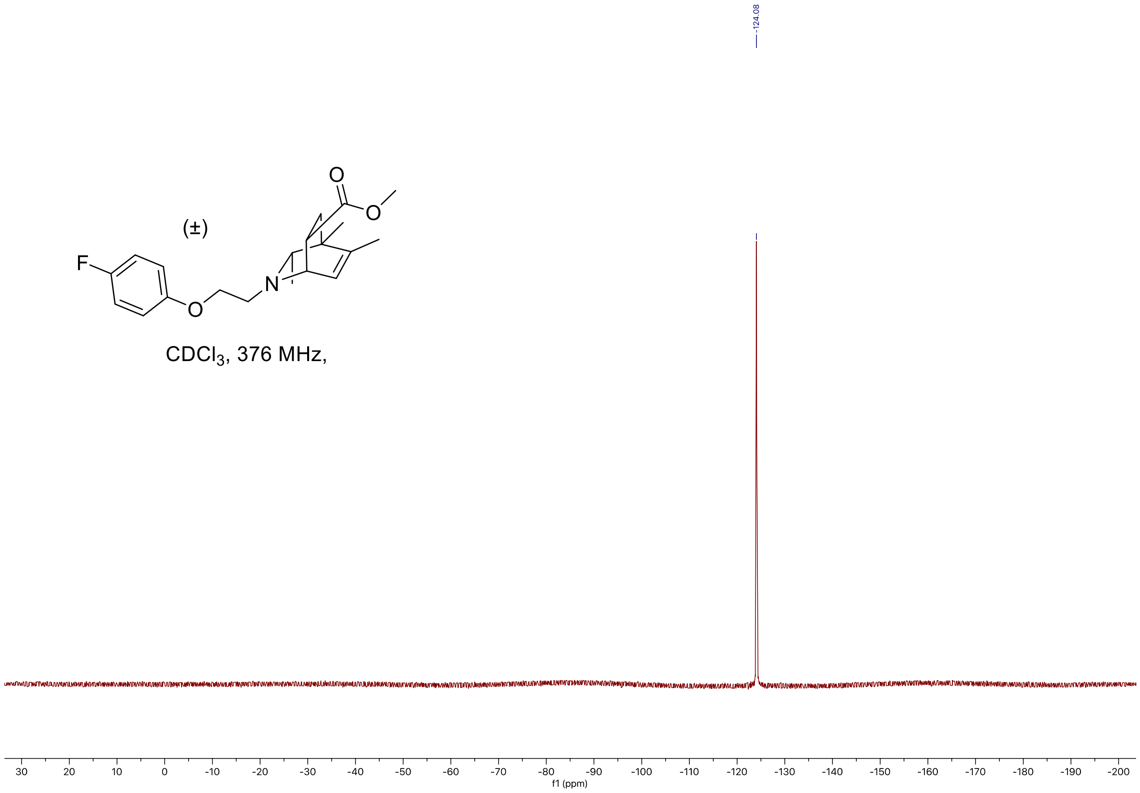

C-H COSY

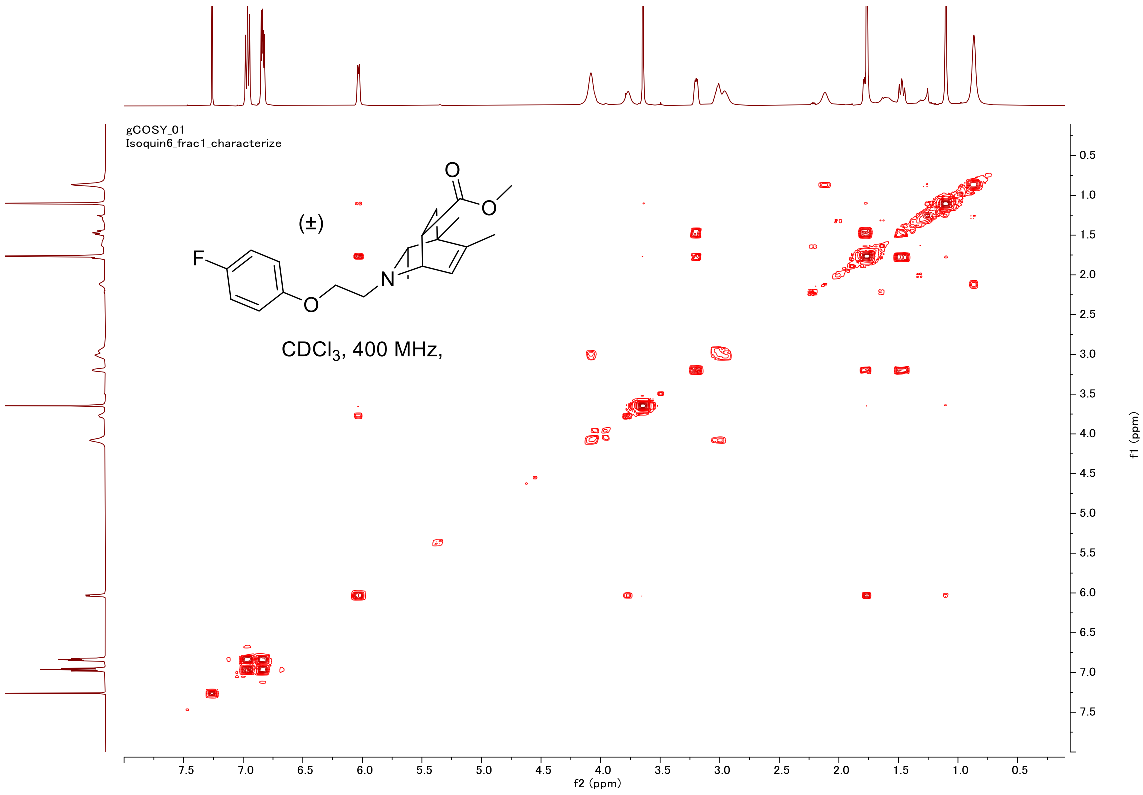

HSQC

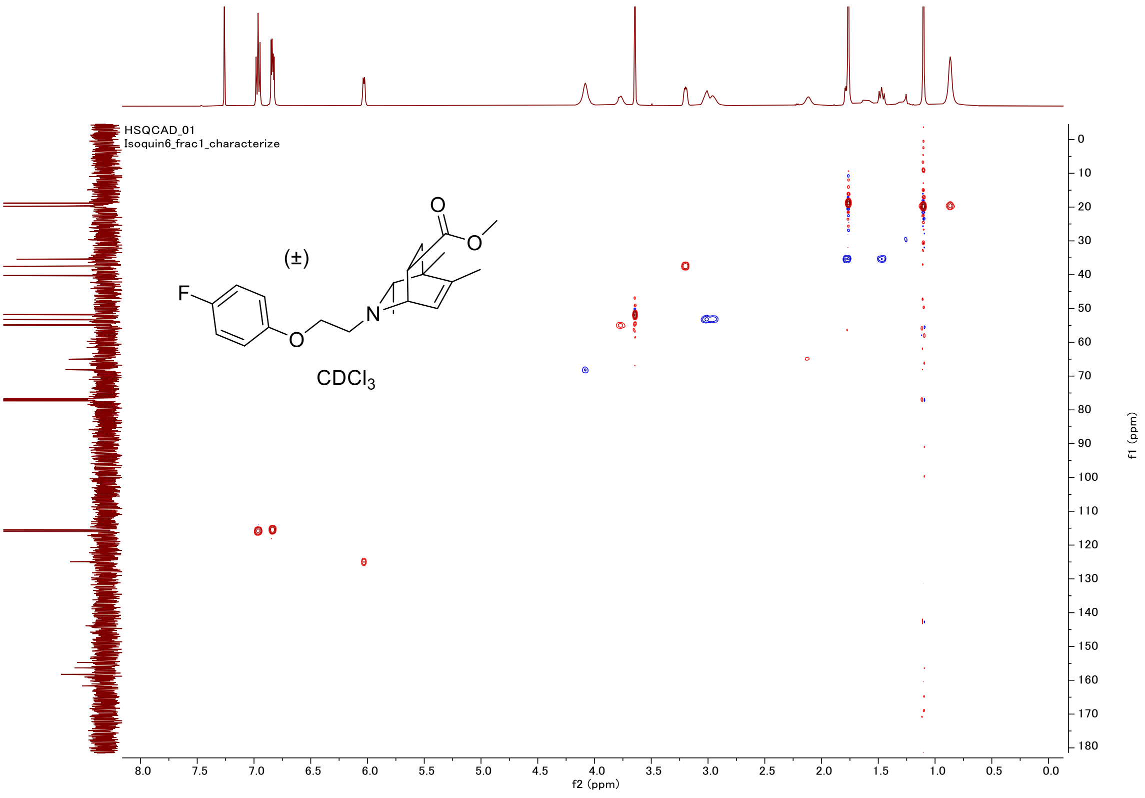
HSQCAD_01
Isoquin6_frac1_characterize
(±)
F
O
N
O
O
CDCl3
f2 (ppm)
f1 (ppm)

Proton (#09)

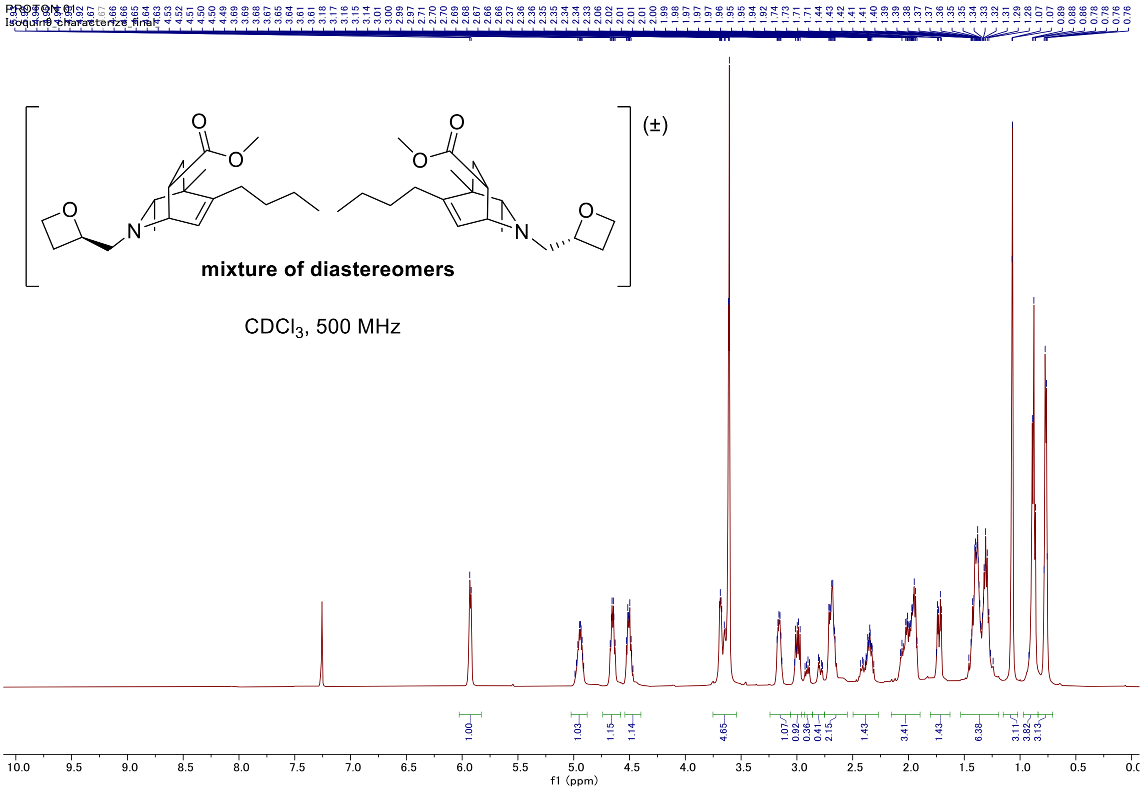

Carbon

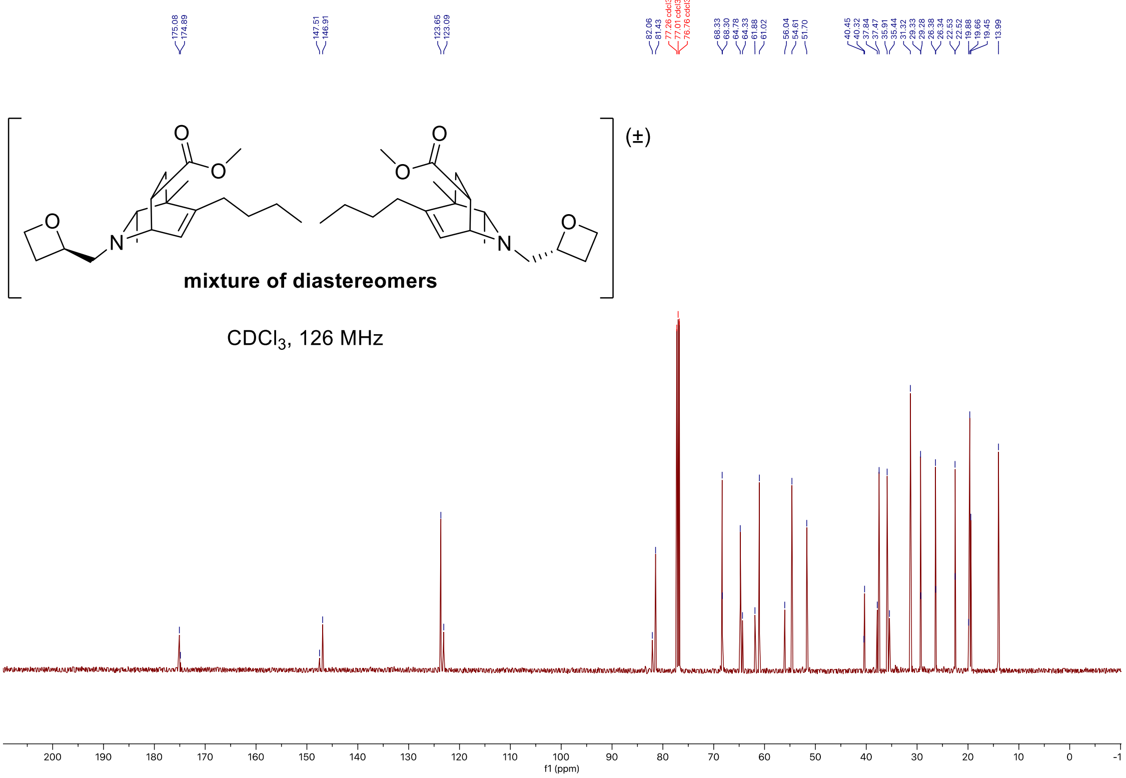

C-H COSY

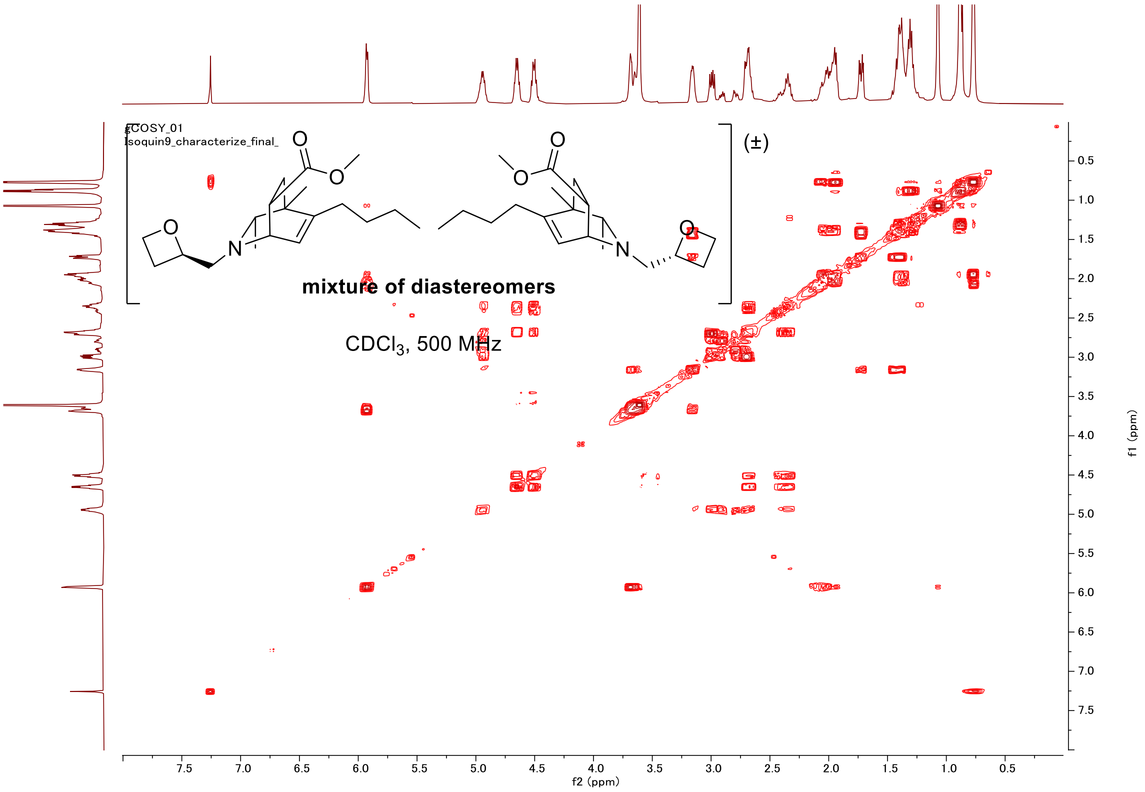

COSY_01
Isoquin9_characterize_final_
(±)
mixture of diastereomers
CDCl3, 500 MHz
f2 (ppm)
f1 (ppm)

HSQC

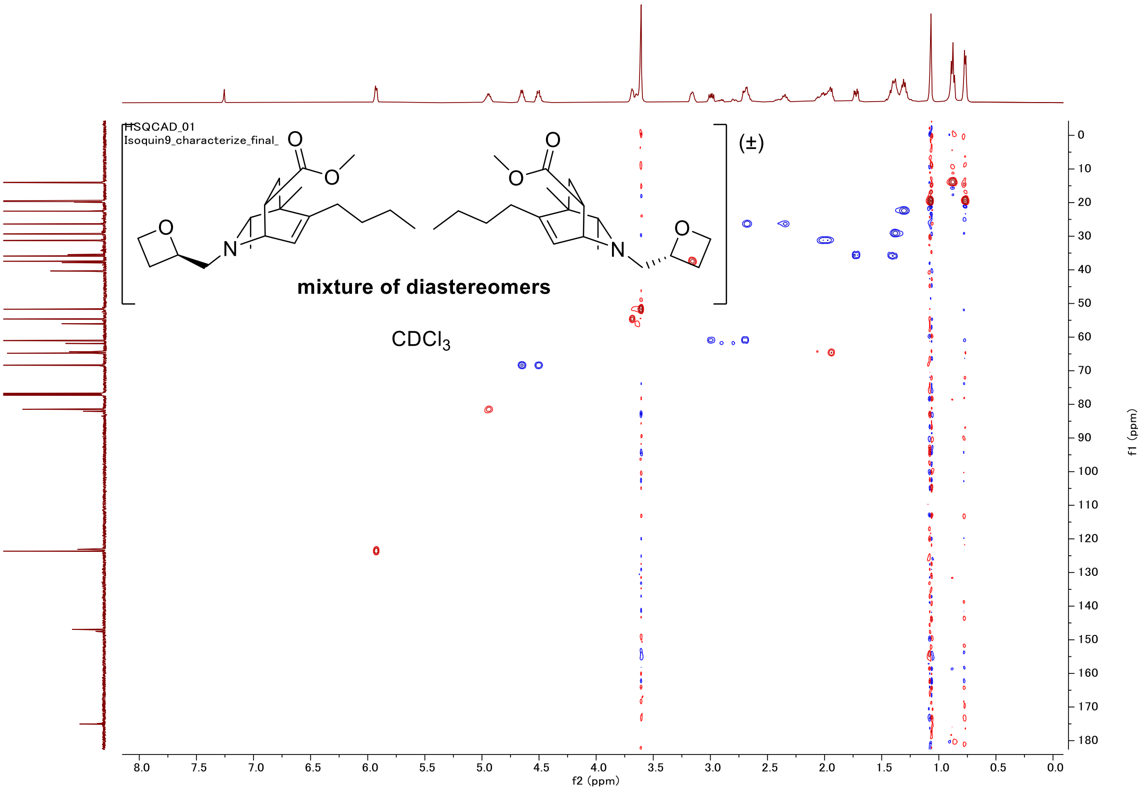

HSQCAD_01
Isoquin9_characterize_final_
(±)
mixture of diastereomers
CDCl3
f2 (ppm)
f1 (ppm)

Proton (#11, isomer1)

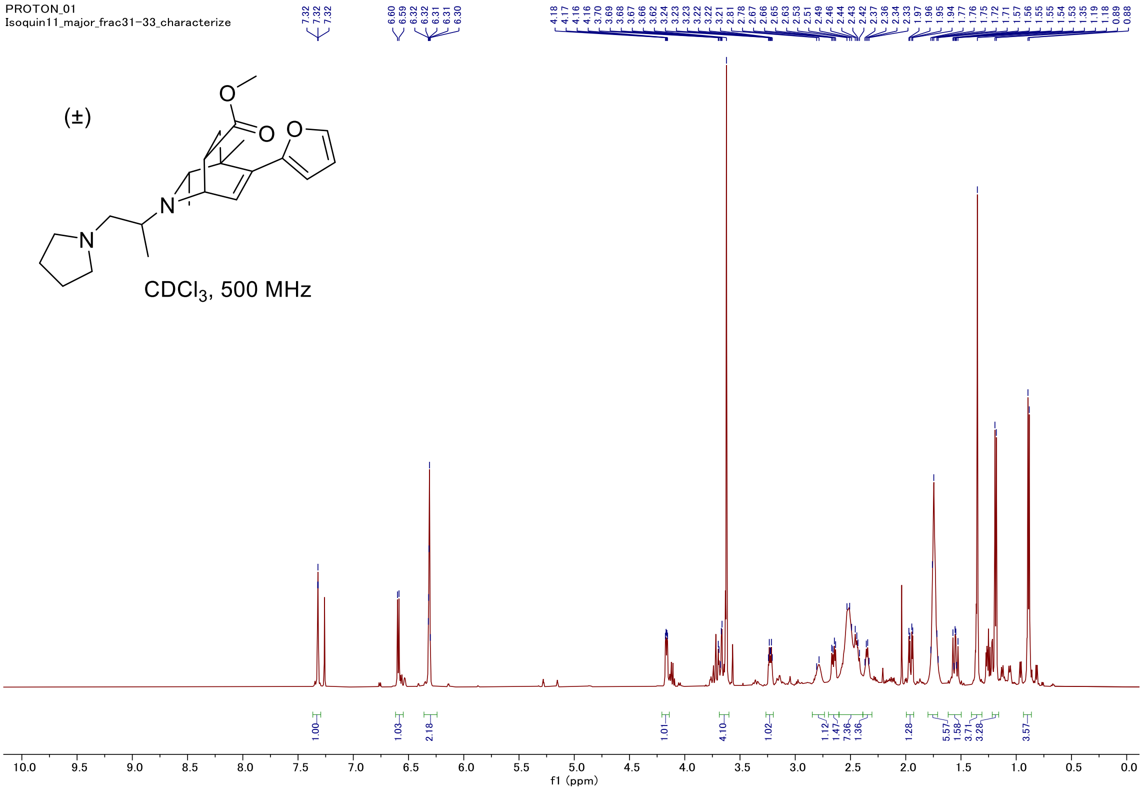

Carbon

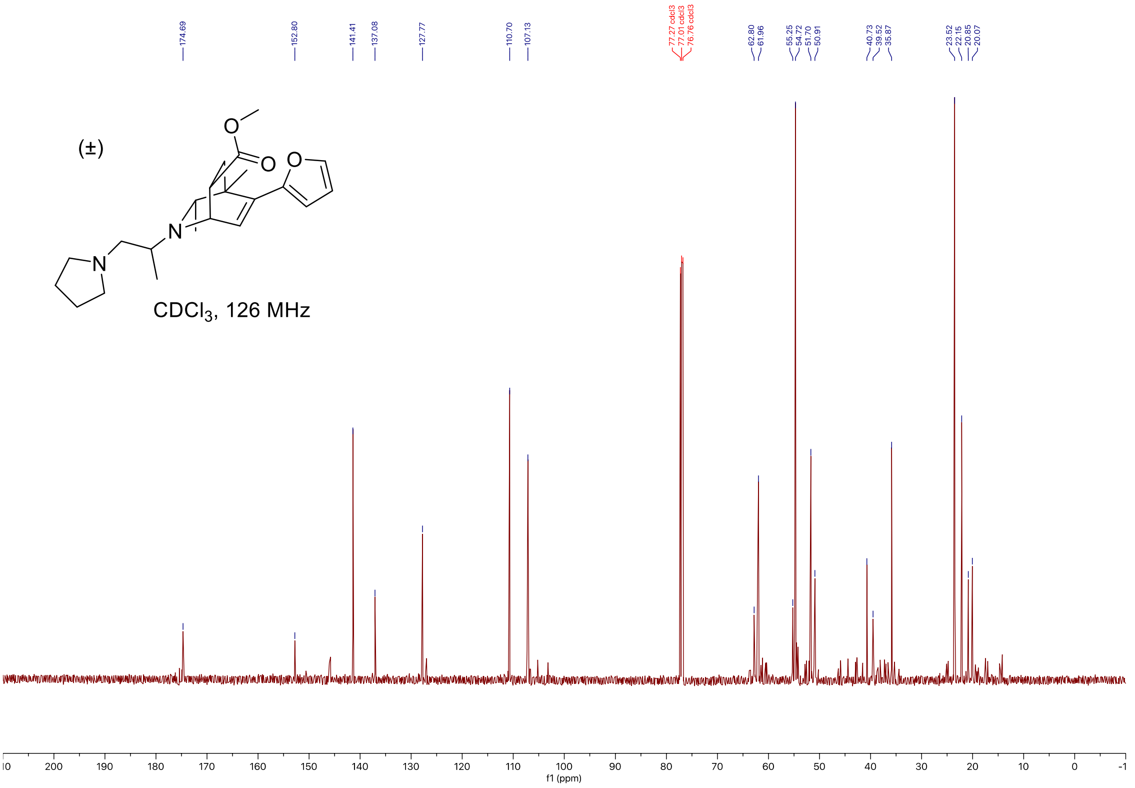

C-H COSY

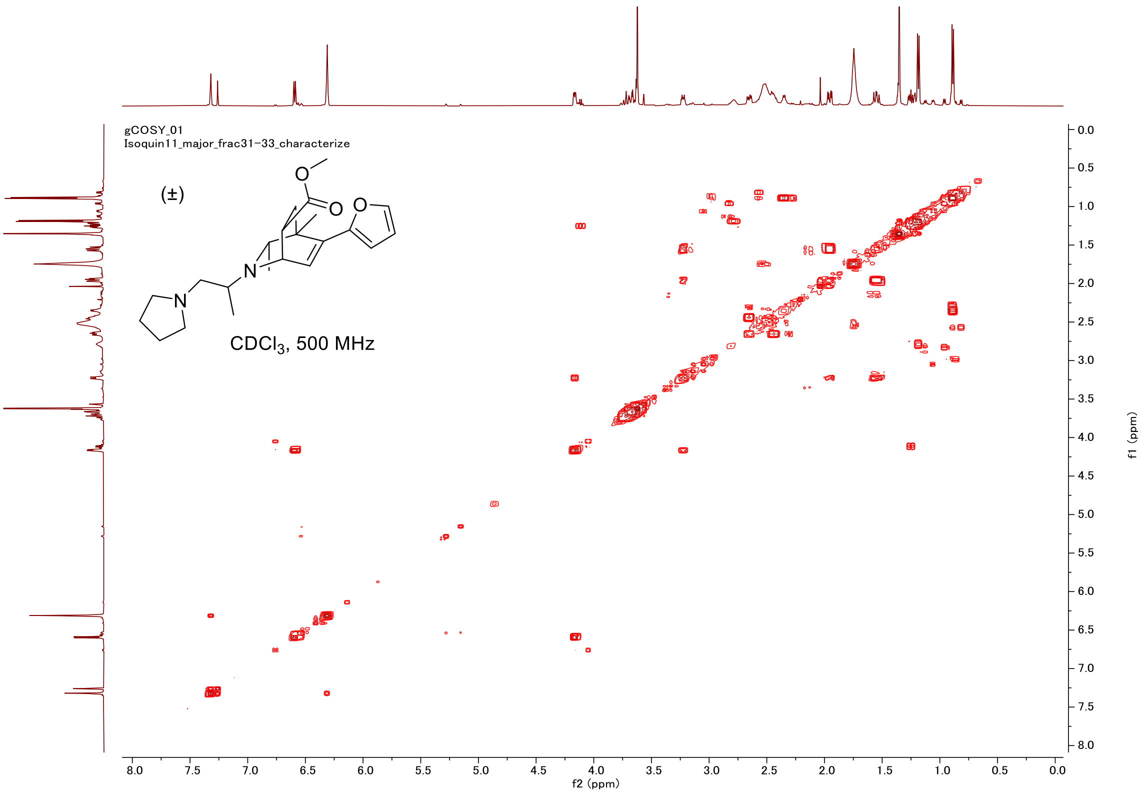
gCOSY_01
Isoquin11_major_frac31-33_characterize
(±)
CDCl$_3$, 500 MHz
f2 (ppm)
f1 (ppm)

HSQC

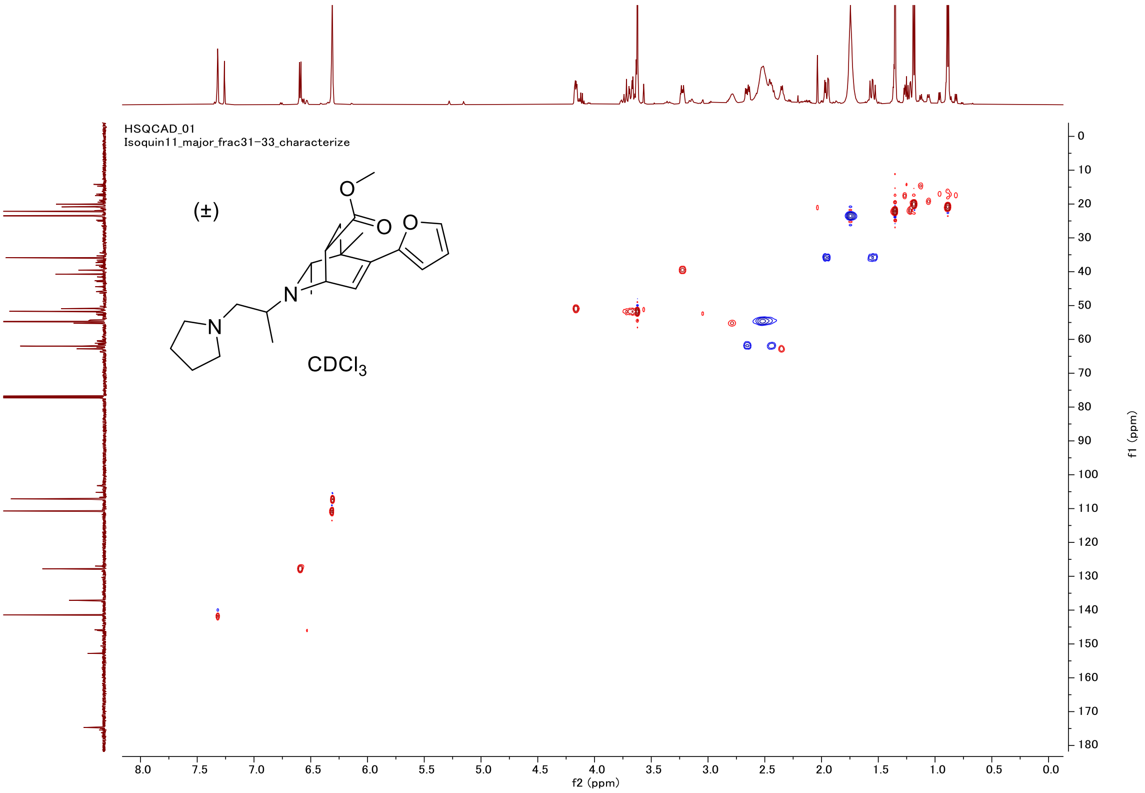
HSQCAD_01
Isoquin11_major_frac31-33_characterize
(±)
CDCl$_3$
f2 (ppm)
f1 (ppm)

Proton (#11, isomer2)

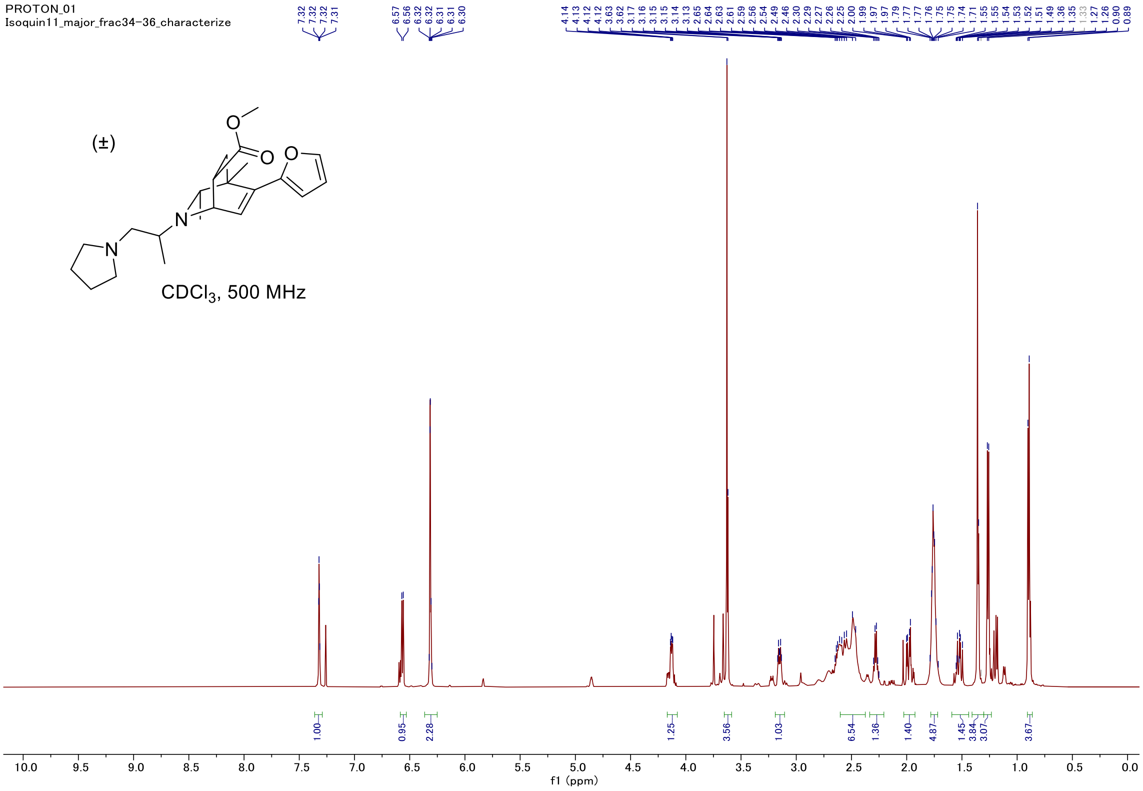

Carbon

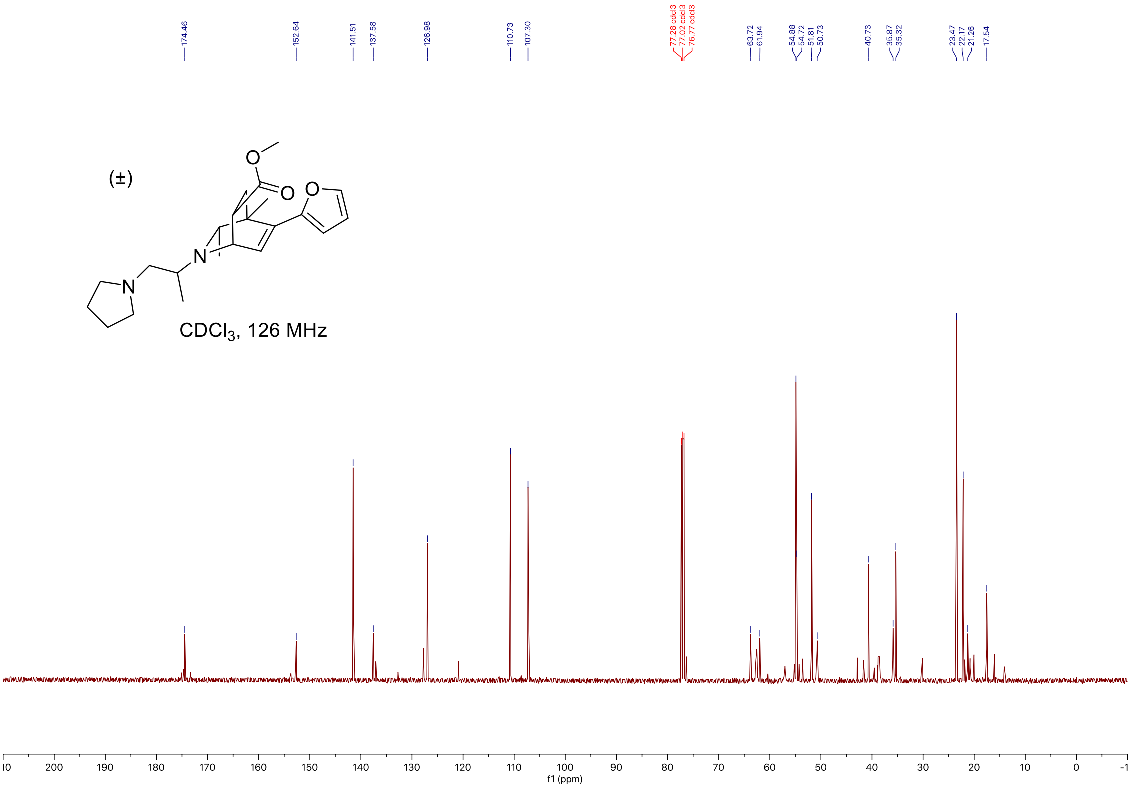

C-H COSY

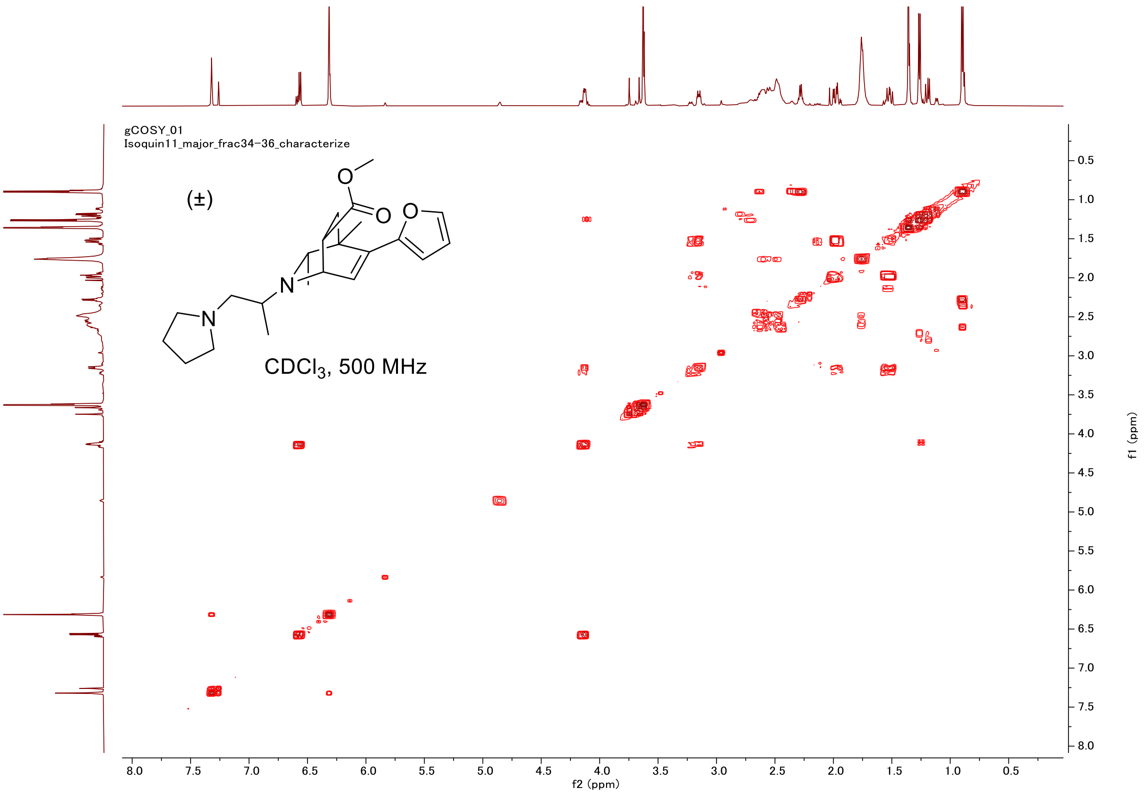
gCOSY_01
Isoquin11_major_frac34-36_characterize
(±)
CDCl$_3$, 500 MHz
f2 (ppm)
f1 (ppm)

HSQC

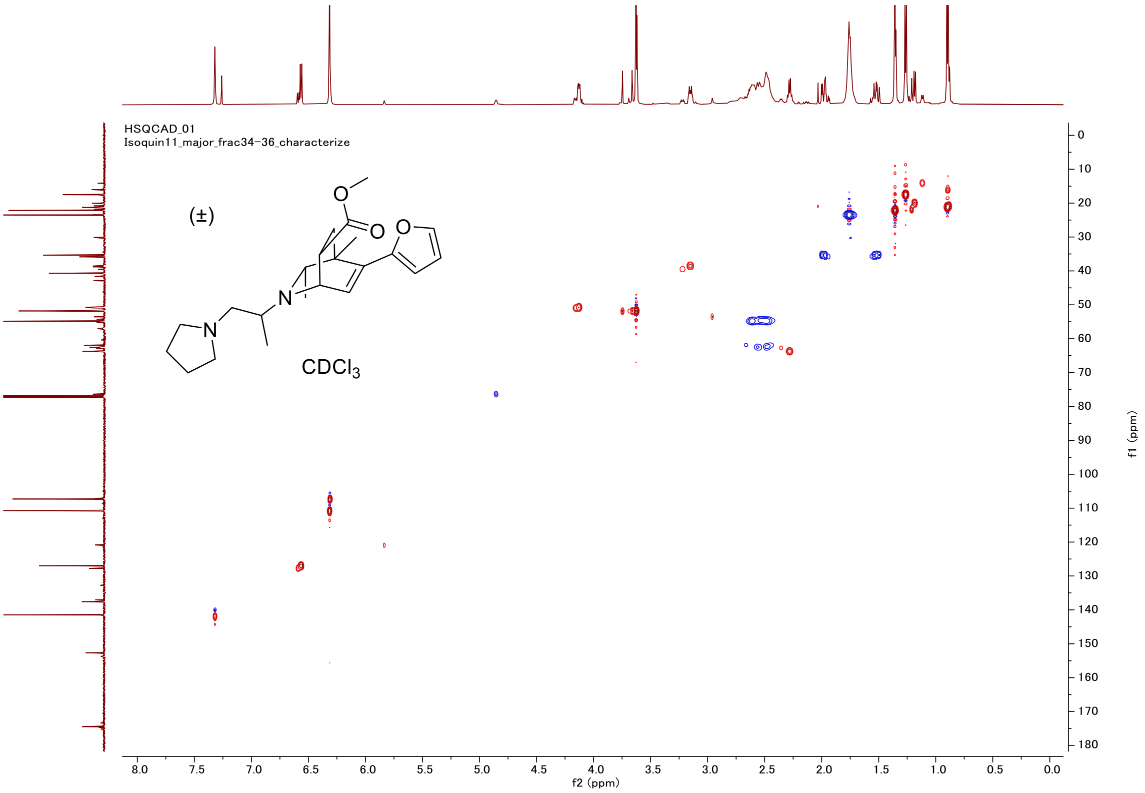
HSQCAD_01
Isoquin11_major_frac34-36_characterize
(±)
CDCl$_3$
f2 (ppm)
f1 (ppm)

Proton (#11, isomer 3)

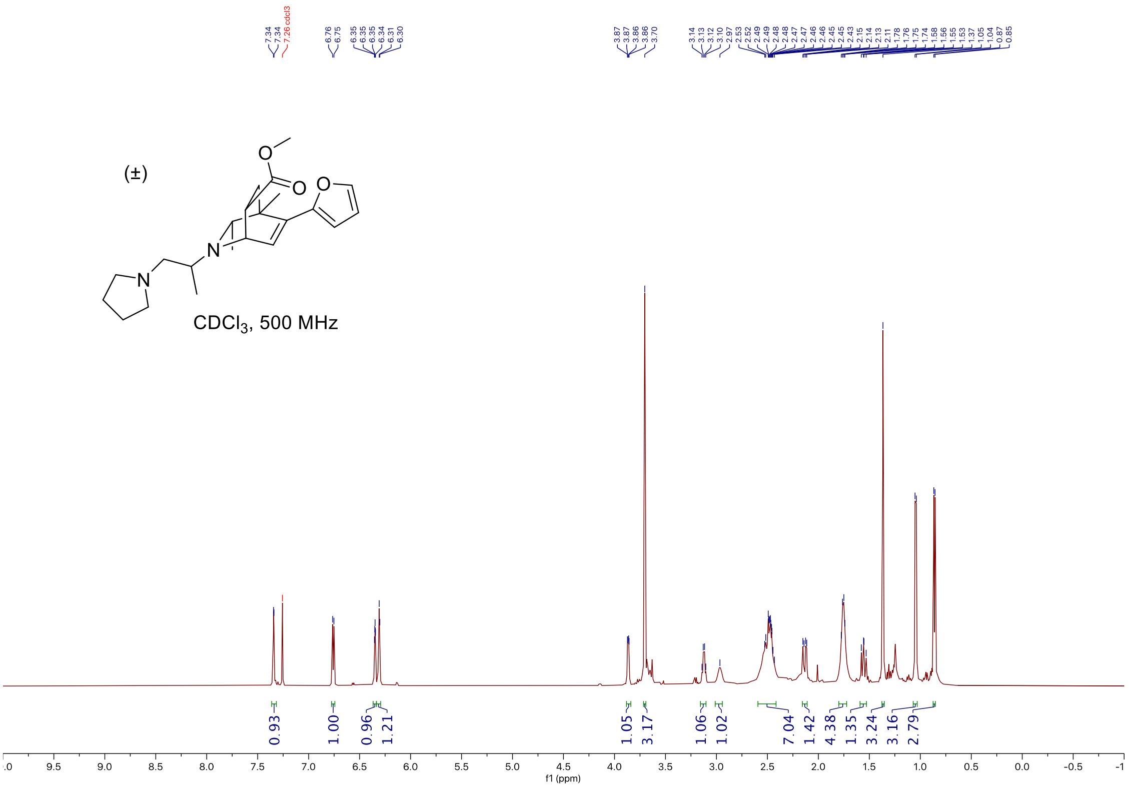

Carbon

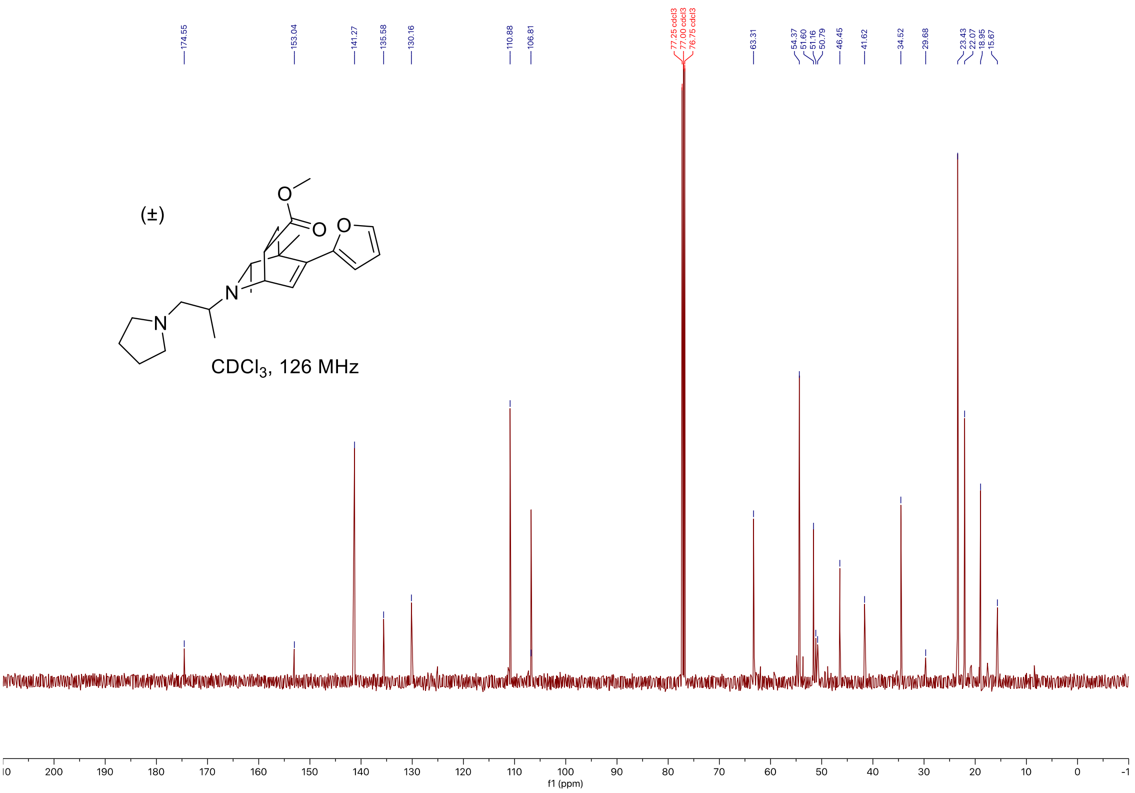

C-H COSY

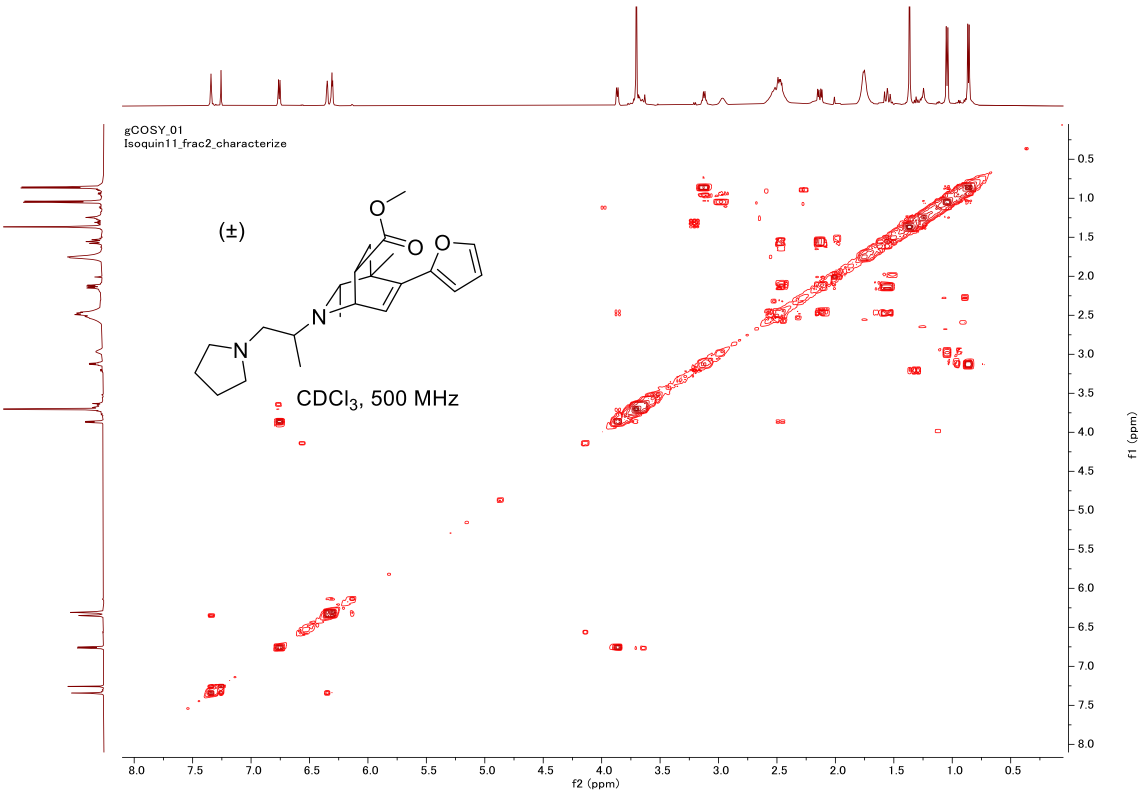
gCOSY_01
Isoquin11_frac2_characterize
(±)
CDCl$_3$, 500 MHz
f2 (ppm)
f1 (ppm)

HSQC

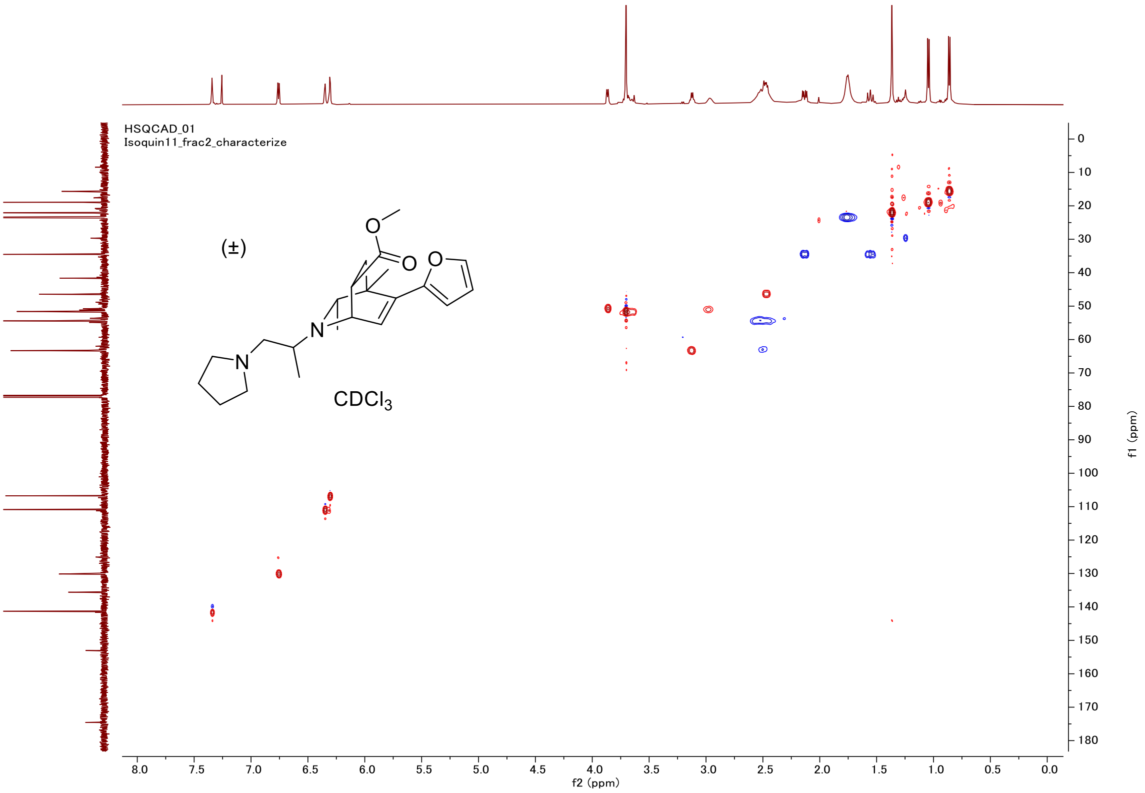
HSQCAD_01
Isoquin11_frac2_characterize
(±)
CDCl$_3$
f2 (ppm)
f1 (ppm)

Proton (#12)

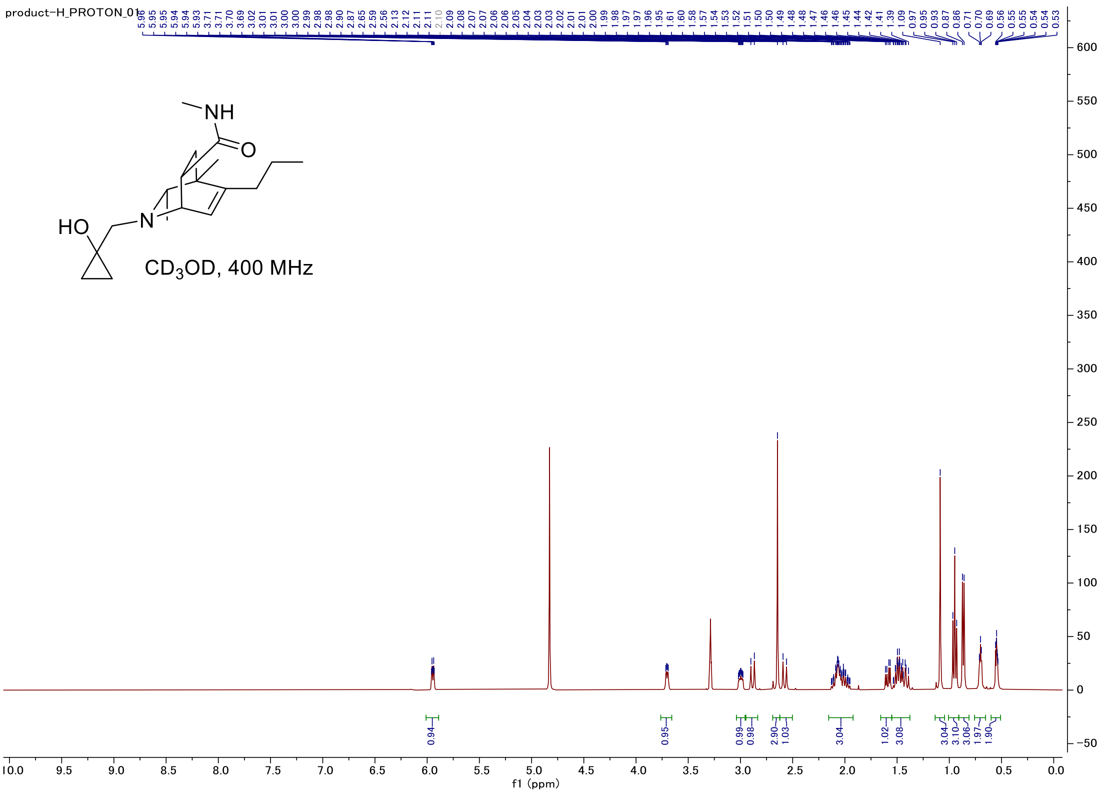

Carbon

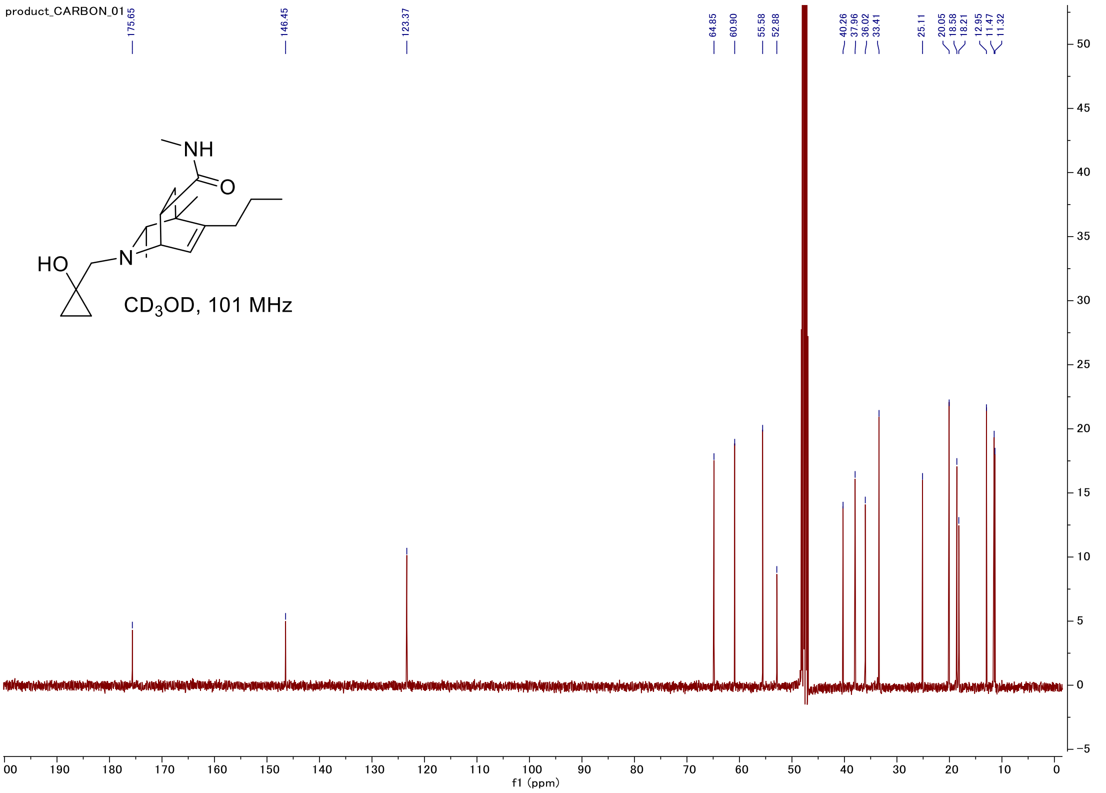

C-H COSY

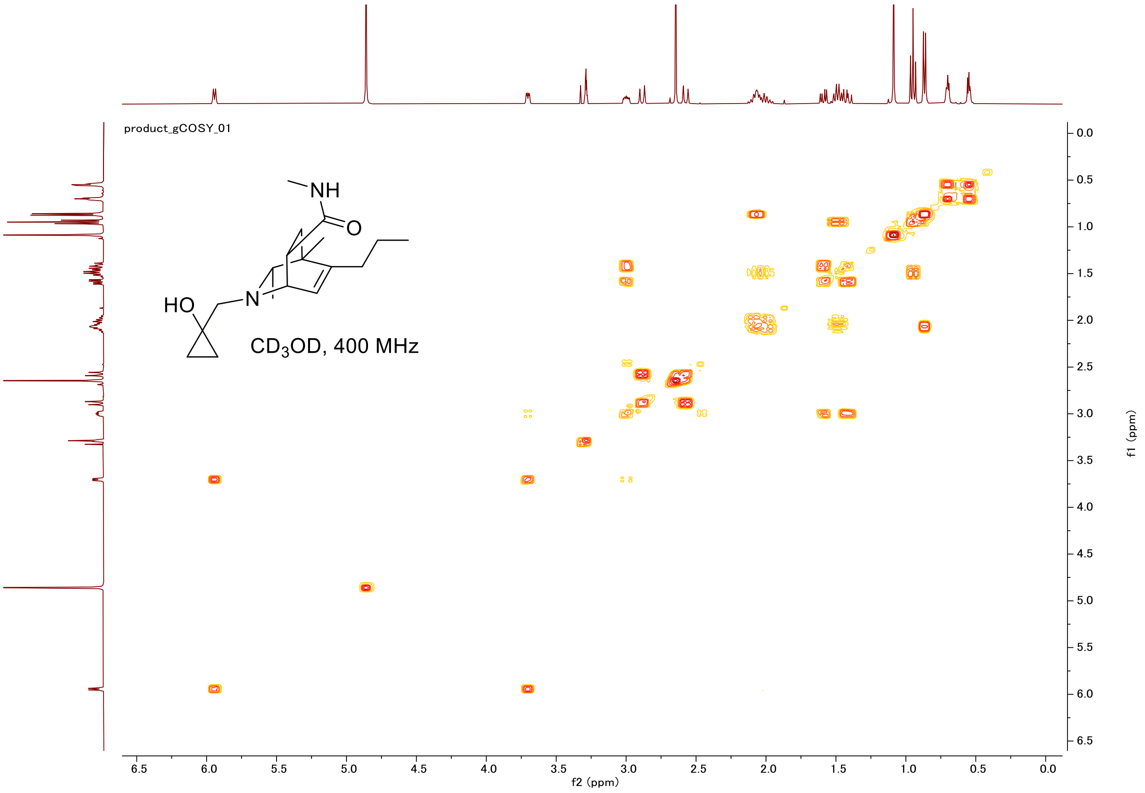

product_gCOSY_01
NH
O
HO
N
CD$_3$OD, 400 MHz
f2 (ppm)
f1 (ppm)

HSQC

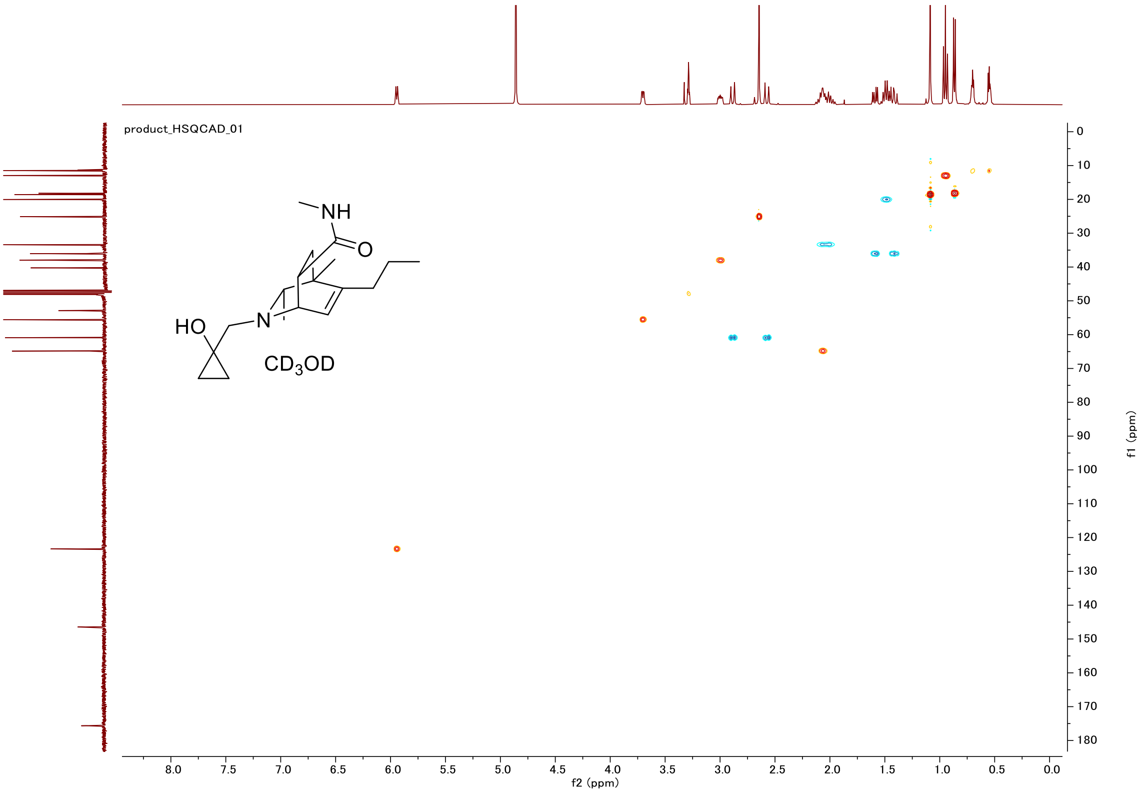

product_HSQCAD_01
NH
O
HO
N
CD$_3$OD
f2 (ppm)
f1 (ppm)

Proton (#14)

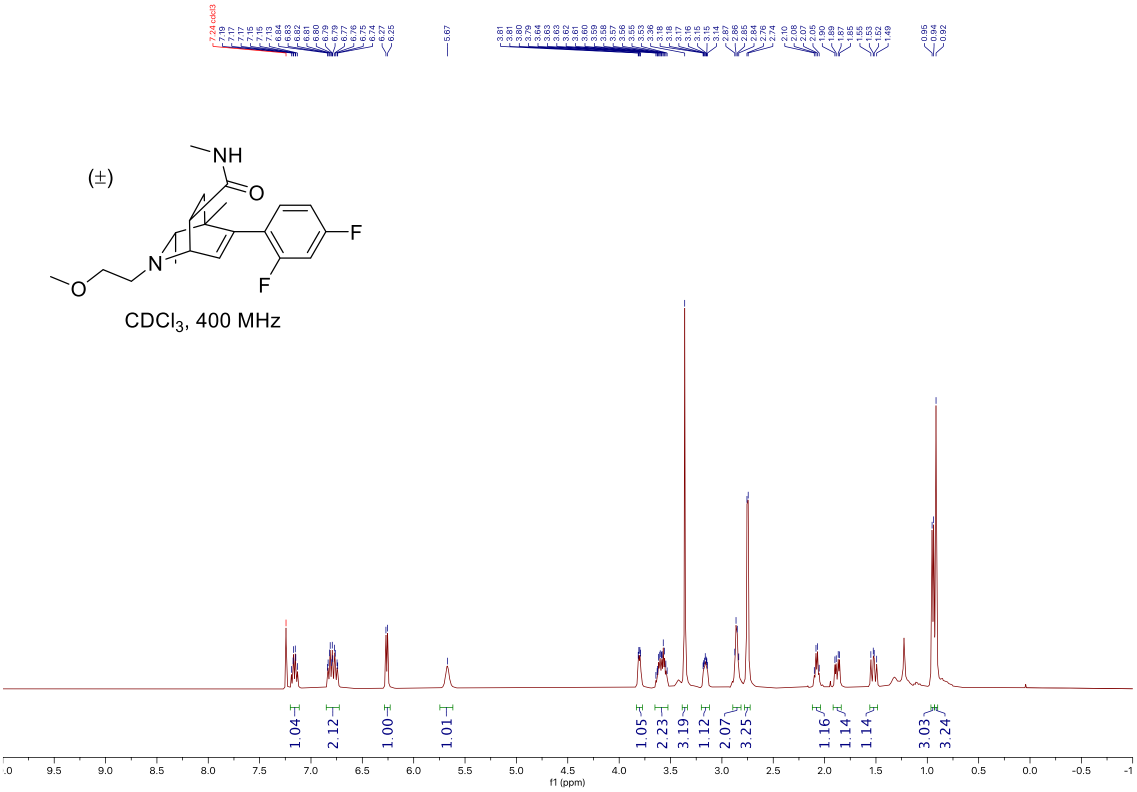

Carbon

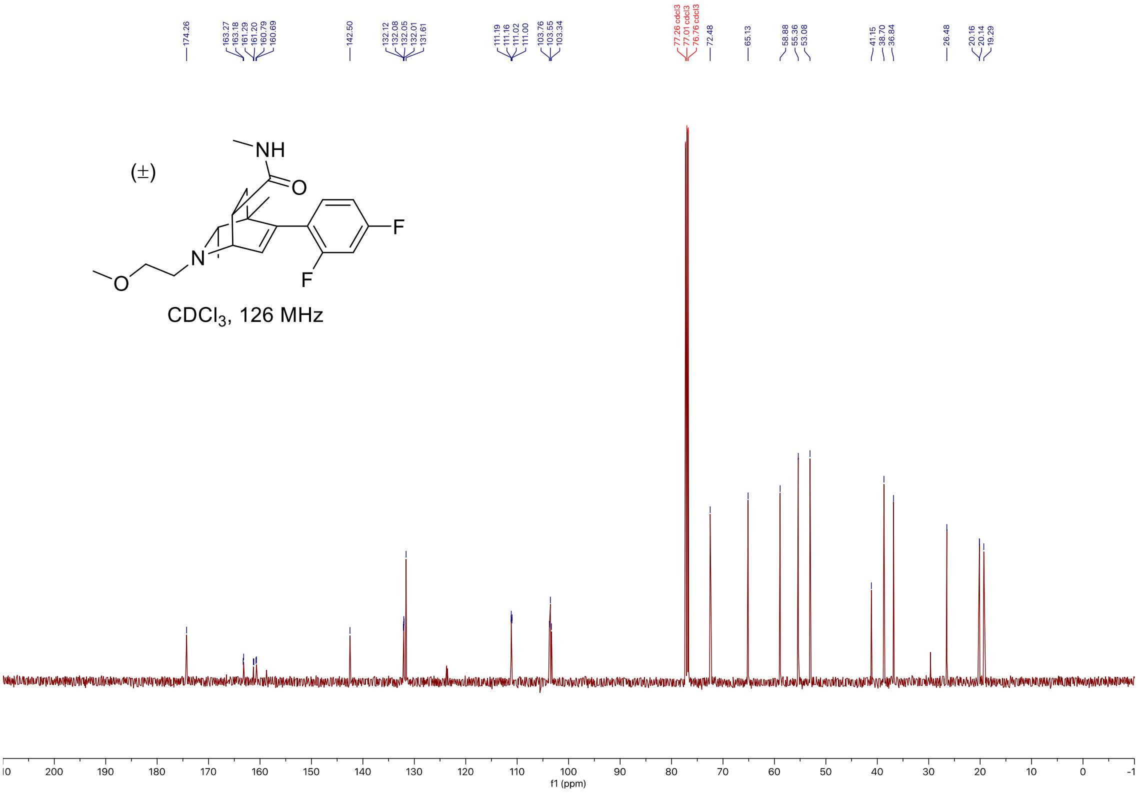

Fluorine

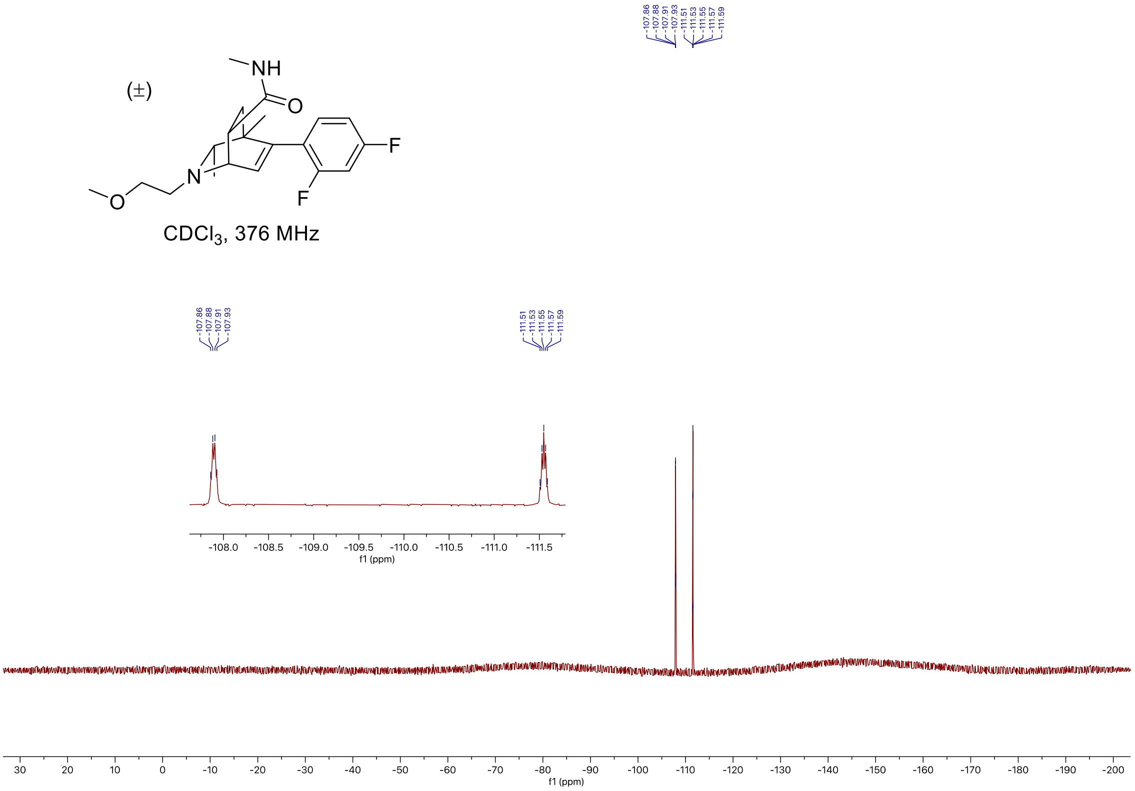

C-H COSY

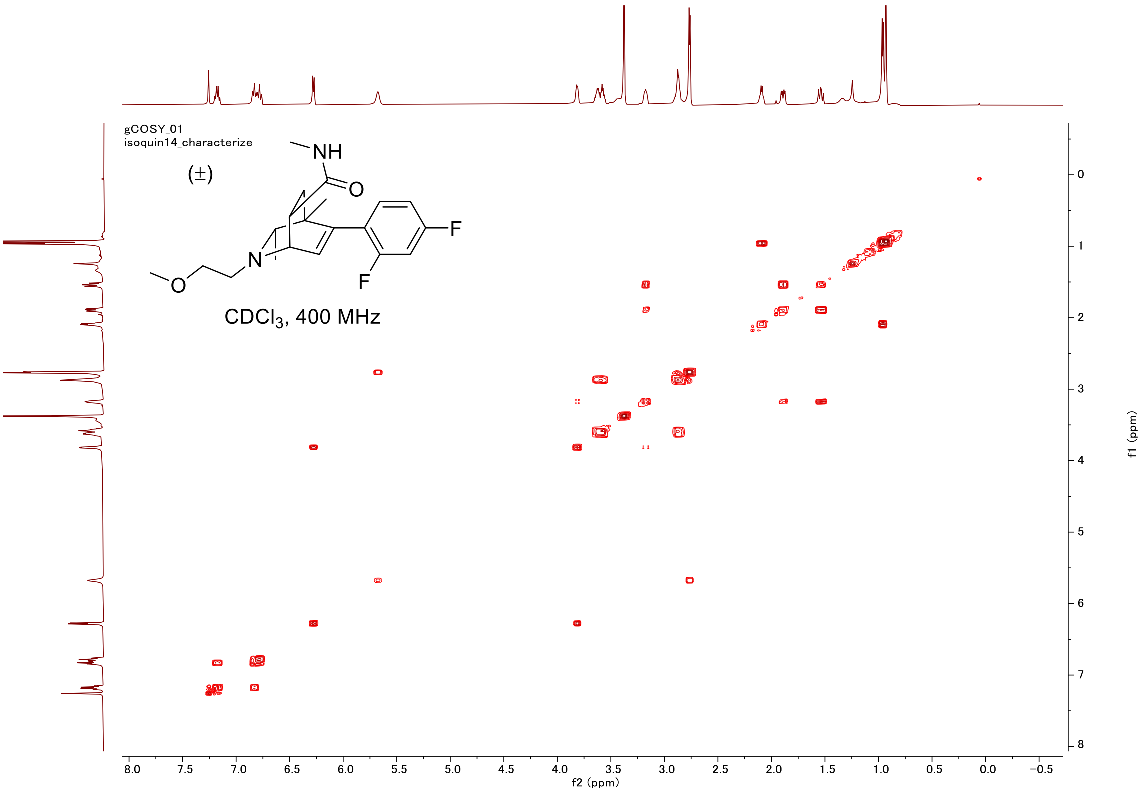

HSQC

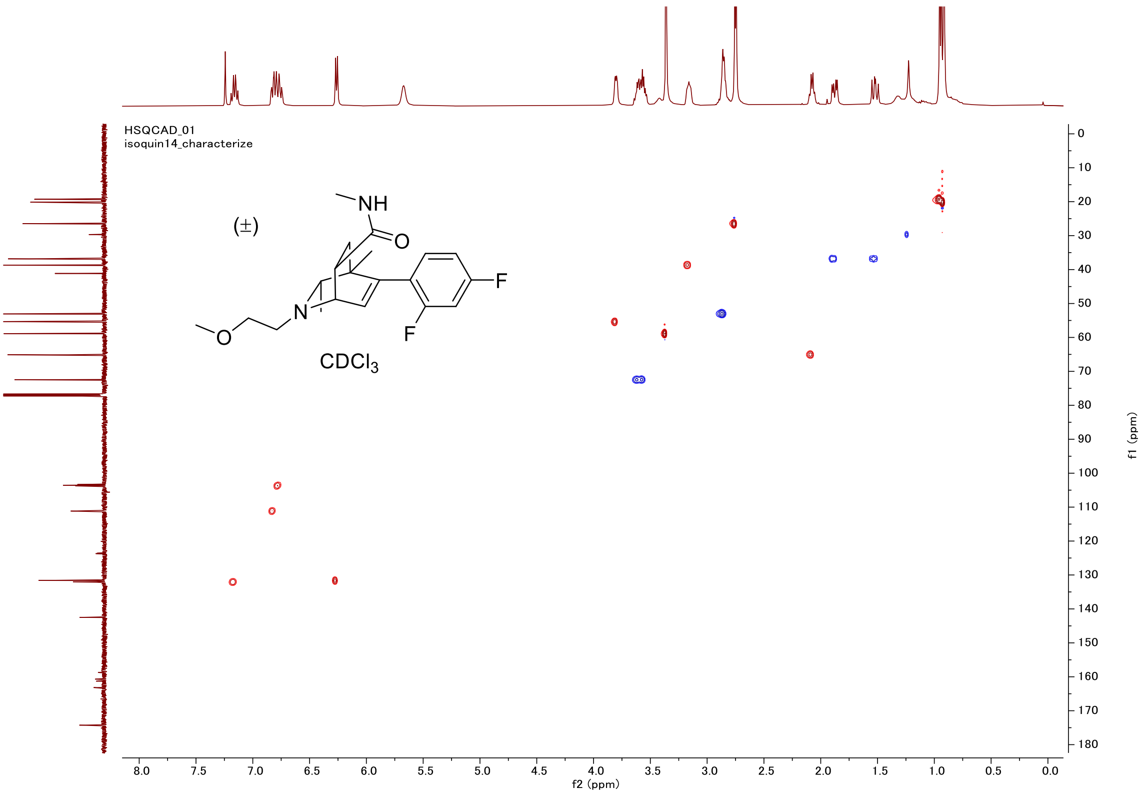
HSQCAD_01
isoquin14_characterize
(±)
NH
O
F
N
O
F
CDCl3
f2 (ppm)
f1 (ppm)

Proton (#17)

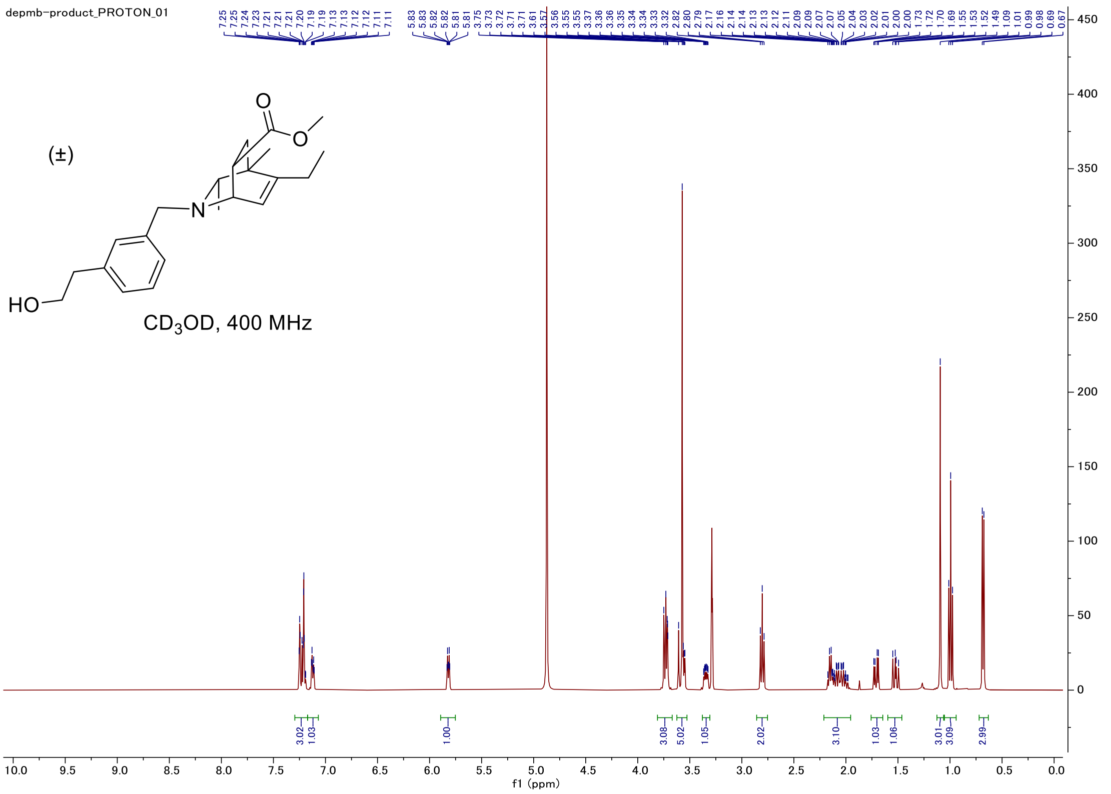

Carbon

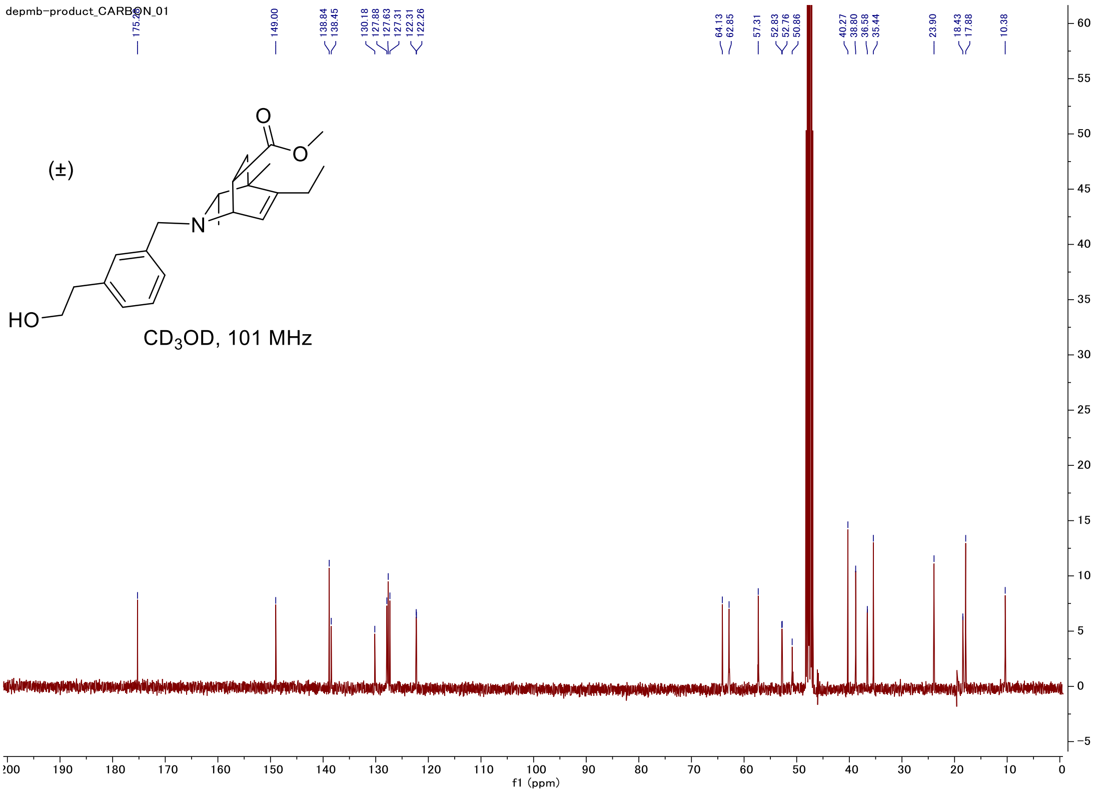

C-H COSY

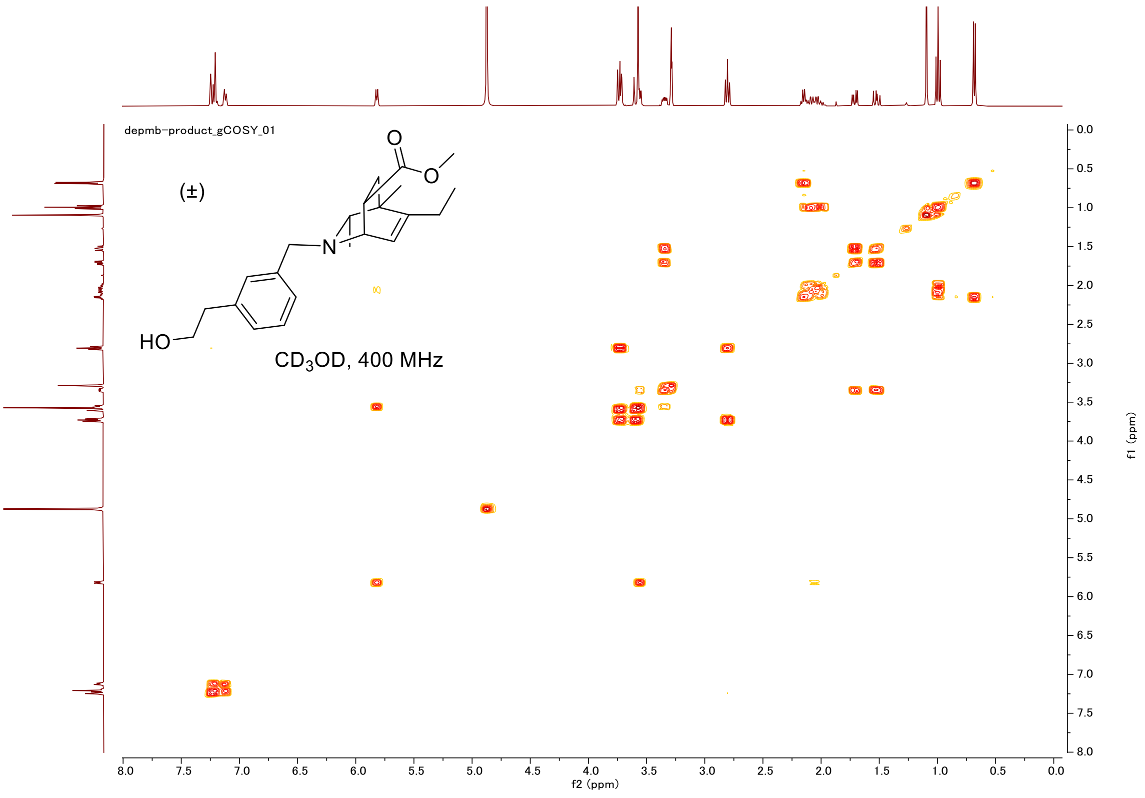
depmb-product_gCOSY_01
(±)
HO
N
O
O
CD3OD, 400 MHz
f2 (ppm)
f1 (ppm)

HSQC

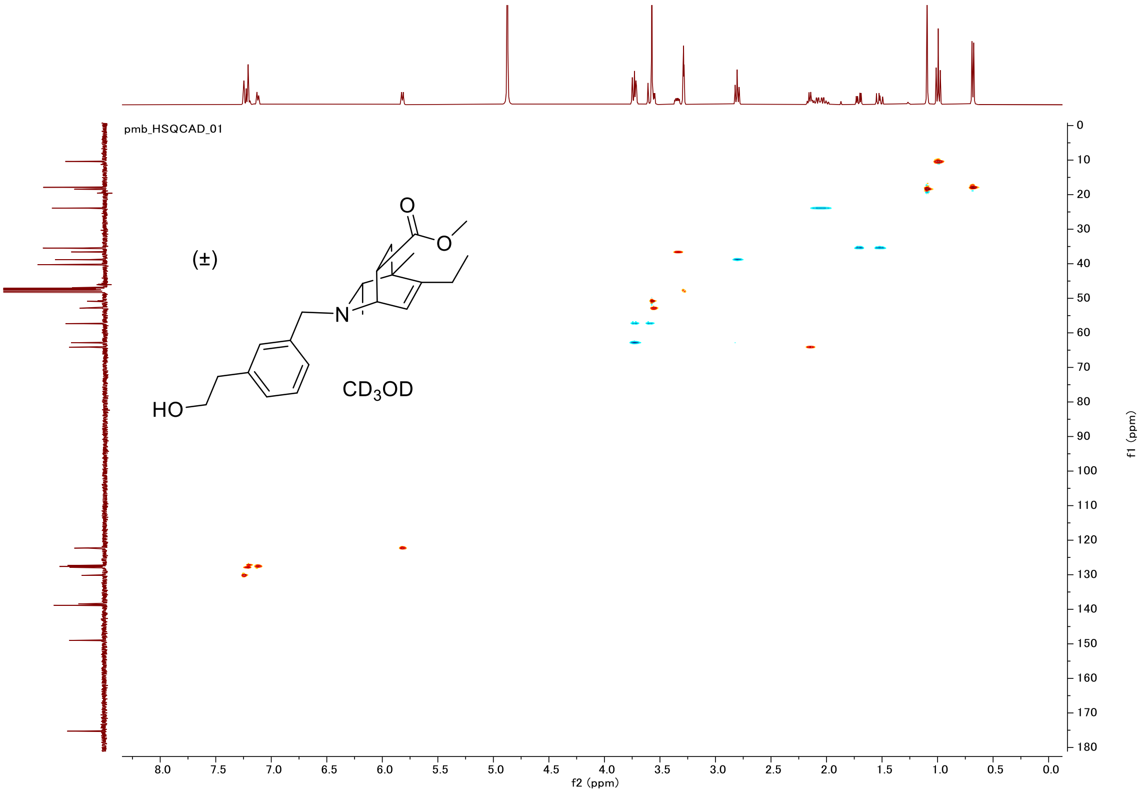
pmb_HSQCAD_01
(±)
HO
N
O
O
CD3OD
f2 (ppm)
f1 (ppm)

Proton (#19-major diastereomer)

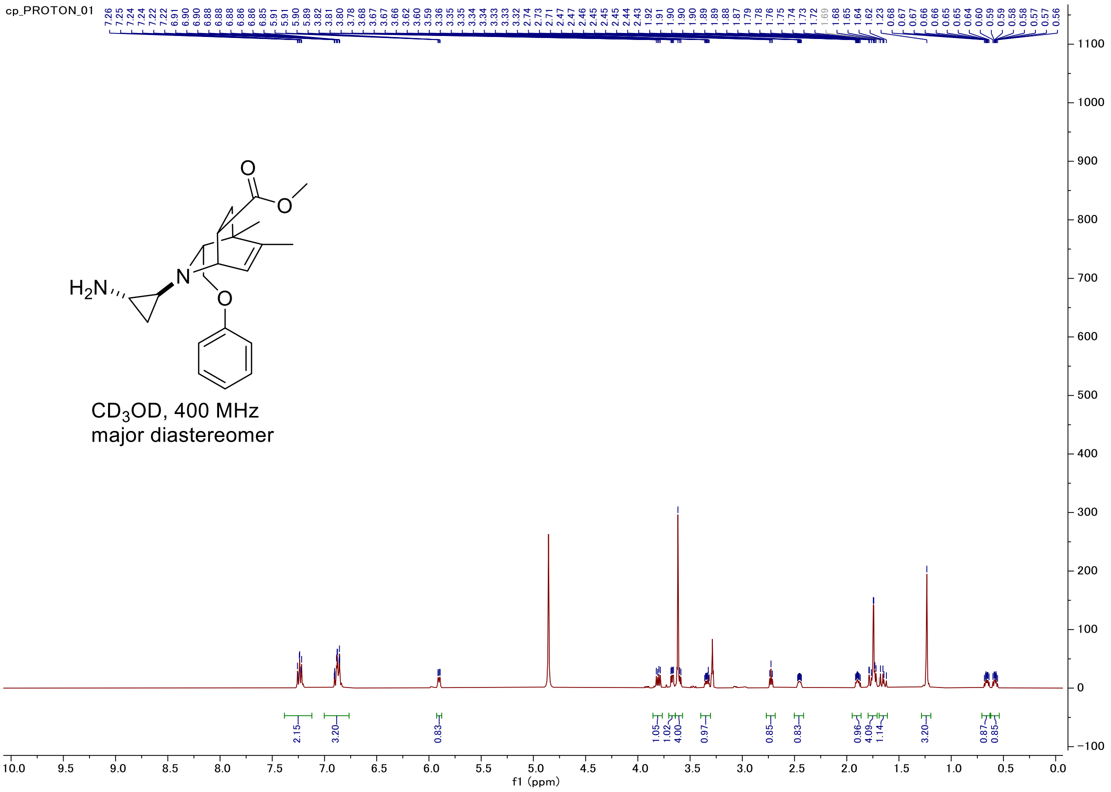

Carbon

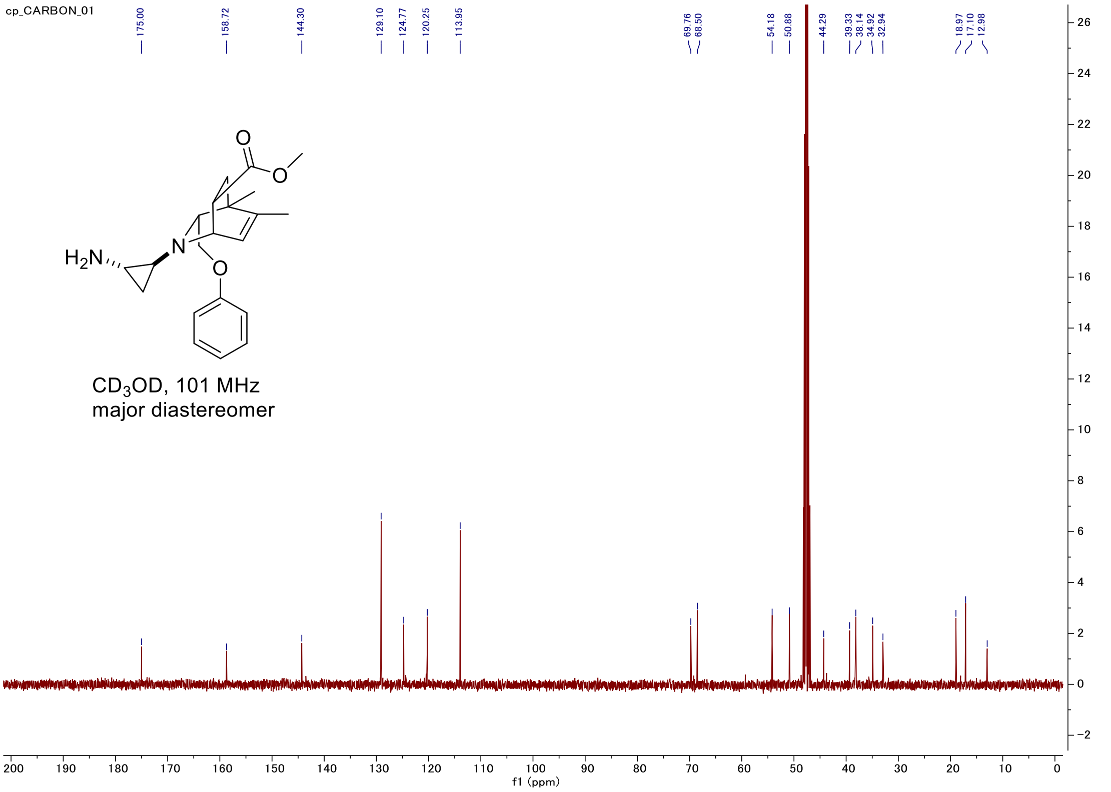

C-H COSY

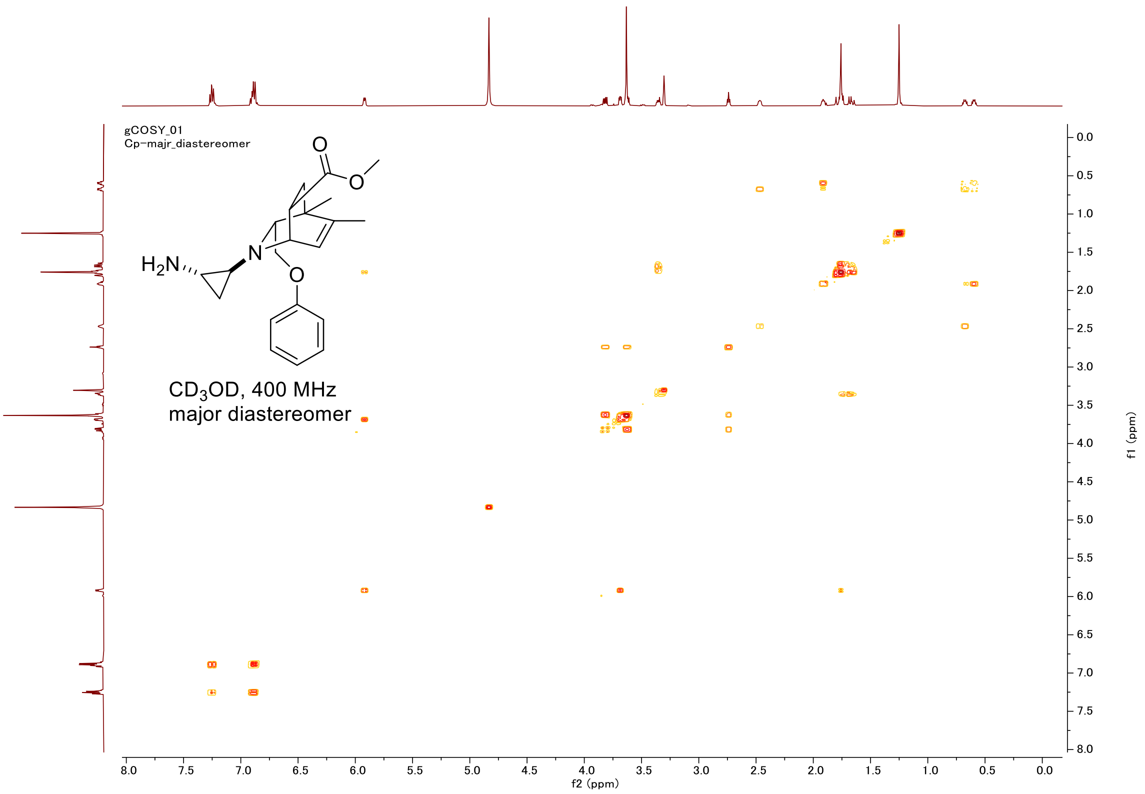
gCOSY_01
Cp−majr_diastereomer
H2N
N
O
O
O
CD3OD, 400 MHz
major diastereomer
f2 (ppm)
f1 (ppm)

HSQC

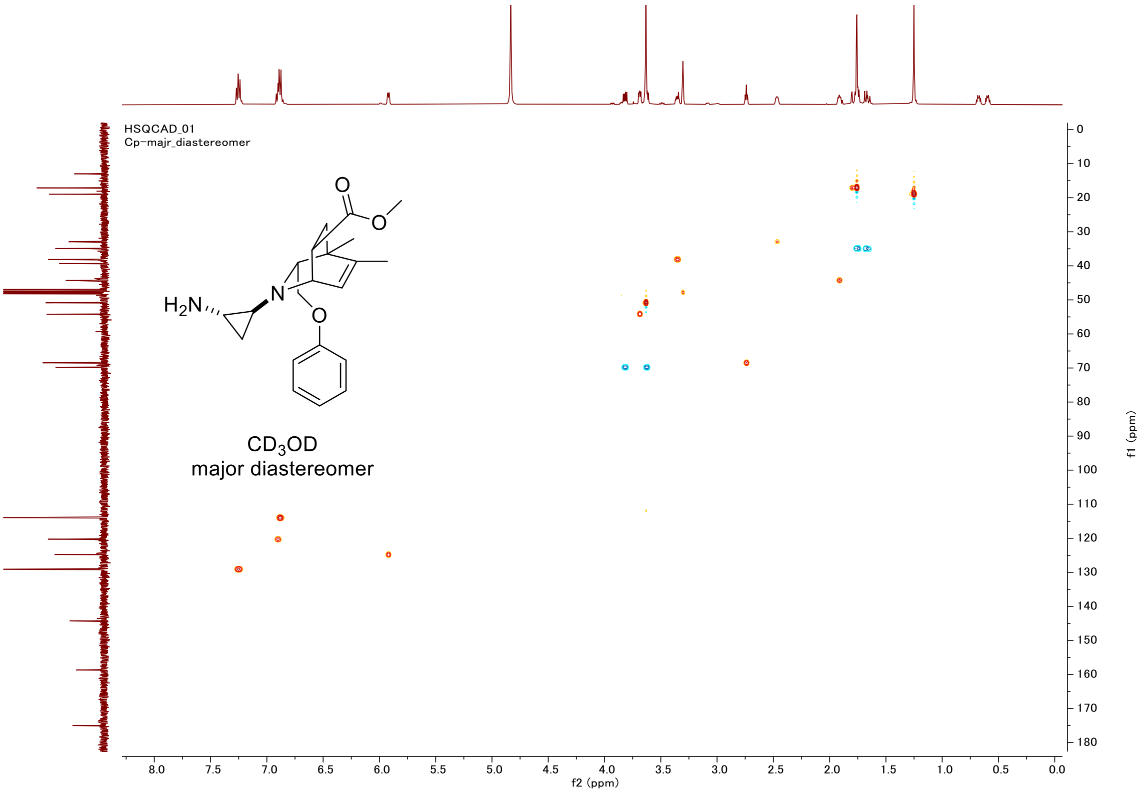
HSQCAD_01
Cp−majr_diastereomer
H2N
N
O
O
O
CD3OD
major diastereomer
f2 (ppm)
f1 (ppm)

Proton (#19-minor diastereomer)

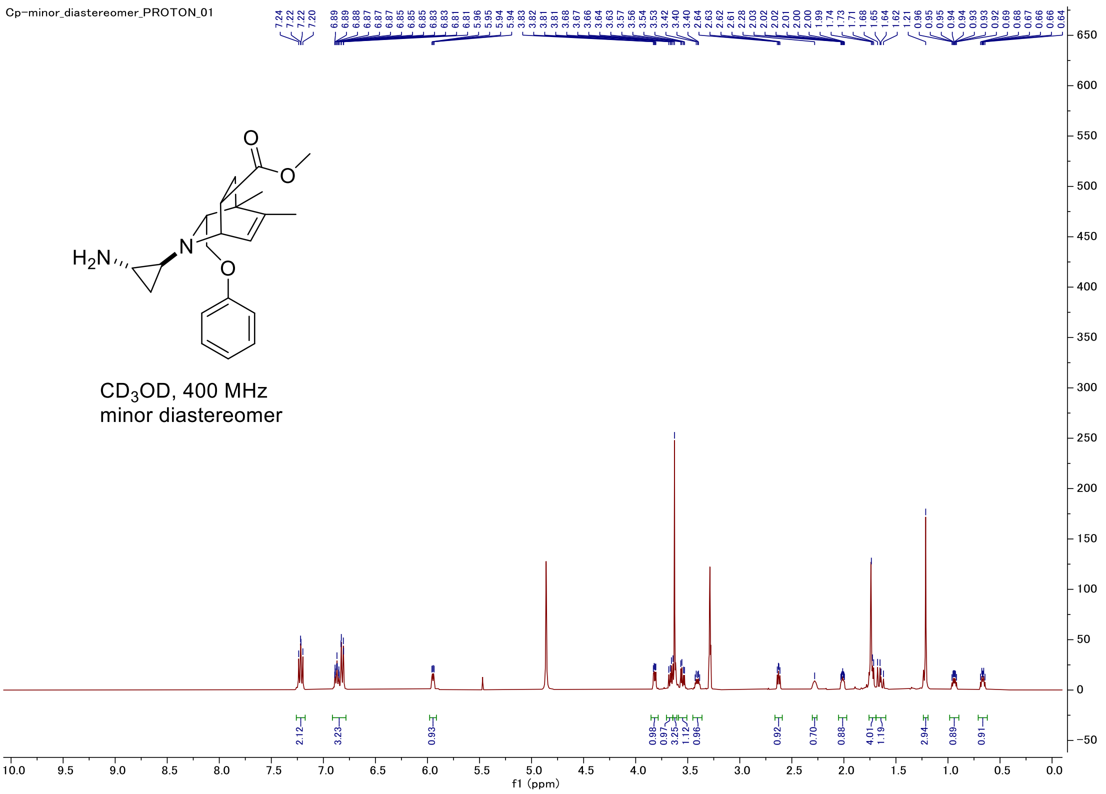

Carbon

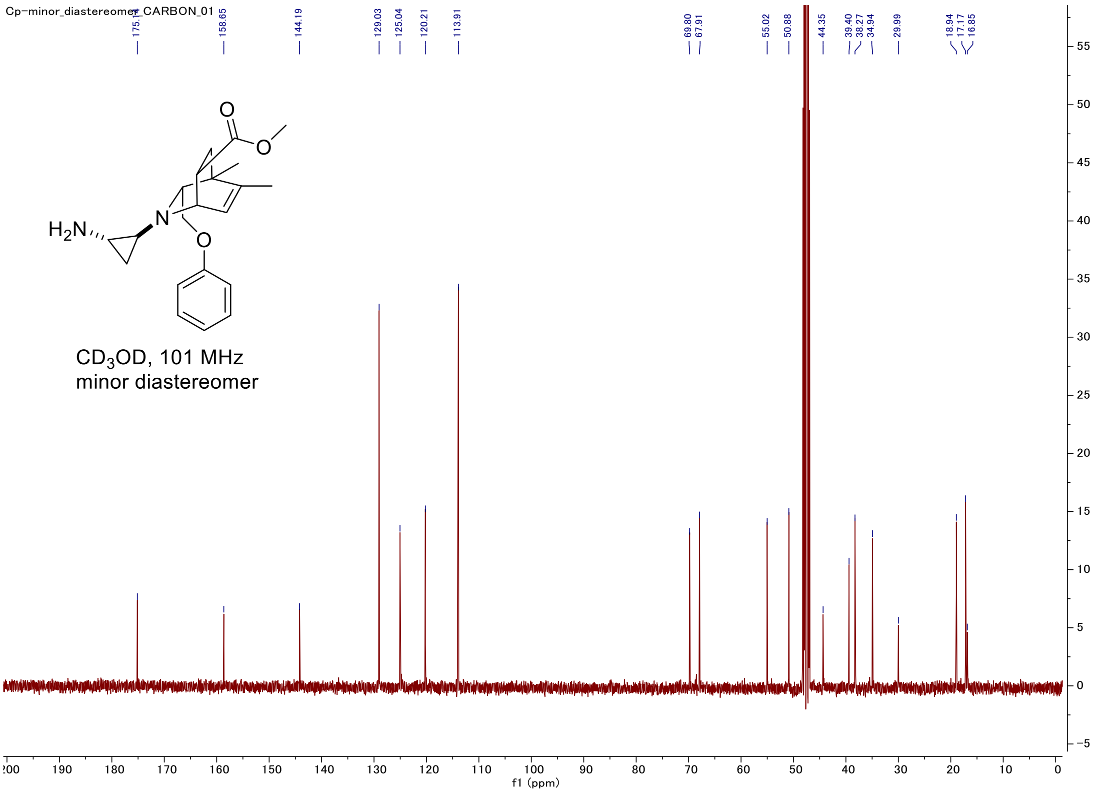

C-H COSY

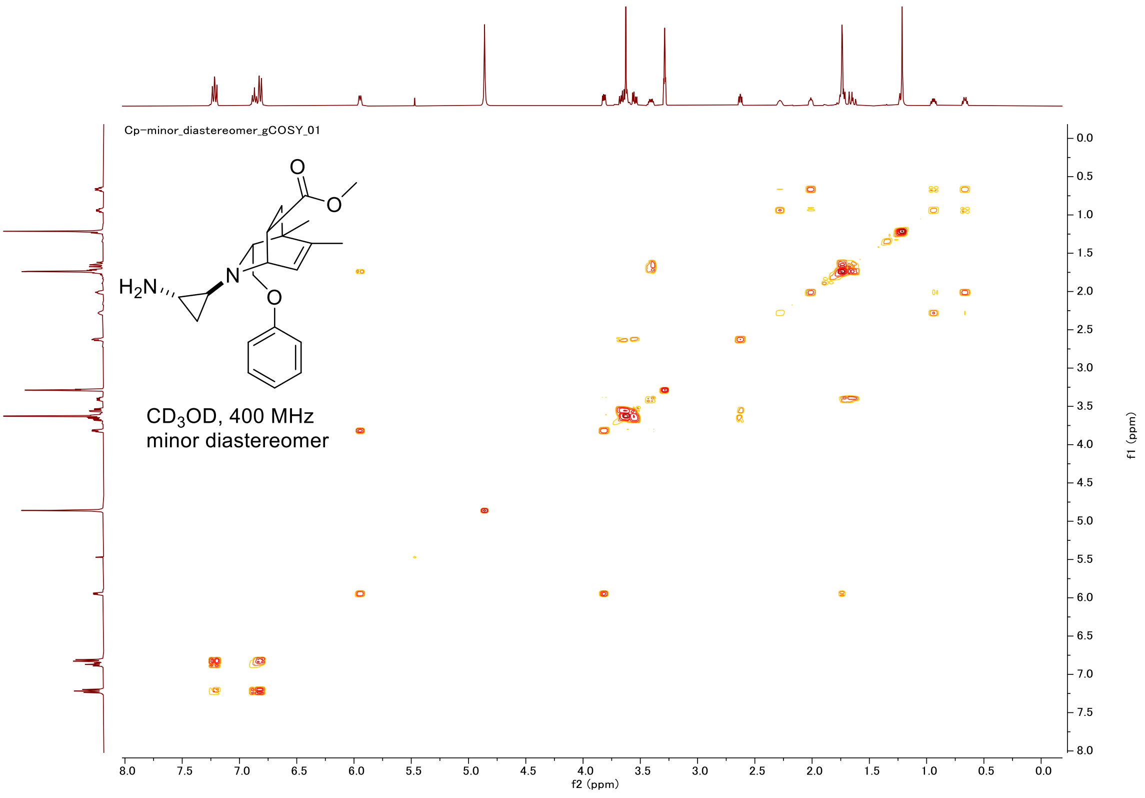
Cp-minor_diastereomer_gCOSY_01
CD3OD, 400 MHz
minor diastereomer
f2 (ppm)
f1 (ppm)

HSQC

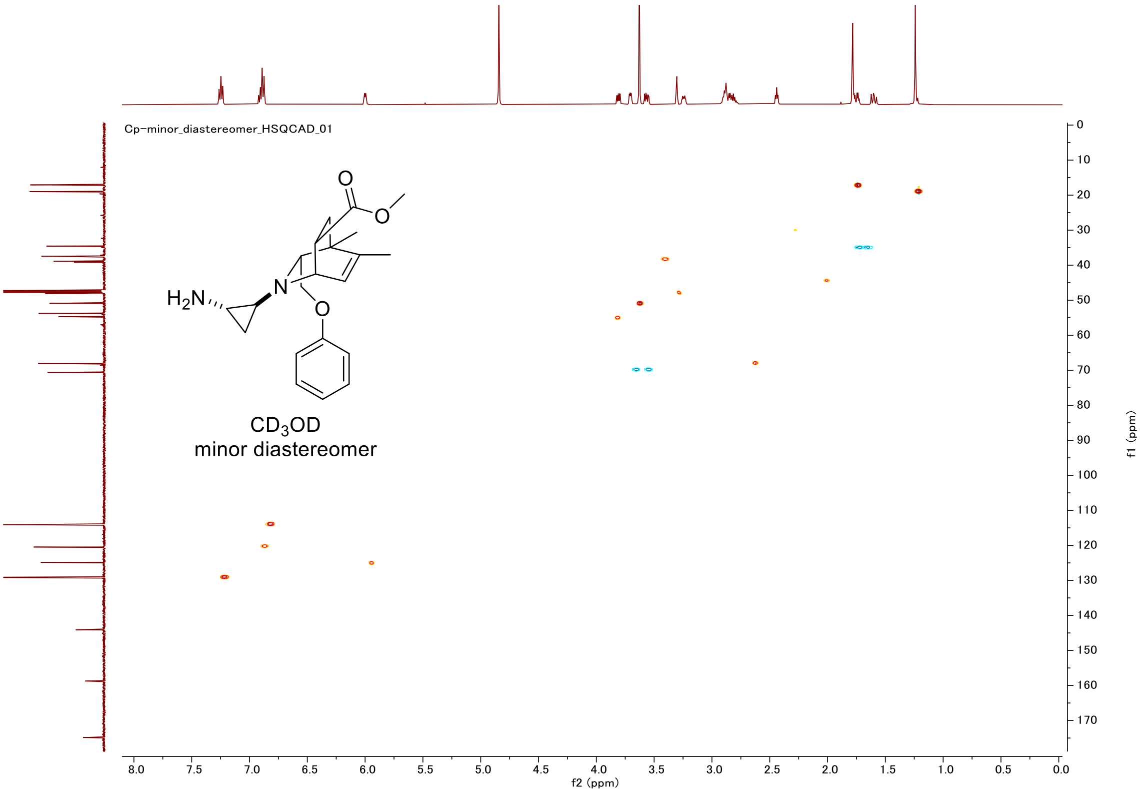
Cp-minor_diastereomer_HSQCAD_01
CD3OD
minor diastereomer
f2 (ppm)
f1 (ppm)

Proton (#21)

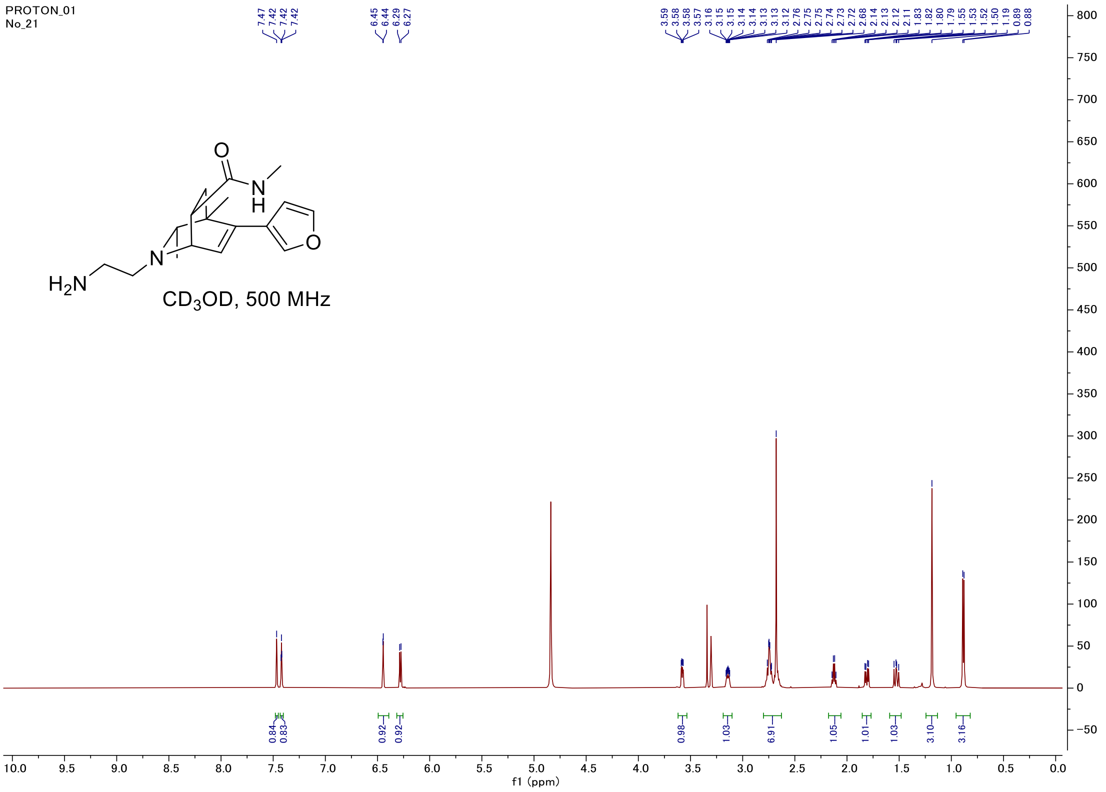

Carbon

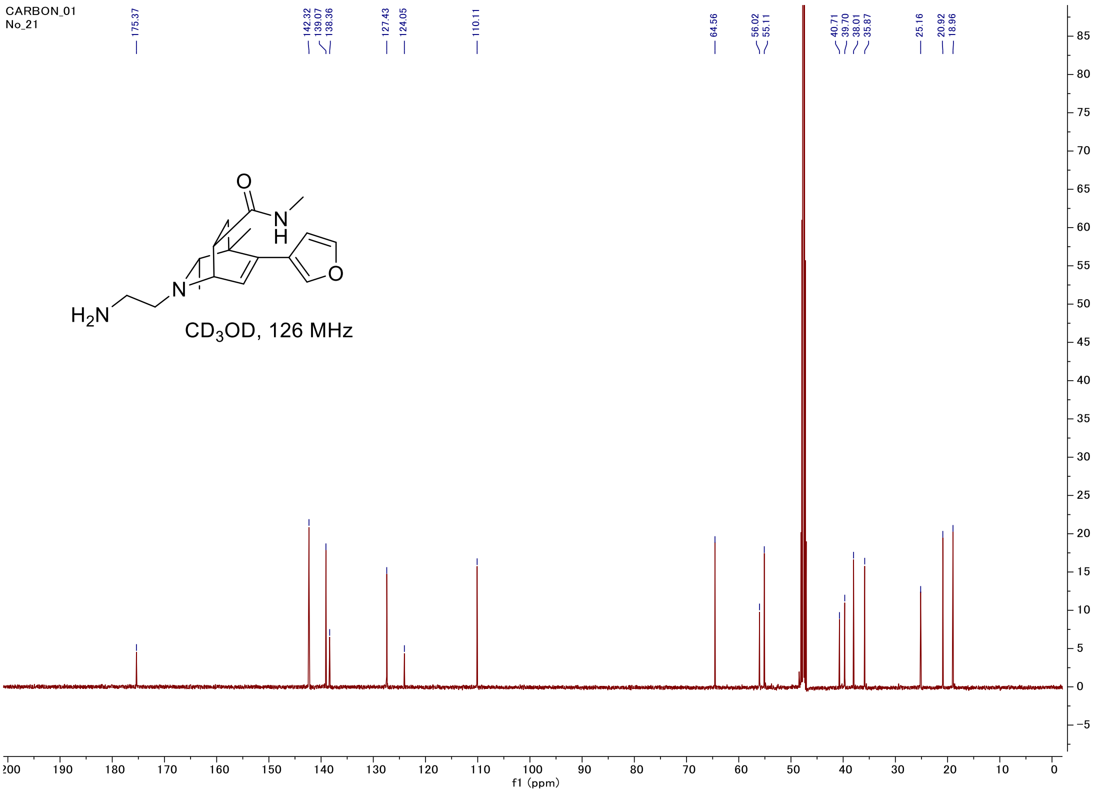

C-H COSY

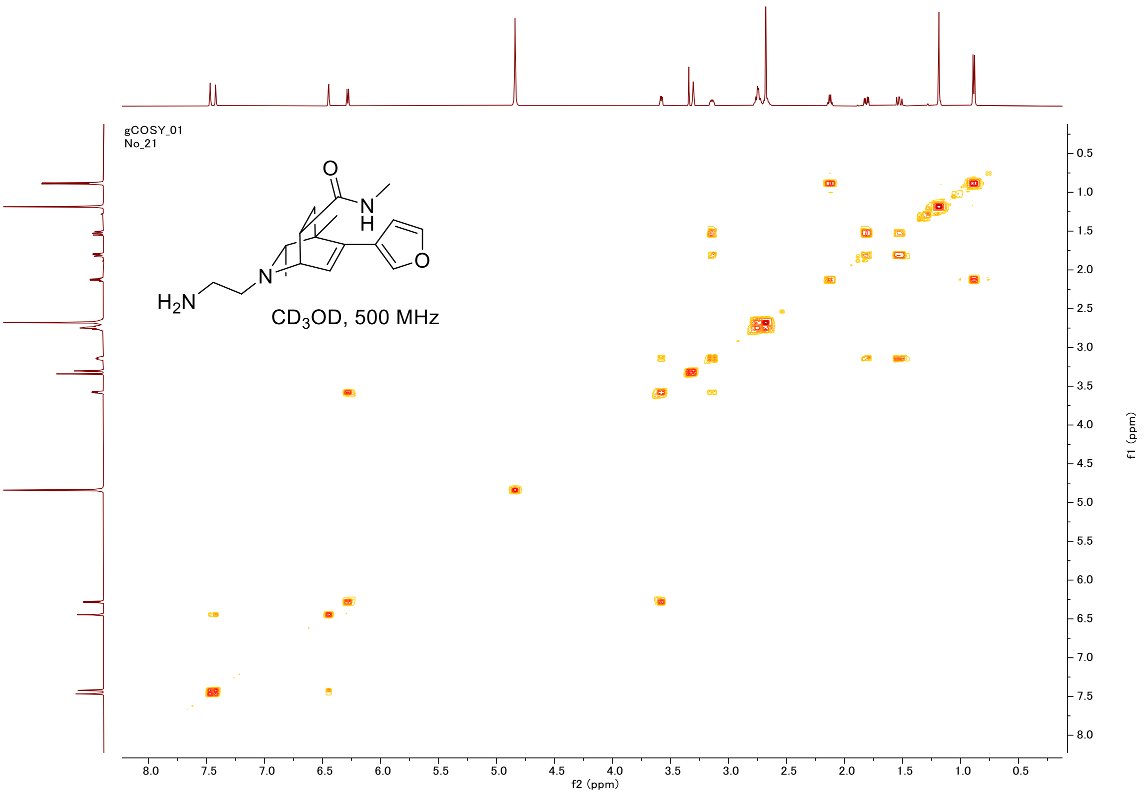
gCOSY_01
No_21
CD3OD, 500 MHz
f2 (ppm)
f1 (ppm)

HSQC

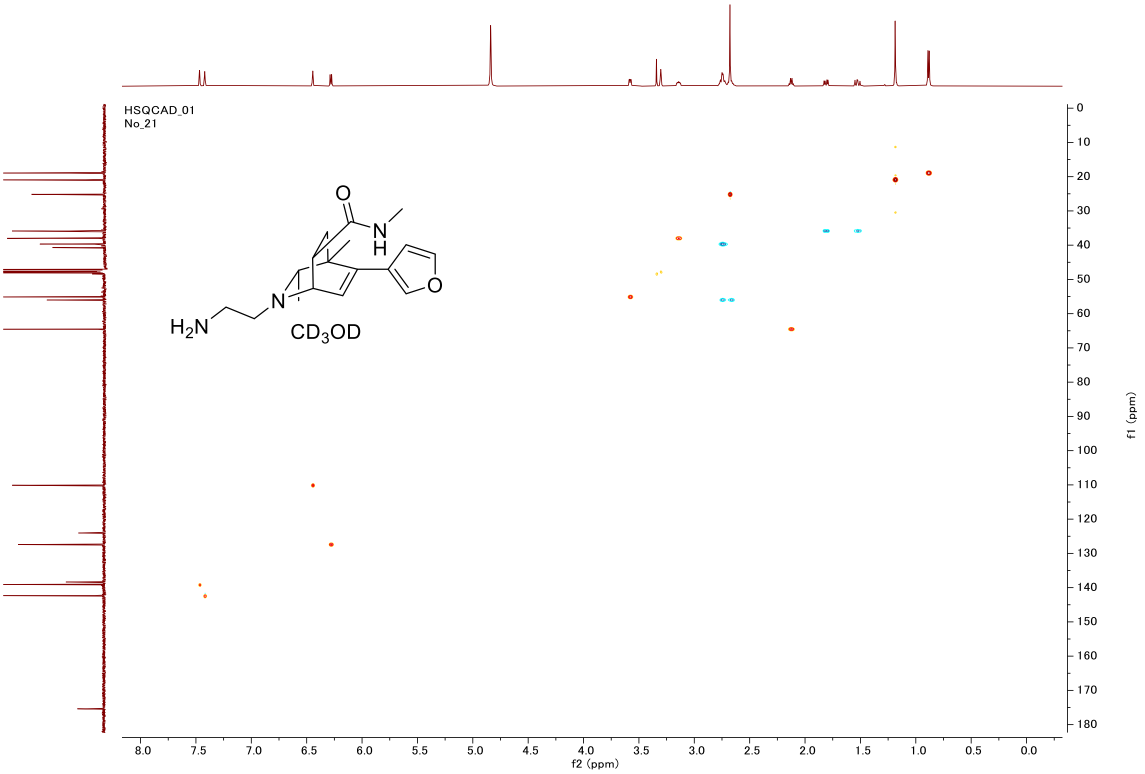
HSQCAD_01
No_21
CD3OD
f2 (ppm)
f1 (ppm)

Proton (#33)

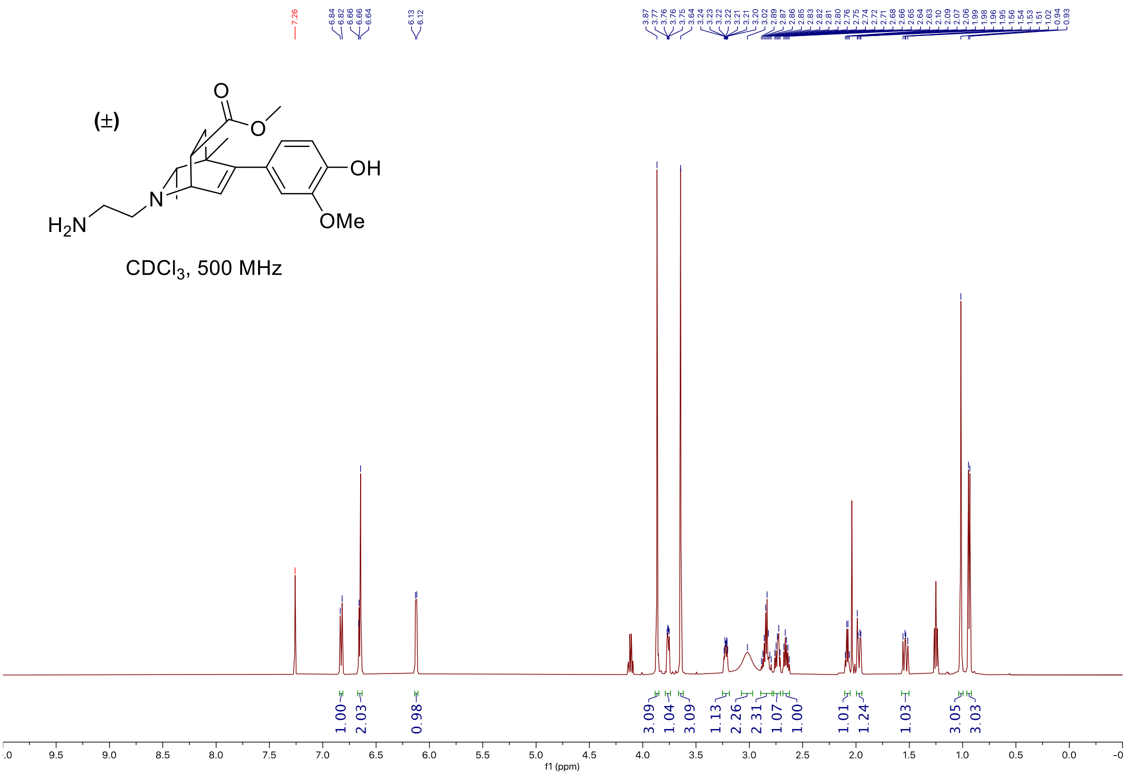

Carbon

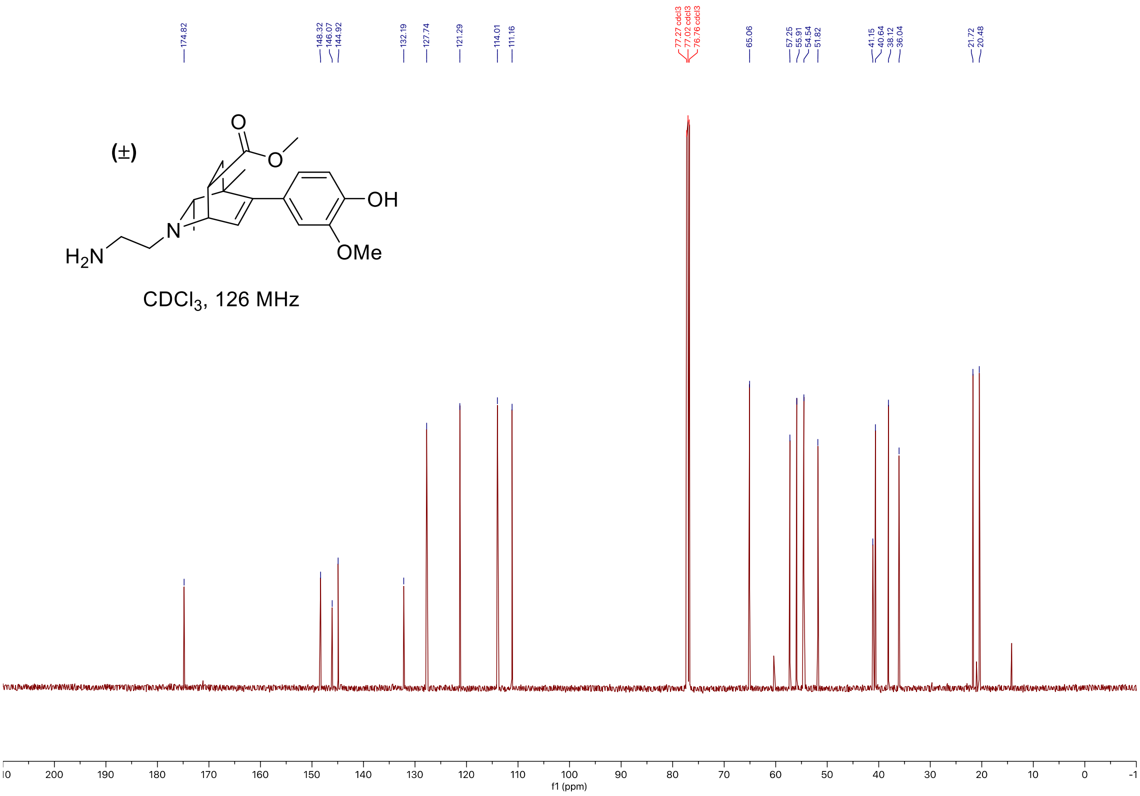

C-H COSY

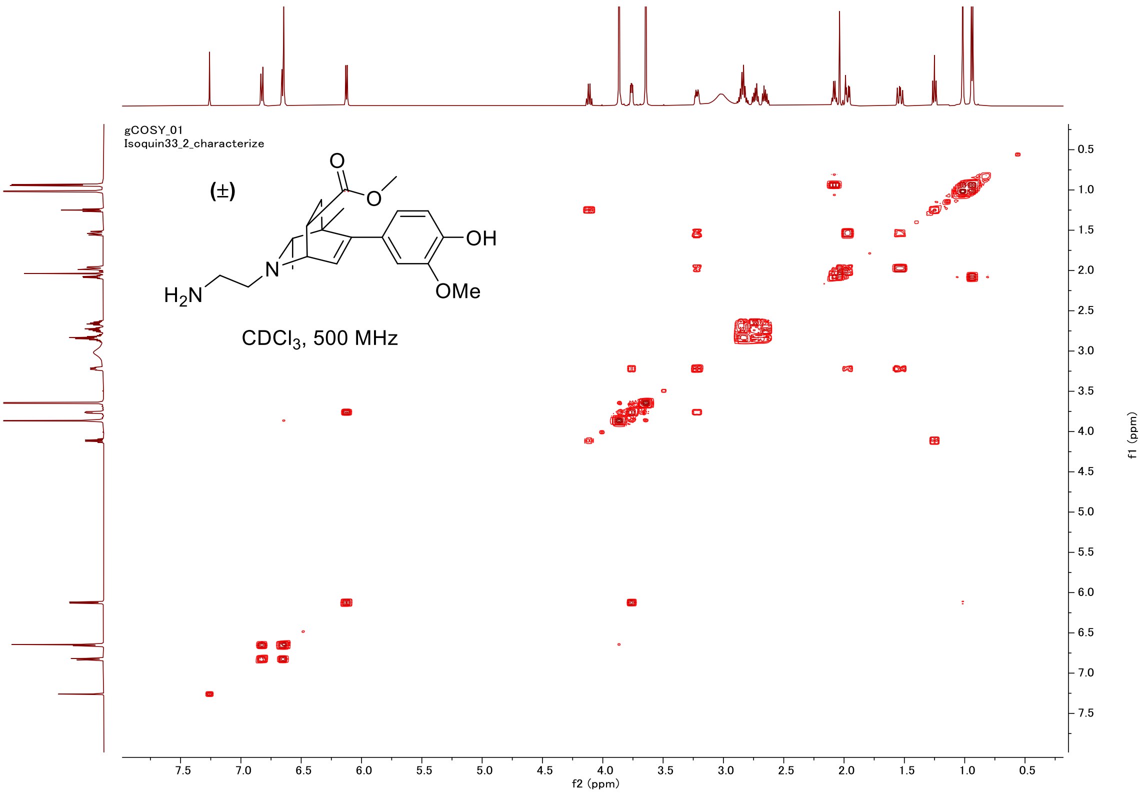
gCOSY_01
Isoquin33_2_characterize
(±)
OH
OMe
H2N
N
O
CDCl3, 500 MHz
f2 (ppm)
f1 (ppm)

HSQC

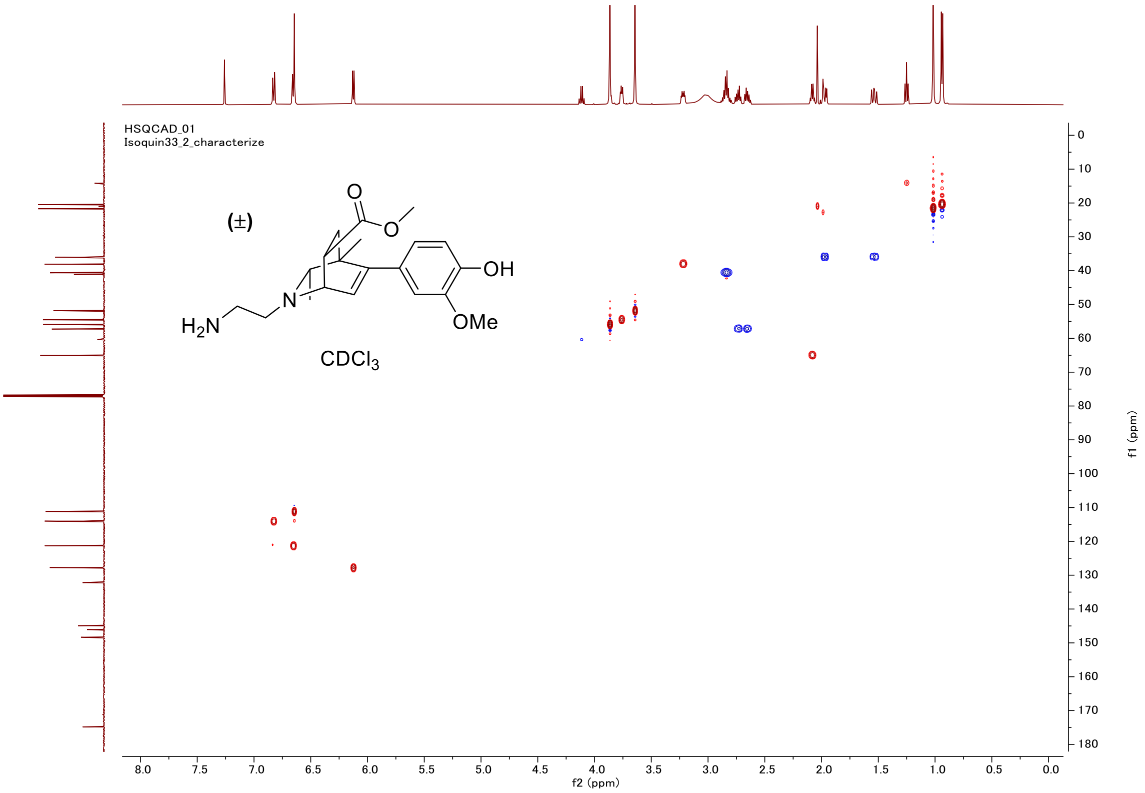
HSQCAD_01
Isoquin33_2_characterize
(±)
OH
OMe
H2N
N
O
CDCl3
f2 (ppm)
f1 (ppm)

Proton (#39)

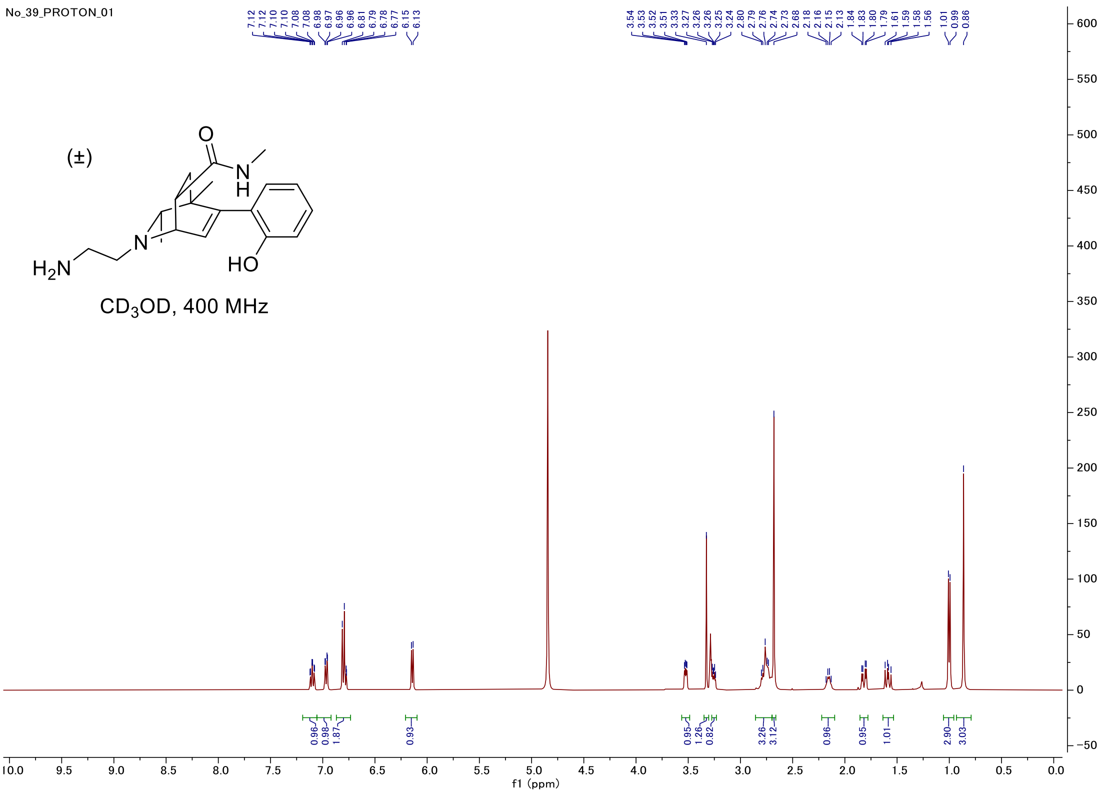

Carbon

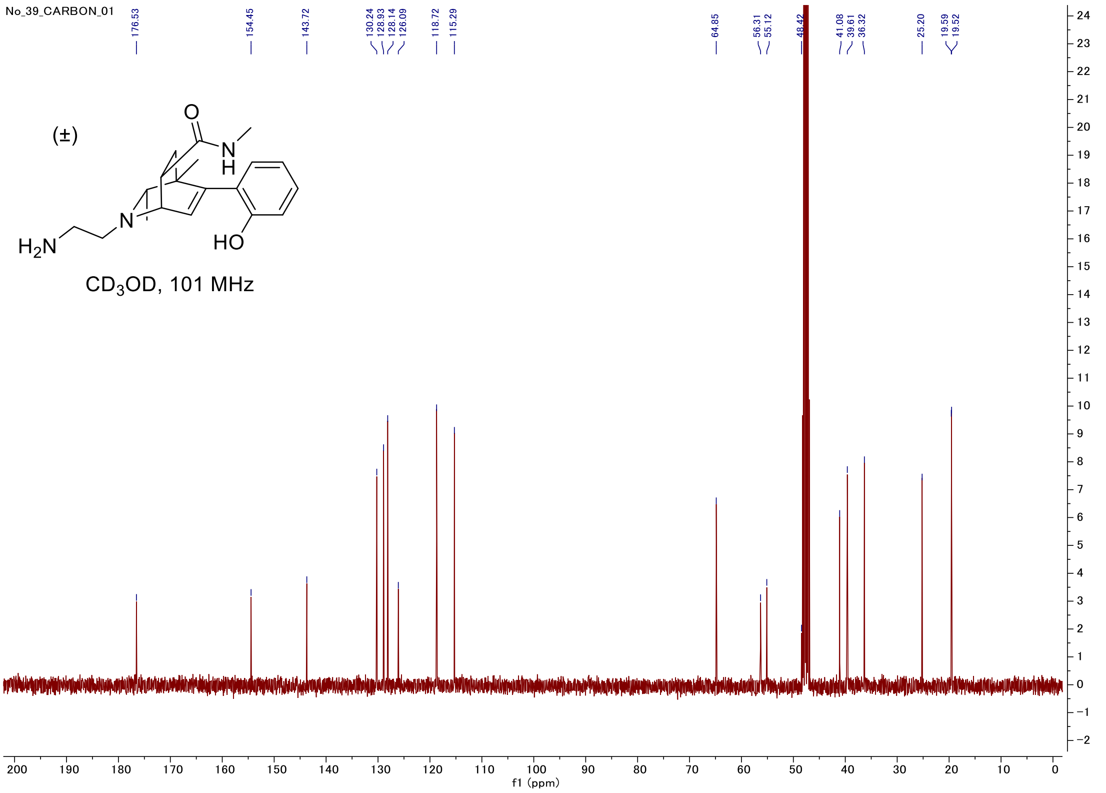

C-H COSY

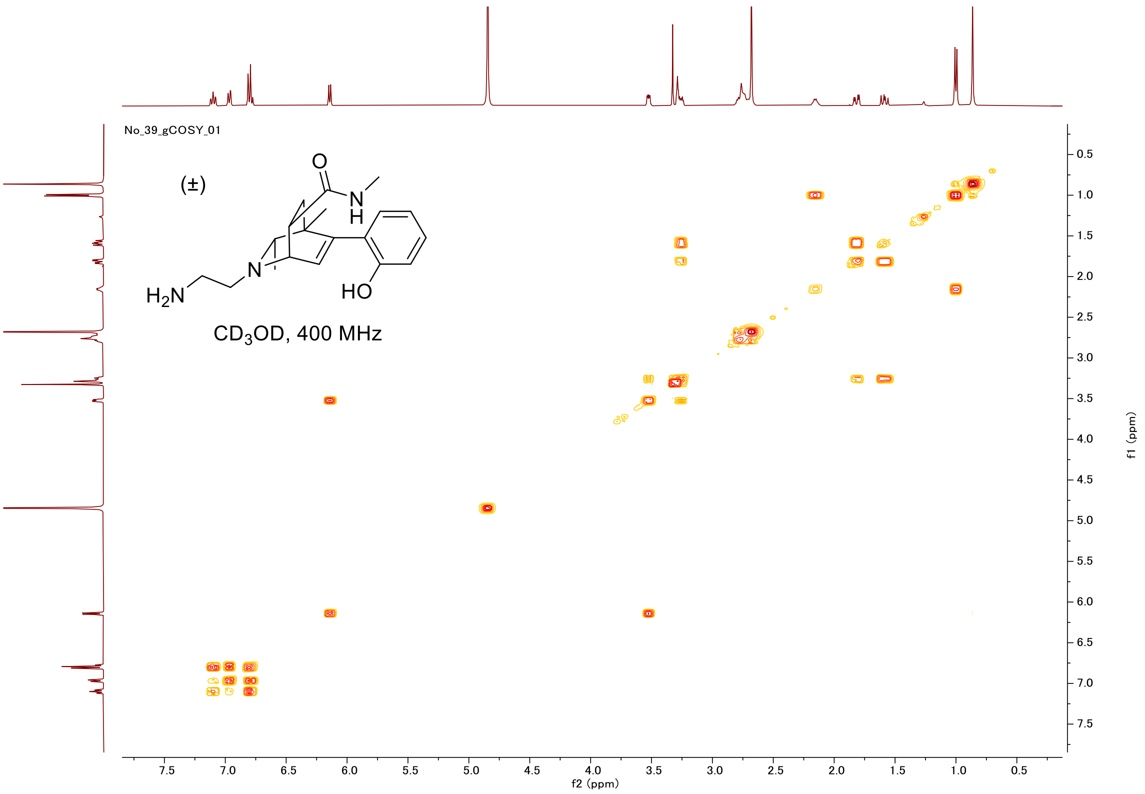
No_39_gCOSY_01
(±)
O
N
H
H2N
N
HO
CD3OD, 400 MHz
f2 (ppm)
f1 (ppm)

HSQC

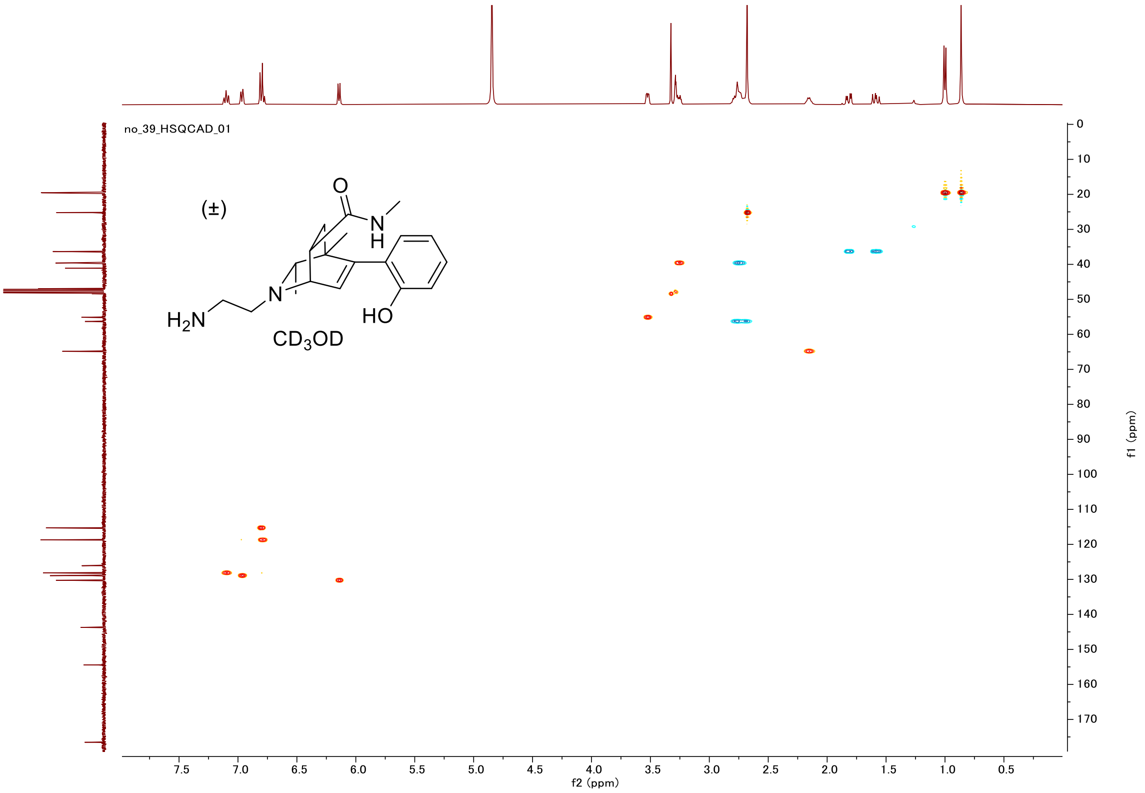
no_39_HSQCAD_01
(±)
O
N
H
H2N
N
HO
CD3OD
f2 (ppm)
f1 (ppm)

Proton (#53)

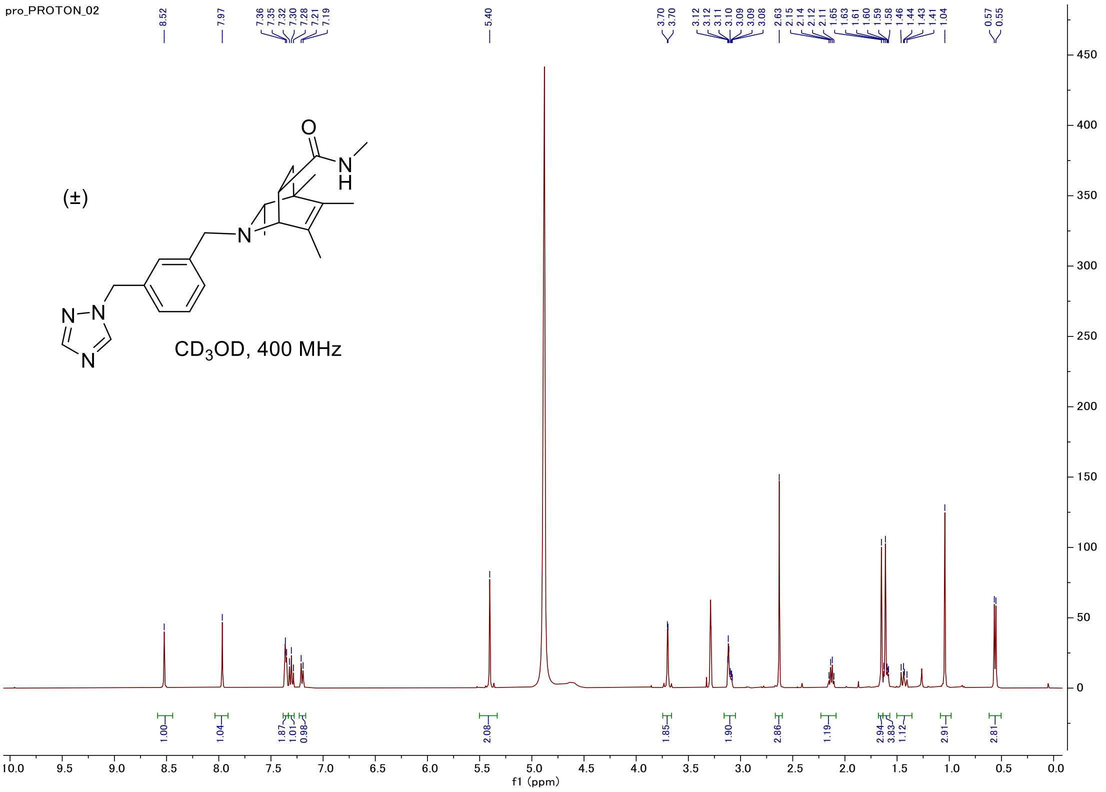

Carbon

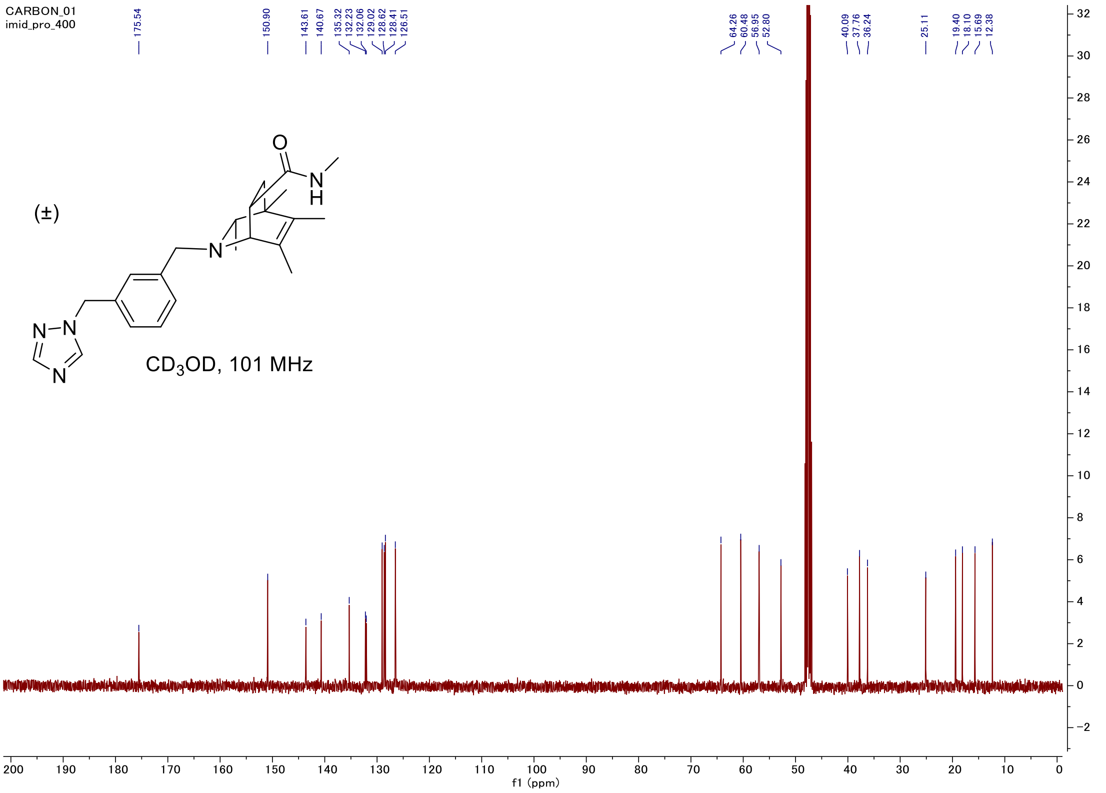

C-H COSY

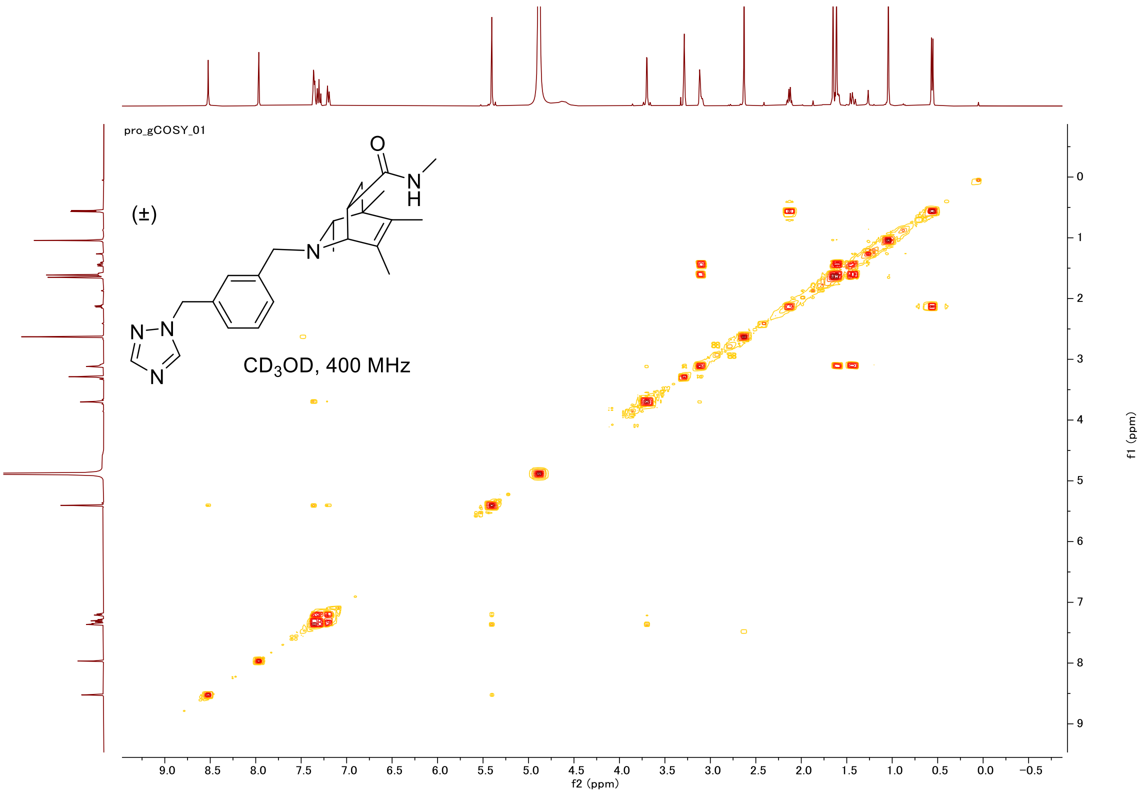
pro_gCOSY_01
(±)
CD$_3$OD, 400 MHz
f2 (ppm)
f1 (ppm)

HSQC

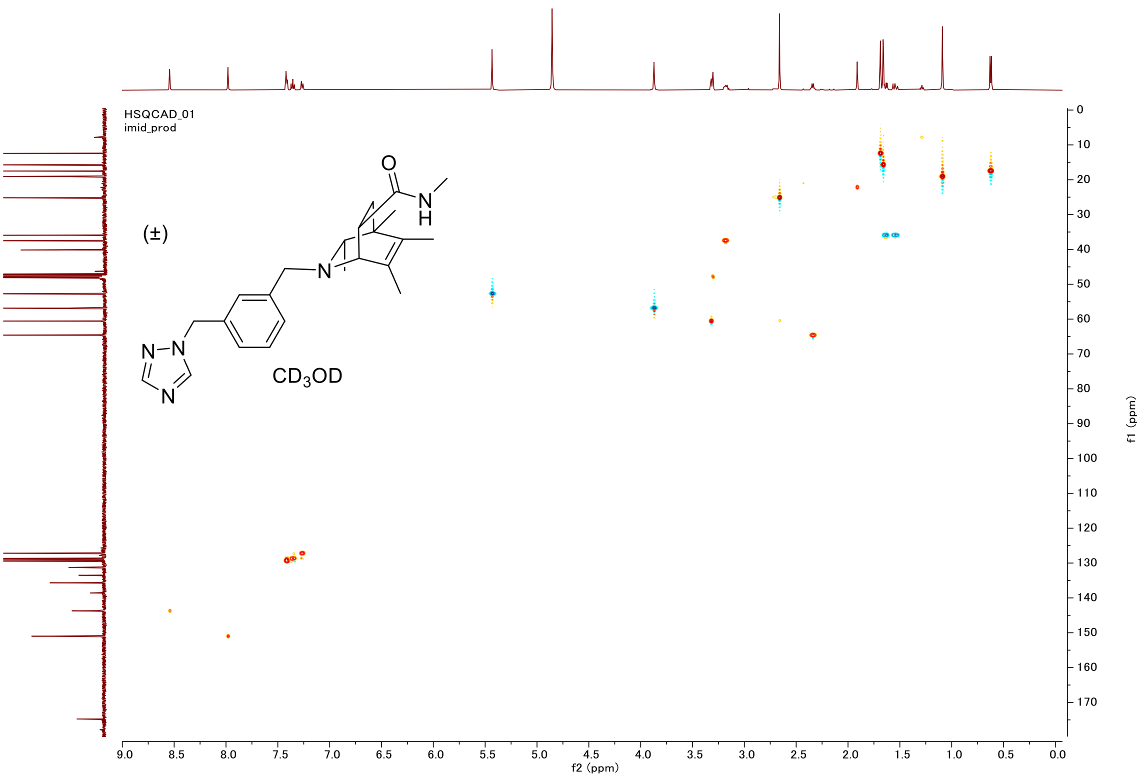
HSQCAD_01
imid_prod
(±)
CD$_3$OD
f2 (ppm)
f1 (ppm)

Proton (#56)

PROTON_01
Isoquin56_characterize

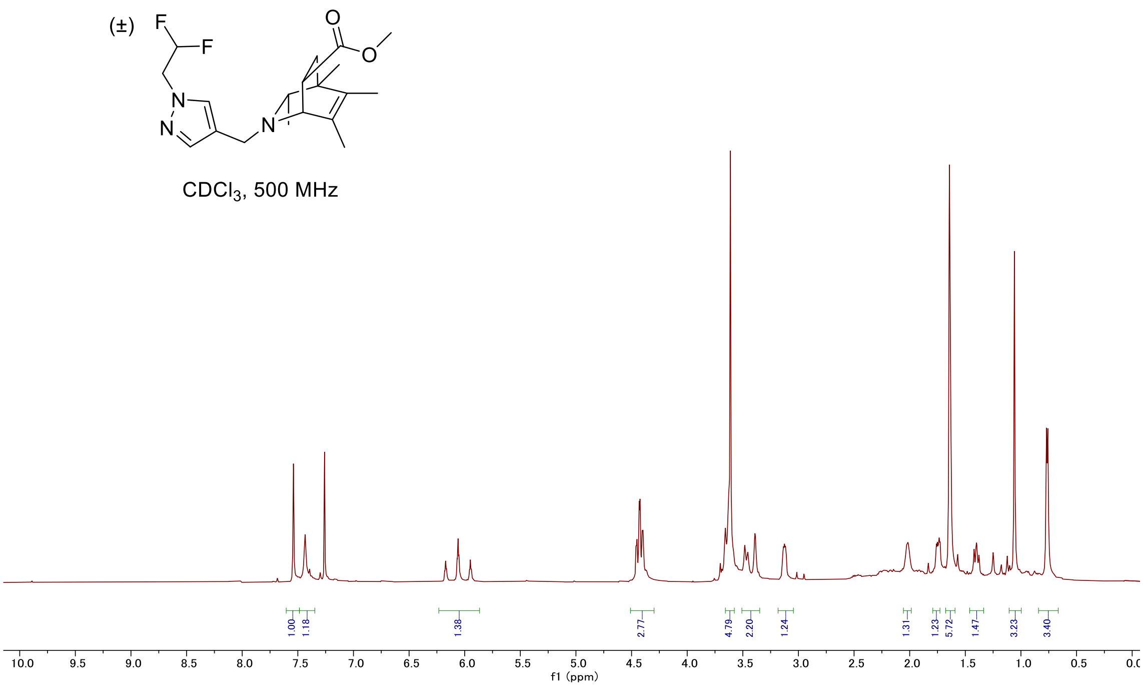

Carbon

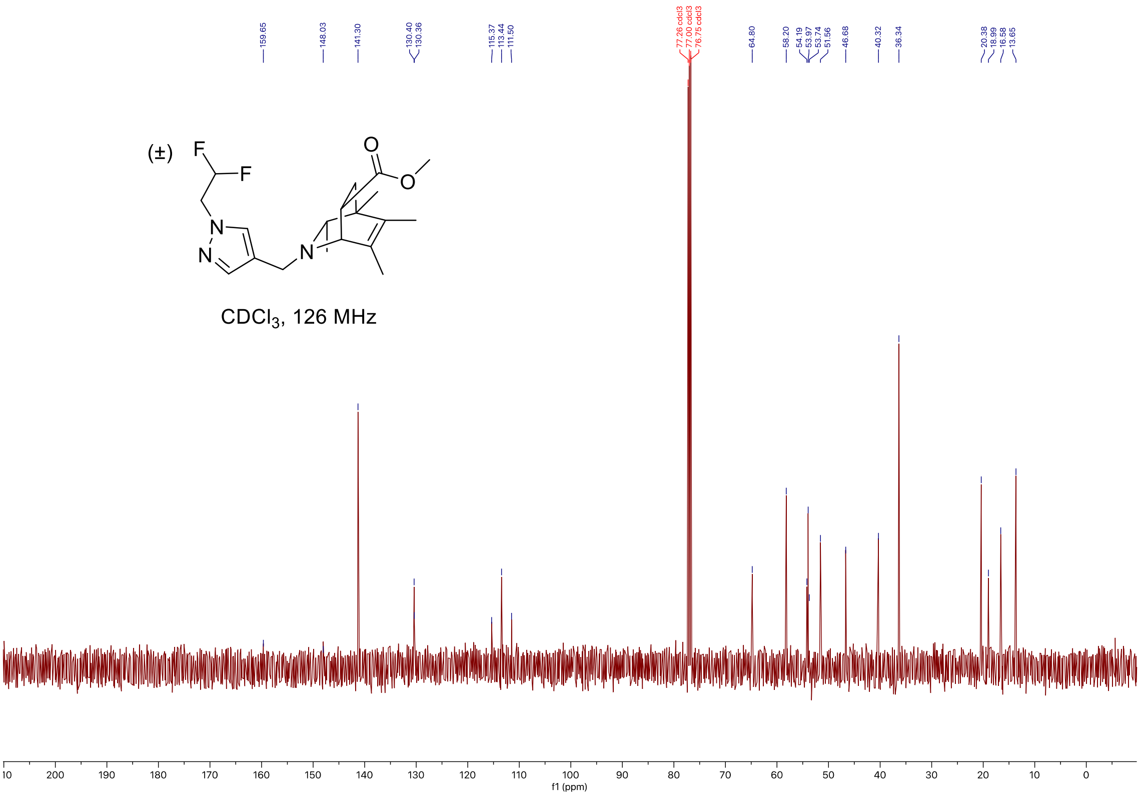

C-H COSY

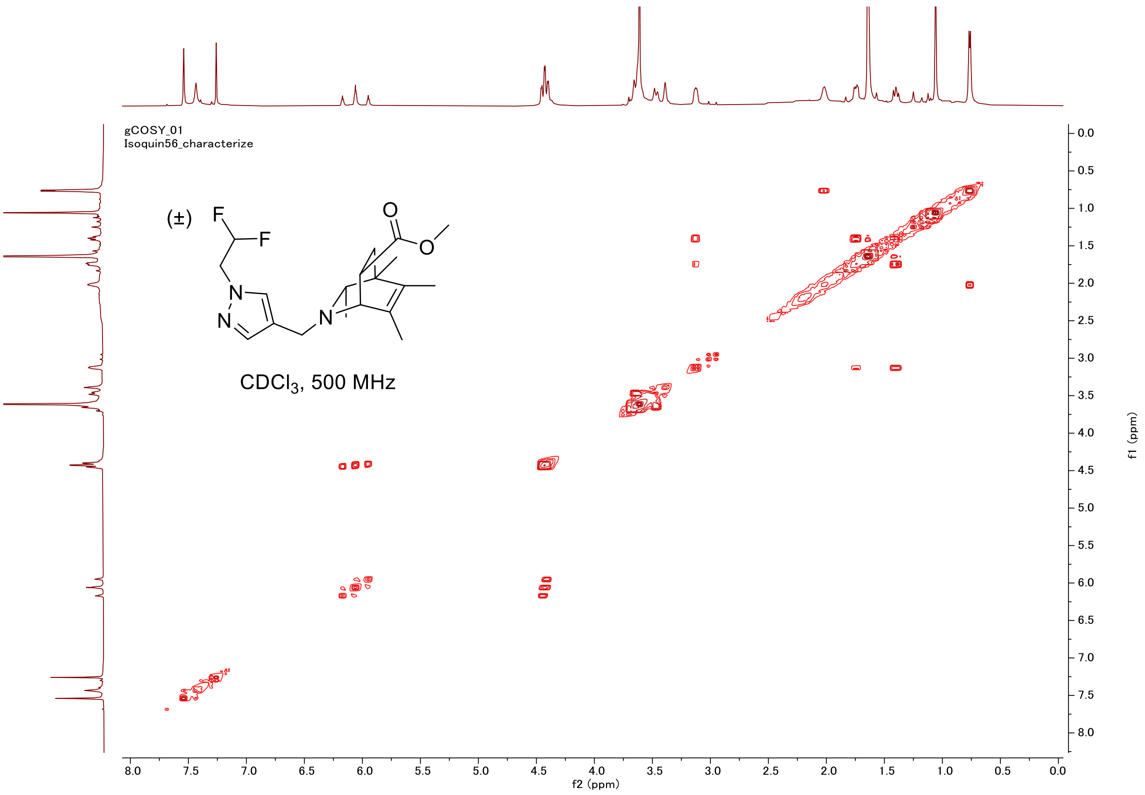
gCOSY_01
Isoquin56_characterize
(±)
CDCl3, 500 MHz
f2 (ppm)
f1 (ppm)

HSQC

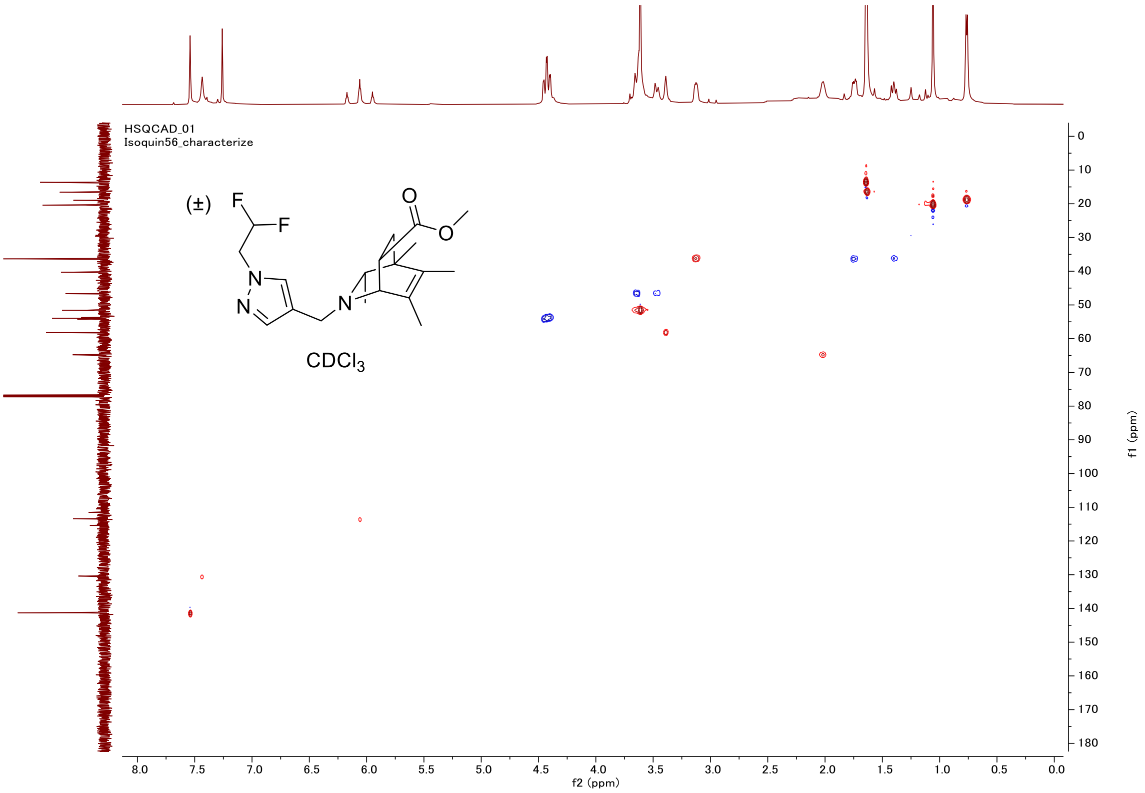
HSQCAD_01
Isoquin56_characterize
(±)
CDCl3
f2 (ppm)
f1 (ppm)

Proton (#57)

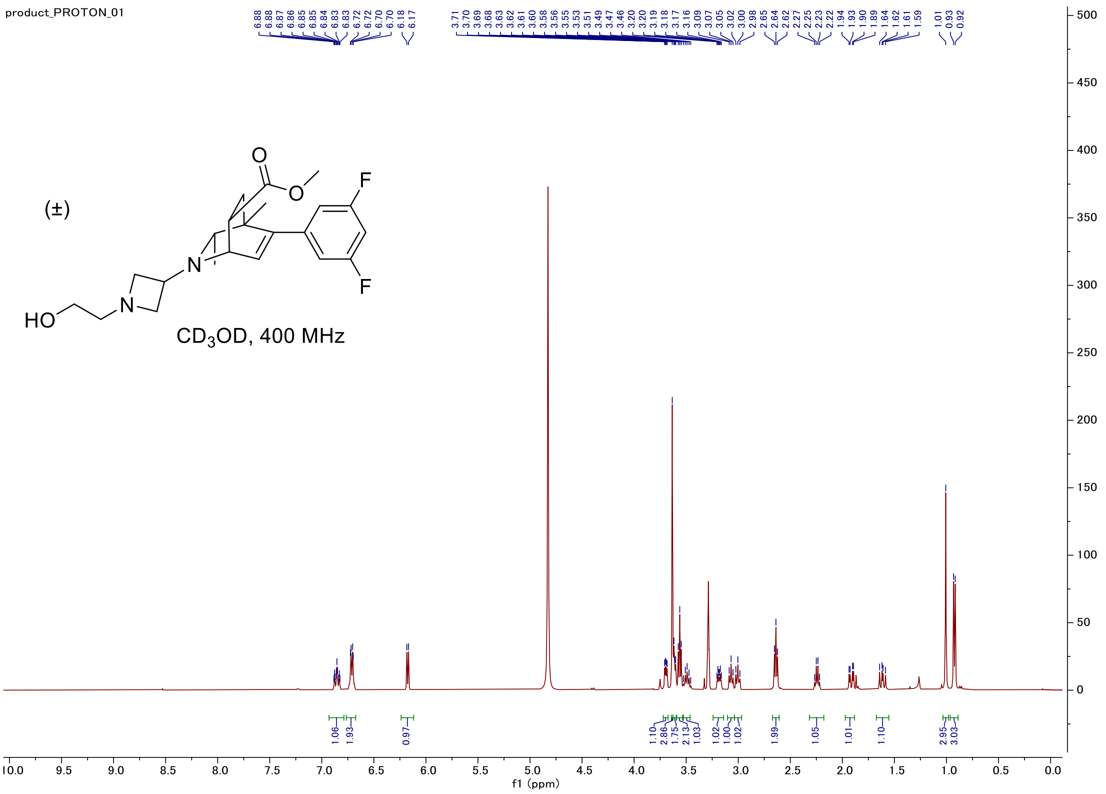

Carbon

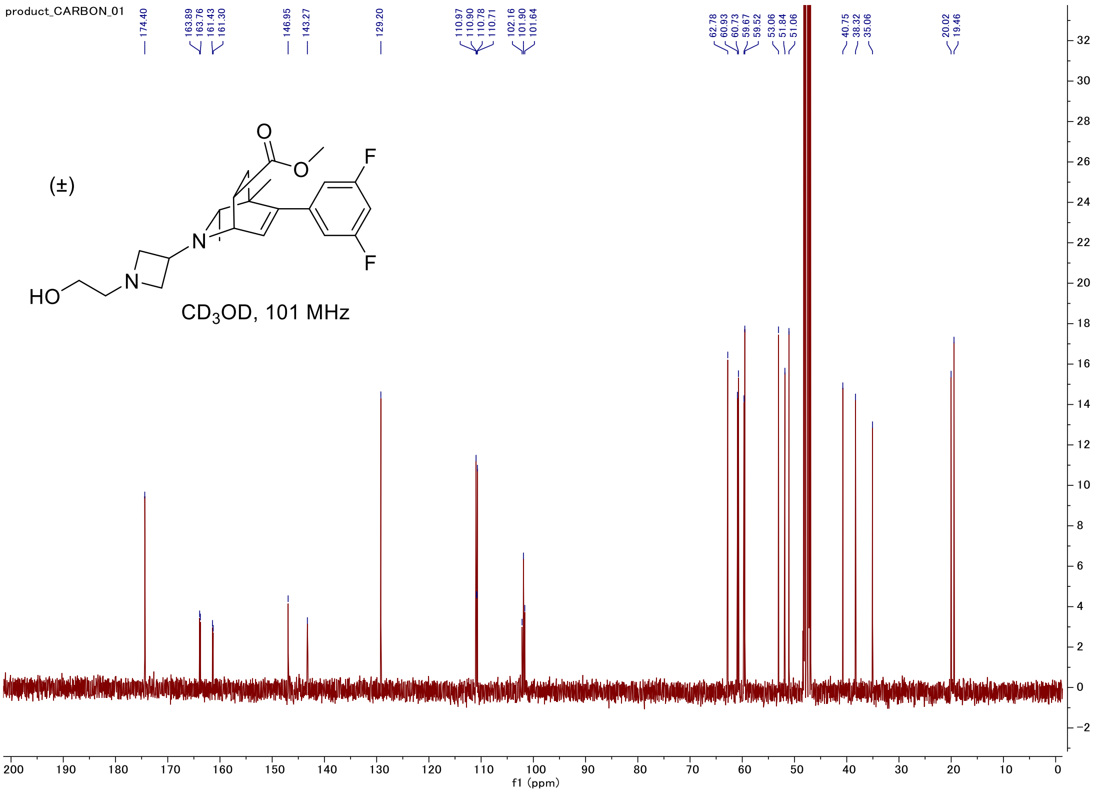

Fluorine

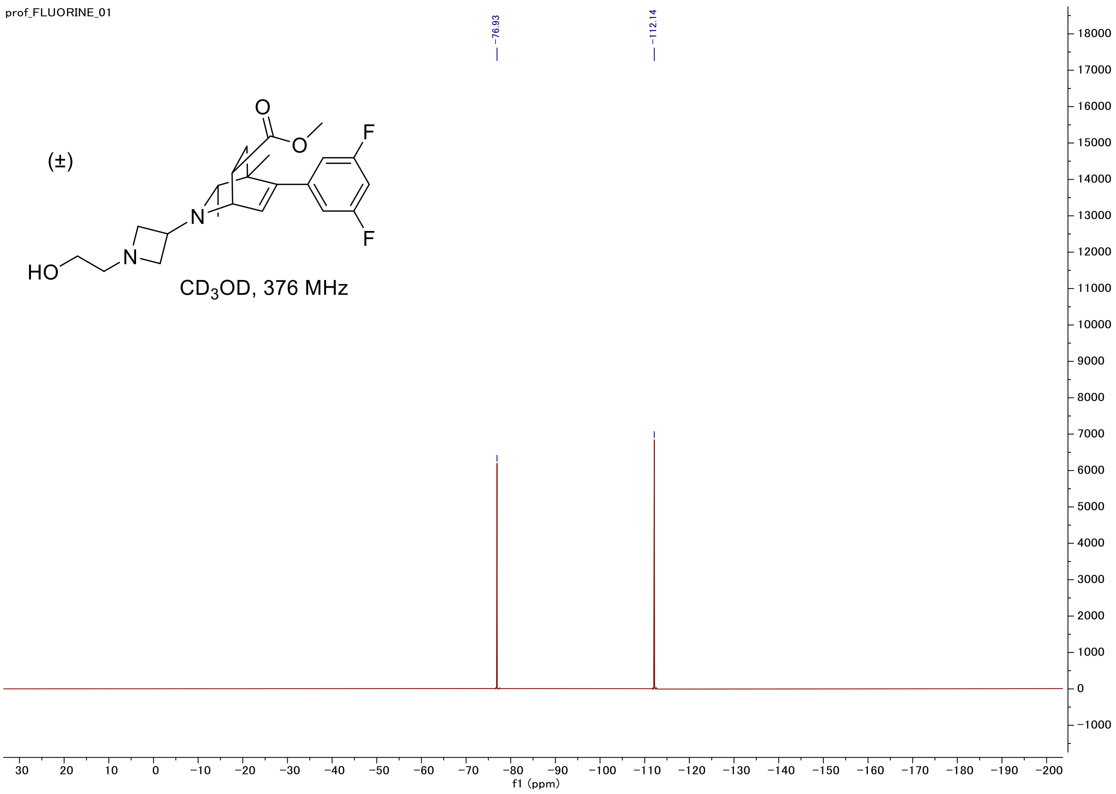

C-H COSY

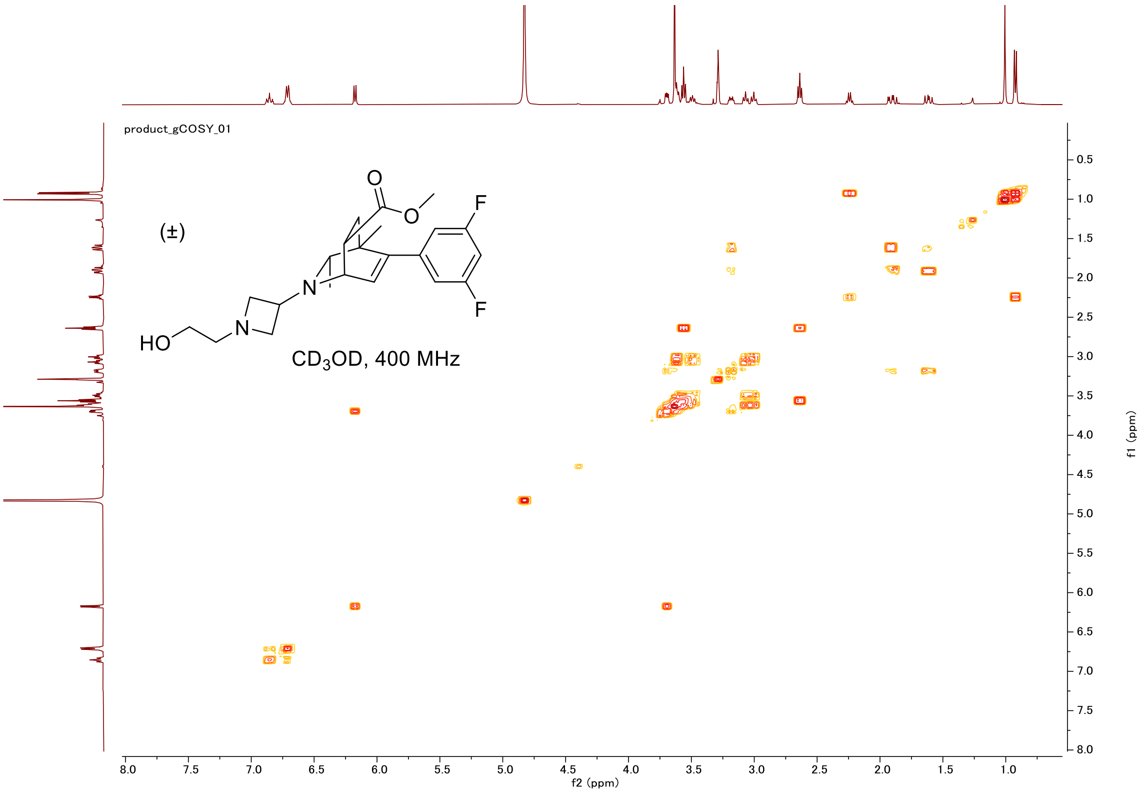

HSQC

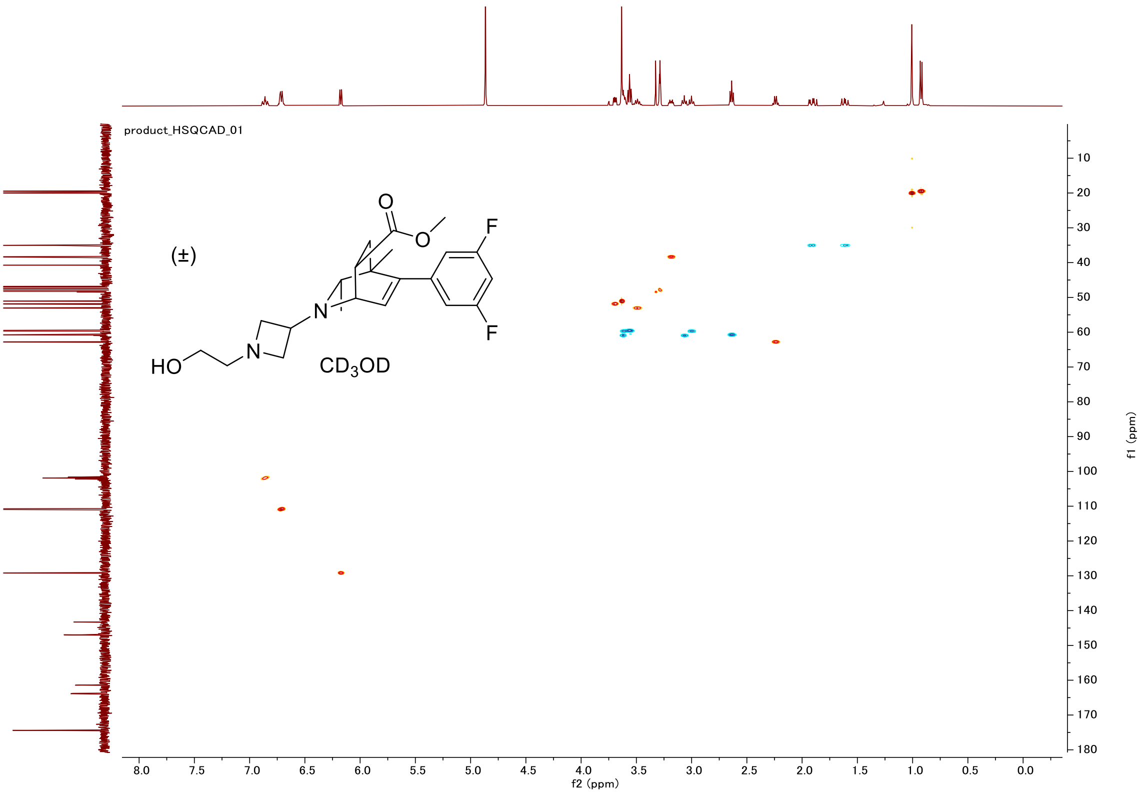

Proton (#58)

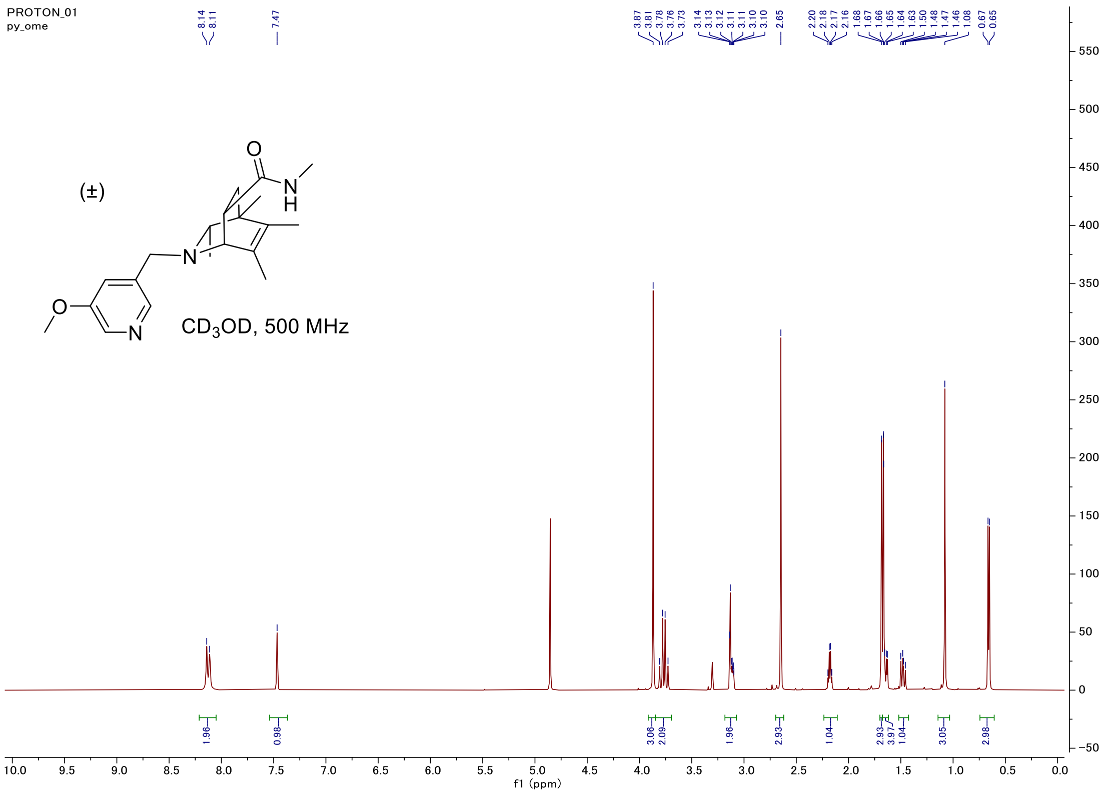

Carbon

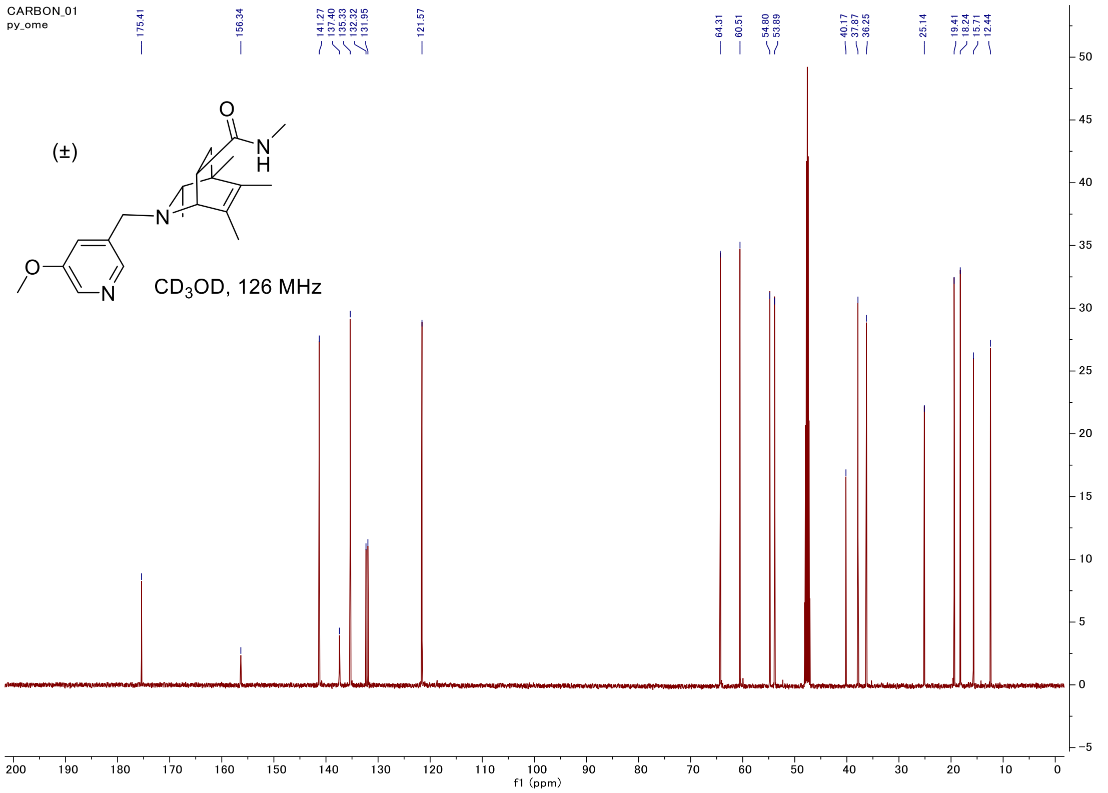

C-H COSY

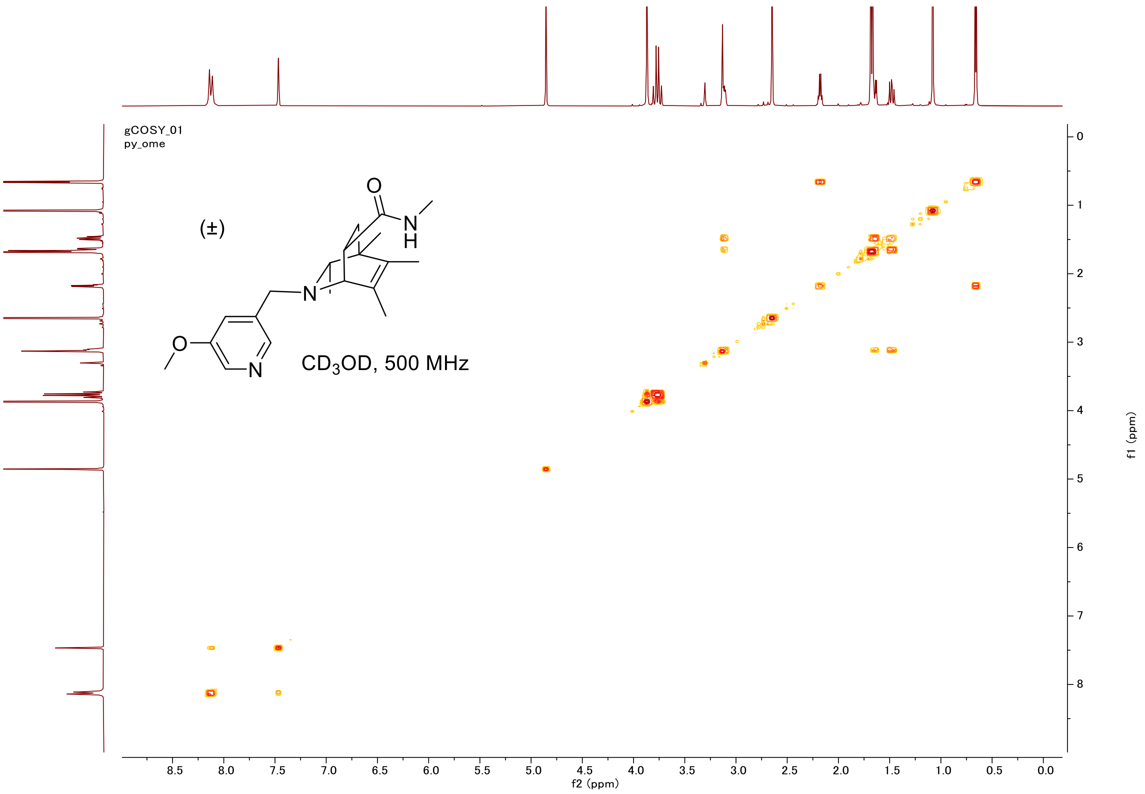
gCOSY_01
py_ome
(±)
CD3OD, 500 MHz
f2 (ppm)
f1 (ppm)

HSQC

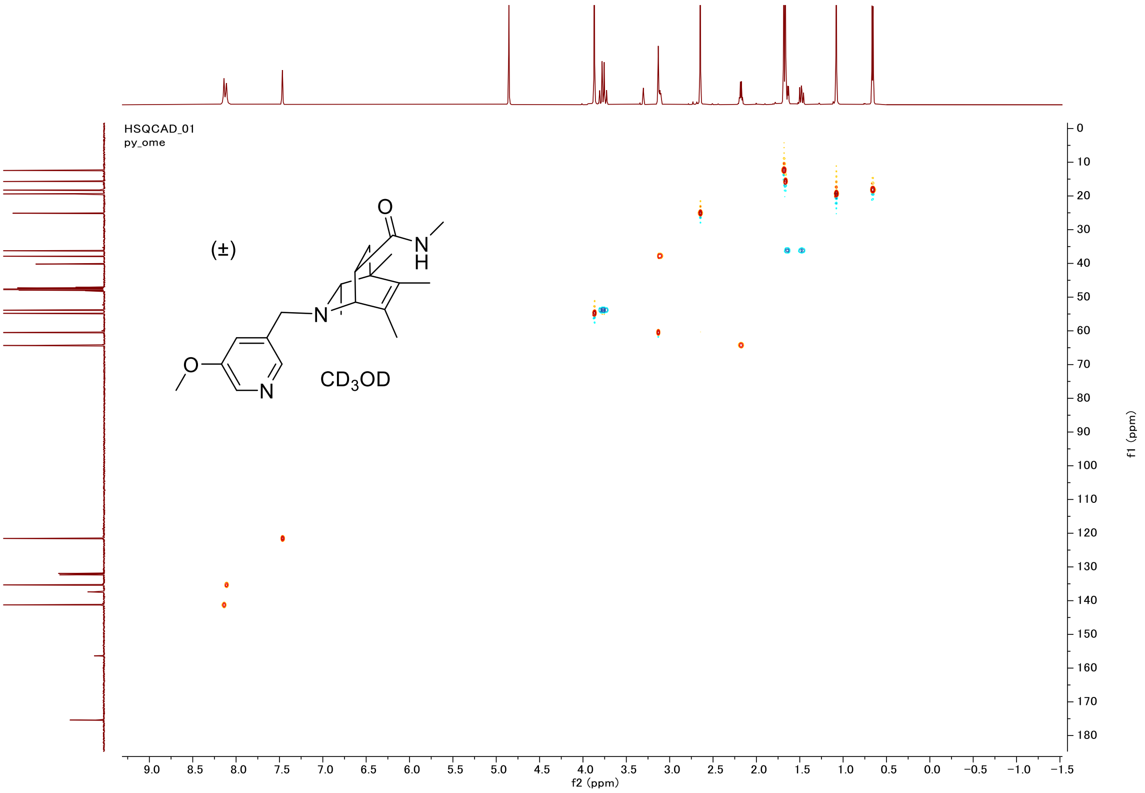
HSQCAD_01
py_ome
(±)
CD3OD
f2 (ppm)
f1 (ppm)

Proton (#61)

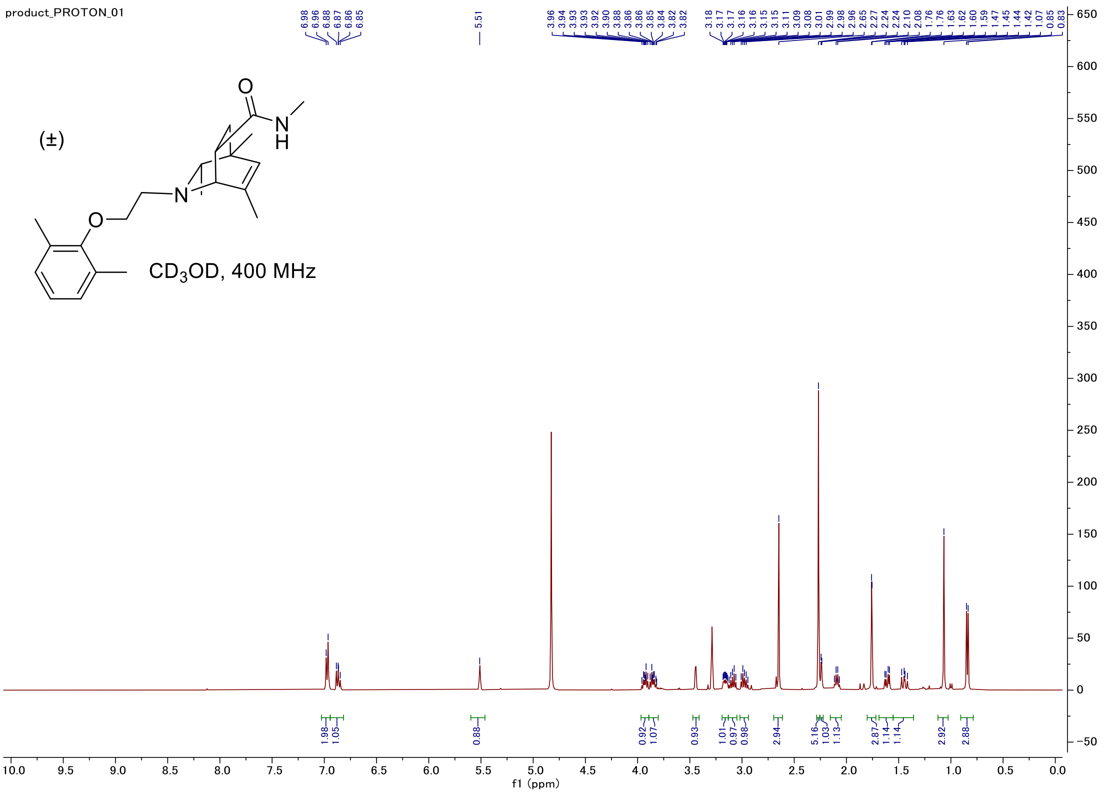

Carbon

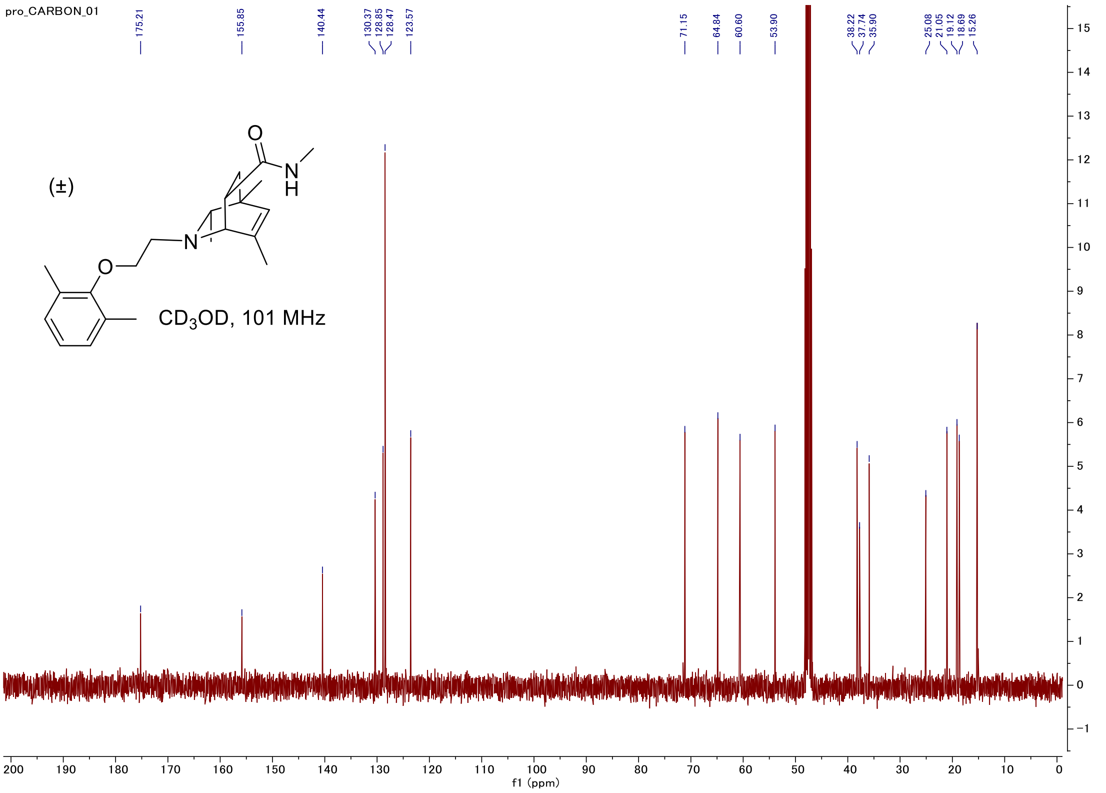

C-H COSY

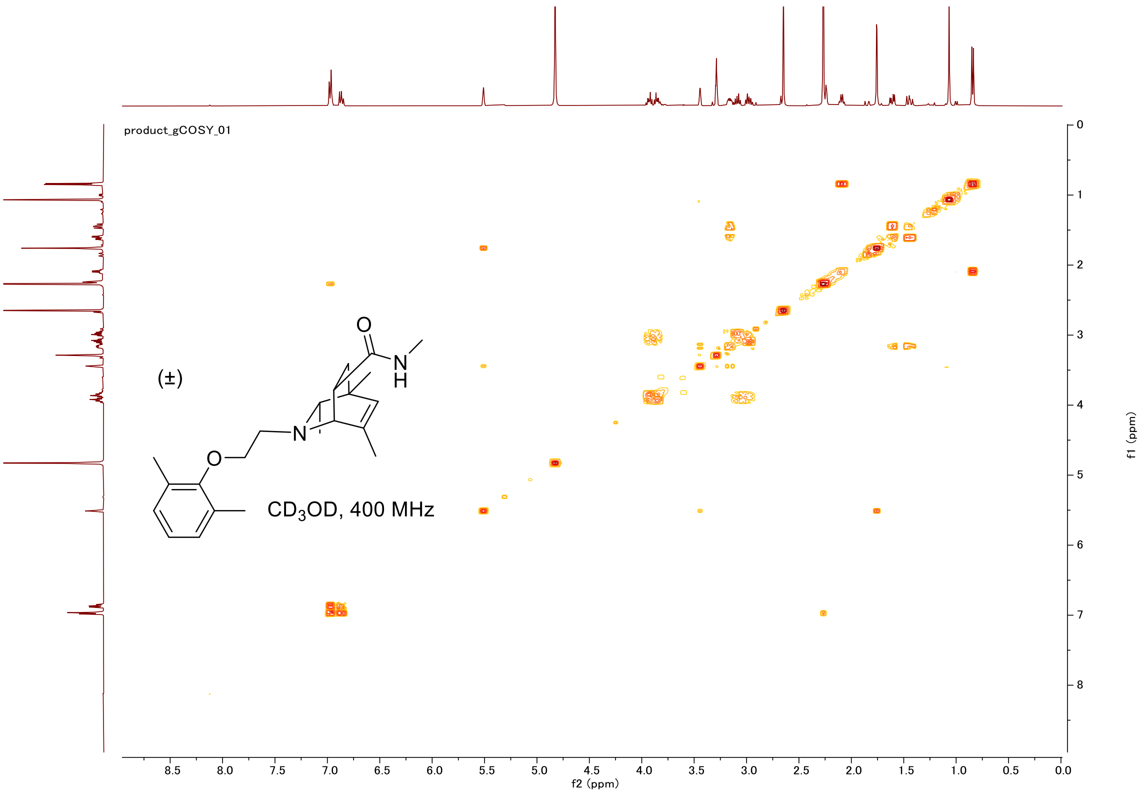
product_gCOSY_01
(±)
O
N
H
N
O
CD3OD, 400 MHz
f2 (ppm)
f1 (ppm)

HSQC

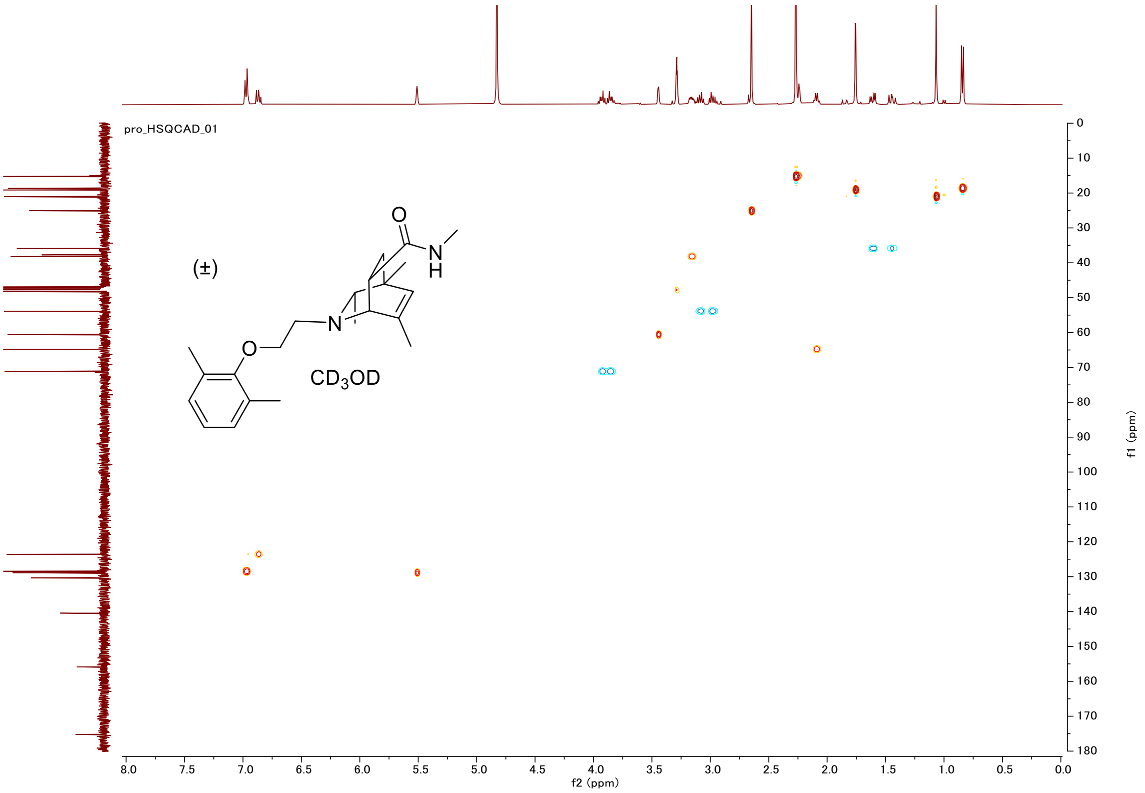
pro_HSQCAD_01
(±)
O
N
H
N
O
CD3OD
f2 (ppm)
f1 (ppm)

Proton (#64)

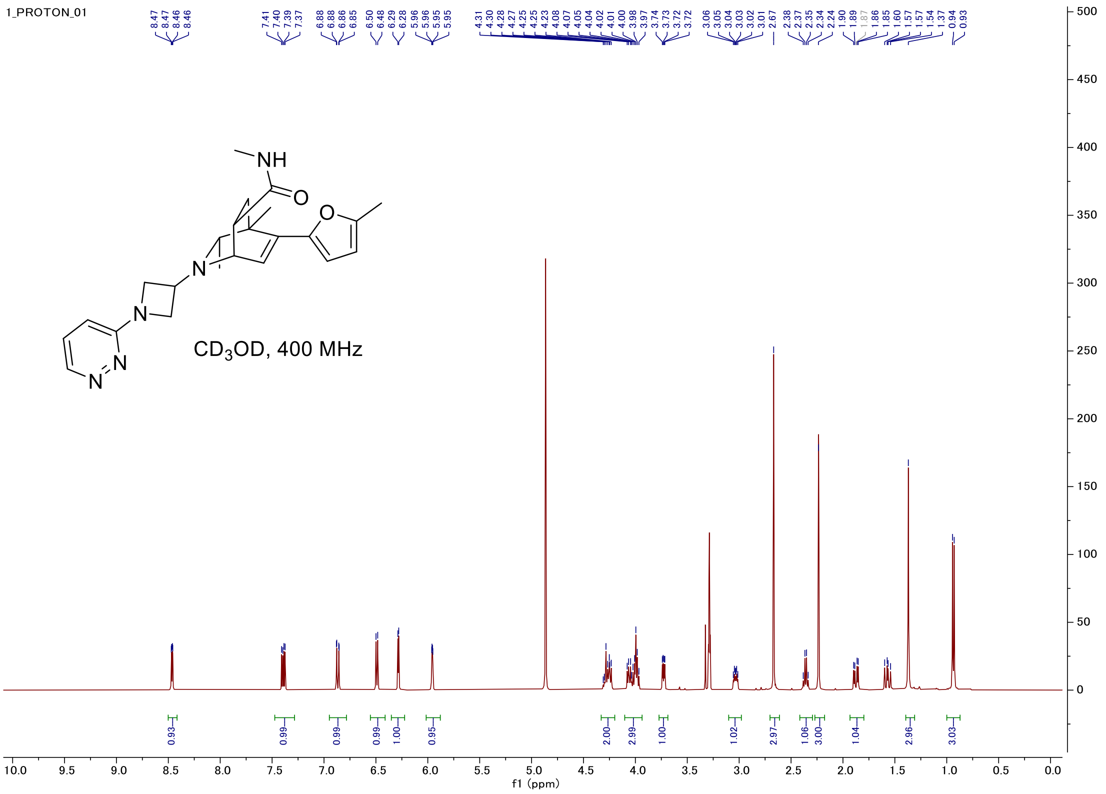

Carbon

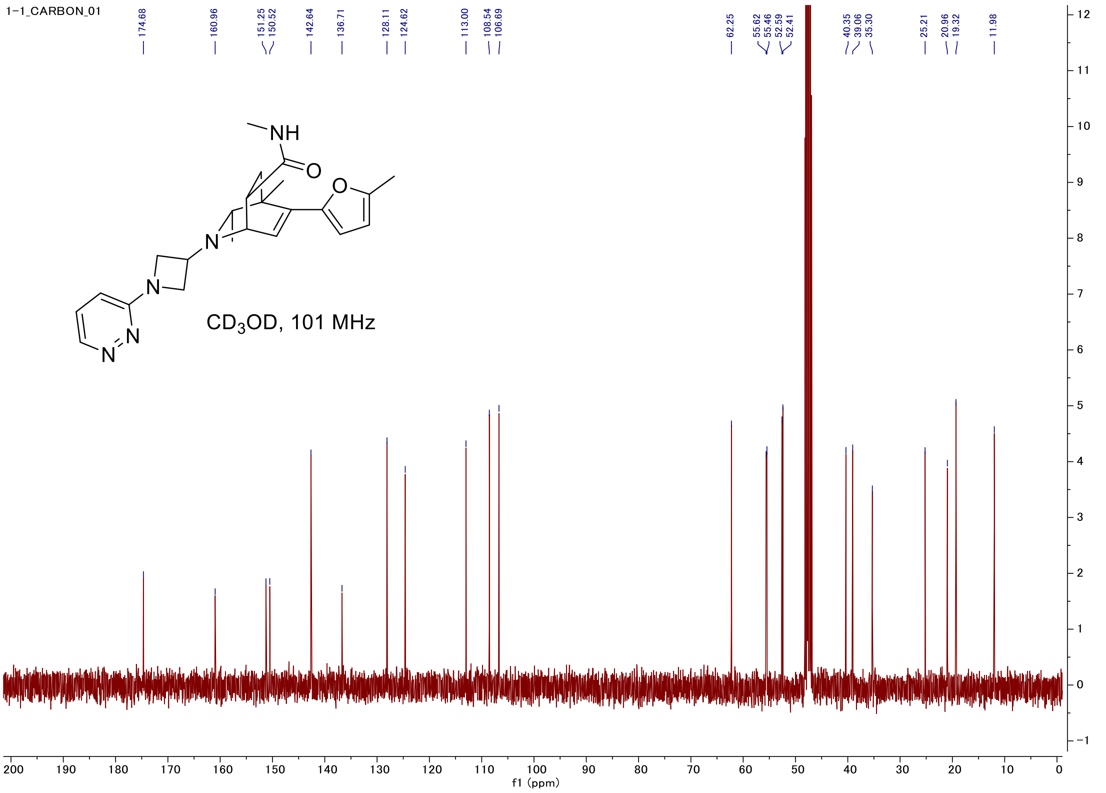

C-H COSY

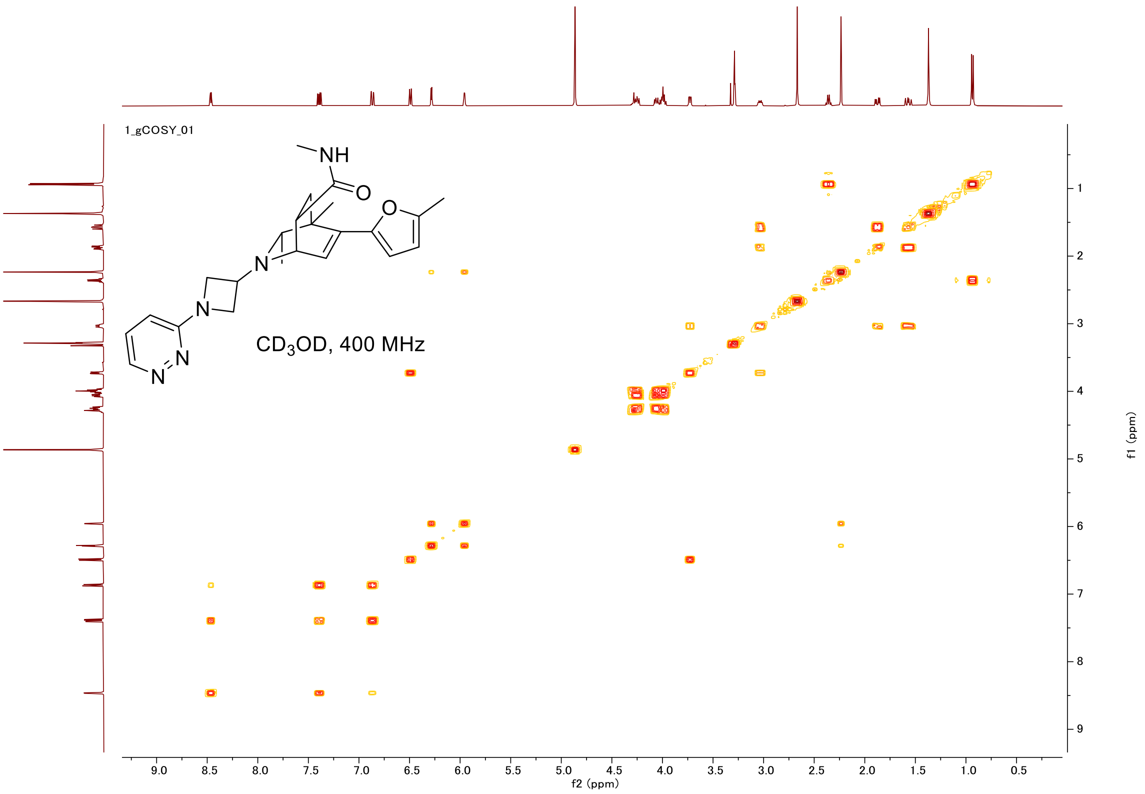
1_gCOSY_01
NH
O
O
N
N
N N
CD3OD, 400 MHz
f2 (ppm)
f1 (ppm)

HSQC

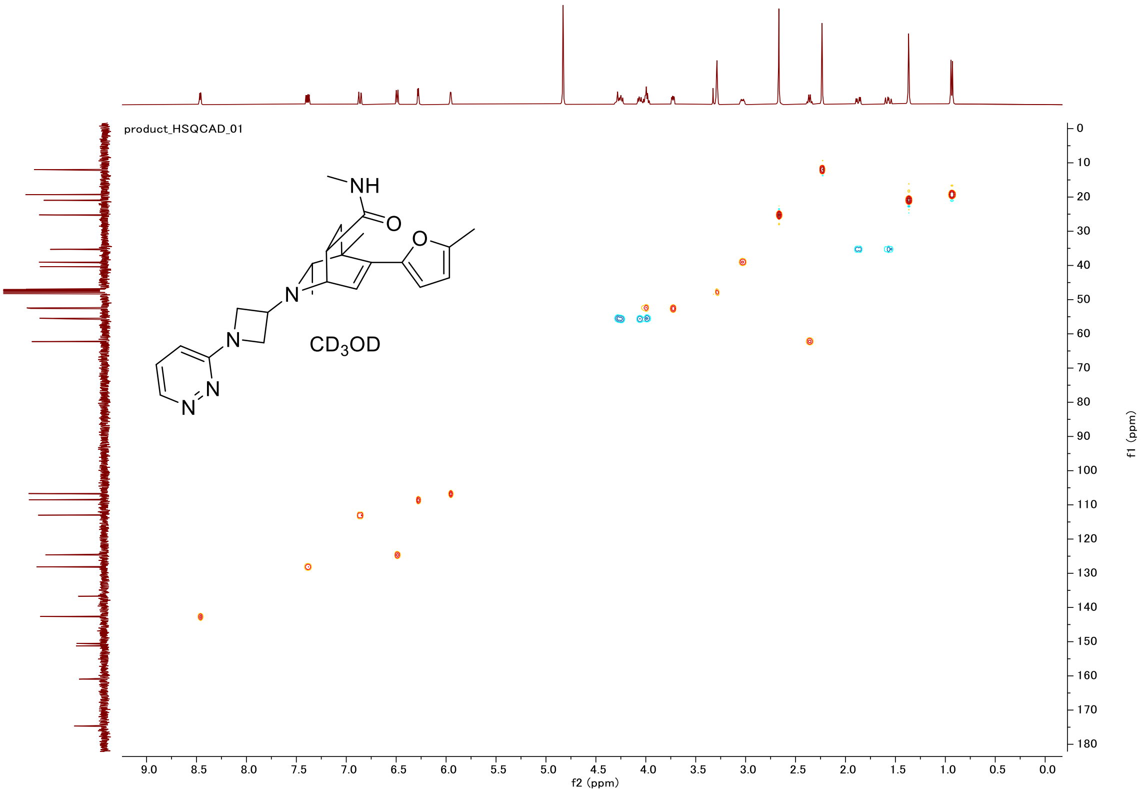
product_HSQCAD_01
NH
O
O
N
N
N N
CD3OD
f2 (ppm)
f1 (ppm)

Proton (#001)

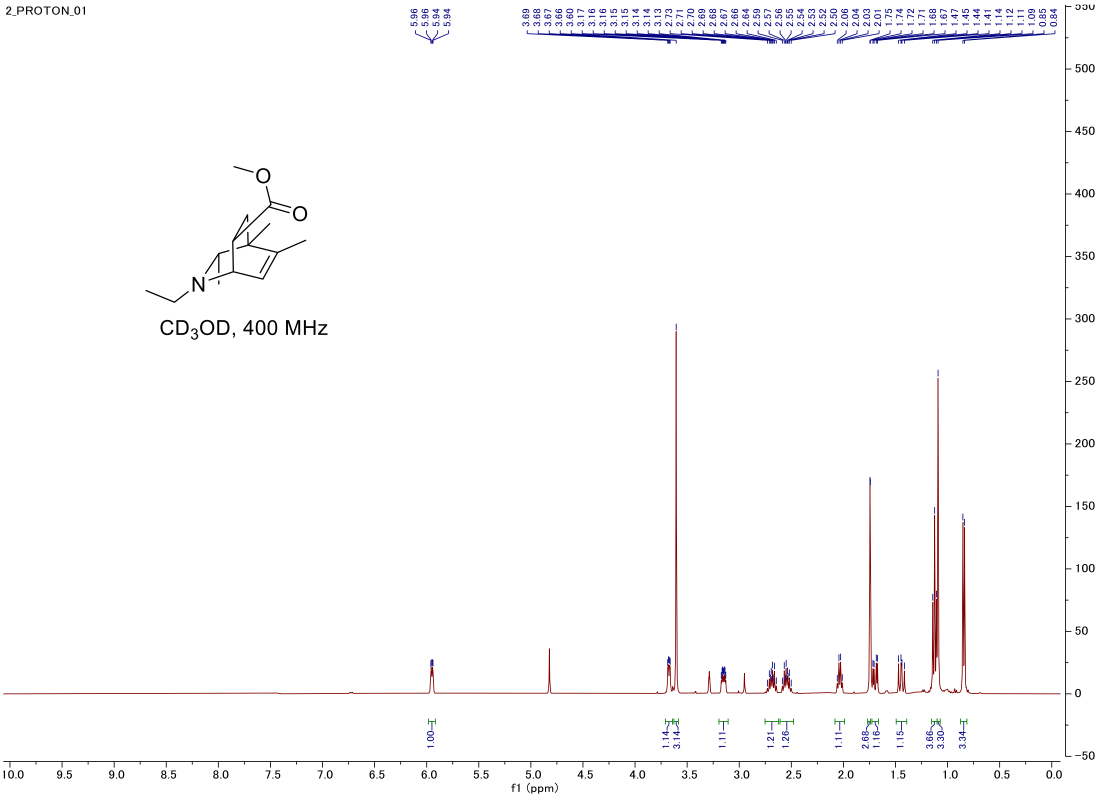

Carbon

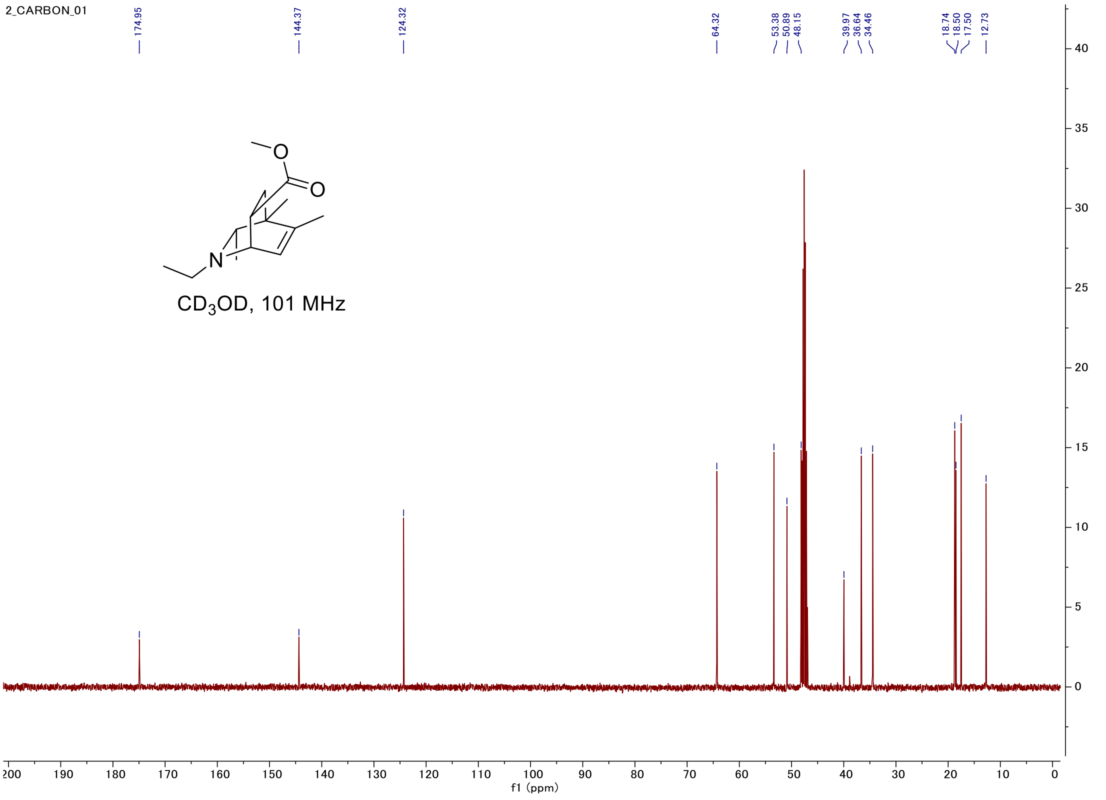

C-H COSY

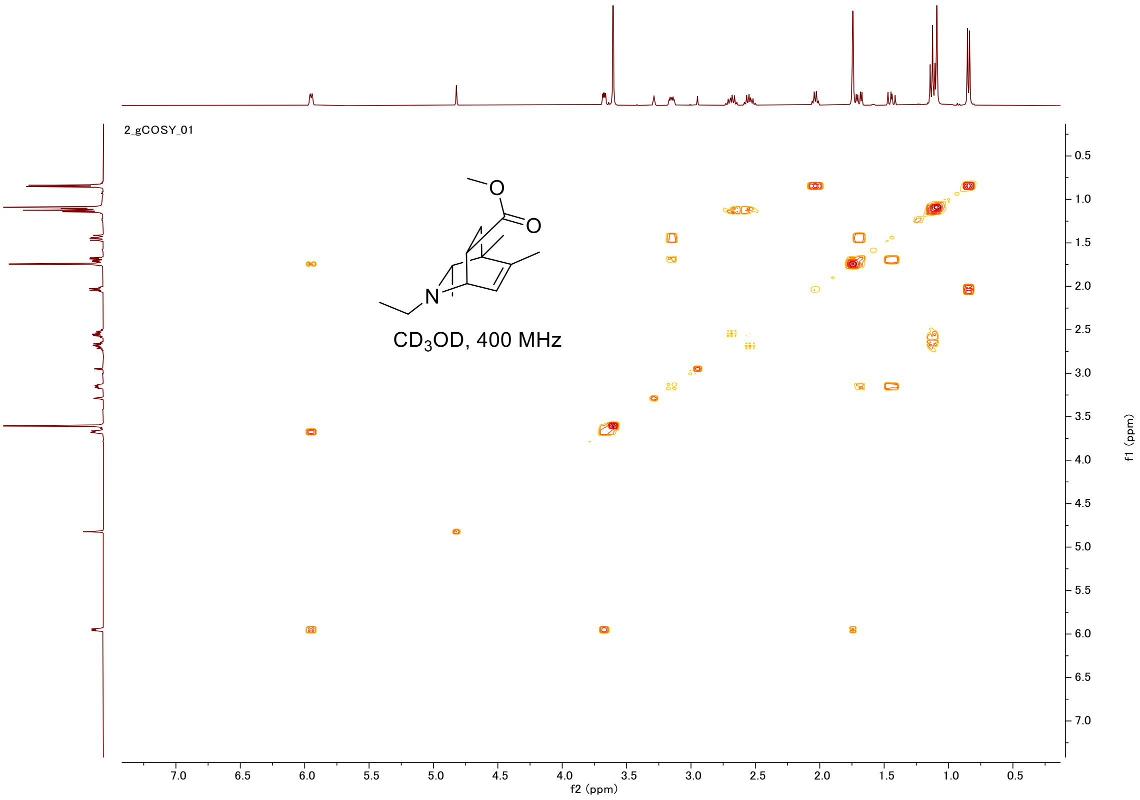
2_gCOSY_01
CD3OD, 400 MHz
f2 (ppm)
f1 (ppm)

HSQC

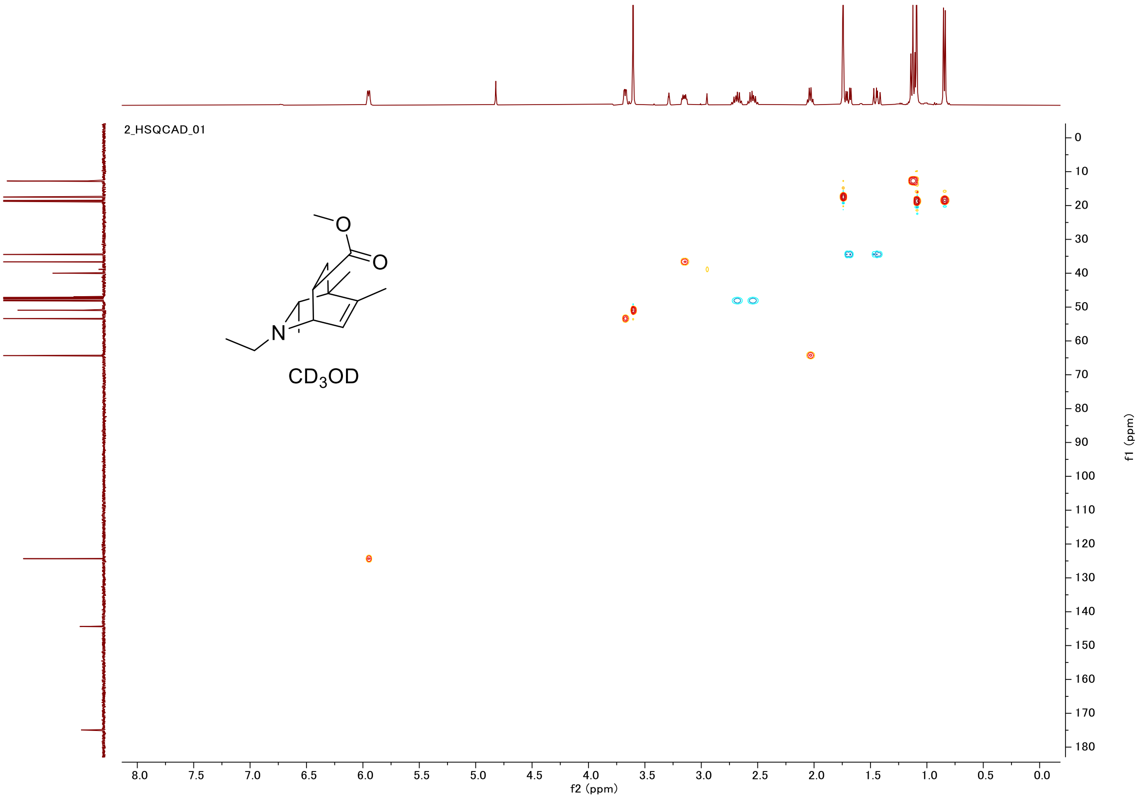
2_HSQCAD_01
CD3OD
f2 (ppm)
f1 (ppm)

Proton (#002)

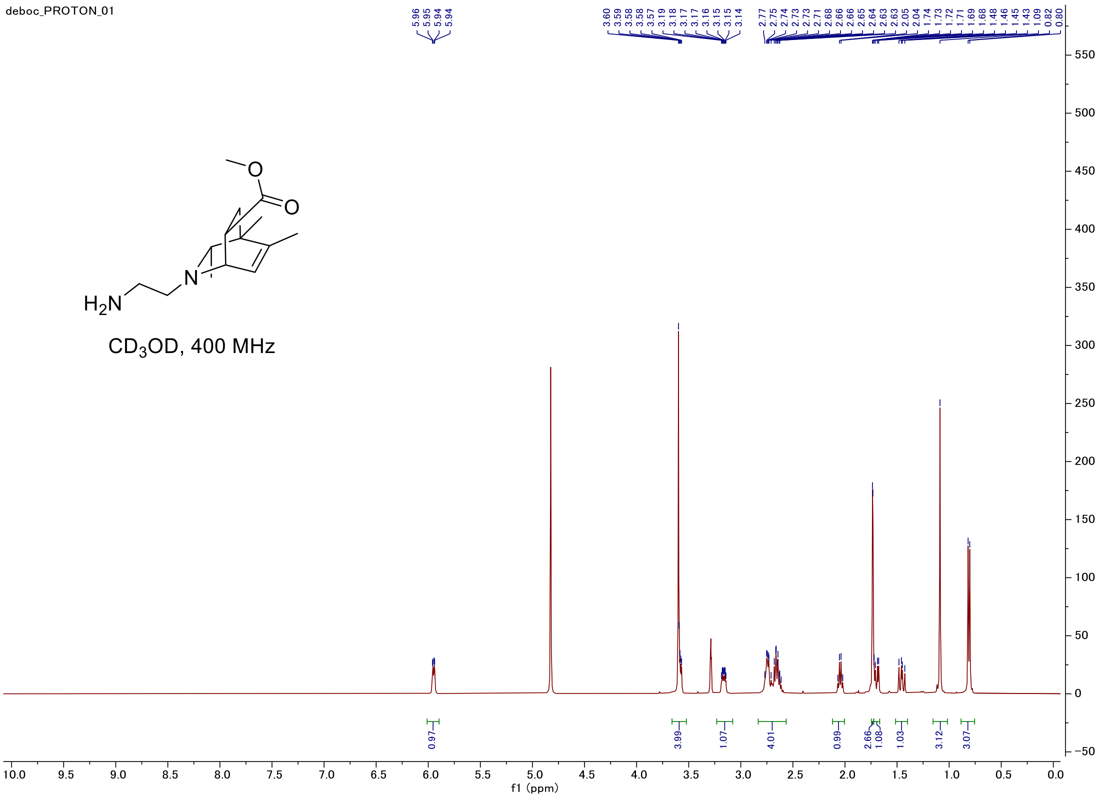

Carbon

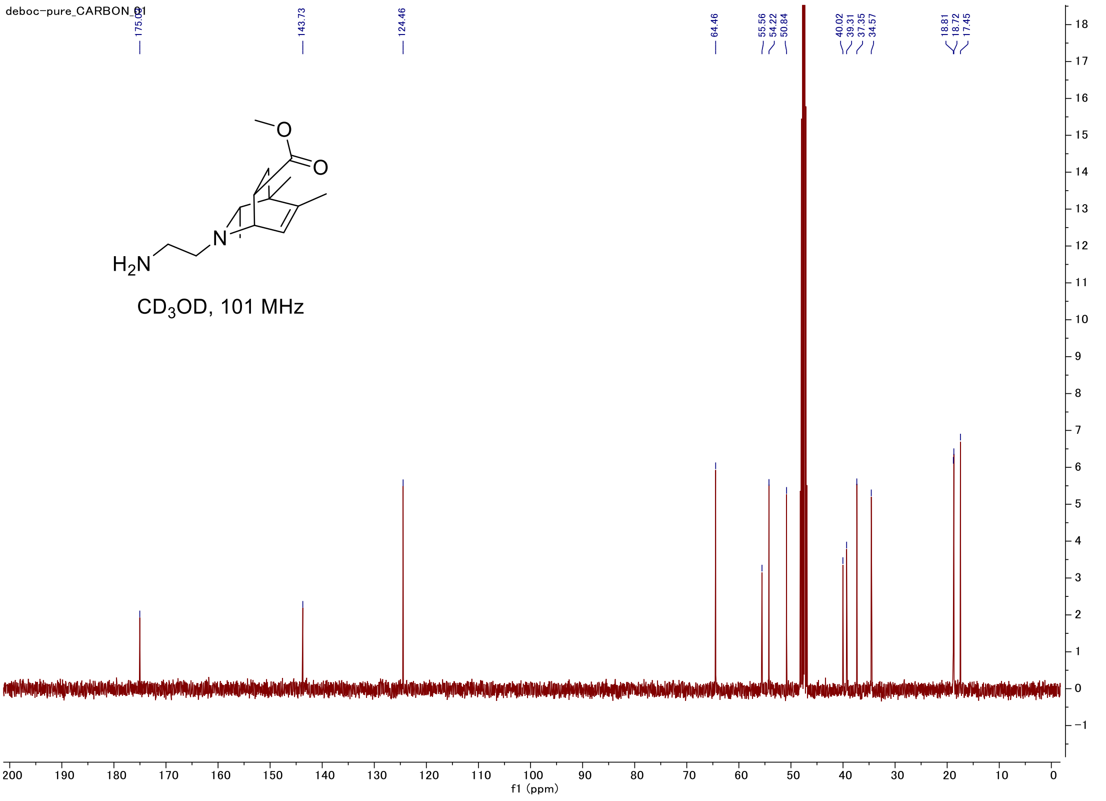

C-H COSY

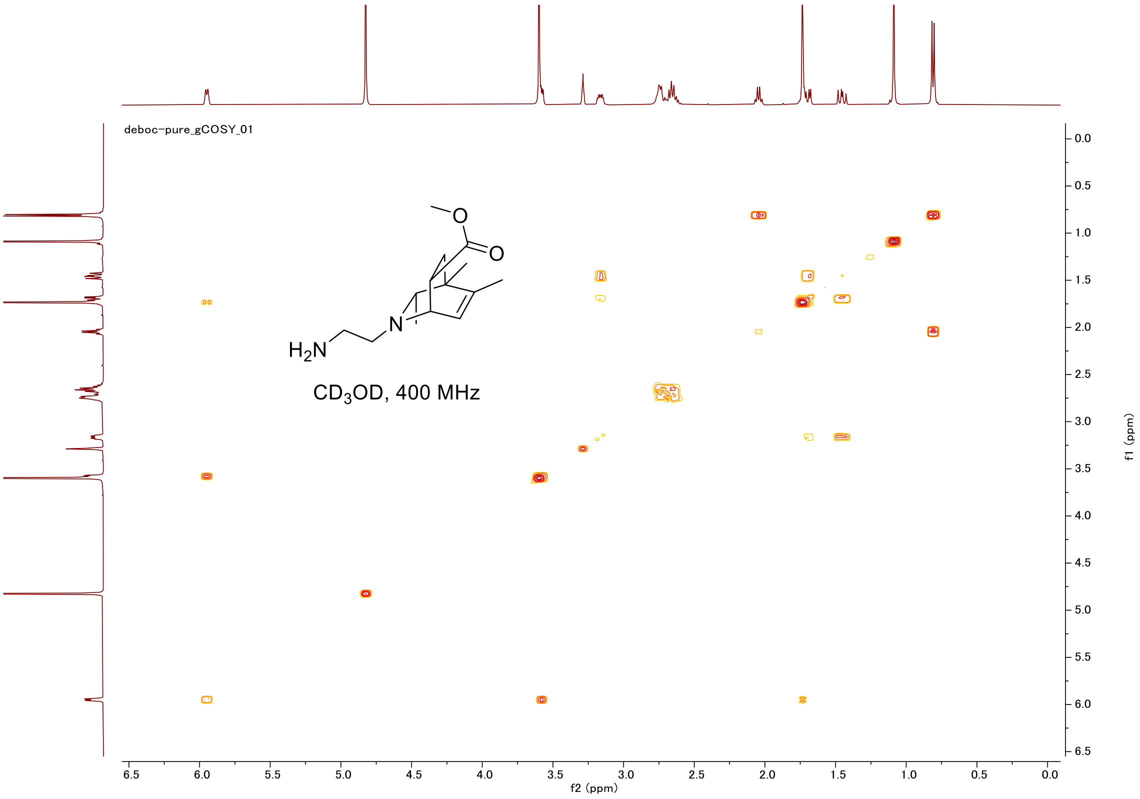

Proton (#003)

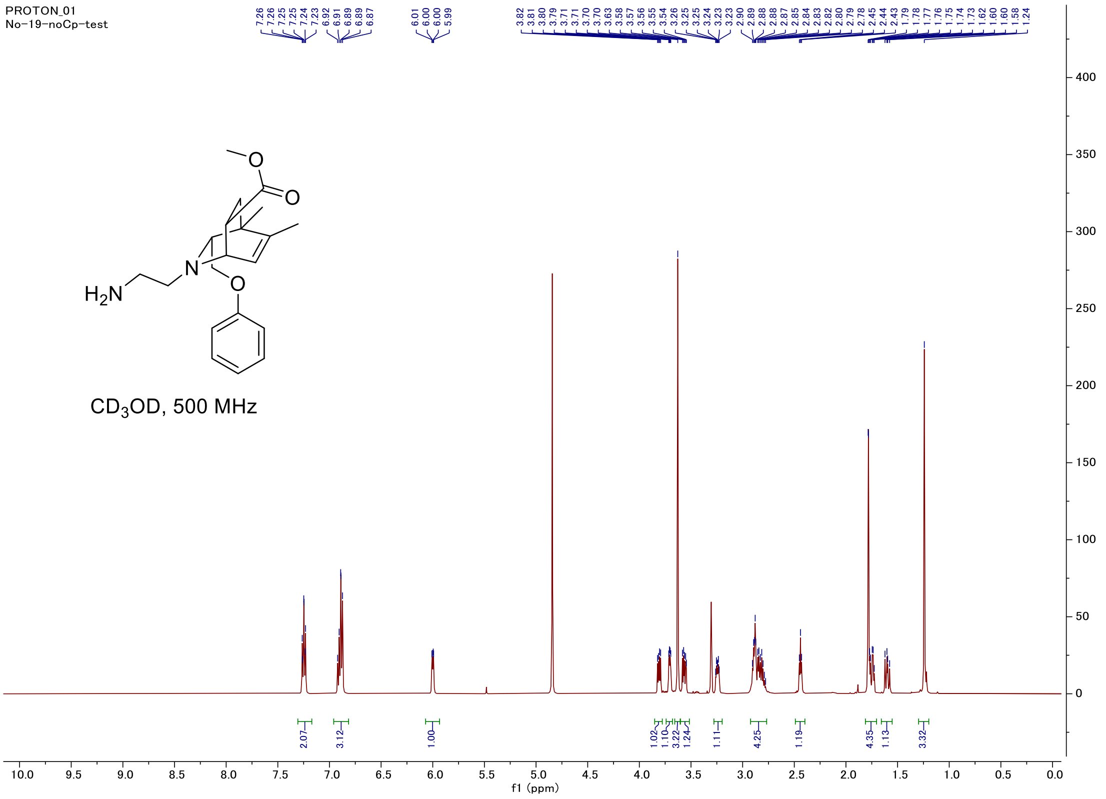

Carbon

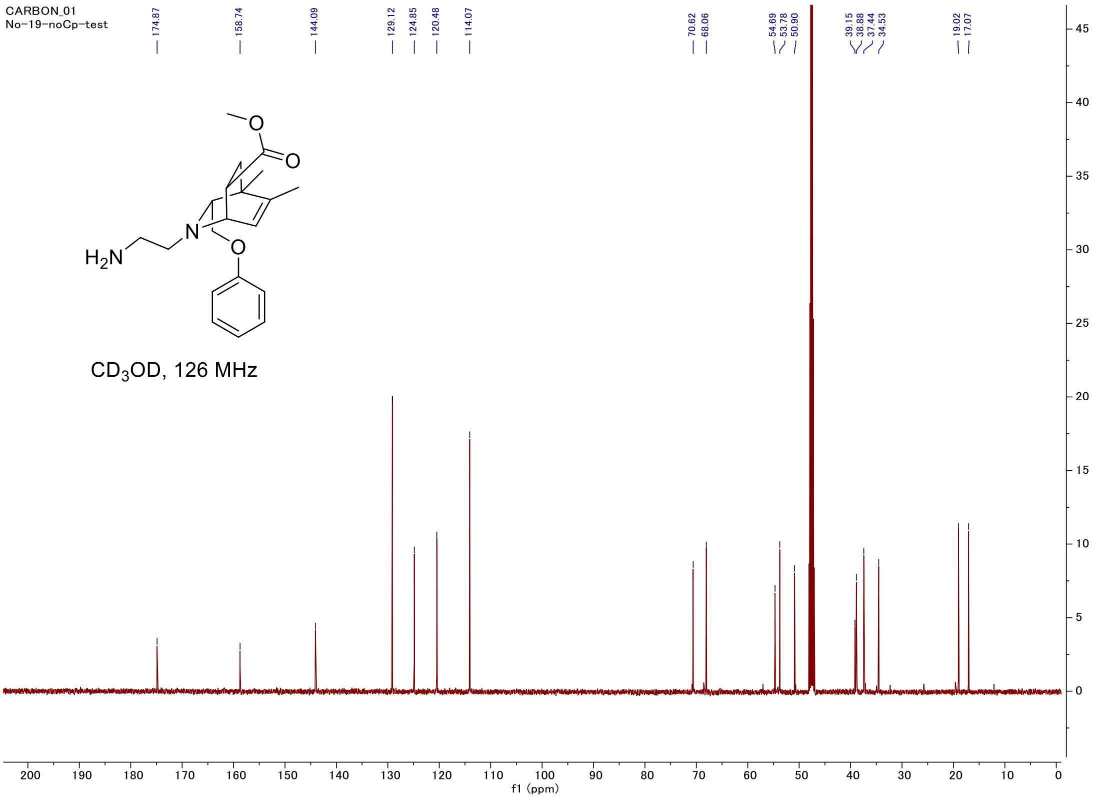

C-H COSY

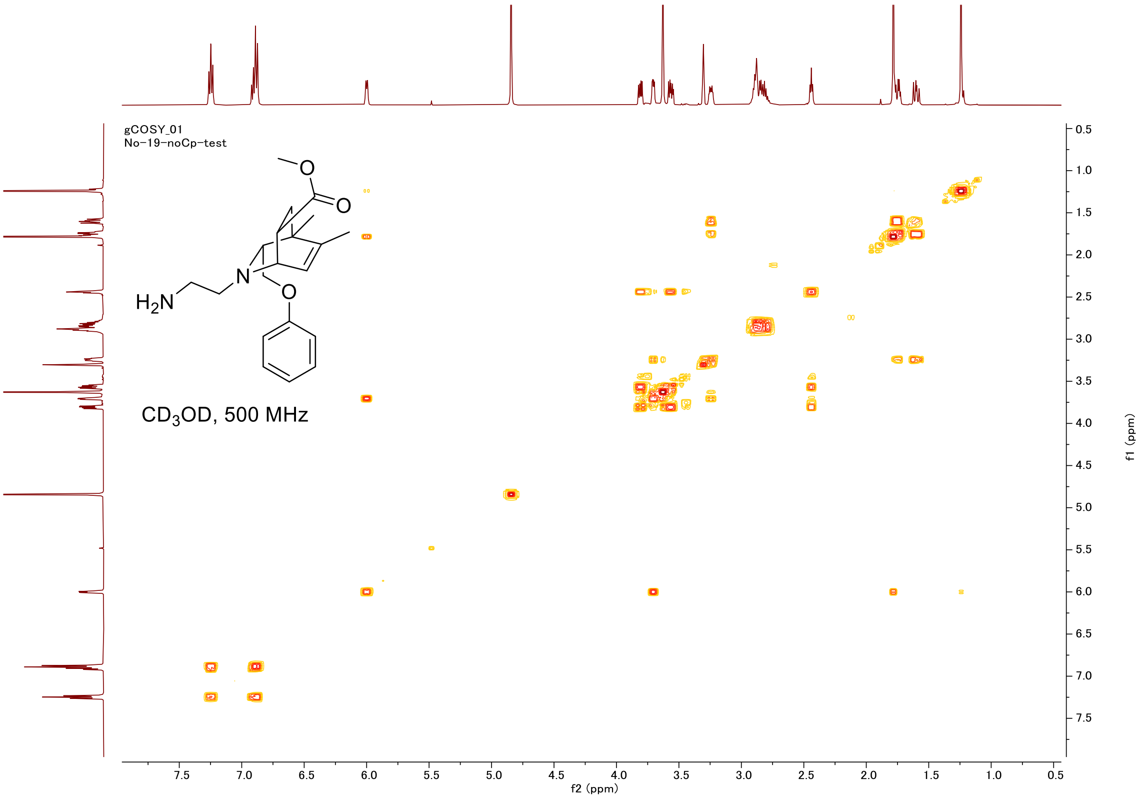
gCOSY_01
No-19-noCp-test
H2N
N
O
O
O
CD3OD, 500 MHz
f2 (ppm)
f1 (ppm)

HSQC

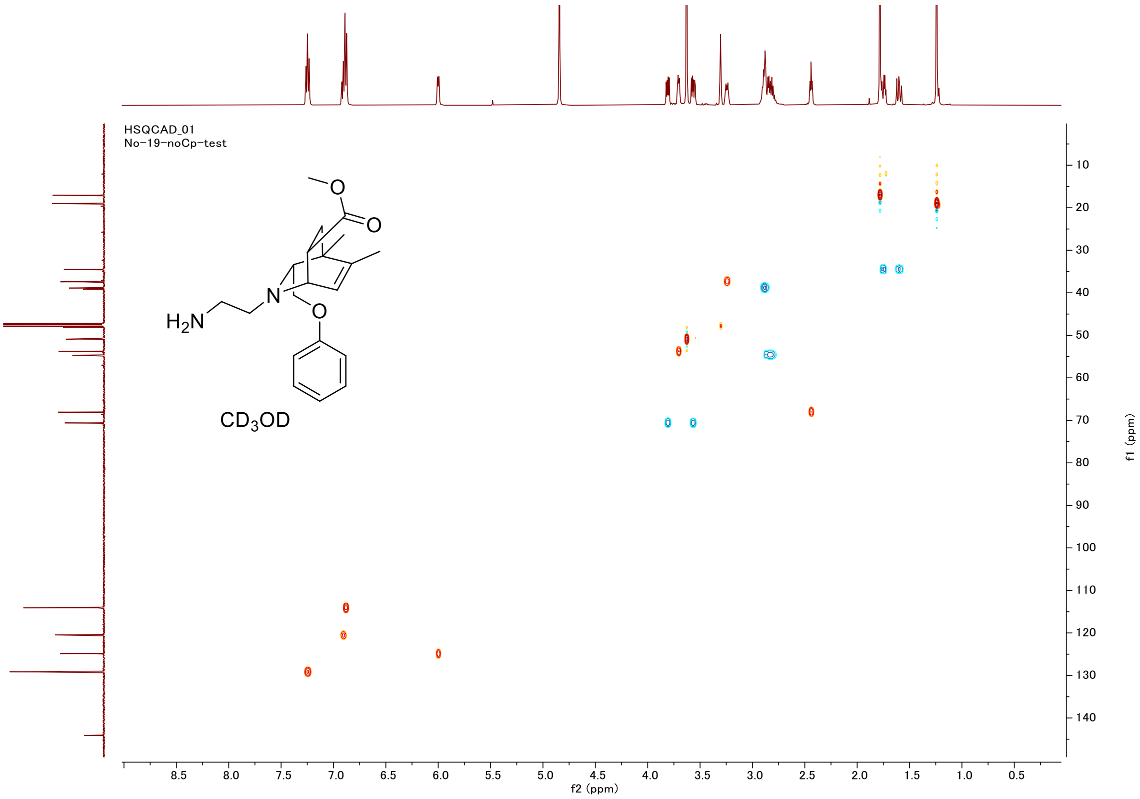
HSQCAD_01
No-19-noCp-test
H2N
N
O
O
O
CD3OD
f2 (ppm)
f1 (ppm)

Proton (#004)

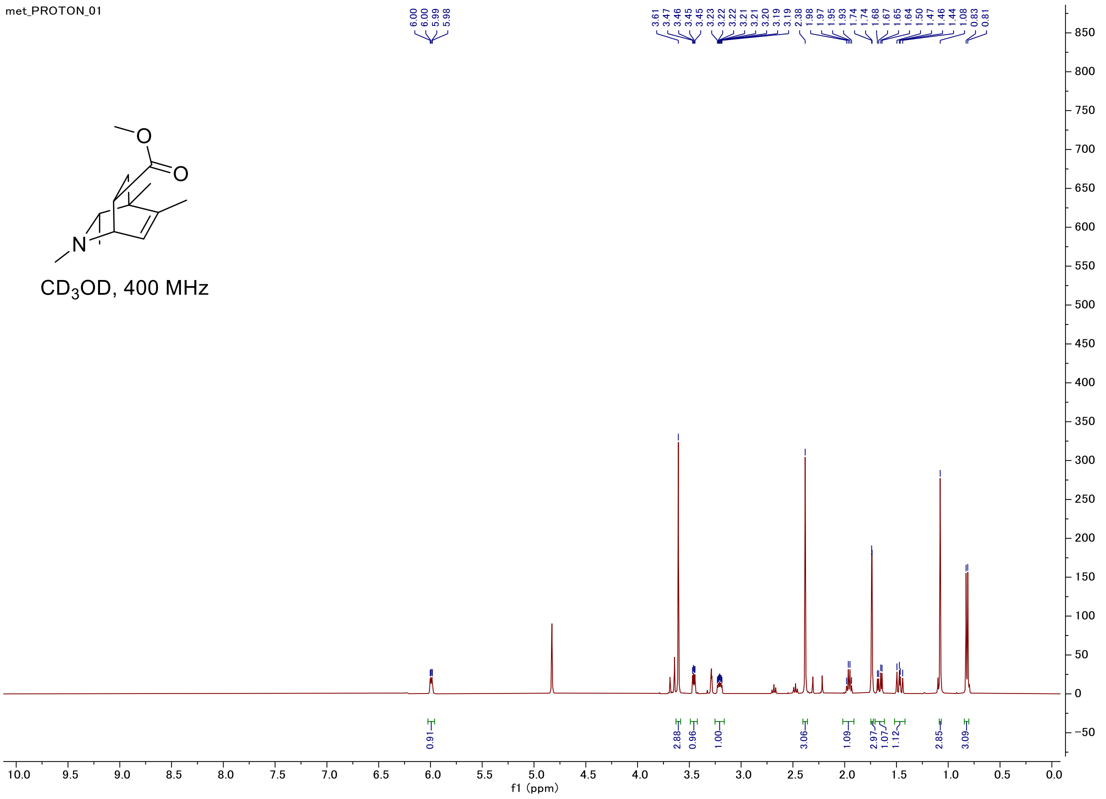

Carbon

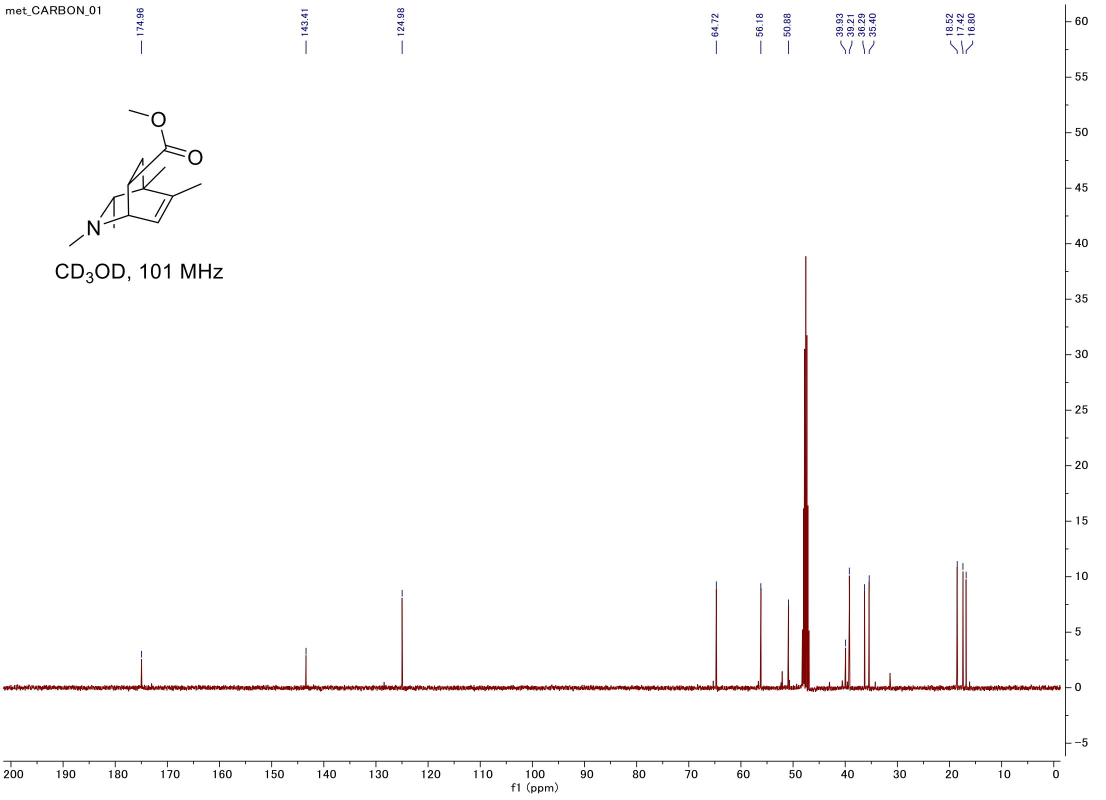

C-H COSY

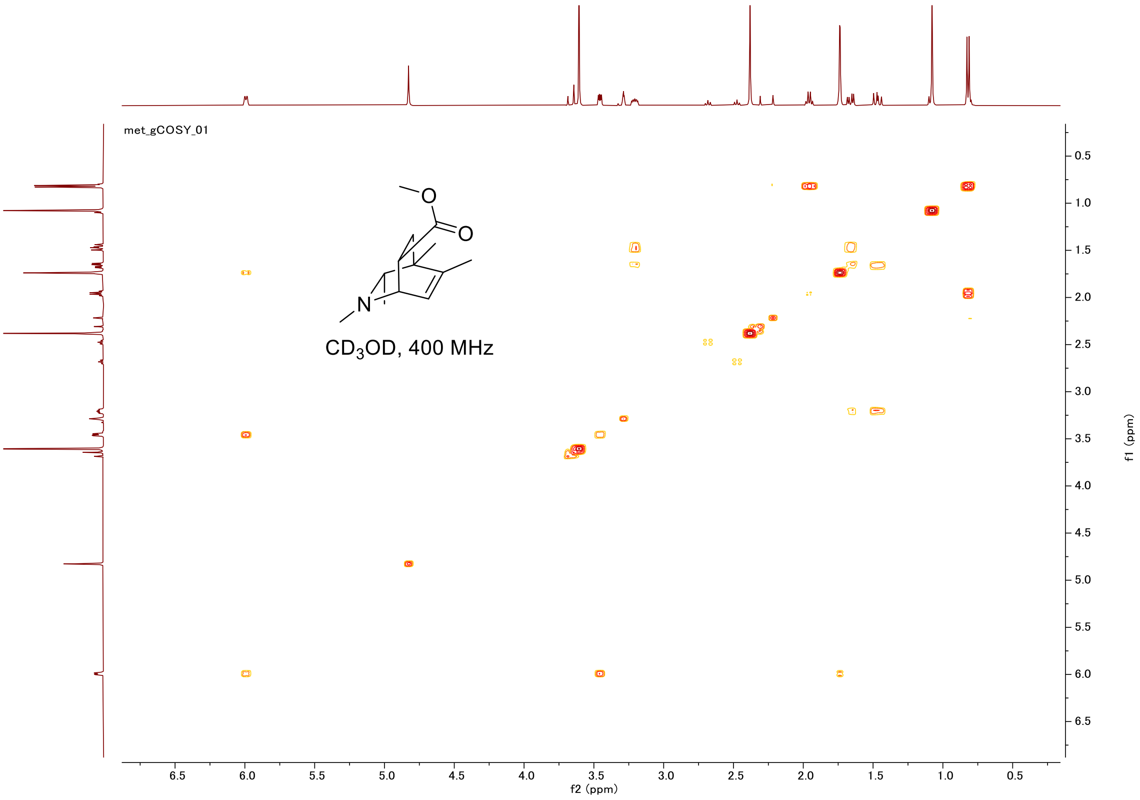
met_gCOSY_01
O
O
N
CD$_3$OD, 400 MHz
f2 (ppm)
f1 (ppm)

HSQC

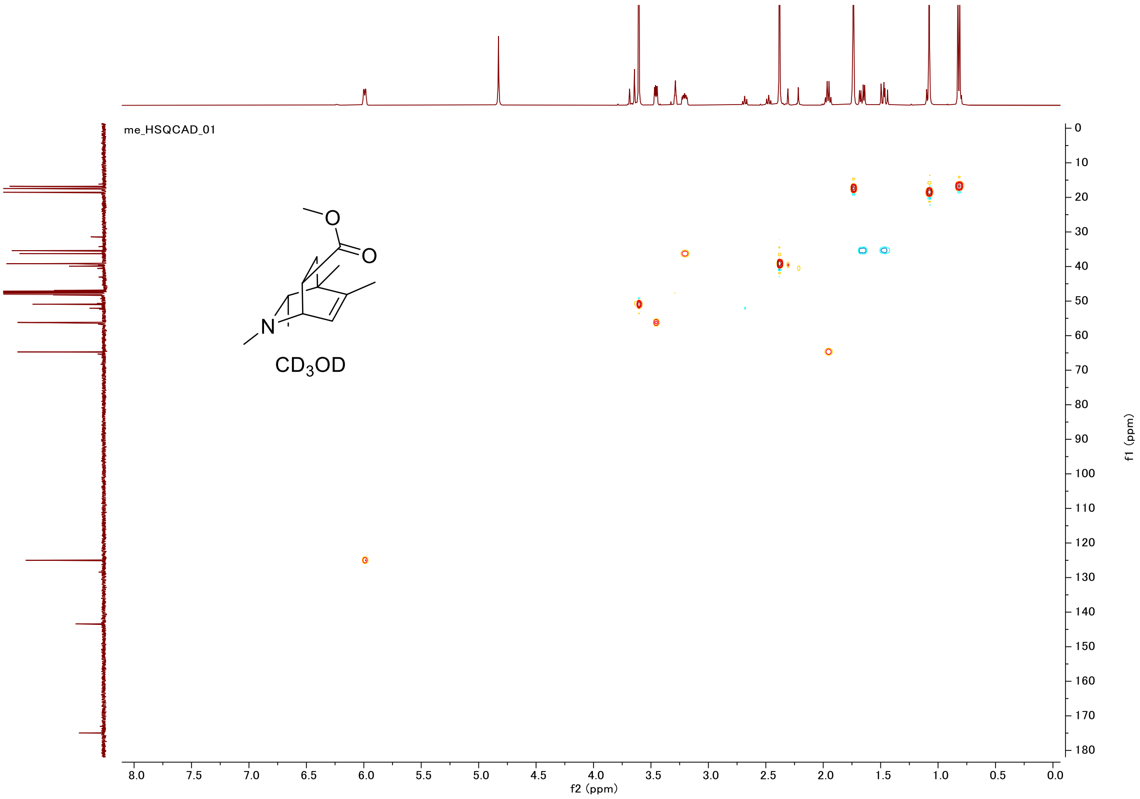
me_HSQCAD_01
O
O
N
CD$_3$OD
f2 (ppm)
f1 (ppm)

Proton (#005)

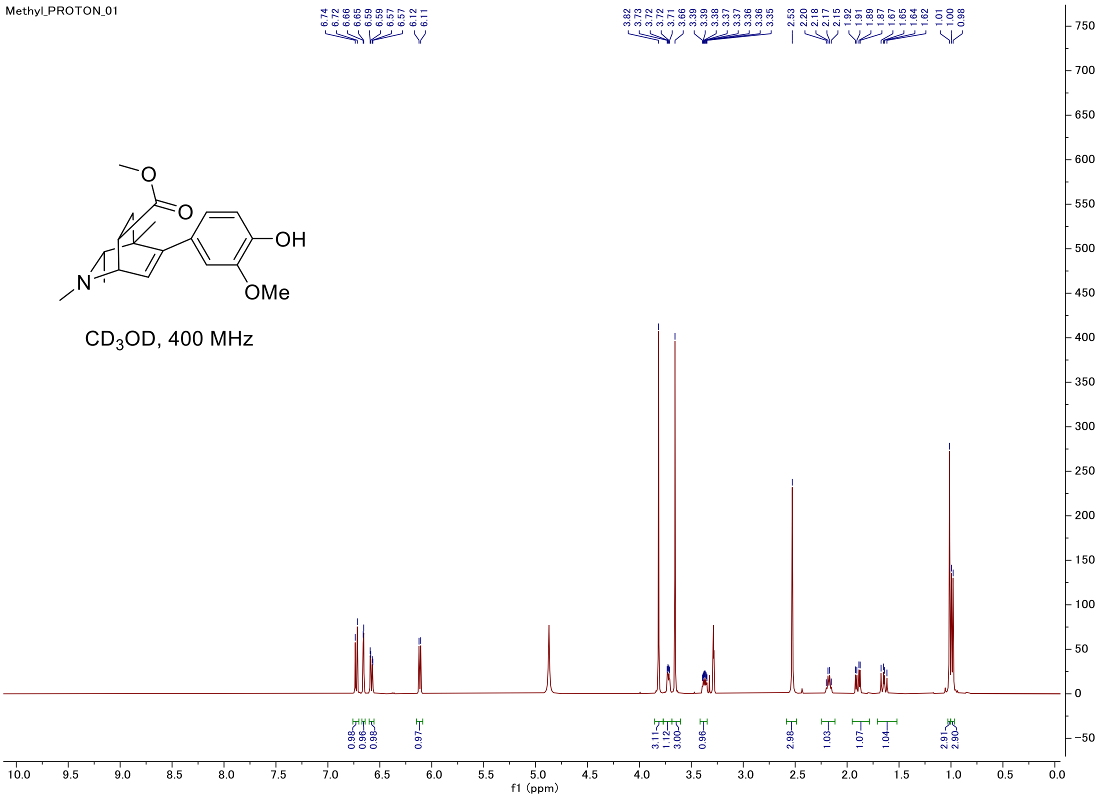

Carbon

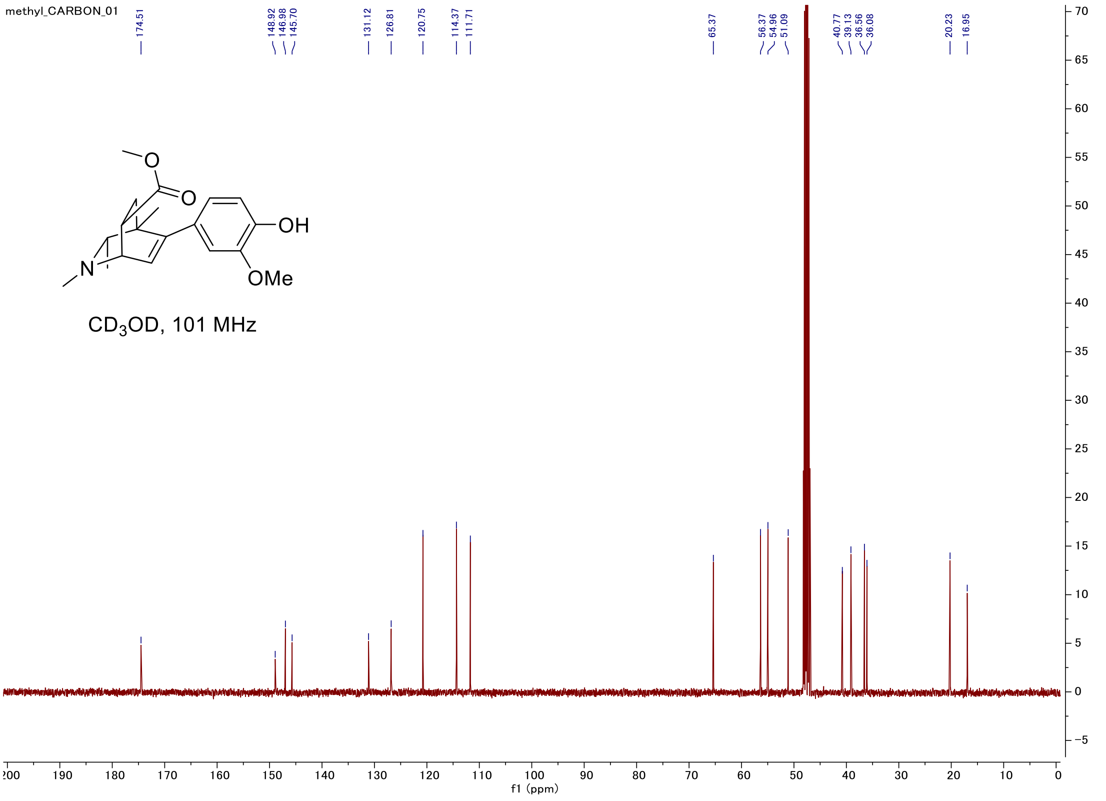

C-H COSY

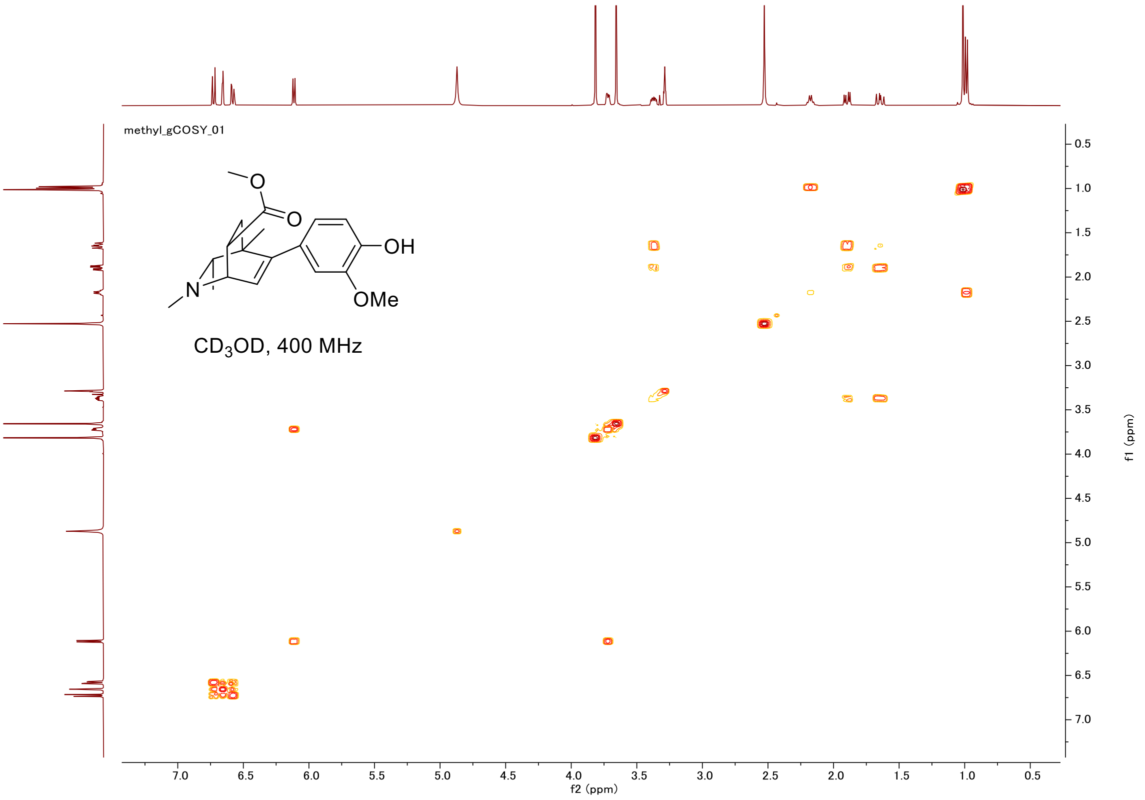
methyl_gCOSY_01
O
O
OH
N
OMe
CD3OD, 400 MHz
f2 (ppm)
f1 (ppm)

HSQC

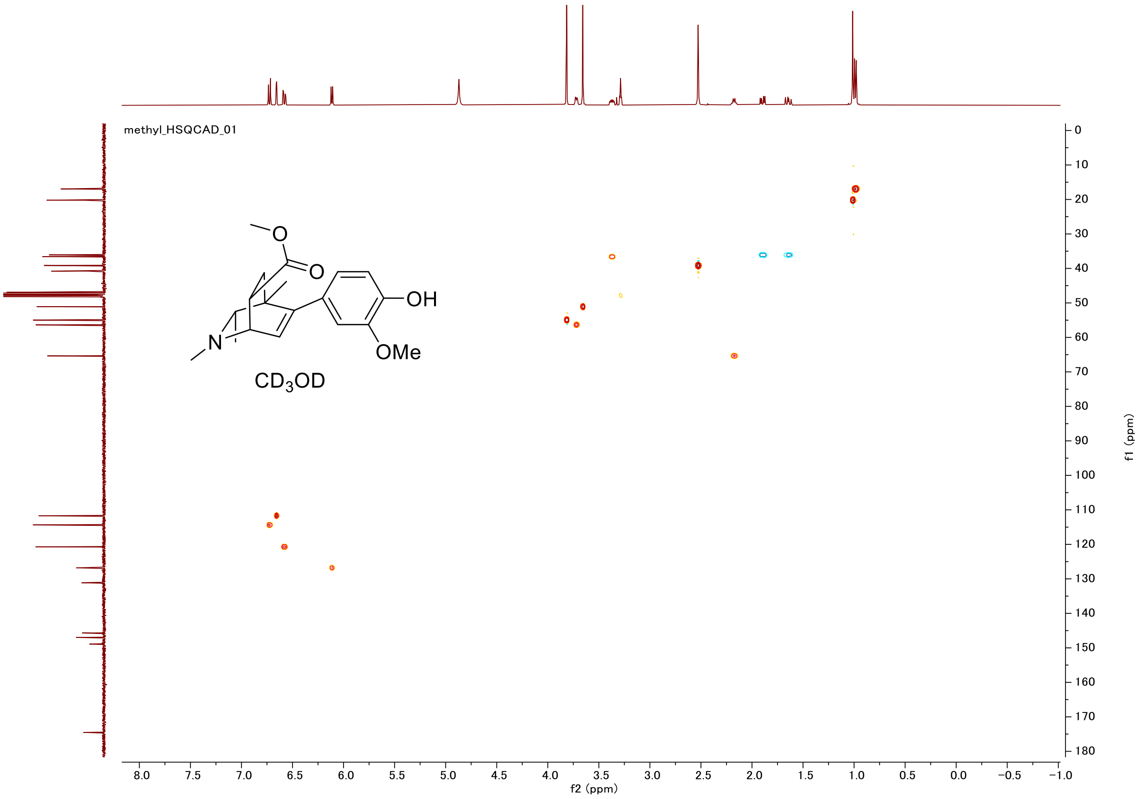
methyl_HSQCAD_01
O
O
OH
N
OMe
CD3OD
f2 (ppm)
f1 (ppm)

Proton (#006)

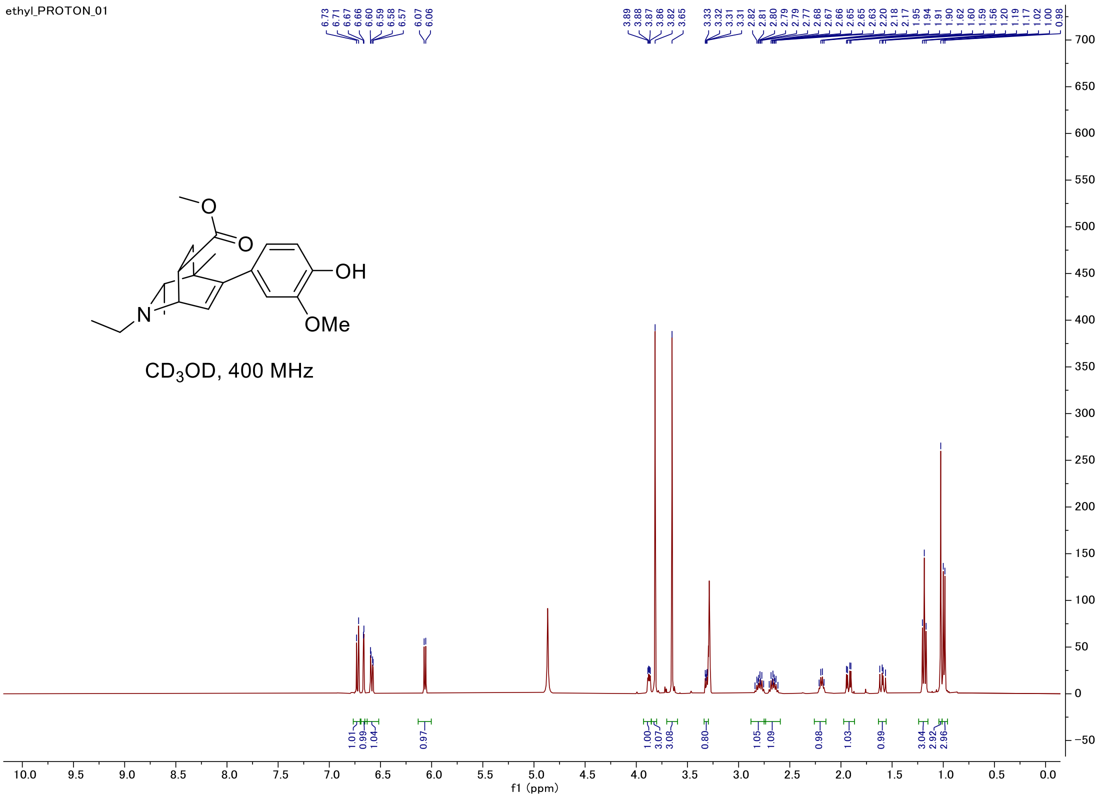

Carbon

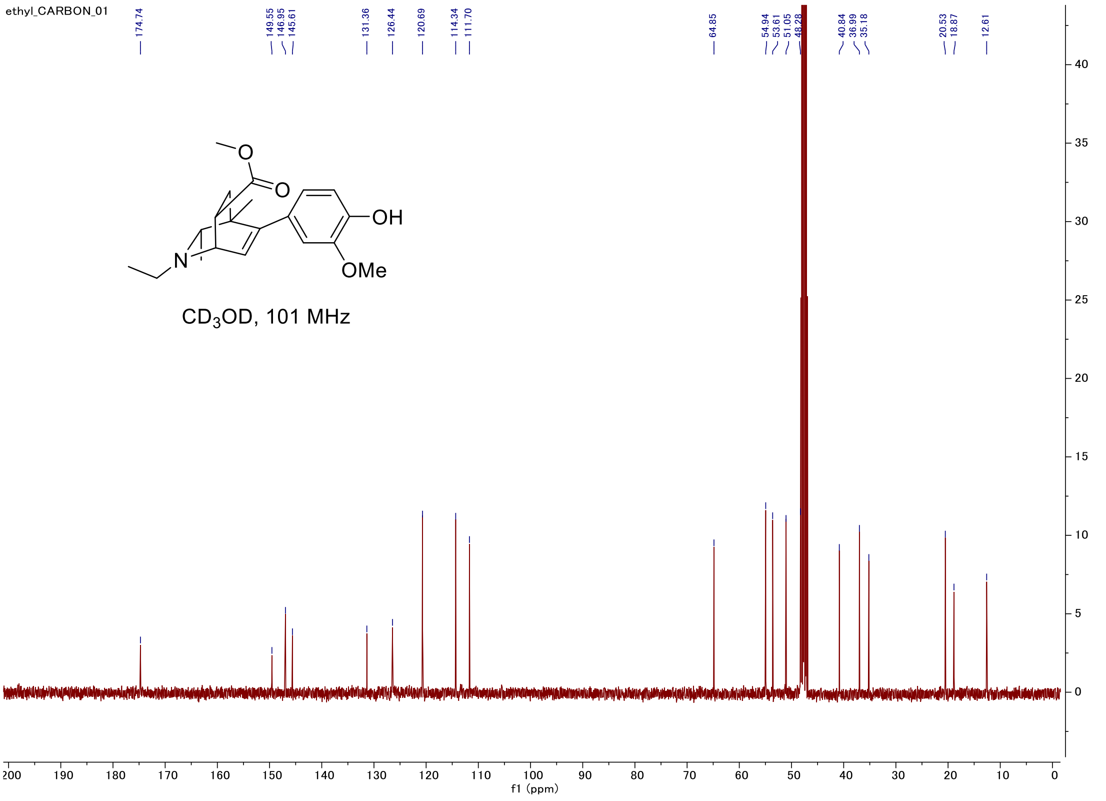

C-H COSY

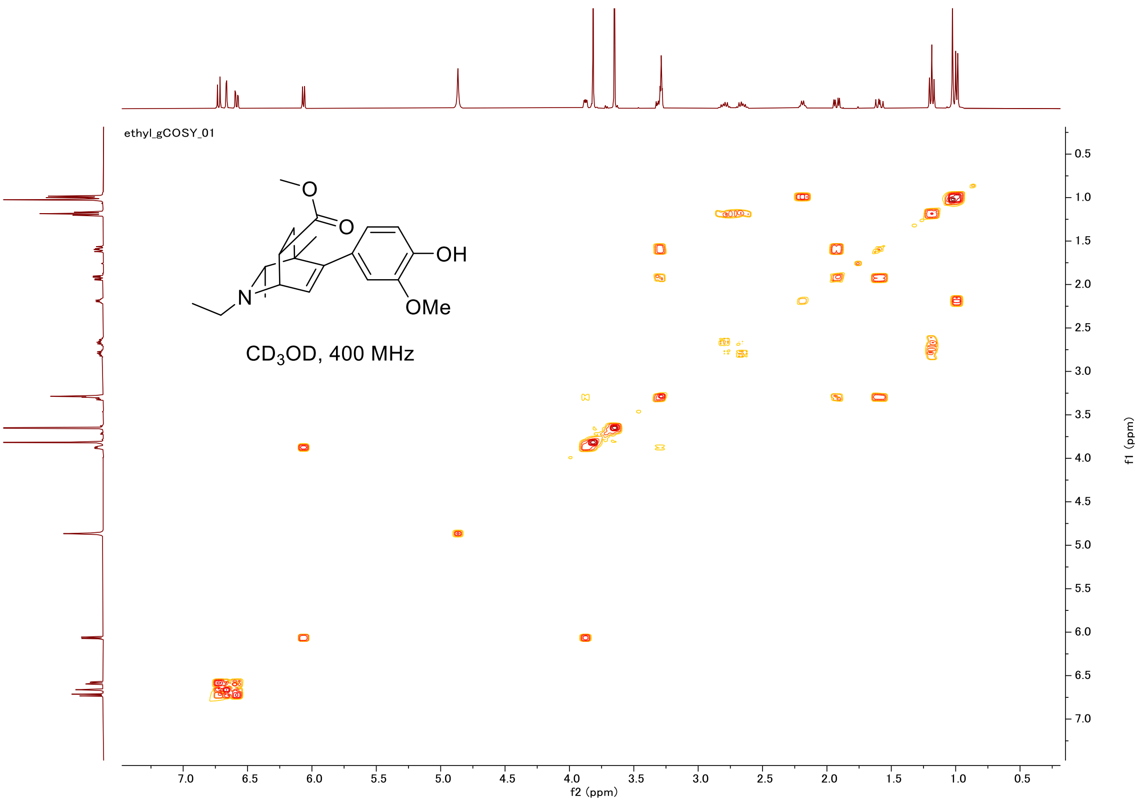
ethyl_gCOSY_01
OH
OMe
N
O
O
CD3OD, 400 MHz
f2 (ppm)
f1 (ppm)

HSQC

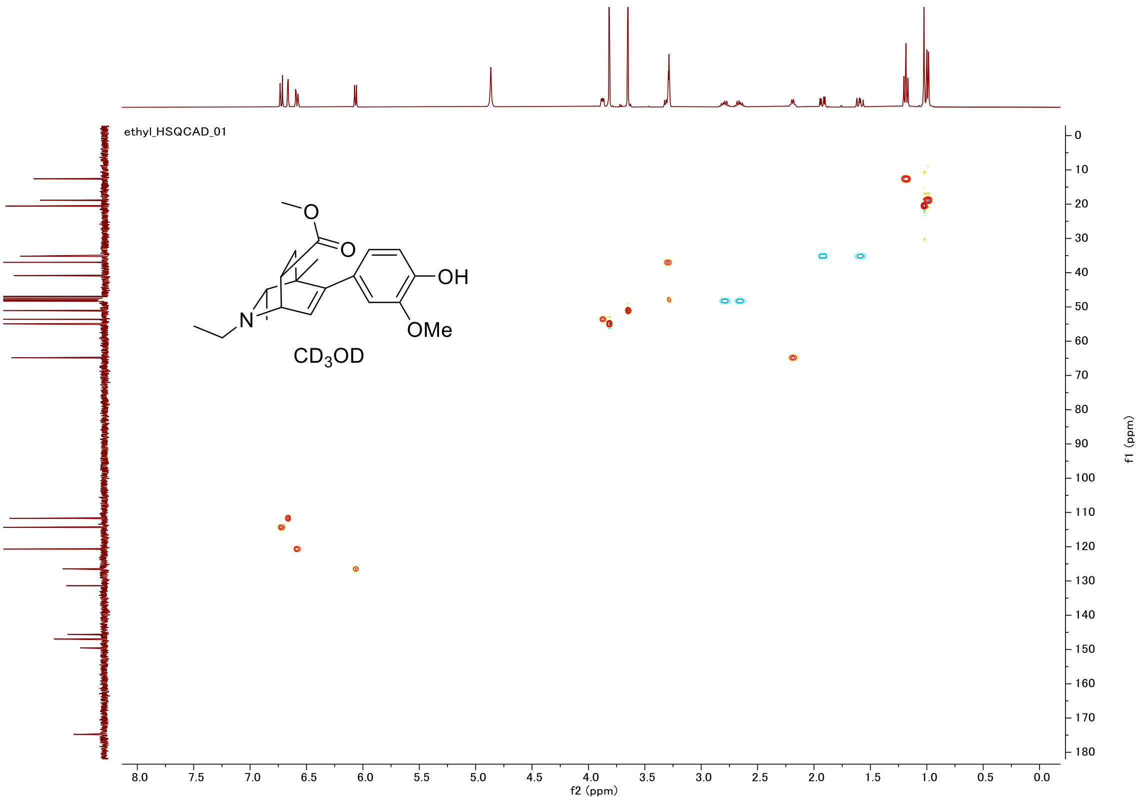
ethyl_HSQCAD_01
OH
OMe
N
O
O
CD3OD
f2 (ppm)
f1 (ppm)

Proton (#007)

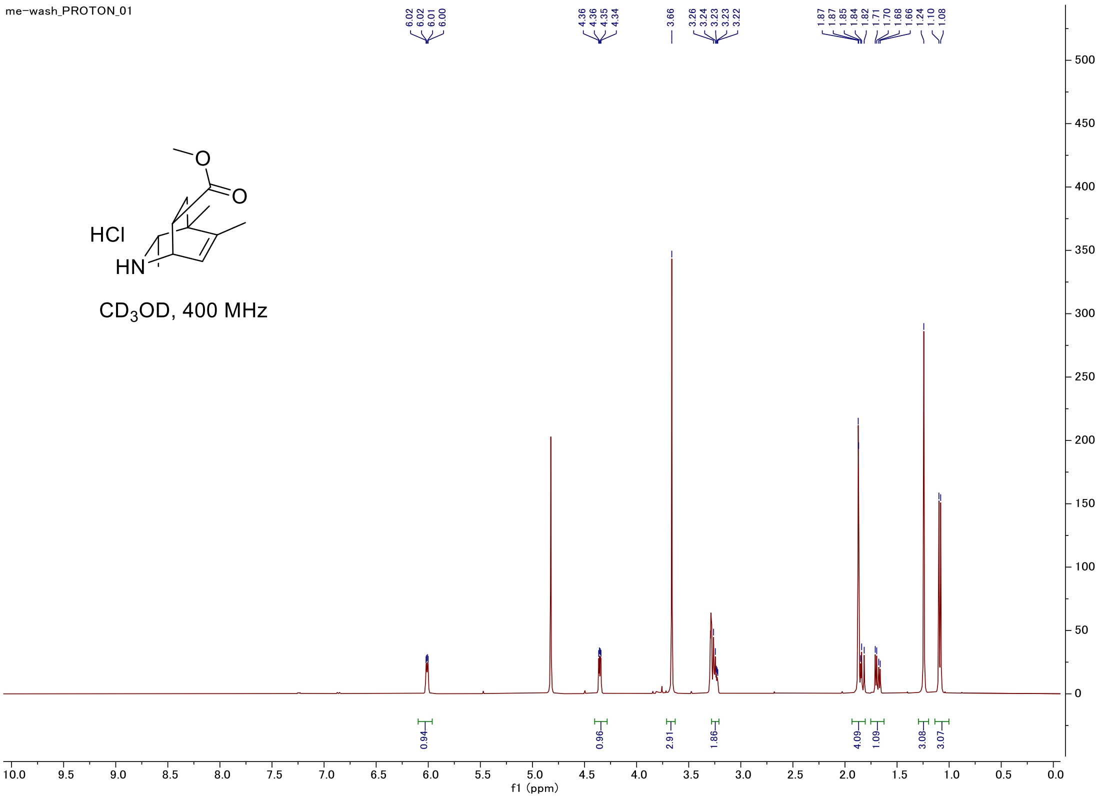

Carbon

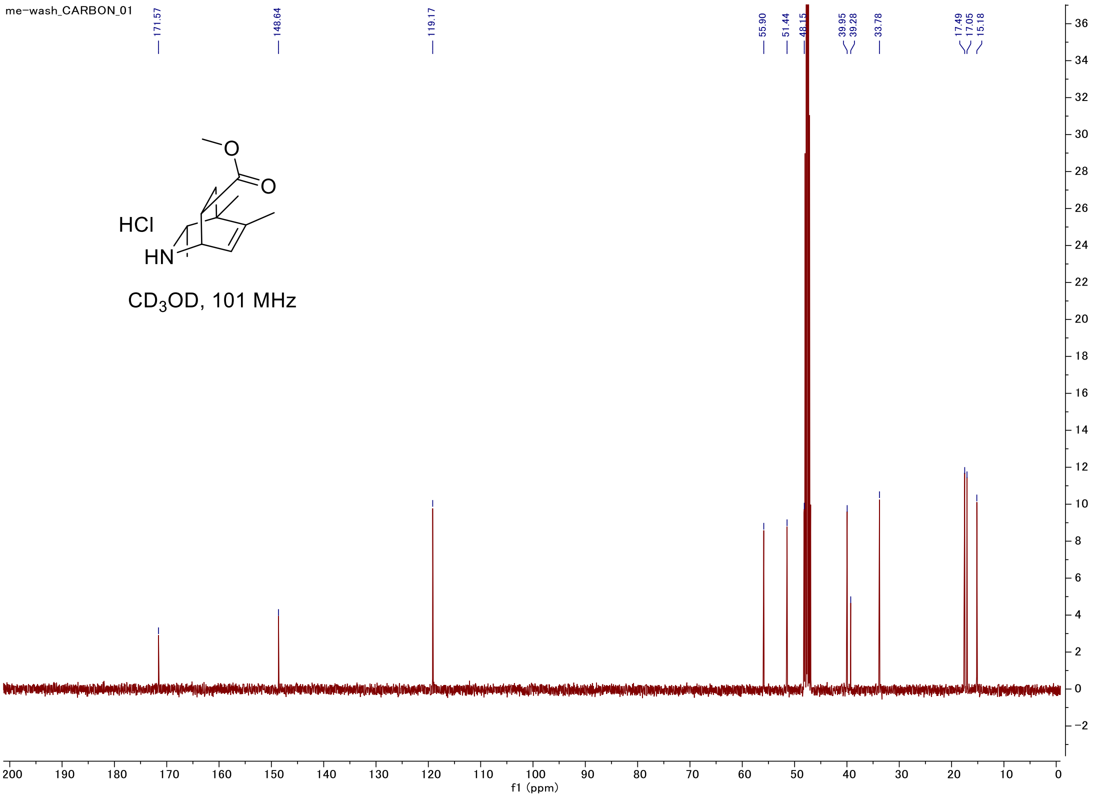

C-H COSY

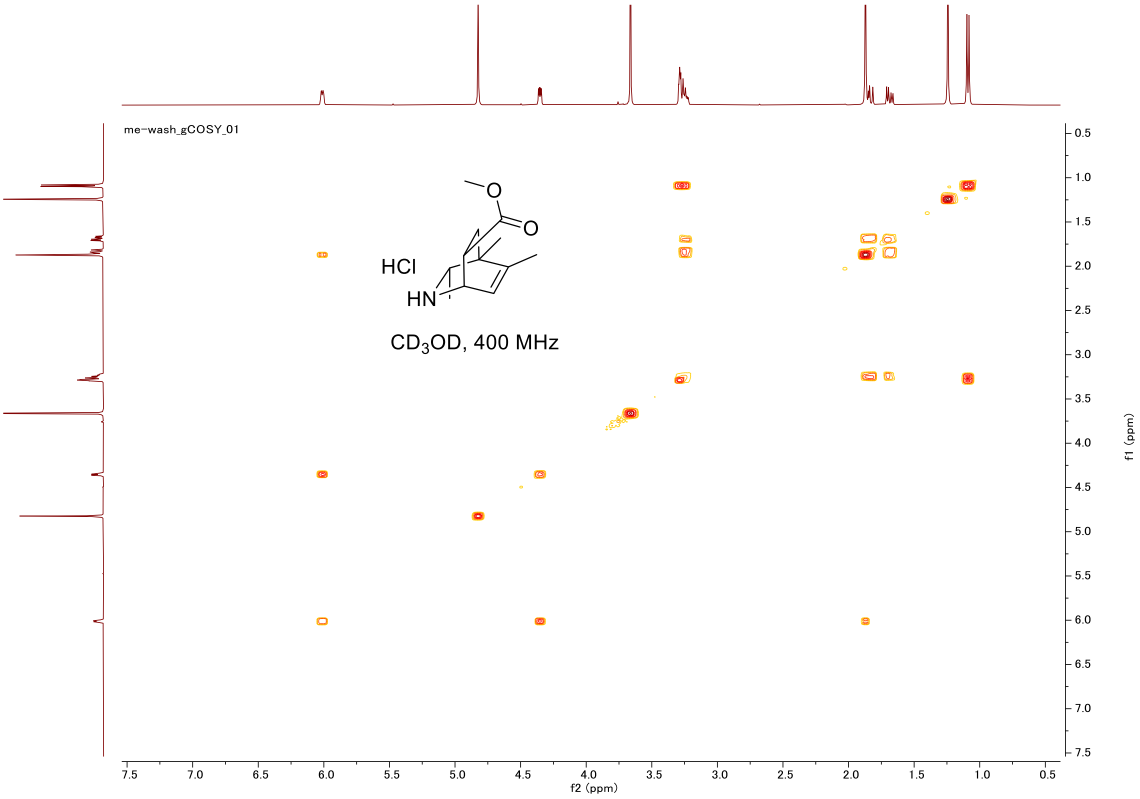
me-wash_gCOSY_01
HCl
HN
O
O
CD$_3$OD, 400 MHz
f2 (ppm)
f1 (ppm)

HSQC

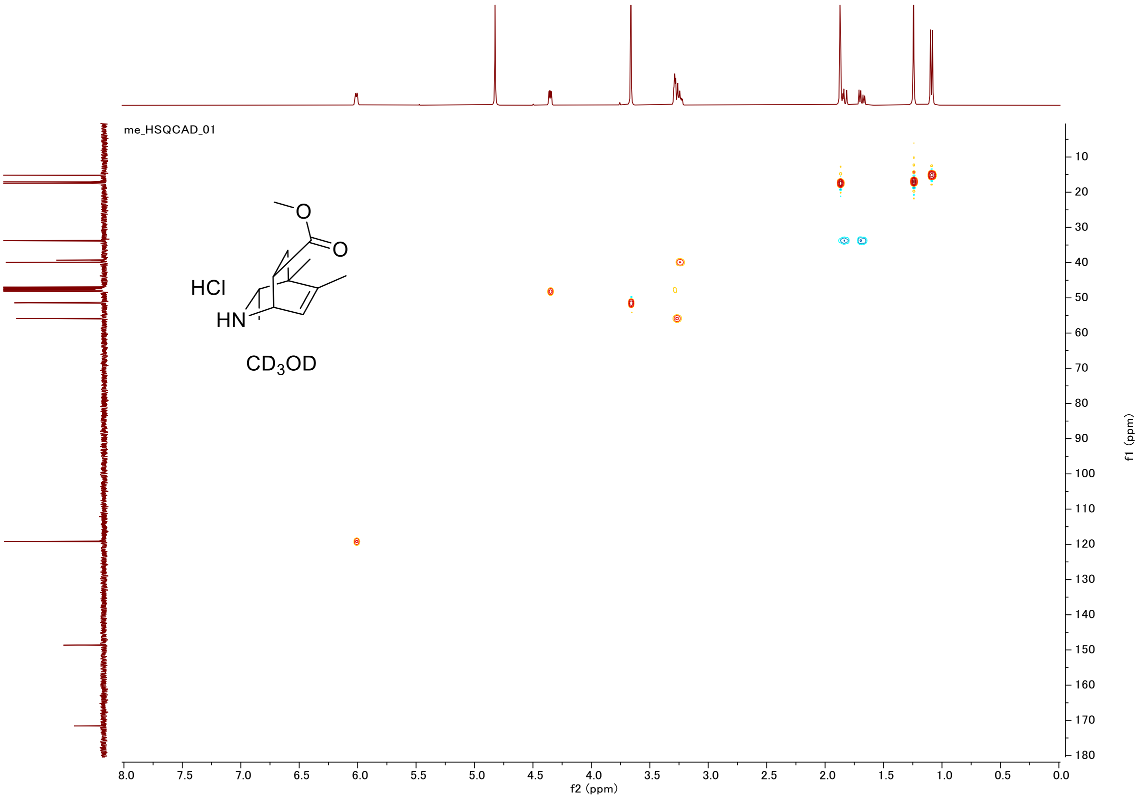
me_HSQCAD_01
HCl
HN
O
O
CD$_3$OD
f2 (ppm)
f1 (ppm)

Proton (#008)

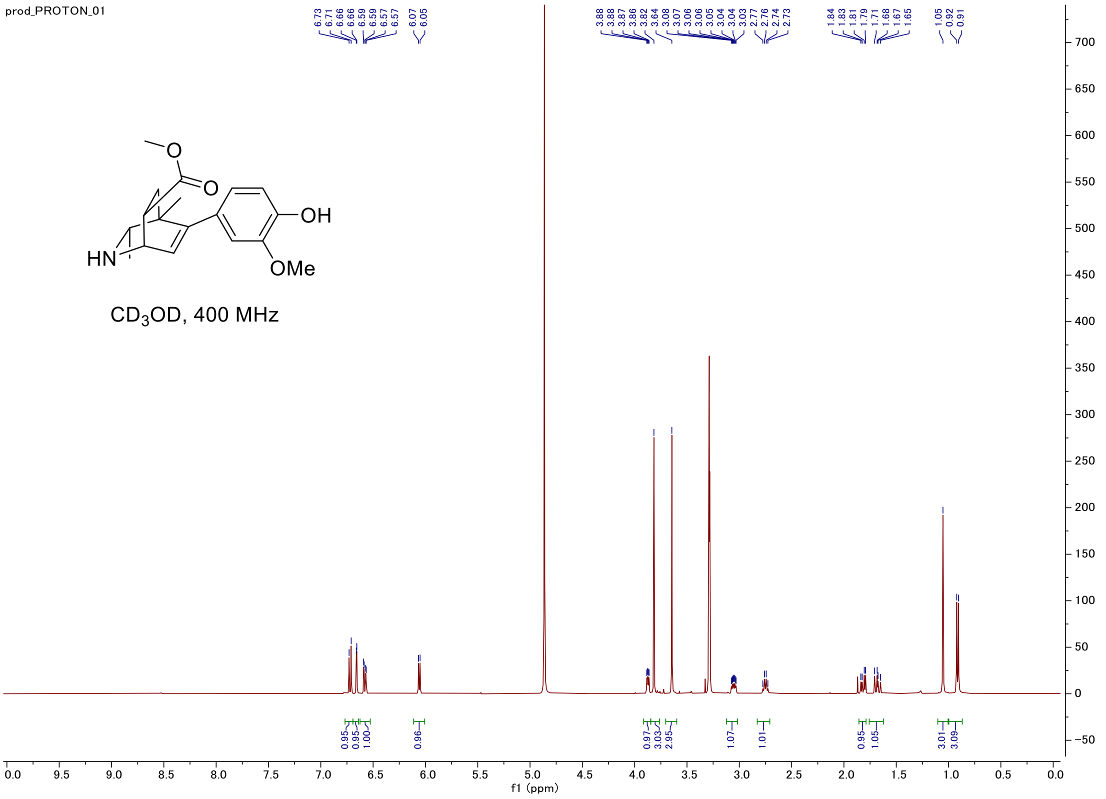

Carbon

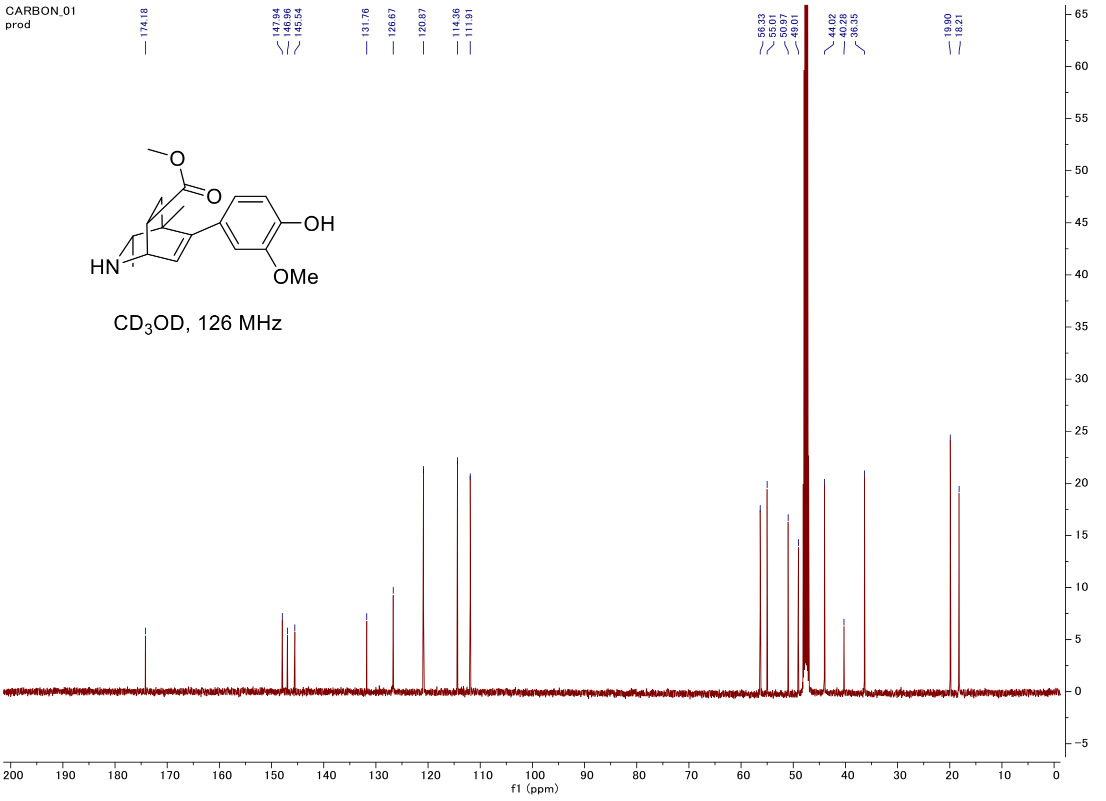

C-H COSY

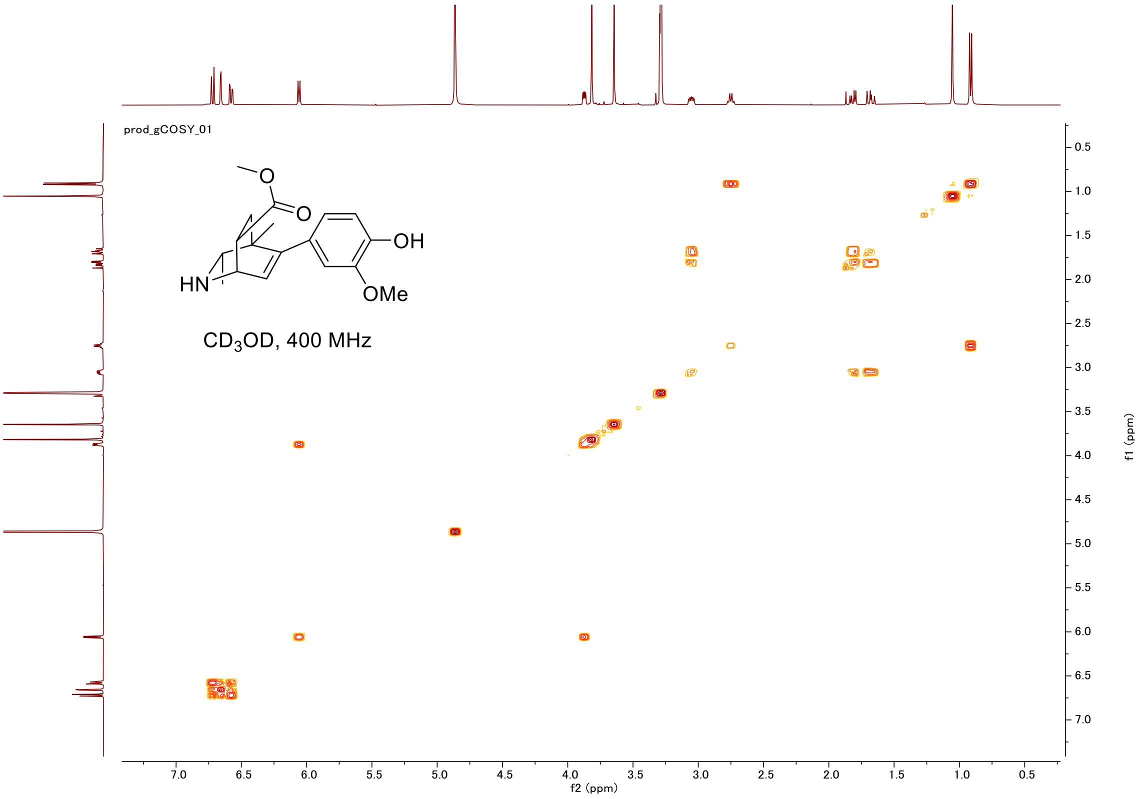
prod_gCOSY_01
OH
HN
OMe
CD3OD, 400 MHz
f2 (ppm)
f1 (ppm)

HSQC

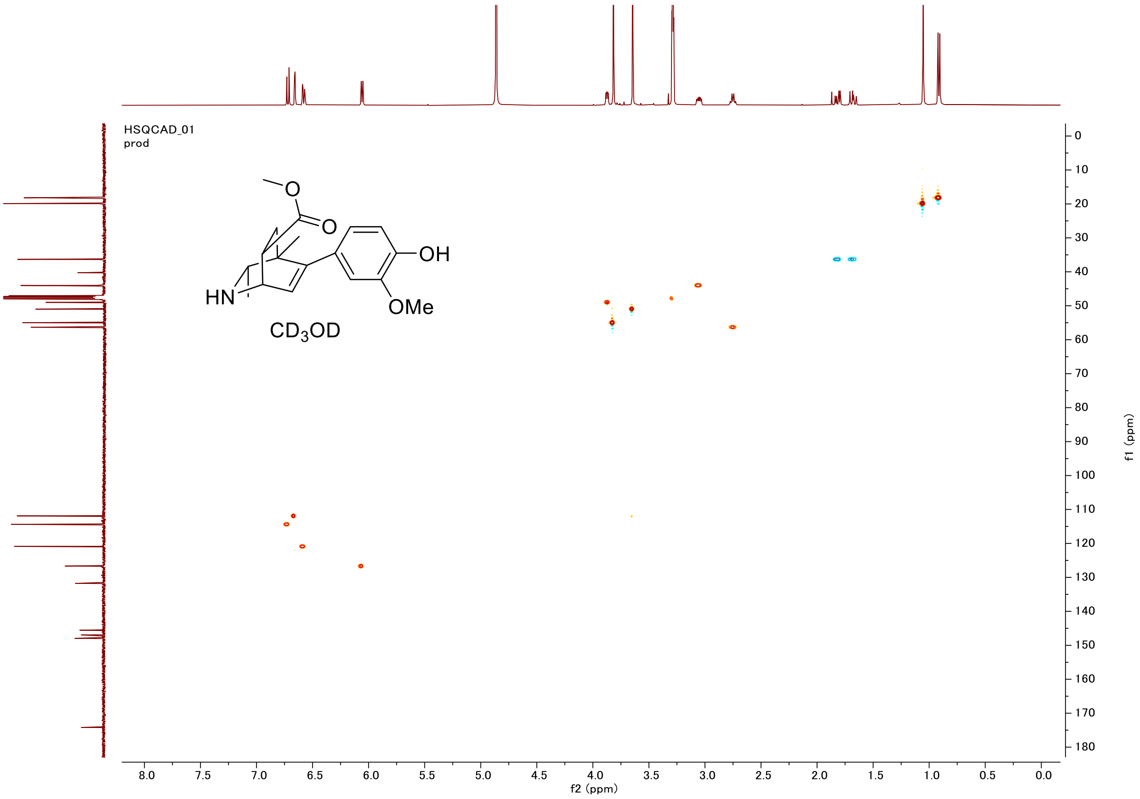
HSQCAD_01
prod
OH
HN
OMe
CD3OD
f2 (ppm)
f1 (ppm)

Proton (#009)

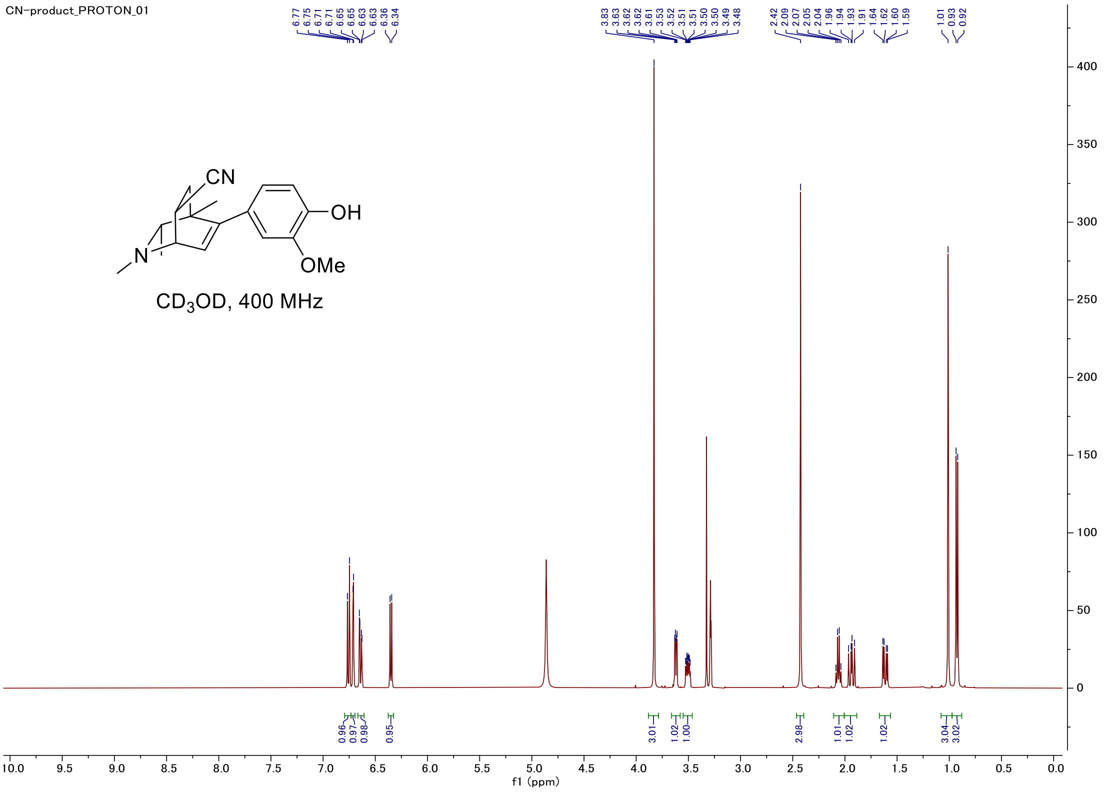

Carbon

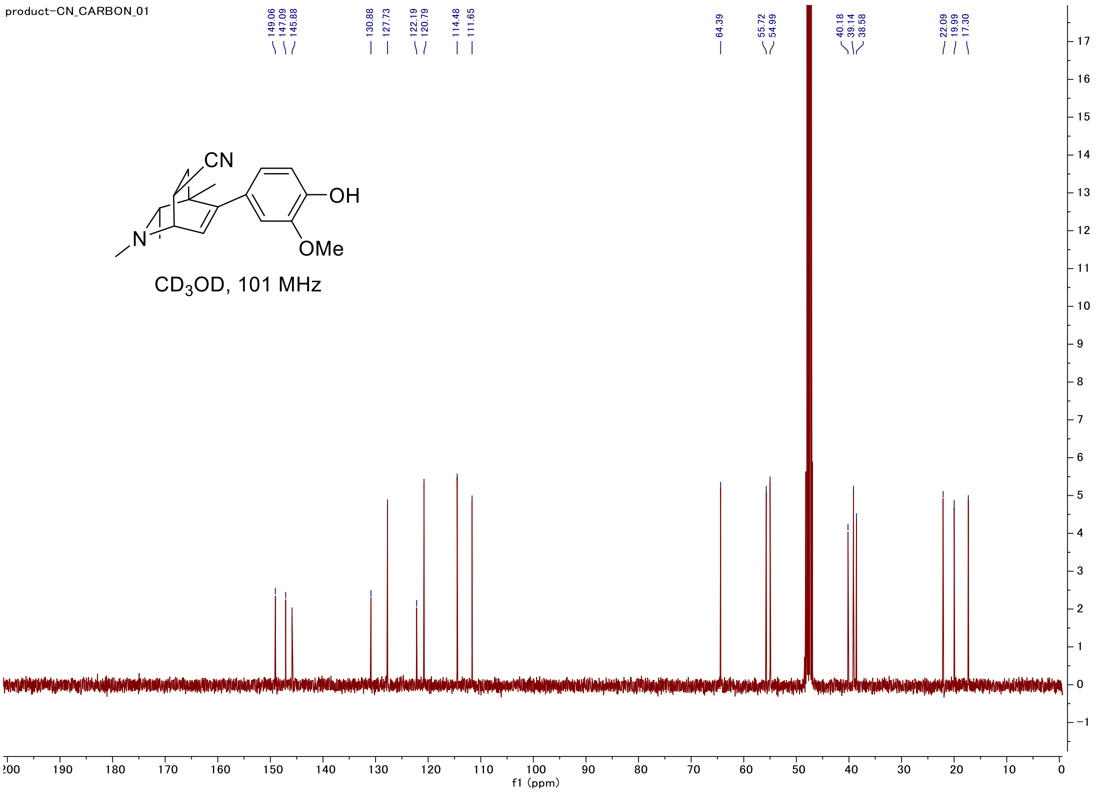

C-H COSY

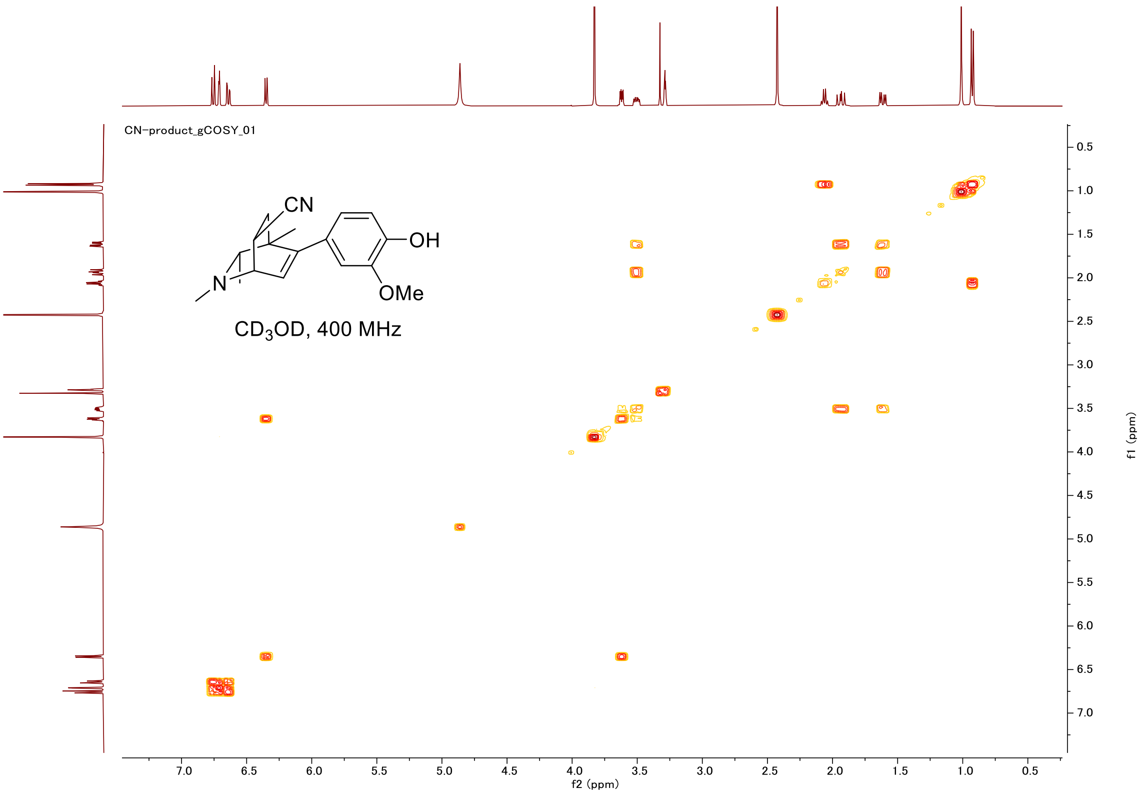

CN-product_gCOSY_01
CN
OH
N
OMe
CD3OD, 400 MHz
f2 (ppm)
f1 (ppm)

HSQC

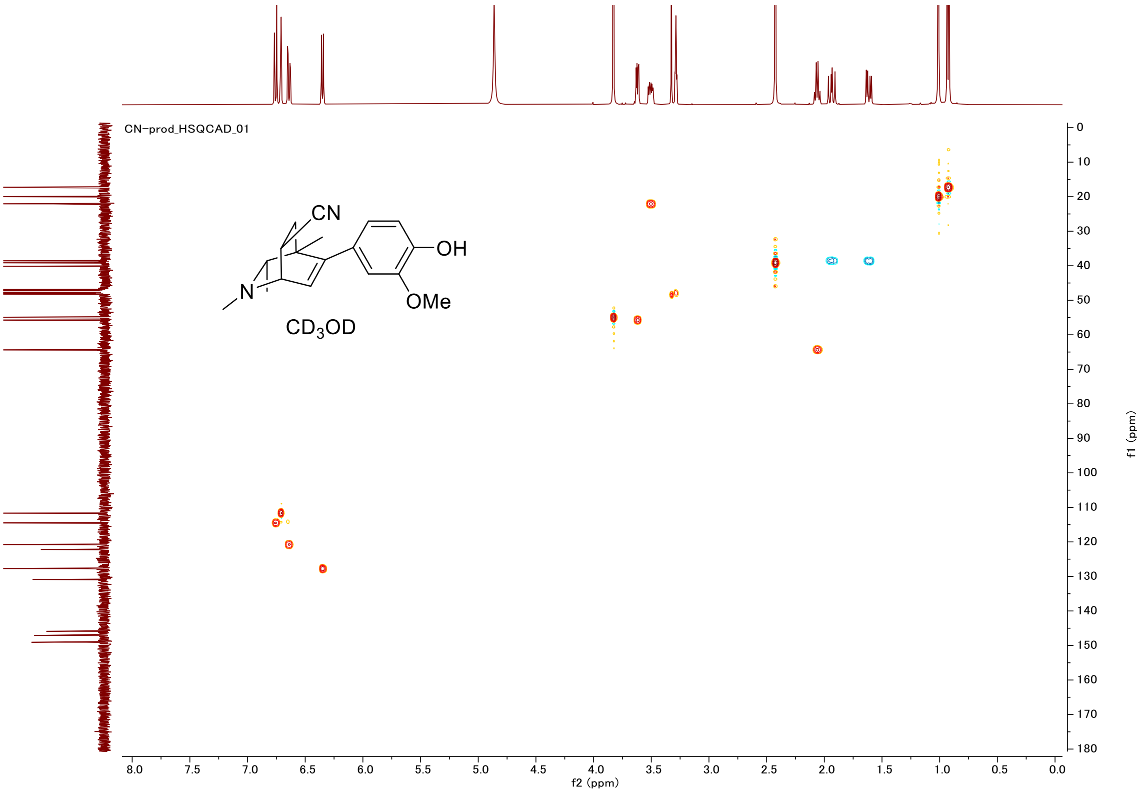

CN-prod_HSQCAD_01
CN
OH
N
OMe
CD3OD
f2 (ppm)
f1 (ppm)

Proton (#010)

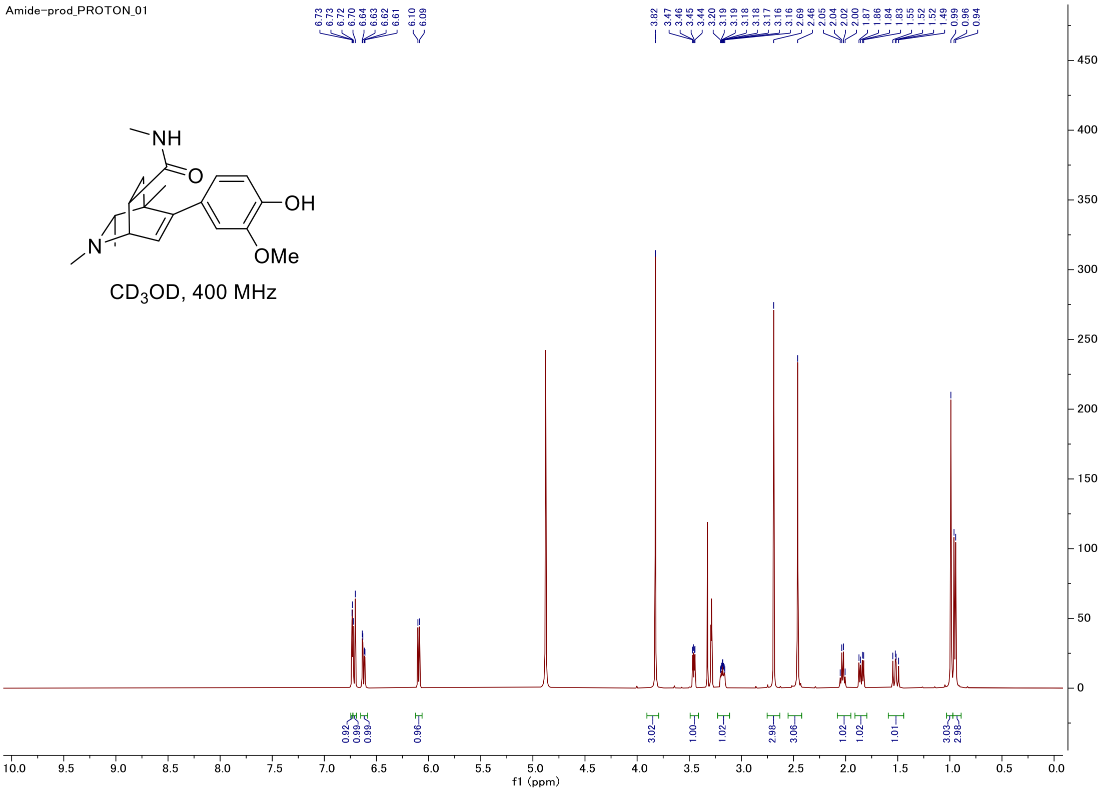

Carbon

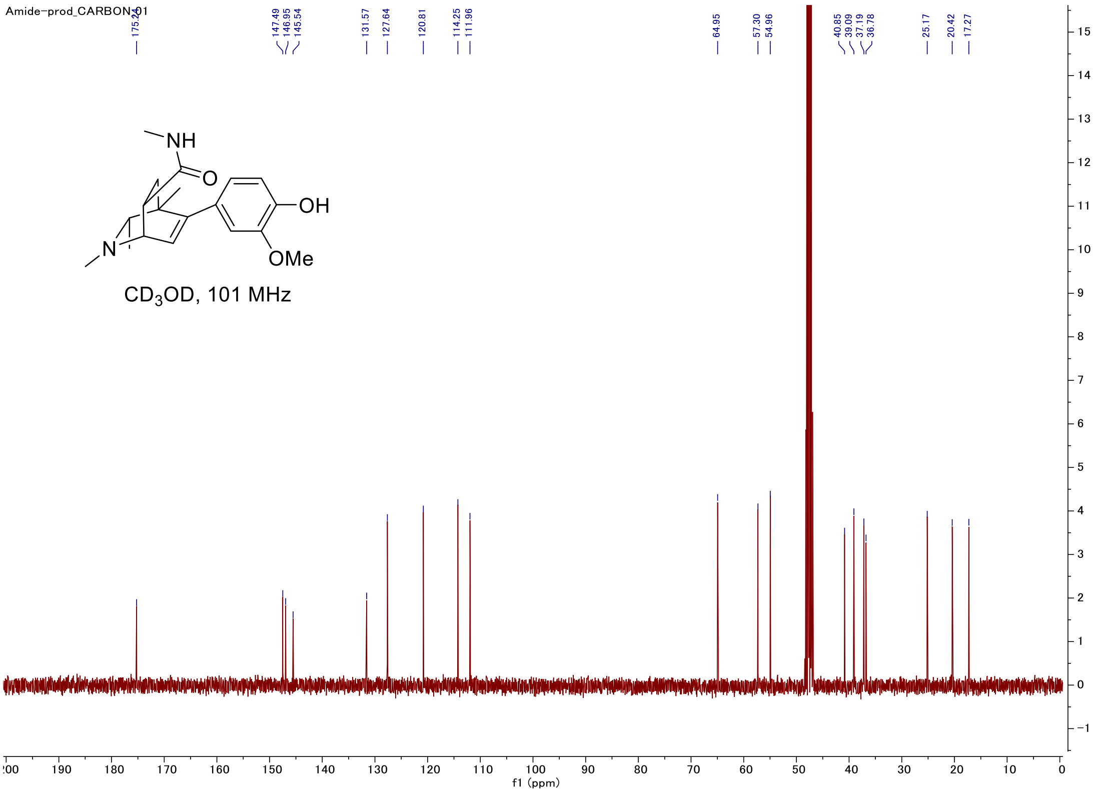

C-H COSY

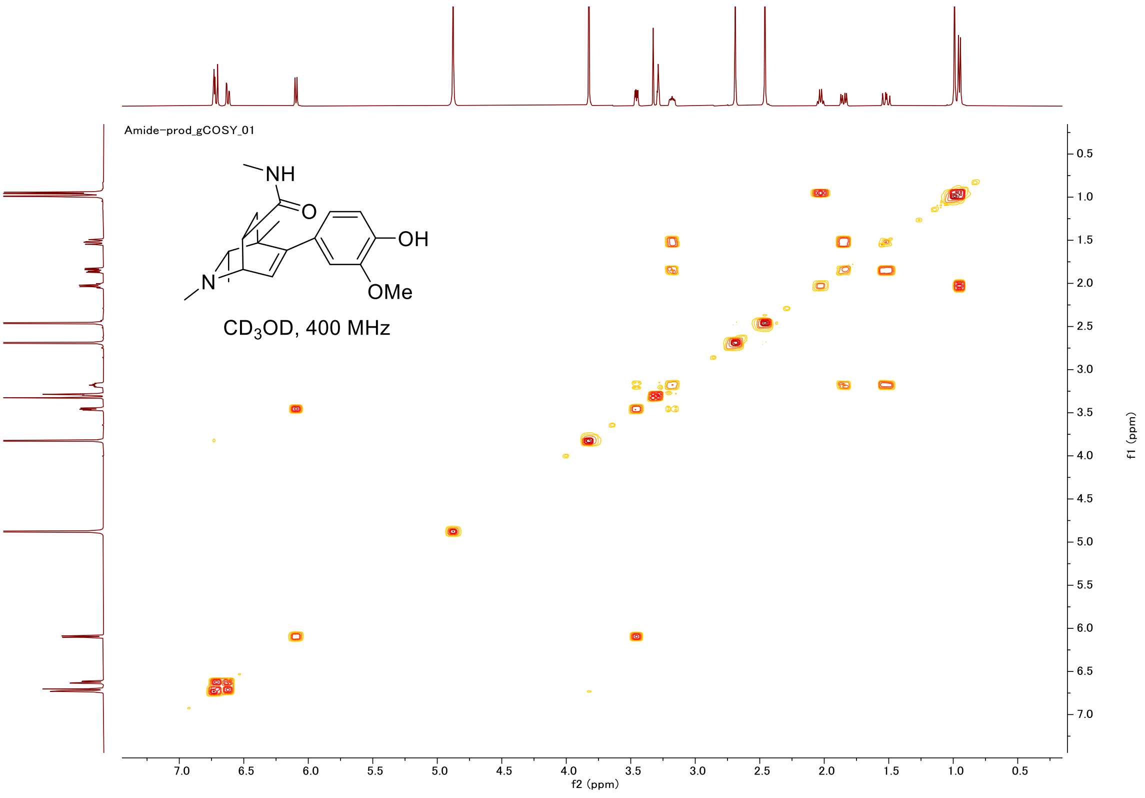
Amide-prod_gCOSY_01
NH
O
OH
N
OMe
CD3OD, 400 MHz
f2 (ppm)
f1 (ppm)

HSQC

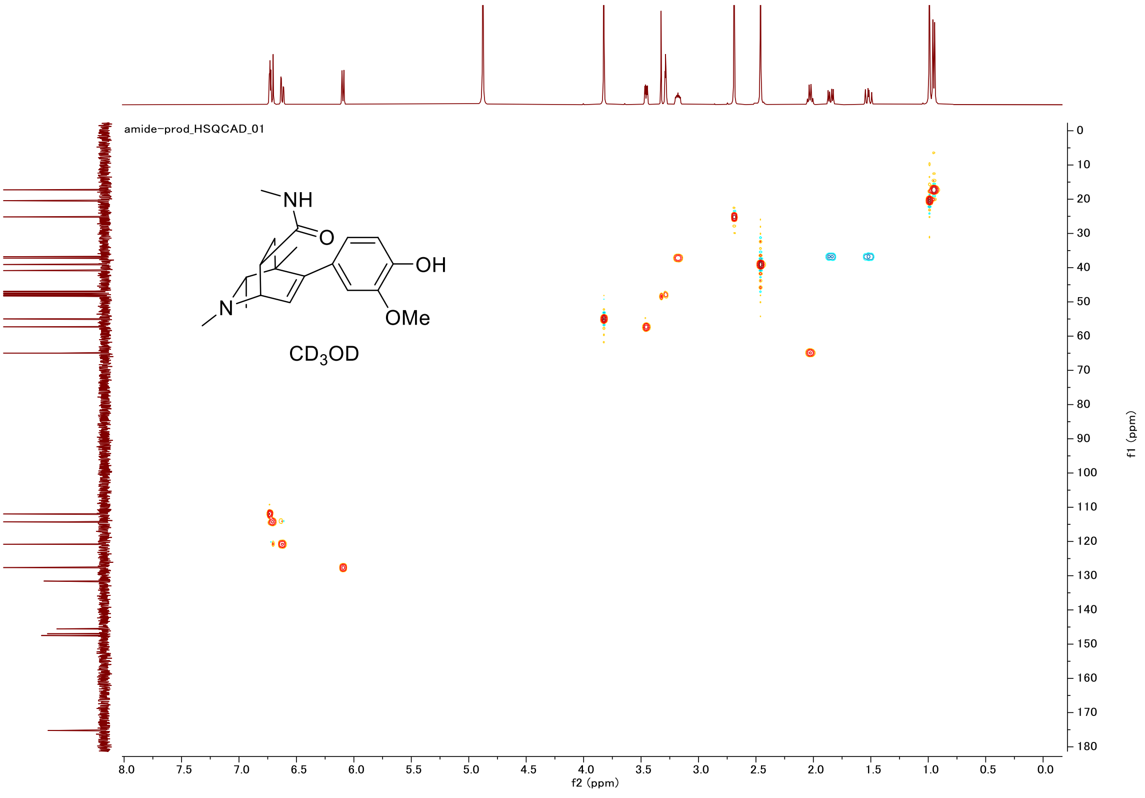
amide-prod_HSQCAD_01
NH
O
OH
N
OMe
CD3OD
f2 (ppm)
f1 (ppm)

Proton (#011)

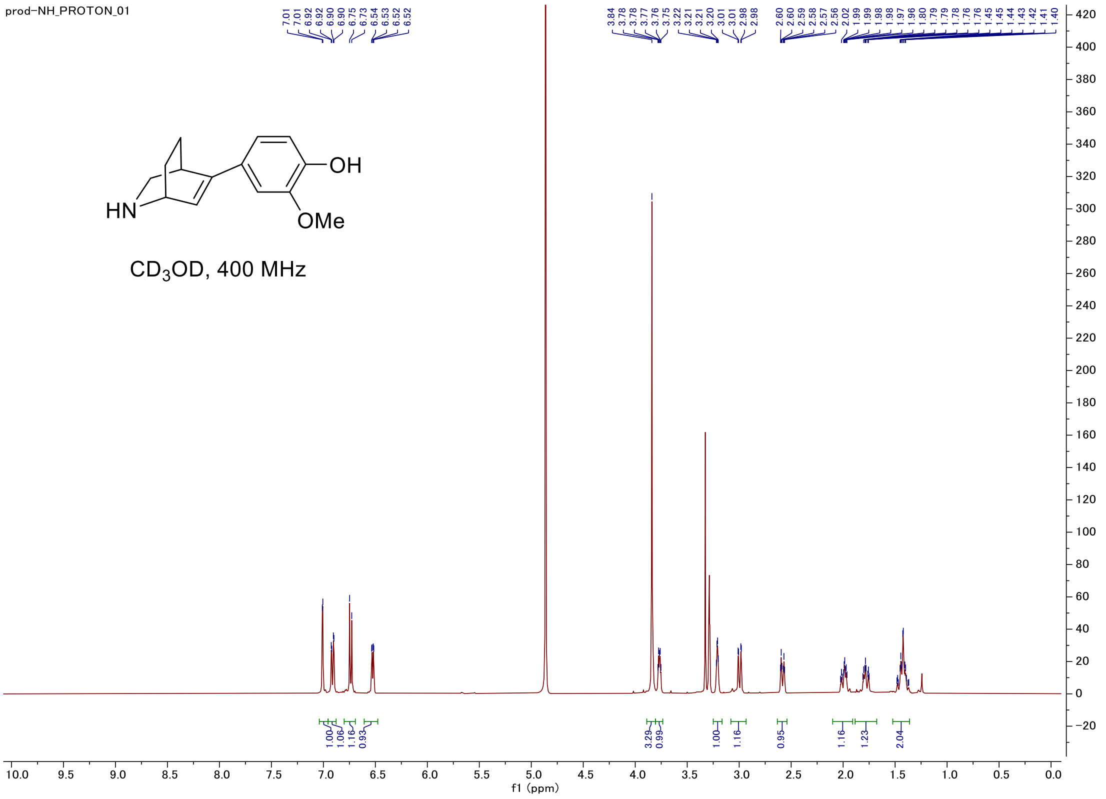

Carbon

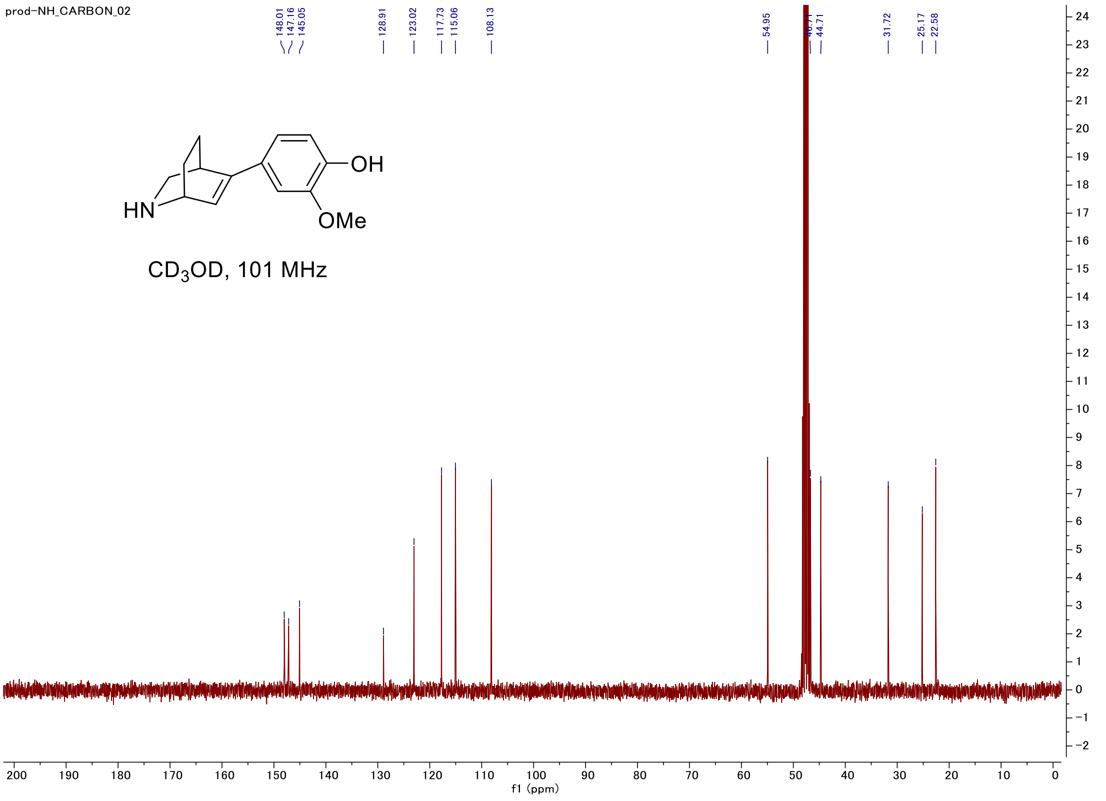

C-H COSY

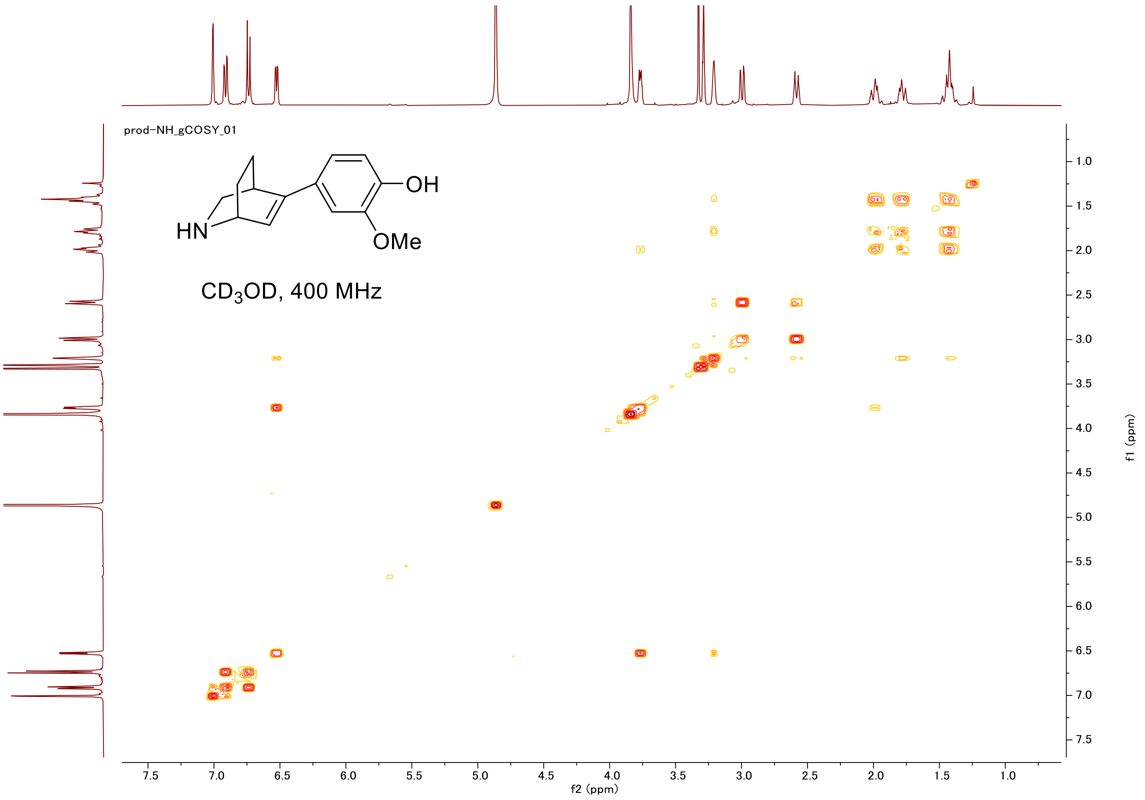
prod-NH_gCOSY_01
HN
OH
OMe
CD3OD, 400 MHz
f2 (ppm)
f1 (ppm)

HSQC

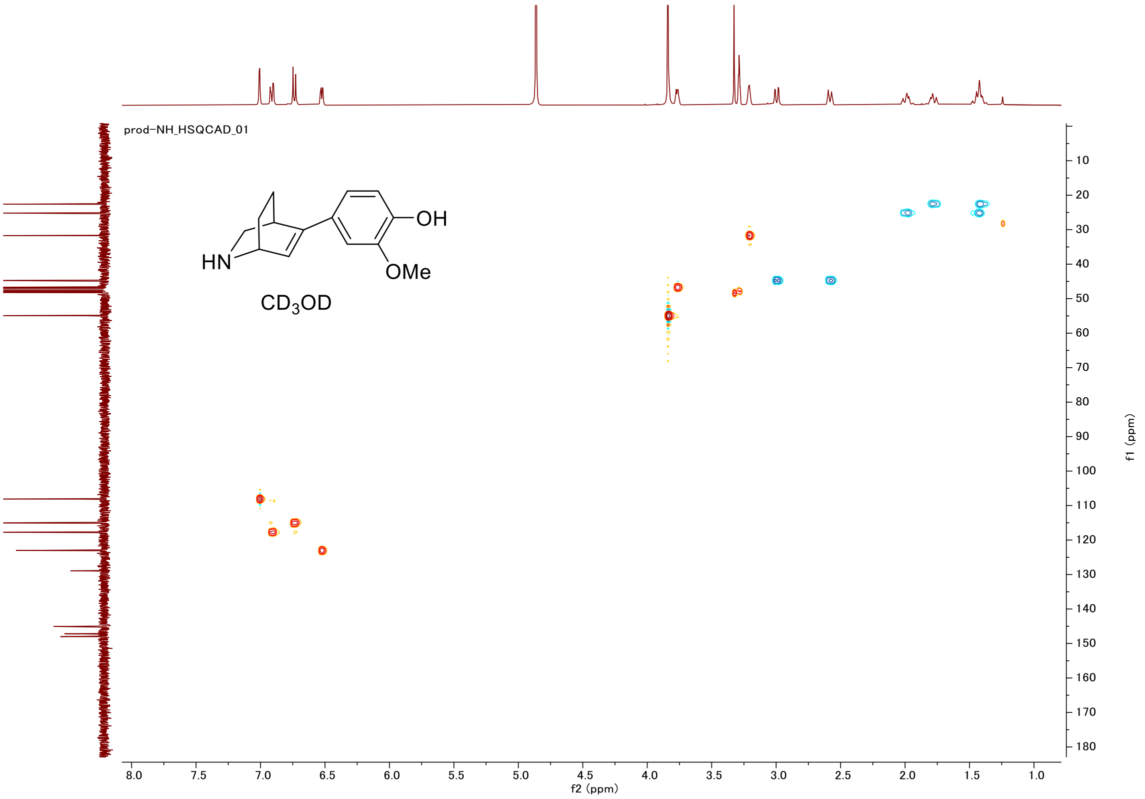
prod-NH_HSQCAD_01
HN
OH
OMe
CD3OD
f2 (ppm)
f1 (ppm)

Proton (#012)

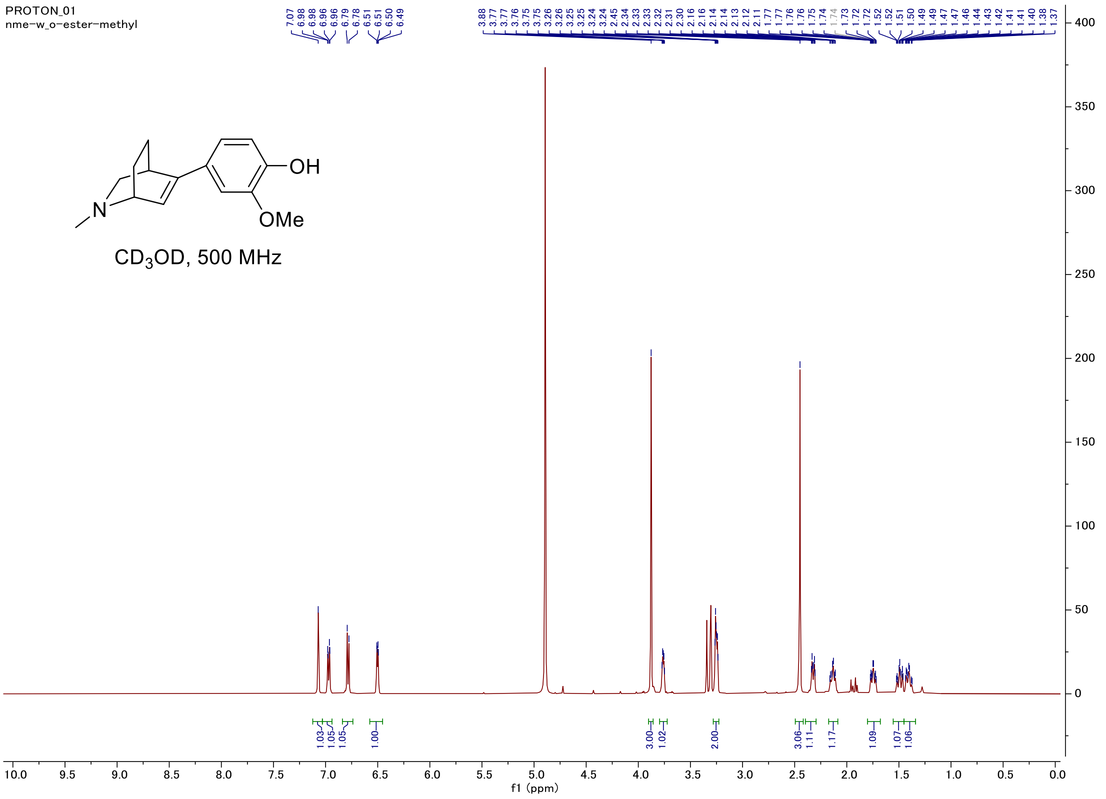

Carbon

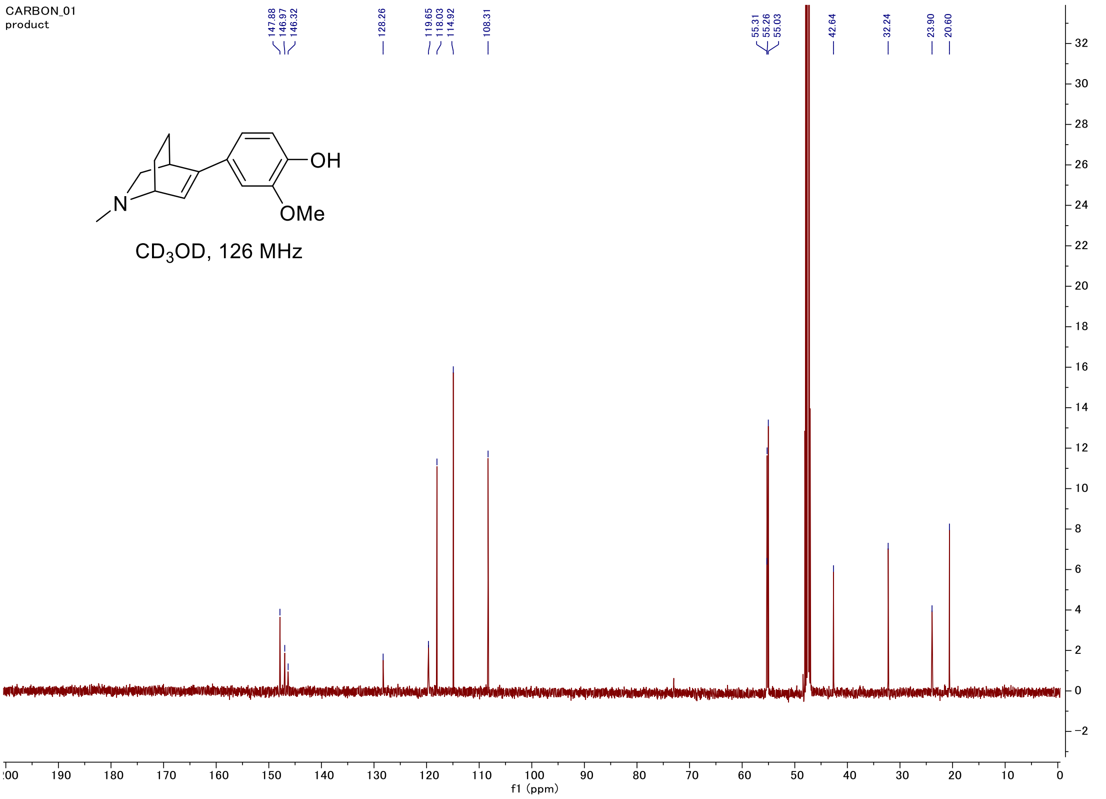

C-H COSY

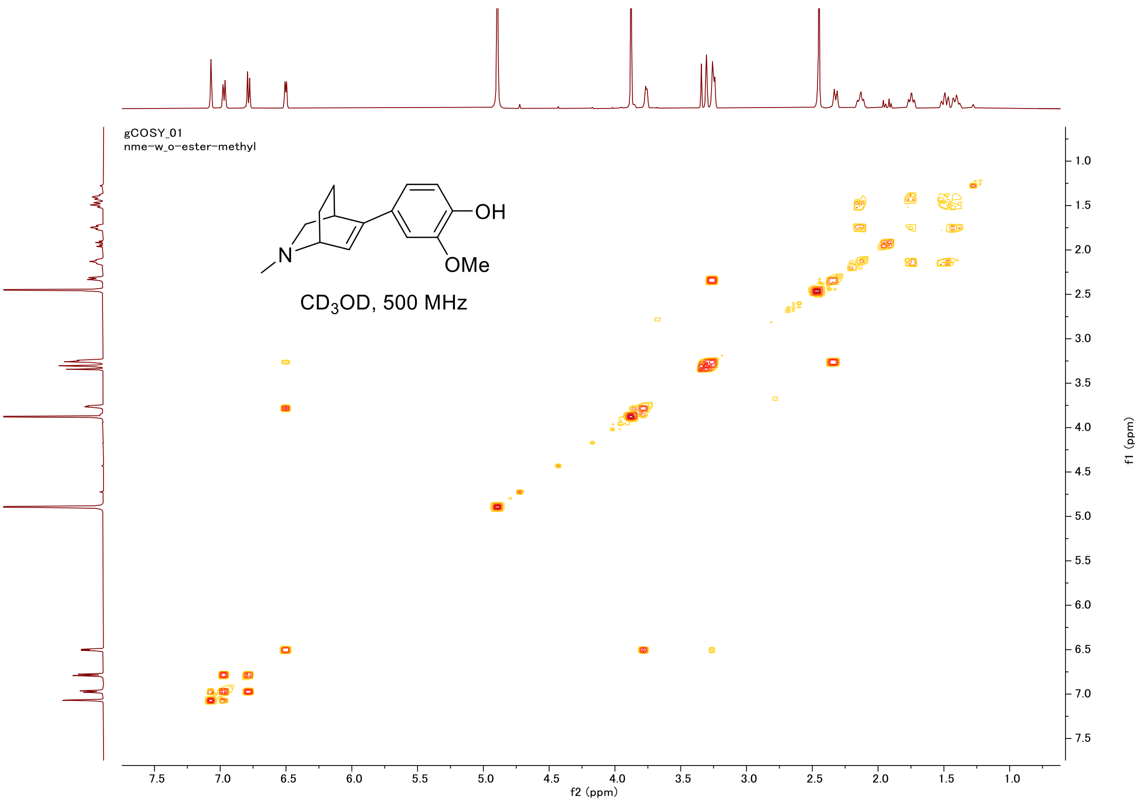
gCOSY_01
nme-w_o-ester-methyl
OH
N
OMe
CD3OD, 500 MHz
f2 (ppm)
f1 (ppm)

HSQC

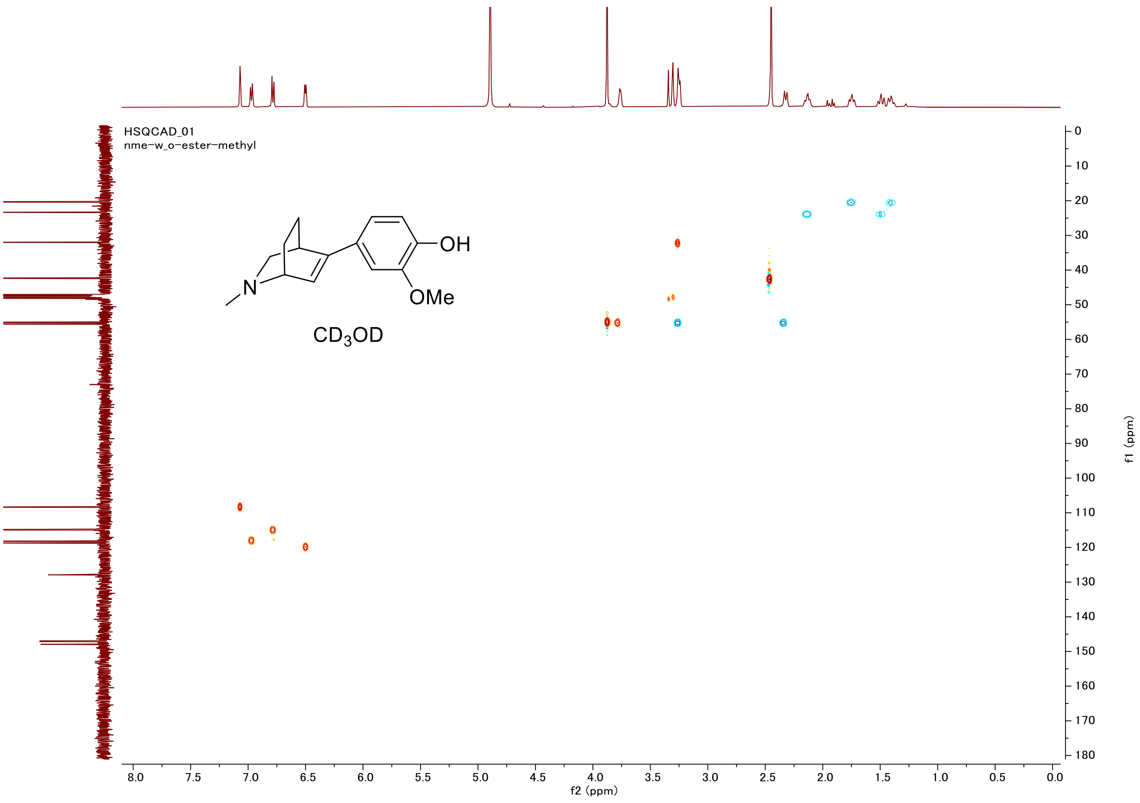
HSQCAD_01
nme-w_o-ester-methyl
OH
N
OMe
CD3OD
f2 (ppm)
f1 (ppm)

Proton (#013)

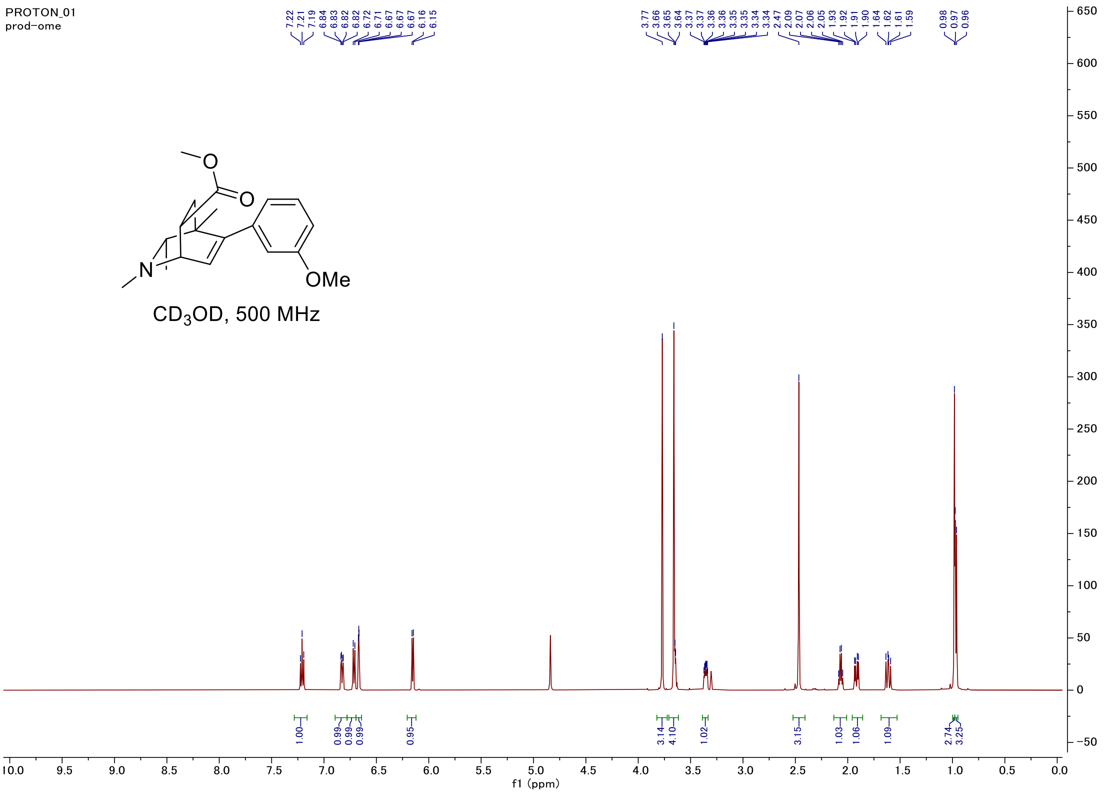

Carbon

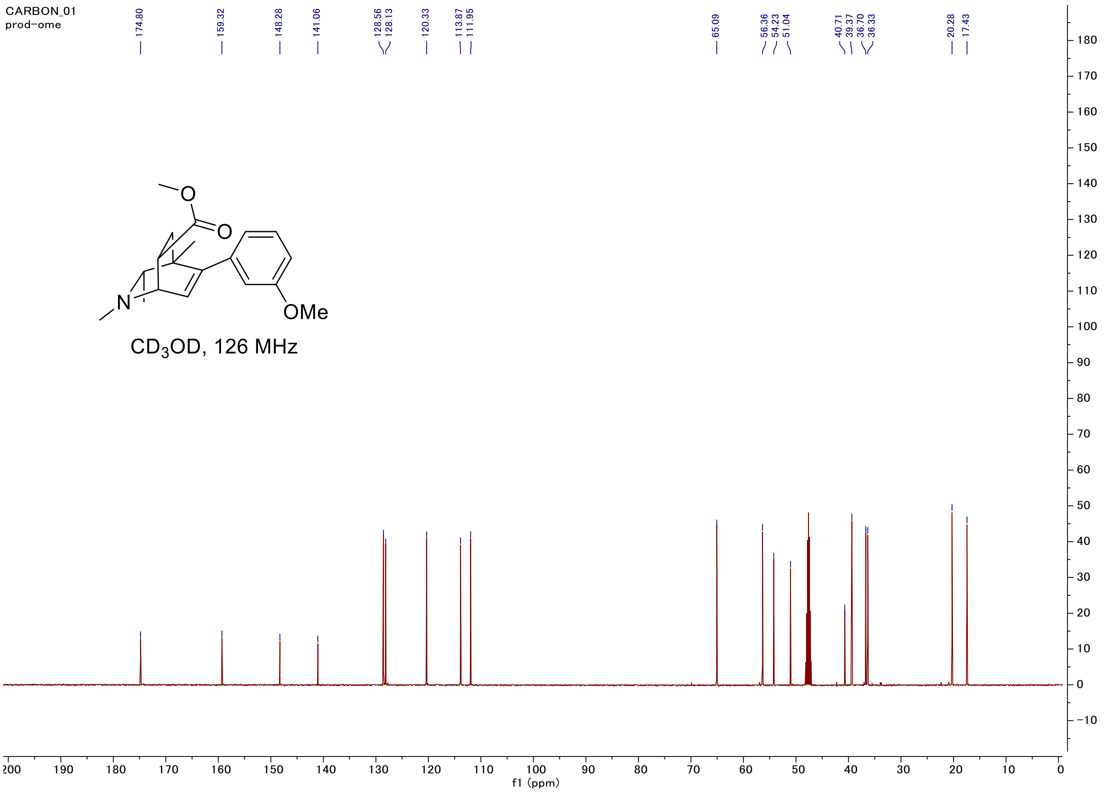

C-H COSY

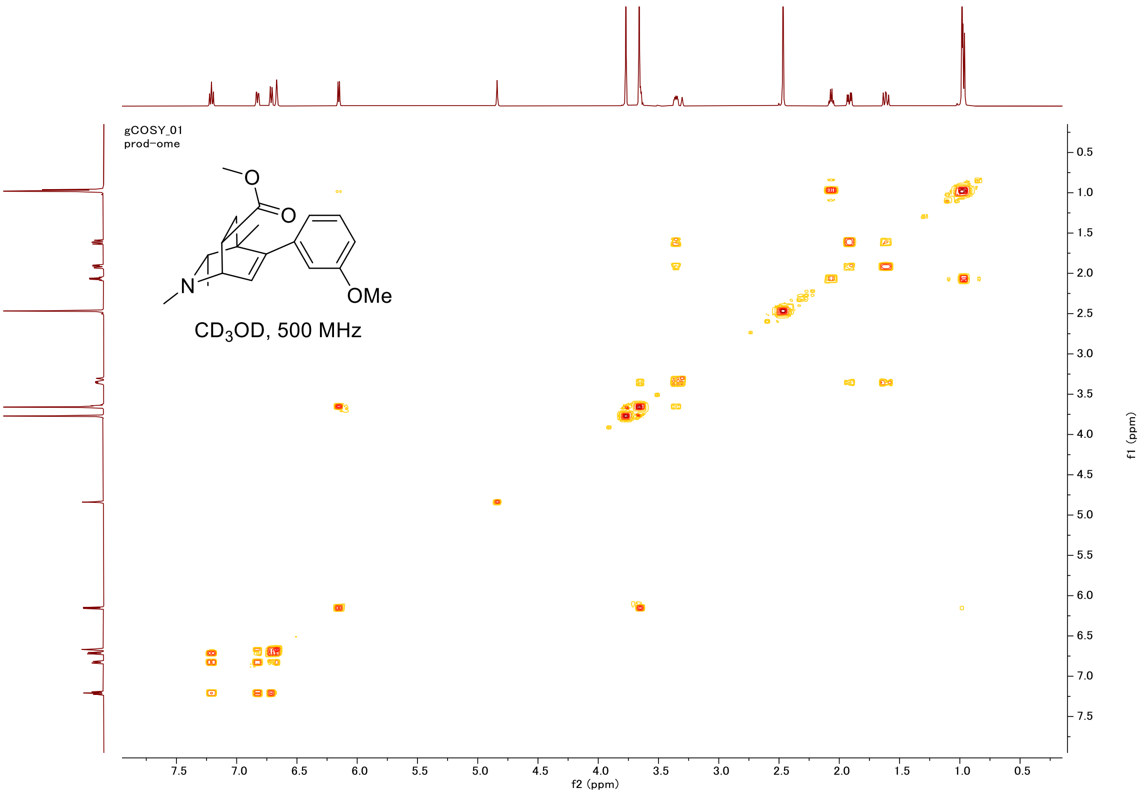
gCOSY_01
prod-ome
O
O
N
OMe
CD$_3$OD, 500 MHz
f2 (ppm)
f1 (ppm)

HSQC

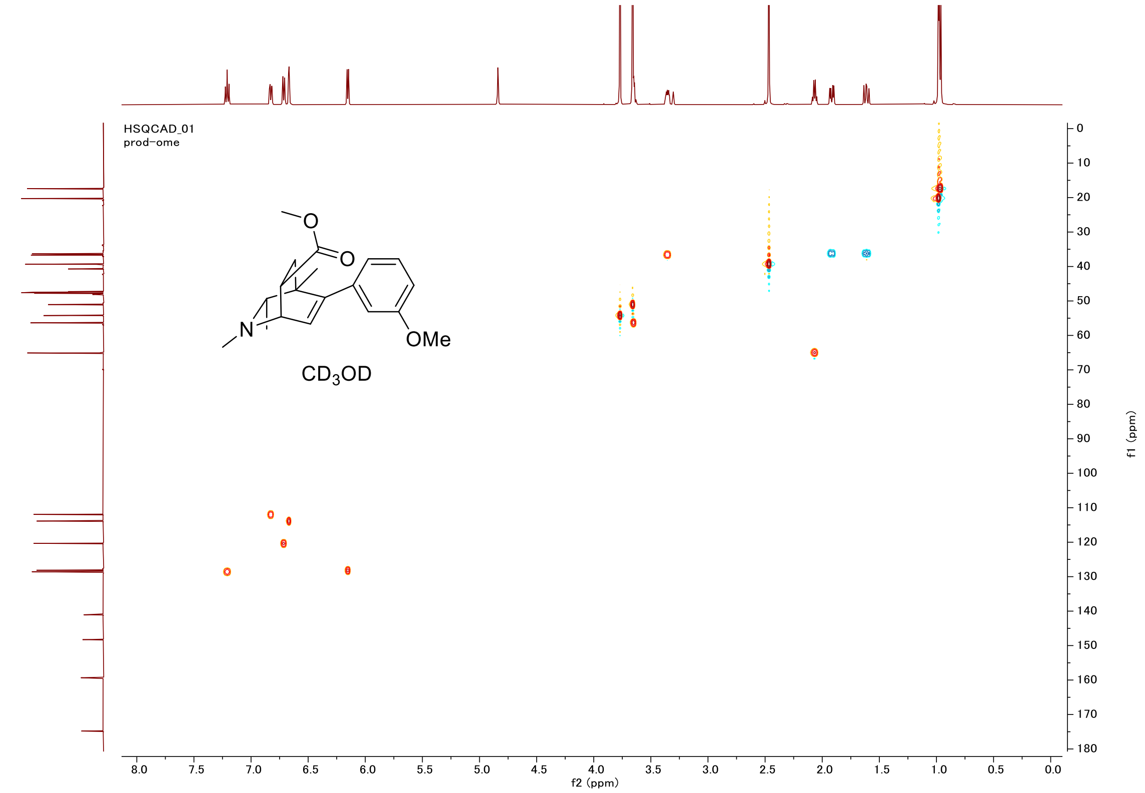
HSQCAD_01
prod-ome
O
O
N
OMe
CD$_3$OD
f2 (ppm)
f1 (ppm)

Proton (#014)

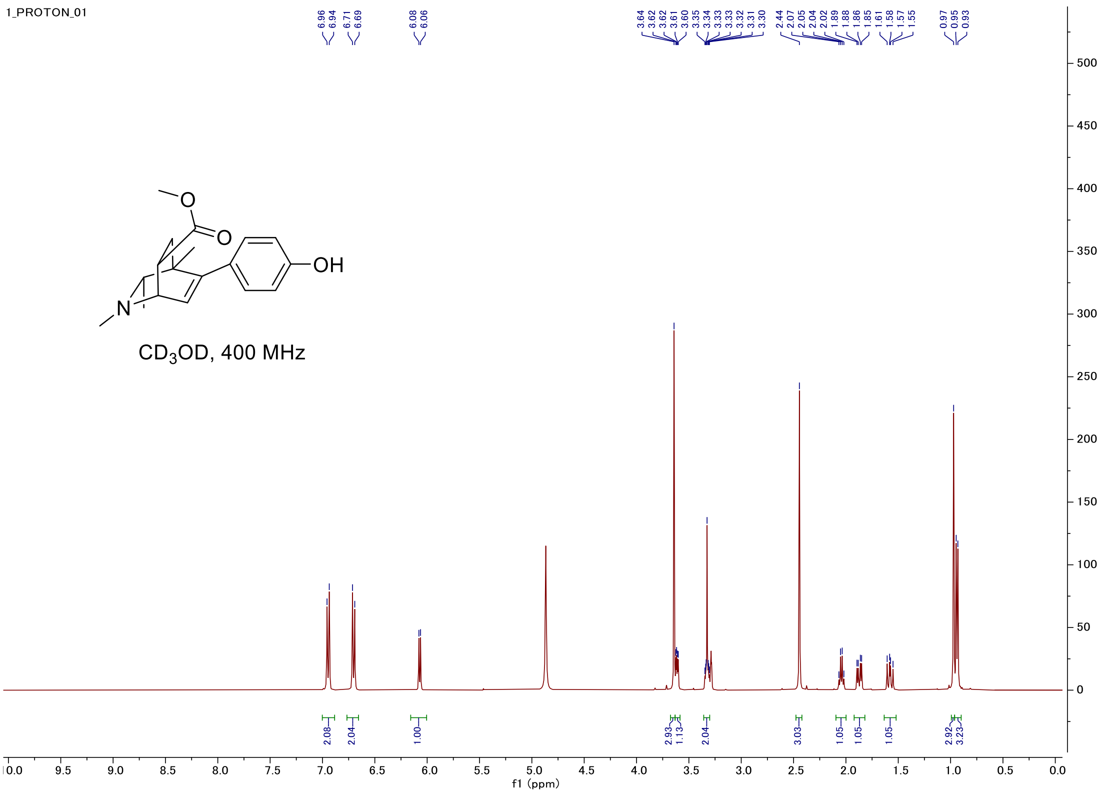

Carbon

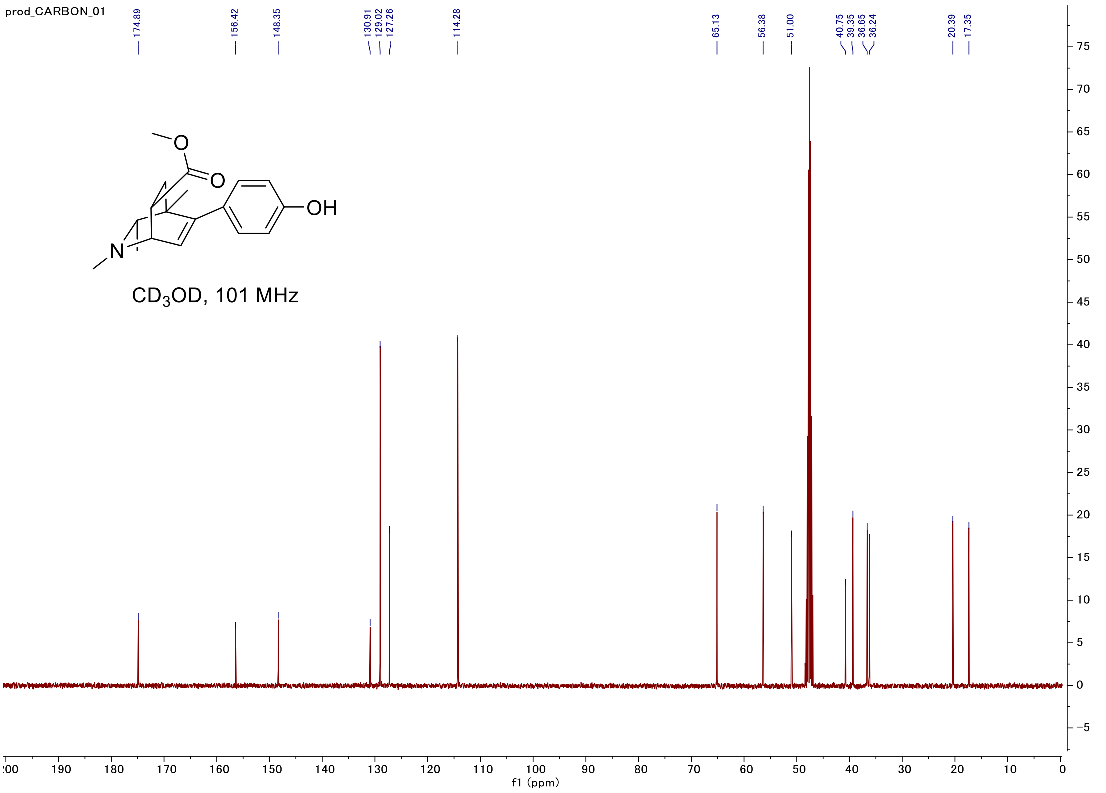

C-H COSY

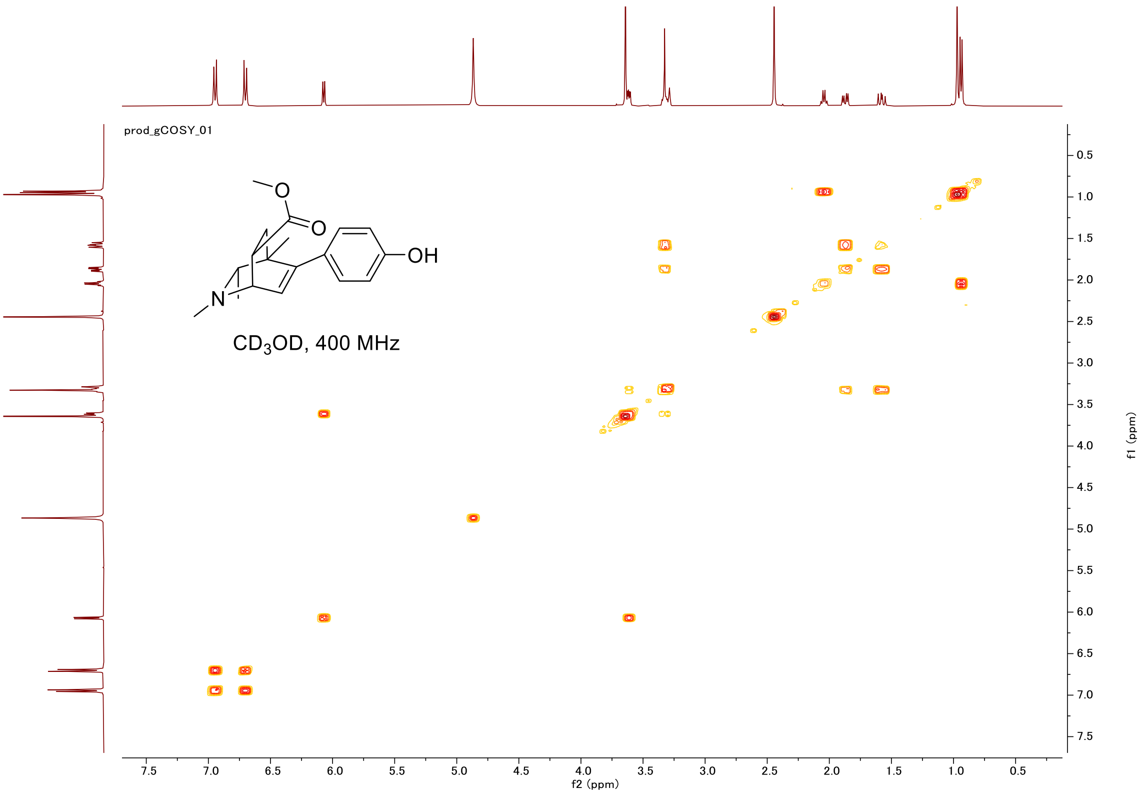
prod_gCOSY_01
OH
N
O
O
CD3OD, 400 MHz
f2 (ppm)
f1 (ppm)

HSQC

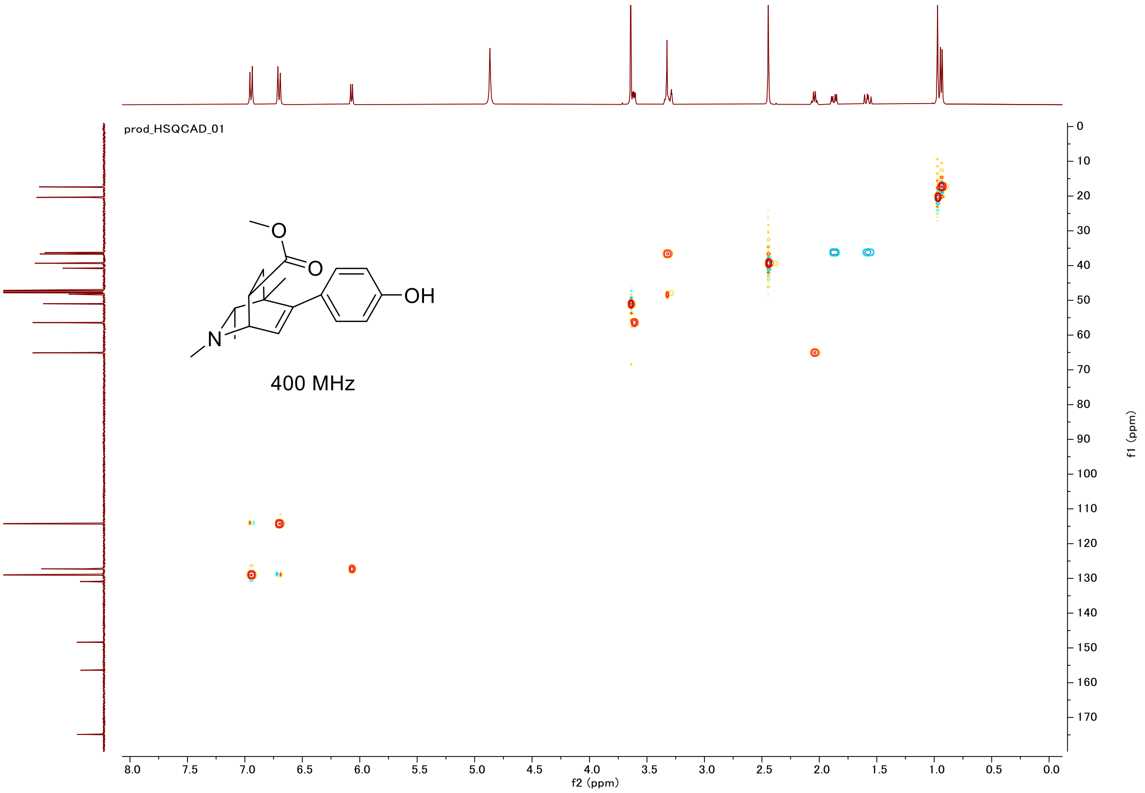
prod_HSQCAD_01
OH
N
O
O
400 MHz
f2 (ppm)
f1 (ppm)

Proton (#015)

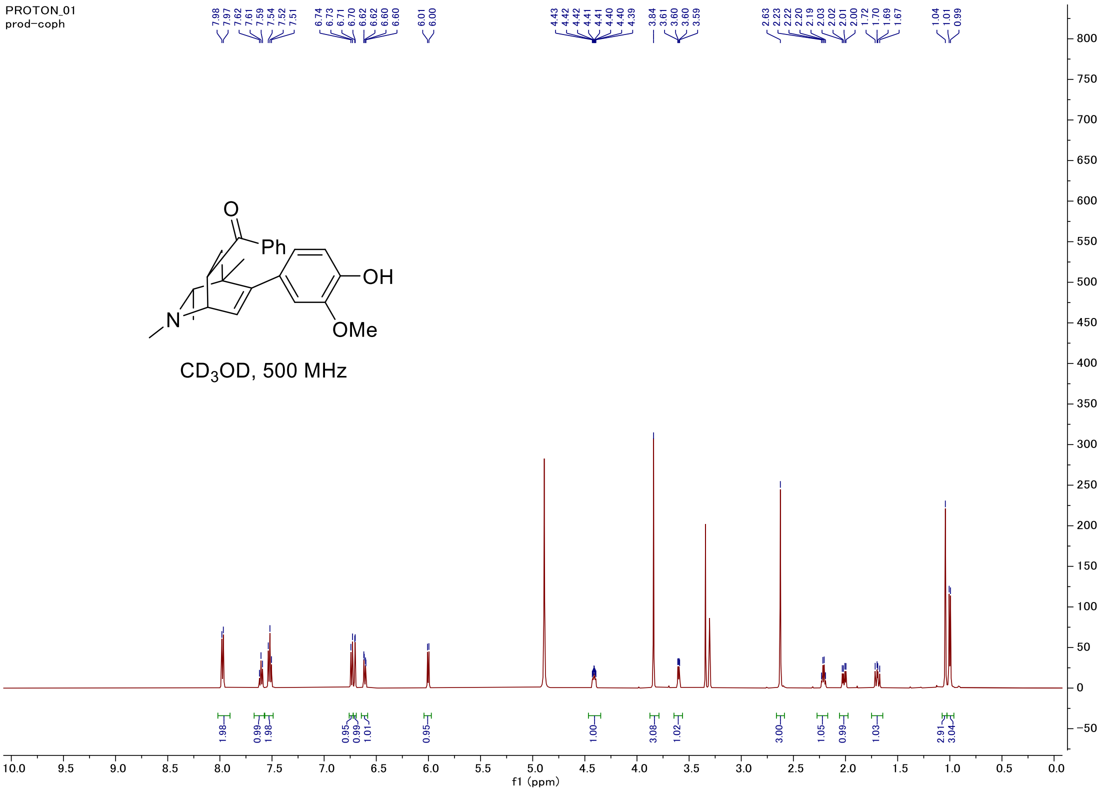

Carbon

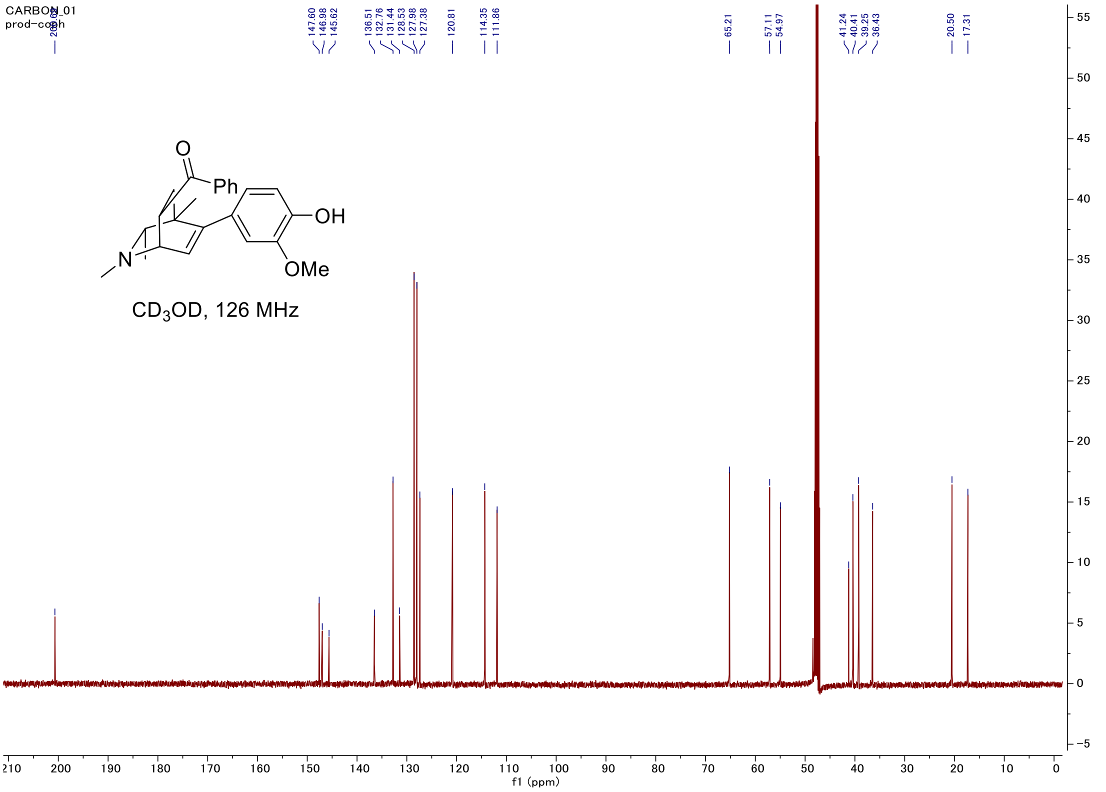

C-H COSY

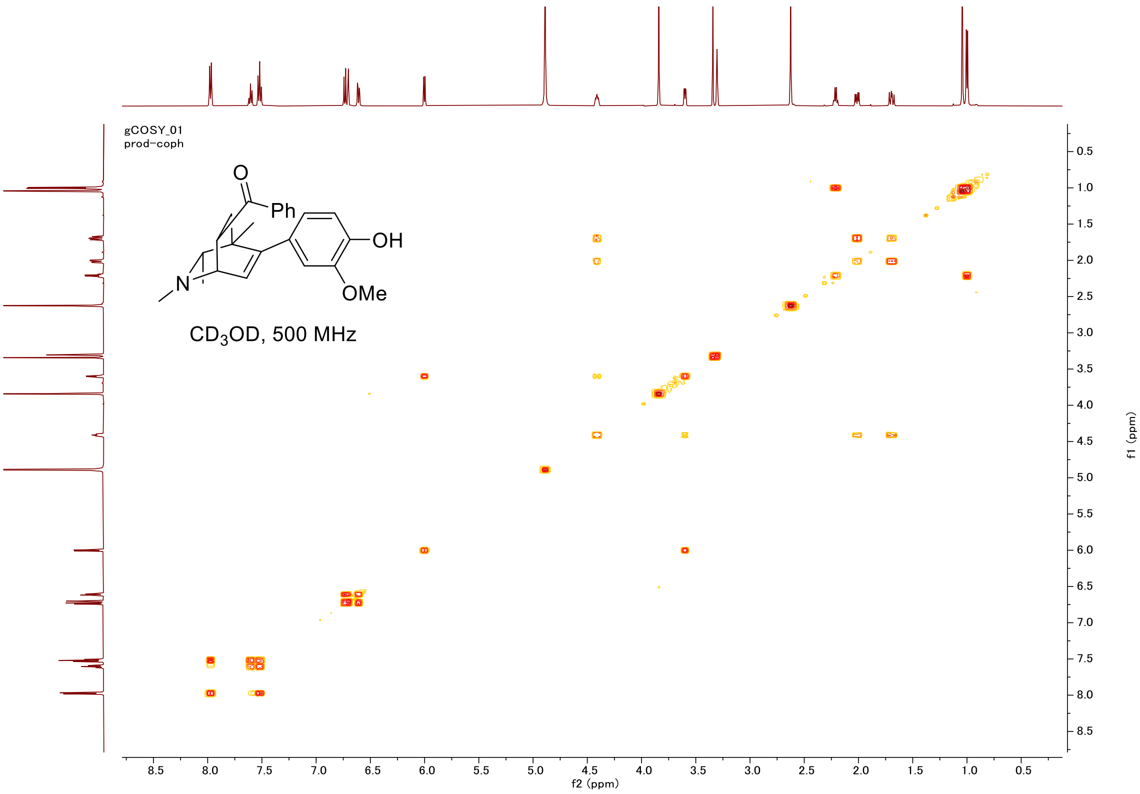
gCOSY_01
prod-coph
O
Ph
OH
N
OMe
CD3OD, 500 MHz
f2 (ppm)
f1 (ppm)

HSQC

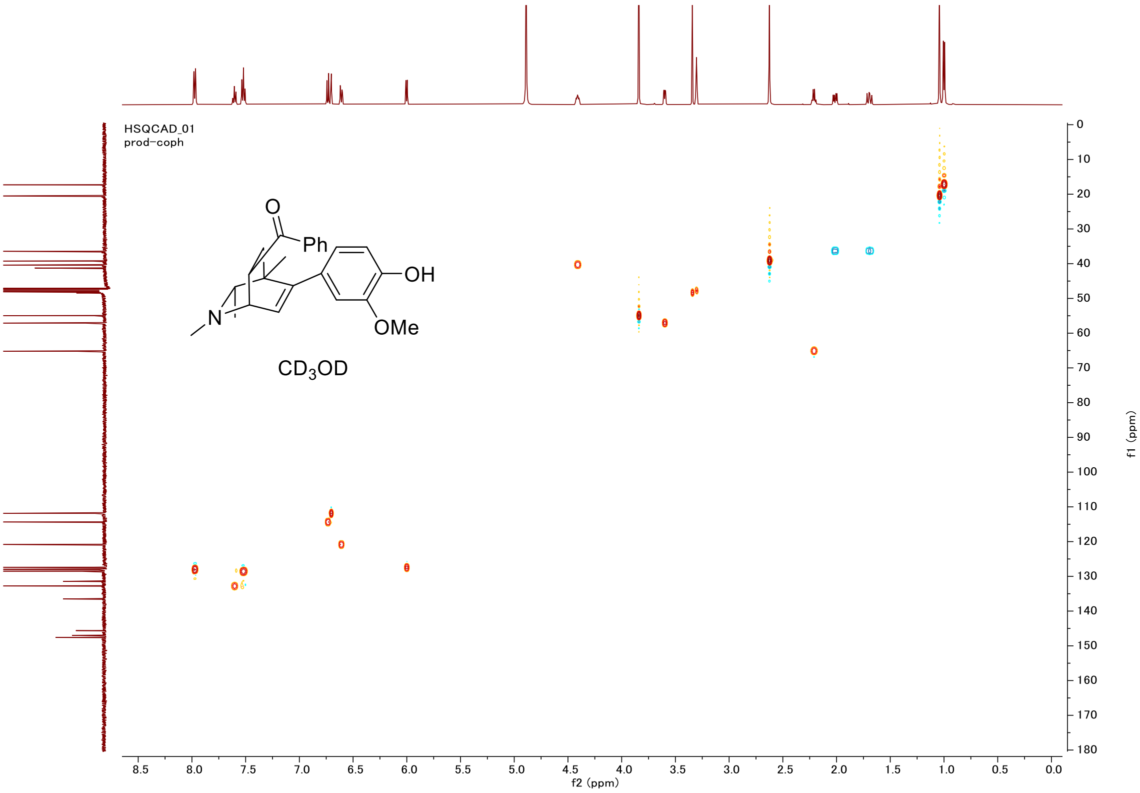
HSQCAD_01
prod-coph
O
Ph
OH
N
OMe
CD3OD
f2 (ppm)
f1 (ppm)

Proton (#016)

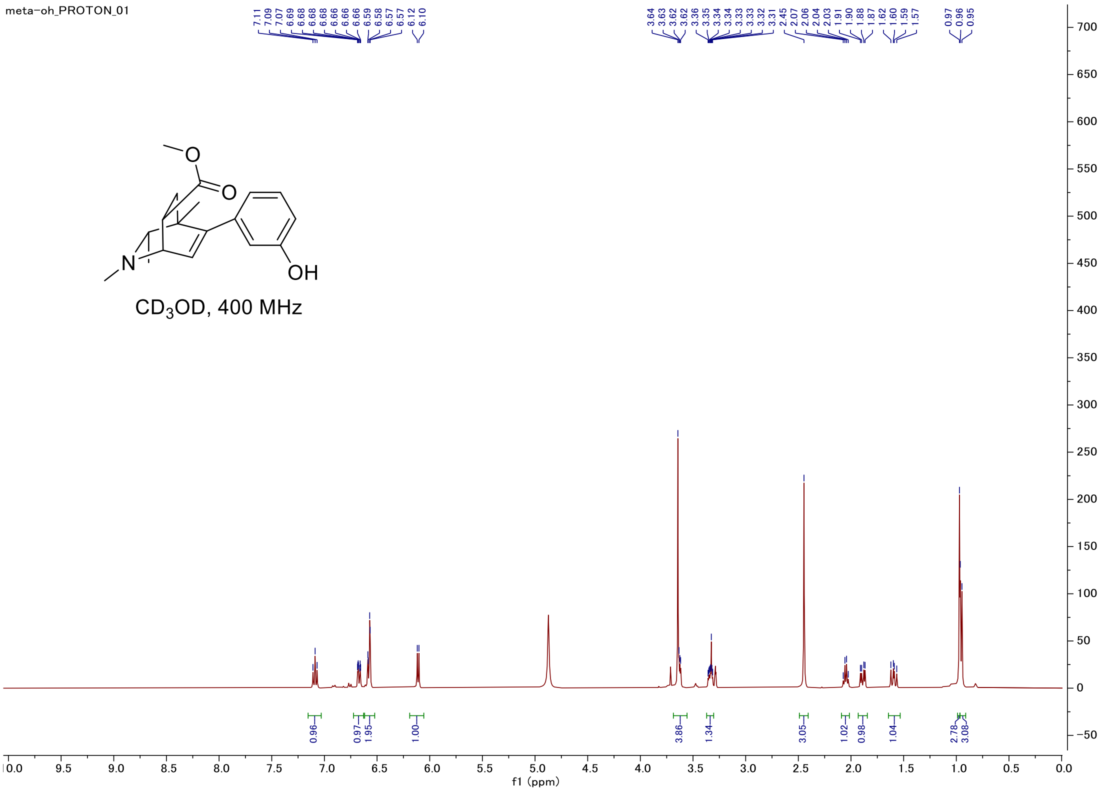

Carbon

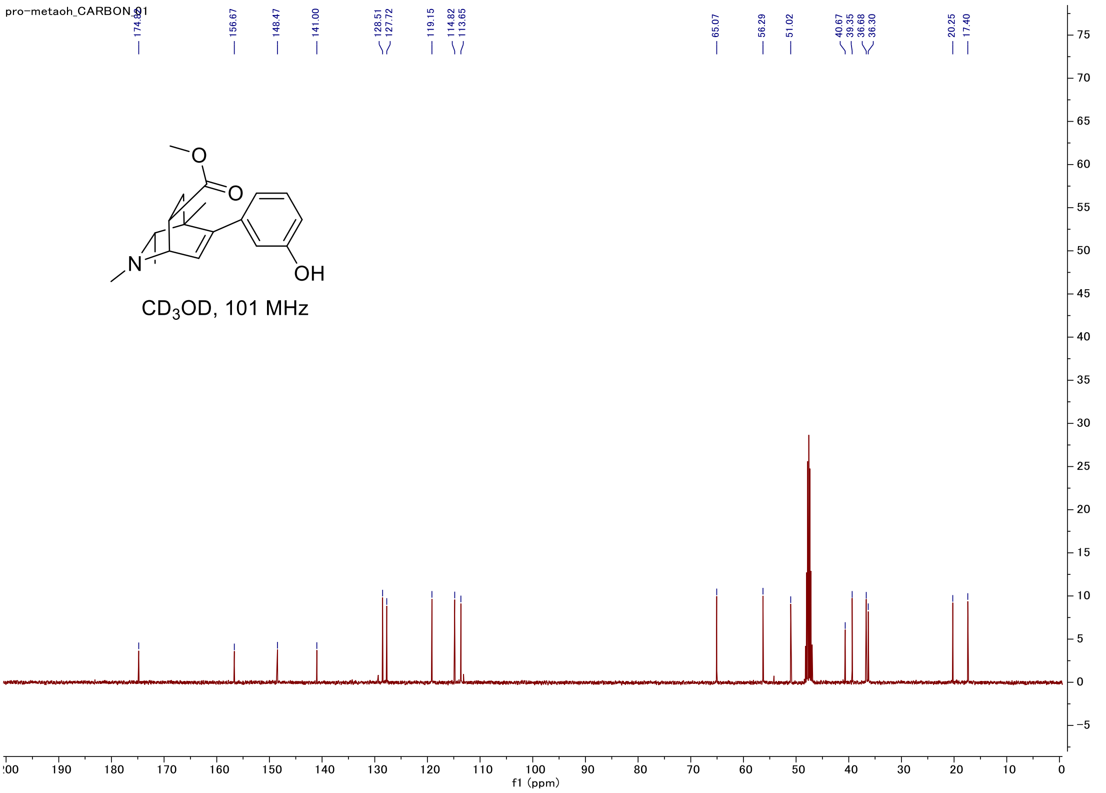

C-H COSY

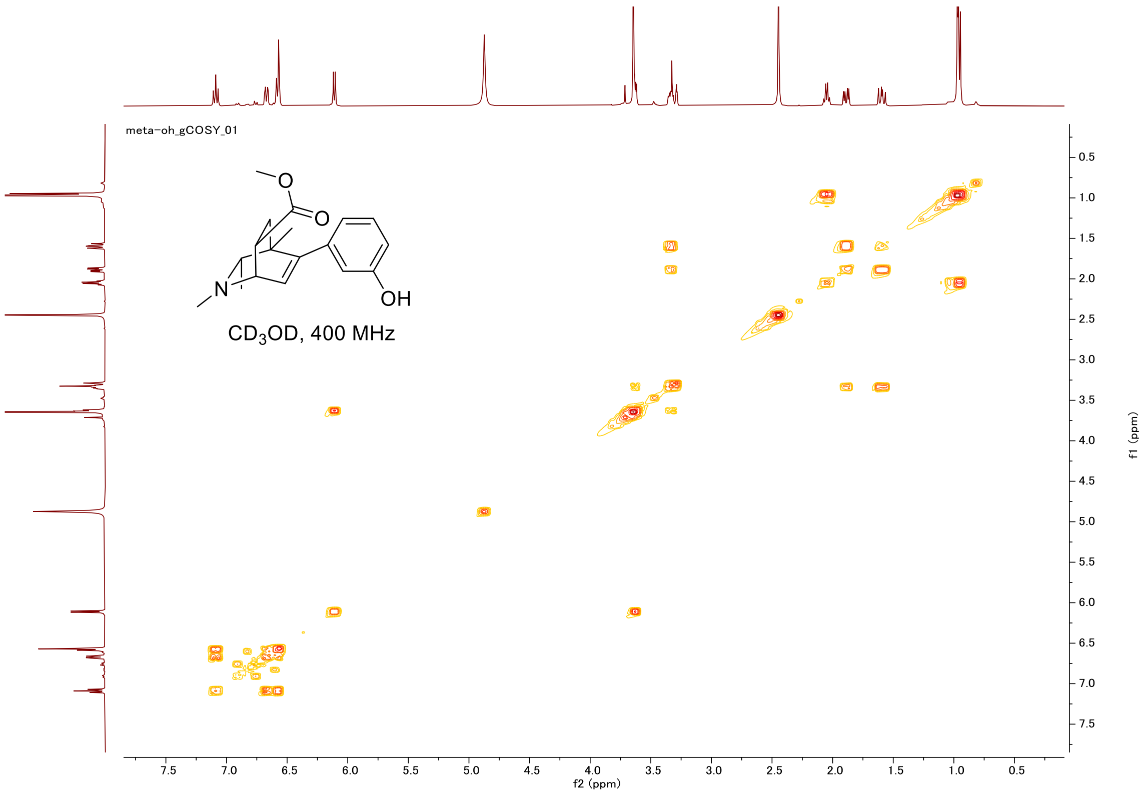
meta-oh_gCOSY_01
OH
N
O
O
CD3OD, 400 MHz
f2 (ppm)
f1 (ppm)

HSQC

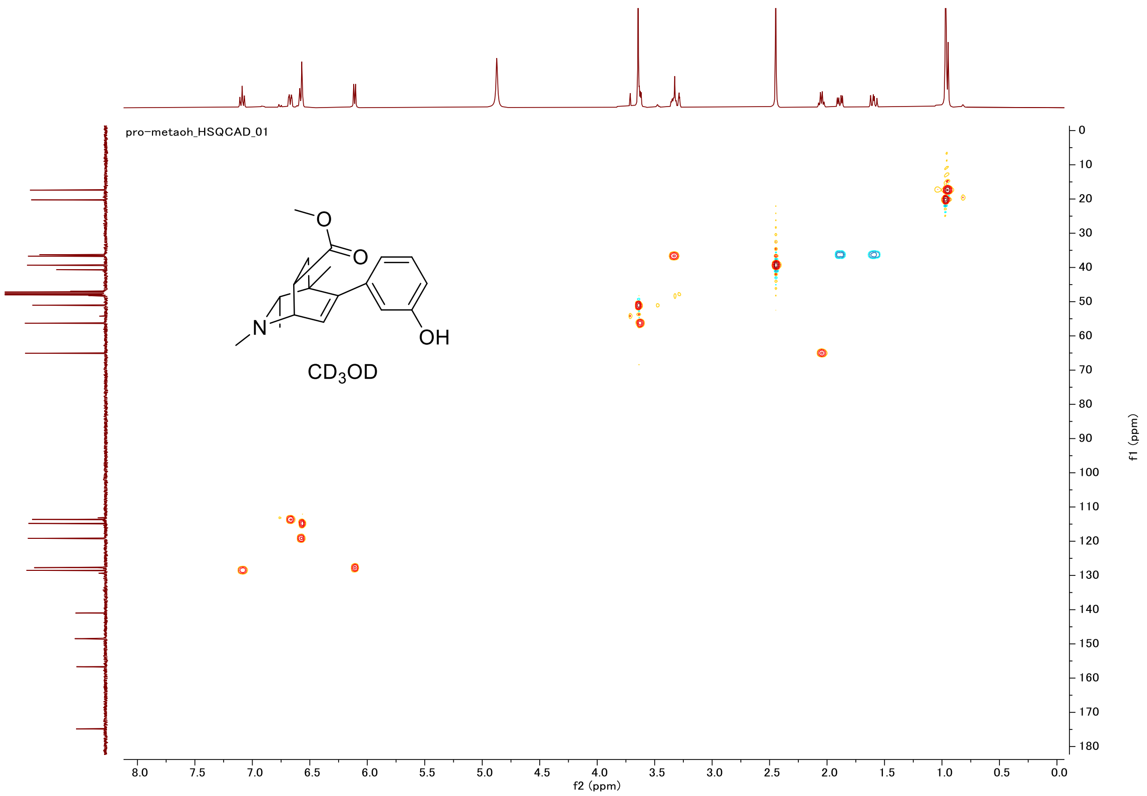
pro-metaoh_HSQCAD_01
OH
N
O
O
CD3OD
f2 (ppm)
f1 (ppm)

Proton (#017)

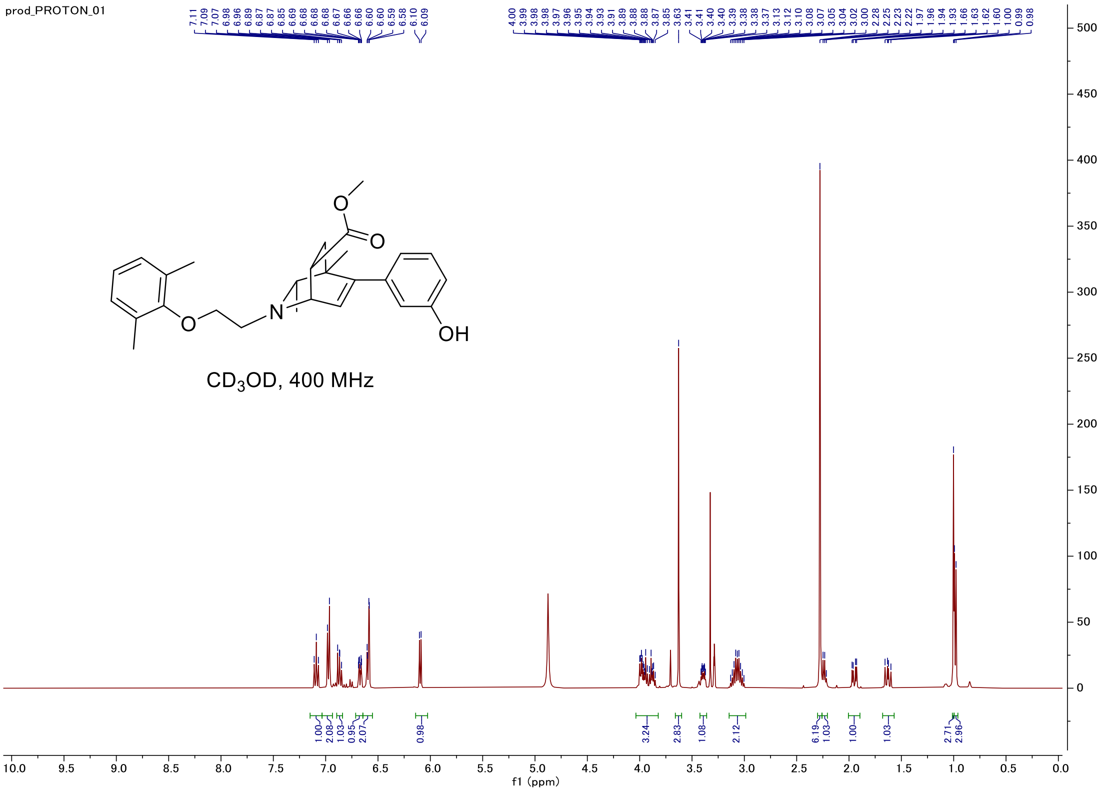

Carbon

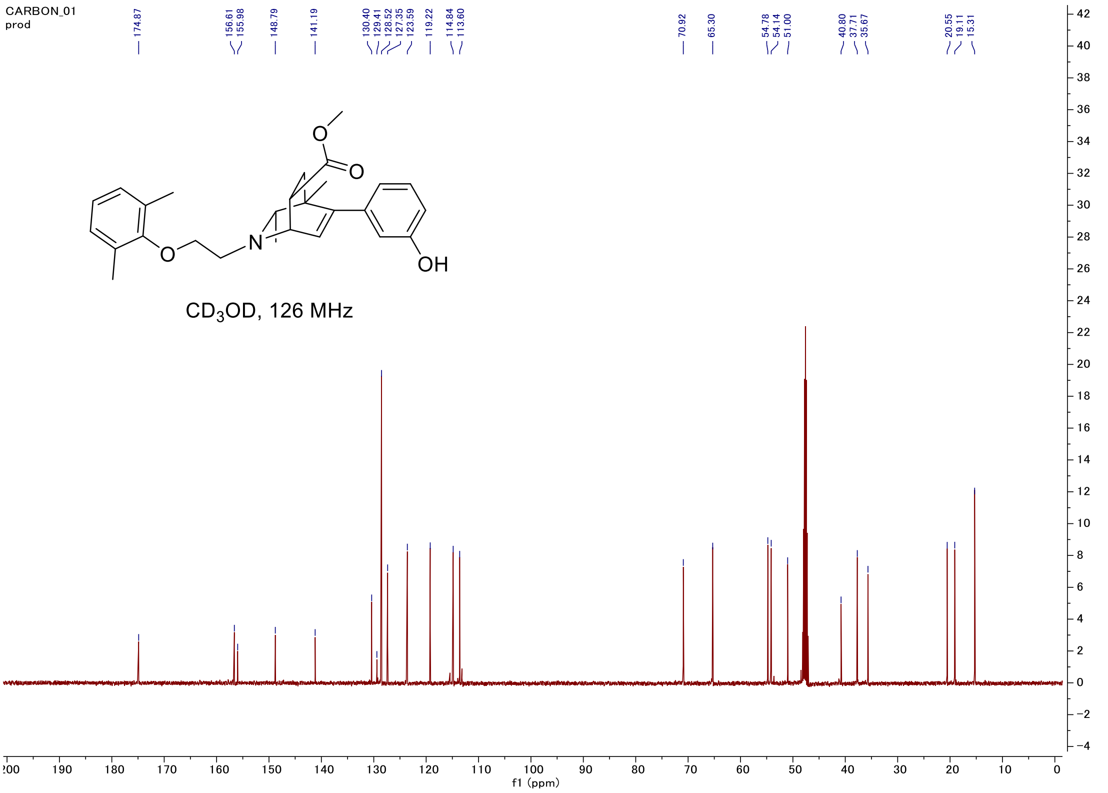

C-H COSY

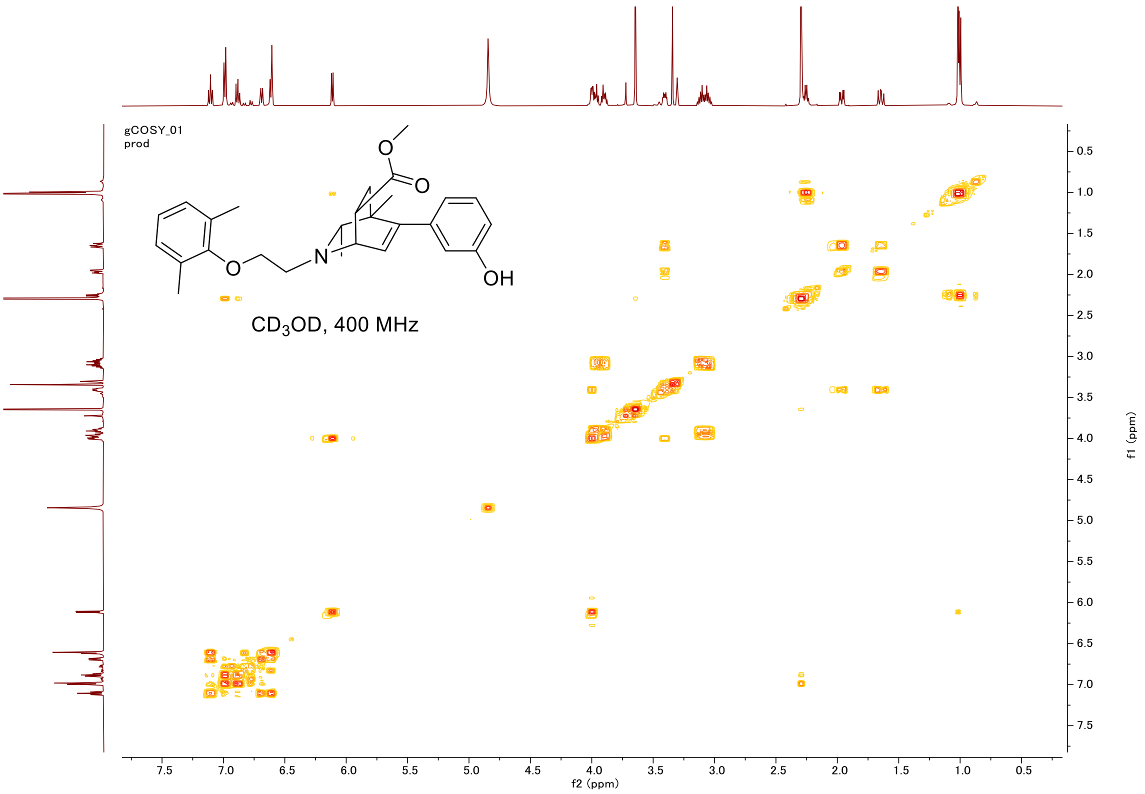
gCOSY_01
prod
OH
CD3OD, 400 MHz
f2 (ppm)
f1 (ppm)

HSQC

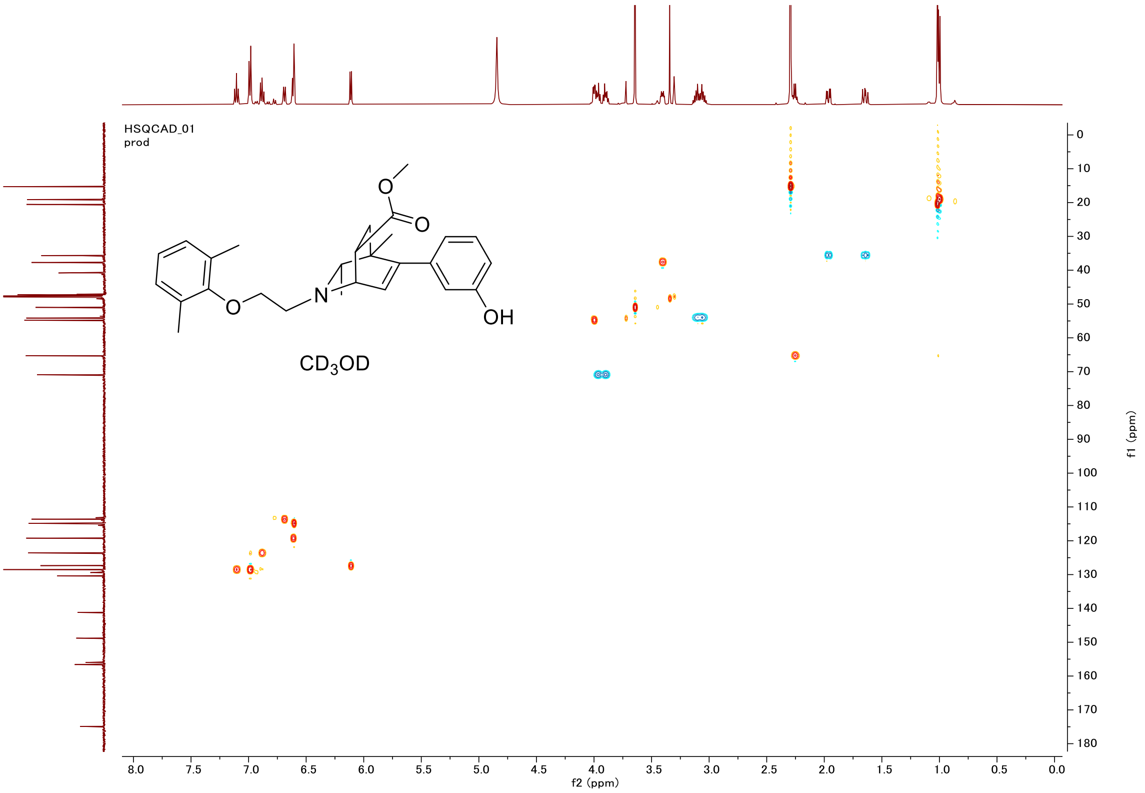
HSQCAD_01
prod
OH
CD3OD
f2 (ppm)
f1 (ppm)

Proton (#018)

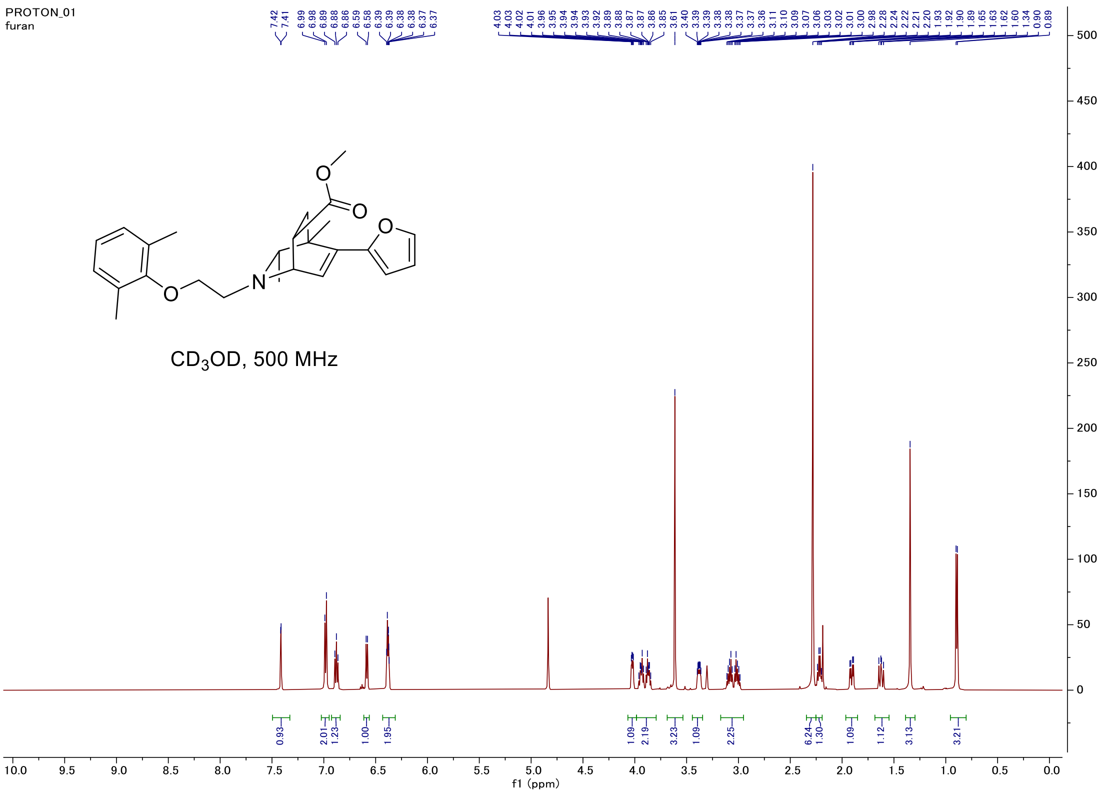

Carbon

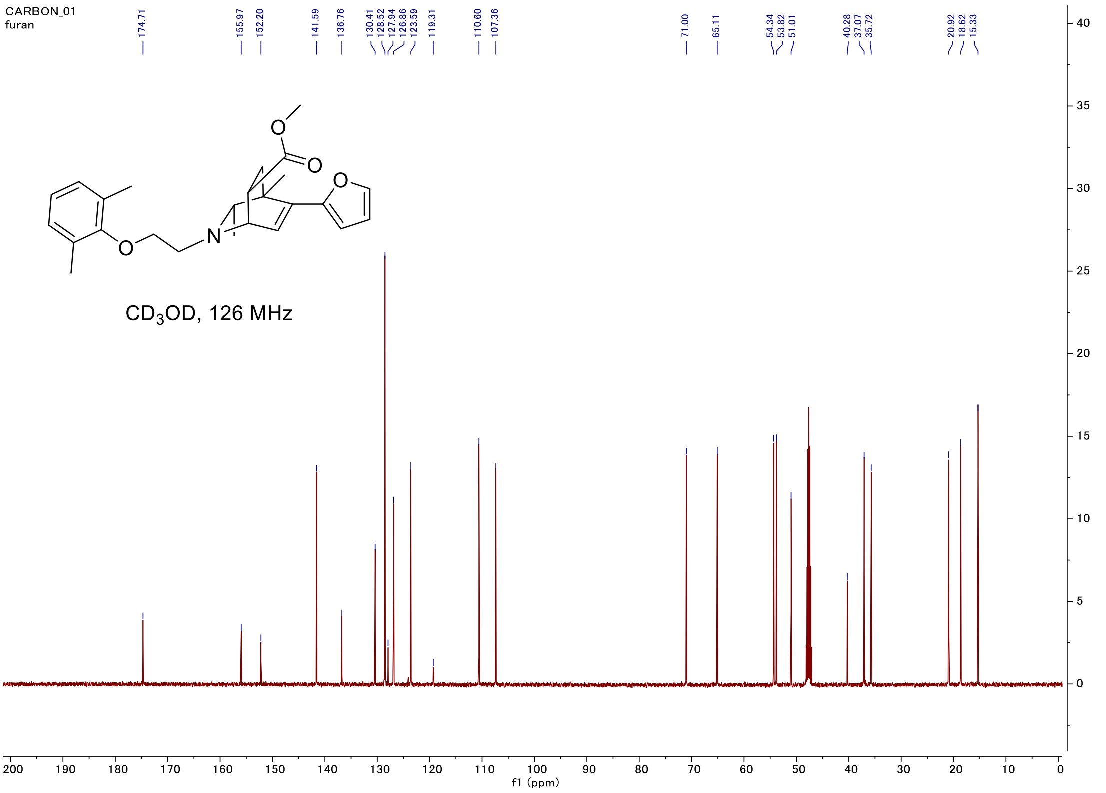

C-H COSY

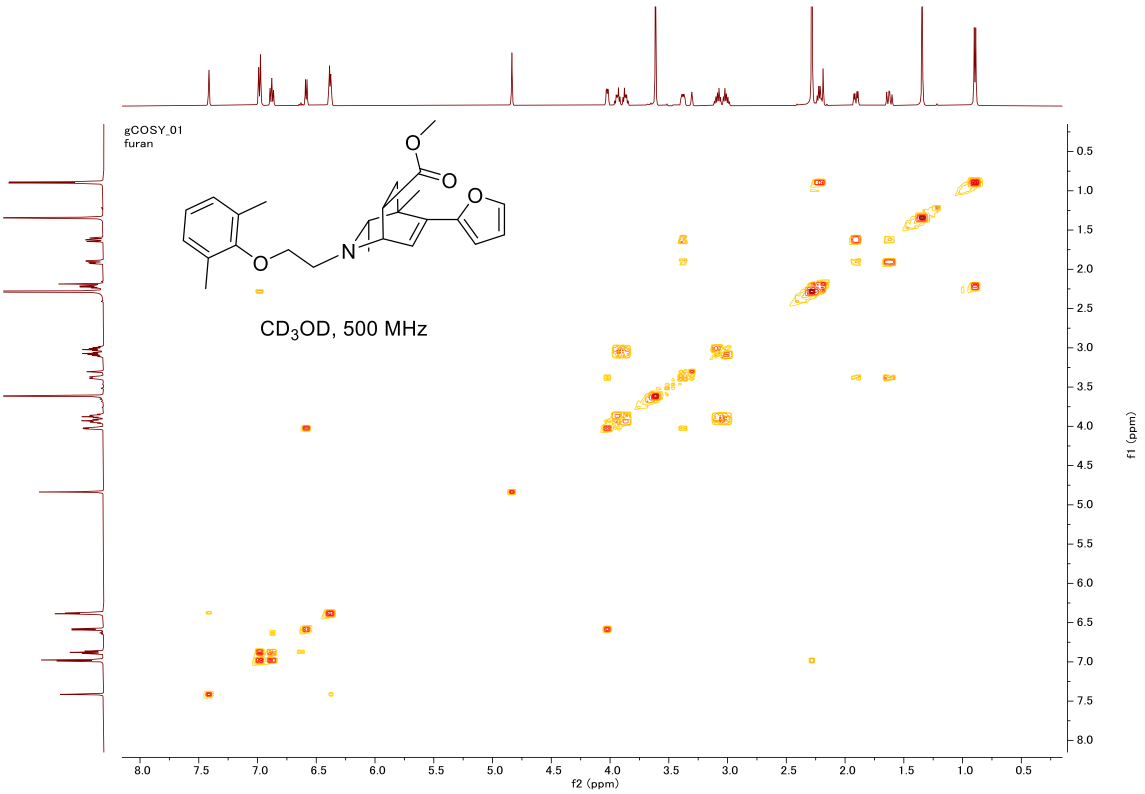
gCOSY_01
furan
CD$_3$OD, 500 MHz
f2 (ppm)
f1 (ppm)

HSQC

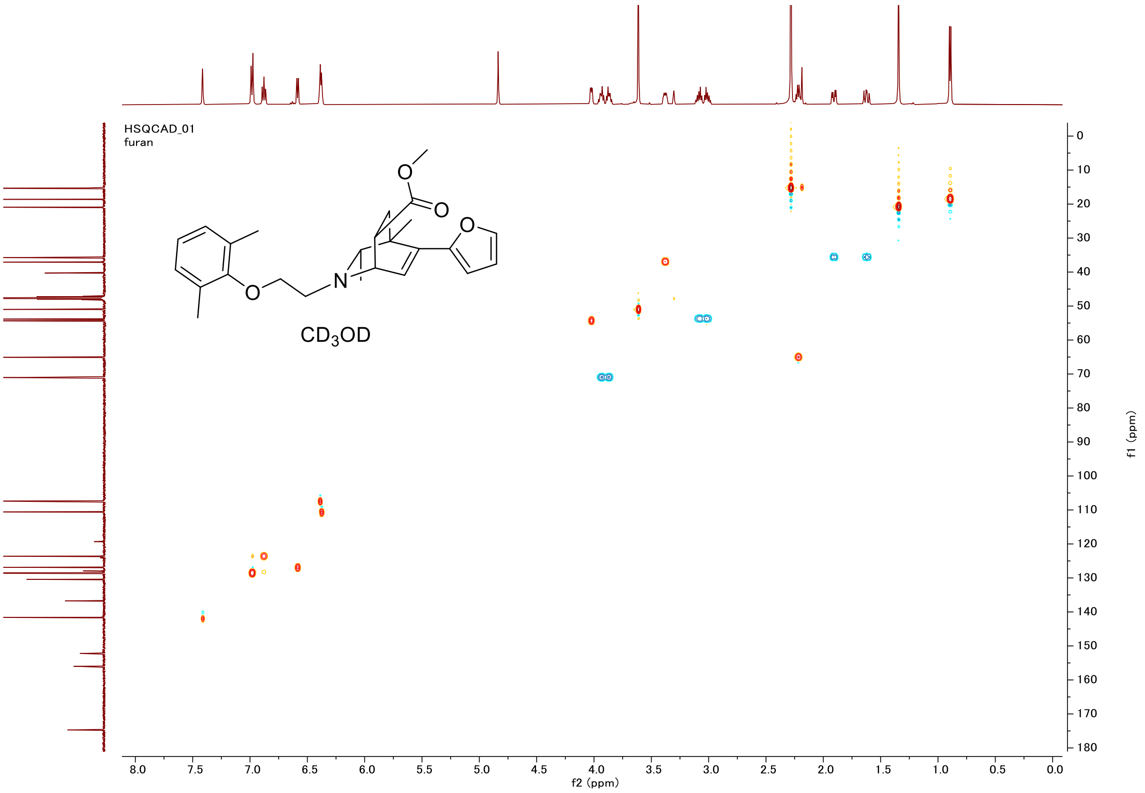
HSQCAD_01
furan
CD$_3$OD
f2 (ppm)
f1 (ppm)

Proton (#019)

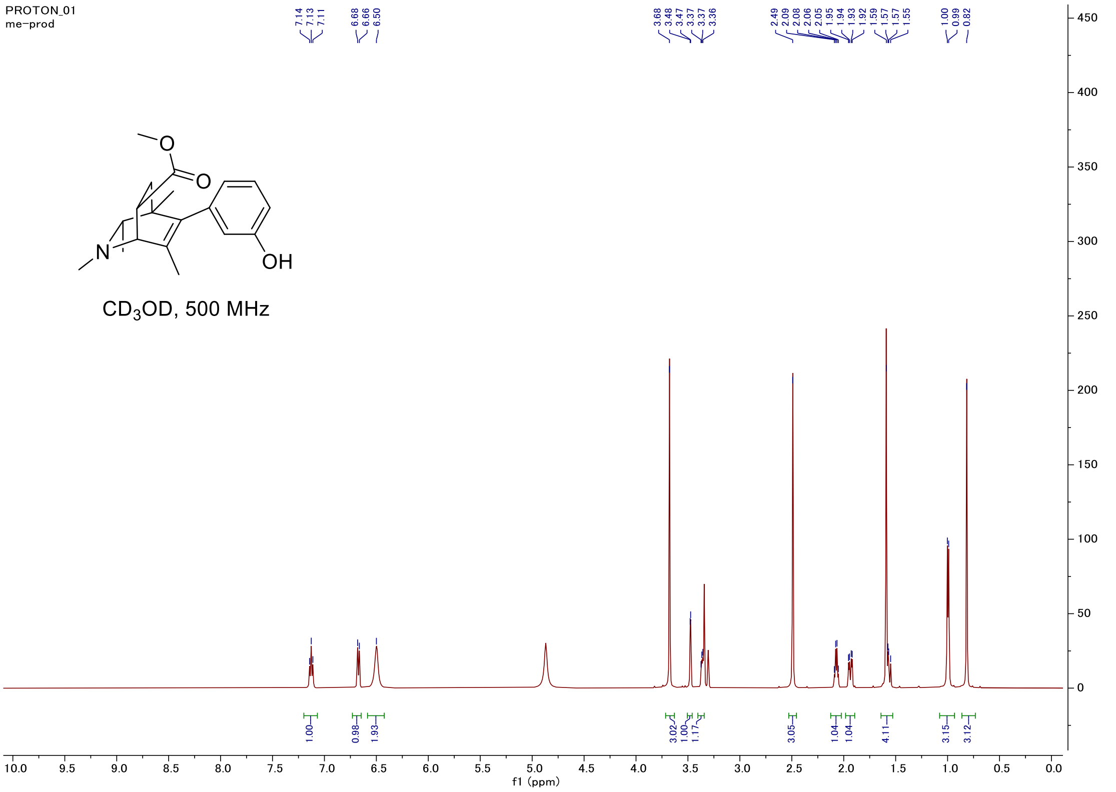

Carbon

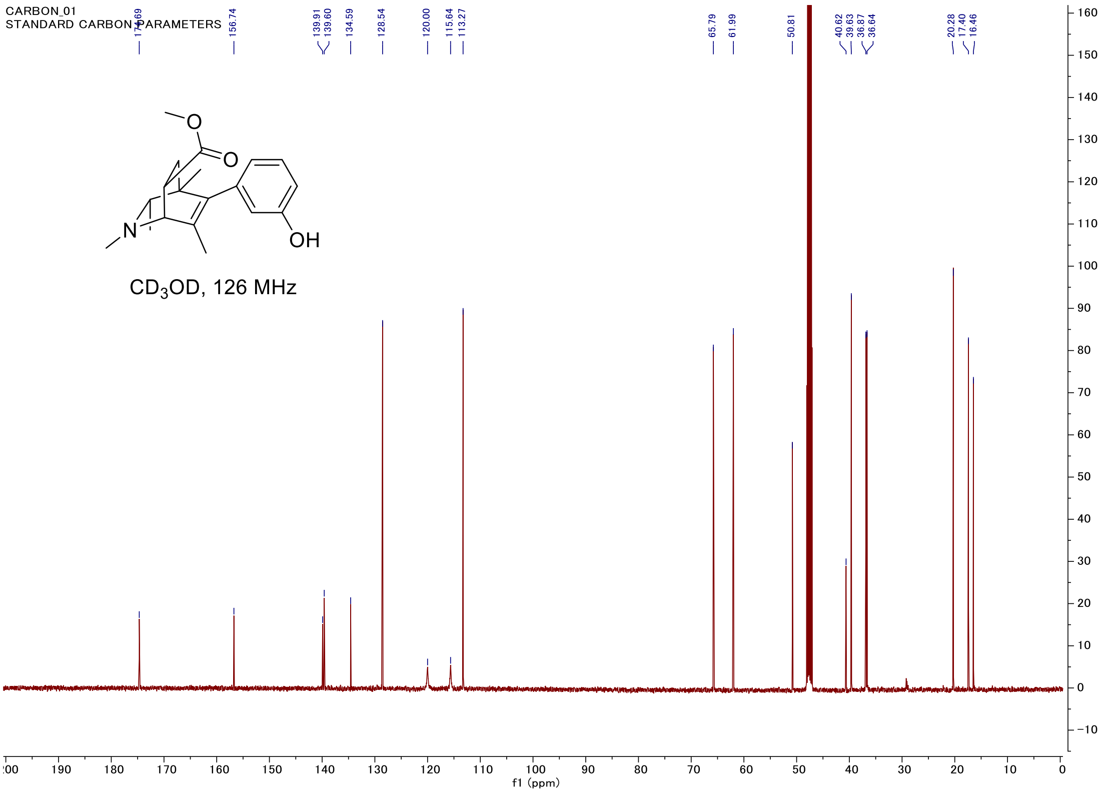

C-H COSY

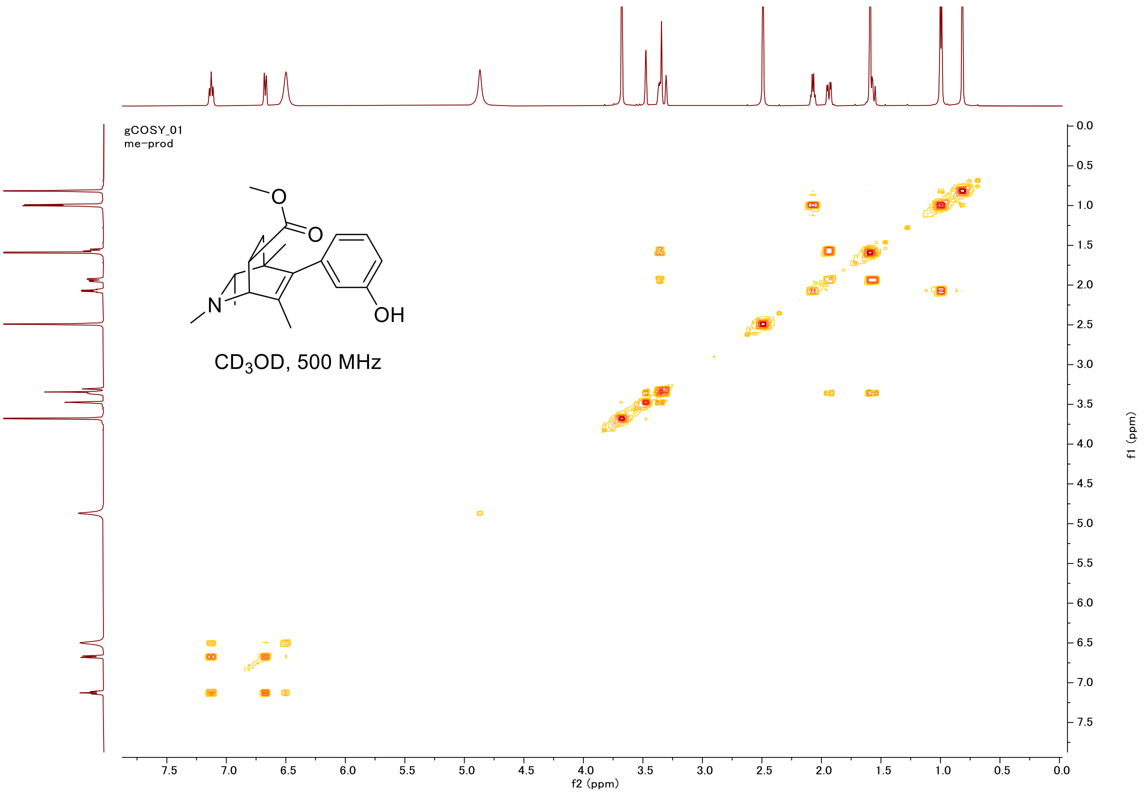
gCOSY_01
me-prod
O
O
N
OH
CD3OD, 500 MHz
f2 (ppm)
f1 (ppm)

HSQC

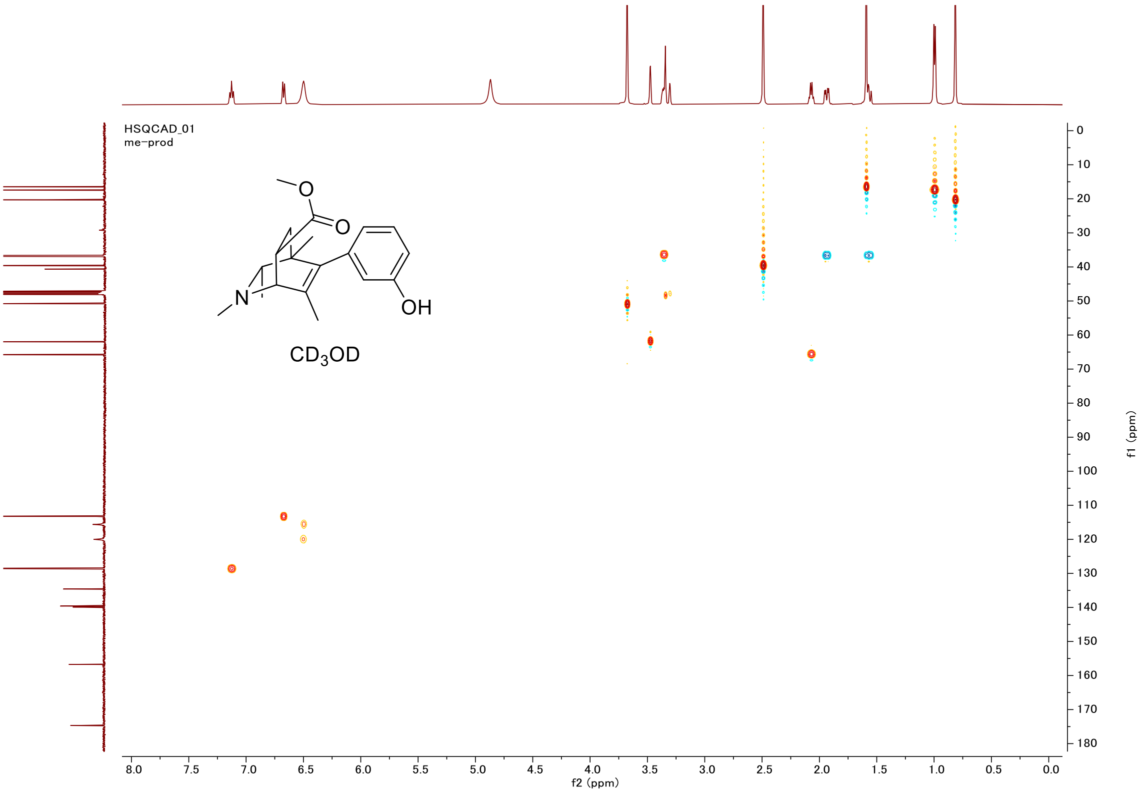
HSQCAD_01
me-prod
O
O
N
OH
CD3OD
f2 (ppm)
f1 (ppm)

Proton (#020-OPMB)

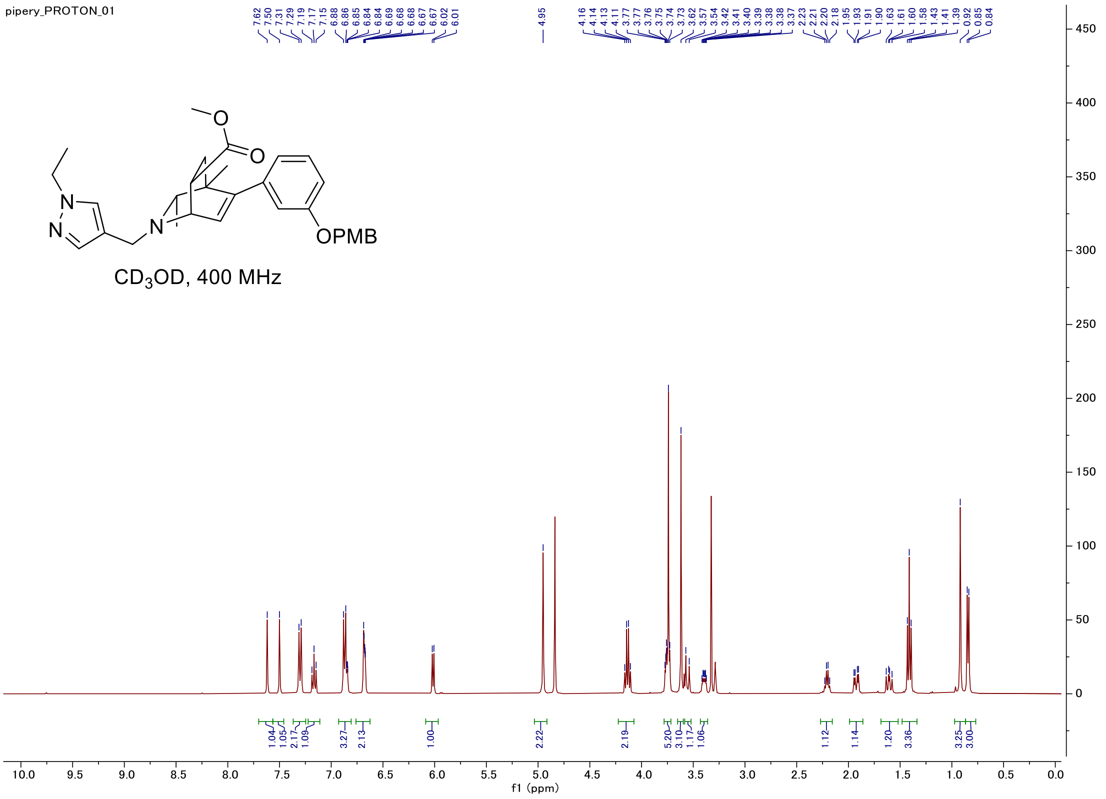

Carbon (#020-OPMB)

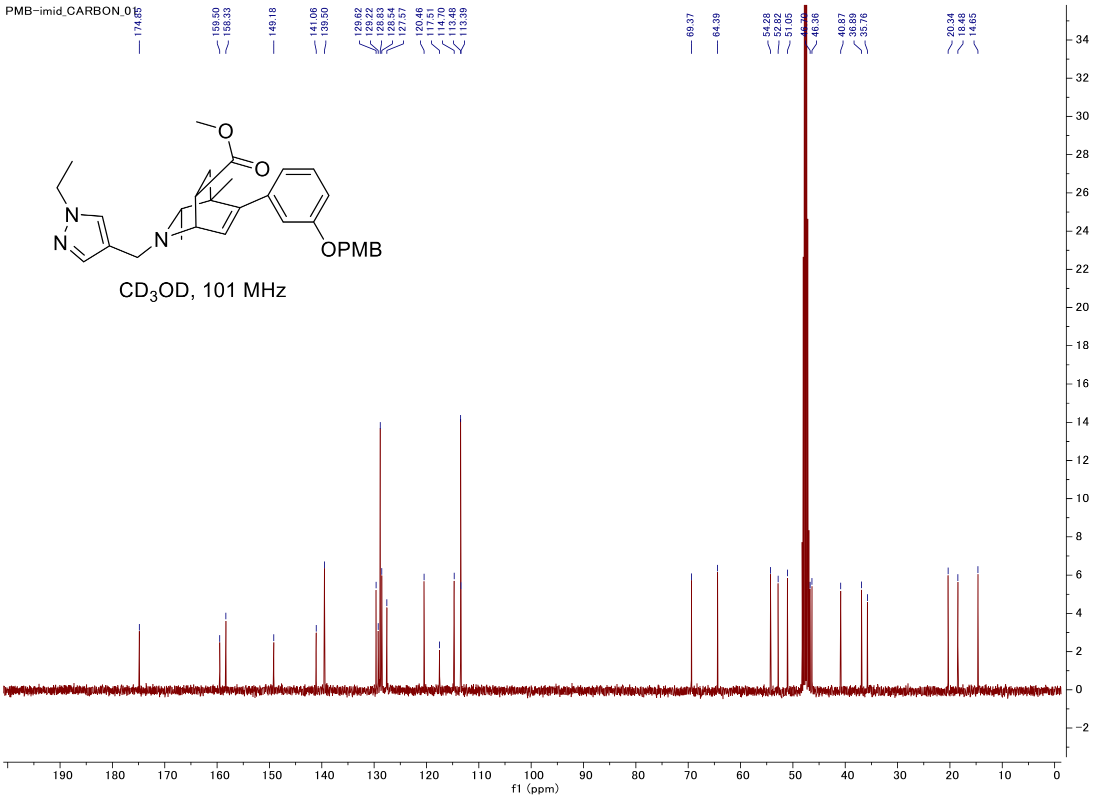

Proton (#020)

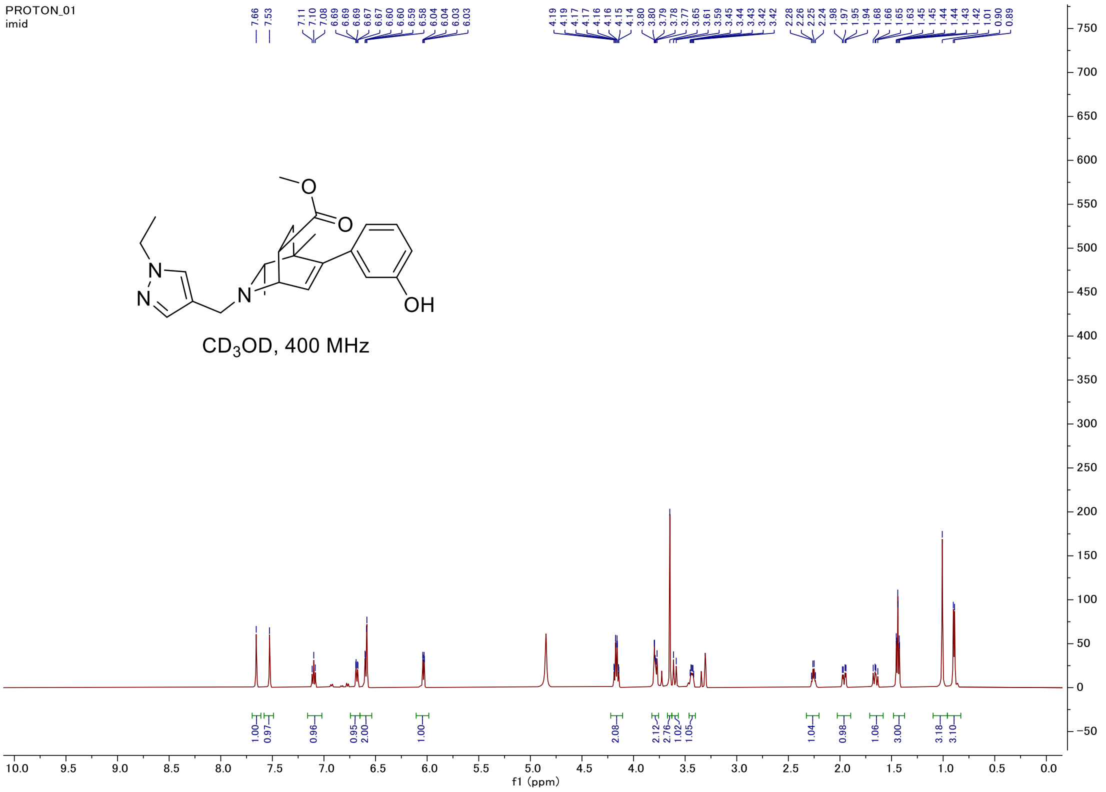

Carbon

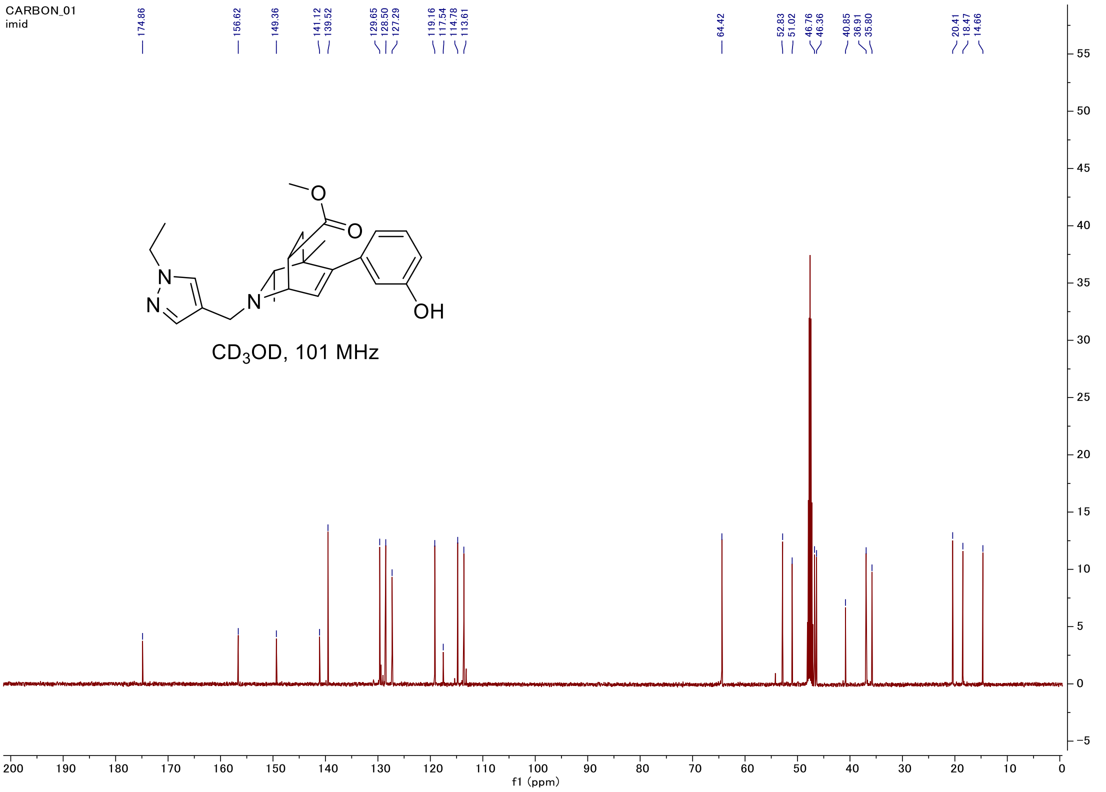

C-H COSY

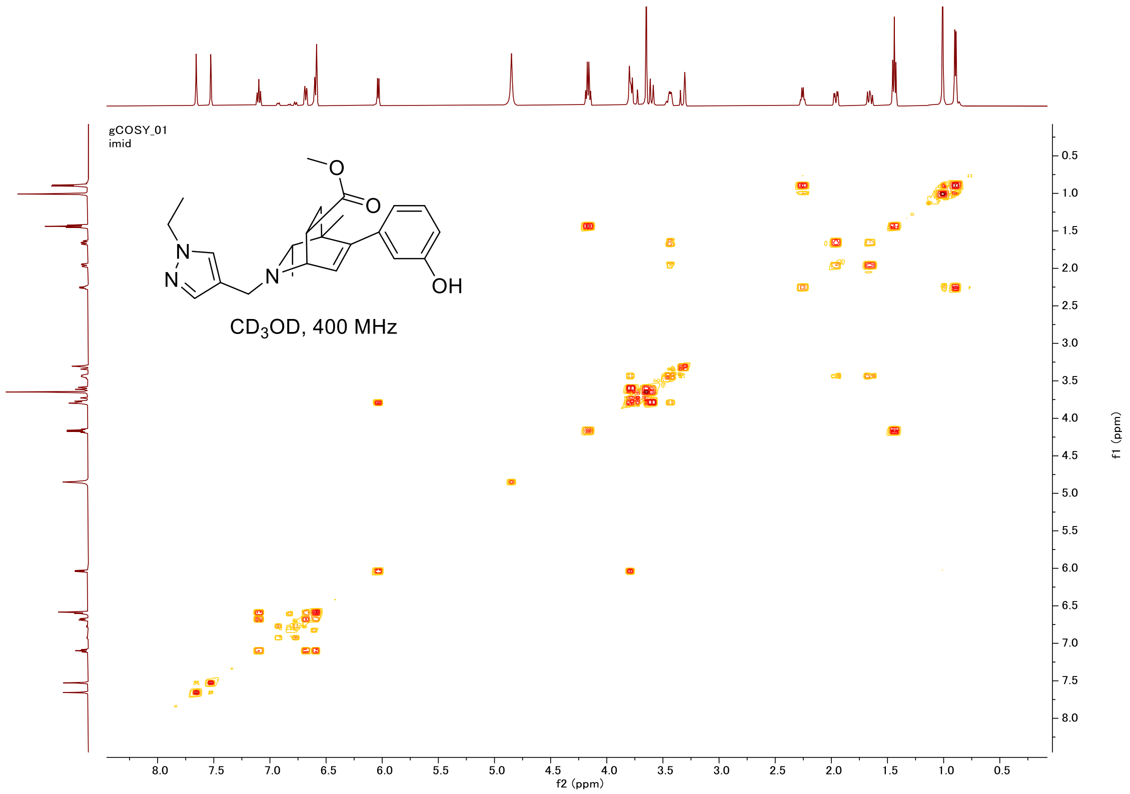
gCOSY_01
imid
N
N
N
O
O
OH
CD3OD, 400 MHz
f2 (ppm)
f1 (ppm)

HSQC

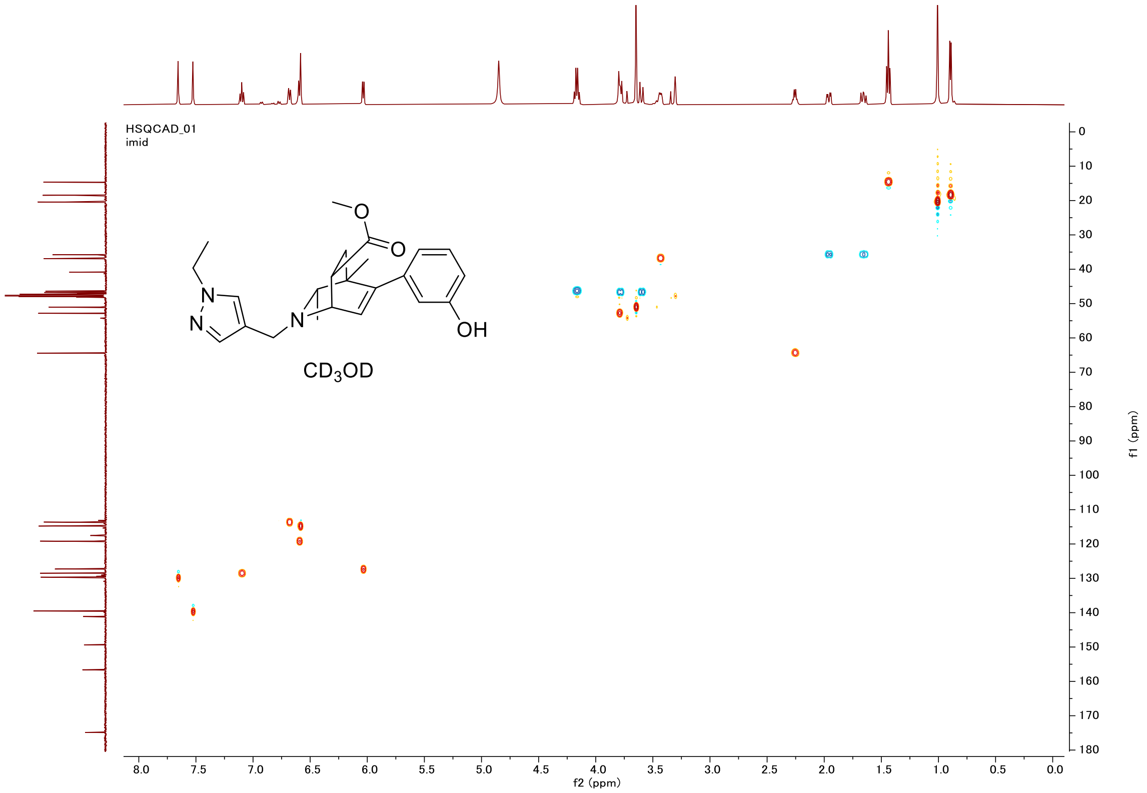
HSQCAD_01
imid
N
N
N
O
O
OH
CD3OD
f2 (ppm)
f1 (ppm)

Proton (#021)

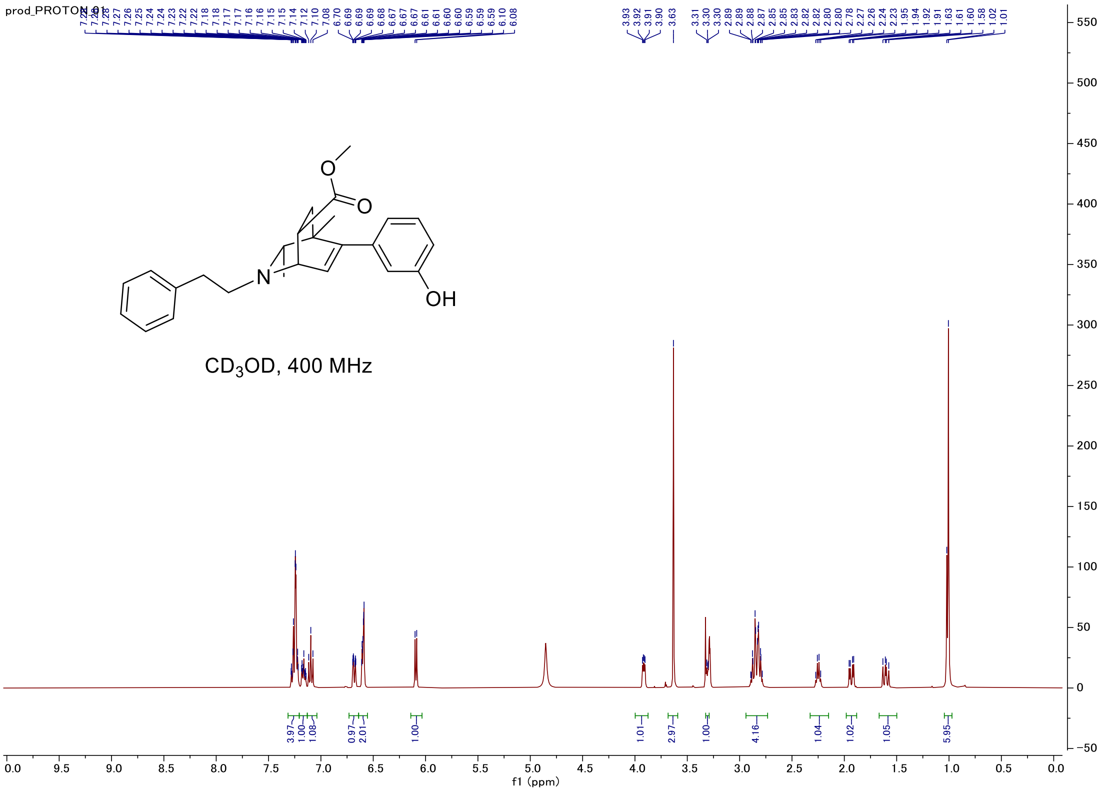

Carbon

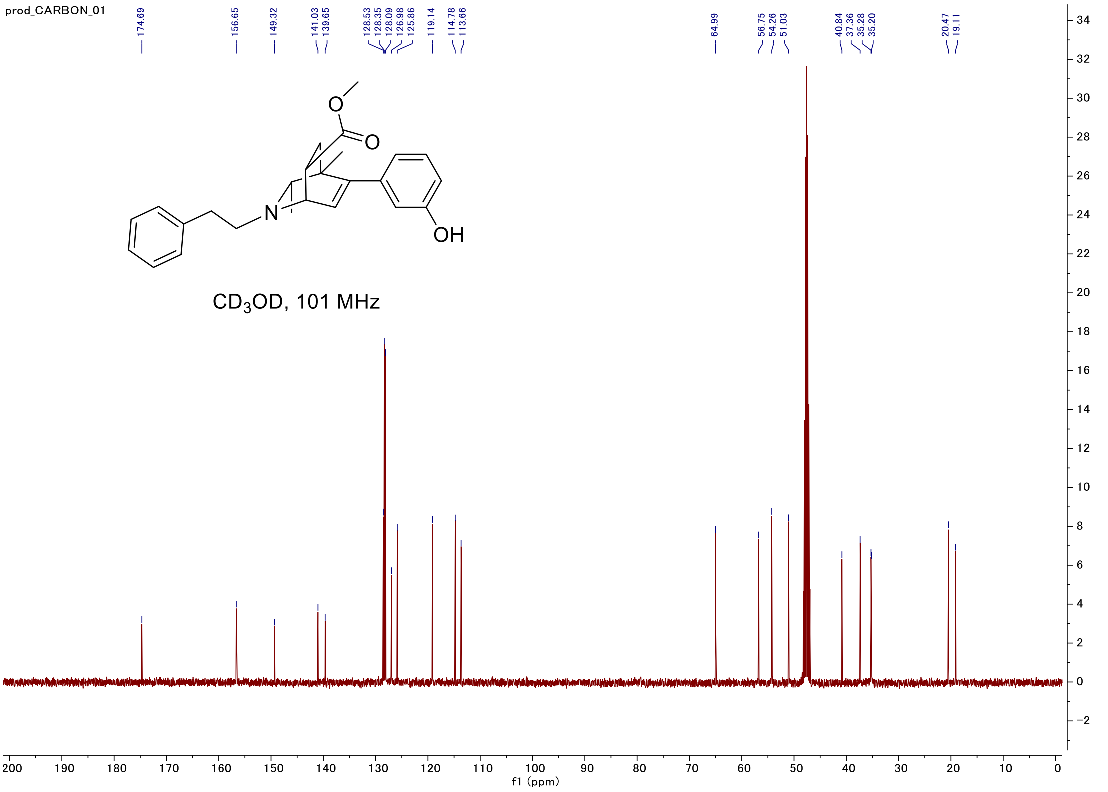

C-H COSY

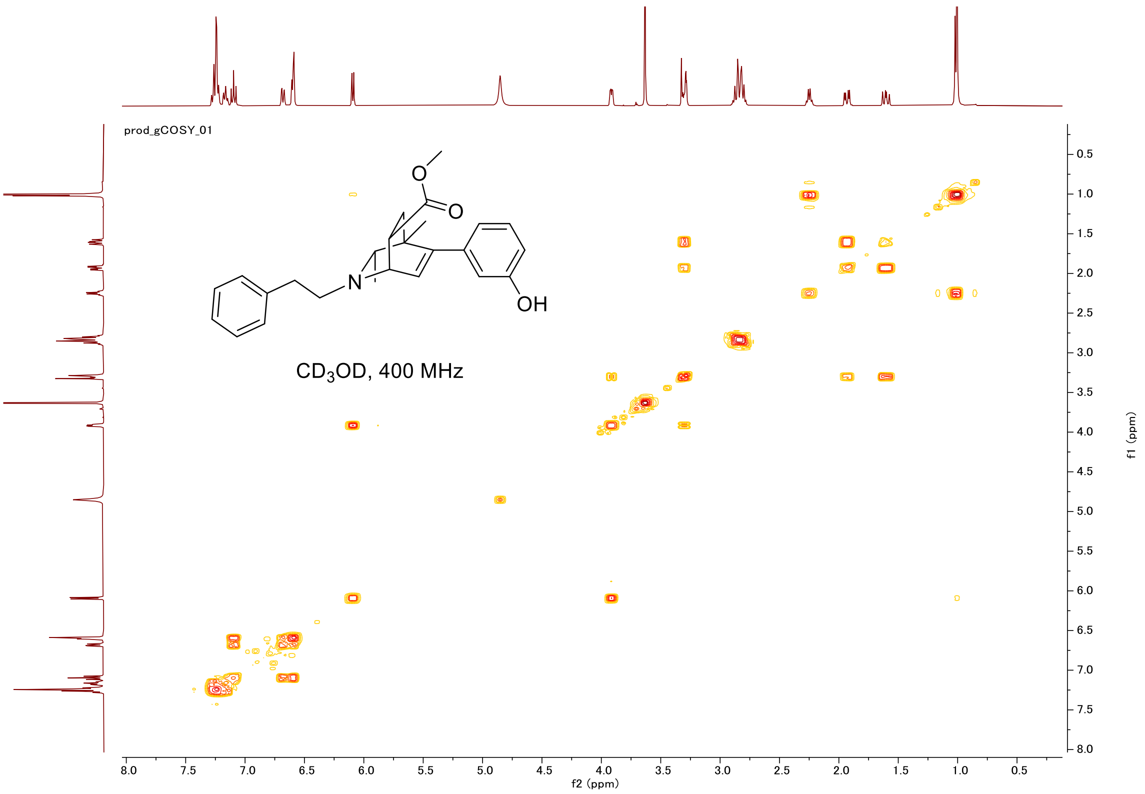
prod_gCOSY_01
CD3OD, 400 MHz
OH
N
O
O
f2 (ppm)
f1 (ppm)

HSQC

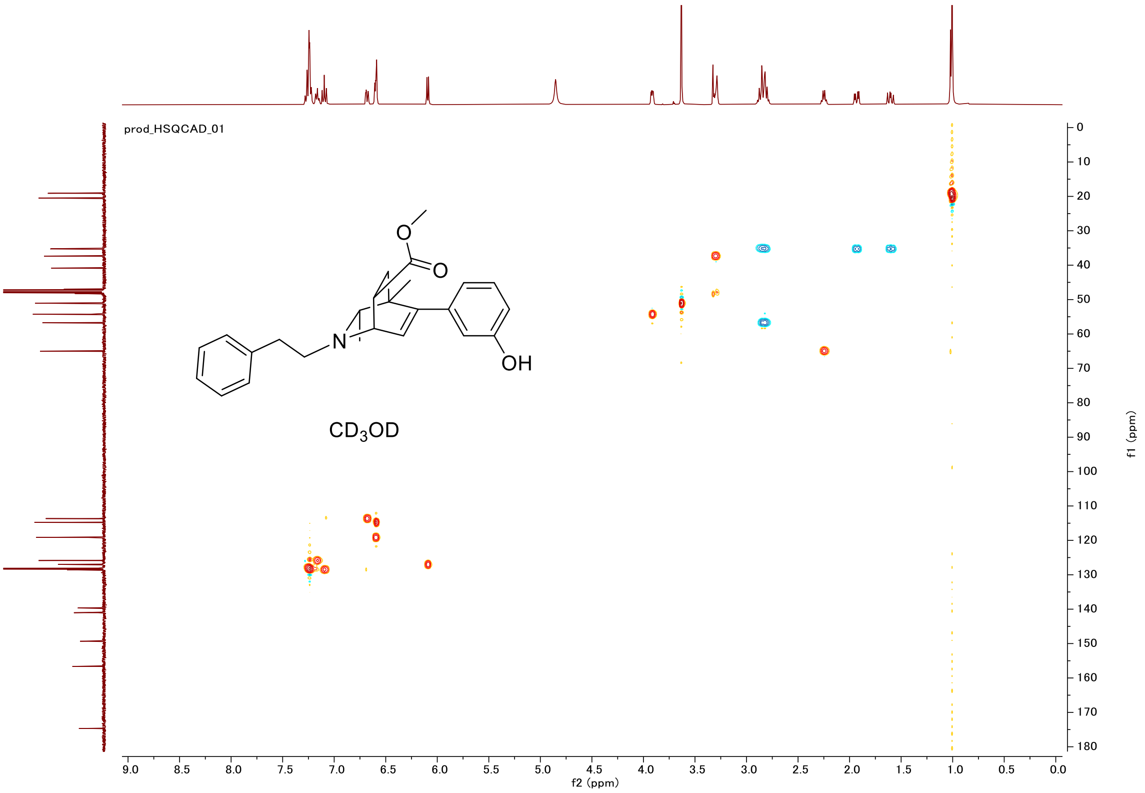
prod_HSQCAD_01
CD3OD
OH
N
O
O
f2 (ppm)
f1 (ppm)

Proton (#022-major)

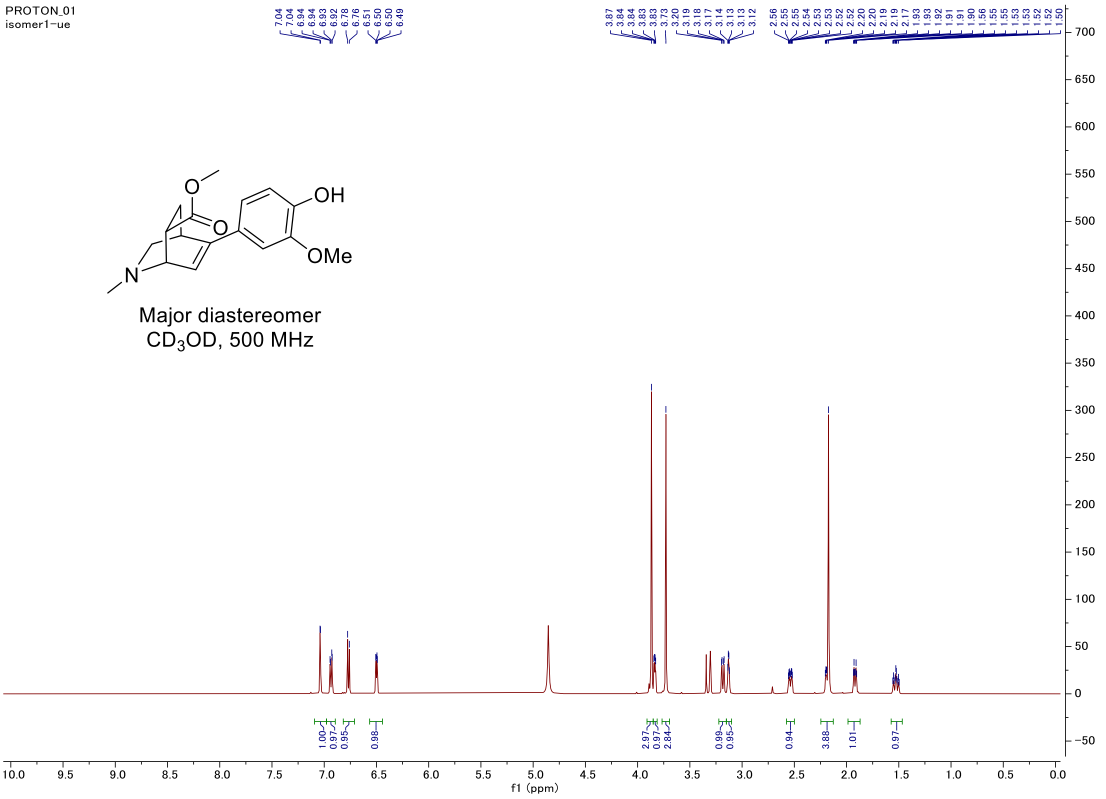

Carbon

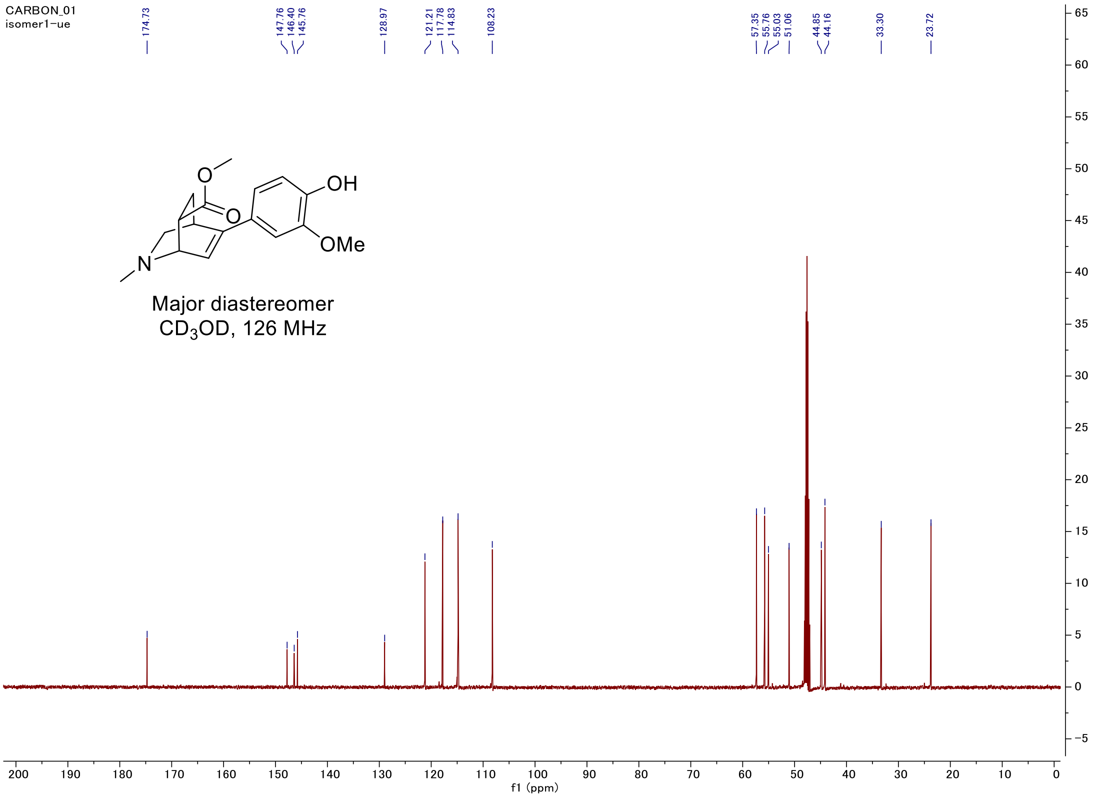

C-H COSY

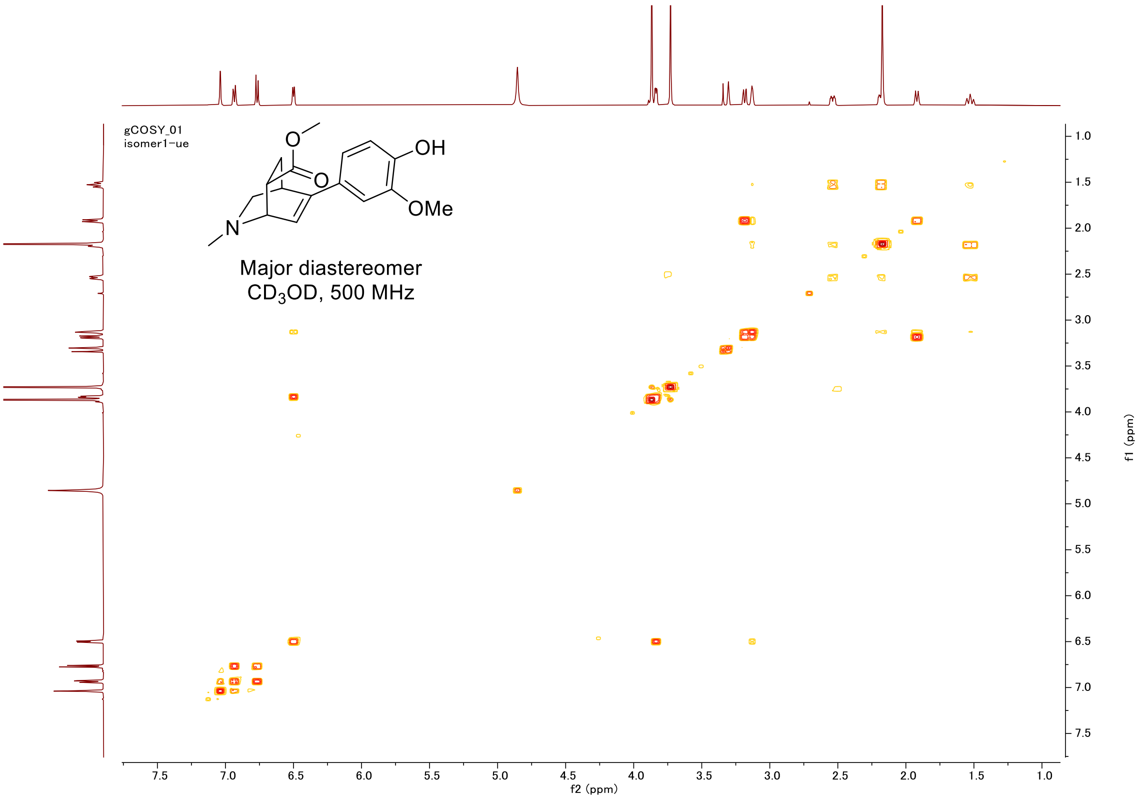
gCOSY_01
isomer1-ue
OH
OMe
O
N
Major diastereomer
CD3OD, 500 MHz
f2 (ppm)
f1 (ppm)

HSQC

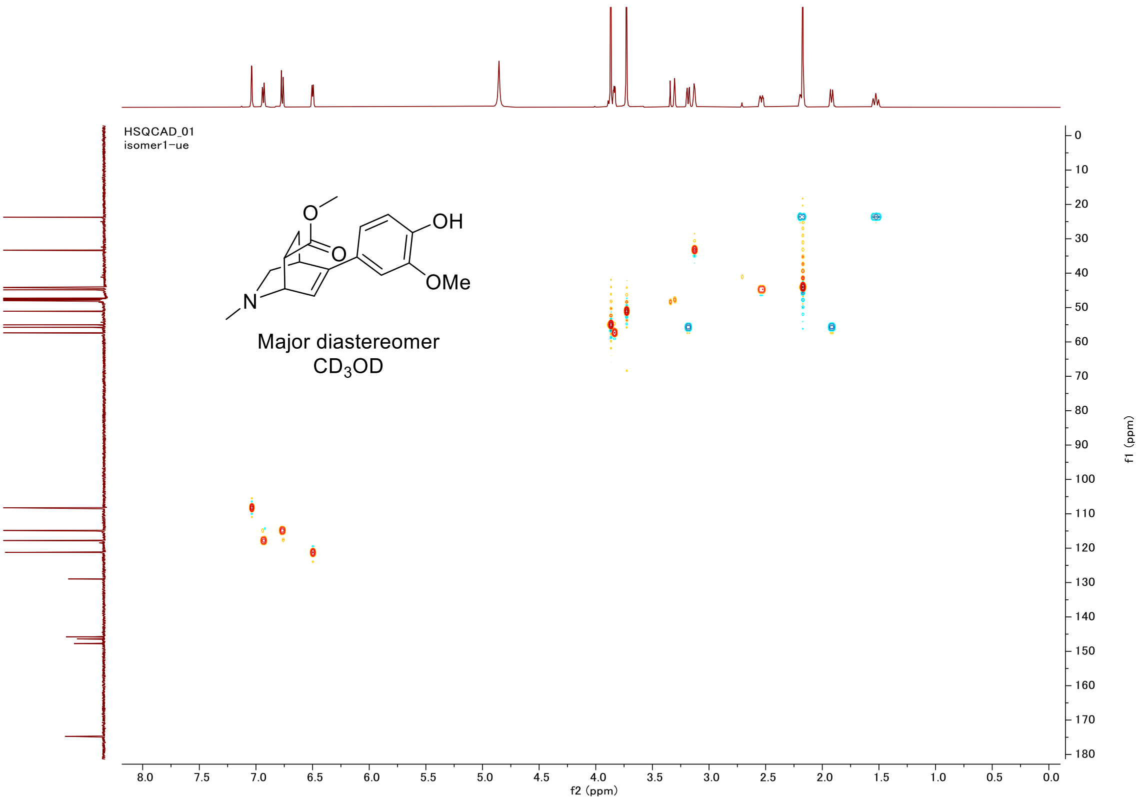
HSQCAD_01
isomer1-ue
OH
OMe
O
N
Major diastereomer
CD3OD
f2 (ppm)
f1 (ppm)

Proton (#022-minor)

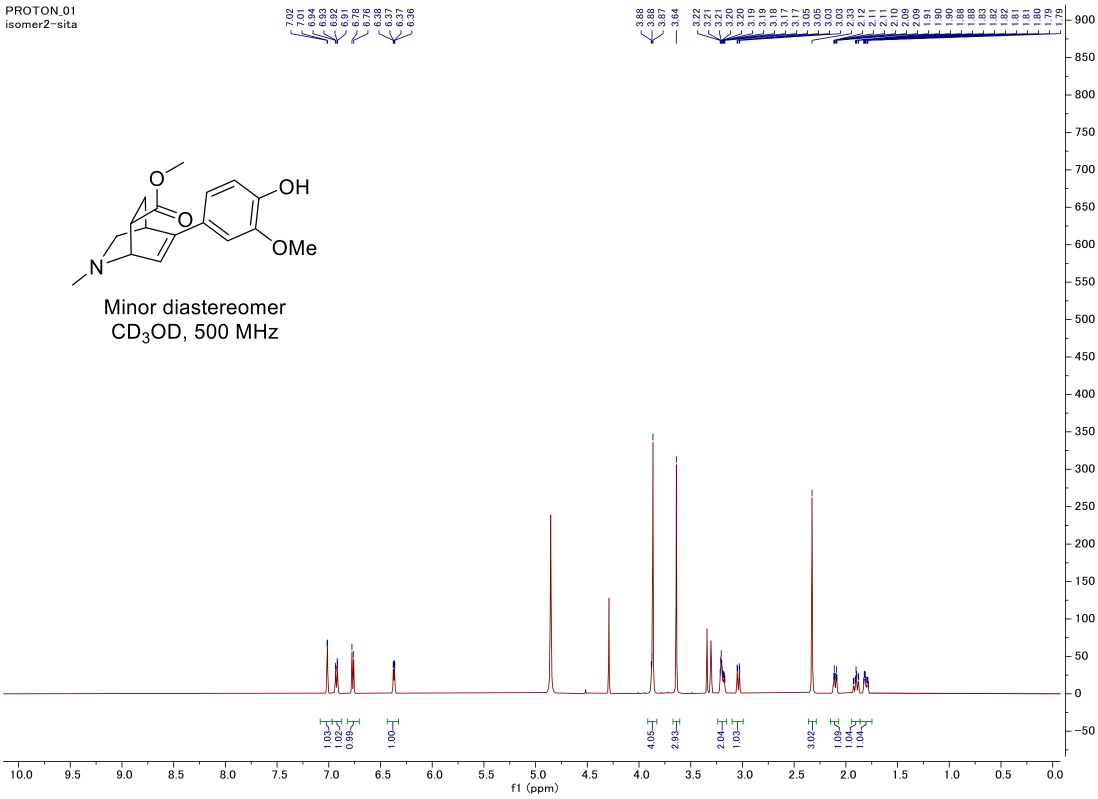

Carbon

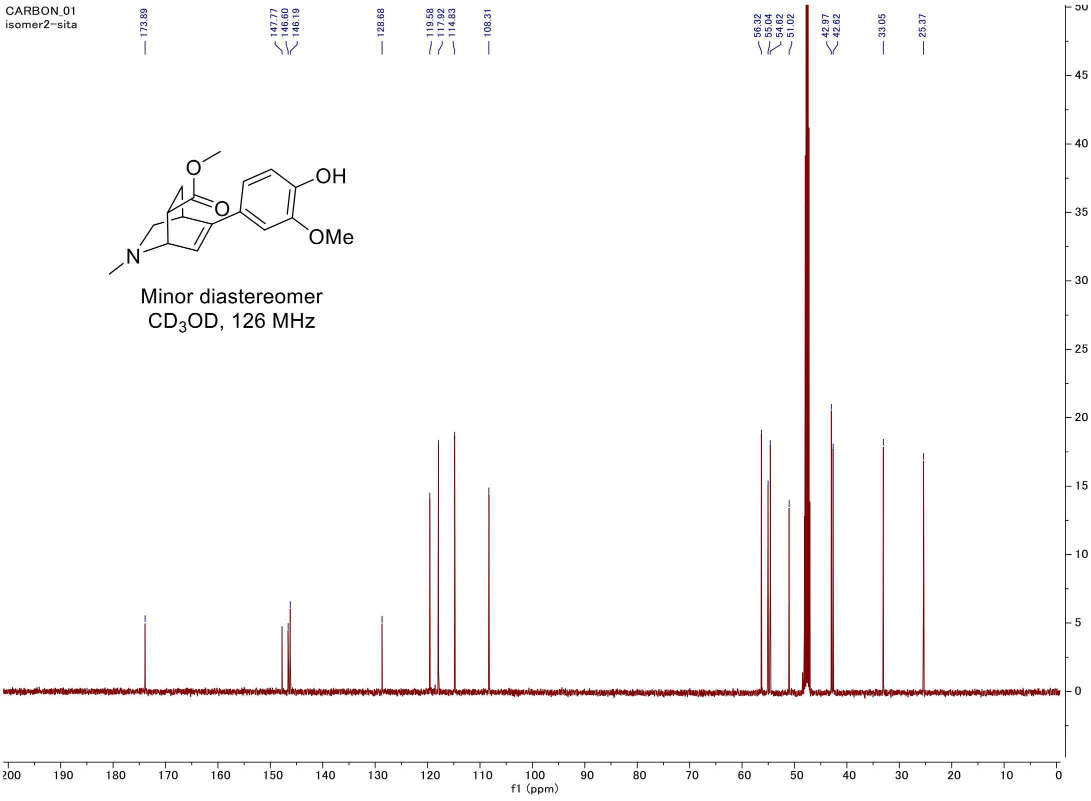

C-H COSY

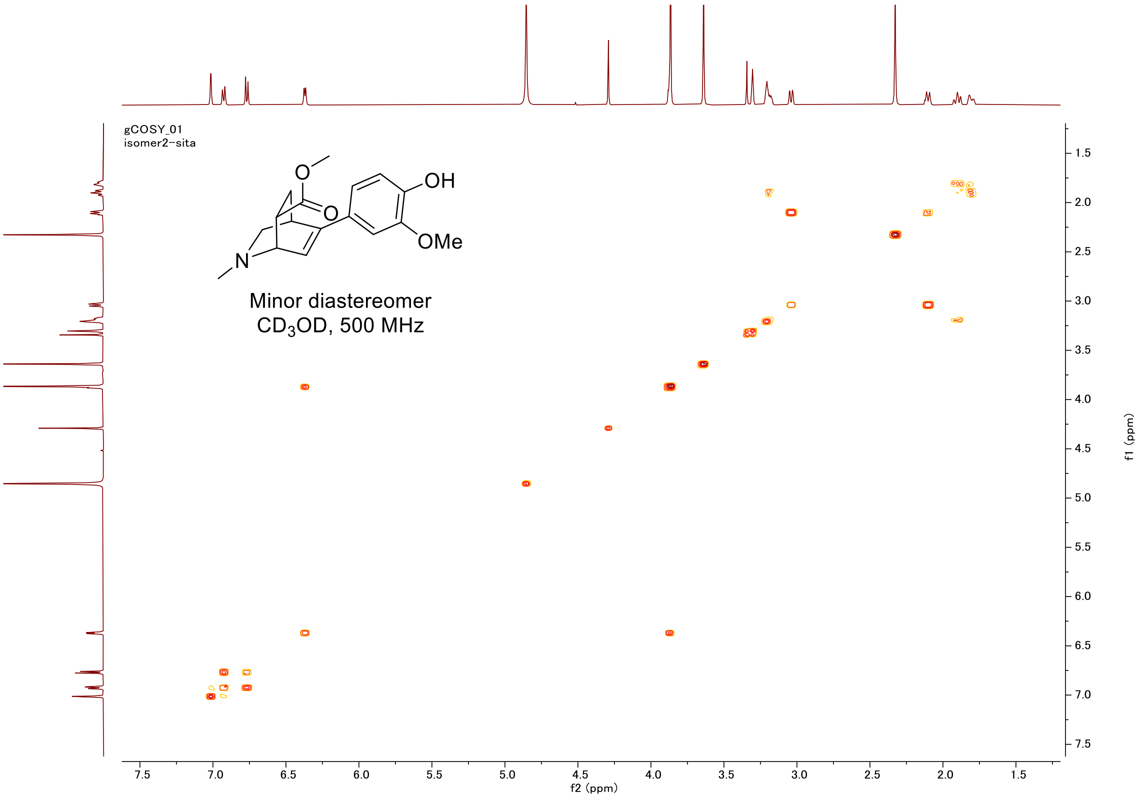
gCOSY_01
isomer2-sita
OH
OMe
O
O
N
Minor diastereomer
CD_3OD, 500 MHz
f2 (ppm)
f1 (ppm)

HSQC

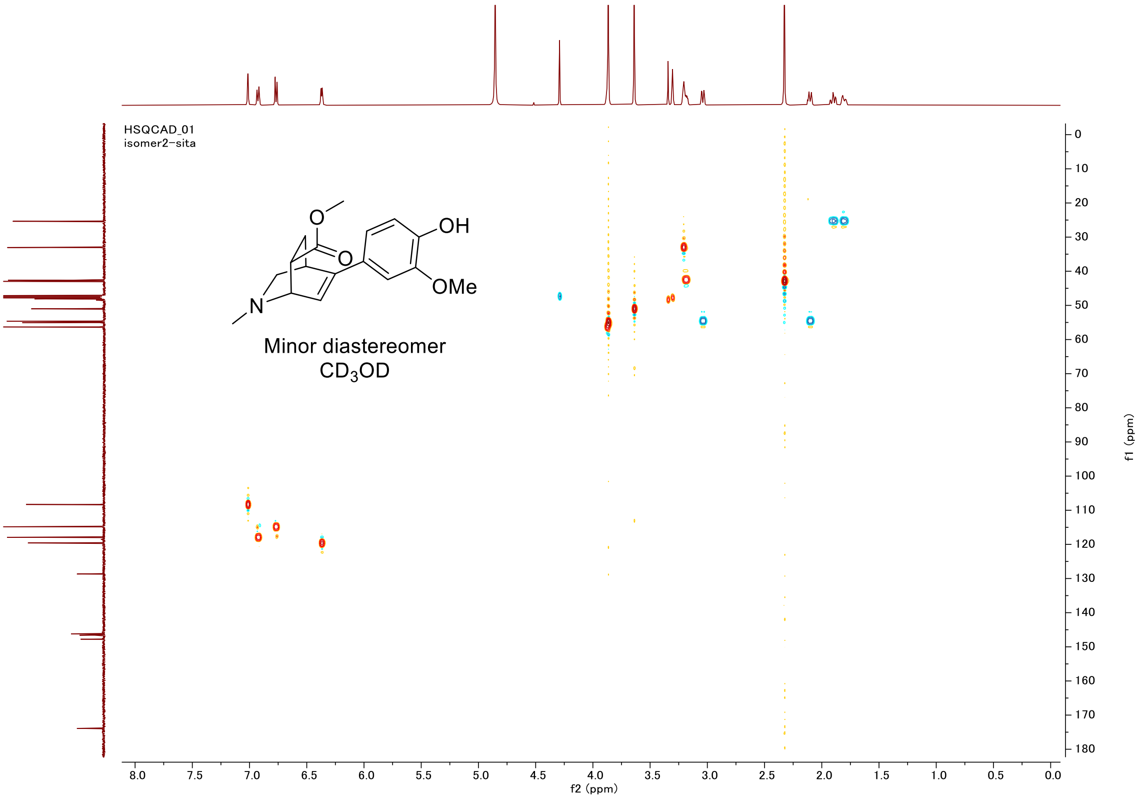
HSQCAD_01
isomer2-sita
OH
OMe
O
O
N
Minor diastereomer
CD_3OD
f2 (ppm)
f1 (ppm)

Proton (#023)

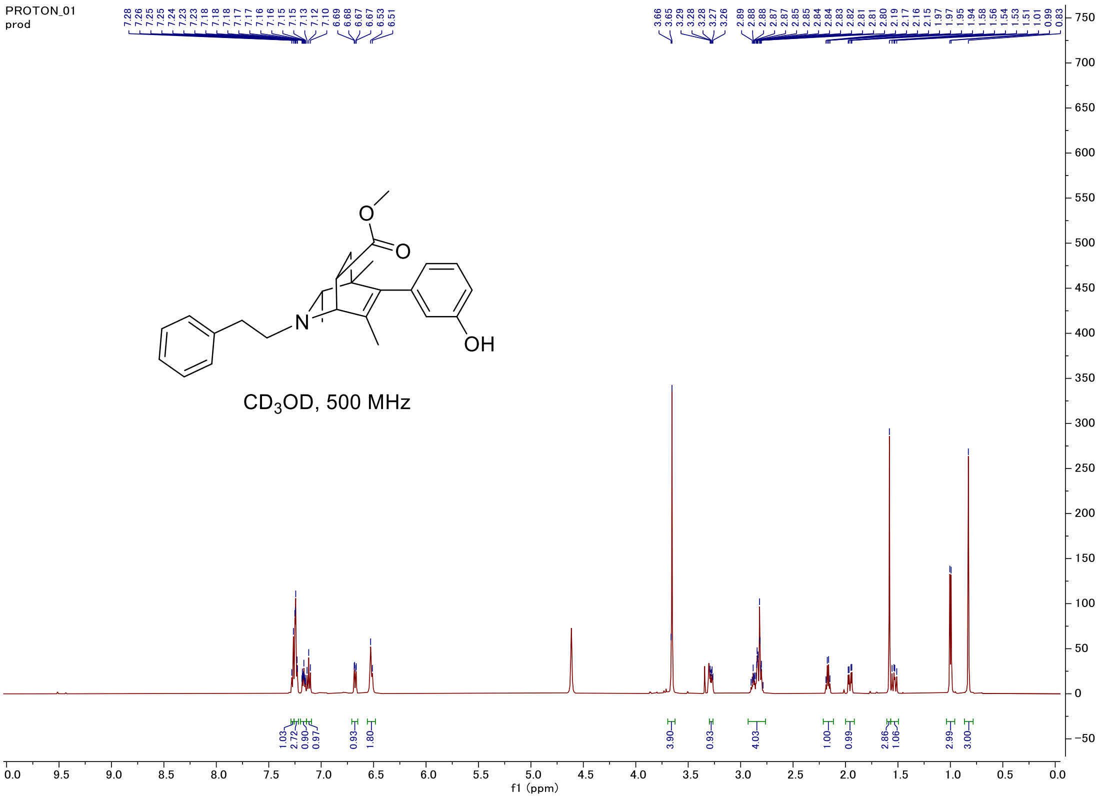

Carbon

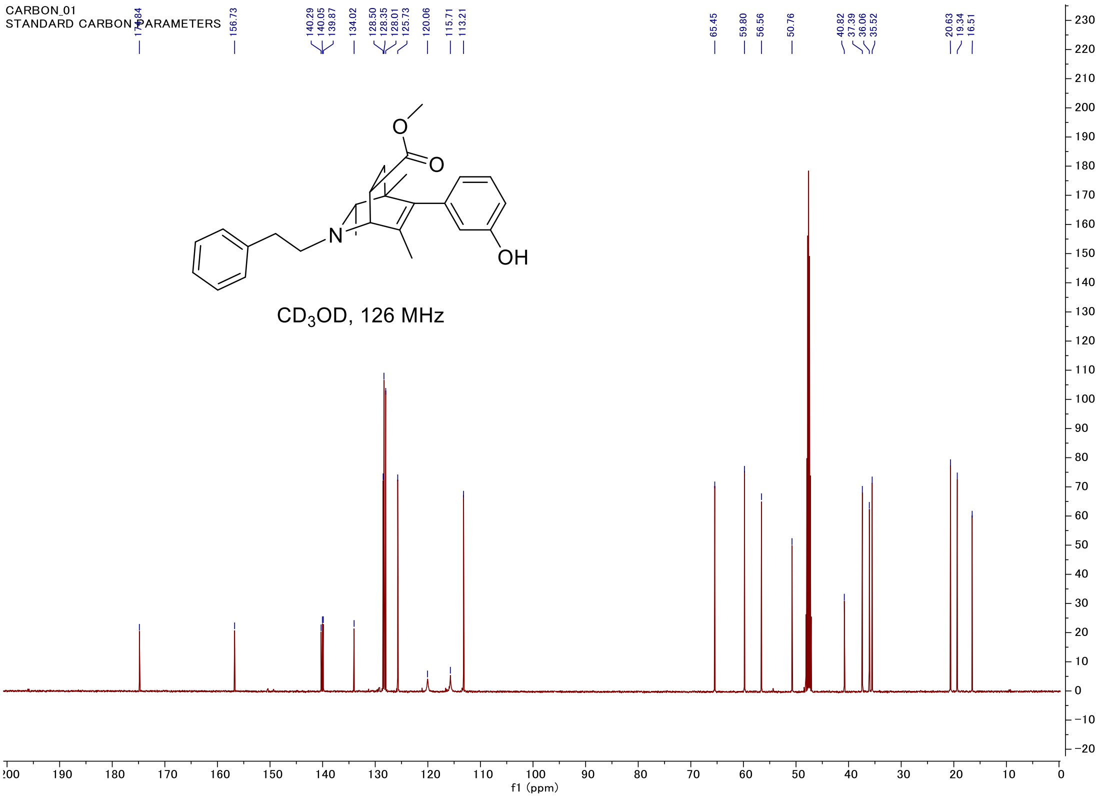

C-H COSY

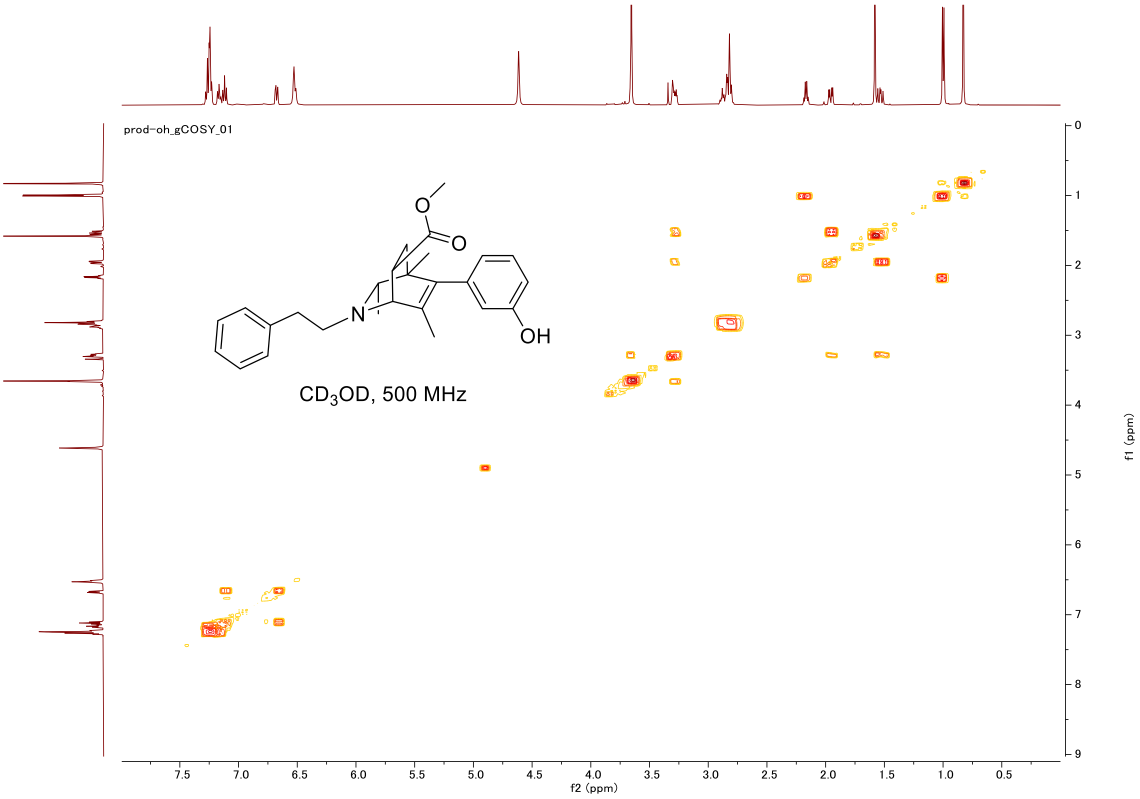
prod-oh_gCOSY_01
OH
N
O
O
CD3OD, 500 MHz
f2 (ppm)
f1 (ppm)

HSQC

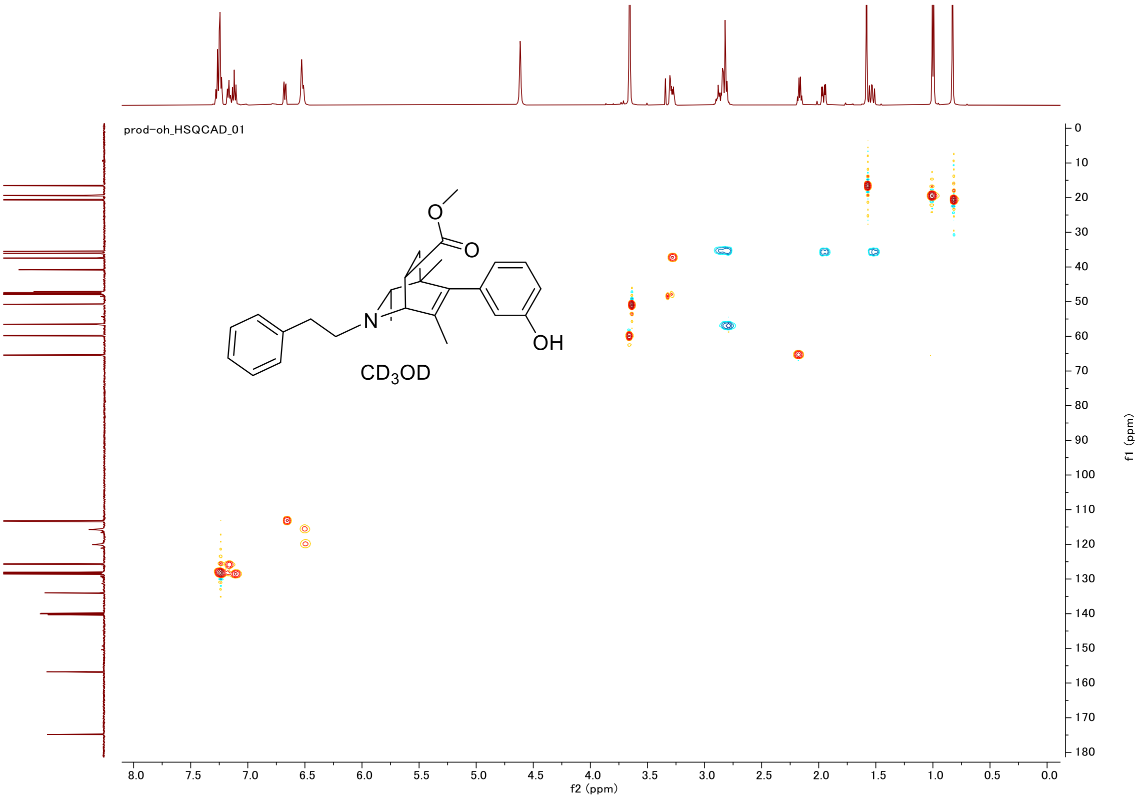
prod-oh_HSQCAD_01
OH
N
O
O
CD3OD
f2 (ppm)
f1 (ppm)

Proton (#024)

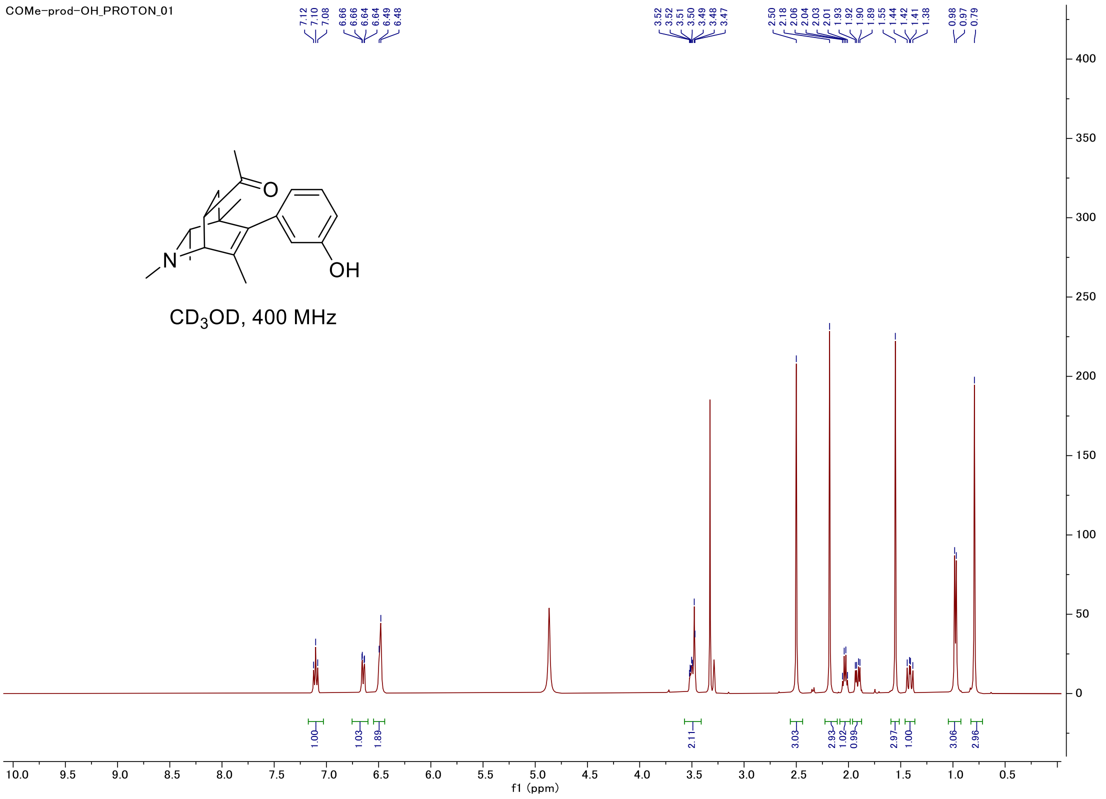

Carbon

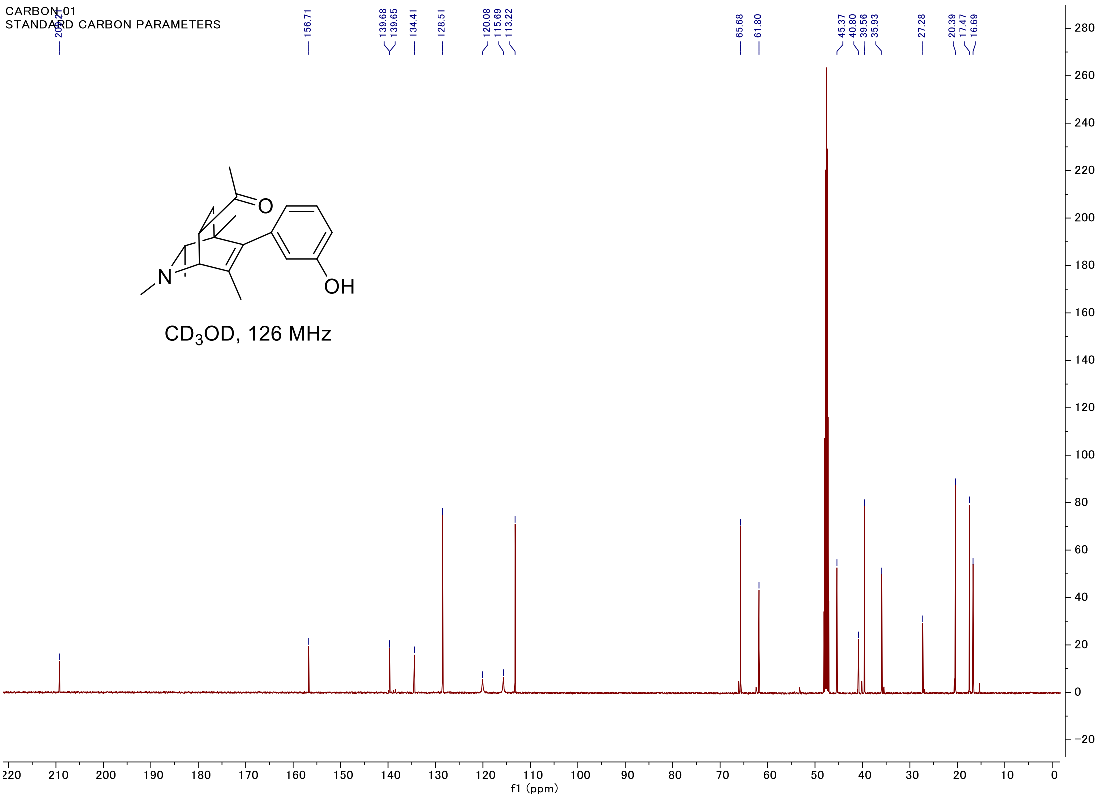

C-H COSY

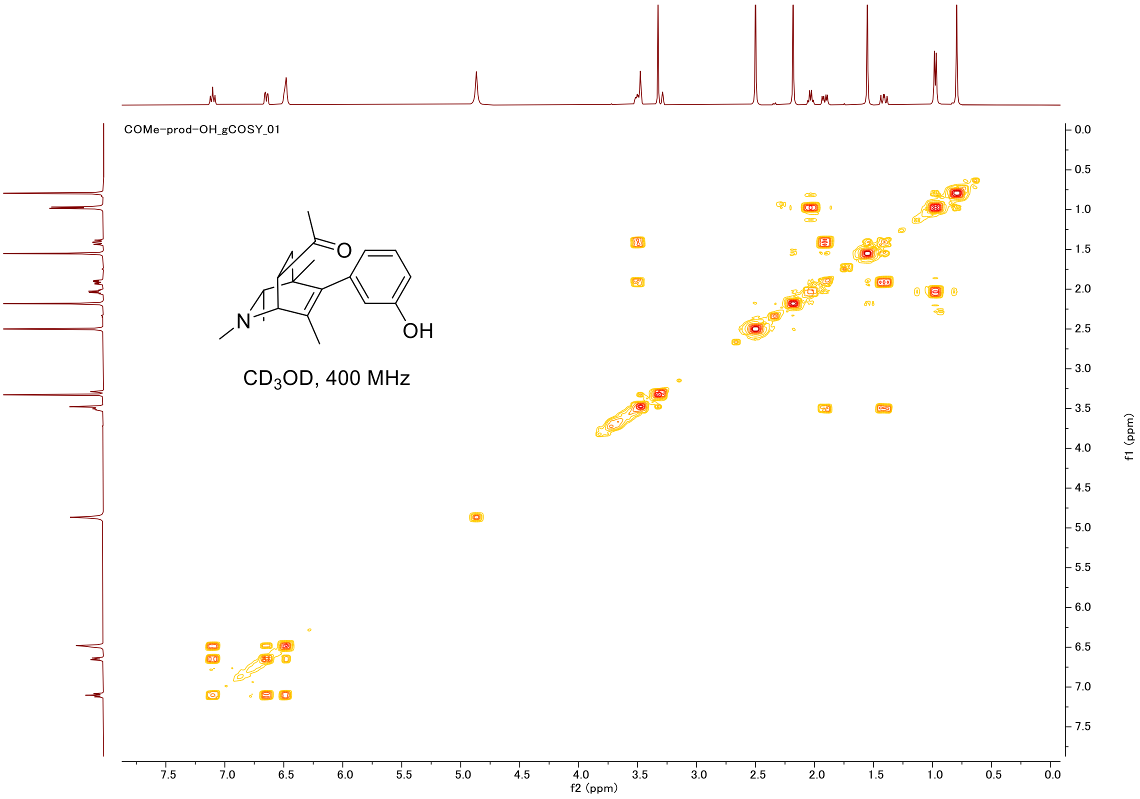
COMe-prod-OH_gCOSY_01
N
O
OH
CD3OD, 400 MHz
f2 (ppm)
f1 (ppm)

HSQC

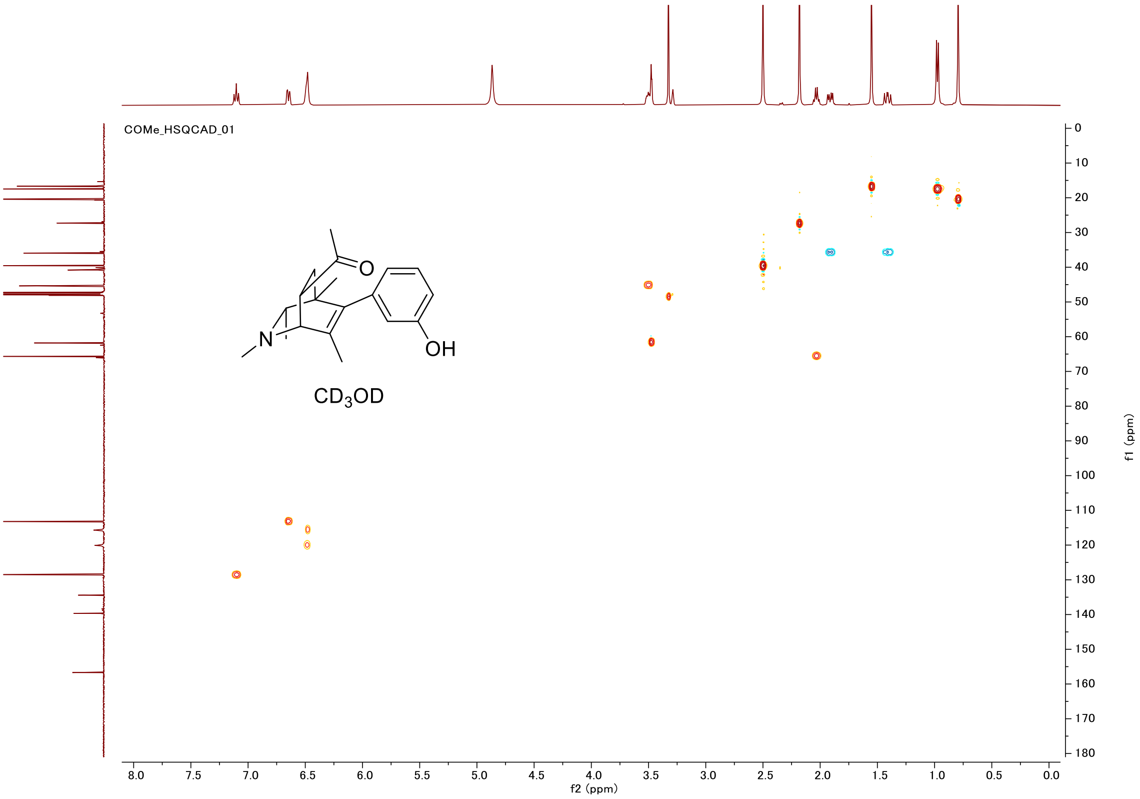
COMe_HSQCAD_01
N
O
OH
CD3OD
f2 (ppm)
f1 (ppm)

Proton (#025)

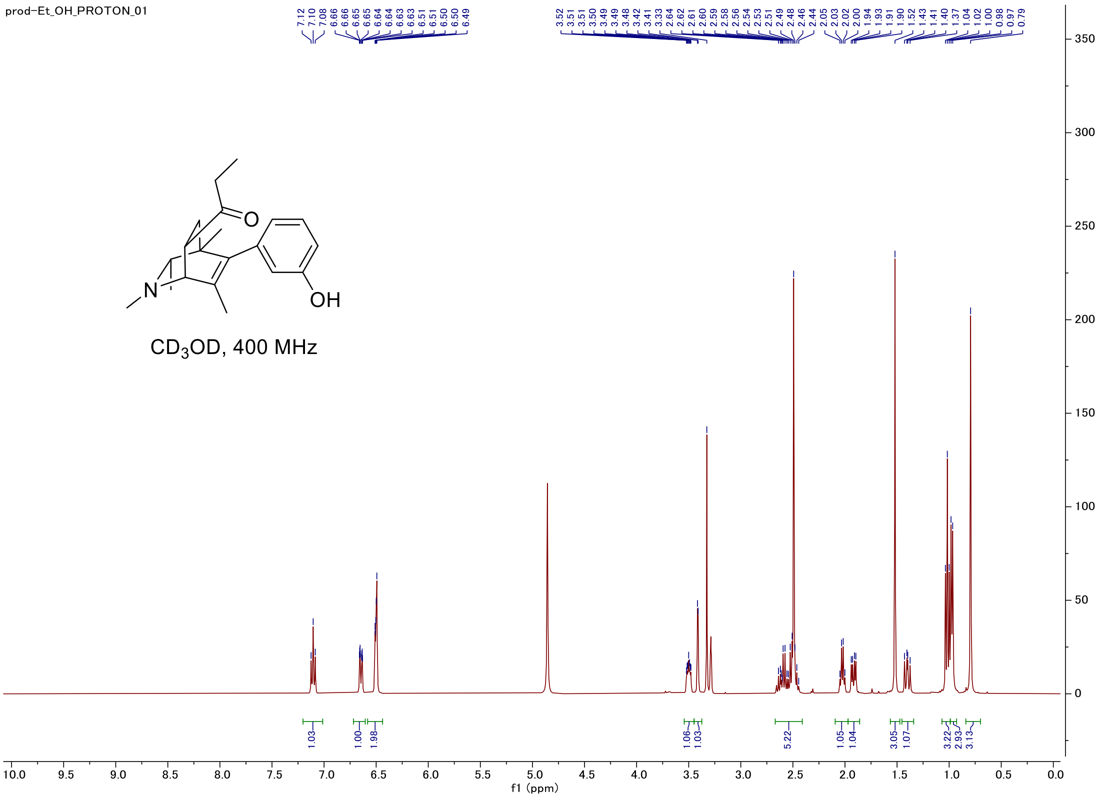

Carbon

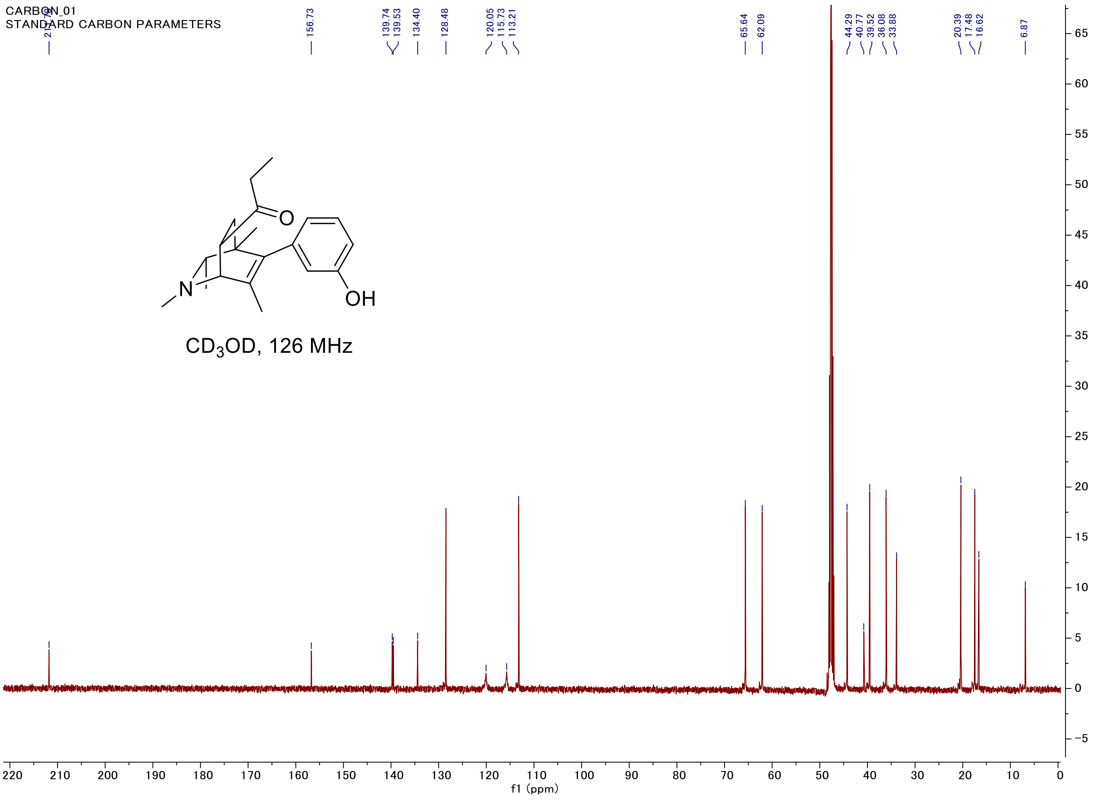

C-H COSY

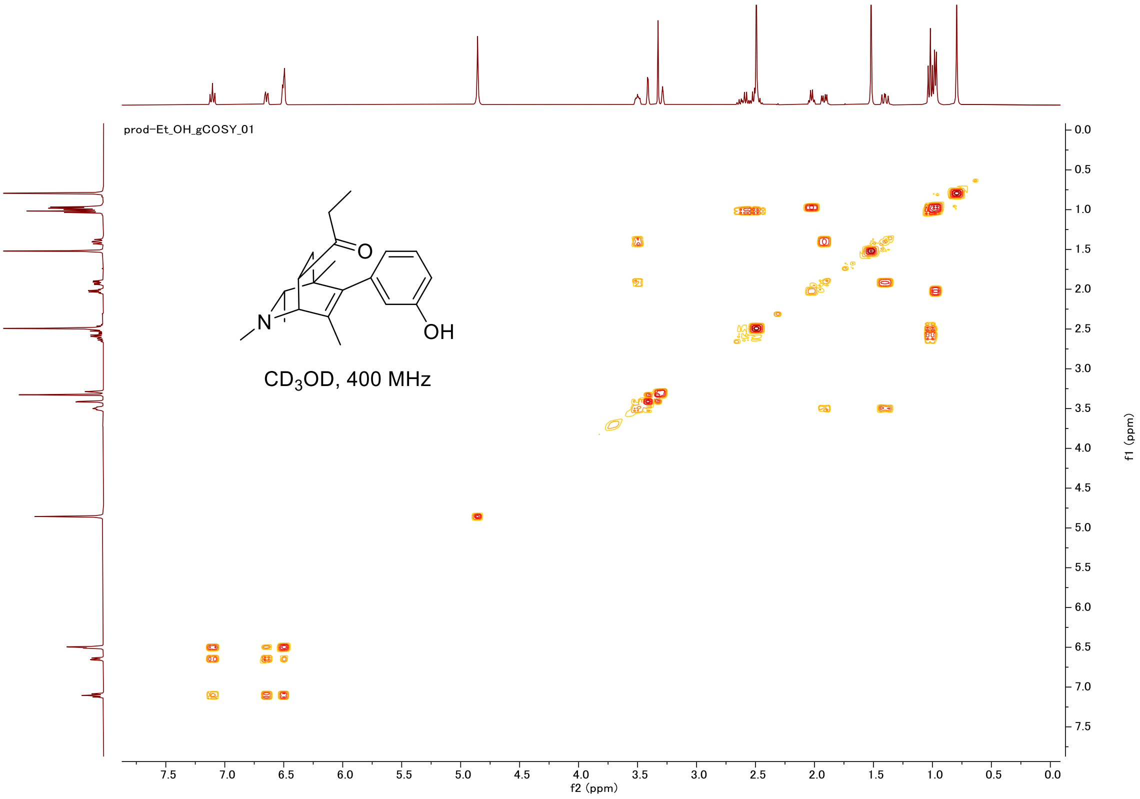
prod-Et_OH_gCOSY_01
N
OH
O
CD3OD, 400 MHz
f2 (ppm)
f1 (ppm)

HSQC

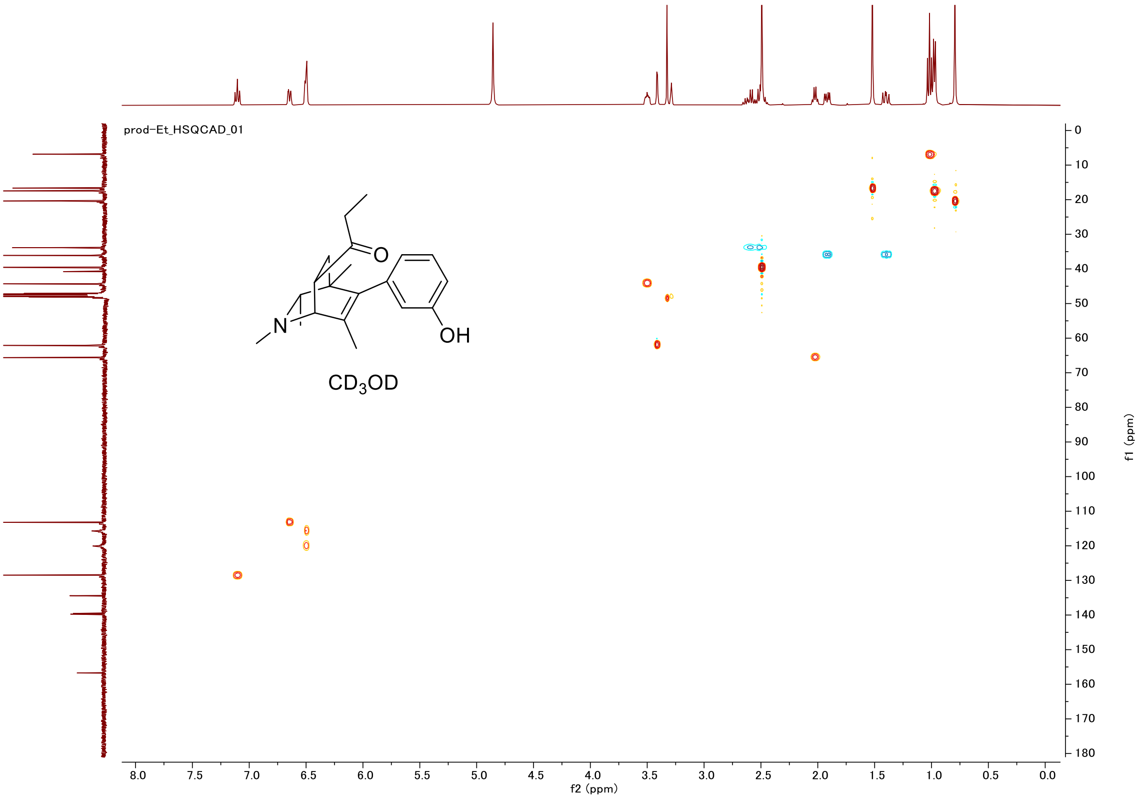
prod-Et_HSQCAD_01
N
OH
O
CD3OD
f2 (ppm)
f1 (ppm)

Proton (#026)

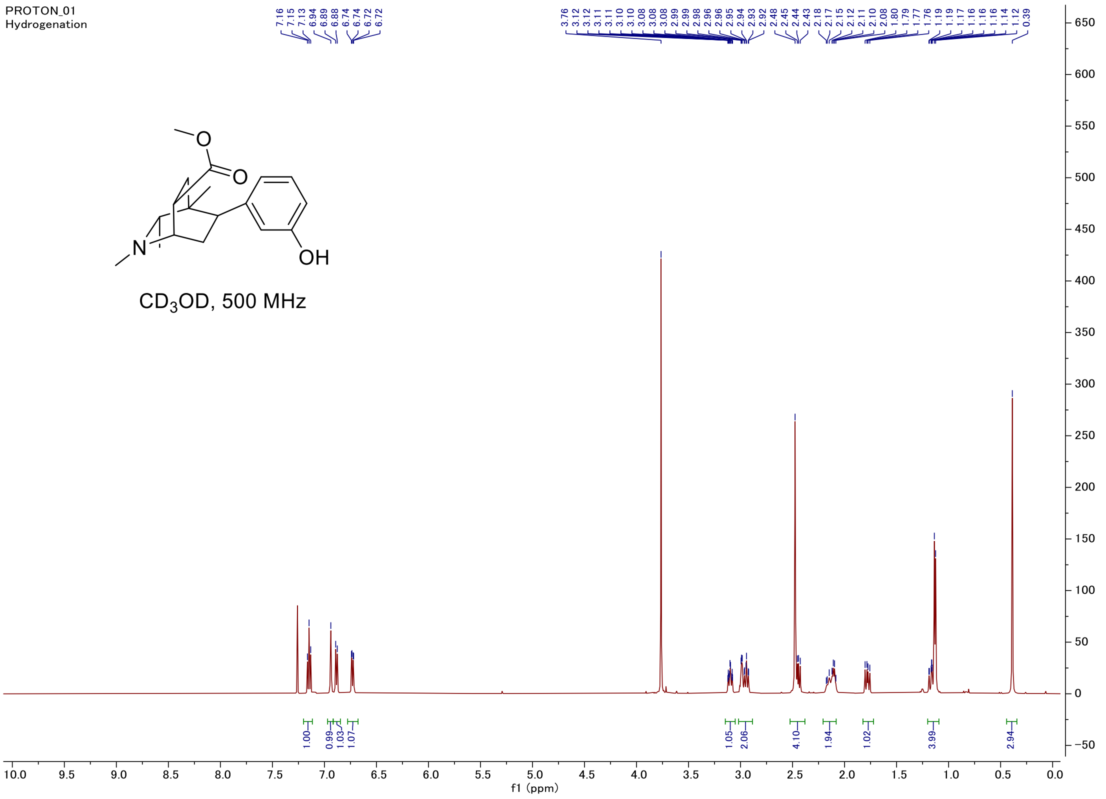

Carbon

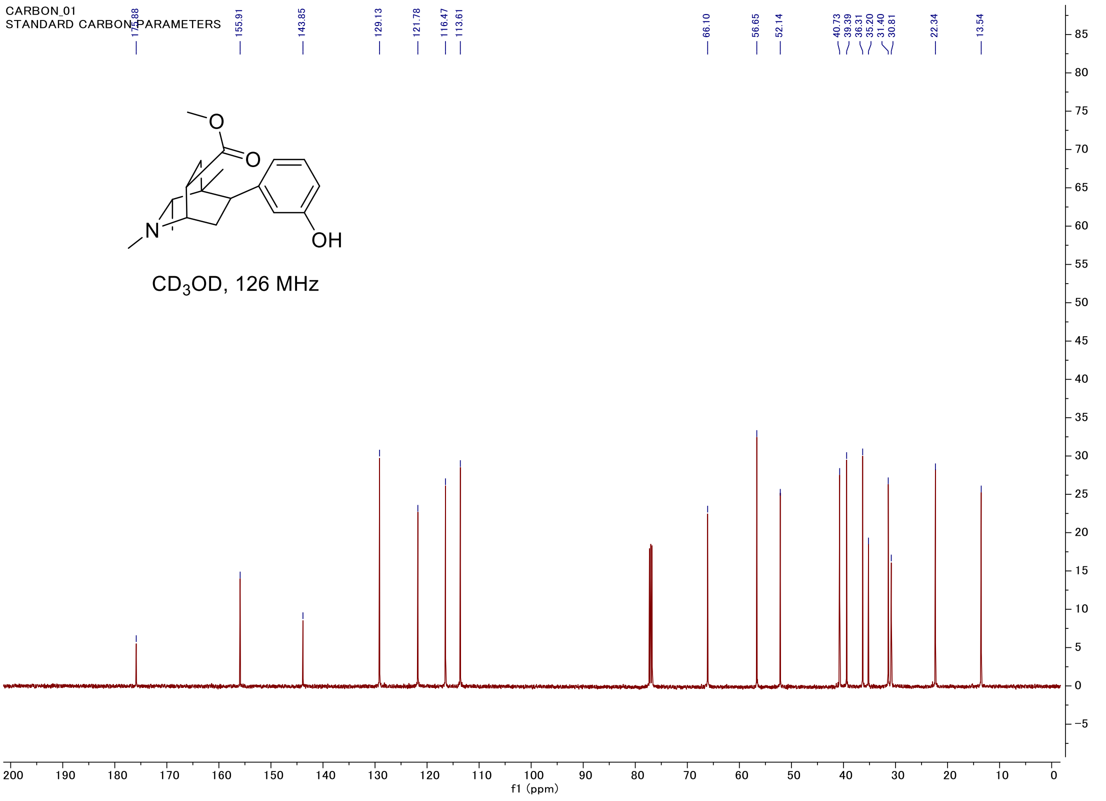

C-H COSY

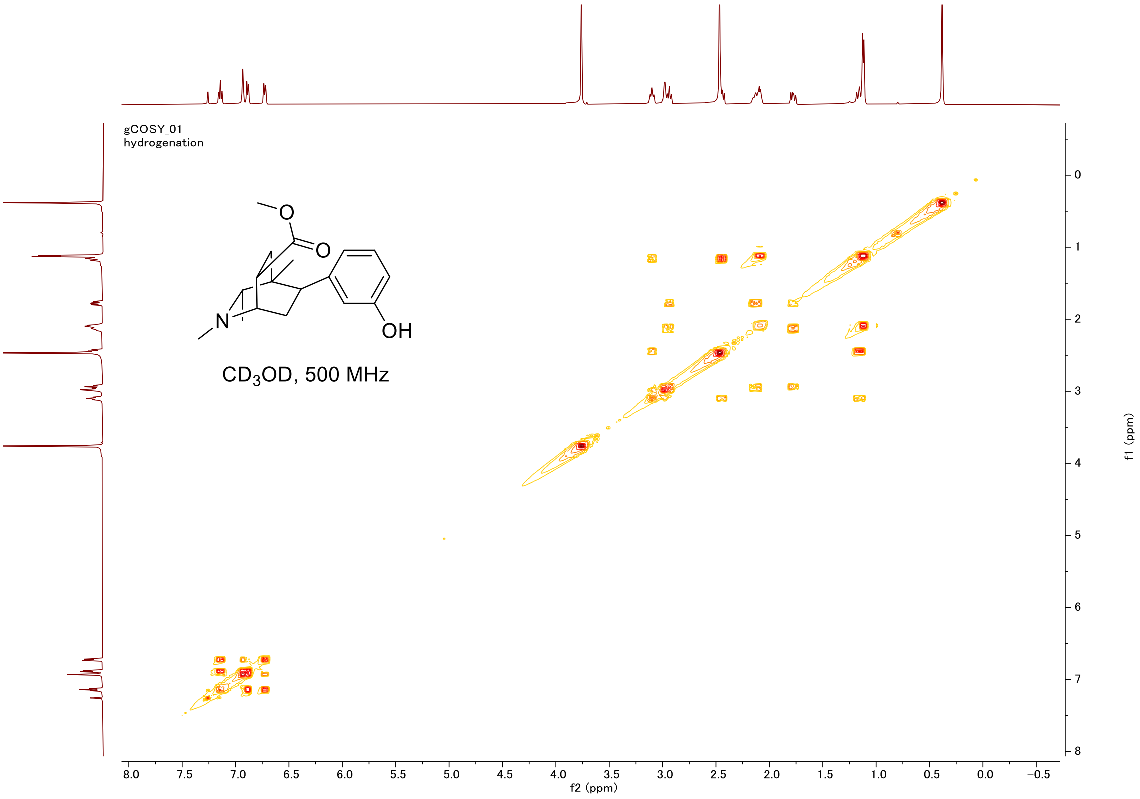
gCOSY_01
hydrogenation
OH
N
O
O
CD$_3$OD, 500 MHz
f2 (ppm)
f1 (ppm)

HSQC

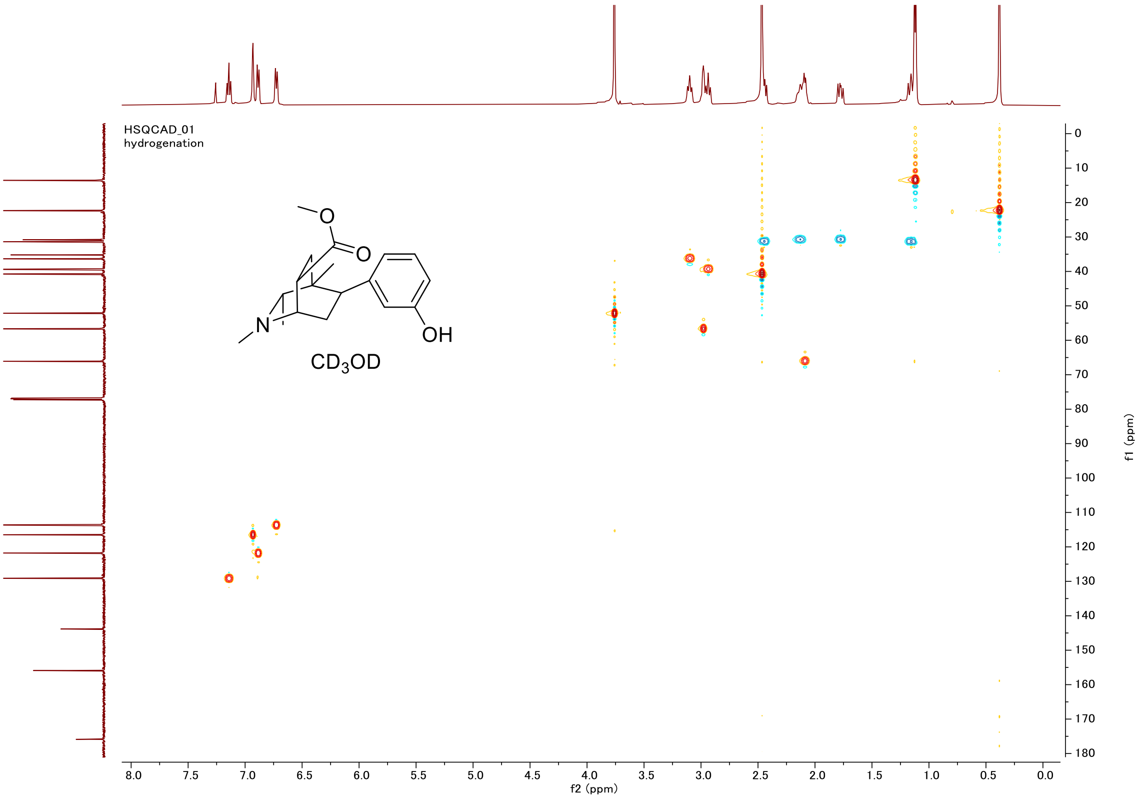
HSQCAD_01
hydrogenation
OH
N
O
O
CD$_3$OD
f2 (ppm)
f1 (ppm)

Proton (#027)

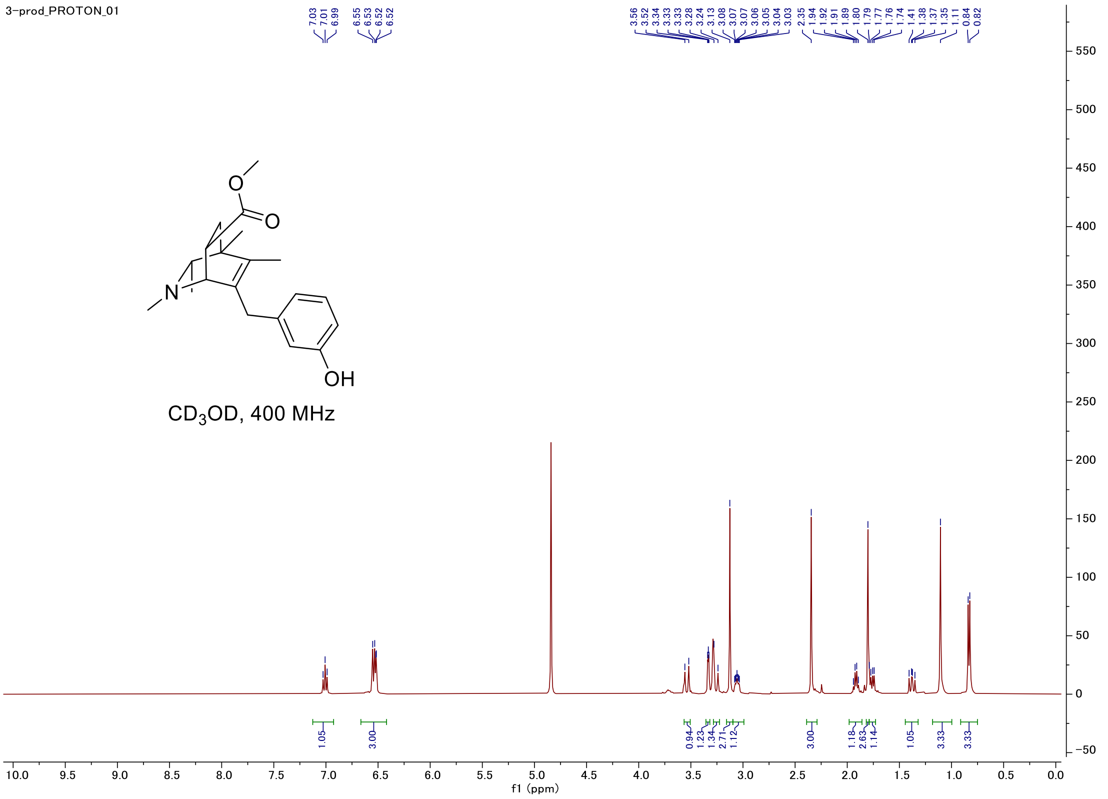

Carbon

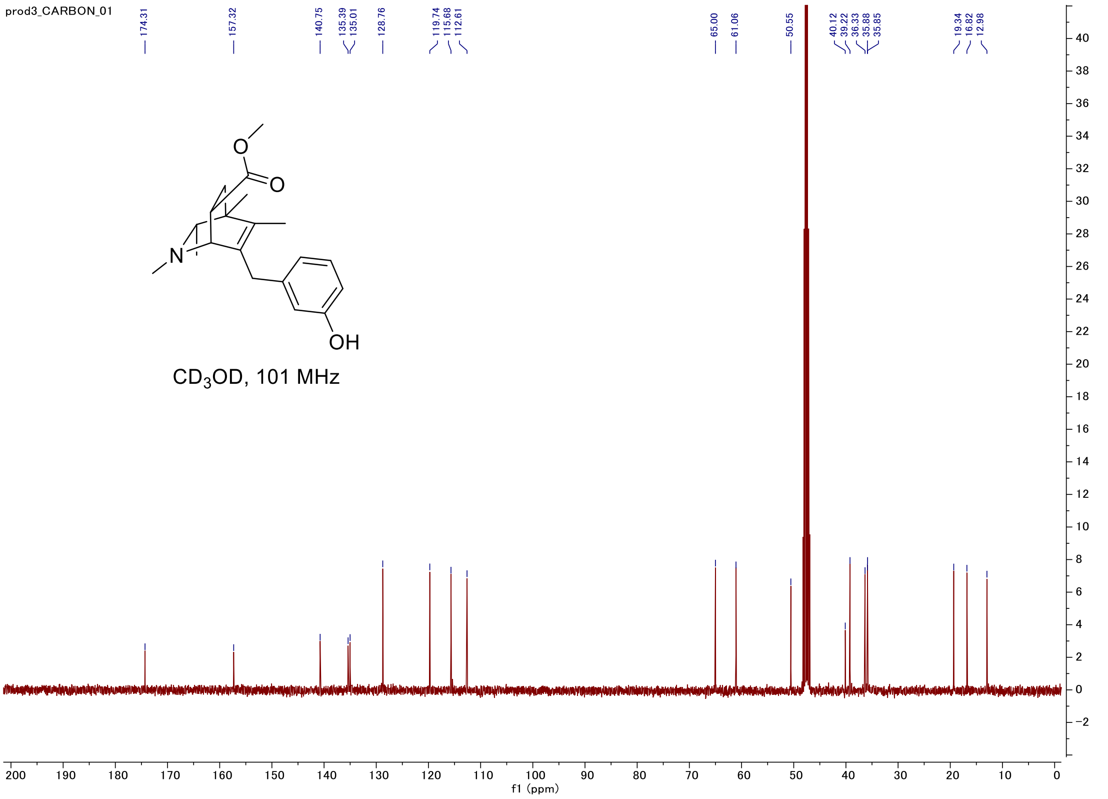

C-H COSY

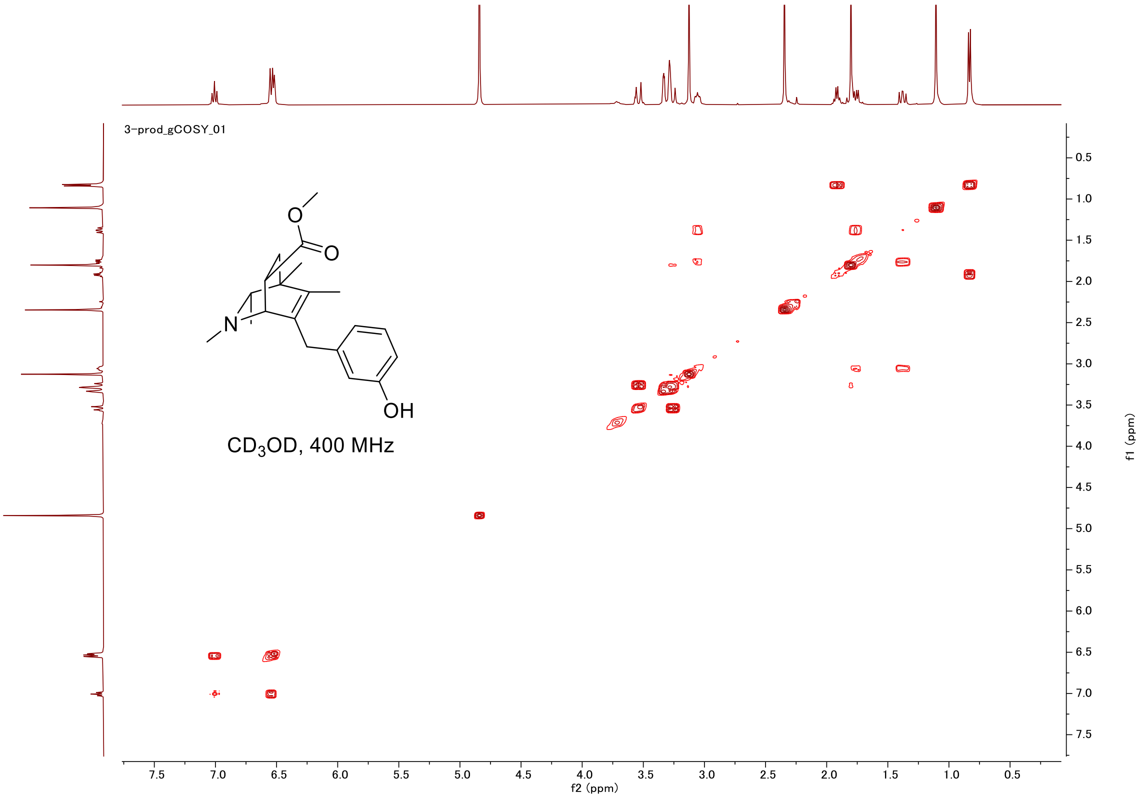
3-prod_gCOSY_01
O
O
N
OH
CD3OD, 400 MHz
f2 (ppm)
f1 (ppm)

HSQC

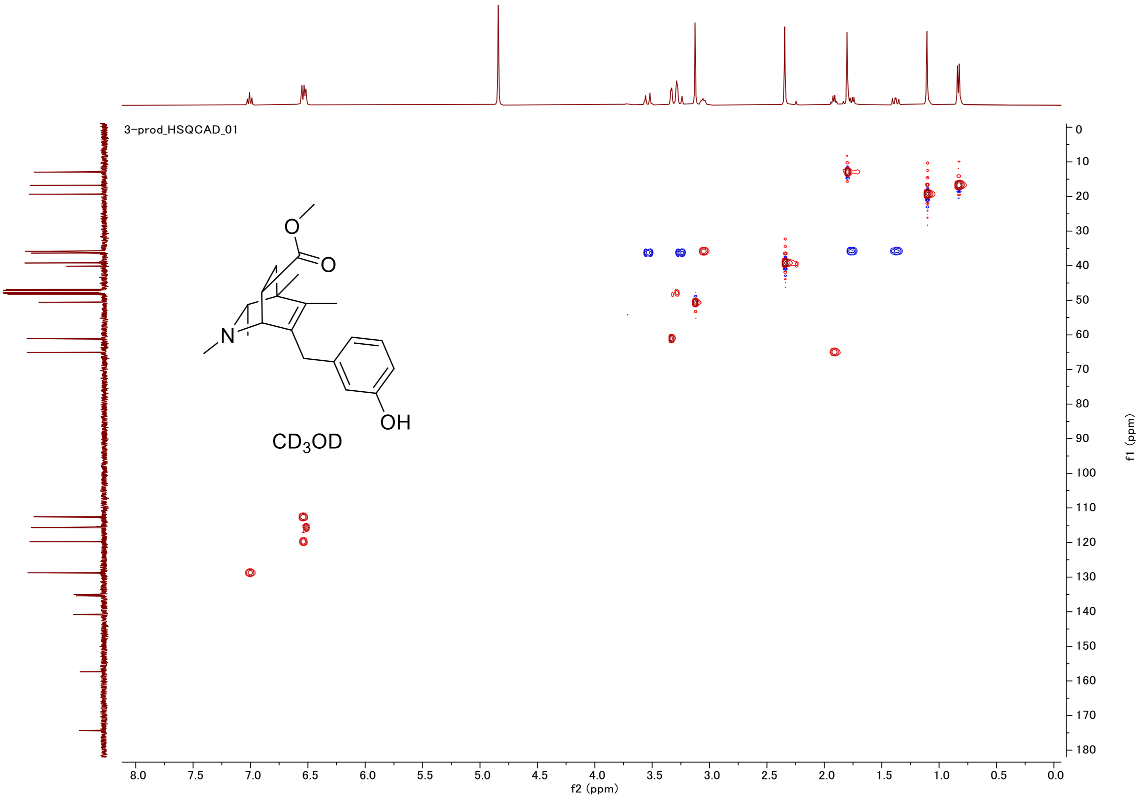
3-prod_HSQCAD_01
O
O
N
OH
CD3OD
f2 (ppm)
f1 (ppm)

Proton (#028)

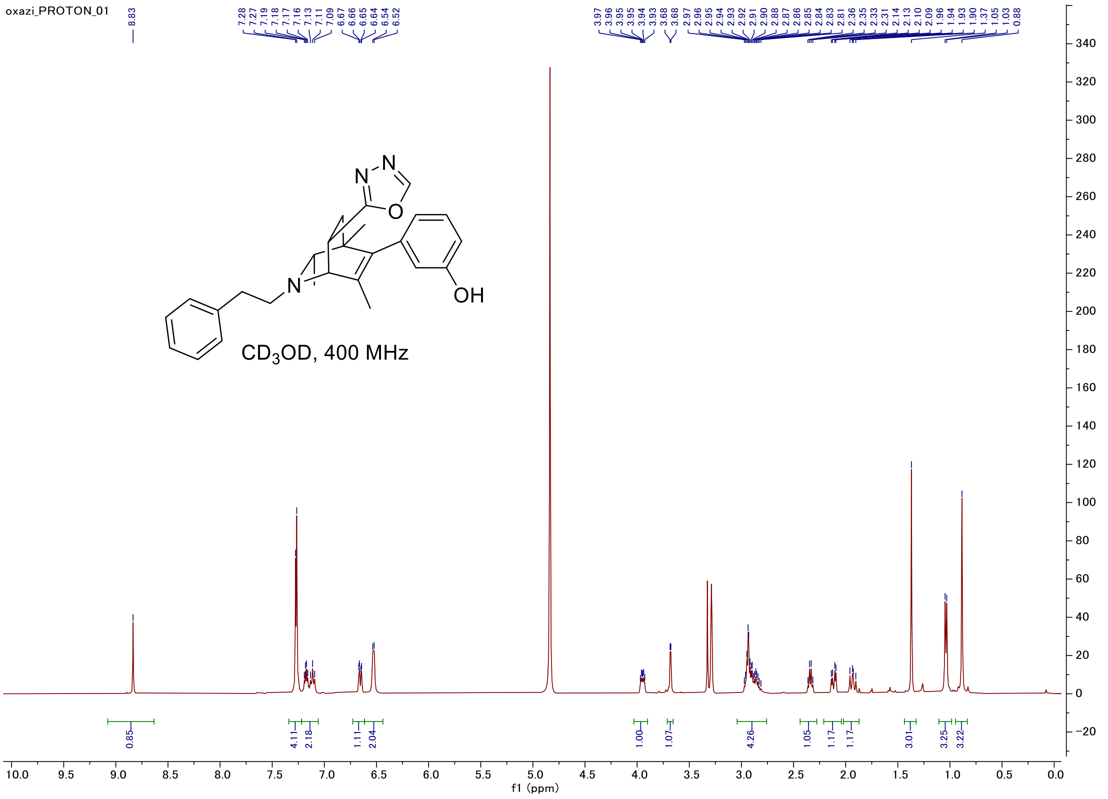

Carbon

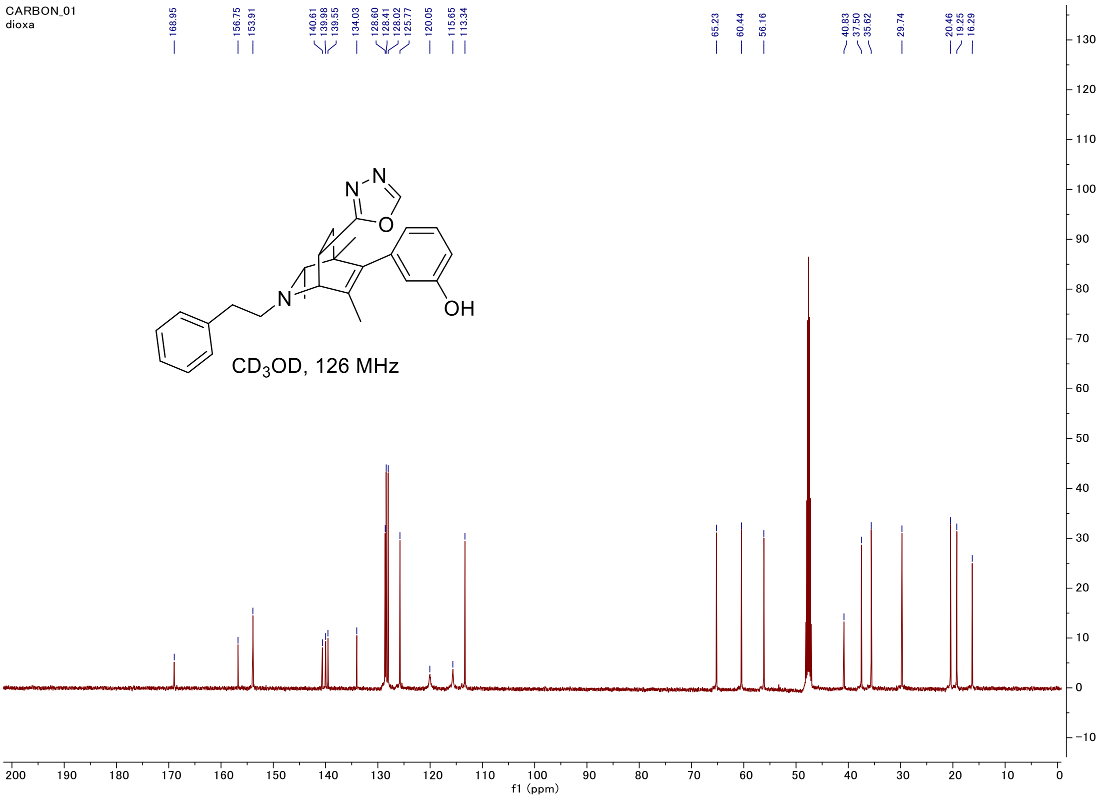

C-H COSY

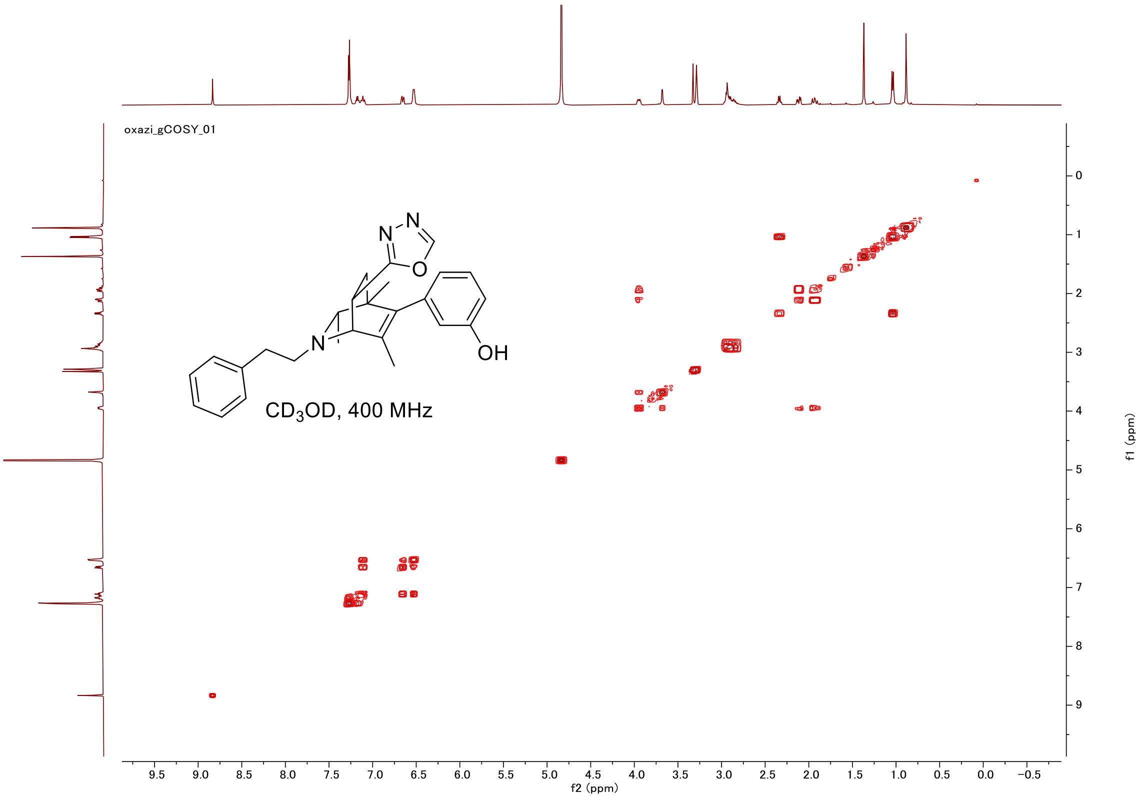
oxazi_gCOSY_01
N
N
O
N
OH
CD3OD, 400 MHz
f2 (ppm)
f1 (ppm)

HSQC

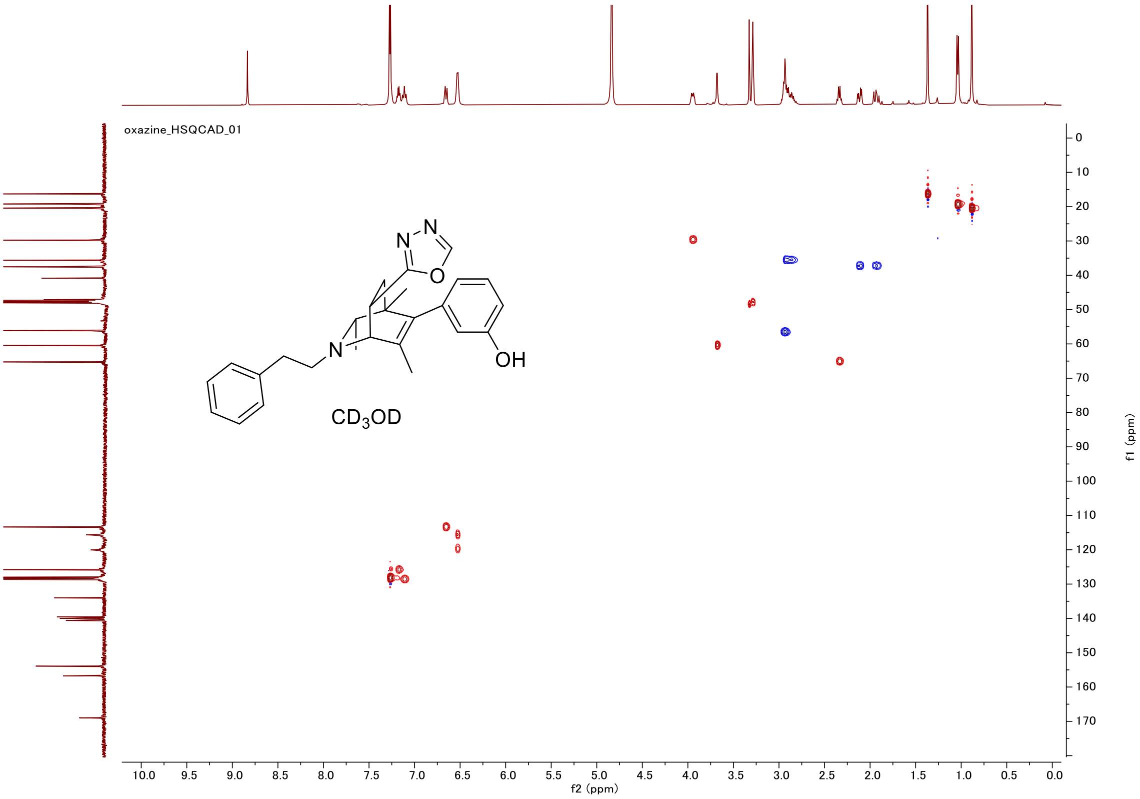
oxazine_HSQCAD_01
N
N
O
N
OH
CD3OD
f2 (ppm)
f1 (ppm)

Proton (#029)

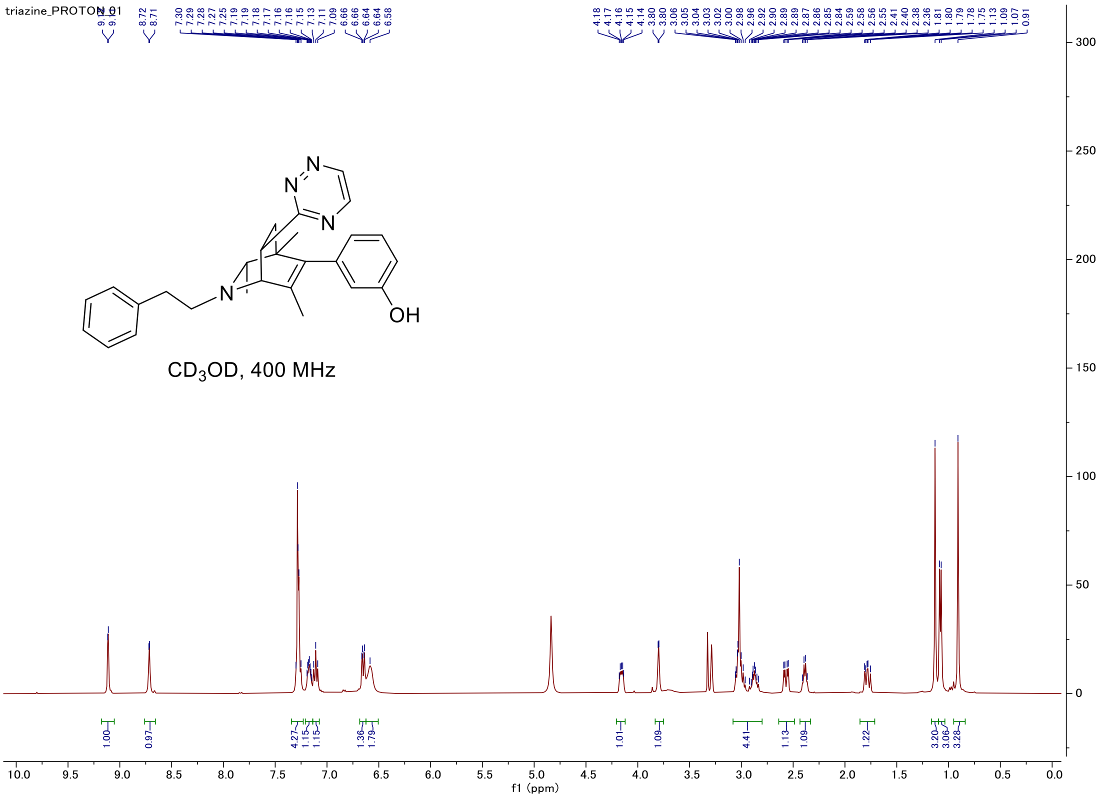

Carbon

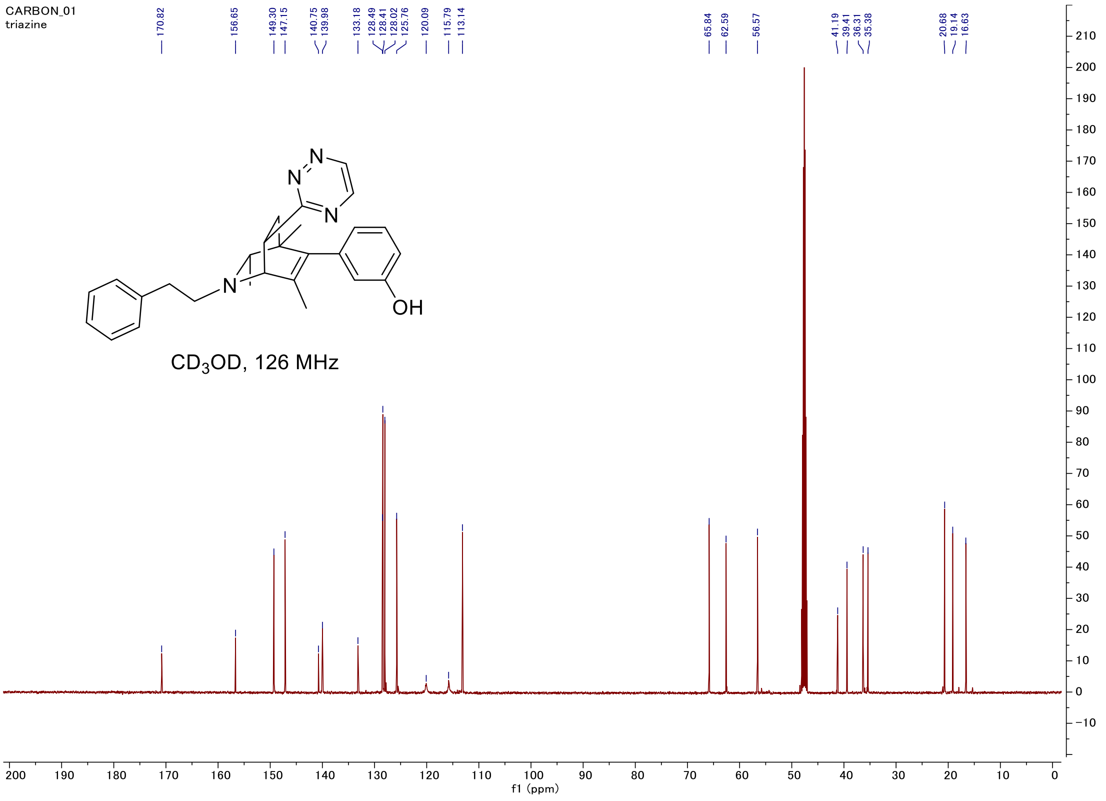

C-H COSY

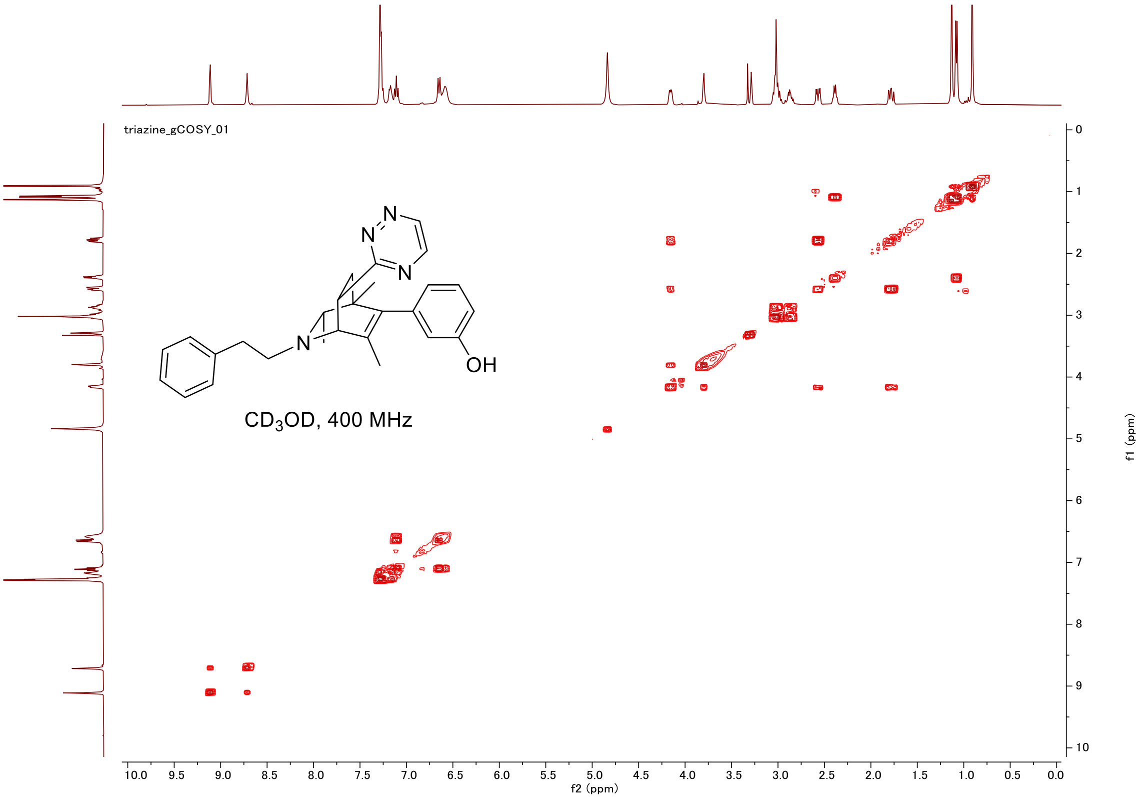
triazine_gCOSY_01
N
N
N
N
OH
CD$_3$OD, 400 MHz
f2 (ppm)
f1 (ppm)

HSQC

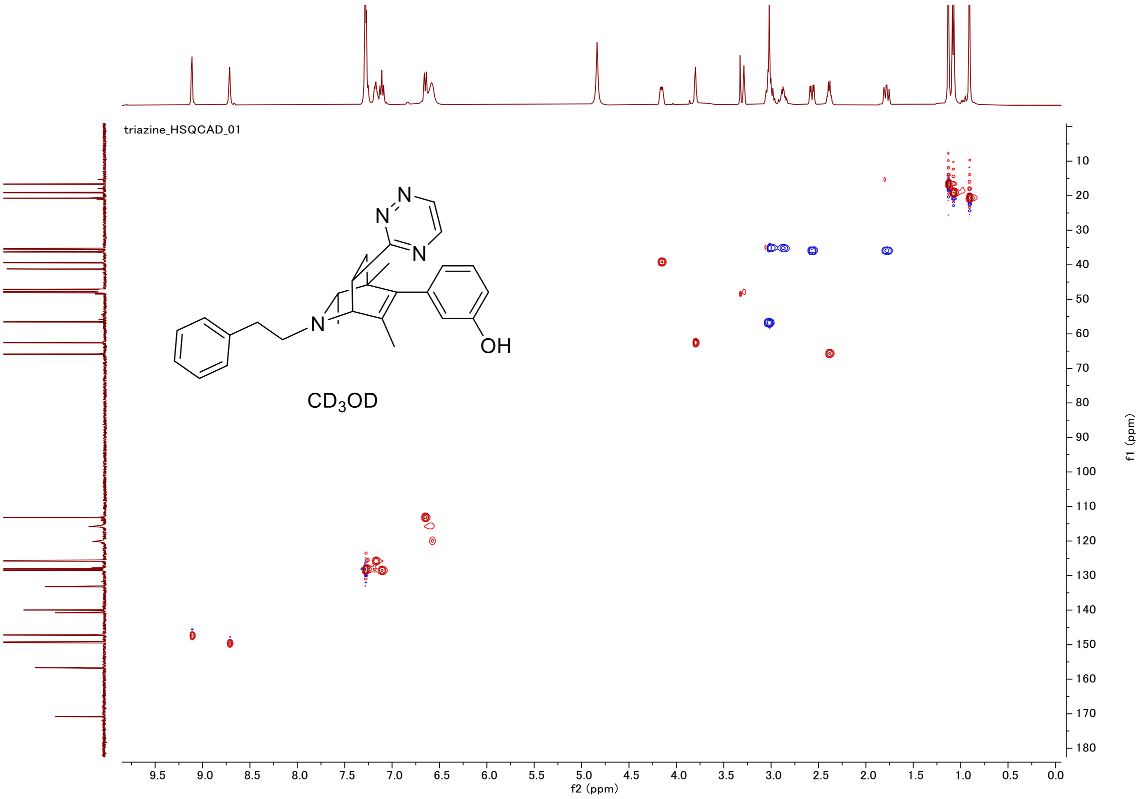
triazine_HSQCAD_01
N
N
N
N
OH
CD$_3$OD
f2 (ppm)
f1 (ppm)

Proton (#030)

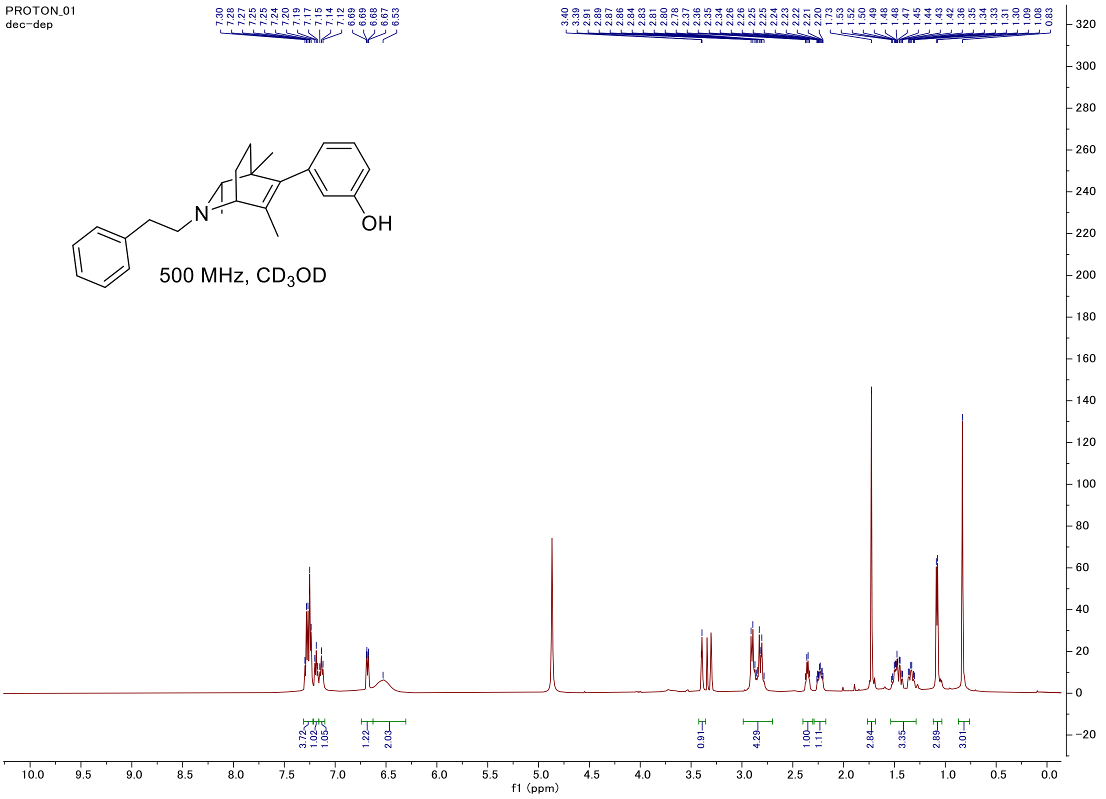

Carbon

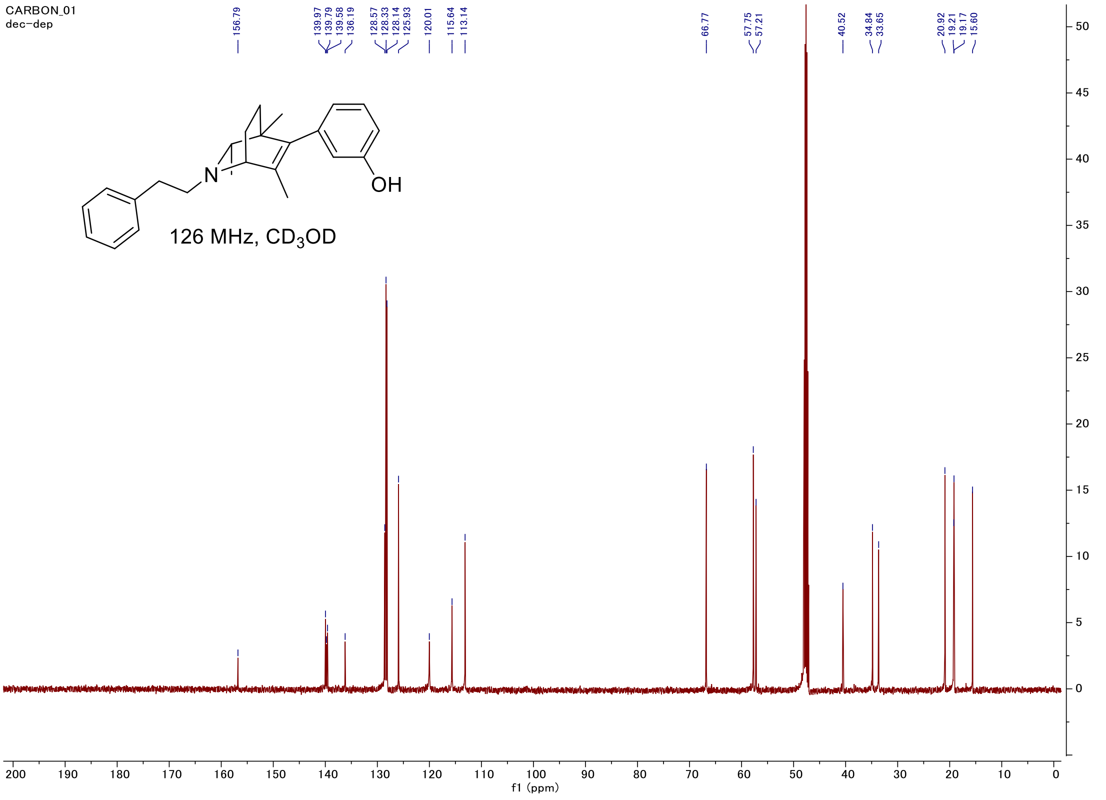

C-H COSY

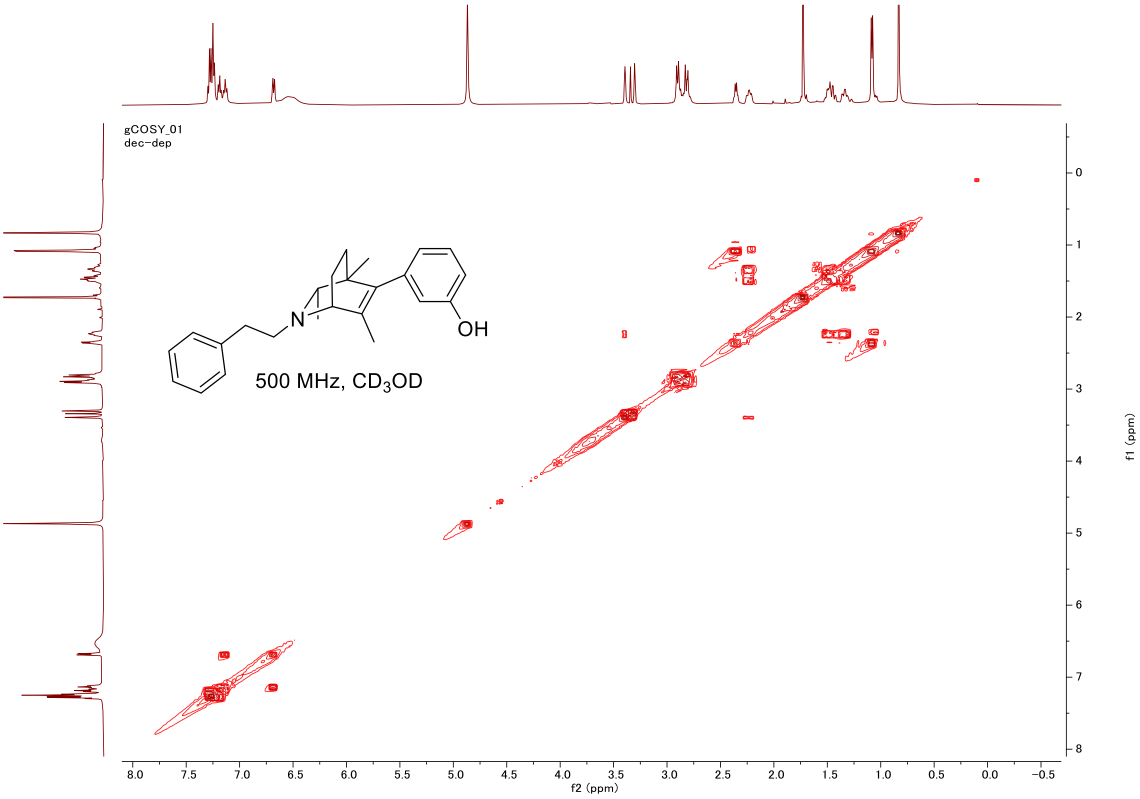
gCOSY_01
dec-dep
N
OH
500 MHz, CD_3OD
f2 (ppm)
f1 (ppm)

HSQC

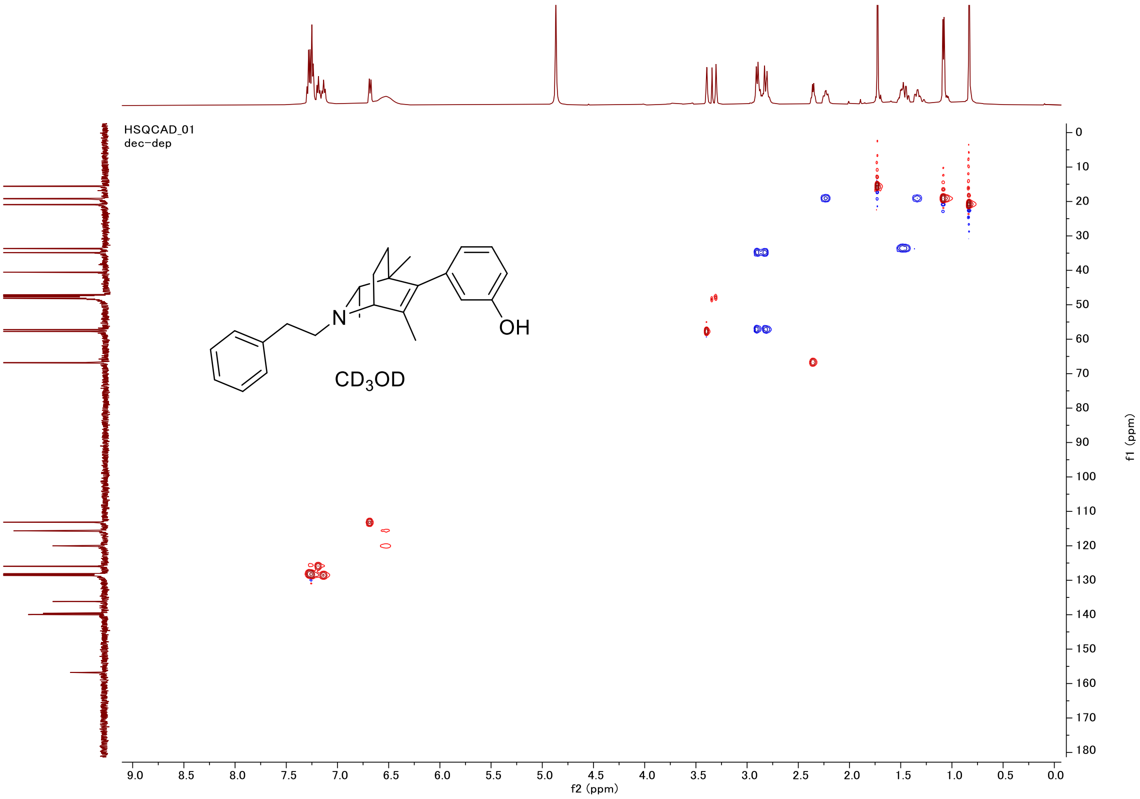
HSQCAD_01
dec-dep
N
OH
CD_3OD
f2 (ppm)
f1 (ppm)

Proton (#031)

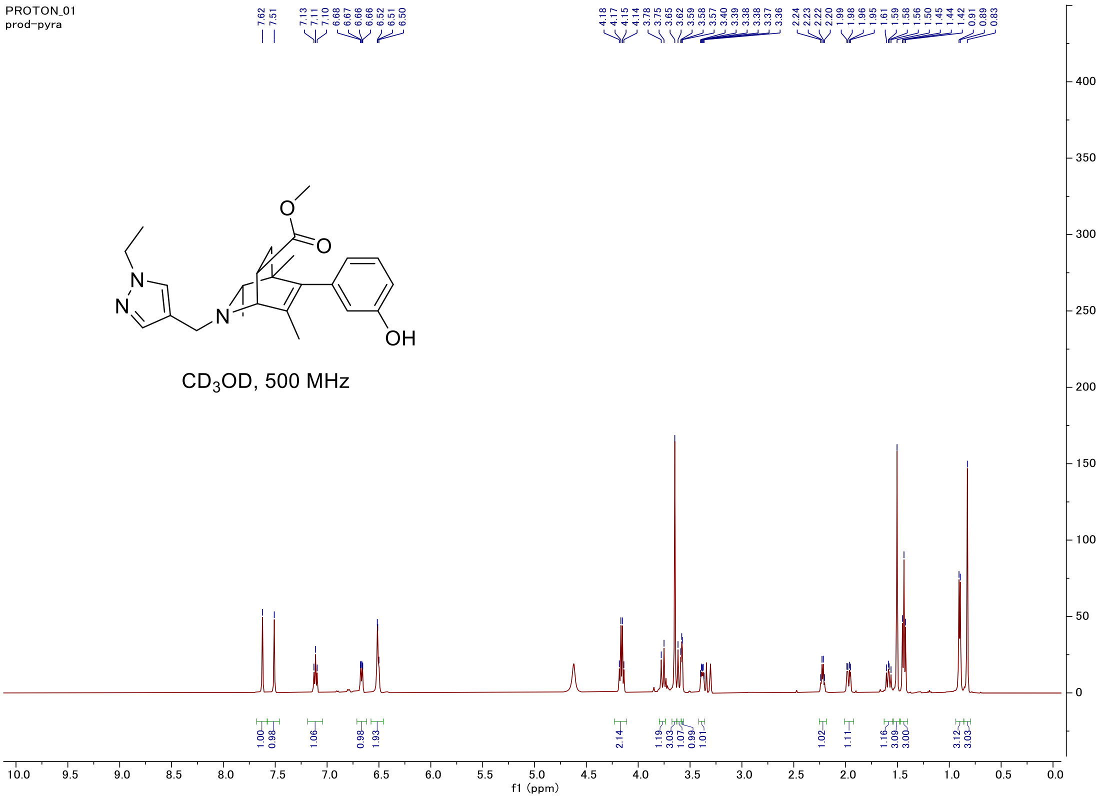

Carbon

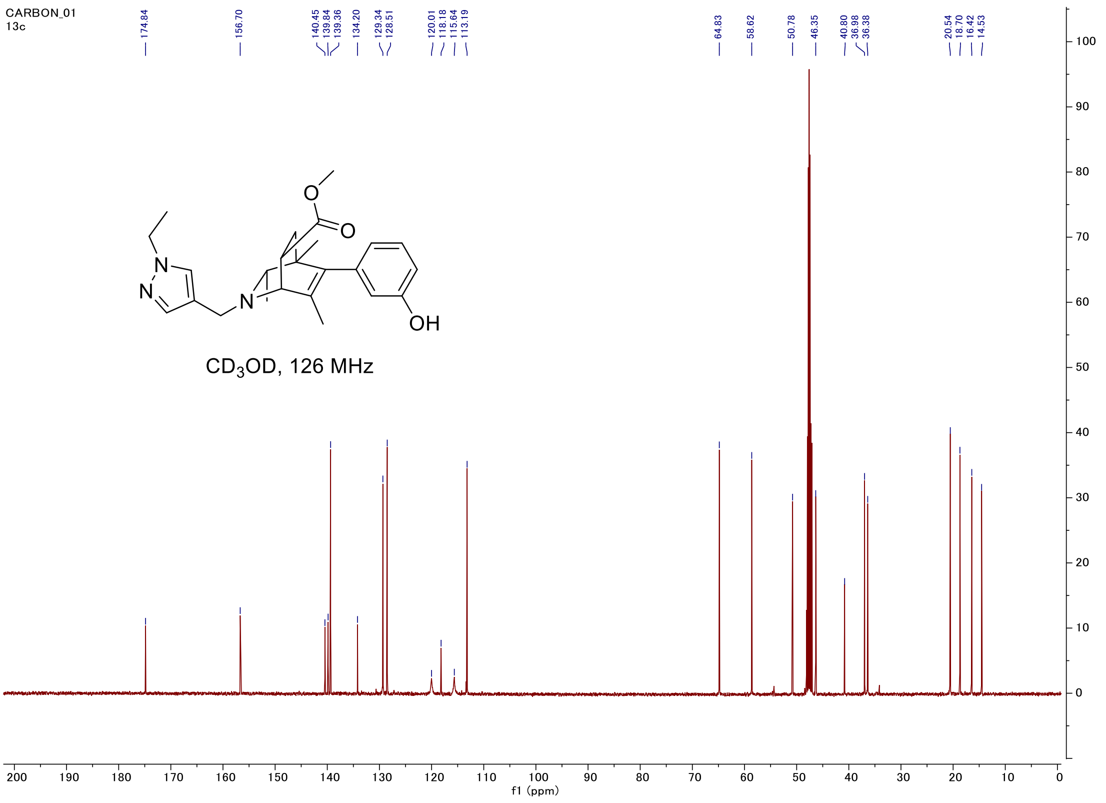

C-H COSY

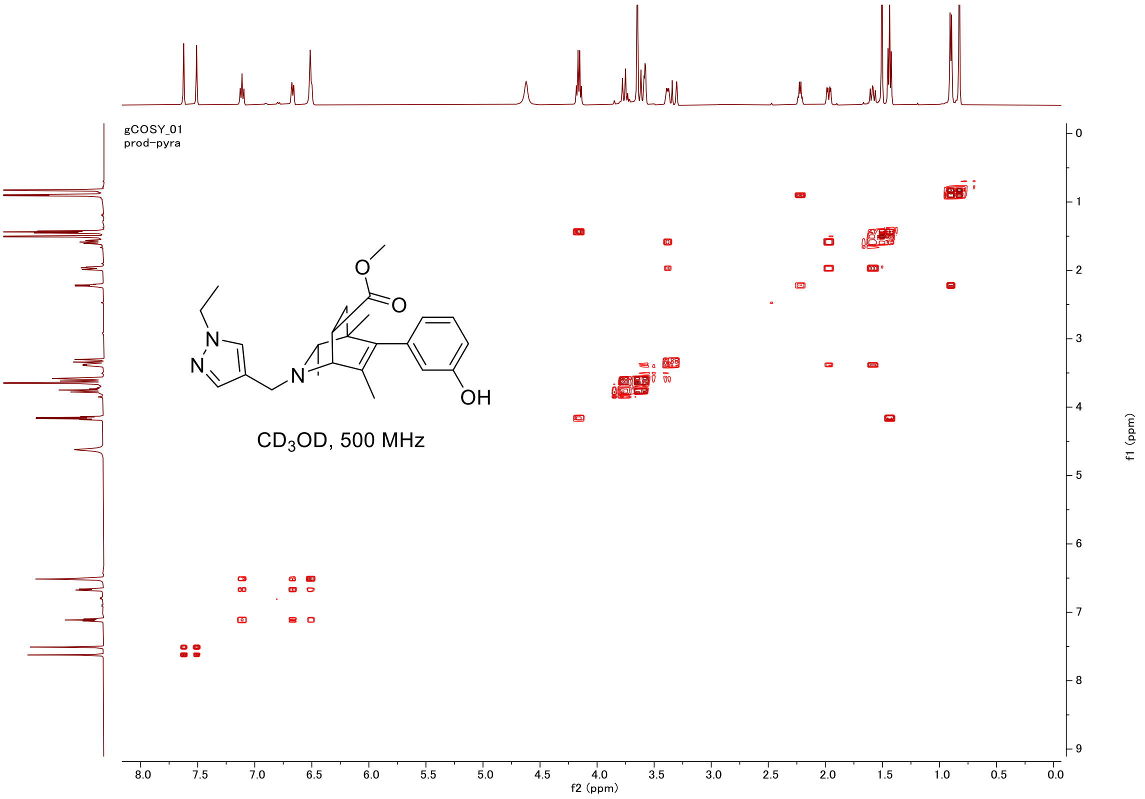
gCOSY_01
prod-pyra
CD3OD, 500 MHz
OH
f2 (ppm)
f1 (ppm)

HSQC

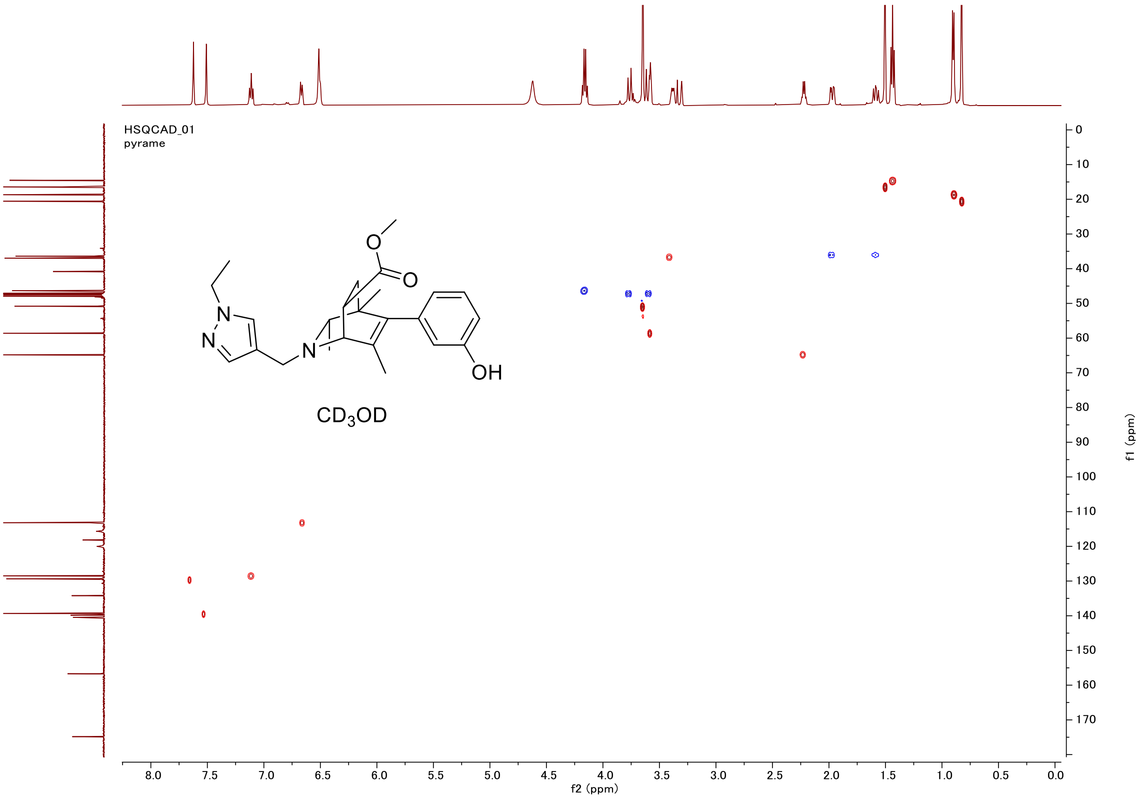
HSQCAD_01
pyrame
CD3OD
OH
f2 (ppm)
f1 (ppm)

Proton (#036)

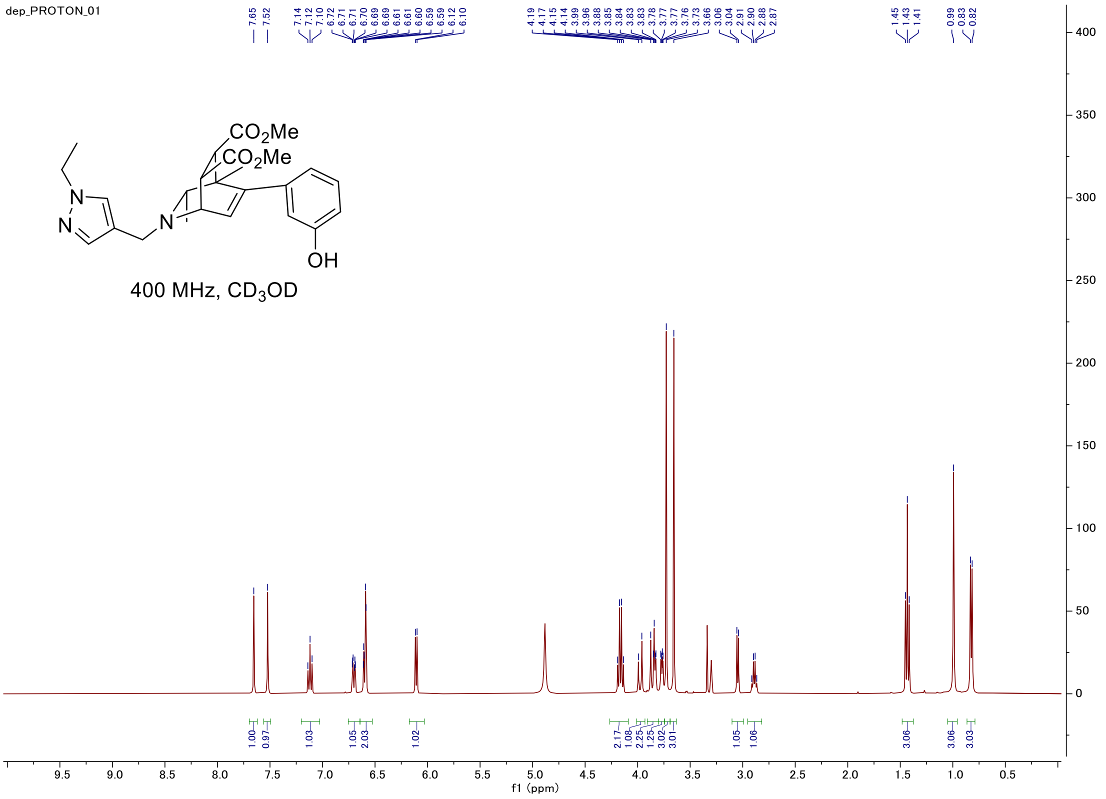

Carbon

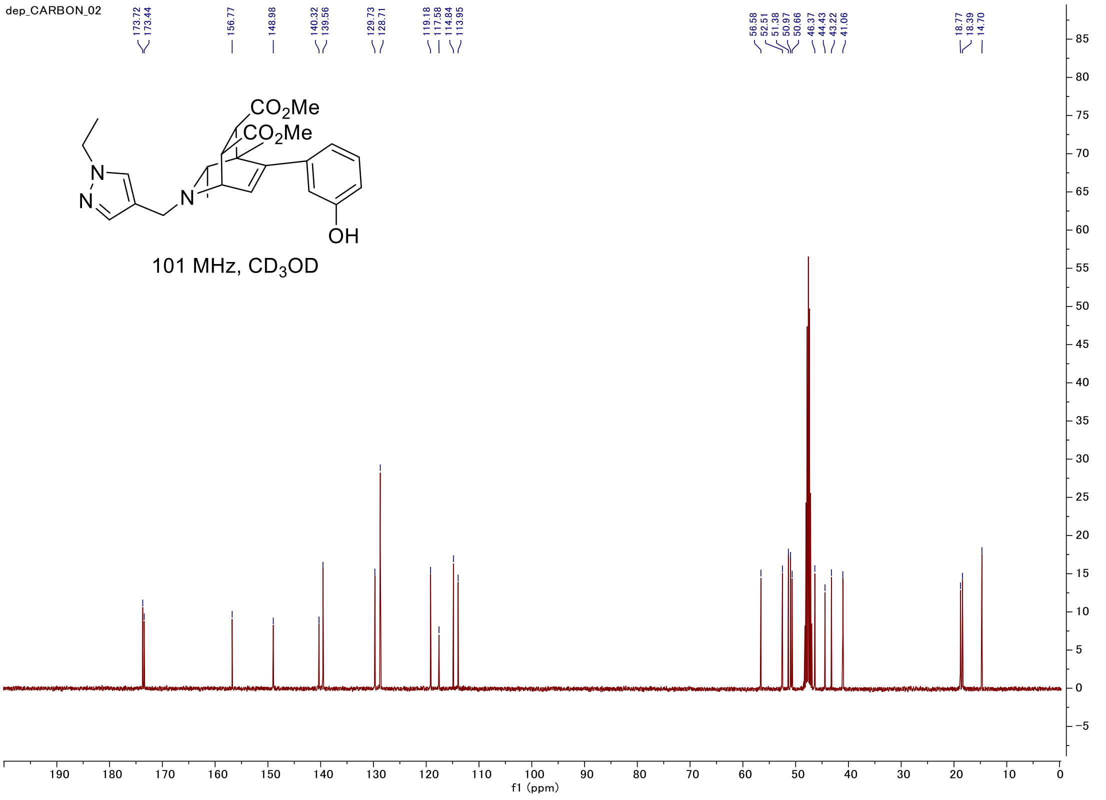

C-H COSY

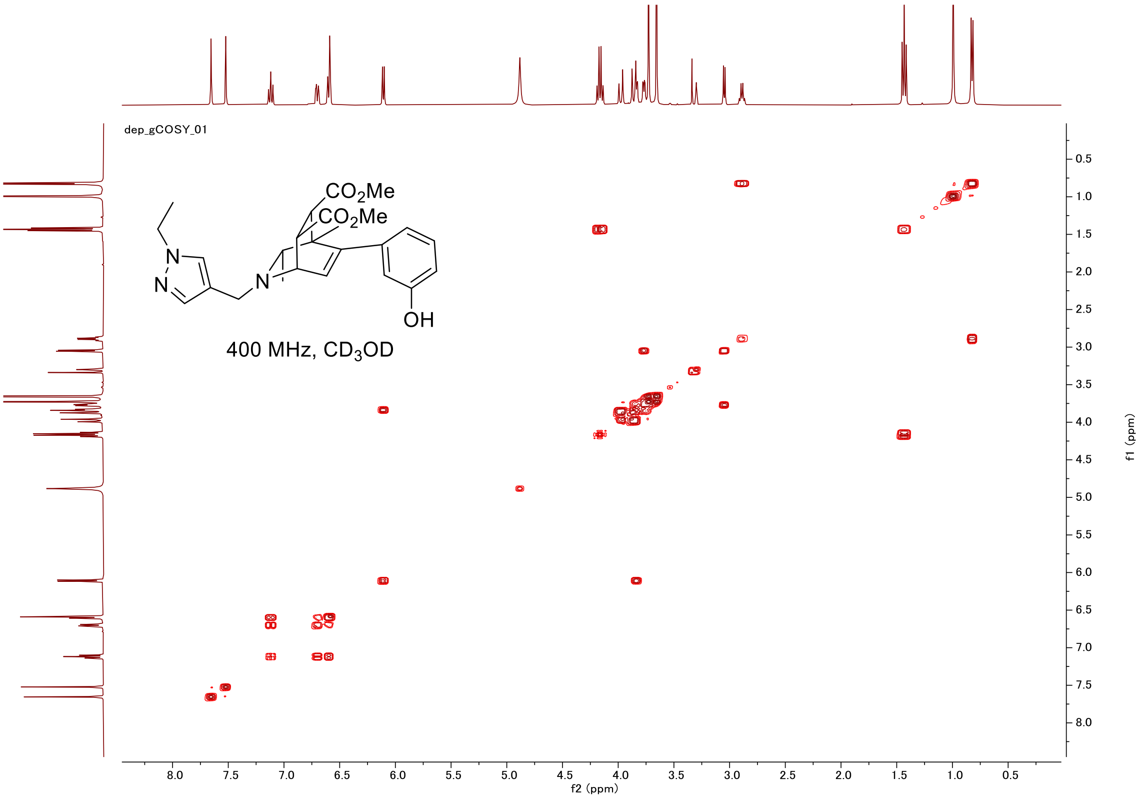
dep_gCOSY_01
CO2Me
CO2Me
N
N
N
OH
400 MHz, CD3OD
f2 (ppm)
f1 (ppm)

HSQC

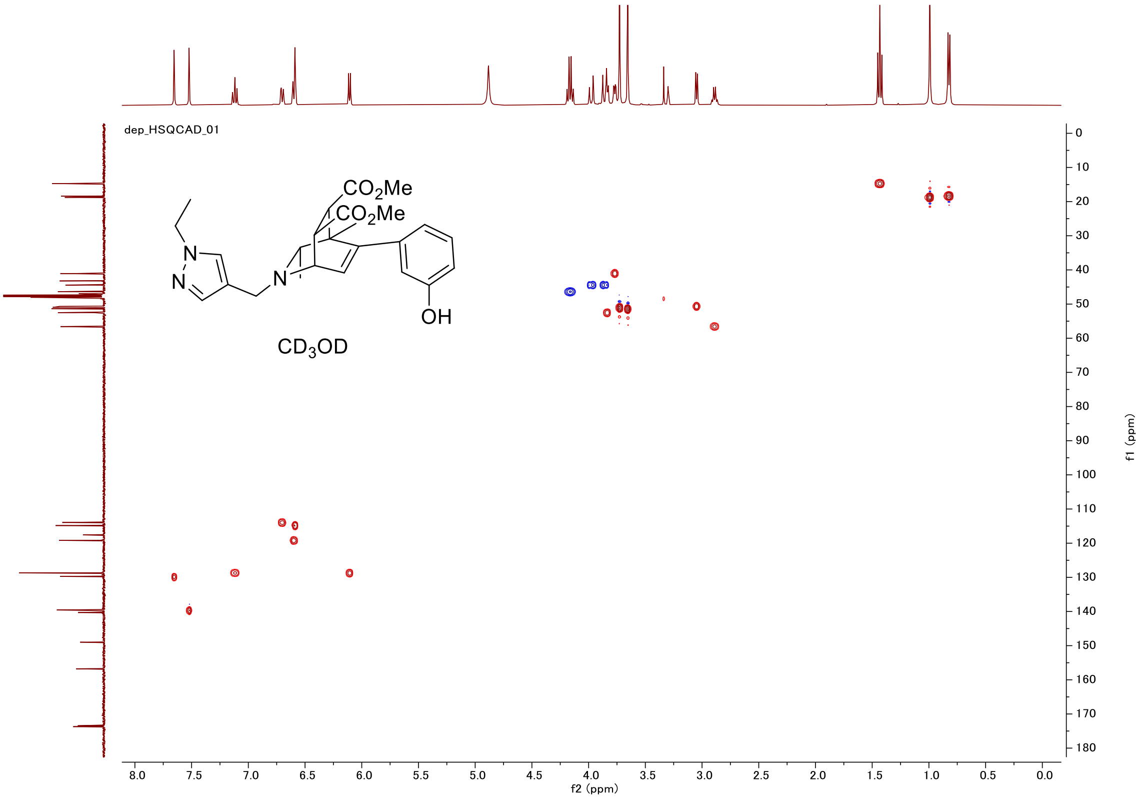
dep_HSQCAD_01
CO2Me
CO2Me
N
N
N
OH
CD3OD
f2 (ppm)
f1 (ppm)
